# Lipophilic Nucleoside Triphosphate Prodrugs of Anti‐HIV Active Nucleoside Analogs as Potential Antiviral Compounds

**DOI:** 10.1002/advs.202306021

**Published:** 2023-10-26

**Authors:** Xiao Jia, Dominique Schols, Chris Meier

**Affiliations:** ^1^ Organic Chemistry Department of Chemistry Faculty of Mathematics, Informatics and Natural Sciences Universität Hamburg Martin‐Luther‐King‐Platz 6 D‐20146 Hamburg Germany; ^2^ Laboratory of Virology and Chemotherapy Department of Microbiology and Immunology and Transplantation Rega Institute for Medical Research KU Leuven, Herestraat 49 Leuven B‐3000 Belgium

**Keywords:** antiviral activity, nucleoside analog, nucleoside diphosphates, nucleoside triphosphates, prodrugs

## Abstract

Nucleoside analogs require three phosphorylation steps catalyzed by cellular kinases to give their triphosphorylated metabolites. Herein, the synthesis of two types of triphosphate prodrugs of different nucleoside analogs is disclosed. Triphosphates comprising: i) a γ‐phosphate or γ‐phosphonate bearing a bioreversible acyloxybenzyl group and a long alkyl group and ii) γ‐dialkyl phosphate/phosphonate modified nucleoside triphosphate analogs. Almost selective conversion of the former Tri*PPP*ro‐compounds into the corresponding γ‐alkylated nucleoside triphosphate derivatives is demonstrated in CEM/0 cell extracts that proved to be stable toward further hydrolysis. The latter γ‐dialkylated triphosphate derivatives lead to the slow formation of the corresponding NDPs. Both types of Tri*PPP*ro‐compounds are highly potent in wild‐type CEM/0 cells and more importantly, they exhibit even better activities against HIV‐2 replication in CEM/TK^−^ cell cultures. A finding of major importance is that, in primer extension assays, γ‐phosphate‐modified‐NTPs, γ‐mono‐alkylated‐triphosphates, and NDPs prove to be substrates for HIV‐RT but not for cellular DNA‐polymerases α,γ.

## Graphical abstract

Two types of Tri*PPP*ro‐nucleotide prodrugs bearing different nucleoside analogs are described. These compounds exhibit high resistance to hydrolysis in CEM/0 cells extracts. Both γ‐(di and mono)‐alkylated‐triphosphates serve as substrates for HIV‐RT but not for cellular DNA‐polymerases α,γ. Remarkably, such Tri*PPP*ro‐prodrugs display high antiviral activity in wild‐type (CEM/0) and CEM/TK^−^ cell cultures, in contrast to their parent nucleoside analogs.

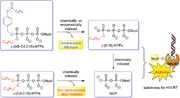

## 1 Introduction

The human immunodeficiency virus (HIV) is a retrovirus that infects and destroys the immune cells that lead to acquired immune deficiency syndrome (AIDS).^[^ ^1^, ^2^, ^3^, ^4^ ^]^ According to the World Health Organization (WHO) on HIV/AIDS report 2022 ≈39 million people are living with HIV/AIDS, including 1.3 million new infections and 0.63 million people died from HIV/AIDS‐related diseases, especially in third‐world countries. Although the HIV pandemic continues, the extraordinary progress in HIV research, especially regarding the development of the combination antiretroviral chemotherapy (cART) that targets multiple steps in the replication cycle of the virus, has proven to dramatically decrease HIV‐associated morbidity and mortality.^[^ ^5^, ^6^, ^7^, ^8^, ^9^ ^]^ Among the Food and Drug Administration (FDA)‐approved HIV drugs, nucleoside reverse transcriptase inhibitors (NRTIs)^[^ ^10^ ^]^ remain the backbone of the current cART. After being processed to their bioactive nucleoside analog triphosphates (NTPs), they target the viral DNA‐ or RNA polymerase, such as HCV‐encoded RNA‐dependent RNA‐polymerase NS5B,^[^ ^11^ ^]^ or HIV reverse transcriptase (HIV‐RT).^[^ ^12^, ^13^ ^]^ These NTPs compete with the natural nucleotides for their incorporation into a growing viral DNA strand to exert the antiviral effect mainly by acting as chain terminators.^[^ ^14^ ^]^ Till now, a significant number of nucleoside analogs are still in clinical use for the treatment of several viral infections (e.g., caused by HIV, hepatitis B, and C viruses, influenza, herpes virus, or SARS‐CoV‐2).^[^ ^11^, ^12^, ^15^, ^16^, ^17^, ^18^ ^]^

**Figure 1:**
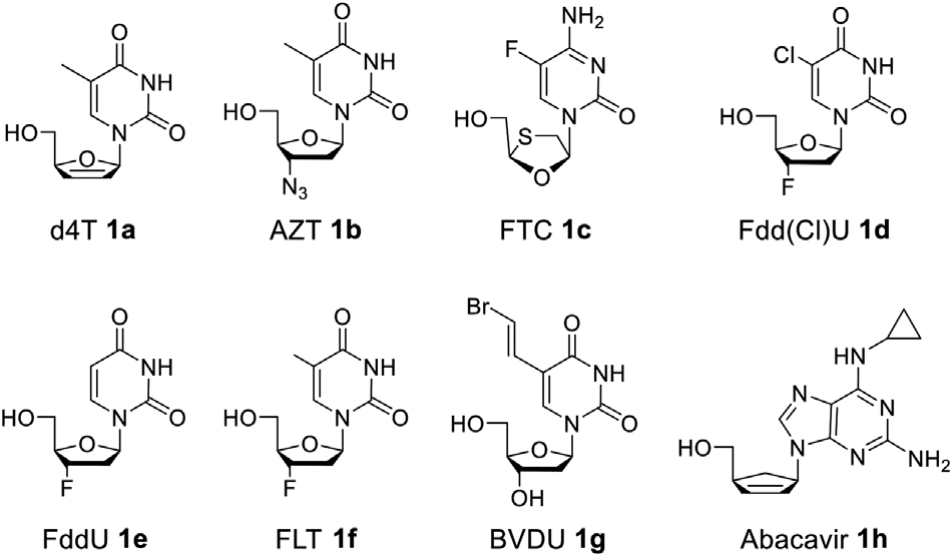
Chemical structures of nucleoside analogs used in this study.

**Scheme 1:**
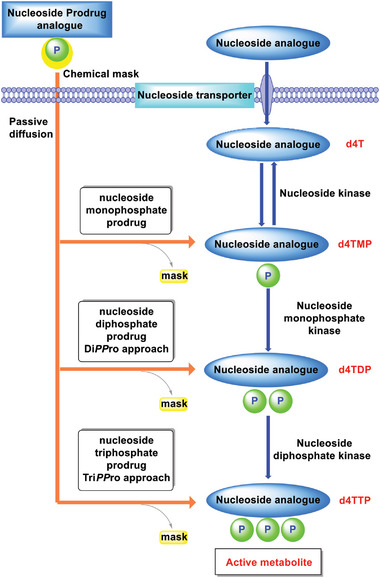
General metabolic pathway of nucleoside analogs (d4T as an example) and the different nucleotide prodrug approaches.

Nucleoside analogs as shown in **Figure** **1** depend on three cellular kinases to undergo the stepwise addition of phosphate units to form sequentially the nucleoside monophosphate (NMP), the nucleoside diphosphate (NDP), and the corresponding active nucleoside triphosphate (NTP).^[^ ^19^, ^20^, ^21^, ^22^ ^]^ However, this conversion often occurs insufficiently with the result of low therapeutic efficiency (**Scheme** **1**). For d4T,^[^ ^20^, ^23^, ^24^ ^]^ the first phosphorylation is metabolism‐limiting, whereas for AZT^[^ ^19^, ^25^ ^]^ and FTC^[^ ^26^, ^27^ ^]^ the rate‐limiting steps are the second and third phosphorylation, respectively. To overcome these phosphorylation limitations, several prodrug strategies, such as nucleoside monophosphate prodrugs (e.g., *cyclo*Sal, HepDirect, SATE‐, bis(AB)‐nucleotides, bis(POM or POC)‐nucleotides, DTE and ProTide)^[^ ^28^, ^29^, ^30^, ^31^, ^32^, ^33^, ^34^, ^35^, ^36^, ^37^, ^38^ ^]^ and nucleoside diphosphate prodrugs (Di*PP*ro‐approach),^[^ ^39^, ^40^, ^41^, ^42^, ^43^, ^44^, ^45^, ^46^, ^47^, ^48^, ^49^ ^]^ have been explored over the past decades (Scheme 1).

**Scheme 2:**
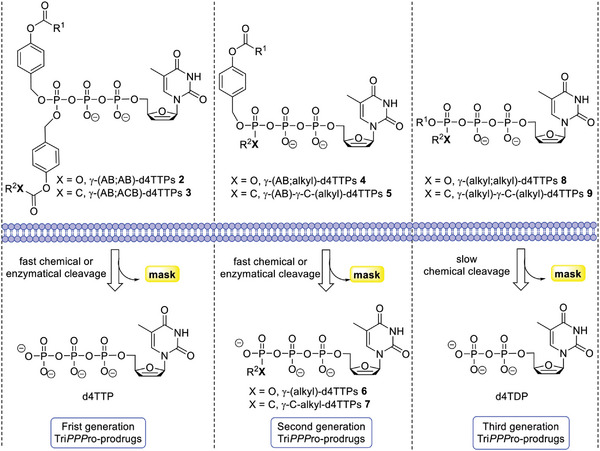
General representation of intracellular activation of different Tri*PPP*ro‐prodrugs.

In 2015, we reported on the development of the first nucleoside triphosphate prodrugs system (Tri*PPP*ro‐approach, compounds 2–9; **Scheme** **2**) for the intracellular NTP delivery^[^ ^50^, ^51^, ^52^, ^53^, ^54^, ^55^, ^56^, ^57^, ^58^, ^59^, ^60^, ^61^ ^]^ comprising two biodegradable masking groups (acyloxybenzyl‐ (AB; ester) or alkoxycarbonyloxybenzyl‐ (ACB; carbonate)) attached to the γ‐phosphate group.^[^ ^50^, ^51^, ^52^, ^53^, ^54^, ^55^ ^]^ In the case of γ‐symmetrically modified nucleoside triphosphate prodrugs 2^[^ ^50^ ^]^ and γ‐non‐symmetric nucleoside triphosphate prodrugs 3,^[^ ^54^ ^]^ the successful formation of the corresponding NTPs was detected in all hydrolysis studies and the intracellular delivery was confirmed by an uptake study using a fluorescent nucleoside triphosphate prodrug.^[^ ^51^ ^]^ Interestingly, some HIV‐inactive nucleoside analogs (e.g., Fdd(Cl)U 1d and the originally anti‐herpes virus active BVDU 1g were converted into potent anti‐HIV active compounds,^[^ ^51^, ^55^ ^]^ proving the high potential of the Tri*PPP*ro‐approach. Later, we disclosed the second generation of Tri*PPP*ro‐compounds 4,5 in which one biodegradable prodrug moiety (AB or ACB) is attached at the γ‐phosphate^[^ ^56^, ^57^ ^]^ or γ‐phosphonate^[^ ^58^ ^]^ unit, respectively, in addition to a non‐cleavable moiety and d4T as nucleoside analog (Scheme 2). We proved that those Tri*PPP*ro‐compounds 4,5 were cleaved selectively by chemical hydrolysis, in incubation studies with pig liver esterase (PLE) as well as human CD4^+^ T‐lymphocyte CEM/0 cell extracts and led to highly stable γ‐alkyl‐modified nucleoside triphosphates 6^[^ ^56^ ^]^ (CEM, *t*_1/2_ >30 h) or γ‐C‐alkyl‐modified nucleoside triphosphates 7^[^ ^58^ ^]^ (CEM, *t*_1/2_ >30 h), respectively. A finding of major importance was that, in primer extension assays, both γ‐(alkyl)‐d4TTPs 6 and γ‐C‐(alkyl)‐d4TTPs 7 proved to be substrates for HIV‐RT and d4TMP was incorporated into a DNA primer strand.^[^ ^56^, ^58^ ^]^ Tri*PPP*ro‐compounds 4,5 showed a 1000‐fold higher antiviral activity as compared to the parent d4T 1a against HIV‐2 in thymidine kinase‐deficient CD4^+^ T‐cells (CEM/TK^−^).

Furthermore, a potential new generation of γ‐dialkylphosphate‐modified‐d4TDPs 8 (CEM, *t*_1/2_ >10 h) and γ‐dialkylphosphonate‐modified‐d4TDPs 9 (CEM, *t*_1/2_ >15 h) was discovered, that comprised two non‐cleavable moieties at the γ‐phosphate group or γ‐phosphonate group, respectively (Scheme 2).^[^ ^61^ ^]^ It is worth noting that these Tri*PPP*ro‐compounds 8,9 showed very good antiviral activity (EC_50_: 0.036 µm) in infected wild‐type CEM/0 cells that was completely retained (EC_50_: 0.0050 µm, 10 000‐fold more active as d4T) in HIV‐infected CEM/TK^−^ cells. More interestingly, it was demonstrated that in addition to d4TTP also d4TDP was accepted by HIV‐RT as a substrate.^[^ ^61^ ^]^

Guided by the previous results from Tri*PPP*ro‐d4TTPs 2–9, we synthesized a series of Tri*PPP*ro‐prodrugs 10 bearing different nucleoside analogs. The chemical formulae of the nucleoside analogs used in our studies are shown in Figure 1. All Tri*PPP*ro‐compounds 10 were studied with regard to their chemical and biological stability. In addition, a few γ‐dialkylphosphate‐modified‐NTPs 10 and γ‐mono‐modified‐NTPs 20 were prepared as well for the primer extension experiments and to further study the hydrolysis properties and the delivery mechanism of Tri*PPP*ro‐compounds 10.

## 2 Results and Discussion

### 2.1 Synthesis of Tri*PPP*ro‐Compounds 10 and γ‐(Alkyl)‐NTPs 20

**Scheme 3:**
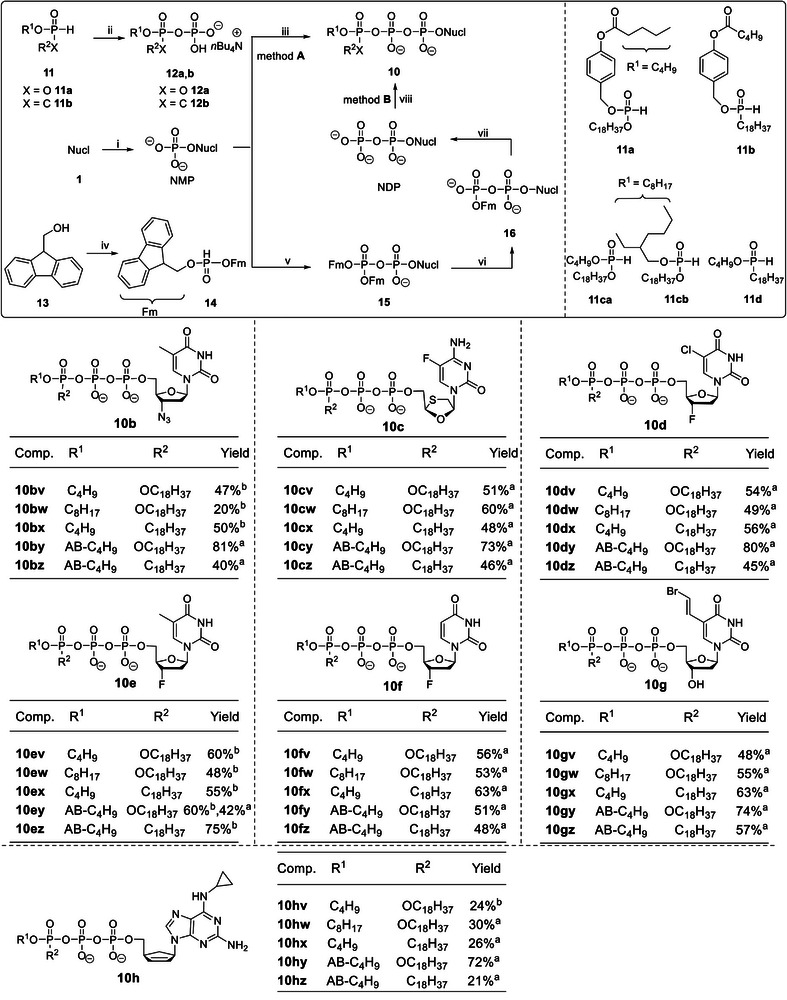
Reagents and conditions: i) a) POCl_3_/pyridine/H_2_O (2:2:1), d4T 1a, CH_3_CN, 0 °C‐rt, 5 h, 75%; b) AZT 1b, POCl_3_, proton sponge, trimethylphosphate, H_2_O, *n‐*Bu_4_N^+^OH^−^ (40% in H_2_O), 0 °C, 1–2 h, 71%; c) Nucl 1c‐h, POCl_3_, trimethylphosphate, H_2_O, *n‐*Bu_4_N^+^OH^−^ (40% in H_2_O), 0 °C, 1–2 h; ii) a) NCS, CH_3_CN or THF, rt, 2 h, b) (*n*Bu)_4_N(H_2_PO_4_), CH_3_CN, rt, 1 h; iii) a) TFAA, Et_3_N, CH_3_CN, 0 °C, 10 min, b) 1‐methylimidazole, Et_3_N, CH_3_CN, 0 °C‐rt, 10 min, c) NMPs, rt, 3–5 h, Dowex 50WX8 (NH_4_^+^ form) ion exchange; iv) DPP, pyridine, CH_2_Cl_2_, 0 °C‐rt, 12 h, 64%; v) a) NCS, THF, rt, 2 h, b) NMP, CH_3_CN, rt, 1–3 h; vi) Et_3_N, CH_3_CN/THF (1:1), 10 min; vii) H_2_O, Et_3_N, CH_3_CN, rt, 24 h, *n‐*Bu_4_N^+^OH^−^ (40% in H_2_O); viii) a) *H*‐phospho(i)nates 11, NCS, CH_3_CN or THF, rt, 2–3 h; b) NDPs, CH_3_CN, rt, 3–5 h.

**Scheme 4:**
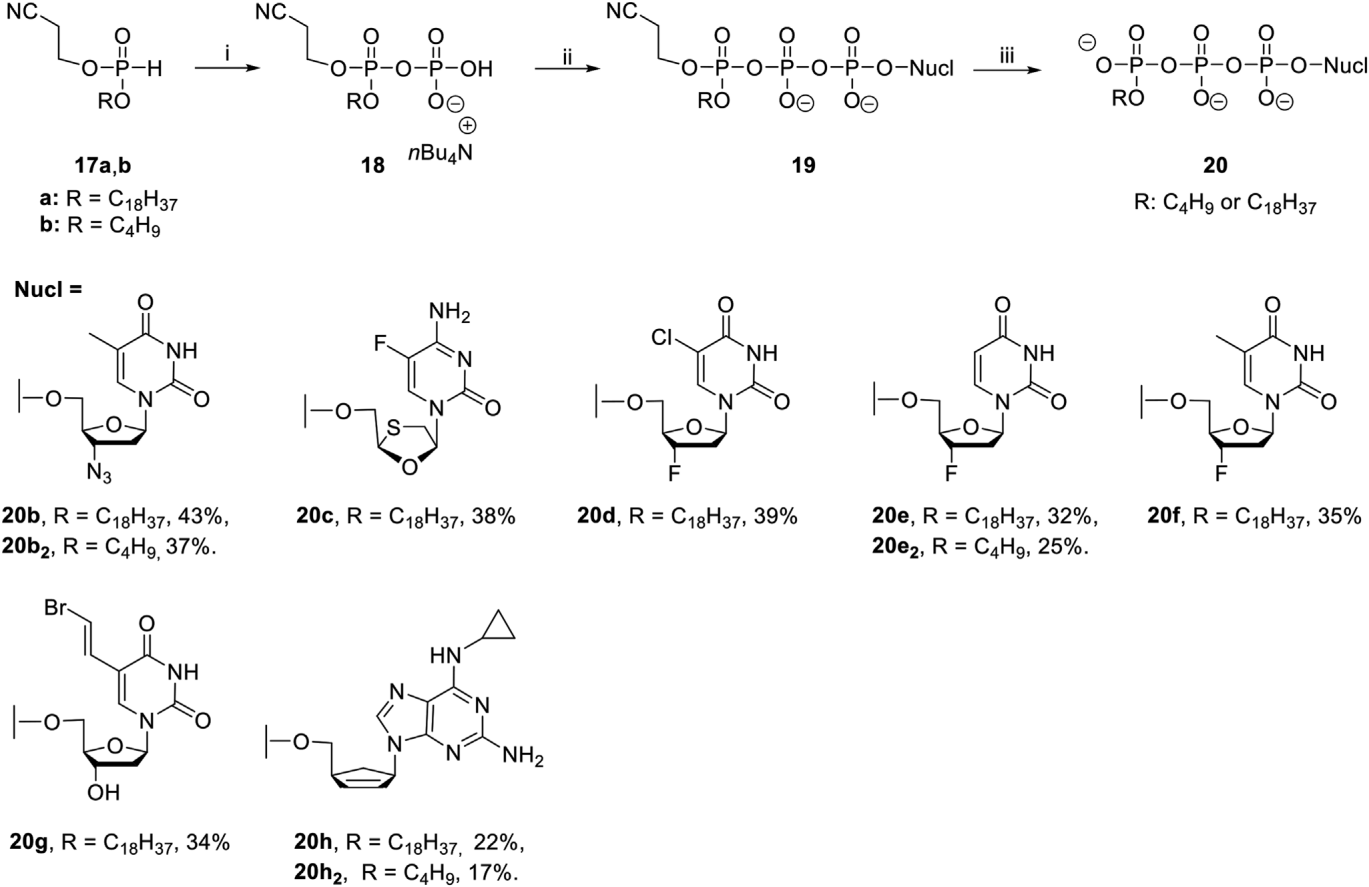
Reagents and conditions: i) a) NCS, CH_3_CN, rt, 2 h, b) (*n*Bu)_4_N(H_2_PO_4_), CH_3_CN, rt, 1 h; ii) a) TFAA, Et_3_N, CH_3_CN, 0 °C, 10 min, b) 1‐methylimidazole, Et_3_N, CH_3_CN, 0 °C‐rt, 10 min, c) NMPs, rt, 3 h; iii) *n‐*Bu_4_N^+^OH^−^ (40% in H_2_O), CH_3_CN, 8–12 h, Dowex 50WX8 (NH_4_^+^ form) ion exchange.

The recently described “*H*‐phosphonate”^[^ ^51^, ^53^, ^54^, ^55^ ^]^ or “*H*‐phosphinate route”^[^ ^58^ ^]^ were used for the synthesis of the γ‐modified nucleoside triphosphate compounds 10 (variation in the masking moiety; **Scheme** **3**). These routes were based on a coupling reaction of compounds 12 and NMPs. In the first step, diphosphates 12a or phosphonate‐phosphates 12b were prepared from compounds 11 followed by an oxidative chlorination with *N*‐chlorosuccinimide (NCS) and subsequent reaction with tetra‐*n*‐butylammonium phosphate. NMPs were synthesized in good yields using the previously reported protocols.^[^ ^62^, ^63^, ^64^ ^]^ After the final coupling, Tri*PPP*ro‐compounds 10 were successfully obtained using method A (Scheme 3) in yields of 21–81%. In addition, γ‐(β‐cyanoethyl;alkyl‐C18 or C4)‐NTPs 19 were synthesized as well using the same route. Subsequently, the β‐cyanoethyl moiety was cleaved under basic conditions to form the corresponding γ‐alkyl‐NTPs 20 in yields between 17% and 43% (variation in the nucleoside part; **Scheme** **4**). However, low yields of Tri*PPP*ro‐compounds 10, such as 10hv, were obtained without good reasons. Thus, method B was used for the synthesis of Tri*PPP*ro‐compounds 10bv–bx,10ev–ez, and 10hv that gave good yields as shown in Scheme 3. First *H*‐phosphonate 14 was prepared from 9‐fluorenylmethanol 13 and diphenyl hydrogen phosphonate (DPP). Next, compound 14 was reacted with NCS to give the phosphorochloridate. Subsequent phosphorylation of NMPs yielded the bis(Fm)‐protected diphosphate in compounds 15. Compounds 15 were hydrolyzed to form β‐Fm‐NDPs 16 with NEt_3_ (10 min) in CH_3_CN:THF (1:1). Intermediates 16 were isolated by reversed‐phase (rp) column chromatography, followed by a deprotection step to form NDPs. The final coupling reaction was accomplished by a stepwise reaction sequence using *H*‐phosph(i)onates 11 with NCS, followed by the addition of NDPs to afford Tri*PPP*ro‐compounds 10. Notably, the remaining NDP can be recycled using this strategy; thus, a more efficient conversion of the parent nucleoside to the Tri*PPP*ro‐compounds was achieved.

### 2.2 Chemical Stability and Enzymatic Activation of Tri*PPP*ro‐Compounds 10 and 20

The hydrolysis properties of Tri*PPP*ro‐compounds 10 and 20 were evaluated in phosphate buffer saline (PBS, pH 7.3), pig liver esterase (PLE) or CEM/0 cell extracts. In both cases, hydrolysis products were analyzed by means of analytical RP18‐HPLC and the hydrolysis half‐lives (**Table** **1**, *t_1/2_*) of Tri*PPP*ro‐compounds 10 and 20 were calculated after complete consumption of the starting materials.

**Table 1:** Hydrolysis half‐lives of Tri*PPP*ro‐NTPs 10 and γ‐alkyl‐NTPs 20 in PBS, PLE and CEM/0 cell extracts as well as retention time.

Comp.	PBS pH 7.3	CEM/0	PLE	RP‐HPLC	Comp.	PBS pH 7.3	CEM/0	PLE	RP‐HPLC	Comp.	PBS pH 7.3	CEM/0	PLE	RP‐HPLC
	*t*_1/2_ [h]	*t*_1/2_ [h]	*t*_1/2_ [h]	*t*_R_ (min)		*t*_1/2_ [h]	*t*_1/2_ [h]	*t*_1/2_ [h]	*t*_R_ (min)		*t*_1/2_ [h]	*t*_1/2_ [h]	*t*_1/2_ [h]	*t*_R_ (min)
10bv	>2000	>30	>150	16.1	10dz	670	>10	1.3	17.2	10gy	600	n.d.^a)^	0.3	17.7
10bw	>2000	n.d.^a)^	n.d.^a)^	17.8	10ev	>2000	>10	>150	16.4	10gz	>1500	n.d.^a)^	n.d.^a)^	17.4
10bx	>1500	>20	>150	16.3	10ew	>2000	n.d.^a)^	n.d.^a)^	17.6	10hv	253	3.0	>150	16.3
10by	418	3.3	0.22	17.6	10ex	148	n.d.^a)^	n.d.^a)^	16.1	10hw	181	n.d.^a)^	n.d.^a)^	17.7
10bz	984	8	0.25	17.3	10ey	>400	n.d.^a)^	n.d.^a)^	17.4	10hx	>1000	>10	>150	16.1
10cv	>2000	>10	>150	16.0	10ez	51	n.d.^a)^	n.d.^a)^	17.1	10hy	46	1.3	0.97	17.4
10cw	>2000	n.d.^a)^	n.d.^a)^	17.1	10fv	>2000	>10	>150	16.4	10hz	534	7.7	1.11	17.1
10cx	>2000	n.d.^a)^	n.d.^a)^	15.8	10fw	>2000	n.d.^a)^	n.d.^a)^	17.5	20b	>2000	>30	>150	14.7
10cy	251	3.4	0.24	17.0	10fx	>2000	n.d.^a)^	n.d.^a)^	16.1	20c	>2000	>30	>150	14.3
10cz	>1500	n.d.^a)^	n.d.^a)^	16.7	10fy	360	n.d.^a)^	n.d.^a)^	17.4	20d	>2000	>20	>150	14.7
10dv	>2000	>20	>150	16.5	10fz	203	n.d.^a)^	n.d.^a)^	17.1	20e	>2000	>20	>150	14.5
10dw	>2000	n.d.^a)^	n.d.^a)^	17.7	10gv	>2000	>10	>150	16.7	20f	>2000	>20	>150	14.5
10dx	>2000	n.d.^a)^	n.d.^a)^	16.2	10gw	>2000	n.d.^a)^	n.d.^a)^	17.8	20g	>2000	>20	>150	14.7
10dy	445	2.9	0.19	17.5	10gx	>1000	n.d.^a)^	n.d.^a)^	16.4	20h	>2000	>10	>150	14.7

^a)^ n.d.: not determined.

Tri*PPP*ro‐compounds 10bv‐hz: γ‐phosphate and γ‐phosphonate prodrugs. Nucleoside analogs: b: AZT; c : FTC; d: Fdd(Cl)U; e: FddU; f: FLT; g: BVDU; h: ABC. Masking groups: v: (alkyl‐C4;alkyl‐C18); w: (alkyl‐C8;alkyl‐C18); x: (alkyl‐C4)‐C‐(alkyl‐C18); y: (AB‐C4;alkyl‐C18); **z**: (AB‐C4)‐C‐(alkyl‐C18). 10bv‐hv: γ‐(alkyl‐C4;alkyl‐C18)‐NTPs; 10bw‐hw: γ‐(alkyl‐C8;alkyl‐C18)‐NTPs; 10bx‐hx: γ‐(alkyl‐C4)‐γ‐C‐(alkyl‐C18)‐NTPs; 10by‐hy: γ‐(AB‐C4;alkyl‐C18)‐NTPs; 10bz‐hz: γ‐(AB‐C4)‐γ‐C‐(alkyl‐C18)‐NTPs.

**Scheme 5:**
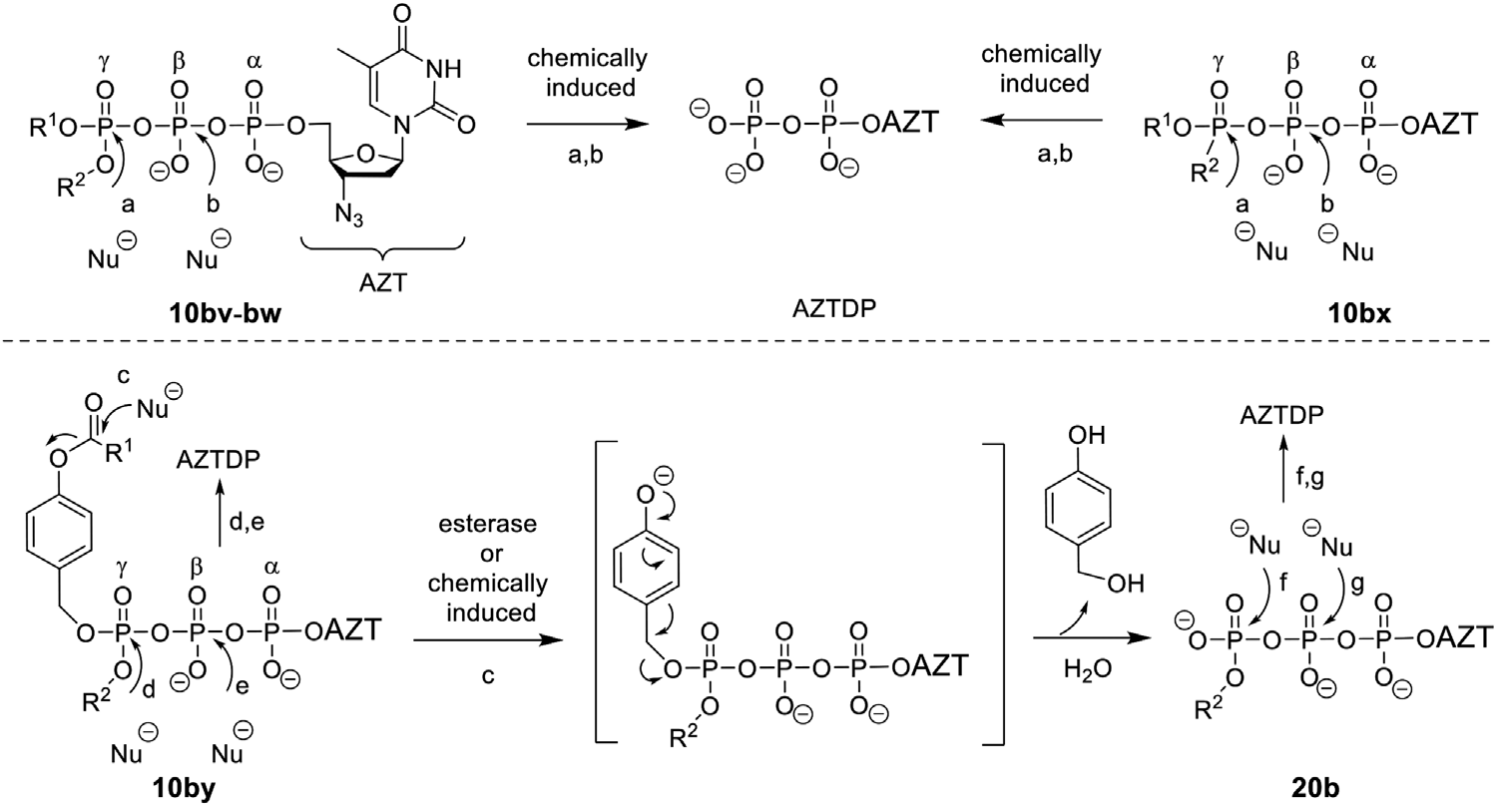
Possible hydrolysis pathways of Tri*PPP*ro‐compounds 10b and 20b.

The hydrolysis pathways of Tri*PPP*ro‐compounds 10 and 20 (**Scheme** **5**) followed mainly those of the previously reported Tri*PPP*ro‐compounds 4–9 (Scheme 2).

In PBS, generally, almost all compounds exhibited very high chemical stability at pH 7.3. The half‐lives of dialkylated γ‐(alkyl‐C4;alkyl‐C18)‐NTPs 10bv–hv (*t*_1/2_ = 253–2000 h) and γ‐(alkyl‐C4)‐γ‐C‐(alkyl‐C18)‐NTPs 10bx–hx (*t*_1/2_ = 148–2000 h) bearing two different alkyl residues were found to be higher than the corresponding alkylated and biocleavable modified γ‐(AB‐C4;alkyl‐C18)‐NTPs 10by–hy (*t*_1/2_ = 46–600 h) and γ‐(AB‐C4)‐γ‐C‐(alkyl‐C18)‐NTPs 10bz–hz (*t*_1/2_ = 51–1500 h) comprising an AB (C4) moiety in combination with a non‐biocleavable moiety (alkyl‐C18) at the γ‐phosphate or γ‐phosphonate group, respectively. Both types of Tri*PPP*ro‐compounds 10 proved more stable as compared to the chemical stabilities of γ‐(AB;ACB)‐NTPs 3 (*t*_1/2_ = 36–84 h).^[^ ^55^ ^]^

**Figure 2:**
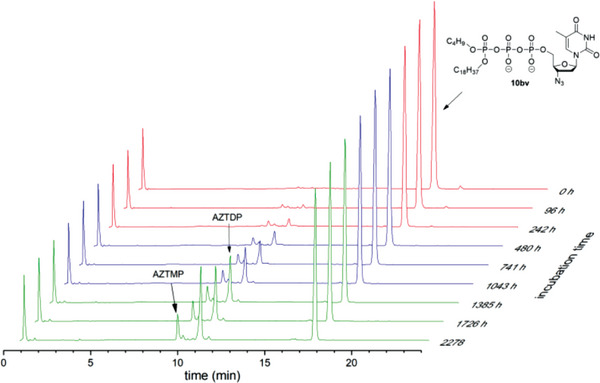
Hydrolysis of Tri*PPP*ro‐compound 10bv in PBS (pH 7.3).

In the case of Tri*PPP*ro‐compounds 10bv–hv, no NTPs or γ‐alkylated nucleoside triphosphate analogs 20 were detected in PBS, but the formation of the corresponding NDPs was observed (**Figure** **2**; Figures S1–S5, Supporting Information). Thus, the cleavage of the alkyl P─O bond in Tri*PPP*ro‐compounds 10bv–hv is impossible, but a nucleophilic reaction at the γ‐phosphate leading to NDPs occurred (pathway a, Scheme 5). In all hydrolysis studies, a very small amount of NMPs was also observed, probably due to chemical phosphoranhydride cleavage in Tri*PPP*ro‐compounds 10bv–hv (pathway b, Scheme 5) or NDPs.

**Figure 3:**
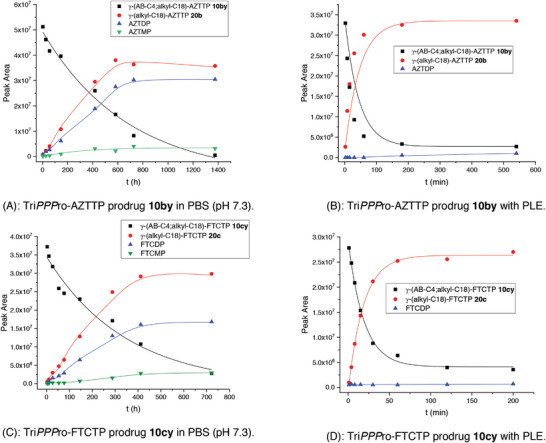
Hydrolysis of Tri*PPP*ro‐prodrugs 10by and 10cy in PBS (pH 7.3) and in PLE.

As shown in **Figure** **3**A,C and Figures S6 and S7 (Supporting information), γ‐(AB‐C4;alkyl‐C18)‐NTPs 10by–hy were mainly hydrolyzed to give the corresponding γ‐(alkyl‐C18)‐NTPs 20 (pathway c, Scheme 5) with some formation of NDPs (pathways d,e, Scheme 5) in PBS. Additionally, almost no formation of NDPs (pathways f,g, Scheme 5) was observed when γ‐(alkyl‐C18)‐NTPs 20 were hydrolyzed in PBS (Figures S8–S10, Supporting Information), potentially caused by the repulsion of the approaching nucleophile by the three negative charges present in γ‐(alkyl‐C18)‐NTPs 20. Therefore, an increase in the concentrations of γ‐(alkyl‐C18)‐NTPs 20 and NDPs were detected before complete consumption of the initial γ‐(AB‐C4;alkyl‐C18)‐NTPs 10by–hy. Interestingly, the cleavage of the masking groups in Tri*PPP*ro‐compounds 10bx–hx (Figures S11–S13, Supporting Information) proceeded similarly to the hydrolysis pathways for Tri*PPP*ro‐compounds 10bv–hv. However, in the case of γ‐(AB‐C4)‐γ‐C‐(alkyl‐C18)‐AZTTP 10bz (Figure S14, Supporting Information) the concentration of AZTDP was higher than that of the phosphonate analog to compound 20b (γ‐C‐(alkyl‐C18)‐AZTTP) indicating that the hydrolysis of Tri*PPP*ro‐AZTTPs 10bz mainly followed pathways d and e (Scheme 5). This contrasted the observation made with γ‐(AB‐C4;alkyl‐C18)‐AZTTP 10by (Figure 3A) and γ‐(AB‐C4;alkyl‐C18)‐FTCTP 10cy (Figure 3C).

Next, Tri*PPP*ro‐compounds 10 and 20 were exposed to PLE in PBS (pH 7.3) and the half‐lives of Tri*PPP*ro‐compounds 10 and 20 are listed in Table 1. As expected γ‐(alkyl‐C4;alkyl‐C18)‐NTPs 10bv–hv, γ‐(alkyl‐C4)‐γ‐C‐(alkyl‐C18)‐NTPs 10bx–hx, and γ‐(alkyl‐C18)‐NTPs 20 were found to be highly stable against PLE (*t*_1/2_ >150 h). In contrast, the cleavage of the AB (C4) masking group in Tri*PPP*ro‐compounds 10by–hy and 10bz–hz was triggered by ester hydrolysis and yielded the corresponding γ‐alkylated nucleoside triphosphate derivatives 20 (Figure 3B,D) and 21 (Figures S17 and S18, Supporting Information), respectively. Here, the half‐lives for Tri*PPP*ro‐compounds 10by–hy and 10bz–hz (*t*_1/2_ = 0.19–1.3 h) were found to be dramatically lower than the corresponding half‐lives (*t*_1/2_ = 46–1500 h) in chemical studies. Moreover, a very small amount of NDPs was observed, probably due to a reaction involving a nucleophilic reaction at the γ‐phosphate or γ‐phosphonate moiety (pathways d and f, Scheme 5).

**Figure 4:**
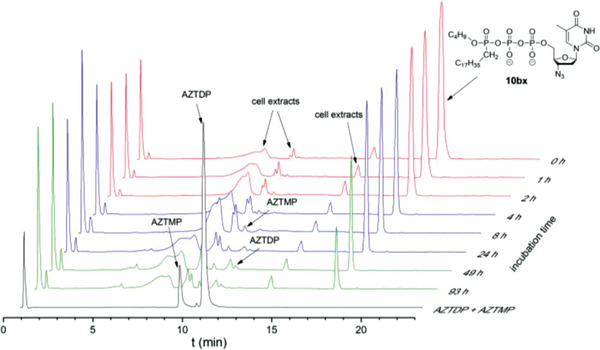
Stack plot of the HPLC chromatograms of 10bx after incubation in CEM/0 cell extracts.

**Figure 5:**
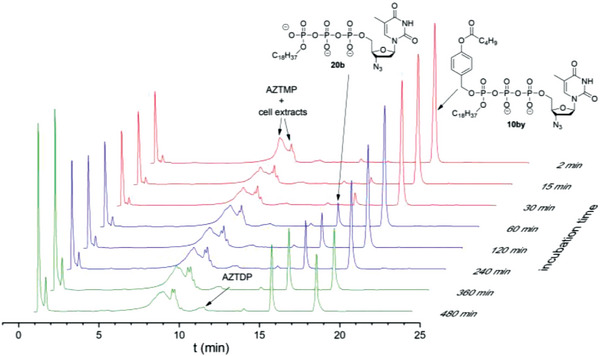
Stack plot of the HPLC chromatograms of 10by after incubation in CEM/0 cell extracts.

The hydrolysis of Tri*PPP*ro‐compounds 10 and 20 was carried out with CEM/0 cell extracts as well. As can be seen in Table 1, the half‐lives determined for Tri*PPP*ro‐compounds 10bv–gv and 10bx–hx (*t*_1/2_ >10 h, except 10hv) bearing two alkyl groups as well as the γ‐mono‐masked triphosphates 20 (*t*_1/2_ >10 h) were very high, thus compounds were highly resistant toward hydrolysis in CEM/0 cell extracts. These Tri*PPP*ro‐compounds 10 and 20 were slowly hydrolyzed to form a small amount of the corresponding NDPs illustrated in **Figure** **4** and Figures S19 and S20 (Supporting Information). As compared to the first generation Tri*PPP*ro‐NTPs 2,3 (d4T as an example; Scheme 2),^[^ ^50^, ^53^, ^54^ ^]^ it was impossible to detect the formation of NTPs because of the missing esterase cleavage site. In contrast, an enzymatic process took place at the acyloxybenzyl moiety of Tri*PPP*ro‐compounds 10by–hy and 10bz–hz that led to γ‐modified triphosphate derivatives 20 and 21, respectively. Moreover, it was shown that an increase in the amount of γ‐alkylated nucleoside triphosphate derivatives 20 and a small amount of NDPs were detected after long incubation times (t ≥8 h) in CEM/0 cell extracts (**Figure** **5**; Figures S21–S24, Supporting Information). Furthermore, the biological stabilities of C18‐phosphonate‐Tri*PPP*ro‐compounds 10bz (*t*_1/2_ = 8 h), 10dz (*t*_1/2_ >10 h), and 10hz (*t*_1/2_ = 7.7 h) were found to be higher as compared to the corresponding C18‐phosphate‐Tri*PPP*ro‐compounds 10by (*t*_1/2_ = 3.3 h), 10dy (*t*_1/2_ = 2.9 h), and 10hy (*t*_1/2_ = 1.3 h), respectively, which is in good agreement with the data reported previously for Tri*PPP*ro‐d4TTPs 4,5.^[^ ^56^, ^58^ ^]^

Next, in order to investigate the hydrolysis properties of γ‐alkylated triphosphate derivatives 10,20 and to explain their antiviral activity, a selection of eight Tri*PPP*ro‐compounds 10 and 20 comprising AZT or ABC (e.g. most active compound 10hv in Table 3) were further studied in human plasma at 37 °C. As expected, γ‐dialkylated triphosphate derivatives 10bv,10hv, and 10hx (*t*_1/2_ > 8 h, **Table** **2**) were found to be more stable than the AB group comprising Tri*PPP*ro‐compounds 10bz (*t*_1/2_ = 4.4–7.6 h) and 10hz (*t*_1/2_ = 4.1–5.6 h), respectively. In the case of dialkylated γ‐(alkyl‐C4;alkyl‐C18)‐AZTTP 10bv in heparin‐stabilized human plasma (Figure S25, Supporting Information) or citrate‐stabilized plasma (Figure S26, Supporting Information) the formation of AZT but almost no AZTMP, AZTDP and γ‐(alkyl‐C18)‐AZTTP 20b were detected. The formation of γ‐monoalkylated triphosphate derivatives 20b (and the phosphonate analogs) was observed from γ‐(AB‐C4;alkyl‐C18)‐AZTTP 10by,  γ‐(AB‐C4)‐γ‐C‐(alkyl‐C18)‐AZTTP 10bz, and γ‐(AB‐C4)‐γ‐C‐(alkyl‐C18)‐ABCTP 10hz (shown in Figures S27–S32, Supporting Information), respectively, which was then led to the slow formation of the corresponding NDPs and NMPs (Figures S33 and S34, Supporting Information). Furthermore, AZTMP (*t*_1/2_ = 2.0–4.5 h) and AZTDP (*t*_1/2_ = 2.8–6.4 h) proved to be surprisingly unstable in human plasma and AZTDP is dephosphorylated to give AZTMP and ultimately AZT (Figures S25–S38, Supporting Information).

**Table 2:** Stabilities of Tri*PPP*ro‐NTPs 10 and γ‐alkyl‐NTPs 20 in human plasma.

Comp.	Plasma (Heparin)	Plasma (Citrate)	Comp.	Plasma (Heparin)	Plasma (Citrate)
	*t*_1/2_ [h]	*t*_1/2_ [h]		*t*_1/2_ [h]	*t*_1/2_ [h]
10bv	>20	>20	10hv	>8	>20
10by	5.2	3.7	10hx	>20	>20
10bz	7.6	4.4	10hz	4.1	5.6
20b	3.7	>20	20h	4.8	>20
AZTDP	2.8	6.4	AZTMP	2.0	4.5

### 2.3 Anti‐HIV Activity

All Tri*PPP*ro‐compounds 10 and 20, as well as their parent nucleosides and the corresponding NTPs, were evaluated for their antiviral activity (expressed as EC_50_ values) against HIV‐1 (wild‐type CEM/0 cells) and HIV‐2 (wild‐type CEM/0 cells and thymidine kinase‐deficient (CEM/TK^−^) cells) with concomitant determination of cytotoxicity in the same cell line (**Table** **3**). Most parent nucleosides such as AZT 1b (EC_50_ >150 µm) showed very poor if any anti‐HIV activity in the mutant thymidine kinase‐deficient (CEM/TK^−^) cells. Similarly, as expected the corresponding triphosphates (e.g. AZTTP; EC_50_ = 57.9 µm) exhibited no significant antiviral activity in CEM/TK^−^ cell cultures.

**Table 3:** Antiviral activity and cytotoxicity of Tri*PPP*ro‐compounds 10, 20, and NTPs.

Comp.	Antiviral activity			Toxicity
	CEM/0 HIV‐1 [HE]	CEM/0 HIV‐2 [ROD]	CEM/TK^−^ HIV‐2 [ROD)]	CEM/0
	EC_50_ ^a)^ [µm]	EC_50_ ^a)^ [µm]	EC_50_ ^a)^ [µm]	CC_50_ ^b)^ [µm]
10bv	0.041 ± 0.019	0.026 ± 0.007	0.29 ± 0.15	64.9
10bw	0.16 ± 0.19	0.054 ± 0.028	6.1 ± 3.0	45.6 ± 0
10bx	0.12 ± 0.088	0.031 ± 0.005	1.19 ± 0.79	>100
10by	0.19 ± 0.12	0.0052 ± 0.0041	0.50 ± 0.35	60.1
10bz	0.15 ± 0.15	0.0075 ± 0.0081	0.31 ± 0.026	58.9
20b	0.028 ± 0.031	0.030 ± 0.001	4.1 ± 2.6	>100
AZTTP	0.0069 ± 0.0087	0.0055 ± 0.0040	57.9 ± 10.9	>100
1b (AZT)	0.014 ± 0.0085	0.0034 ± 0.0021	>100	>100
10cv	0.039 ± 0.035	0.074 ± 0.027	0.0032	51.2 ± 7.7
10cw	0.21 ± 0.06	0.18 ± 0.10	0.12 ± 0.10	64.0 ± 23.6
10cx	0.049 ± 0.045	0.094 ± 0.023	0.011	46.0 ± 0
10cy	0.14 ± 0.065	0.18 ± 0.15	0.062 ± 0.059	51.87
10cz	0.16 ± 0.06	0.14 ± 0.04	0.020 ± 0.021	66.0 ± 35.6
20c	0.35 ± 0.25	0.22 ± 0.02	0.051 ± 0.069	63.4 ± 13.7
FTCTP	0.028 ± 0.015	0.043 ± 0.040	0.025 ± 0.027	>100
1c (FTC)	0.0086 ± 0.0057	0.020 ± 0.001	0.010 ± 0.010	>100
10dv	2.28 ± 0.68	4.81 ± 4.61	>20	50.1 ± 10.2
10dw	1.20 ± 0.05	2.20 ± 1.24	>20	56.1 ± 15.8
10dx	2.59 ± 0.71	2.23 ± 1.20	3.21 ± 1.12	54.4 ± 15.9
10dy	7.32 ± 2.28	1.95 ± 0.86	2.53 ± 1.38	52.1
10dz	2.02 ± 0.80	0.51 ± 0.14	4.66 ± 3.42	54.9
20d	3.14 ± 2.93	2.24 ± 0.65	62.1 ± 0	>100
FddClUTP	0.84 ± 0.86	0.65 ± 0.13	>100	>100
1d (FddClU)	0.82 ± 0.33	1.95 ± 2.63	>100	>100
10ev	0.63 ± 0.14	0.40 ± 0.11	0.34 ± 0.23	54.9 ± 16.9
10ew	1.50 ± 1.39	0.86 ± 0.19	0.28 ± 0.12	52.6 ± 10.9
10ex	0.39 ± 0.13	0.52 ± 0.67	1.01 ± 1.14	43.6 ± 0
10ey	0.30 ± 0.30	0.78 ± 0.30	1.58 ± 1.97	54.1 ± 15.8
10ez	0.46 ± 0.36	0.46 ± 0.40	1.83 ± 1.65	45.3 ± 16.7
20e	0.092 ± 0.082	0.39 ± 0.30	3.56 ± 1.88	63.7 ± 13.6
FddUTP	0.15 ± 0.13	0.26 ± 0.32	35.42 ± 43.92	>100
1e (FddU)	0.21 ± 0.02	0.15 ± 0.017	>100	>100
10fv	0.038 ± 0.0037	0.037 ± 0.030	0.052 ± 0.046	48.7 ± 9.1
10fw	0.37 ± 0.47	0.14 ± 0.062	0.11 ± 0.14	63.5 ± 27.2
10fx	0.041 ± 0.044	0.029 ± 0.0071	0.15 ± 0.06	67.3 ± 33.8
10fy	0.026 ± 0.028	0.014 ± 0.016	0.36 ± 0.24	43.3 ± 5.1
10fz	0.064 ± 0.080	0.039 ± 0.033	0.16 ± 0.04	42.0 ± 10.6
20f	0.019 ± 0.019	0.010 ± 0.001	1.27 ± 0.54	>100
FLTTP	0.0076 ± 0.0091	0.018 ± 0.009	4.92 ± 2.43	>100
1f (FLT)	0.0025 ± 0.0029	0.0043 ± 0.0017	>100	>100
10gv	>10	5.37 ± 0.69	36.45 ± 34.73	48.0 ± 4.2
10gw	>10	4.21 ± 0.37	11.35 ± 4.24	60.3 ± 20.7
10gx	>10	>10	23.95 ± 2.72	51.5 ± 17.0
10gy	8.57 ± 5.02	2.87 ± 1.72	11.09 ± 1.34	52.1
10gz	7.58 ± 2.20	8.01 ± 2.81	11.22 ± 4.99	43.9 ± 8.3
20g	>10	>10	15.72 ± 0.98	>100
BVDUTP	4.73 ± 3.05	5.64 ± 4.45	>100	>100
1g (BVDU)	>250	>250	>250	207 ± 60
10hv	0.024 ± 0.0014	0.046 ± 0.016	0.0048 ± 0.0021	39.0 ± 4.7
10hw	0.10 ± 0.03	0.18 ± 0.11	0.026 ± 0.035	>100
10hx	0.15 ± 0.14	0.044 ± 0.033	0.0058	38.6 ± 0
10hy	0.96 ± 0.49	0.29 ± 0.16	0.21 ± 0.11	>100
10hz	0.20 ± 0	0.17 ± 0.064	0.019 ± 0.001	38.2 ± 1.3
20h	0.26 ± 0.13	0.42 ± 0.11	0.16 ± 0.16	>100
ABCTP	1.85 ± 2.14	1.02 ± 0.13	0.85 ± 0.40	>100
1h (ABC)	9.39 ± 2.45	3.51 ± 3.25	1.98 ± 0.86	>100

^a)^ Antiviral activity determined in CD4^+^ T‐lymphocytes: 50% effective concentration; values are the mean ±SD of n = 2–3 independent experiments.

^b)^ Cytotoxicity: 50% cytostatic concentration or compound concentration required to inhibit CD4^+^ T‐cell (CEM) proliferation by 50%; values are the mean ±SD of n = 2–3 independent experiments.

As summarized in Table 3, some of the Tri*PPP*ro‐compounds 10 were highly antivirally active against HIV‐1 and HIV‐2 in wild‐type CEM/0 cells, while others were as active as their corresponding nucleosides 1. The high antiviral activity determined for some Tri*PPP*ro‐compounds 10 in the wild‐type CEM/0 cells was often completely retained in HIV‐2 infected CEM/TK^−^ cells. For Tri*PPP*ro‐ABCTP 10hv (EC_50_ = 0.0048 µm/HIV‐2) the antiviral activity in CEM/TK^−^ cells was improved by tenfold as compared to the antiviral activity of Tri*PPP*ro‐ABCTP 10hv (EC_50_ = 0.024 µm/HIV‐1; EC_50_ = 0.046 µm/HIV‐2) in wild‐type CEM/0 cells. For Tri*PPP*ro‐ABCTP 10hv the activity was almost 400‐fold and 80‐fold higher as the parent abacavir (ABC) 1h (EC_50_ = 9.39 µm/HIV‐1; EC_50_ = 3.51 µm/HIV‐2) in wild‐type CEM/0 cells. Tri*PPP*ro‐ABCTP 10hv (EC_50_ = 0.0048 µm/HIV‐2) is one of the most active compounds disclosed here, as also the antiviral activity was improved in CEM/TK^−^ cells by 410‐fold compared to ABC 1h (EC_50_ = 1.98 µm/HIV‐2).

As compared to previously studied *cyclo*Sal‐ABCMP (EC_50_ = 0.70 µm/HIV‐1; EC_50_ = 0.75 µm/HIV‐2)^[^ ^65^, ^66^ ^]^ the antiviral activity of Tri*PPP*ro‐ABCTP 10hv in wild‐type CEM/0 cells was improved by 30‐fold and 16‐fold, respectively, demonstrating the potential of Tri*PPP*ro‐ABCTP 10hv comprising two non‐cleavable alkyl groups. The inhibition of the HIV replication by Tri*PPP*ro‐compounds 10hv–hz was also tenfold to 440‐fold better compared to our previously reported bis(acyloxybenzyl)‐Tri*PPP*ro‐ABCTP‐prodrug (EC_50_ = 5.3 µm/ CEM/0 cells; EC_50_ = 2.1 µm/CEM/TK^−^ cells) in HIV‐2‐infected cells.^[^ ^51^ ^]^ These antiviral activities are particularly interesting because they point to a different mode of action of the phosphorylated abacavir (ABC) metabolites here.

It has been reported that abacavir acts as a prodrug for the toxic carbovir (CBV).^[^ ^67^, ^68^ ^]^ However, after intracellular monophosphorylation ABCMP is converted by adenosine monophosphate deaminase (AMPDA) into carbovir‐monophosphate (CBVMP), which is then processed into its triphosphate.^[^ ^67^, ^68^ ^]^ Thus, the active form of abacavir is the HIV‐RT inhibitor carbovir‐triphosphate (CBVTP). However, for the Tri*PPP*ro‐compounds disclosed here, it was shown that the ABCDP is formed that cannot be converted into carbovir‐diphosphate (CBVDP). This suggests that the active form in our case is indeed ABCTP or ABCDP itself.

Furthermore, besides the full retention of the antiviral activity in HIV‐2‐infected wild‐type CEM/0 cells, Tri*PPP*ro‐FddUTP 10ev (EC_50_ = 0.34 µm) and Tri*PPP*ro‐FLTTP 10fv (EC_50_ = 0.052 µm) also showed much better activities against HIV‐2 than their corresponding completely inactive parent nucleosides FddU 1e and FLT 1f (both EC_50_ >100 µm) in CEM/TK^−^ cells. The improvements in activity here were >290‐fold and >1900‐fold, respectively. As compared to the previously reported bis(AB)‐Tri*PPP*ro‐FLTTP (EC_50_ = 0.54 µm/HIV‐2)^[^ ^69^ ^]^ or AB,alkyl‐Tri*PPP*ro‐FLTTP (EC_50_ = 1.16 µm/HIV‐2))^[^ ^69^ ^]^ the activity of Tri*PPP*ro‐FLTTP 10fv in CEM/TK^−^ cells was increased by a factor of tenfold and 22‐fold. Therefore, it was concluded that Tri*PPP*ro‐compounds 10 released nucleo*t*ide analog metabolites intracellularly, thus bypassing the kinases (Scheme 1).

Most Tri*PPP*ro‐compounds 20 bearing a long lipophilic alkyl chain (C18) attached to the γ‐phosphate moiety were endowed with moderate antiviral activity in the cell assay using CEM/TK^−^ cells. It seems that Tri*PPP*ro‐compounds 20 were at least in part able to cross the cell membrane and deliver phosphorylated metabolites presumably NDPs, or are used as substrates in their mono‐alkylated form. Nevertheless, some Tri*PPP*ro‐compounds 10 comprising non‐bioreversible and hydrolytically stable alkyl groups, such as 10hv (EC_50_ = 0.0048 µm/HIV‐2), had much higher (33‐fold) activity as compared to the corresponding Tri*PPP*ro‐compounds 20h (EC_50_ = 0.16 µm/HIV‐2), proving the advantage of prodrug strategy. Notably, Tri*PPP*ro‐compounds 10 and 20 did not show a significant increase in cytotoxicity as compared to their parent nucleosides.

### 2.4 Lipophilicity for Tri*PPP*ro‐Compounds 10,20

As can be seen in Table 1, the lipophilicity of Tri*PPP*ro‐compounds 10bv–hv (alkyl‐C4;alkyl‐C18) and 10bw–hw (alkyl‐C8;alkyl‐C18) increased with increasing alkyl chain lengths (R^1^). However, lipophilicities of Tri*PPP*ro‐compounds 10bv–hv (*t*_R_ = 16.0–16.7 min) and 10by–hy (*t*_R_ = 17.0–17.7 min) were in the same range as Tri*PPP*ro‐compounds 10bx–hx (*t*_R_ = 15.8–16.4 min) and 10bz–hz (*t*_R_ = 16.7–17.4 min), respectively. In addition, all Tri*PPP*ro‐compounds 10by–hy and 10bz–hz comprising an AB‐group (C4) in addition to a non‐cleavable moiety (alkyl‐C18) at the γ‐phosphate or γ‐phosphonate group, respectively, showed higher lipophilicity than Tri*PPP*ro‐compounds 10bv–hv and 10bx–hx comprising two non‐bioreversible alkyl moieties. Therefore, it was concluded that the lipophilicity of Tri*PPP*ro‐compounds 10 was significantly influenced by the masking moiety and slightly governed by the attached nucleoside analog. As compared to Tri*PPP*ro‐compounds 10, γ‐(alkyl‐C18)‐NTPs 20 (*t*_R_ = 14.3–14.7 min) bearing only one non‐bioreversible moiety showed a marked loss of lipophilicity. As a consequence, γ‐(alkyl‐C18)‐NTPs 20 showed mostly a loss of antiviral activity in CEM/TK^−^ cell cultures, probably due to the insufficient lipophilicity of these Tri*PPP*ro‐compounds 20 to cross the biological barriers.

### 2.5 Primer Extension Assays

We examined Tri*PPP*ro‐prodrugs 10 and 20 in primer extension assays and investigated their suitability to act as substrates for the HIV‐RT as compared to two different human DNA polymerases α,γ. In these primer extension assays, the four canonical NTPs were added to the polymerases (positive control (+ lane)) or were added in the absence of the polymerase (negative control (−lane)). TTP, dCTP, and dATP were used as the reference compounds because they were substrates for HIV‐RT and DNA polymerases α,γ.

**Figure 6:**
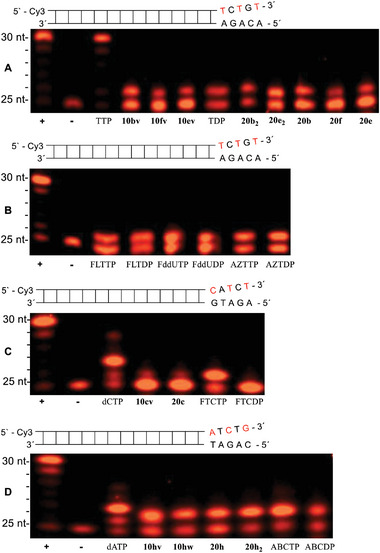
Primer extension assay using HIV‐RT (30 min, 6U). A) Lane 1: (+) dATP, dCTP, dGTP and TTP with HIV‐RT. Lane 2: (−) dATP, dCTP, dGTP and TTP without HIV‐RT. Lane 3: TTP. Lane 4: γ‐(C4;C18)‐AZTTP 10bv. Lane 5: γ‐(C4;C18)‐FLTTP 10fv. Lane 6: γ‐(C4;C18)‐FddUTP 10ev. Lane 7: TDP. Lane 8: γ‐C4‐AZTTP 20b_2_. Lane 9: γ‐C4‐FddUTP 20e_2_. Lane 10: γ‐C18‐AZTTP 20b. Lane 11: γ‐C18‐FLTTP 20f. Lane 12: γ‐C18‐FddUTP 20e. B) Lane 1: (+) dATP, dCTP, dGTP and TTP with HIV‐RT. Lane 2: (−) dATP, dCTP, dGTP and TTP without HIV‐RT. Lane 3: FLTP. Lane 4: FLTDP. Lane 5: FddUTP. Lane 6: FddUDP. Lane 7: AZTTP. Lane 8: AZTDP. C) Lane 1: (+) dATP, dCTP, dGTP and TTP with HIV‐RT. Lane 2: (−) dATP, dCTP, dGTP and TTP without HIV‐RT. Lane 3: dCTP. Lane 4: γ‐(C4;C18)‐FTCTP 10cv. Lane 5: γ‐C18‐FTCTP 20c. Lane 6: FTCTP. Lane 7: FTCDP. D) Lane 1: (+) dATP, dCTP, dGTP and TTP with HIV‐RT. Lane 2: (−) dATP, dCTP, dGTP and TTP without HIV‐RT. Lane 3: dATP. Lane 4: γ‐(C4;C18)‐ABCTP 10hv. Lane 5: γ‐(C4)‐γ‐(C18)‐ABCTP 10hw. Lane 6: γ‐C18‐ABCTP 20h. Lane 7: γ‐C4‐ABCTP 20h_2_. Lane 8: ABCTP. Lane 9: ABCDP.

The result of a primer extension assay in which γ‐(alkyl‐C4;alkyl‐C18)‐NTPs 10, γ‐(alkyl‐C18)‐NTPS 20 and HIV‐RT were used is shown in **Figure** **6**. Except for FTCDP, HIV‐RT can also utilize NDP (Figure 6A,B,D) in the polymerization reaction.^[^ ^70^ ^]^ What makes the difference to FTCDP remains unclear. Therefore, n+1 band (26 nt) was observed because NMPs were incorporated and acted as an obligate chain terminator. Thus, γ‐(alkyl‐C4;alkyl‐C18)‐NTPs 10 and γ‐(C18 or C4)‐NTPs 20 were substrates HIV‐RT.

**Figure 7:**
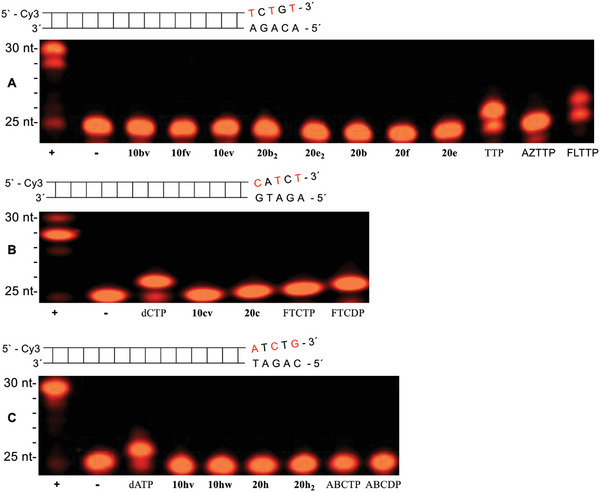
Primer extension assay using human polymerase α (60 min, 2U). A) Lane 1: (+) dATP, dCTP, dGTP and TTP with human polymerase α. Lane 2: (−) dATP, dCTP, dGTP and TTP without polymerase α. Lane 3: γ‐(C4;C18)‐AZTTP 10bv. Lane 4: γ‐(C4;C18)‐FLTTP 10fv. Lane 5: γ‐(C4;C18)‐FddUTP 10ev. Lane 6: γ‐C4‐AZTTP 20b_2_. Lane 7: γ‐C4‐FddUTP 20e_2_. Lane 8: γ‐C18‐AZTTP 20b. Lane 9: γ‐C18‐FLTTP 20f. Lane 10: γ‐C18‐FddUTP 20e. Lane 11: TTP. Lane 12: AZTTP. Lane 13: FLTTP. B) Lane 1: (+) dATP, dCTP, dGTP and TTP with polymerase α. Lane 2: (−) dATP, dCTP, dGTP and TTP without polymerase α. Lane 3: dCTP. Lane 4: γ‐(C4;C18)‐FTCTP 10cv. Lane 5: γ‐C18‐FTCTP 20c. Lane 6: FTCTP. Lane 7: FTCDP. C) Lane 1: (+) dATP, dCTP, dGTP and TTP with polymerase α. Lane 2: (−) dATP, dCTP, dGTP and TTP without polymerase α. Lane 3: dATP. Lane 4: γ‐(C4;C18)‐ABCTP 10hv. Lane 5: γ‐(C4)‐γ‐(C18)‐ABCTP 10hw. Lane 6: γ‐C18‐ABCTP 20h. Lane 7: γ‐C4‐ABCTP 20h_2_. Lane 8: ABCTP. Lane 9: ABCDP.

**Figure 8:**
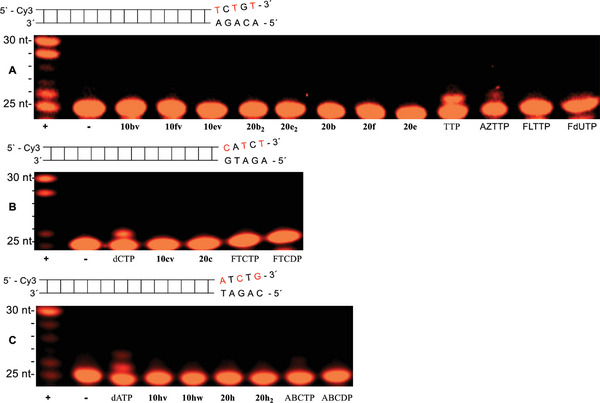
Primer extension assay using human polymerase γ (120 min, 2U). A) Lane 1: (+) dATP, dCTP, dGTP and TTP with polymerase γ. Lane 2: (−) dATP, dCTP, dGTP, and TTP without polymerase γ. Lane 3: γ‐(C4;C18)‐AZTTP 10bv. Lane 4: γ‐(C4;C18)‐FLTTP 10fv. Lane 5: γ‐(C4;C18)‐FddUTP 10ev. Lane 6: γ‐C4‐AZTTP 20b_2_. Lane 7: γ‐C4‐FddUTP 20e_2_. Lane 8: γ‐C18‐AZTTP 20b. Lane 9: γ‐C18‐FLTTP 20f. Lane 10: γ‐C18‐FddUTP 20e. Lane 11: TTP. Lane 12: AZTTP. Lane 13: FLTTP. Lane 14: FddUTP. B) Lane 1: (+) dATP, dCTP, dGTP and TTP with polymerase γ. Lane 2: (−) dATP, dCTP, dGTP and TTP without polymerase γ. Lane 3: dCTP. Lane 4: γ‐(C4;C18)‐FTCTP 10cv. Lane 5: γ‐C18‐FTCTP 20c. Lane 6: FTCTP. Lane 7: FTCDP. C) Lane 1: (+) dATP, dCTP, dGTP and TTP with polymerase γ. Lane 2: (−) dATP, dCTP, dGTP and TTP without polymerase γ. Lane 3: dATP. Lane 4: γ‐(C4;C18)‐ABCTP 10hv. Lane 5: γ‐(C4)‐C‐γ‐(C18)‐ABCTP 10hw. Lane 6: γ‐C18‐ABCTP 20h. Lane 7: γ‐C4‐ABCTP 20h_2_. Lane 8: ABCTP. Lane 9: ABCDP.

Human DNA polymerases α (**Figure** **7**) and γ (**Figure** **8**) were also tested to ensure n+1 band incorporation. As compared to HIV‐RT, no incorporation was detected in primer extension assays using human DNA polymerases α or γ for Tri*PPP*ro‐compounds 10 and 20. Thus, these experiments proved that the double alkylated Tri*PPP*ro‐prodrugs 10 and single alkylated compounds 20 were not substrates for human DNA polymerases α or γ.

## 3 Conclusion

In summary, the synthesis of a series of Tri*PPP*ro‐compounds 10 and 20 bearing different nucleoside analogs is described here, demonstrating the applicability of the Tri*PPP*ro‐strategy. Tri*PPP*ro‐NTPs 10 as well as γ‐mono‐masked triphosphates 20 were prepared by using the new *H*‐phosphonate route and *H*‐phosphinate route with modest to very good yields.

As compared to the studies of the ester or carbonate‐bearing Tri*PPP*ro‐compounds 2,3 comprising two biodegradable masking groups attached to the γ‐phosphate group,^[^ ^50^, ^54^ ^]^ we have proven that Tri*PPP*ro‐compounds 10bv–hv and 10bw–hw comprising two non‐cleavable alkyl moieties were very stable toward hydrolysis in chemical and biological media. Compounds bearing one bioreversible AB group showed an almost selective cleavage of this AB‐group from Tri*PPP*ro‐compounds 10by–hy (AB‐C4;C18) that was initiated by ester hydrolysis to yield the corresponding γ‐C18‐NTPs 20b–h. The latter compounds were stable in PBS (*t*_1/2_ >2000 h), PLE (*t*_1/2_ >150 h), CEM/0 cell extracts (*t*_1/2_ >10 h) and human citrate plasma (*t*_1/2_ >20 h). In all cases, before complete consumption of Tri*PPP*ro‐compounds 10 and 20, no formation of NTPs was observed in these studies, which is in good agreement with the data obtained from Tri*PPP*ro‐d4TTPs 4–9.^[^ ^50^, ^53^, ^54^, ^56^, ^57^, ^58^ ^]^

Remarkably, all Tri*PPP*ro‐compounds 10 and 20 were highly antivirally active in CEM/TK^−^ cell cultures in contrast to their parent nucleoside analogs 1, proving the excellent potential of the Tri*PPP*ro‐concept. In antiviral assays, very good anti‐HIV activity of γ‐(C4;C18)‐FLTTP 10fv (EC_50_ = 0.052 µm/HIV‐2) and γ‐(C4; C18)‐ABCTP 10hv (EC_50_ = 0.0048 µm/HIV‐2) was detected in CEM/TK^−^ cells with >1900‐fold and 410‐fold improved activity as compared to the parent FLT 1f (EC_50_ >100 µm/HIV‐2) or ABC 1h (EC_50_ = 1.98 µm/HIV‐2). Tri*PPP*ro‐compounds 10hv–hz of ABC were active against HIV‐2 in CEM/0 cells, more importantly, they exhibited similar or even better activities (up to tenfold) against HIV‐2 in cultures of infected CEM/TK^−^ cells. As a consequence, obviously Tri*PPP*ro‐compounds 10 and 20 possess enough lipophilicity to cross the biological barriers and delivered the nucleotide analogs, most likely their corresponding NDPs. These compounds were also proved to be substrates for HIV‐RT, which can explain the marked anti‐HIV activity in HIV‐infected cells.

The approach also points to interesting details on the activation of uracil‐bearing nucleoside analogs. This was shown for FddClU 1d, FddU 1e which were active in the wild‐type cells but inactive in the TK‐deficient cells. The conversion of these nucleosides into their triphosphate prodrug form restored the antiviral activity pointing to at least a contribution of thymidine‐kinase to the metabolic phosphorylation of uracil comprising nucleosides. Interestingly, the approach also converted BVDU, an anti‐VZV, and HSV‐1 (herpes viruses) active nucleoside analog, into a powerful anti‐HIV active compound and thus broadens the antiviral spectrum of the parent 1g.

In conclusion, it was convincingly shown that this Tri*PPP*ro‐approach provides high potential for further antiviral chemotherapies. Highly active Tri*PPP*ro‐prodrugs may be developed for the treatment of infections by not only HIV but also for SARS‐CoV‐2 and other RNA viruses in the future.

## Conflict of Interest

The authors declare no conflict of interest.

## Supporting information

Supporting Information

## Data Availability

The data that support the findings of this study are available in the supplementary material of this article.
