# Supplementary material for: Lipophilic Nucleoside Triphosphate Prodrugs of Anti‐HIV Active Nucleoside Analogs as Potential Antiviral Compounds
Source: Adv Sci (Weinh). 2023 Oct 26;10(36):2306021. doi: 10.1002/advs.202306021 (PMC10754118; doi:10.1002/advs.202306021)
Supplement: Supplementary file 1 — Supporting Information [file ADVS-10-2306021-s001.pdf]

ADVANCED SCIENCE
Open Access

Supporting Information

for *Adv. Sci.*, DOI 10.1002/advs.202306021

Lipophilic Nucleoside Triphosphate Prodrugs of Anti-HIV Active Nucleoside Analogs as Potential Antiviral Compounds

*Xiao Jia, Dominique Schols and Chris Meier**

Supporting Information

Lipophilic Nucleoside Triphosphate Prodrugs of Anti-HIV Active Nucleoside Analogues as Potential Antiviral Compounds

Xiao Jia,[1] *Dominique Schols,*[2] *Chris Meier*[1*]

[1]Organic Chemistry, Department of Chemistry, Faculty of Mathematics, Informatics and Natural Sciences, Universität Hamburg, Martin-Luther-King-Platz 6, D-20146 Hamburg, Germany.

[2]Laboratory of Virology and Chemotherapy, Department of Microbiology and Immunology and Transplantation, Rega Institute for Medical Research, KU Leuven, Herestraat 49, B-3000 Leuven, Belgium.

Table of Contents

Figure S1-S38. HPLC profiles of compounds **10** and **20** after incubation in PBS (pH 7.3), PLE, CEM cell extracts and human plasma. 2

Experimental Section. 14

Part 1. General Procedure 1: Preparation of Tri*PPP*ro-compounds **10** (method 1). 15

Part 2. General Procedure 2: Preparation of Tri*PPP*ro-compounds **10** (method 2). 15

Part 3. Synthesis and Characterization. 16

Part 4. Chemical Hydrolysis of Tri*PPP*ro-compounds **10** and **20**. 41

Part 5. Hydrolysis of Tri*PPP*ro-compounds **10** and **20** with PLE 41

Part 6. Enzyme-Catalyzed Hydrolysis of Tri*PPP*ro-compounds **10** and **20** in CEM Cell extracts. 41

Part 7. Hydrolysis of Tri*PPP*ro-compounds **10** and **20** in human plasma……………………..…………...41

Part 8. Preparation of cell extracts and human plasma. 42

Part 9. Anti-HIV activity assay. 42

Part 10. Primer-extension assays…………………………………………………………………...…….42

Part 11. Spectral Data for New Compounds. 44

Reference. 124

Figure S1-S38. HPLC profiles of compounds **10** and **20** after incubation in PBS (pH 7.3), PLE, CEM cell extracts and human plasma.

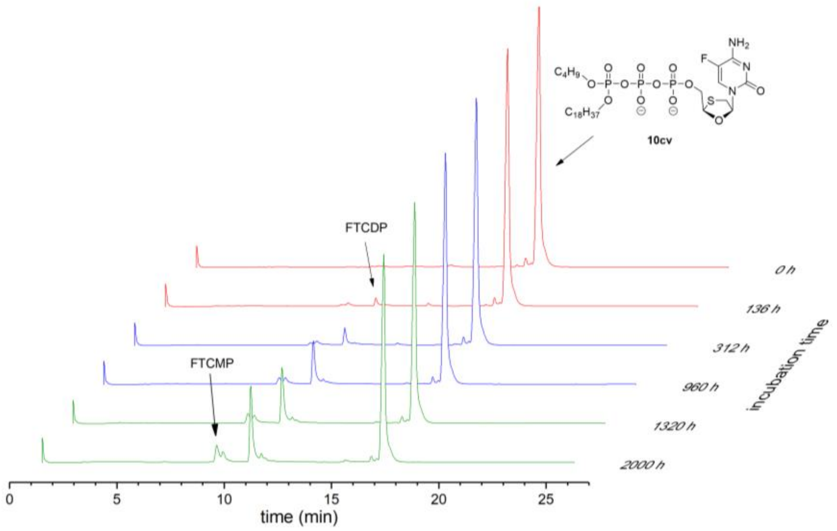

Figure S1. HPLC profiles of **10cv** after incubation in PBS (pH 7.3).

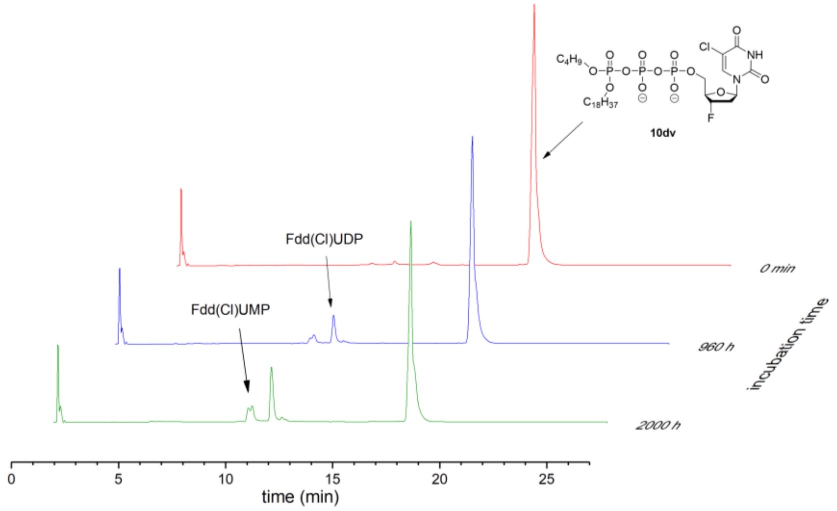

Figure S2. HPLC profiles of **10dv** after incubation in PBS (pH 7.3).

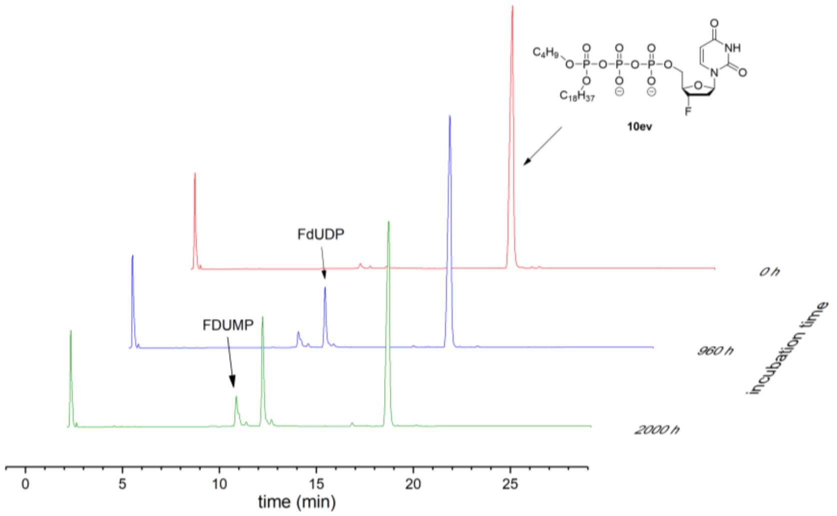

Figure S3. HPLC profiles of **10ev** after incubation in PBS (pH 7.3).

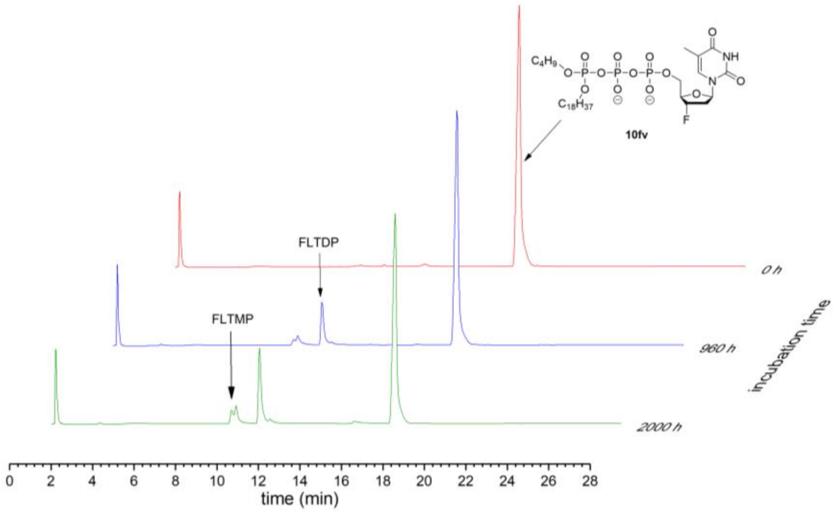

Figure S4. HPLC profiles of **10fv** after incubation in PBS (pH 7.3).

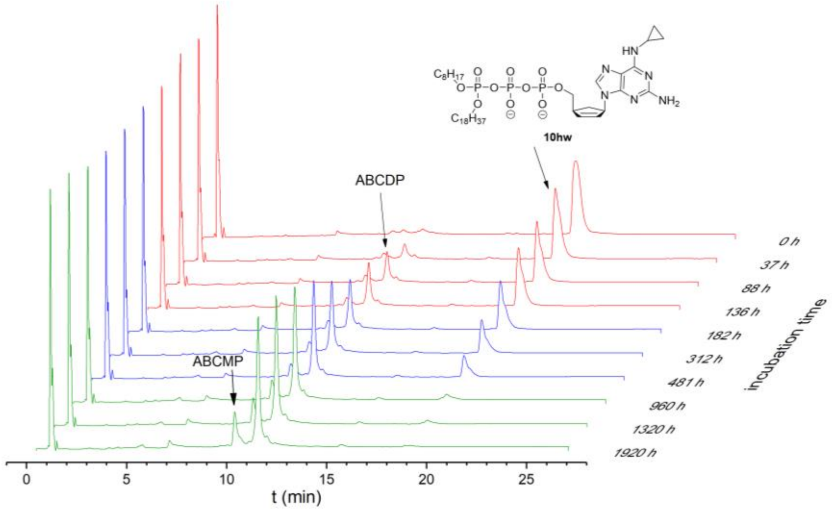

Figure S5. HPLC profiles of **10hw** after incubation in PBS (pH 7.3).

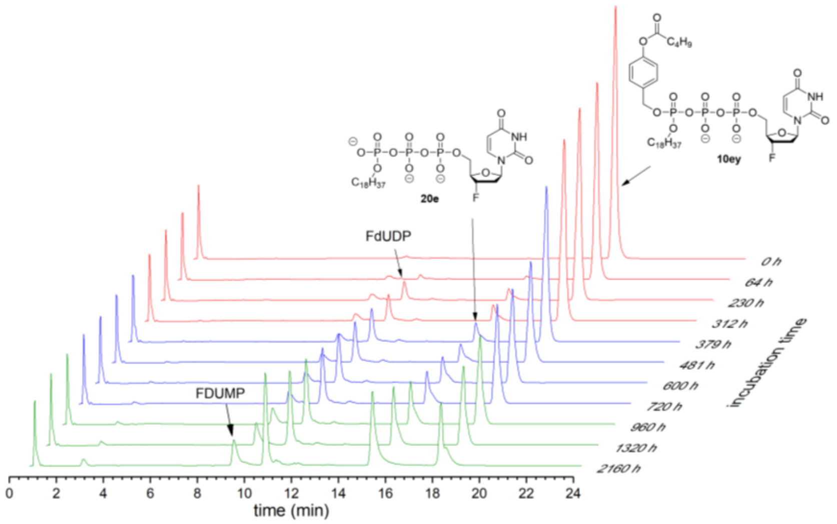

Figure S6. HPLC profiles of **10ey** after incubation in PBS (pH 7.3).

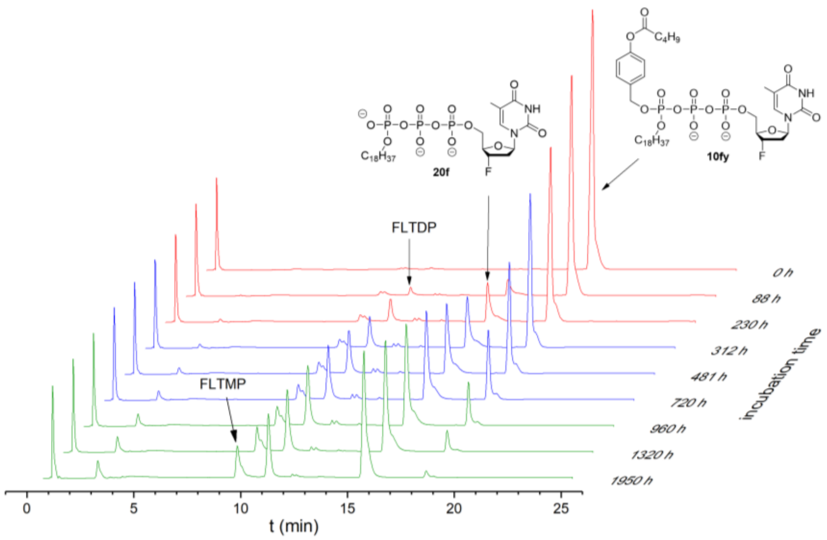

Figure S7. HPLC profiles of **10fy** after incubation in PBS (pH 7.3).

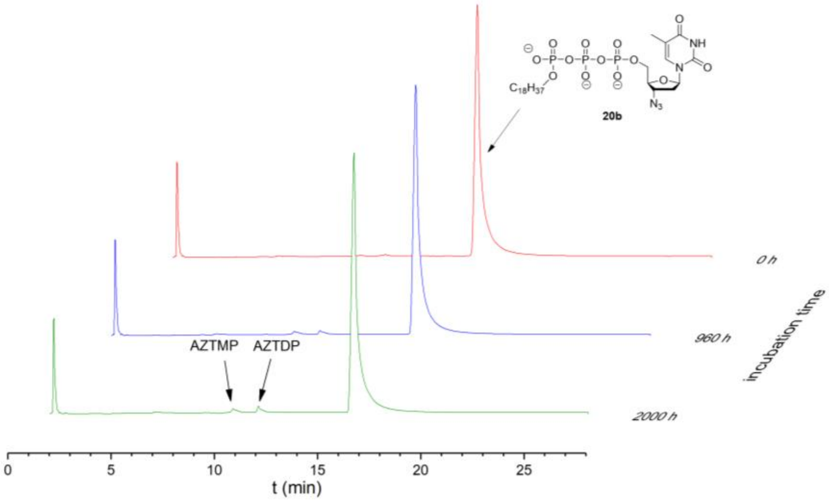

Figure S8. HPLC profiles of **20b** after incubation in PBS (pH 7.3).

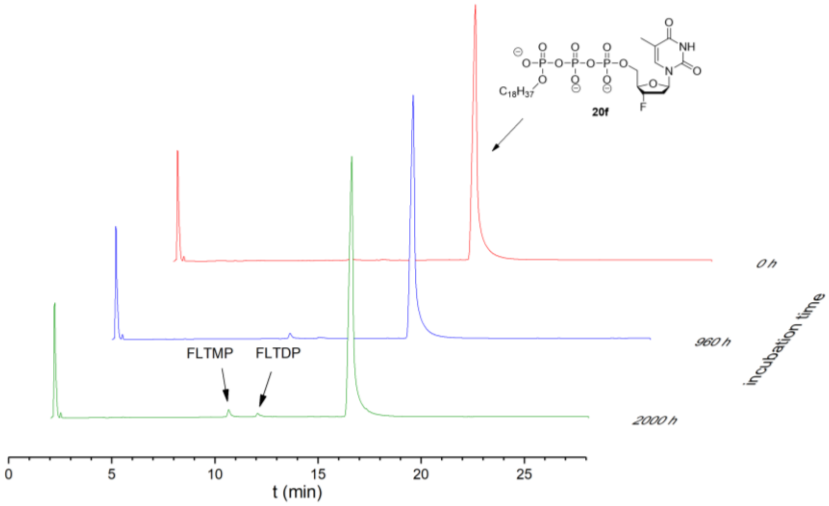

Figure S9. HPLC profiles of **20f** after incubation in PBS (pH 7.3).

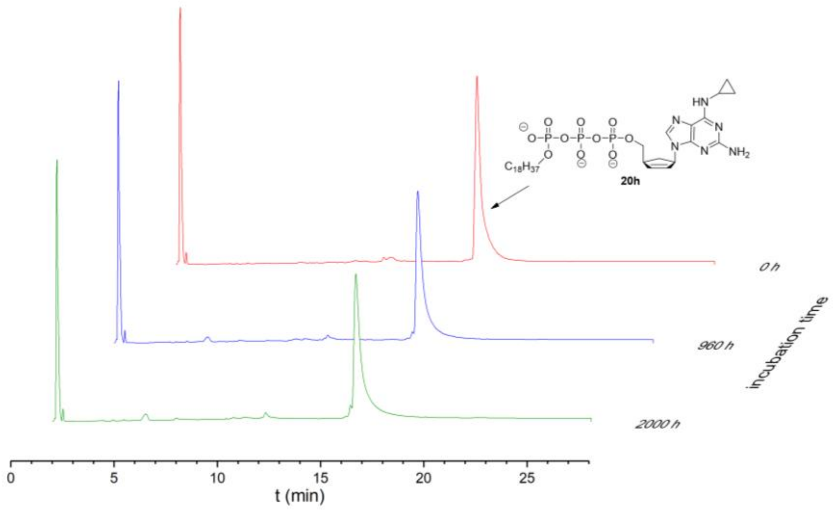

Figure S10. HPLC profiles of **20h** after incubation in PBS (pH 7.3).

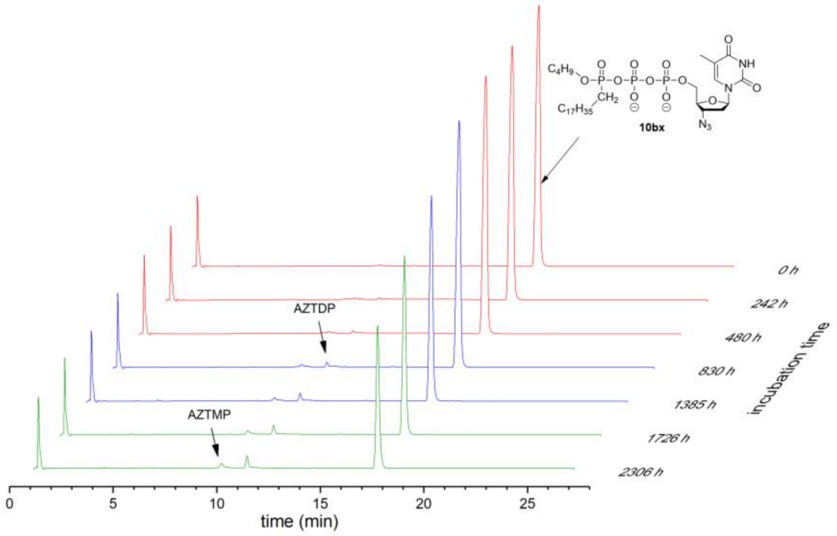

Figure S11. HPLC profiles of **10bx** after incubation in PBS (pH 7.3).

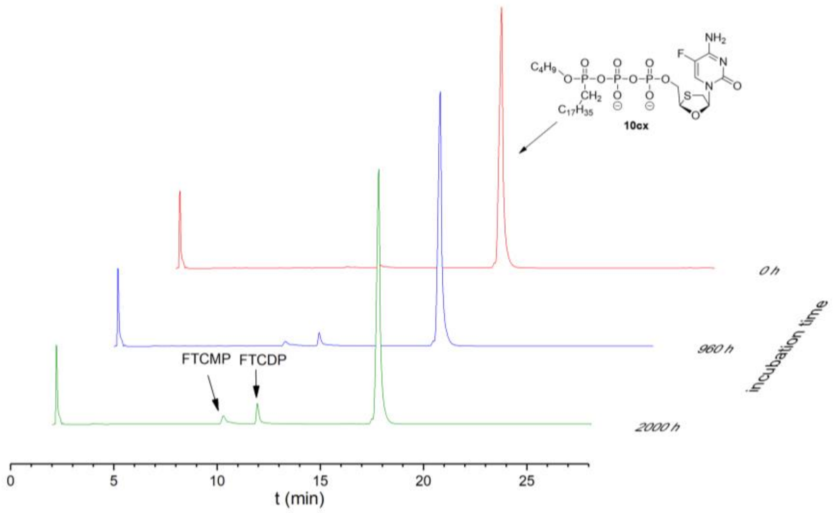

Figure S12. HPLC profiles of **10cx** after incubation in PBS (pH 7.3).

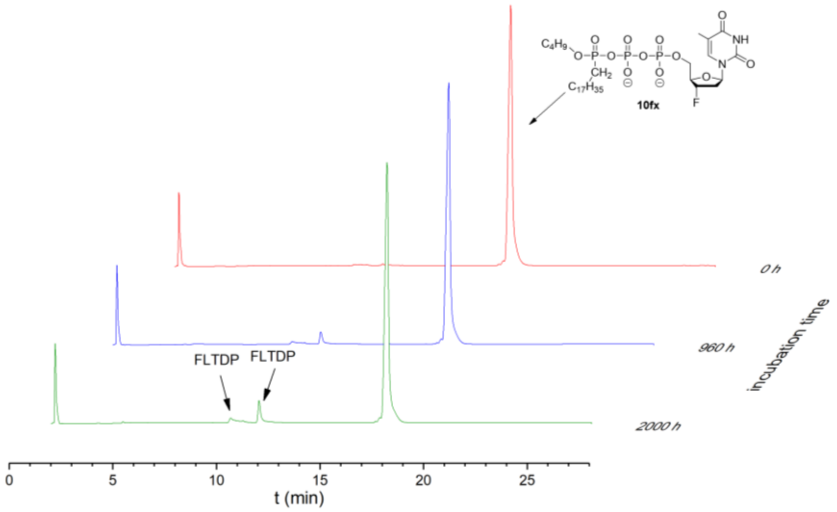

Figure S13. HPLC profiles of **10fx** after incubation in PBS (pH 7.3).

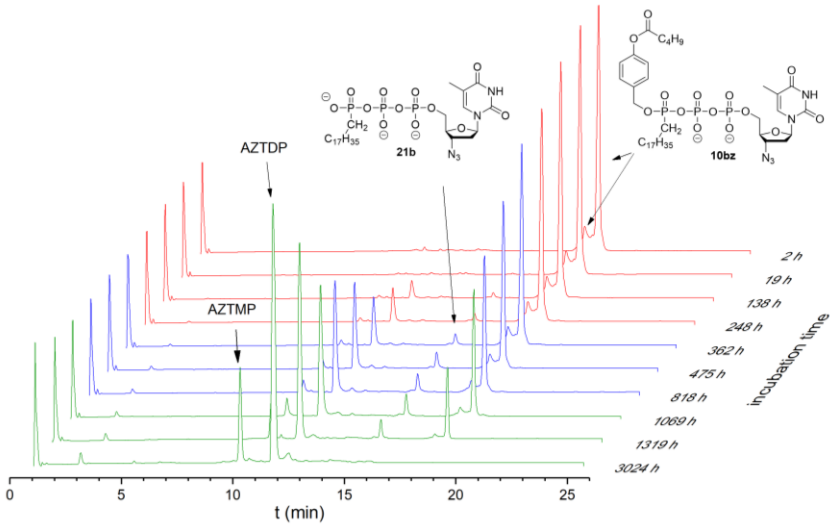

Figure S14. HPLC profiles of **10bz** after incubation in PBS (pH 7.3).

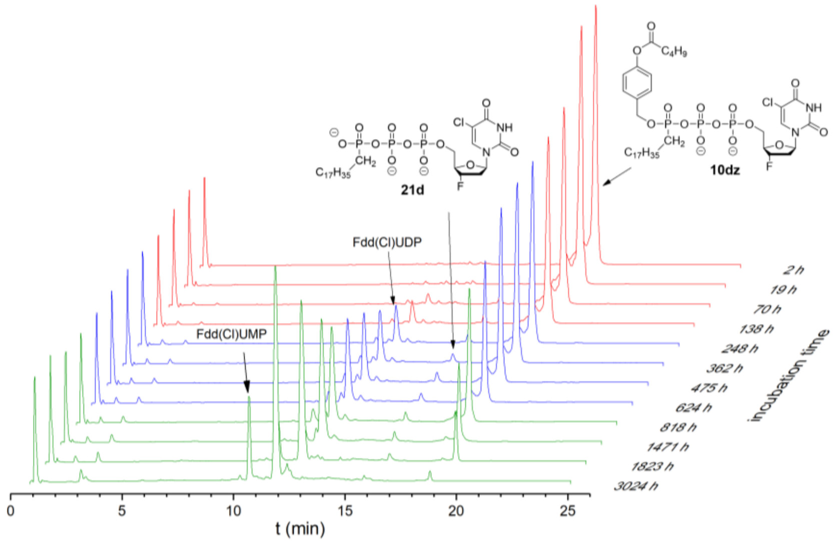

Figure S15. HPLC profiles of **10dz** after incubation in PBS (pH 7.3).

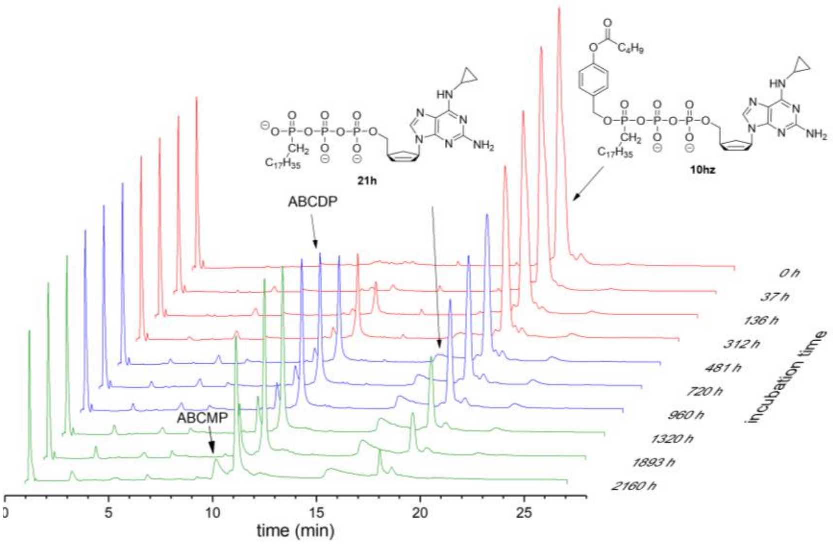

Figure S16. HPLC profiles of **10hz** after incubation in PBS (pH 7.3).

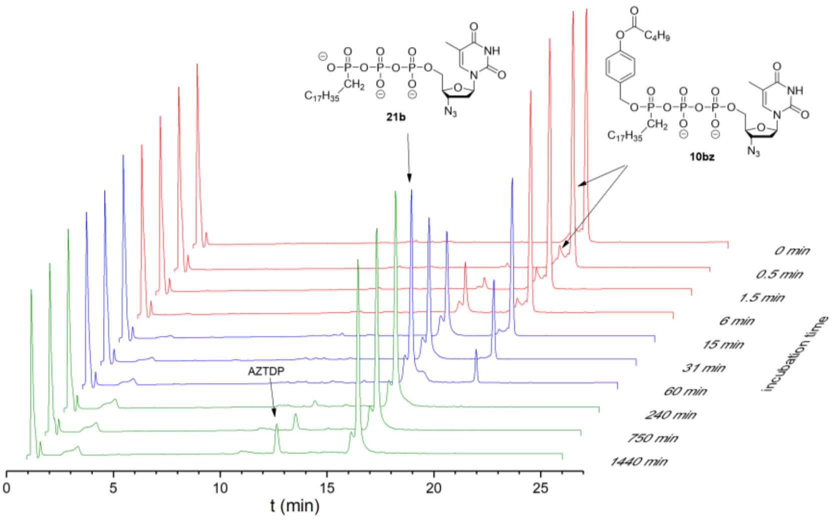

Figure S17. HPLC profiles of **10bz** after incubation with PLE.

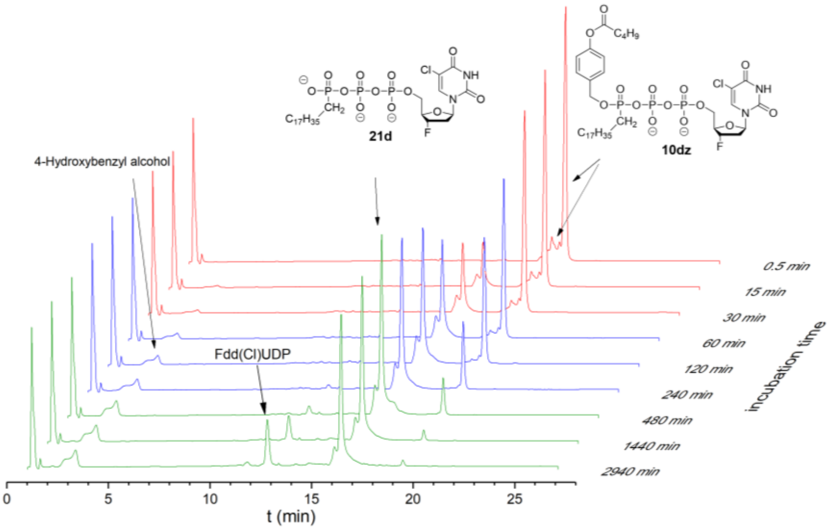

Figure S18. HPLC profiles of **10dz** after incubation with PLE.

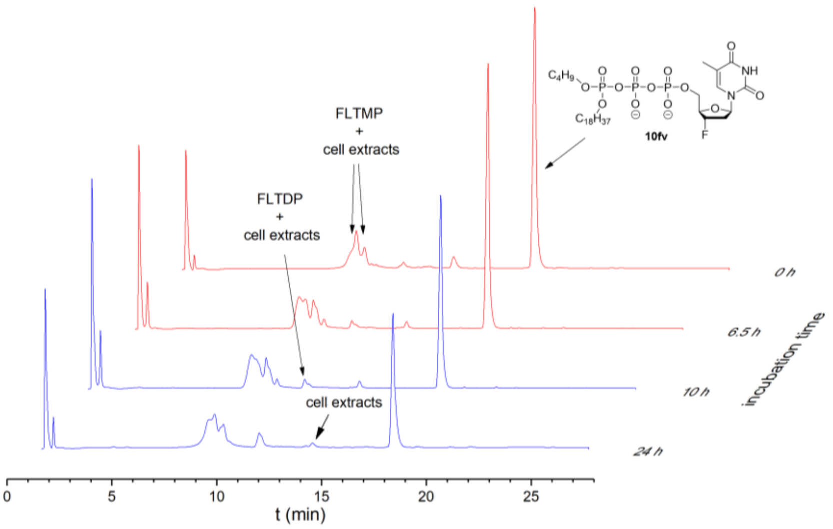

Figure S19. HPLC profiles of **10fv** after incubation in CEM/0 cell extracts.

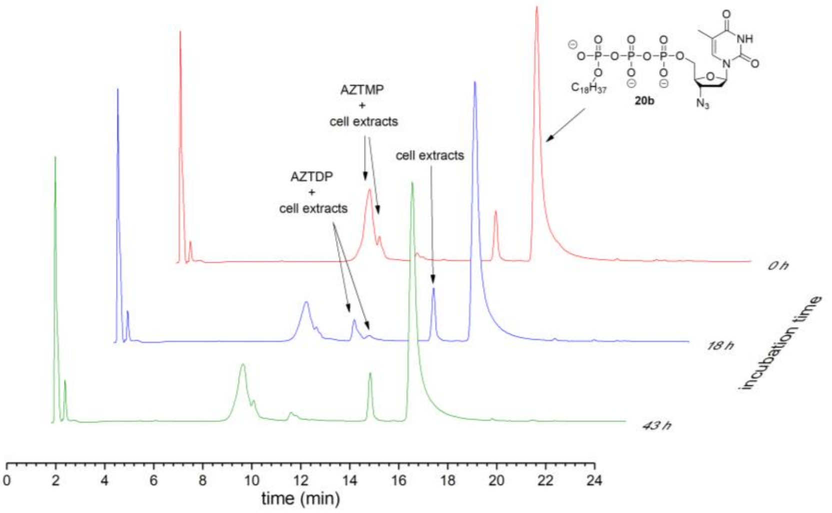

Figure S20. HPLC profiles of **20b** after incubation in CEM/0 cell extracts.

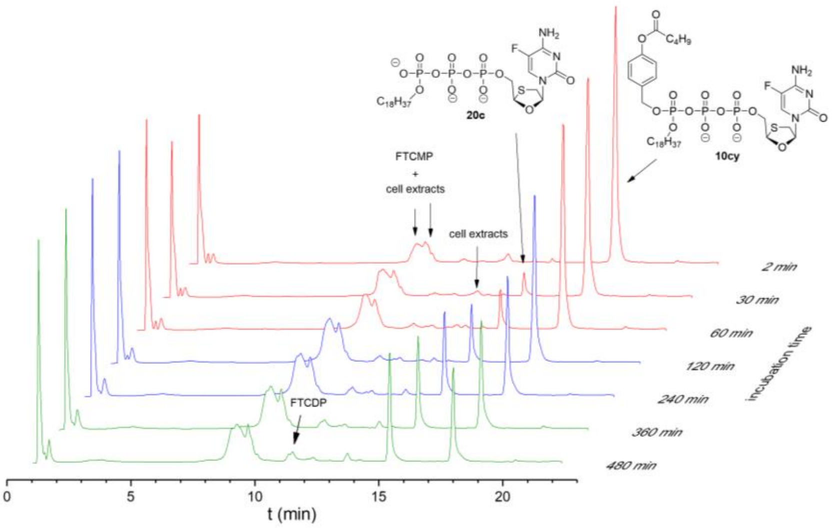

Figure S21. HPLC profiles of **10cy** after incubation in CEM/0 cell extracts.

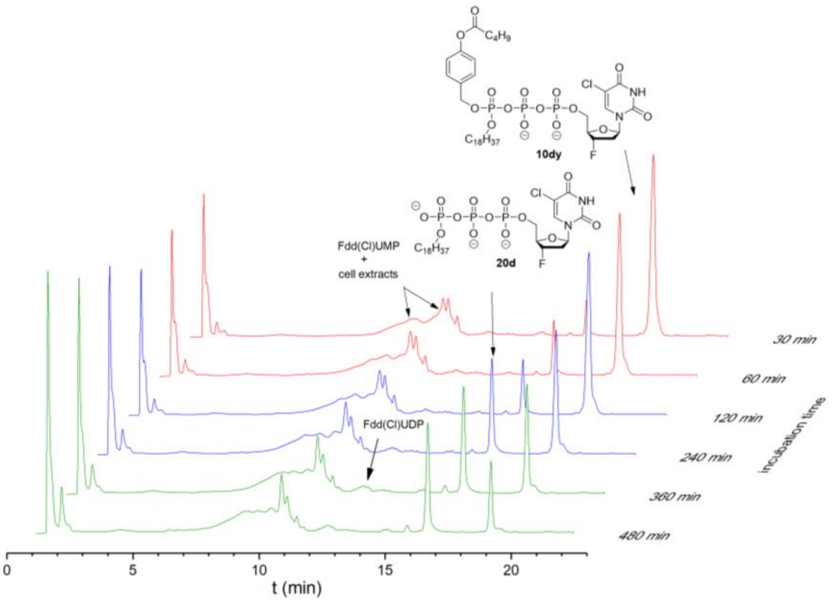

Figure S22. HPLC profiles of **10dy** after incubation in CEM/0 cell extracts.

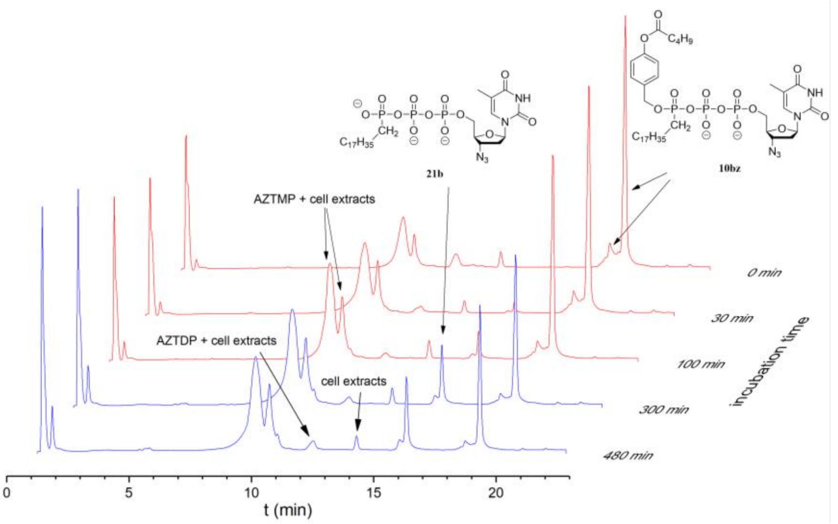

Figure S23. HPLC profiles of **10bz** after incubation in CEM/0 cell extracts.

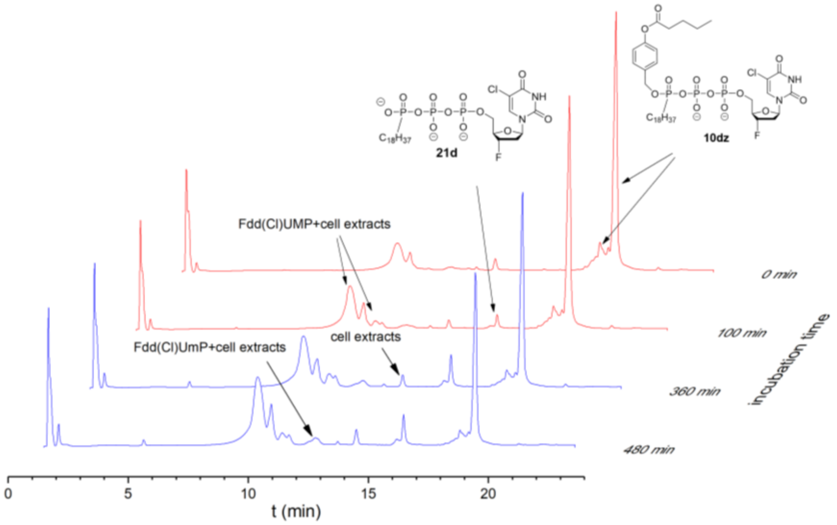

Figure S24. HPLC profiles of **10dz** after incubation in CEM/0 cell extracts.

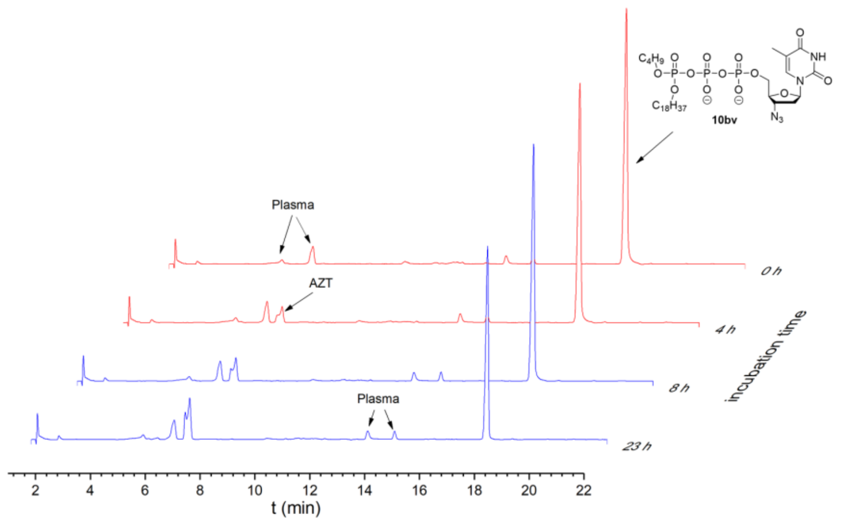

Figure S25. HPLC profiles of **10bv** after incubation in human heparin plasma.

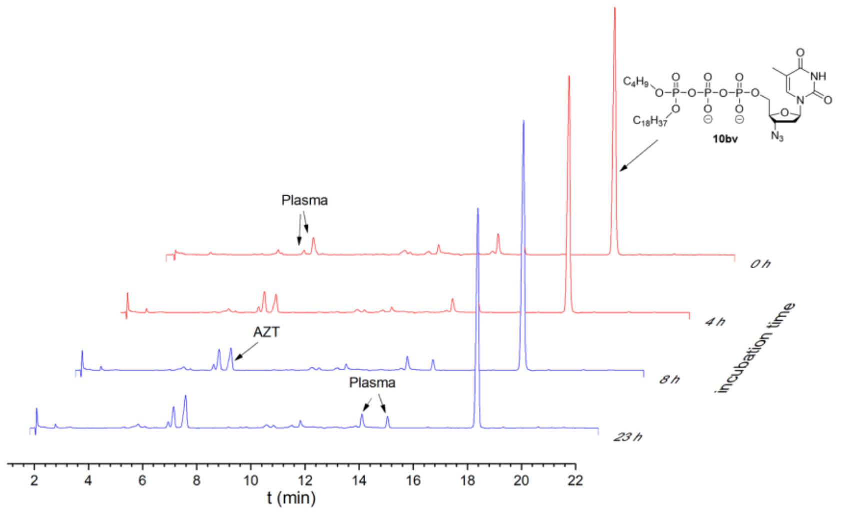

Figure S26. HPLC profiles of **10bv** after incubation in human citrate plasma.

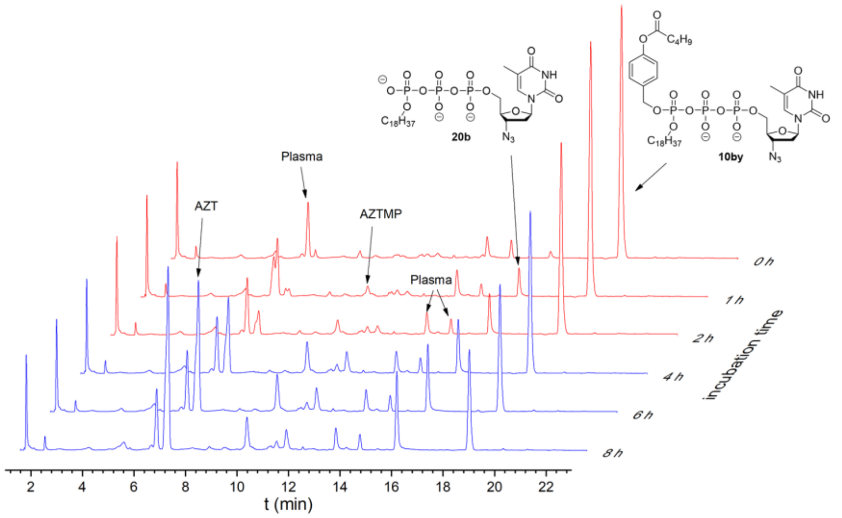

Figure S27. HPLC profiles of **10by** after incubation in human heparin plasma.

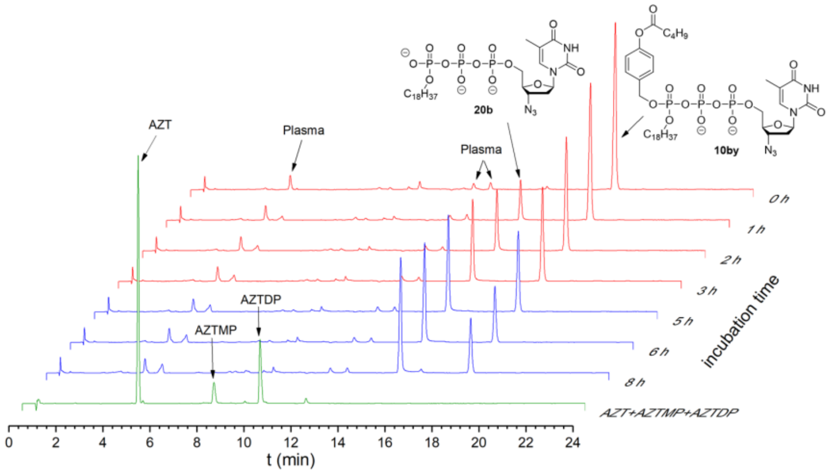

Figure S28. HPLC profiles of **10by** after incubation in human citrate plasma.

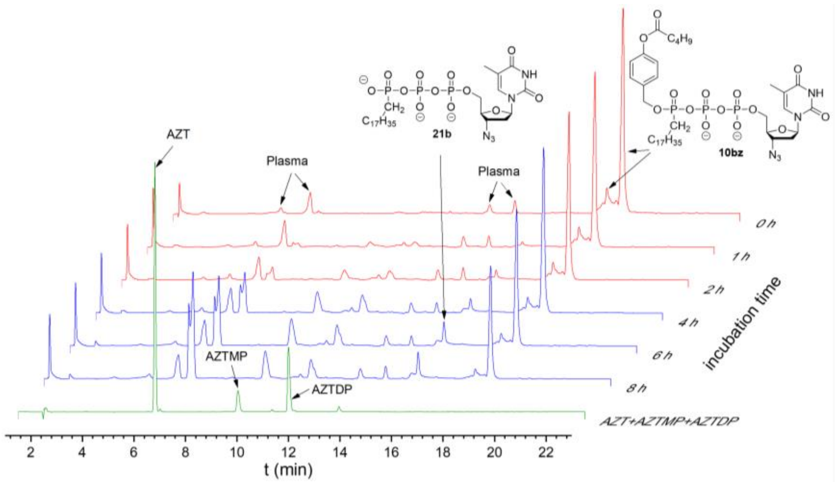

Figure S29. HPLC profiles of **10bz** after incubation in human heparin plasma.

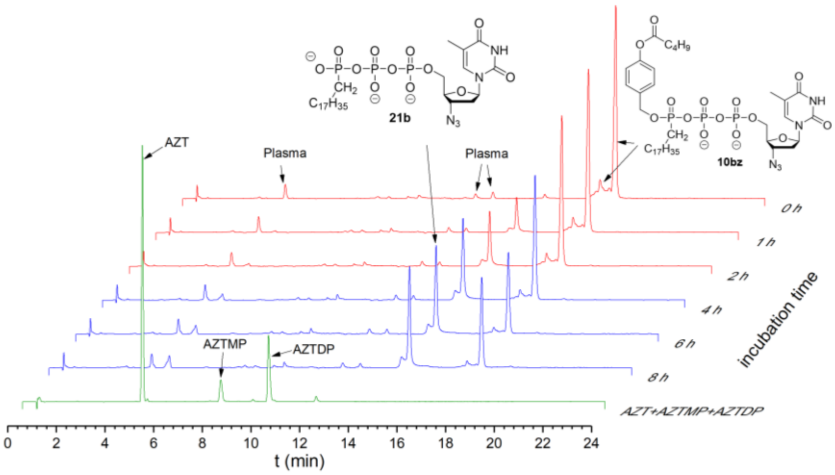

Figure S30. HPLC profiles of **10bz** after incubation in human citrate plasma.

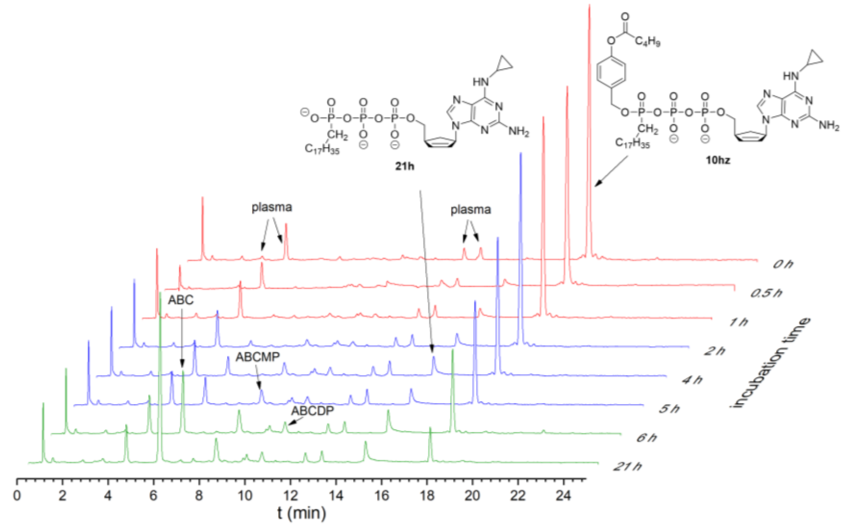

Figure S31. HPLC profiles of **10hz** after incubation in human heparin plasma.

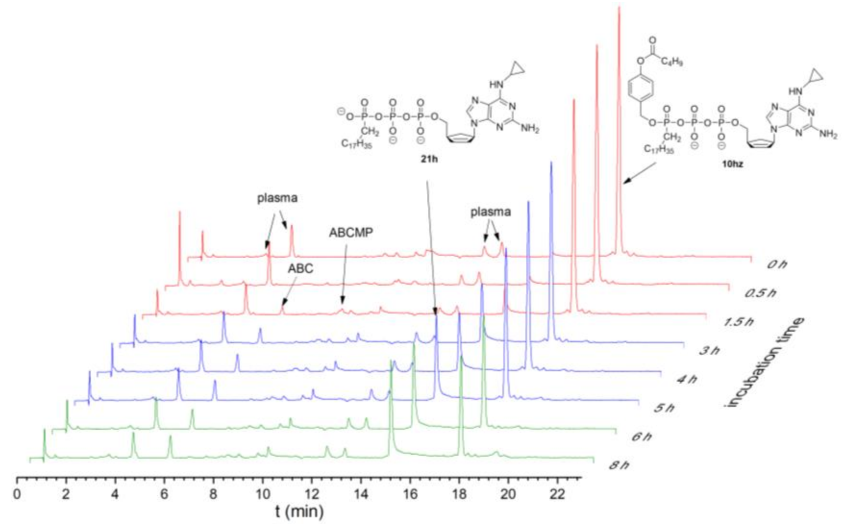

Figure S32. HPLC profiles of **10hz** after incubation in human citrate plasma.

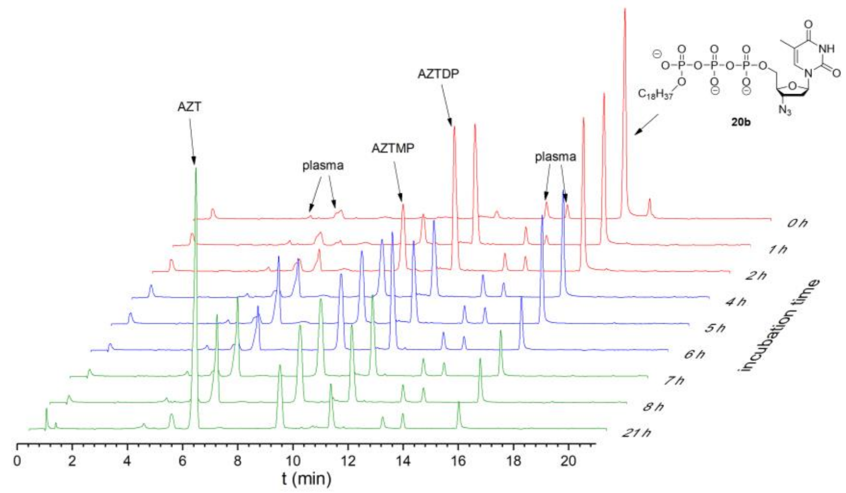

Figure S33. HPLC profiles of **20b** after incubation in human heparin plasma.

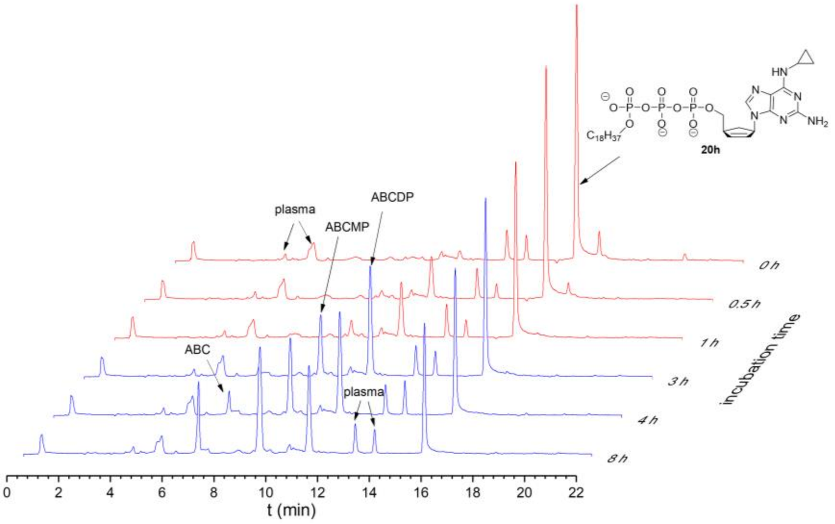

Figure S34. HPLC profiles of **20h** after incubation in human heparin plasma.

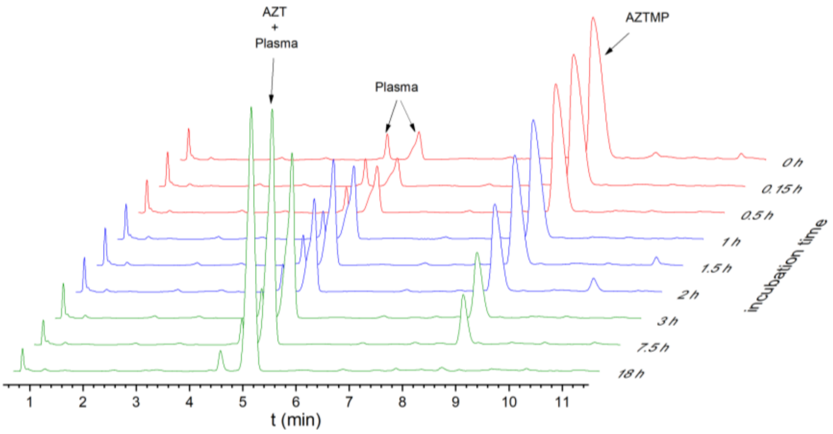

Figure S35. HPLC profiles of AZTMP after incubation in human heparin plasma.

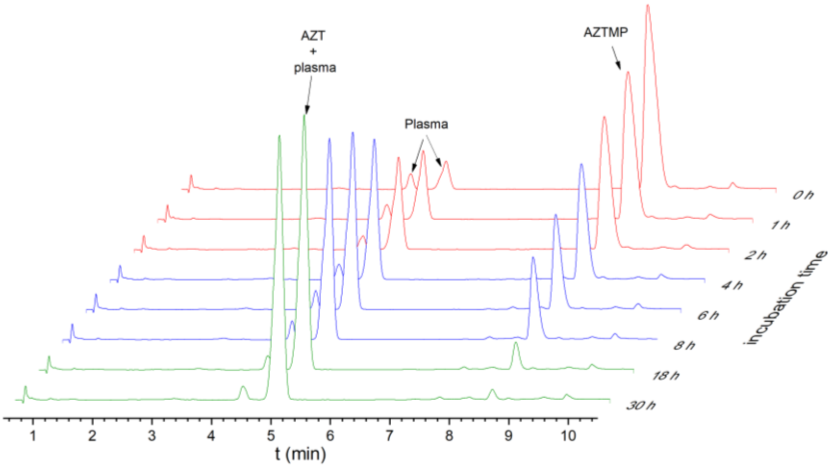

Figure S36. HPLC profiles of AZTMP after incubation in human citrate plasma.

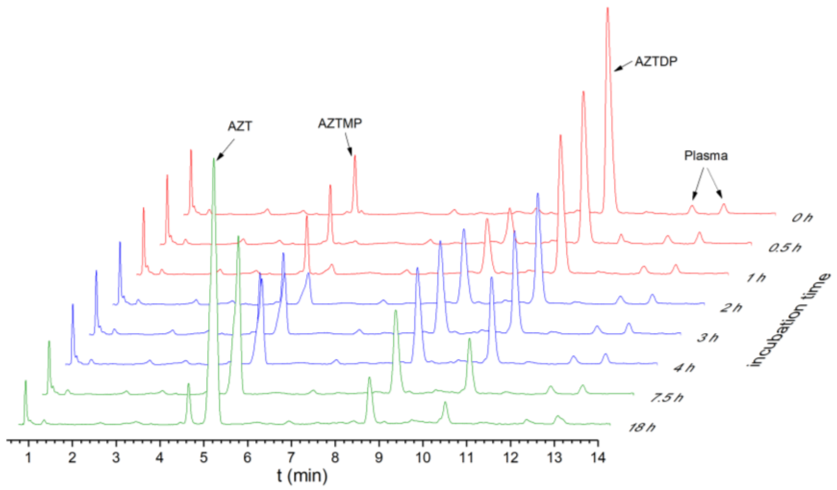

Figure S37. HPLC profiles of AZTDP after incubation in human heparin plasma.

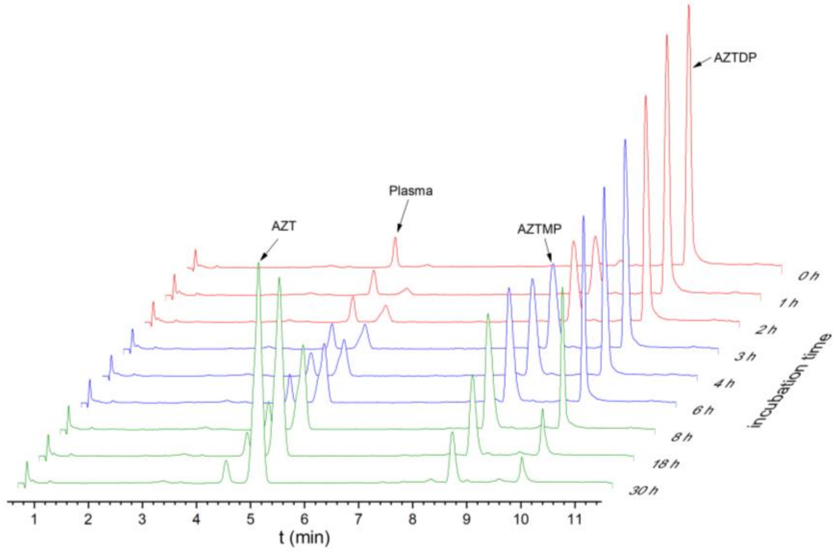

Figure S38. HPLC profiles of AZTDP after incubation in human citrate plasma.

Experimental Section.

General: All experiments were carried out under anhydride conditions and nitrogen atmosphere.

NMR spectra were measured on 400 MHz, 500 MHz and 600MHz instruments at room temperature. HRMS (ESI) mass spectra were measured on VG Analytical Finnigan ThermoQuest MAT 95 XL or Agilent 6224 EIS-TOF spectrometer. MALDI measurements were performed with a Bruker UltrafleXtreme spectrometer.

Chemical compounds were purchased as reagent grade and used without further purification. For product purification by flash column chromatography, silica gel 60 M (0.04-0.063 mm) was used. NMPs were synthesized by us using the previous reported protocols.[1-4] All prodrugs were purified by automatic reserved phase automated flash chromatography, various of Chromabond® Flash RS40 C_{18} ec were used in combination with an Interchim Puriflash 430 systhem.

High Performance Liquid Chromatography (HPLC) was used for analytical studies and monitoring reactions. A VWR-Hitachi LaChromElite HPLC system (L-2130, L-2200, L-2455) equipped with EzChromElite software was available. HPLC Method: Nucleodur 100-5 C18ec; 0-20 min: TBAA buffer/acetonitrile (HPLC grade) gradient (5-80%); 20-30 min: buffer/acetonitrile (80%); 30-33 min: buffer/acetonitrile (80-5%); 33-38 min:

buffer/acetonitrile (5%); flow: 1 mL/min. Buffer: 2 mM tetra-*n*-Butylammonium acetate solution (TBAA, pH 6.3). All target Tri*PPP*ro-compounds were analyzed for purity by RP-HPLC−UV and NMR spectroscopy. Purity of Tri*PPP*ro-compounds was > 95%.

Part 1. General Procedure 1: Preparation of Tri*PPP*ro-compounds 10 (method 1).

The reactions were performed in a nitrogen (N_2) atmosphere and dry conditions. a) *H*-phosphonates **15a**,**c** or *H*-phosphinates **15b**,**d** (0.3 mmol, 1.0 equiv.) were dissolved in 6 mL CH_3CN and 2 mL THF and *N*-chlorosuccinimide (NCS, 0.3 mmol, 1.0 equiv.) was added. After stirring for 4 h at room temperature, the corresponding NDP (0.5-0.7 equiv.) in 6 mL CH_3CN was added. The mixture was stirred for 3 h and the solvent was removed in vacuum. The crude product was purified by automatic RP18 flash chromatography, and then followed by ion-exchange to the ammonium form with Dowex 50WX8 cation-exchange resin and a second RP18 chromatography purification step. Product-containing fractions were collected and the organic solvent evaporated. The remaining aqueous solutions were freeze-dried and the desired product were obtained as white solids.

Part 2. General Procedure 2: Preparation of Tri*PPP*ro-compounds 10 (method 2).

The reactions were performed in a nitrogen (N_2) atmosphere and dry conditions. a) *H*-phosphonates **15a**,**c** or *H*-phosphinates **15b**,**d** (0.3 mmol, 1.0 equiv.) were dissolved in 6 mL CH_3CN and 2 mL THF and *N*-chlorosuccinimide (NCS, 0.6 mmol, 2.0 equiv.) was added. After stirring for 2 h at room temperature, tetrabutylammonium phosphate solution (0.4 M in acetonitrile) (0.9 mmol, 3.0 equiv.) was added quickly. The mixture was stirred for 1 h and the solvent was removed in vacuum. The residue was extracted with CH_2Cl_2/H_2O. The organic phase was dried over sodium sulfate and the solvent was removed by evaporation to afford pyrophosphates or phosphonate-phosphates in almost quantitative yield. b) The corresponding pyrophosphate or phosphonate-phosphates was dissolved in 6 mL CH_3CN and cooled down to 0 °C. A mixture of trifluoroacetic anhydride (TFAA,1.5 mmol, 5.0 equiv.) and Et_3N (2.4 mmol, 8.0 equiv.) in 6 mL CH_3CN was cooled to 0 °C and added to the mixture. After stirring for 10 min, all volatile components were removed in vacuum. The residue was subsequently dissolved in 8 mL CH_3CN (or added THF) at 0 °C. 1-Methylimidazole (0.9 mmol, 3.0 equiv.) and Et_3N (TEA, 1.5 mmol, 5.0 equiv.) was added. The mixture was warmed to room temperature and stirred for 10 min. The resulting activated imidazolidate formed and the corresponding NMP (0.7 equiv.) in 6 mL CH_3CN was added, and the mixture was stirred at rt for 3-5 h. The solvent was removed in vacuum and the crude product was purified by automatic RP18 flash chromatography (H_2O/CH_3CN or H_2O/THF). After ion-exchange Dowex 50WX8 cation-exchange resin and freeze-drying, the crude product was purified by automatic RP18 flash chromatography again. Product-containing fractions were collected and the organic solvent evaporated. The remaining aqueous solutions were freeze-dried and the desired products were obtained as solids.

Part 3. Synthesis and Characterization.

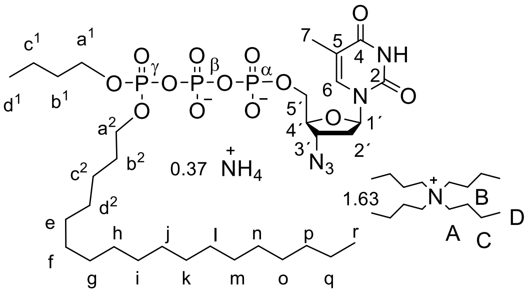

10bv

γ-(C4;C18)-AZTTP 10bv. According to general procedure 1 with 117 mg *H*-phosphonate **15ca** (0.3 mmol, 1.0 equiv.), 40 mg NCS (0.3 mmol, 1.0 equiv.) and 204 mg AZTDP 2.25×nBu$_4$N$^+$ salt (0.21 mmol, 0.7 equiv.). Reaction time was 3 h at room temperature. Yield: 120 mg (0.10 mmol, 47%) white solid. HPLC-UV analysis confirmed purity: > 98%. **^{1}H NMR (400 MHz, CD$_3$OD):** δ [ppm] = 7.91 (d, $^4J_{HH}$= 1.1 Hz, 1H, H-6), 6.27 (dd, $^3J_{HH}$= 8.8 Hz, $^4J_{HH}$=5.5 Hz, 1H, H-1´), 4.77-4.72 (m, 1H, H-3´), 4.35–4.12 (m, 6H, H-5´, H-a^1, H-a^2), 4.12-4.06 (m, 1H, H-4´), 3.28–3.20 (m, 13H, H-A), 2.60–2.45 (m, 1H, H-2´a), 2.32–2.22 (m, 1H, H-2´b), 1.95 (d, $^4J_{HH}$=1.1 Hz, 3H, H-7), 1.74-1.58 (m, 17H, H-B, H-b^1, H-b^2), 1.50-1.35 (m, 17H, H-C, H-c^1, H-c^2), 1.32-1.26 (m, 28H, H-d^2, H-e, H-f, H-j, H-h, H-i, H-j, H-k, H-l, H-m, H-n, H-o, H-p, H-q), 1.02 (t, $^3J_{HH}$= 7.3 Hz, 19.5H, H-D), 0.98-0.86 (m, 6H, H-d^1, H-r). **^{13}C NMR (101 MHz, CD$_3$OD):** δ [ppm] = 166.5 (C-4), 152.5 (C-2), 138.2 (C-6), 112.4 (C-5), 85.8 (C-1´), 85.0 (d, $^3J_{CP}$= 9.6 H$_Z$, C-4´), 69.5, 69.2 (2 × d, $^3J_{CP}$= 6.7 H$_Z$, $^3J_{CP}$= 6.2 H$_Z$, C-a^1, C-a^2), 67.2 (d, $^3J_{CP}$= 5.7 H$_Z$, C-5´), 63.7 (C-3´), 59.5 (t, $^3J_{CP}$= 2.8 H$_Z$, C-A), 37.9 (C-2´), 33.4 (t, $^3J_{CP}$= 7.4 Hz, C-b^1), 31.3 (t, $^3J_{CP}$= 7.4 Hz, C-b^2), 33.47, 33.40, 33.1, 30.78, 30.75, 30.71, 30.5, 30.4, 23.7 (C-d^2, C-e, C-f, C-g, C-h, C-i, C-j, C-k, C-l, C-m, C-n, C-o, C-p, C-q), 26.6 (C-c^2), 24.8 (C-B), 20.7 (t, $^4J_{CP}$= 1.4 H$_Z$, C-C), 19.8 (C-c^1), 14.4 (C-r), 14.0 (C-d^1). 13.9 (C-D), 12.7 (C-7). **^{31}P NMR (162 MHz, CD$_3$OD):** δ [ppm] = -8.40 (d, $^2J_{pp}$=19.6 Hz, P-α), -9.08 (d, $^2J_{pp}$=17.6 Hz, P-γ), -20.39 (t, $^2J_{pp}$= 17.7 Hz, P-β). **MALDI-MS (m/z):** calculated for $C_{32}H_{60}N_5O_{13}P_3$ [M-H]$^-$; 814.333 found, 814.178.

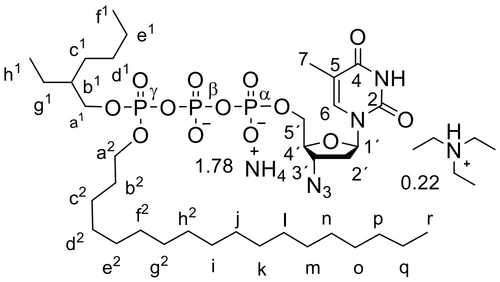

10bw

γ-(C8;C18)-AZTTP 10bw. According to general procedure 1 with 134 mg *H*-phosphonate **15cb** (0.3 mmol, 1.0 equiv.), 40 mg NCS (0.3 mmol, 1.0 equiv.) and 146 mg AZTDP 2.25×nBu$_4$N$^+$ salt (0.15 mmol, 0.5 equiv.). Reaction time was 3 h at room temperature. Yield: 28 mg (0.03 mmol, 20%) white solid. HPLC-UV analysis confirmed purity: > 98%. **^{1}H NMR (400 MHz, CD$_3$OD):** δ [ppm] = 7.83 (s, 1H, H-6), 6.30-6.20 (m, 1H, H-1´), 4.64-4.54 (m, 1H, H-3´), 4.38-3.96 (m, 7H, H-4´, H-5´, H-a^1, H-a^2), 3.02 (q, $^3J_{HH}$= 7.1 Hz, 1.32H, HN(CH$_2$CH$_3$)$_3$$^+$), 2.54-2.40 (m, 1H, H-2´$_a$), 2.38-2.24 (m, 1H, H-2´$_b$), 1.94 (s, 3H, H-7), 1.76-1.64 (m, 2H, H-b^2), 1.62-1.54 (m, 1H, H-b^1), 1.45-1.25 (m, 39.98H, HN(CH$_2$CH$_3$)$_3$$^+$, H-c^1, H-c^2, H-d^1, H-d^2, H-e^1, H-e^2, H-f^2, H-g^1, H-g^2, H-h^2, H-i, H-j, H-k, H-l, H-m, H-n, H-o, H-p, H-q), 1.02-0.82 (m, 9H, H-f^1, H-h^1, H-r). **^{13}C NMR (101 MHz, CD$_3$OD):** δ [ppm] = 166.4 (C-4), 152.4 (C-2), 137.9 (C-6), 112.3 (C-5), 85.8 (C-1´), 84.7 (d, $^3J_{CP}$= 9.7 Hz, C-4´), 71.61, 71.59, 71.57 (C-a^1), 69.7 (d, $^3J_{CP}$= 5.5 Hz, C-a^2), 67.1 (d, $^3J_{CP}$= 5.5 Hz, C-5´), 63.2 (C-3´), 47.5 (HN(CH$_2$CH$_3$)$_3$$^+$), 41.4 (d, $^3J_{CP}$= 7.8 Hz, C-b^1), 38.0 (C-2´), 33.1, 31.37, 31.32, 31.04, 31.03, 30.78, 30.75, 30.70, 30.5, 30.3, 30.06, 30.03, 24.32, 24.29, 24.1, 23.7 (C-b^2, C-c^1, C-d^1, C-d^2, C-e^1, C-e^2, C-f^2, C-g^1, C-g^2, C-h^2, C-i, C-j, C-k, C-l, C-m, C-n, C-o, C-p, C-q), 26.7 (C-c^2), 14.4 (C-f^1, C-r), 12.6 (C-7), 11.3 (d, $^4J_{CP}$= 1.3 Hz, C-h^1), 9.2 (HN(CH$_2$CH$_3$)$_3$$^+$). **^{31}P NMR (162 MHz, CD$_3$OD):** δ [ppm] = -11.94 (d, $^2J_{pp}$=17.5 Hz, P-γ), -12.55 (d, $^2J_{pp}$=17.6

Hz, P-α), -23.61 (t, $^2J_{pp}$= 17.5 Hz, P-β). **HRMS (ESI$^-$, m/z):** calculated for $C_{36}H_{68}N_5O_{13}P_3$ [M-H]$^-$ 870.3954; found, 870.3848.

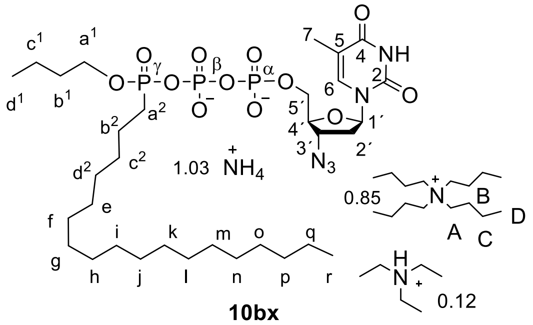

10bx

γ-(C4)-γ-C-(C18)-AZTTP 10bx. According to general procedure 1 with 112 mg *H*-phosphinate **15d** (0.3 mmol, 1.0 equiv.), 40 mg NCS (0.3 mmol, 1.0 equiv.) and 204 mg AZTDP 2.25×nBu$_4$N$^+$ salt (0.21 mmol, 0.7 equiv.). Reaction time was 3 h at room temperature. Yield: 108 mg (0.11 mmol, 50%) white solid. HPLC-UV analysis confirmed purity: > 99%. **^{1}H NMR (400 MHz, CD$_3$OD):** δ [ppm] = 7.86 (d, $^4J_{HH}$= 1.2 Hz, 1H, H-6), 6.27 (dd, $^3J_{HH}$= 8.4 Hz, $^4J_{HH}$=5.7 Hz, 1H, H-1´), 4.71-4.64 (m, 1H, H-3´), 4.32–4.12 (m, 4H, H-5´, H-a^1), 4.12-4.06 (m, 1H, H-4´), 3.28–3.20 (m, 6.8H, H-A), 3.20-3.15 (m, 0.72H, HN(CH$_2$CH$_3$)$_3^+$), 2.55–2.43 (m, 1H, H-2´a), 2.35–2.25 (m, 1H, H-2´b), 2.12–1.97 (m, 1H, H-a^2), 1.95 (d, $^4J_{HH}$=1.1 Hz, 3H, H-7), 1.74-1.57 (m, 10.8H, H-B, H-b^1, H-b^2), 1.50-1.36 (m, 10.8H, H-C, H-c^1, H-c^2), 1.35-1.26 (m, 29.08H, HN(CH$_2$CH$_3$)$_3^+$, H-d^2, H-e, H-f, H-j, H-h, H-i, H-j, H-k, H-l, H-m, H-n, H-o, H-p, H-q), 1.03 (t, $^3J_{HH}$= 7.3 Hz, 10.2H, H-D), 0.98-0.86 (m, 6H, H-d^1, H-r). **^{13}C NMR (101 MHz, CD$_3$OD):** δ [ppm] = 166.4 (C-4), 152.5 (C-2), 138.0 (C-6), 112.3 (C-5), 85.8 (C-1´), 84.8 (d, $^3J_{CP}$= 9.9 Hz, C-4´), 67.1, 66.8 (2 × d, $^3J_{CP}$= 7.4 Hz, $^3J_{CP}$= 5.6 Hz, C-a^1, C-5´), 63.4 (C-3´), 59.5 (t, $^3J_{CP}$= 2.8 Hz, C-A), 47.4 (HN(CH$_2$CH$_3$)$_3^+$), 37.9 (C-2´), 33.6, 33.5, 33.1, 31.7, 31.5, 30.78, 30.75, 30.72, 30.6, 30.5, 30.3, 23.7, 19.9, 14.4, 14.0, 11.6 (C-c^2, C-d^2, C-e, C-f, C-g, C-h, C-i, C-j, C-k, C-l, C-m, C-n, C-o, C-p, C-q), 27.3, 26.2 (C-a^2), 24.8 (C-B), 23.4 (d, $^3J_{CP}$= 5.3 Hz, C-b^2), 20.7 (t, $^4J_{CP}$= 1.4 Hz, C-C), 13.9 (C-D), 12.7 (C-7), 9.1 (HN(CH$_2$CH$_3$)$_3^+$). **^{31}P NMR (162 MHz, CD$_3$OD):** δ [ppm] = 27.88 (d, $^2J_{pp}$=23.4 Hz, P-γ), -8.11 (d, $^2J_{pp}$=19.5 Hz, P-α), -19.91 (t, $^2J_{pp}$= 21.5 Hz, P-β). **HRMS (ESI$^-$, m/z):** calculated for $C_{32}H_{60}N_5O_{12}P_3$ [M-H]$^-$; 798.338 found, 798.221.

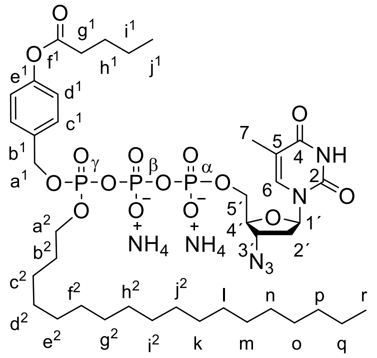

10by

γ-(AB-C4;C18)-AZTTP 10by. According to general procedure 2 with 157 mg *H*-phosphonate **15a** (0.3 mmol, 1.0 equiv.), 80 mg NCS (0.6 mmol, 2.0 equiv.), and 174 mg (*n*-Bu$_4$N)$_2$·AZTMP salt (0.21 mmol, 0.7 equiv.). Reaction time was 3 h. Yield: 167 mg (0.17 mmol, 81%) white solid. HPLC-UV analysis confirmed purity: > 95%. **^{1}H NMR (600 MHz, CD$_3$OD):** δ [ppm] = 7.79 (s, 1H, H-6), 7.52-7.46 (m, 2H, H-c^1), 7.12-7.06 (m, 2H, H-d^1), 6.23 (dd, $^3J_{HH}$= 7.9 Hz, $^3J_{HH}$=6.1 Hz, 1H, H-1´), 5.19 (d, $^3J_{HH}$= 8.3 Hz, 4H, H-a^1), 4.62-4.55 (m, 1H, H-3´), 4.26-4.18 (m, 2H, H-5´), 4.16-4.05 (m, 3H, H-4´, H-a^2), 2.58 (t, $^3J_{HH}$= 7.4 Hz, 2H, H-g^1), 2.48-2.36 (m, 1H, H-2´a), 2.33-2.23 (m, 1H, H-2´b), 1.92 (d, $^3J_{HH}$= 2.5 Hz, 3H, H-7), 1.73 (quint, $^3J_{HH}$= 7.4 Hz, 2H, H-h^1), 1.62 (quint, $^3J_{HH}$= 6.7 Hz, 2H, H-b^2), 1.46 (sext, $^3J_{HH}$= 7.5 Hz, 2H, H-i^1), 1.36-1.26 (m, 30H, H-c^2, H-d^2, H-e^2, H-f^2, H-j^2, H-h^2, H-i^2, H-j^2, H-k, H-l, H-m, H-n, H-o, H-p, H-q), 0.98 (t, $^3J_{HH}$= 7.3 Hz, 3H, H-j^1), 0.89 (t, $^3J_{HH}$= 6.8 Hz, 3H, H-r). **^{13}C-NMR (151 MHz, CD$_3$OD):** δ [ppm] = 173.6 (C-f^1), 163.7 (C-4), 152.4 (C-2), 152.3 (C-e^1), 137.9 (C-6), 135.1 (d, $^3J_{CP}$= 7.3 Hz, C-b^1), 130.3 (d, $^3J_{CP}$= 1.8 Hz, C-c^1), 122.8 (C-d^1), 112.2 (C-5), 85.9 (C-1´), 84.6 (d, $^3J_{CP}$= 9.6 Hz, C-4´), 70.2 (dd, $^3J_{CP}$= 5.5 Hz, $^3J_{CP}$= 2.7 Hz, C-a^1), 69.8 (d, $^3J_{CP}$= 6.6 Hz, C-a^2), 67.0 (d,

$^{3}J_{CP}$= 5.5 Hz, C-5´), 63.0 (C-3´), 37.9 (C-2´), 34.8 (C-g^{1}), 31.2 (d, $^{3}J_{CP}$= 7.5 Hz, C-b^{2}), 33.0, 30.78, 30.73, 30.71, 30.65, 30.4, 30.3, 23.7 (C-d^{2}, C-e^{2}, C-f^{2}, C-g^{2}, C-h^{2}, C-i^{2}, C-j^{2}, C-k, C-l, C-m, C-n, C-o, C-p, C-q), 28.0 (C-h^{1}), 26.5 (C-c^{2}), 23.2 (C-i^{1}), 14.5 (C-r), 14.1 (C-j^{1}), 12.6 (C-7). **^{31}P NMR (162 MHz, CD_3OD):** δ [ppm] = -11.85 (d, $^{2}J_{pp}$= 17.6 Hz, P-α), -12.86 (dd, $^{2}J_{pp}$= 15.8 Hz, $^{3}J_{pp}$= 4.0 Hz, P-γ), -23.55 (t, $^{2}J_{pp}$= 16.4 Hz, P-β). **MALDI-MS (m/z):** calculated for $C_{40}H_{66}N_5O_{15}P_3$ [M-H]$^-$ 948.370; found, 948.340.

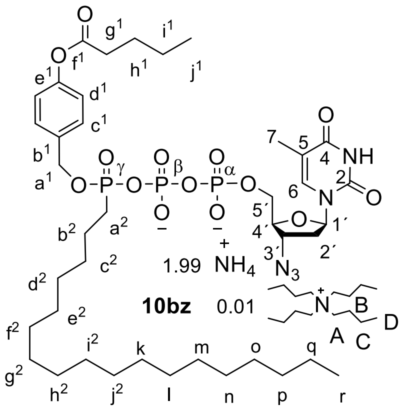

γ-(AB-C4)-γ-C-(C18)-AZTTP 10bz. According to general procedure 2 with 153 mg *H*-phosphinate **15b** (0.3 mmol, 1.0 equiv.), 80 mg NCS (0.6 mmol, 2.0 equiv.), and 174 mg (n-$Bu_4N)_2$·AZTMP (0.21 mmol, 0.7 equiv.). Reaction time was 3 h. Yield: 82 mg (0.08 mmol, 40%) white solid. HPLC-UV analysis confirmed purity: > 98%. **^{1}H NMR (600 MHz, CD_3OD):** δ [ppm] = 7.80 (d, $^{4}J_{HH}$= 1.2 Hz, 1H, H-6), 7.52-7.47 (m, 2H, H-c^{1}), 7.12-7.05 (m, 2H, H-d^{1}), 6.23 (dd, $^{3}J_{HH}$= 7.3 Hz, $^{3}J_{HH}$=5.9 Hz, 1H, H-1´), 5.28-5.15 (m, 2H, H-a^{1}), 4.62-4.55 (m, 1H, H-3´), 4.28-4.18 (m, 2H, H-5´), 4.10-4.04 (m, 1H, H-4´), 3.28-3.18 (m, 0.08H, H-A), 2.58 (t, $^{3}J_{HH}$= 7.4 Hz, 2H, H-g^{1}), 2.50-2.36 (m, 1H, H-2´a), 2.35-2.25 (m, 1H, H-2´b), 2.08-1.96 (m, 2H, H-a^{2}), 1.92 (dd, $^{3}J_{HH}$= 2.0 Hz, $^{4}J_{HH}$= 1.3 Hz, 3H, H-7), 1.73 (quint, $^{3}J_{HH}$= 7.4 Hz, 2H, H-h^{1}), 1.64-1.54 (m, 2.08H, H-B, H-b^{2}), 1.49 (sext, $^{3}J_{HH}$= 7.5 Hz, 2H, H-i^{1}), 1.42-1.24 (m, 30.08H, H-C, H-c^{2}, H-d^{2}, H-e^{2}, H-f^{2}, H-j^{2}, H-h^{2}, H-i^{2}, H-j^{2}, H-k, H-l, H-m, H-n, H-o, H-p, H-q), 0.12 (t, $^{3}J_{HH}$= 7.3 Hz, 0.12H, H-D), 0.98 (t, $^{3}J_{HH}$= 7.3 Hz, 3H, H-j^{1}), 0.89 (t, $^{3}J_{HH}$= 6.8 Hz, 3H, H-r). **^{13}C-NMR (151 MHz, CD_3OD):** δ [ppm] = 173.7 (C-f^{1}), 166.4 (C-4), 152.4 (C-2), 152.2 (d, $^{4}J_{CP}$= 0.8 Hz, C-e^{1}), 137.9 (C-6), 135.5 (dd, $^{3}J_{CP}$= 7.2 Hz, $^{4}J_{CP}$= 1.4 Hz, C-b^{1}), 130.3 (d, $^{3}J_{CP}$= 3.6 Hz, C-c^{1}), 122.8 (C-d^{1}), 112.2 (C-5), 85.9 (C-1´), 84.6 (d, $^{3}J_{CP}$= 9.2 Hz, C-4´), 67.9 (dd, $^{3}J_{CP}$= 7.0 Hz, $^{3}J_{CP}$= 2.7 Hz, C-a^{1}), 67.0 (d, $^{3}J_{CP}$= 5.6 Hz, C-5´), 63.0 (C-3´), 59.5 (C-A), 37.9 (C-2´), 34.8 (C-g^{1}), 33.1, 31.6, 31.4, 30.79, 30.74, 30.70, 30.6, 30.53, 30.46, 30.2, 23.7, 9.1 (C-d^{2}, C-e^{2}, C-f^{2}, C-g^{2}, C-h^{2}, C-i^{2}, C-j^{2}, C-k, C-l, C-m, C-n, C-o, C-p, C-q), 28.1 (C-h^{1}), 28.0, 26.1 (C-a^{2}), 24.8 (C-B), 23.31, 23.23 (C-b^{2}, C-i^{1}), 20.7 (C-C), 14.4 (C-r), 14.1 (C-j^{1}). 13.9 (C-D), 12.6 (C-7). **^{31}P NMR (162 MHz, CD_3OD):** δ [ppm] = 24.54 (d, $^{2}J_{pp}$=25.4 Hz, P-γ), -11.84 (d, $^{2}J_{pp}$=19.5 Hz, P-α), -23.44 (t, $^{2}J_{pp}$= 21.5 Hz, P-β). **MALDI-MS (m/z):** calculated for $C_{40}H_{66}N_5O_{14}P_3$ [M-H]$^-$ 932.375; found, 932.327.

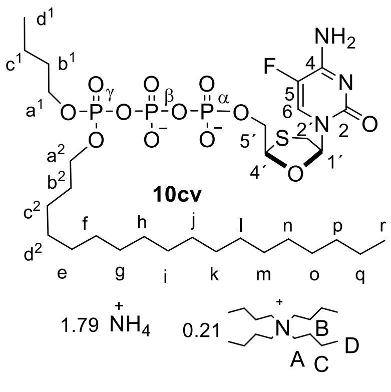

γ-(C4;C18)-FTCTP 10cv. According to general procedure 2 with 117 mg *H*-phosphonate **15ca** (0.3 mmol, 1.0 equiv.), 80 mg NCS (0.6 mmol, 2.0 equiv.), and 170 mg (n-$Bu_4N)_2$·FTCMP (0.21 mmol, 0.7 equiv.). Reaction time was 3 h. Yield: 94 mg (0.11 mmol, 51%) white solid. HPLC-UV analysis confirmed purity: > 97%. **^{1}H-NMR (600 MHz, CD_3OD):** δ [ppm] = 8.14 (d, $^{3}J_{HH}$= 6.6 Hz, 1H, H-6), 6.24 (dt, $^{3}J_{HH}$= 5.0 Hz, $^{3}J_{HH}$= 1.7 Hz, 1H, H-1´), 5.40 (t, $^{3}J_{HH}$= 4.8 Hz, 1H, H-4´), 5.21 (d, $^{3}J_{HH}$= 8.2 Hz, 2H, H-a^{1}), 4.42-4.28 (m, 2H, H-5´), 4.22-4.07 (m, 2H, H-a^{2}), 3.49 (dd, $^{3}J_{HH}$= 12.0 Hz, $^{3}J_{HH}$= 5.2 Hz, 1H, H-2´$_{a}$), 3.28-3.20 (m, 1.68H, H-A), 3.15 (dd, $^{3}J_{HH}$= 12.1 Hz, $^{3}J_{HH}$= 4.8 Hz, 1H, H-2´$_{b}$), 1.75-1.60 (m, 5.68H, H-b^{1}, H-b^{2}, H-B), 1.49-1.38 (m, 5.68H, H-c^{1}, H-c^{2}, H-C), 1.37-1.26 (m, 30H, H-d^{2}, H-e, H-f, H-j, H-h, H-i, H-j, H-k, H-l, H-m, H-n, H-o, H-p, H-q), 1.02 (t,

$^{3}J_{HH}$= 7.3 Hz, 3H, H-D), 0.95 (t, $^{3}J_{HH}$= 7.4 Hz, 3H, H-d^{1}), 0.89 (t, $^{3}J_{HH}$= 7.4 Hz, 3H, H-r). **^{13}C-NMR (151 MHz, CD$_3$OD):** δ [ppm] = 159.4 (d, $^{2}J_{CP}$= 15.0 Hz, C-4), 155.6 (C-2), 139.7, 136.5 (C-5), 127.3, 126.9 (C-6), 89.2 (C-1´), 85.6 (d, $^{3}J_{CP}$= 9.2 Hz, C-4´), 69.7 (d, $^{3}J_{CP}$= 6.2 Hz, C-a^{1}), 69.3 (d, $^{3}J_{CP}$= 6.2 Hz, C-a^{2}), 68.0 (d, $^{3}J_{CP}$= 5.7 Hz, C-5´), 59.5 (d, $^{3}J_{CP}$= 2.9 Hz, C-A), 37.9 (C-2´), 33.39, 33.29, 33.1, 31.35, 31.25, 30.78, 30.75, 30.72, 30.70, 30.5, 30.3, 23.7 (C-b^{1}, C-b^{2}, C-d^{2}, C-e, C-f, C-g, C-h, C-i, C-j, C-k, C-l, C-m, C-n, C-o, C-p, C-q), 26.6 (C-c^{2}), 24.8 (C-B), 20.7 (C-C), 19.7 (C-c^{1}), 14.5 (C-r), 14.0 (C-j^{1}), 14.0 (C-D). **^{31}P NMR (162 MHz, CD$_3$OD):** δ [ppm] = -11.95 (d, $^{2}J_{pp}$=17.6 Hz, P-α), -12.85 (d, $^{2}J_{pp}$=17.6 Hz, P-γ), -23.69 (t, $^{2}J_{pp}$= 17.6 Hz, P-β). **^{19}F-NMR (188 MHz, CD$_3$OD):** δ [ppm] = -167.6 -- -167.9. **HRMS (ESI$^-$, m/z):** calculated for $C_{30}H_{57}FN_3O_{12}SP_3$ [M-H]$^-$ 794.2787; found, 794.2506.

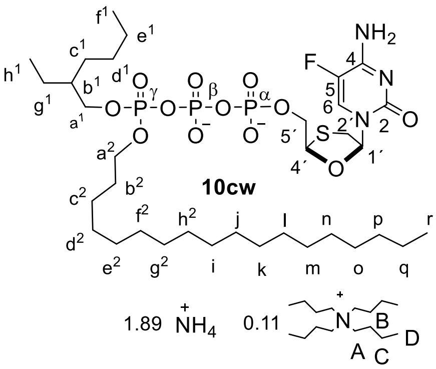

γ-(C8;C18)-FTCTP 10cw. According to general procedure 2 with 134 mg *H*-phosphonate **15cb** (0.3 mmol, 1.0 equiv.), 80 mg NCS (0.6 mmol, 2.0 equiv.), and 170 mg (n-Bu$_4$N)$_2$·FTCMP (0.21 mmol, 0.7 equiv.). Reaction time was 3 h. Yield: 114 mg (0.13 mmol, 60%) white solid. HPLC-UV analysis confirmed purity: > 97%. **^{1}H-NMR (600 MHz, CD$_3$OD):** δ [ppm] = 8.19 (d, $^{3}J_{HH}$= 6.6 Hz, 1H, H-6), 6.23 (dt, $^{3}J_{HH}$= 5.0 Hz, $^{3}J_{HH}$= 1.7 Hz, 1H, H-1´), 5.44 (dd, $^{3}J_{HH}$= 5.0 Hz, $^{3}J_{HH}$= 3.7 Hz, 1H, H-4´), 4.43-4.20 (m, 6H, H-5´, H-a^{1}, H-a^{2}), 3.49 (dd, $^{3}J_{HH}$= 12.0 Hz, $^{3}J_{HH}$= 5.3 Hz, 1H, H-2´$_a$), 3.27-3.21 (m, 0.88H, H-A), 3.15 (dd, $^{3}J_{HH}$= 11.9 Hz, $^{3}J_{HH}$= 4.8 Hz, 1H, H-2´$_b$), 1.76-1.65 (m, H-b^{2}, H-B), 1.62-1.56 (H-b^{1}), 1.45-1.25 (m, 38.88H, H-C, H-c^{1}, H-c^{2}, H-d^{1}, H-d^{2}, H-e^{1}, H-e^{2}, H-f^{2}, H-g^{1}, H-g^{2}, H-h^{2}, H-i, H-j, H-k, H-l, H-m, H-n, H-o, H-p, H-q), 1.03 (t, $^{3}J_{HH}$= 7.3 Hz, 1.32H), 0.94-0.88 (m, 9H, H-h^{1}, H-f^{1}, H-r). **^{13}C-NMR (151 MHz, CD$_3$OD):** δ [ppm] = 159.1 (d, $^{2}J_{CP}$= 15.4 Hz, C-4), 155.1 (C-2), 138.8, 137.2 (C-5), 127.5, 127.3 (C-6), 89.2 (C-1´), 85.8 (d, $^{3}J_{CP}$= 9.5 Hz, C-4´), 71.6 (dd, $^{3}J_{CP}$= 6.6 Hz, $^{3}J_{CP}$= 2.7 Hz, C-a^{1}), 69.8 (d, $^{3}J_{CP}$= 6.3 Hz, C-a^{2}), 68.1 (d, $^{3}J_{CP}$= 5.7 Hz, C-5´), 59.5 (t, $^{3}J_{CP}$= 2.9 Hz, C-A), 41.4 (d, $^{3}J_{CP}$= 7.5 Hz, C-b^{1}), 37.9 (C-2´), 33.1, 31.36, 31.32, 31.03, 31.02, 30.79, 30.75, 30.71, 30.70, 30.5, 30.3, 30.05, 30.03, 24.31, 24.29, 24.1, 23.7 (C-b^{2}, C-c^{1}, C-d^{1}, C-d^{2}, C-e^{1}, C-e^{2}, C-f^{2}, C-g^{1}, C-g^{2}, C-h^{2}, C-i, C-j, C-k, C-l, C-m, C-n, C-o, C-p, C-q), 26.7 (C-c^{2}), 24.8 (C-B), 20.7 (C-C), 14.46, 14.45 (C-f^{1}, C-r), 13.9 (C-D), 11.3 (d, $^{4}J_{CP}$= 1.6 Hz, C-h^{1}). **^{31}P NMR (162 MHz, CD$_3$OD):** δ [ppm] = -11.86 (d, $^{2}J_{pp}$=17.6 Hz, P-α), -12.67 (d, $^{2}J_{pp}$=14.8 Hz, P-γ), -23.52 (t, $^{2}J_{pp}$= 17.6 Hz, P-β). **^{19}F-NMR (188 MHz, CD$_3$OD):** δ [ppm] = -167.6 -- -167.9. **MALDI-MS (m/z):** calculated for $C_{34}H_{65}FN_3O_{12}SP_3$ [M-H]$^-$ 850.3413; found, 850.4928.

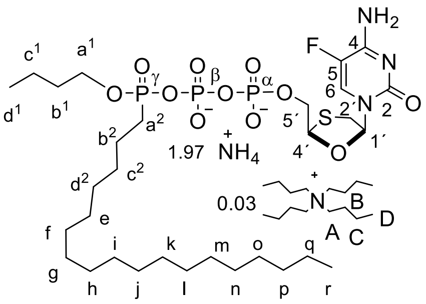

10cx

γ-(C4)-γ-C-(C18)-FTCTP 10cx. According to general procedure 2 with 112 mg *H*-phosphinate **15d** (0.3 mmol, 1.0 equiv.), 80 mg NCS (0.6 mmol, 2.0 equiv.), and 170 mg (n-Bu$_4$N)$_2$·FTCMP (0.21 mmol, 0.7 equiv.). Reaction time was 3 h. Yield: 83 mg (0.10 mmol, 48%) white solid. HPLC-UV analysis confirmed purity: > 99%. **^{1}H-NMR (600 MHz, CD$_3$OD):** δ [ppm] = 8.21 (d, $^{3}J_{HH}$= 6.6 Hz, 1H, H-6), 6.23 (dt, $^{3}J_{HH}$= 5.0 Hz, $^{3}J_{HH}$= 1.7 Hz, 1H, H-1´), 5.44 (dd, $^{3}J_{HH}$= 4.9 Hz,

$^3J_{HH}$= 3.7 Hz, 1H, H-4´), 4.43-4.20 (m, 4H, H-5´, H-a^1), 3.49 (dd, $^3J_{HH}$= 12.0 Hz, $^3J_{HH}$= 5.4 Hz, 1H, H-2´$_a$), 3.27-3.21 (m, 0.24H, H-A), 3.15 (dd, $^3J_{HH}$= 11.9 Hz, $^3J_{HH}$= 4.8 Hz, 1H, H-2´$_b$), 2.10-1.95 (m, 2H, H-a^2), 1.76-1.58 (m, 4.24H, H-b^1, H-b^2, H-B), 1.50-1.37 (m, 4.24H, H-c^1, H-c^2, H-C), 1.37-1.25 (m, 28H, H-d^2, H-e, H-f, H-g, H-h, H-i, H-j, H-k, H-l, H-m, H-n, H-o, H-p, H-q), 1.05 (t, $^3J_{HH}$= 7.4 Hz, 0.36H, H-D), 0.97-0.88 (m, 6H, H-d^1, H-r). **^{13}C-NMR (151 MHz, CD$_3$OD):** δ [ppm] = 157.3 (d, $^2J_{CP}$= 16.2 Hz, C-4), 153.1 (C-2), 137.3, 135.7 (C-5), 126.4, 126.2 (C-6), 87.8 (C-1´), 84.4 (d, $^3J_{CP}$= 9.1 Hz, C-4´), 66.7 (d, $^3J_{CP}$= 5.6 Hz, C-a^1), 66.5 (d, $^3J_{CP}$= 7.3 Hz, C-5´), 36.5 (C-2´), 32.15 (d, $^3J_{CP}$= 6.8 Hz, C-b^1), 32.13, 30.24, 30.13, 29.39, 29.36, 29.34, 29.18, 29.07, 28.88, 23.4 (C-b^2, C-c^1, C-d^2, C-e, C-f, C-g, C-h, C-i, C-j, C-k, C-l, C-m, C-n, C-o, C-p, C-q), 26.0, 25.0 (C-a^2), 21.95 (d, $^3J_{CP}$= 5.6 Hz, C-b^2), 18.5 (C-c^1), 13.0, 12.6 (C-d^1, C-r). **^{31}P NMR (162 MHz, CD$_3$OD):** δ [ppm] = 28.1 (d, $^2J_{pp}$=25.6 Hz, P-γ), -7.95 (d, $^2J_{pp}$=17.6 Hz, P-α), -19.72 (t, $^2J_{pp}$= 20.6 Hz, P-β). **^{19}F-NMR (188 MHz, CD$_3$OD):** δ [ppm] = -167.5 -- -167.9. **HRMS (ESI$^-$, m/z):** calculated for $C_{30}H_{57}FN_3O_{11}SP_3$ [M-H]$^-$ 778.2838; found, 778.2757.

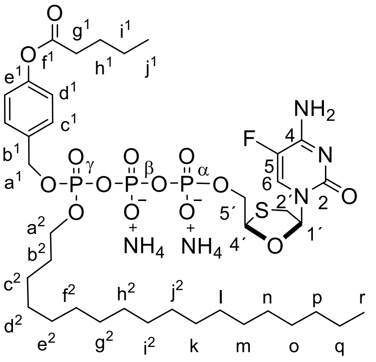

10cy

γ-(AB-C4;C18)-FTCTP 10cy. According to general procedure 2 with 157 mg *H*-phosphonate **15a** (0.3 mmol, 1.0 equiv.), 80 mg NCS (0.6 mmol, 2.0 equiv.), and 170 mg (n-Bu$_4$N)$_2$·FTCMP (0.21 mmol, 0.7 equiv.). Reaction time was 3 h. Yield: 148 mg (0.15 mmol, 73%) white solid. HPLC-UV analysis confirmed purity: > 96%. **^{1}H-NMR (600 MHz, CD$_3$OD):** δ [ppm] = 8.16 (d, $^3J_{HH}$= 6.5 Hz, 1H, H-6), 7.50-7.47 (m, 2H, H-c^1), 7.10-7.07 (m, 2H, H-d^1), 6.21 (dt, $^3J_{HH}$= 5.1 Hz, $^3J_{HH}$= 1.5 Hz, 1H, H-1´), 5.40 (t, $^3J_{HH}$= 4.2 Hz, 1H, H-4´), 5.21 (d, $^3J_{HH}$= 8.2 Hz, 2H, H-a^1), 4.42-4.28 (m, 2H, H-5´), 4.16-4.07 (m, 2H, H-a^2), 3.47 (dd, $^3J_{HH}$= 12.1 Hz, $^3J_{HH}$= 5.2 Hz, 1H, H-2´$_a$), 3.15 (dd, $^3J_{HH}$= 12.1 Hz, $^3J_{HH}$= 4.7 Hz, 1H, H-2´$_b$). 2.58 (t, $^3J_{HH}$= 7.4 Hz, 2H, H-g^1), 1.73 (quint, $^3J_{HH}$= 7.4 Hz, 2H, H-h^1), 1.62 (quint, $^3J_{HH}$= 6.8 Hz, 2H, H-b^2), 1.45 (sext, $^3J_{HH}$= 7.5 Hz, 2H, H-i^1), 1.35-1.26 (m, 30H, H-c^2, H-d^2, H-e^2, H-f^2, H-j^2, H-h^2, H-i^2, H-j^2, H-k, H-l, H-m, H-n, H-o, H-p, H-q), 0.98 (t, $^3J_{HH}$= 7.4 Hz, 3H, H-j^1), 0.89 (t, $^3J_{HH}$= 7.0 Hz, 3H, H-r). **^{13}C-NMR (151 MHz, CD$_3$OD):** δ [ppm] = 173.6 (C-f^1), 158.8 (d, $^2J_{CP}$= 15.5 Hz, C-4), 154.7 (C-2), 152.3 (C-e^1), 138.7, 137.1 (C-5), 135.1 (d, $^3J_{CP}$= 7.7 Hz, C-b^1), 130.4 (C-c^1), 127.6, 127.4 (C-6), 122.8 (C-d^1), 89.1 (C-1´), 85.8 (d, $^3J_{CP}$= 9.7 Hz, C-4´), 70.3 (d, $^3J_{CP}$= 5.5 Hz, C-a^1), 69.9 (d, $^3J_{CP}$= 6.5 Hz, C-a^2), 67.9 (d, $^3J_{CP}$= 5.3 Hz, C-5´), 37.9 (C-2´), 34.8 (C-g^1), 31.2 (d, $^3J_{CP}$= 6.8 Hz, C-b^2), 33.0, 30.80, 30.79, 30.74, 30.68, 30.5, 30.3, 23.7 (C-d^2, C-e^2, C-f^2, C-g^2, C-h^2, C-i^2, C-j^2, C-k, C-l, C-m, C-n, C-o, C-p, C-q), 28.0 (C-h^1), 26.5 (C-c^2), 23.2 (C-i^1), 14.5 (C-r), 14.1 (C-j^1). **^{31}P NMR (162 MHz, CD$_3$OD):** δ [ppm] = -11.85 (d, $^2J_{pp}$=17.3 Hz, P-α), -13.01 (d, $^2J_{pp}$=17.1 Hz, $^3J_{pp}$=5.8 Hz, P-γ), -23.52 (t, $^2J_{pp}$= 16.2 Hz, P-β). **^{19}F-NMR (188 MHz, CD$_3$OD):** δ [ppm] = -167.5 -- -167.8. **MALDI-MS (m/z):** calculated for $C_{38}H_{63}FN_3O_{14}SP_3$ [M-H]$^-$ 928.315; found, 928.275.

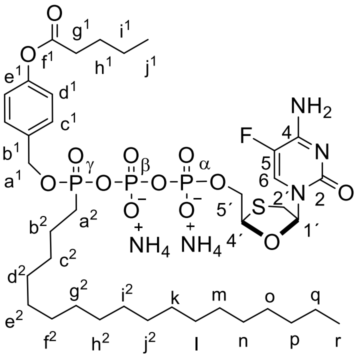

10cz

γ-(AB-C4)-γ-C-(C18)-FTCTP 10cz. According to general procedure 2 with 153 mg *H*-phosphinate **15b** (0.3 mmol, 1.0 equiv.), 80 mg NCS (0.6 mmol, 2.0 equiv.), and 170 mg (n-Bu$_4$N)$_2$·FTCMP (0.21 mmol, 0.7 equiv.). Reaction time was 3 h. Yield: 92 mg (0.10 mmol, 46%) white solid. HPLC-UV analysis confirmed purity: > 98%. **^{1}H-NMR (600 MHz, CD$_3$OD):** δ

[ppm] = 8.25 (d, $^3J_{HH}$= 6.5 Hz, 1H, H-6), 7.52-7.47 (m, 2H, H-c^1), 7.10-7.06 (m, 2H, H-d^1), 6.21 (dt, $^3J_{HH}$= 6.3 Hz, $^3J_{HH}$= 1.5 Hz, 1H, H-1´), 5.40 (q, $^3J_{HH}$= 4.1 Hz, 1H, H-4´), 5.26-5.16 (m, 2H, H-a^1), 4.44-4.30 (m, 2H, H-5´), 3.48 (dd, $^3J_{HH}$= 12.0 Hz, $^3J_{HH}$= 5.1 Hz, 1H, H-2´$_a$), 3.20-3.15 (m, 1H, H-2´$_b$). 2.58 (t, $^3J_{HH}$= 7.4 Hz, 2H, H-g^1), 2.10-1.96 (m, 2H, H-a^2), 1.71 (quint, $^3J_{HH}$= 7.4 Hz, 2H, H-h^1), 1.67-1.55 (m, 2H, H-b^2), 1.45 (sext, $^3J_{HH}$= 7.5 Hz, 2H, H-i^1), 1.40-1.24 (m, 30H, H-c^2, H-d^2, H-e^2, H-f^2, H-j^2, H-h^2, H-i^2, H-j^2, H-k, H-l, H-m, H-n, H-o, H-p, H-q), 0.98 (t, $^3J_{HH}$= 7.4 Hz, 3H, H-j^1), 0.90 (t, $^3J_{HH}$= 7.0 Hz, 3H, H-r). **^{13}C-NMR (151 MHz, CD$_3$OD):** δ [ppm] = 173.7 (C-f^1), 158.0 (d, $^2J_{CP}$= 16.9 Hz, C-4), 153.4 (C-2), 152.2 (C-e^1), 138.4, 136.8 (C-5), 135.5 (d, $^3J_{CP}$= 7.7 Hz, C-b^1), 130.4 (d, $^3J_{CP}$= 2.8 Hz, C-c^1), 128.1 (d, $^2J_{CP}$= 32.8 Hz, C-6), 122.8 (C-d^1), 89.1 (C-1´), 86.0 (dd, $^3J_{CP}$= 9.1 Hz, $^3J_{CP}$= 3.4 Hz, C-4´), 67.98, 67.93, 67.88 (C-a^1, C-5´), 38.0 (d, $^3J_{CP}$= 2.8 Hz, C-2´), 34.8 (C-g^1), 33.1, 31.6, 31.5, 30.80, 30.76, 30.74, 30.6, 30.5, 30.2, 23.7 (C-c^2, C-d^2, C-e^2, C-f^2, C-g^2, C-h^2, C-i^2, C-j^2, C-k, C-l, C-m, C-n, C-o, C-p, C-q), 28.1 (C-h^1), 27.5, 26.6 (C-a^2), 23.28, 23.25 (C-b^2, C-i^1), 14.5 (C-r), 14.1 (C-j^1). **^{31}P NMR (162 MHz, CD$_3$OD):** δ [ppm] = 24.55 (d, $^2J_{pp}$=23.6 Hz, P-γ), -11.9 (d, $^2J_{pp}$=20.6 Hz, P-α), -23.52 (dd, $^2J_{pp}$= 23.4 Hz, $^2J_{pp}$= 18.0 Hz, P-β).**^{19}F-NMR (188 MHz, CD$_3$OD):** δ [ppm] = -167.7- -167.9. **HRMS (ESI$^-$, m/z):** calculated for $C_{38}H_{63}FN_3O_{13}SP_3$ [M-H]$^-$ 912.3206; found, 912.2875.

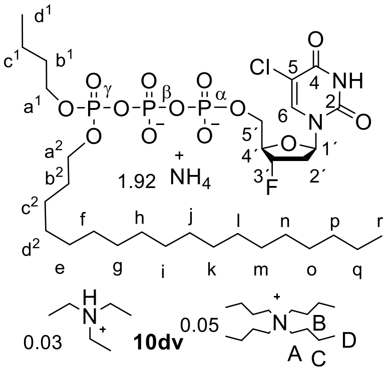

γ-(C4;C18)-Fdd(Cl)UTP 10dv. According to general procedure 2 with 117 mg *H*-phosphonate **15ca** (0.3 mmol, 1.0 equiv.), 80 mg NCS (0.6 mmol, 2.0 equiv.), and 174 mg (n-Bu$_4$N)$_2$·FddClUMP (0.21 mmol, 0.7 equiv.). Reaction time was 3 h. Yield: 98 mg (0.11 mmol, 54%) white solid. HPLC-UV analysis confirmed purity: > 97%. **^{1}H-NMR (600 MHz, CD$_3$OD):** δ [ppm] = 8.17 (s, 1H, H-6), 6.30 (dd, $^3J_{HH}$= 9.5 Hz, $^3J_{HH}$= 5.3 Hz, 1H, H-1´), 5.48 (dd, $^2J_{HH}$= 53.2 Hz, $^3J_{HH}$= 4.5 Hz, 1H, H-3´), 4.43-4.36 (m, 1H, H-4´), 4.33-4.27 (m, 1H, H-5´$_a$), 4.22-4.12 (m, 5H, H-5´$_b$, H-a^1, H-a^2), 3.27-3.21 (m, 0.4H, H-A), 3.21-3.17 (m, 0.18H, HN(CH$_2$CH$_3$)$_3^+$), 2.58-2.35 (m, 2H, H-2´), 1.74-1.63 (m, 4.4H, H-B, H-b^1, H-b^2), 1.46-1.37 (m, 4.4H, H-C, H-c^1, H-c^2), 1.34-1.26 (m, 28H, H-d^2, H-e, H-f, H-j, H-h, H-i, H-j, H-k, H-l, H-m, H-n, H-o, H-p, H-q), 1.02 (t, $^3J_{HH}$= 7.4 Hz, 1H, H-D), 0.98 (t, $^3J_{HH}$= 7.4 Hz, 3H, H-d^1), 0.89 (t, $^3J_{HH}$= 7.0 Hz, 3H, H-r). **^{13}C-NMR (151 MHz, CD$_3$OD):** δ [ppm] = 161.5 (C-4), 151.4 (C-2), 139.0 (C-6), 110.1 (C-5), 96.0 (d,$^1J_{CF}$= 175.2 Hz, C-3´), 86.8 (C-1´), 85.6 (dd, $^3J_{CP}$= 25.1 Hz, $^3J_{CP}$= 9.2 Hz, C-4´), 69.7, 69.4 (2 x d, $^3J_{CP}$= 6.3 Hz, $^3J_{CP}$= 6.2 Hz, C-a^1, C-a^2), 66.5 (dd, $^2J_{CP}$= 11.9 Hz, $^3J_{CP}$= 5.4 Hz, C-5´), 59.5 (C-A), 47.5 (HN(CH$_2$CH$_3$)$_3^+$), 39.0 (d, $^2J_{CF}$= 20.3 Hz, C-2´), 33.3 (d, $^3J_{CP}$= 7.3 Hz, C-b^1), 31.3 (d, $^3J_{CP}$= 7.3 Hz, C-b^2), 33.1, 30.78, 30.75, 30.71, 30.69, 30.5, 30.3, 23.7 (C-d^2, C-e^2, C-f^2, C-g^2, C-h^2, C-i^2, C-j^2, C-k, C-l, C-m, C-n, C-o, C-p, C-q), 26.6 (C-c^2), 24.8 (C-B), 20.7 (C-C), 19.8 (C-c^1), 14.4 (C-r), 13.99 (C-d^1), 13.94 (C-D), 9.1 (HN(CH$_2$CH$_3$)$_3^+$). **^{31}P NMR (243 MHz, CD$_3$OD):** δ [ppm] = -11.97 (d, $^2J_{pp}$=17.8 Hz, P-α), -12.73 (d, $^2J_{pp}$=14.5 Hz, P-γ), -23.55 (t, $^2J_{pp}$= 17.5 Hz, P-β). **^{19}F-NMR (188 MHz, CD$_3$OD):** δ [ppm] = -175.6 -- -176.4. **HRMS (ESI$^-$, m/z):** calculated for $C_{31}H_{57}FClN_2O_{13}P_3$ [M-H]$^-$ 811.2673; found, 811.2290.

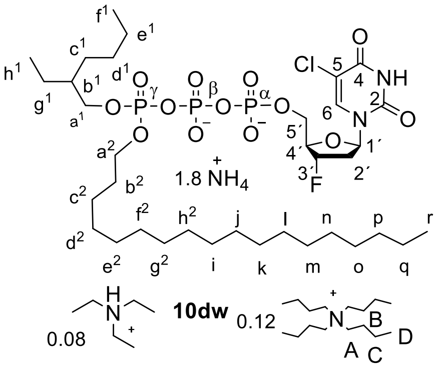

γ-(C8;C18)-Fdd(Cl)UTP 10dw. According to general procedure 2 with 134 mg *H*-phosphonate **15cb** (0.3 mmol, 1.0 equiv.), 80 mg NCS (0.6 mmol, 2.0 equiv.), and 174 mg $(n\text{-}Bu_4N)_2$·FddClUMP (0.21 mmol, 0.7 equiv.). Reaction time was 3 h. Yield: 96 mg (0.10 mmol, 49%) white solid. HPLC-UV analysis confirmed purity: > 95%. **^{1}H-NMR (600 MHz, CD_3OD):** δ [ppm] = 8.18 (s, 1H, H-6), 6.30 (dd, $^{3}J_{HH}$= 9.5 Hz, $^{3}J_{HH}$= 5.3 Hz, 1H, H-1´), 5.54 (dd, $^{2}J_{HH}$= 53.1 Hz, $^{3}J_{HH}$= 4.4 Hz, 1H, H-3´), 4.43-4.35 (m, 1H, H-4´), 4.34-4.28 (m, 1H, H-5´$_{a}$), 4.22-4.03 (m, 5H, H-5´$_{b}$, H-a^{1}, H-a^{2}), 3.27-3.21 (m, 0.96H, H-A), 3.21-3.17 (m, 0.48H, $HN(CH_2CH_3)_3^{+}$), 2.56-2.35 (m, 2H, H-2´), 1.74-1.63 (m, 2.96H, H-B, H-b^{2}), 1.62-1.55 (m, 1H, H-b^{1}), 1.46-1.25 (m, 39.44H, H-C, $HN(CH_2CH_3)_3^{+}$, H-c^{1}, H-c^{2}, H-d^{1}, H-d^{2}, H-e^{1}, H-e^{2}, H-f^{2}, H-g^{1}, H-g^{2}, H-h^{2}, H-i, H-j, H-k, H-l, H-m, H-n, H-o, H-p, H-q), 1.03 (t, $^{3}J_{HH}$= 7.4 Hz, 1.44H, H-D), 0.96-0.88 (m, 9H, H-h^{1}, H-f^{1}, H-r). **^{13}C-NMR (151 MHz, CD_3OD):** δ [ppm] = 161.5 (C-4), 151.4 (C-2), 139.0 (C-6), 110.1 (C-5), 96.0 (d,$^{1}J_{CF}$= 175.1 Hz, C-3´), 86.8 (C-1´), 85.6 (dd, $^{3}J_{CP}$= 25.4 Hz, $^{3}J_{CP}$= 9.5 Hz, C-4´), 71.6 (dd, $^{3}J_{CP}$= 6.3 Hz, $^{3}J_{CP}$= 2.8 Hz, C-a^{1}), 69.7 (d, $^{3}J_{CP}$= 6.2 Hz, C-a^{2}), 66.5 (dd, $^{2}J_{CP}$= 12.9 Hz, $^{3}J_{CP}$= 6.4 Hz, C-5´), 59.5 (t, $^{3}J_{CP}$= 2.8 Hz, C-A), 47.5 ($HN(CH_2CH_3)_3^{+}$), 41.4 (d, $^{3}J_{CP}$= 7.9 Hz, C-b^{1}), 39.0 (d, $^{2}J_{CF}$= 21.0 Hz, C-2´), 31.3 (d, $^{3}J_{CP}$= 7.3 Hz, C-b^{2}), 33.1, 31.0, 30.78, 30.76, 30.70, 30.5, 30.3, 30.06, 30.04, 24.32, 24.29, 24.1, 23.7 (C-c^{1}, C-d^{1}, C-d^{2}, C-e^{1}, C-e^{2}, C-f^{2}, C-g^{1}, C-g^{2}, C-h^{2}, C-i, C-j, C-k, C-l, C-m, C-n, C-o, C-p, C-q), 26.7 (C-c^{2}), 24.8 (C-B), 20.7 (C-C), 14.4 (C-f^{1}, C-r), 14.0 (C-d^{1}), 13.9 (C-D), 11.4 (C-h^{1}), 9.2 ($HN(CH_2CH_3)_3^{+}$). **^{31}P NMR (243 MHz, CD_3OD):** δ [ppm] = -12.02 (d, $^{2}J_{pp}$=17.7 Hz, P-α), -12.62 (d, $^{2}J_{pp}$=14.8 Hz, P-γ), -23.63 (t, $^{2}J_{pp}$= 17.5 Hz, P-β). **^{19}F-NMR (188 MHz, CD_3OD):** δ [ppm] = -175.4 -- -176.4. **MALDI-MS (m/z):** calculated for $C_{35}H_{65}FClN_2O_{13}P_3$ $[M\text{-}H]^{-}$ 867.3299; found, 867.3542.

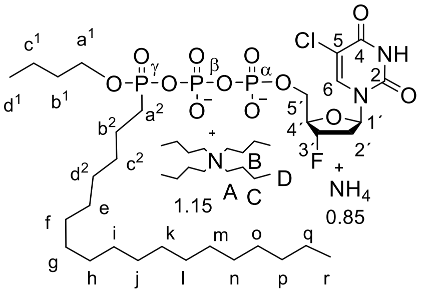

10dx

γ-(C4)-γ-C-(C18)-Fdd(Cl)UTP 10dx. According to general procedure 2 with 112 mg *H*-phosphinate **15d** (0.3 mmol, 1.0 equiv.), 80 mg NCS (0.6 mmol, 2.0 equiv.), and 174 mg $(n\text{-}Bu_4N)_2$·FddClUMP (0.21 mmol, 0.7 equiv.). Reaction time was 3 h. Yield: 128 mg (0.12 mmol, 56%) white solid. HPLC-UV analysis confirmed purity: > 97%. **^{1}H-NMR (600 MHz, CD_3OD):** δ [ppm] = 8.19 (d, $^{4}J_{HH}$= 0.8 Hz, 1H, H-6), 6.31 (dd, $^{3}J_{HH}$= 9.2 Hz, $^{3}J_{HH}$= 5.5 Hz, 1H, H-1´), 5.56 (dd, $^{2}J_{HH}$= 53.0 Hz, $^{3}J_{HH}$= 3.8 Hz, 1H, H-3´), 4.43-4.35 (m, 1H, H-4´), 4.35-4.29 (m, 1H, H-5´$_{a}$), 4.24-4.12 (m, 3H, H-5´$_{b}$, H-a^{1}), 3.28-3.20 (m, 9.2H, H-A), 2.55-2.36 (m, 2H, H-2´), 2.08-1.98 (m, 2H, H-a^{2}), 1.72-1.60 (m, 13.2H, H-B, H-b^{1}, H-b^{2}), 1.48-1.37 (m, 13.2H, H-C, H-c^{1}, H-c^{2}), 1.34-1.26 (m, 28H, H-d^{2}, H-e, H-f, H-j, H-h, H-i, H-j, H-k, H-l, H-m, H-n, H-o, H-p, H-q), 1.03 (t, $^{3}J_{HH}$= 7.4 Hz, 13.8H, H-D), 0.95 (t, $^{3}J_{HH}$= 7.4 Hz, 3H, H-d^{1}), 0.90 (t, $^{3}J_{HH}$= 7.0 Hz, 3H, H-r). **^{13}C-NMR (151 MHz, CD_3OD):** δ [ppm] = 161.5 (C-4), 151.4 (C-2), 139.1 (C-6), 110.1 (C-5), 96.7 (d,$^{1}J_{CF}$= 175.2 Hz, C-3´), 86.7 (C-1´), 85.7 (dd, $^{3}J_{CP}$= 25.5 Hz, $^{3}J_{CP}$= 9.6 Hz, C-4´), 66.8 (d, $^{3}J_{CP}$= 7.4 Hz, C-a^{1}), 66.4 (dd, $^{2}J_{CP}$= 11.9 Hz, $^{3}J_{CP}$= 5.1 Hz, C-5´), 59.5 (t, $^{3}J_{CP}$= 6.8 Hz, C-A), 39.0 (d, $^{2}J_{CF}$= 20.5 Hz, C-2´), 33.5 (d, $^{3}J_{CP}$= 6.8 Hz, C-b^{1}), 31.5 (d, $^{2}J_{CP}$= 17.3 Hz, C-b^{2}), 33.1, 30.78, 30.75, 30.72, 30.6, 30.5, 30.3, 23.7 (C-c^{2}, C-d^{2}, C-e, C-f, C-g, C-h, C-i, C-j, C-k, C-l, C-m, C-n, C-o, C-p, C-q), 27.3, 26.4 (C-a^{2}), 24.8 (C-B), 20.7 (t, $^{4}J_{CP}$= 1.4 Hz, C-C), 23.3 (d, $^{3}J_{CP}$= 5.2 Hz, C-b^{2}), 19.9 (C-c^{1}), 14.4 (C-r), 14.0 (C-d^{1}), 13.9

(C-D). **^{31}P NMR (243 MHz, CD_3OD):** δ [ppm] = 24.0 (d, $^{2}J_{pp}$=23.5 Hz, P-γ), -12.14 (d, $^{2}J_{pp}$=20.4 Hz, P-α), -23.92 (t, $^{2}J_{pp}$= 20.7 Hz, P-β). **^{19}F-NMR (188 MHz, CD_3OD):** δ [ppm] = -175.4 -- -176.6. **HRMS (ESI$^-$, m/z):** calculated for $C_{31}H_{57}FClN_2O_{12}P_3$ [M-H]$^-$ 795.2724; found, 795.2163.

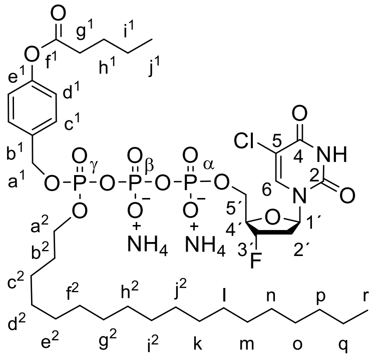

10dy

γ-(AB-C4;C18)-Fdd(Cl)UTP 10dy. According to general procedure 2 with 157 mg *H*-phosphonate **15a** (0.3 mmol, 1.0 equiv.), 80 mg NCS (0.6 mmol, 2.0 equiv.), and 174 mg (n-$Bu_4N)_2$·FddClUMP (0.21 mmol, 0.7 equiv.). Reaction time was 3 h. Yield: 165 mg (0.17 mmol, 80%) white solid. HPLC-UV analysis confirmed purity: > 97%. **^{1}H-NMR (600 MHz, CD_3OD):** δ [ppm] = 8.13 (s, 1H, H-6), 7.52-7.47 (m, 2H, H-c^1), 7.11-7.07 (m, 2H, H-d^1), 6.29 (dd, $^{3}J_{HH}$= 9.5 Hz, $^{3}J_{HH}$= 5.3 Hz, 1H, H-1´), 5.48 (dd, $^{2}J_{HH}$= 53.1 Hz, $^{3}J_{HH}$= 2.9 Hz, 1H, H-3´), 5.23-5.17 (m, 2H, H-a^1), 4.45-4.33 (m, 1H, H-4´), 4.32-4.24 (m, 1H, H-5´$_a$), 4.19-4.13 (m, 1H, H-5´$_b$), 4.13-4.08 (m, 2H, H-a^2), 2.57 (t, $^{3}J_{HH}$= 7.4 Hz, 2H, H-g^1), 2.55-2.30 (m, 2H, H-2´), 1.73 (quint, $^{3}J_{HH}$= 7.4 Hz, 2H, H-h^1), 1.62 (quint, $^{3}J_{HH}$= 6.7 Hz, 2H, H-b^2), 1.44 (sext, $^{3}J_{HH}$= 7.5 Hz, 2H, H-i^1), 1.34-1.26 (m, 30H, H-c^2, H-d^2, H-e^2, H-f^2, H-j^2, H-h^2, H-i^2, H-j^2, H-k, H-l, H-m, H-n, H-o, H-p, H-q), 0.98 (t, $^{3}J_{HH}$= 7.4 Hz, 3H, H-j^1), 0.89 (t, $^{3}J_{HH}$= 7.0 Hz, 3H, H-r). **^{13}C-NMR (151 MHz, CD_3OD):** δ [ppm] = 173.6 (C-f^1), 161.4 (C-4), 152.3 (C-e^1), 151.3 (C-2), 138.9 (C-6), 135.0 (d, $^{3}J_{CP}$= 7.7 Hz, C-b^1), 130.3 (d, $^{3}J_{CP}$= 3.3 Hz, C-c^1), 122.8 (C-d^1), 110.0 (C-5), 96.0 (d,$^{1}J_{CF}$= 175.7 Hz, C-3´), 86.8 (C-1´), 85.6 (dd, $^{3}J_{CP}$= 25.9 Hz, $^{3}J_{CP}$= 9.9 Hz, C-4´), 70.2 (dd, $^{3}J_{CP}$= 5.6 Hz, $^{3}J_{CP}$= 3.3 Hz, C-a^1), 69.8 (d, $^{3}J_{CP}$= 6.6 Hz, C-a^2), 66.5 (dd, $^{2}J_{CP}$= 11.9 Hz, $^{3}J_{CP}$= 5.4 Hz, C-5´), 38.9 (d, $^{2}J_{CF}$= 21.0 Hz, C-2´), 34.7 (C-g^1), 31.2 (d, $^{3}J_{CP}$= 7.6 Hz, C-b^2), 33.1, 30.80, 30.74, 30.72, 30.66, 30.5, 30.3, 23.7 (C-d^2, C-e^2, C-f^2, C-g^2, C-h^2, C-i^2, C-j^2, C-k, C-l, C-m, C-n, C-o, C-p, C-q), 28.0 (C-h^1), 26.5 (C-c^2), 23.2 (C-i^1), 14.5 (C-r), 14.1 (C-j^1). **^{31}P NMR (243 MHz, CD_3OD):** δ [ppm] = -11.97 (d, $^{2}J_{pp}$=17.4 Hz, P-α), -12.91 (dd, $^{2}J_{pp}$=17.6 Hz, $^{3}J_{pp}$=5.8 Hz, P-γ), -23.53 (t, $^{2}J_{pp}$= 16.5 Hz, P-β). **^{19}F-NMR (188 MHz, CD_3OD):** δ [ppm] = -175.3 -- -176.5. **MALDI-MS (m/z):** calculated for $C_{39}H_{63}FClN_2O_{15}P_3$ [M-H]$^-$ 945.304; found, 945.253.

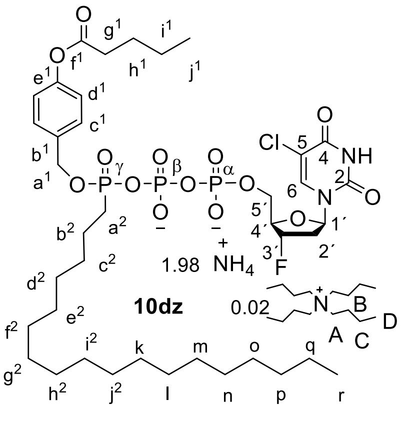

γ-(AB-C4)-γ-C-(C18)-Fdd(Cl)UTP 10dz. According to general procedure 2 with 153 mg *H*-phosphinate **15b** (0.3 mmol, 1.0 equiv.), 80 mg NCS (0.6 mmol, 2.0 equiv.), and 174 mg (n-$Bu_4N)_2$·FddClUMP (0.21 mmol, 0.7 equiv.). Reaction time was 3 h. Yield: 92 mg (0.09 mmol, 45%) white solid. HPLC-UV analysis confirmed purity: > 96%. **^{1}H NMR (600 MHz, CD_3OD):** δ [ppm] = 8.15 (d, $^{3}J_{HH}$= 2.1 Hz, 1H, H-6), 7.52-7.47 (m, 2H, H-c^1), 7.12-7.05 (m, 2H, H-d^1), 6.32-6.27 (m, 1H, H-1´), 5.54 (dt, $^{2}J_{HH}$= 53.2 Hz, $^{3}J_{HH}$= 4.1 Hz, 1H, H-3´), 5.27-5.17 (m, 2H, H-a^1), 4.42-4.12 (m, 3H, H-4´, H-5´), 3.26-3.20 (m, 0.16H, H-A), 2.58 (t, $^{3}J_{HH}$= 7.4 Hz, 2H, H-g^1), 2.55-2.36 (m, 1H, H-2´), 2.06-1.98 (m, 2H, H-a^2), 1.71 (quint, $^{3}J_{HH}$= 7.5 Hz, 2H, H-h^1), 1.67-1.56 (m, 2.16H, H-B, H-b^2), 1.46 (sext, $^{3}J_{HH}$= 7.5 Hz, 2H, H-i^1), 1.40-1.24 (m, 30.16H, H-C, H-c^2, H-d^2, H-e^2, H-f^2, H-g^2, H-h^2, H-i^2, H-j^2, H-k, H-l, H-m, H-n, H-o, H-p, H-q), 1.02 (t, $^{3}J_{HH}$= 7.4 Hz, 0.24H, H-D), 0.98 (t, $^{3}J_{HH}$= 7.4 Hz, 3H, H-j^1), 0.89 (t, $^{3}J_{HH}$= 7.0 Hz, 3H, H-r). **^{13}C-NMR (101 MHz, CD_3OD):** δ [ppm] = 173.7 (C-f^1), 161.5 (C-4), 152.2 (C-e^1), 151.3 (C-2), 139.0 (C-6), 135.5 (dd, $^{3}J_{CP}$= 7.2 Hz, $^{4}J_{CP}$= 1.4 Hz, C-b^1), 130.4 (d, $^{3}J_{CP}$=

3.3 Hz, C-c[1]), 122.8 (C-d[1]), 110.1 (C-5), 97.2 (dd, $^{1}J_{CF}$= 176.1 Hz, $^{2}J_{CF}$= 10.4 Hz, C-3´), 86.8 (C-1´), 85.5 (C-4´), 67.9 (dd, $^{3}J_{CP}$= 6.4 Hz, $^{4}J_{CP}$= 1.3 Hz, C-a[1]), 66.63, 66.55, 66.47 (C-5´), 59.5 (C-A), 39.1 (d, $^{2}J_{CF}$= 20.6 Hz, C-2´), 34.8 (C-g[1]), 33.1, 31.6, 31.4, 30.80, 30.76, 30.72, 30.55, 30.47, 30.2, 23.7 (C-d[2], C-e[2], C-f[2], C-g[2], C-h[2], C-i[2], C-j[2], C-k, C-l, C-m, C-n, C-o, C-p, C-q), 28.1 (C-h[1]), 28.0, 26.1 (C-a[2]), 24.8 (C-B), 23.3, 23.2 (C-b[2], C-i[1]), 20.7 (C-C), 14.5 (C-r), 14.10 (C-j[1]), 13.95 (C-D). **^{31}P NMR (162 MHz, CD_3OD):** δ [ppm] = 24.55 (d, $^{2}J_{pp}$=23.5 Hz, P-γ), -11.85 (d, $^{2}J_{pp}$=16.8 Hz, P-α), -23.40 (t, $^{2}J_{pp}$= 20.8 Hz, P-β). **^{19}F-NMR (188 MHz, CD_3OD):** δ [ppm] = -175.6 -- -176.2. **MALDI-MS (m/z):** calculated for $C_{39}H_{63}FClN_2O_{14}P_3$ [M-H]$^-$ 929.309; found, 929.270.

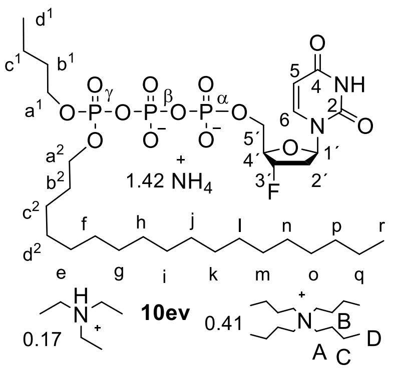

γ-(C4;C18)-FddUTP 10ev. According to general procedure 1 with 117 mg *H*-phosphonate **15ca** (0.3 mmol, 1.0 equiv.), 40 mg NCS (0.3 mmol, 1.0 equiv.), and 233 mg (*n*-$Bu_4N)_3$·FddUDP (0.21 mmol, 0.7 equiv.). Reaction time was 3 h. Yield: 116 mg (0.13 mmol, 60%) white solid. HPLC-UV analysis confirmed purity: > 97%. **^{1}H-NMR (600 MHz, CD_3OD):** δ [ppm] = 8.02 (d, $^{3}J_{HH}$= 8.1 Hz, 1H, H-6), 6.36 (dd, $^{3}J_{HH}$= 9.5 Hz, $^{3}J_{HH}$= 5.4 Hz, 1H, H-1´), 5.83 (d, $^{3}J_{HH}$= 8.2 Hz, 1H, H-5), 5.52 (dd, $^{2}J_{HH}$= 53.3 Hz, $^{3}J_{HH}$= 4.4 Hz, 1H, H-3´), 4.43-4.35 (m, 1H, H-4´), 4.33-4.27 (m, 1H, H-5´$_a$), 4.22-4.12 (m, 5H, H-5´$_b$, H-a[1], H-a[2]), 3.27-3.21 (m, 3.3H, H-A), 3.21-3.17 (m, 1.02H, $HN(CH_2CH_3)_3^+$), 2.58-2.32 (m, 2H, H-2´), 1.74-1.63 (m, 7.3H, H-B, H-b[1], H-b[2]), 1.46-1.37 (m, 7.3H, H-C, H-c[1], H-c[2]), 1.34-1.26 (m, 29.5H, $HN(CH_2CH_3)_3^+$, H-d[2], H-e, H-f, H-j, H-h, H-i, H-j, H-k, H-l, H-m, H-n, H-o, H-p, H-q), 1.03 (t, $^{3}J_{HH}$= 7.4 Hz, 4.95H, H-D), 0.95 (t, $^{3}J_{HH}$= 7.4 Hz, 3H, H-d[1]), 0.90 (t, $^{3}J_{HH}$= 7.0 Hz, 3H, H-r). **^{13}C-NMR (151 MHz, CD_3OD):** δ [ppm] = 166.1 (C-4), 152.3 (C-2), 142.4 (C-6), 103.6 (C-5), 96.3 (d,$^{1}J_{CF}$= 175.2 Hz, C-3´), 86.3 (C-1´), 85.6 (dd, $^{3}J_{CP}$= 25.3 Hz, $^{3}J_{CP}$= 9.6 Hz, C-4´), 69.7, 69.3 (2 x d, $^{3}J_{CP}$= 6.2 Hz, $^{3}J_{CP}$= 5.7 Hz, C-a[1], C-a[2]), 66.7 (dd, $^{2}J_{CP}$= 11.9 Hz, $^{3}J_{CP}$= 5.7 Hz, C-5´), 59.5 (t, $^{3}J_{CP}$= 2.6 Hz, C-A), 47.5, 43.1 ($HN(CH_2CH_3)_3^+$), 39.1 (d, $^{2}J_{CF}$= 20.5 Hz, C-2´), 33.3 (d, $^{3}J_{CP}$= 7.4 Hz, C-b[1]), 31.3 (d, $^{3}J_{CP}$= 7.3 Hz, C-b[2]), 33.1, 30.78, 30.75, 30.71, 30.69, 30.5, 30.3, 23.7 (C-d[2], C-e[2], C-f[2], C-g[2], C-h[2], C-i[2], C-j[2], C-k, C-l, C-m, C-n, C-o, C-p, C-q), 26.6 (C-c[2]), 24.8 (C-B), 20.7 (t, $^{4}J_{CP}$= 1.4 Hz, C-C), 19.8 (C-c[1]), 14.4 (C-r), 13.99 (C-d[1]), 13.94 (C-D), 11.6, 9.1 ($HN(CH_2CH_3)_3^+$). **^{31}P NMR (243 MHz, CD_3OD):** δ [ppm] = -11.96 (d, $^{2}J_{pp}$=17.7 Hz, P-α), -12.77 (d, $^{2}J_{pp}$=17.6 Hz, P-γ), -23.77 (t, $^{2}J_{pp}$= 17.7 Hz, P-β). **^{19}F-NMR (188 MHz, CD_3OD):** δ [ppm] = -175.2 -- -176.6. **HRMS (ESI$^-$, m/z):** calculated for $C_{31}H_{58}FN_2O_{13}P_3$ [M-H]$^-$ 777.3063; found, 777.3047.

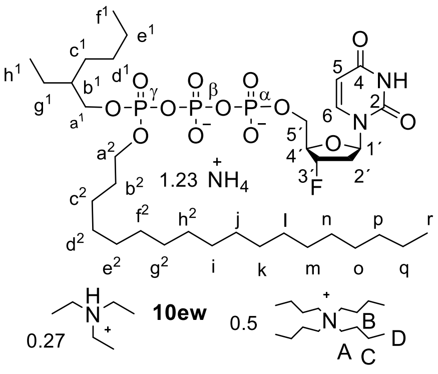

γ-(C8;C18)-FddUTP 10ew. According to general procedure 1 with 134 mg *H*-phosphonate **15cb** (0.3 mmol, 1.0 equiv.), 40 mg NCS (0.3 mmol, 1.0 equiv.), and 233 mg (*n*-$Bu_4N)_3$·FddUDP (0.21 mmol, 0.7 equiv.). Reaction time was 3 h. Yield: 101 mg (0.10 mmol, 48%) white solid. HPLC-UV analysis confirmed purity: > 97%. **^{1}H-NMR (600 MHz, CD_3OD):** δ [ppm] = 8.02 (d, $^{3}J_{HH}$= 8.1 Hz, 1H, H-6), 6.36 (dd, $^{3}J_{HH}$= 9.5 Hz, $^{3}J_{HH}$= 5.3 Hz, 1H, H-1´), 5.83 (d, $^{3}J_{HH}$= 8.2 Hz, 1H, H-5), 5.52 (dd, $^{2}J_{HH}$= 53.0 Hz, $^{3}J_{HH}$= 4.4 Hz, 1H, H-3´), 4.43-4.35 (m, 1H, H-4´), 4.34-4.28 (m, 1H, H-5´$_a$), 4.23-4.03 (m, 5H, H-5´$_b$, H-a[1], H-a[2]), 3.27-3.21 (m, 4H, H-A), 3.21-3.17 (m, 1.62H, $HN(CH_2CH_3)_3^+$), 2.55-2.32 (m, 2H, H-2´), 1.76-

1.66 (m, 6H, H-B, H-b^2), 1.65-1.55 (m, 1H, H-b^1), 1.46-1.25 (m, 43.62H, H-C, HN(CH$_2$CH$_3$)$_3^+$, H-c^1, H-c^2, H-d^1, H-d^2, H-e^1, H-e^2, H-f^2, H-g^1, H-g^2, H-h^2, H-i, H-j, H-k, H-l, H-m, H-n, H-o, H-p, H-q), 1.03 (t, $^3J_{HH}$= 7.4 Hz, 1.44H, H-D), 0.95-0.88 (m, 9H, H-h^1, H-f^1, H-r). **^{13}C-NMR (151 MHz, CD$_3$OD):** δ [ppm] = 166.1 (C-4), 152.3 (C-2), 142.4 (C-6), 103.6 (C-5), 96.3 (d,$^1J_{CF}$= 175.1 Hz, C-3´), 86.2 (C-1´), 85.5 (dd, $^3J_{CP}$= 25.4 Hz, $^3J_{CP}$= 9.6 Hz, C-4´), 71.6, 69.7 (2 x d, $^3J_{CP}$= 8.2 Hz, $^3J_{CP}$= 6.7 Hz, C-a^1, C-a^2), 66.8 (dd, $^2J_{CP}$= 11.6 Hz, $^3J_{CP}$= 5.6 Hz, C-5´), 59.5 (t, $^3J_{CP}$= 2.8 Hz, C-A), 47.5, 43.1 (HN(CH$_2$CH$_3$)$_3^+$), 41.4 (d, $^3J_{CP}$= 7.4 Hz, C-b^1), 39.1 (d, $^2J_{CF}$= 20.7 Hz, C-2´), 31.3 (d, $^3J_{CP}$= 6.9 Hz, C-b^2), 33.1, 31.03, 31.02, 30.78, 30.75, 30.70, 30.5, 30.2, 30.06, 30.03, 24.32, 24.29, 24.1, 23.7 (C-c^1, C-d^1, C-d^2, C-e^1, C-e^2, C-f^2, C-g^1, C-g^2, C-h^2, C-i, C-j, C-k, C-l, C-m, C-n, C-o, C-p, C-q), 26.7 (C-c^2), 24.8 (C-B), 20.7 (t, $^4J_{CP}$= 1.4 Hz, C-C), 14.4 (C-f^1, C-r), 13.99 (C-d^1), 13.94 (C-D), 11.3 (d, $^3J_{CP}$= 2.2 Hz,), 9.1 (HN(CH$_2$CH$_3$)$_3^+$). **^{31}P NMR (243 MHz, CD$_3$OD):** δ [ppm] = -12.05 (d, $^2J_{pp}$=18.0 Hz, P-α), -12.65 (d, $^2J_{pp}$=15.8 Hz, P-γ), -23.87 (t, $^2J_{pp}$= 17.3 Hz, P-β). **^{19}F-NMR (188 MHz, CD$_3$OD):** δ [ppm] = -175.4 -- -176.2. **MALDI-MS (m/z):** calculated for $C_{35}H_{66}FN_2O_{13}P_3$ [M-H]$^-$ 833.3689; found, 833.4318.

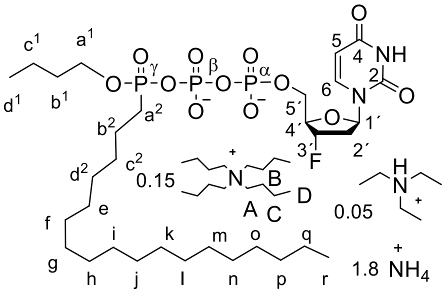

10ex

γ-(C4)-γ-C-(C18)-FddUTP 10ex. According to general procedure 1 with 112 mg *H*-phosphinate **15d** (0.3 mmol, 1.0 equiv.), 40 mg NCS (0.3 mmol, 1.0 equiv.), and 233 mg (*n*-Bu$_4$N)$_3$·FddUDP (0.21 mmol, 0.7 equiv.). Reaction time was 3 h. Yield: 96 mg (0.12 mmol, 55%) white solid. HPLC-UV analysis confirmed purity: > 97%. **^{1}H-NMR (600 MHz, CD$_3$OD):** δ [ppm] = 8.02 (d, $^4J_{HH}$= 8.1 Hz, 1H, H-6), 6.36 (dd, $^3J_{HH}$= 9.5 Hz, $^3J_{HH}$= 5.5 Hz, 1H, H-1´), 5.83 (d, $^3J_{HH}$= 8.2 Hz, 1H, H-5), 5.50 (dd, $^2J_{HH}$= 53.4 Hz, $^3J_{HH}$= 3.8 Hz, 1H, H-3´), 4.43-4.35 (m, 1H, H-4´), 4.32-4.25 (m, 1H, H-5´$_a$), 4.24-4.10 (m, 3H, H-5´$_b$, H-a^1), 3.28-3.21 (m, 1.2H, H-A), 3.20-3.17 (m, 0.24H, HN(CH$_2$CH$_3$)$_3^+$), 2.55-2.30 (m, 2H, H-2´), 2.06-1.96 (m, 2H, H-a^2), 1.72-1.60 (m, 5.2H, H-B, H-b^1, H-b^2), 1.48-1.37 (m, 5.2H, H-C, H-c^1, H-c^2), 1.34-1.26 (m, 28.36H, HN(CH$_2$CH$_3$)$_3^+$, H-d^2, H-e, H-f, H-j, H-h, H-i, H-j, H-k, H-l, H-m, H-n, H-o, H-p, H-q), 1.03 (t, $^3J_{HH}$= 7.4 Hz, 1.8H, H-D), 0.95 (t, $^3J_{HH}$= 7.4 Hz, 3H, H-d^1), 0.90 (t, $^3J_{HH}$= 7.0 Hz, 3H, H-r). **^{13}C-NMR (151 MHz, CD$_3$OD):** δ [ppm]= 166.1 (C-4), 152.3 (C-2), 142.4 (C-6), 103.6 (C-5), 96.7 (d,$^1J_{CF}$= 175.1 Hz, C-3´), 86.2 (C-1´), 85.3 (C-4´), 66.9 (d, $^3J_{CP}$= 7.3 Hz, C-a^1), 66.77, 66.69, 66.62 (C-5´), 59.5 (t, $^3J_{CP}$= 2.8 Hz, C-A), 47.5, 43.1 (HN(CH$_2$CH$_3$)$_3^+$), 39.1 (d, $^2J_{CF}$= 19.7 Hz, C-2´), 33.5 (t, $^3J_{CP}$= 6.8 Hz, C-b^1), 33.1, 31.7, 31.5, 30.79, 30.76, 30.73, 30.6, 30.5, 30.3, 23.7 (C-c^2, C-d^2, C-e, C-f, C-g, C-h, C-i, C-j, C-k, C-l, C-m, C-n, C-o, C-p, C-q), 27.8, 26.0 (C-a^2), 24.8 (C-B), 23.4 (d, $^3J_{CP}$= 5.3 Hz, C-b^2), 20.7 (t, $^4J_{CP}$= 1.4 Hz, C-C), 19.9 (C-c^1), 14.5 (C-r), 14.03 (C-d^1), 13.95 (C-D), 11.6, 9.1 (HN(CH$_2$CH$_3$)$_3^+$). **^{31}P NMR (243 MHz, CD$_3$OD):** δ [ppm] = 24.1 (d, $^2J_{pp}$=23.5 Hz, P-γ), -11.82 (d, $^2J_{pp}$=17.6 Hz, P-α), -23.51 (t, $^2J_{pp}$= 21.5 Hz, P-β). **^{19}F-NMR (188 MHz, CD$_3$OD):** δ [ppm] = -175.4- -176.6. **HRMS (ESI$^-$, m/z):** calculated for $C_{31}H_{58}$ $FN_2O_{12}P_3$ [M-H]$^-$ 761.3114; found, 761.3110.

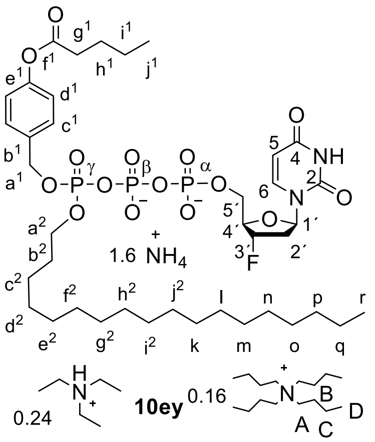

γ-(AB-C4;C18)-FddUTP 10ey. According to general procedure 1 with 157 mg *H*-phosphonate **15a** (0.3 mmol, 1.0 equiv.), 40 mg NCS (0.3 mmol, 1.0 equiv.), and 233 mg (n-$Bu_4N)_3$·FddUDP (0.21 mmol, 0.7 equiv.). Reaction time was 3 h. Yield: 126 mg (0.13 mmol, 60%) white solid. HPLC-UV analysis confirmed purity: > 98%. **^{1}H-NMR (600 MHz, CD_3OD):** δ [ppm]= 8.00 (d, $^4J_{HH}$= 8.1 Hz, 1H, H-6), 7.52-7.47 (m, 2H, H-c^1), 7.12-7.07 (m, 2H, H-d^1), 6.35 (dd, $^3J_{HH}$= 9.6 Hz, $^3J_{HH}$= 5.3 Hz, 1H, H-1´), 5.83 (dd, $^3J_{HH}$= 8.2 Hz, $^3J_{HH}$= 2.1 Hz, 1H, H-5), 5.48 (dd, $^2J_{HH}$= 53.1 Hz, $^3J_{HH}$= 2.9 Hz, 1H, H-3´), 5.24-5.18 (m, 2H, H-a^1), 4.42-4.33 (m, 1H, H-4´), 4.32-4.25 (m, 1H, H-5´$_a$), 4.19-4.14 (m, 1H, H-5´$_b$), 4.14-4.08 (m, 2H, H-a^2), 3.28-3.21 (m, 1.28H, H-A), 3.20-3.14 (m, 1.44H, $HN(CH_2CH_3)_3^+$), 2.57 (t, $^3J_{HH}$= 7.4 Hz, 2H, H-g^1), 2.55-2.28 (m, 2H, H-2´), 1.71 (quint, $^3J_{HH}$= 7.4 Hz, 2H, H-h^1), 1.68-1.62 (m, 3.28H, H-B, H-b^2), 1.49-1.37 (m, 3.28H, H-C, H-i^1), 1.34-1.25 (m, 30.16H, $HN(CH_2CH_3)_3^+$, H-c^2, H-d^2, H-e^2, H-f^2, H-j^2, H-h^2, H-i^2, H-j^2, H-k, H-l, H-m, H-n, H-o, H-p, H-q), 1.02 (t, $^3J_{HH}$= 7.4 Hz, 1.92H, H-D), 0.98 (t, $^3J_{HH}$= 7.3 Hz, 3H, H-j^1), 0.90 (t, $^3J_{HH}$= 7.0 Hz, 3H, H-r). **^{13}C-NMR (151 MHz, CD_3OD):** δ [ppm] = 173.7 (C-f^1), 166.1 (C-4), 152.4 (C-e^1), 152.3 (C-2), 142.4 (C-6), 135.1 (d, $^3J_{CP}$= 7.3 Hz, C-b^1), 130.4 (d, $^3J_{CP}$= 2.3 Hz, C-c^1), 122.9 (C-d^1), 103.6 (C-5), 96.2 (d,$^1J_{CF}$= 175.2 Hz, C-3´), 86.2 (C-1´), 85.5 (dd, $^3J_{CP}$= 25.4 Hz, $^3J_{CP}$= 9.1 Hz, C-4´), 70.2 (dd, $^3J_{CP}$= 5.6 Hz, $^3J_{CP}$= 2.8 Hz, C-a^1), 69.8 (dd, $^3J_{CP}$= 6.2 Hz, $^4J_{CP}$= 1.7 Hz, C-a^2), 66.8 (dd, $^2J_{CP}$= 11.9 Hz, $^3J_{CP}$= 5.6 Hz, C-5´), 59.5 (t, $^3J_{CP}$= 2.8 Hz, C-A), 47.5, 43.2 ($HN(CH_2CH_3)_3^+$), 39.1 (d, $^2J_{CF}$= 20.4 Hz, C-2´), 34.8 (C-g^1), 31.2 (d, $^3J_{CP}$= 7.2 Hz, C-b^2), 33.1, 30.79, 30.75, 30.73, 30.67, 30.5, 30.3, 23.7 (C-d^2, C-e^2, C-f^2, C-g^2, C-h^2, C-i^2, C-j^2, C-k, C-l, C-m, C-n, C-o, C-p, C-q), 28.1 (C-h^1), 26.5 (C-c^2), 24.8 (C-B), 23.2 (C-i^1), 20.7 (C-C), 14.5 (C-r), 14.1 (C-j^1), 13.9 (C-D), 11.6, 9.1 ($HN(CH_2CH_3)_3^+$). **^{31}P NMR (243 MHz, CD_3OD):** δ [ppm] = -11.90 (d, $^2J_{pp}$=17.4 Hz, P-α), -12.93 (dd, $^2J_{pp}$=17.7 Hz, $^3J_{pp}$=5.8 Hz, P-γ), -23.69 (t, $^2J_{pp}$= 17.7 Hz, P-β). **^{19}F-NMR (188 MHz, CD_3OD):** δ [ppm] = -175.3 -- -176.0. **HRMS (ESI$^-$, m/z):** calculated for $C_{39}H_{64}FN_2O_{15}P_3$ [M-H]$^-$ 911.3431; found, 911.3368.

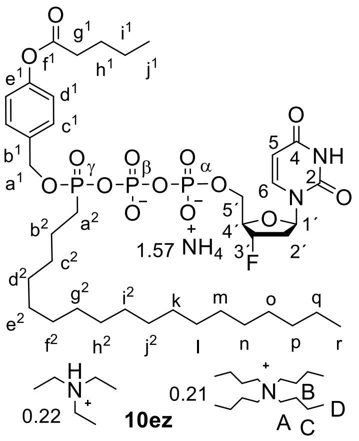

γ-(AB-C4)-γ-C-(C18)-FddUTP 10ez. According to general procedure 1 with 153 mg *H*-phosphinate **15b** (0.3 mmol, 1.0 equiv.), 40 mg NCS (0.3 mmol, 1.0 equiv.), and 233 mg (n-$Bu_4N)_3$·FddUDP (0.21 mmol, 0.7 equiv.). Reaction time was 3 h. Yield: 157 mg (0.16 mmol, 75%) white solid. HPLC-UV analysis confirmed purity: > 98%. **^{1}H NMR (600 MHz, CD_3OD):** δ [ppm] = 8.01 (d, $^3J_{HH}$= 8.1 Hz, 1H, H-6), 7.52-7.47 (m, 2H, H-c^1), 7.12-7.06 (m, 2H, H-d^1), 6.35 (dd, $^3J_{HH}$= 9.6 Hz, $^3J_{HH}$= 5.3 Hz, 1H, H-1´), ´), 5.81 (dd, $^3J_{HH}$= 8.1 Hz, $^4J_{HH}$= 0.6 Hz, 1H, H-5), 5.48 (dt, $^2J_{HH}$= 53.2 Hz, $^3J_{HH}$= 3.5 Hz, 1H, H-3´), 5.26-5.15 (m, 2H, H-a^1), 4.42-4.33 (m, 1H, H-4´), 4.32-4.25 (m, 1H, H-5´$_a$), 4.19-4.14 (m, 1H, H-5´$_b$), 3.26-3.21 (m, 1.68H, H-A), 3.20-3.15 (m, 1.32H, $HN(CH_2CH_3)_3^+$), 2.58 (t, $^3J_{HH}$= 7.4 Hz, 2H, H-g^1), 2.55-2.28 (m, 2H, H-2´), 2.06-1.95 (m, 2H, H-a^2), 1.71 (quint, $^3J_{HH}$= 7.4 Hz, 2H, H-h^1), 1.68-1.55 (m, 3.68H, H-B, H-b^2), 1.49-1.35 (m, 5.68H, H-C, H-i^1, H-c^2), 1.37-1.24 (m, 29.98 H, $HN(CH_2CH_3)_3^+$, H-d^2, H-e^2, H-f^2, H-g^2, H-h^2, H-i^2, H-j^2, H-k, H-l, H-m, H-n, H-o, H-p, H-q), 1.02 (t, $^3J_{HH}$= 7.4 Hz, 2.52H, H-D), 0.98 (t, $^3J_{HH}$= 7.3 Hz, 3H, H-j^1), 0.90 (t, $^3J_{HH}$= 7.0 Hz, 3H, H-r). **^{13}C-NMR (101 MHz, CD_3OD):** δ [ppm] = 173.7 (C-f^1), 166.1 (C-4), 152.3 (C-e^1), 152.2 (C-2), 142.4 (C-6), 135.6

(d, $^{3}J_{CP}$= 7.3 Hz, C-b^{1}), 130.4 (d, $^{3}J_{CP}$= 2.3 Hz, C-c^{1}), 122.8 (C-d^{1}), 103.6 (C-5), 96.2 (d,$^{1}J_{CF}$= 175.4 Hz, C-3´), 86.2 (C-1´), 85.5 (dd, $^{3}J_{CP}$= 25.4 Hz, $^{3}J_{CP}$= 9.1 Hz, C-4´), 67.9 (t, $^{3}J_{CP}$= 6.6 Hz, C-a^{1}), 66.8 (dd, $^{3}J_{CP}$= 11.8 Hz, $^{3}J_{CP}$= 5.7 Hz, C-5´), 59.5 (t, $^{3}J_{CP}$= 2.8 Hz, C-A), 47.5, 43.2 (HN(CH$_2$CH$_3$)$_3^+$), 39.1 (d, $^{2}J_{CF}$= 20.8 Hz, C-2´), 34.8 (C-g^{1}), 31.5 (d, $^{3}J_{CP}$= 17.5 Hz, C-b^{2}), 33.1, 30.80, 30.75, 30.72, 30.54, 30.47, 30.2, 23.7 (C-c^{2}, C-d^{2}, C-e^{2}, C-f^{2}, C-g^{2}, C-h^{2}, C-i^{2}, C-j^{2}, C-k, C-l, C-m, C-n, C-o, C-p, C-q), 28.1 (C-h^{1}), 27.5, 26.5 (C-a^{2}), 24.8 (C-B), 23.3 (d, $^{2}J_{CP}$= 5.3 Hz, C-b^{2}), 23.2 (C-i^{1}), 20.7 (C-C), 14.4 (C-r), 14.1 (C-j^{1}), 13.9 (C-D), 11.6, 9.2 (HN(CH$_2$CH$_3$)$_3^+$). **^{31}P NMR (162 MHz, CD$_3$OD):** δ [ppm] = 24.45 (d, $^{2}J_{pp}$=23.4 Hz, P-γ), -11.80 (d, $^{2}J_{pp}$=25.5 Hz, P-α), -23.54 (t, $^{2}J_{pp}$= 22.1 Hz, P-β). **^{19}F-NMR (188 MHz, CD$_3$OD):** δ [ppm] = -175.0 -- -176.5. **HRMS (ESI$^-$, m/z):** calculated for $C_{39}H_{64}FN_2O_{14}P_3$ [M-H]$^-$ 895.3482; found, 895.3554.

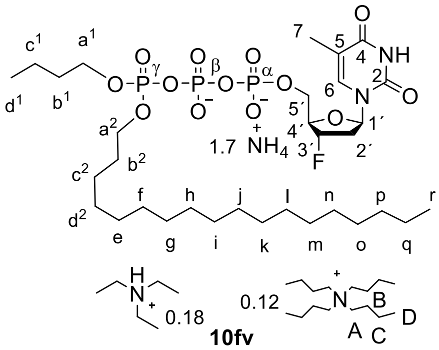

γ-(C4;C18)-FLTTP 10fv. According to general procedure 2 with 117 mg *H*-phosphonate **15ca** (0.3 mmol, 1.0 equiv.), 80 mg NCS (0.6 mmol, 2.0 equiv.), and 169 mg (*n*-Bu$_4$N)$_2$·FLTMP (0.21 mmol, 0.7 equiv.). Reaction time was 3 h. Yield: 102 mg (0.12 mmol, 56%) white solid. HPLC-UV analysis confirmed purity: > 99%. **^{1}H NMR (400 MHz, CD$_3$OD):** δ [ppm] = 7.83 (d, $^{4}J_{HH}$= 1.1 Hz, 1H, H-6), 6.42-6.34 (m, 1H, H-1´), 5.50 (dd, $^{2}J_{HH}$= 53.8 Hz, $^{3}J_{HH}$=2.4 Hz, 1H, H-3´), 4.44-4.32 (m, 1H, H-4´), 4.32-4.10 (m, 6H, H-5´, H-a^{1}, H-a^{2}), 3.27-3.22 (m, 0.96H, H-A), 3.21 (q, $^{3}J_{HH}$= 7.5 Hz, 1.08H, HN(CH$_2$CH$_3$)$_3^+$), 2.55-2.28 (m, 2H, H-2´), 1.94 (d, $^{4}J_{HH}$= 1.0 Hz, 3H, H-7), 1.75-1.60 (m, 4.96H, H-b^{1}, H-b^{2}, H-B), 1.49-1.36 (m, 4.96H, H-c^{1}, H-c^{2}, H-C), 1.36-1.25 (m, 29.62H, HN(CH$_2$CH$_3$)$_3^+$, H-d^{2}, H-e, H-f, H-j, H-h, H-i, H-j, H-k, H-l, H-m, H-n, H-o, H-p, H-q), 1.02 (t, $^{3}J_{HH}$= 7.4 Hz, 1.44 H, H-D), 0.95 (t, $^{3}J_{HH}$= 7.4 Hz, 3H, H-d^{1}), 0.90 (d, $^{3}J_{HH}$= 7.0 Hz, 3H, H-r). **^{13}C NMR (101 MHz, CD$_3$OD):** δ [ppm] = 166.4 (C-4), 152.5 (C-2), 137.9 (C-6), 112.4 (C-5), 95.3 (d, $^{1}J_{CF}$= 175.1 Hz, C-3´), 86.0 (C-1´), 85.3 (dd, $^{2}J_{CP}$= 25.0 Hz, $^{3}J_{CP}$= 9.6 Hz, C-4´), 69.7, 69.3 (2 x d, $^{3}J_{CP}$= 6.3 Hz, $^{3}J_{CP}$= 6.3 Hz, C-a^{1}, C-a^{2}), 66.7 (dd, $^{2}J_{CP}$= 11.6 Hz, $^{3}J_{CP}$= 5.6 Hz, C-5´), 59.5 (t, $^{3}J_{CP}$= 2.8 Hz, C-A), 47.5 (HN(CH$_2$CH$_3$)$_3^+$), 38.8 (d, $^{2}J_{CP}$= 20.7 Hz, C-2´), 33.3 (d, $^{3}J_{CP}$= 7.4 Hz, C-b^{1}), 31.3 (t, $^{3}J_{CP}$= 7.3 Hz, C-b^{2}), 33.1, 30.78, 30.75, 30.70, 30.68, 30.5, 30.3, 23.7 (C-d^{2}, C-e, C-f, C-g, C-h, C-i, C-j, C-k, C-l, C-m, C-n, C-o, C-p, C-q), 26.6 (C-c^{2}), 24.8 (C-B), 20.7 (C-C), 19.8 (C-c^{1}), 14.5 (C-r), 13.99, 13.95 (C-d^{1}, C-D), 12.6 (C-7), 9.1 (HN(CH$_2$CH$_3$)$_3^+$). **^{31}P NMR (162 MHz, CD$_3$OD):** δ [ppm] = -8.05 (d, $^{2}J_{pp}$= 17.6 Hz, P-γ), -8.74 (d, $^{2}J_{pp}$= 17.6 Hz, P-α), -19.56 (t, $^{2}J_{pp}$= 17.2 Hz, P-β). **HRMS (ESI$^-$, m/z):** calculated for $C_{32}H_{60}FN_2O_{13}P_3$ [M-H]$^-$ 791.3220; found, 791.3231. **^{19}F-NMR (188 MHz, CD$_3$OD):** δ [ppm] = -171.2 -- -171.9.

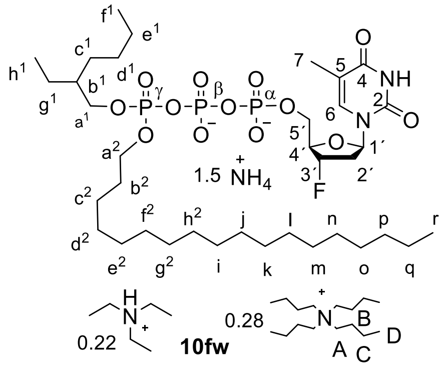

γ-(C8;C18)-FLTTP 10fw. According to general procedure 2 with 134 mg *H*-phosphonate **15cb** (0.3 mmol, 1.0 equiv.), 80 mg NCS (0.6 mmol, 2.0 equiv.), and 169 mg (*n*-Bu$_4$N)$_2$·FLTMP (0.21 mmol, 0.7 equiv.). Reaction time was 3 h. Yield: 107 mg (0.11 mmol, 53%) white solid. HPLC-UV analysis confirmed purity: > 98%. **^{1}H NMR (400 MHz, CD$_3$OD):** δ [ppm] = 7.84 (d, $^{4}J_{HH}$= 1.0 Hz, 1H, H-6), 6.42-6.35 (m, 1H, H-1´), 5.50 (dt, $^{2}J_{HH}$= 53.8 Hz, $^{3}J_{HH}$= 2.6 Hz, 1H,

H-3´), 4.44-4.33 (m, 1H, H-4´), 4.32-4.28 (m, 1H, H-5´$_{a}$), 4.24-4.08 (m, 5H, H-5´$_{b}$, H-a^{1}, H-a^{2}), 3.27-3.22 (m, 2.24H, H-A), 3.20 (q, $^{3}J_{HH}$= 7.5 Hz, 1.38H, $HN(CH_2CH_3)_3^+$), 2.55-2.35 (m, 2H, H-2´), 1.94 (d, $^{4}J_{HH}$= 1.2 Hz, 3H, H-7), 1.75-1.60 (m, 4.24H, H-b^{2}, H-B), 1.62-1.56 (m, 1H, H-b^{1}), 1.46-1.25 (m, 42.31H, H-C, $HN(CH_2CH_3)_3^+$, H-c^{1}, H-c^{2}, H-d^{1}, H-d^{2}, H-e^{1}, H-e^{2}, H-f^{2}, H-g^{1}, H-g^{2}, H-h^{2}, H-i, H-j, H-k, H-l, H-m, H-n, H-o, H-p, H-q), 1.02 (t, $^{3}J_{HH}$= 7.4 Hz, 3.36H, H-D), 0.96-0.88 (m, 9H, H-h^{1}, H-f^{1}, H-r). **^{13}C NMR (101 MHz, CD_3OD):** δ [ppm] = 166.4 (C-4), 152.5 (C-2), 137.9 (C-6), 112.4 (C-5), 96.2 (dd, $^{1}J_{CF}$= 175.0 Hz, C-3´), 85.9 (C-1´), 85.4 (dd, $^{2}J_{CP}$= 25.3 Hz, $^{3}J_{CP}$= 9.4 Hz, C-4´), 71.59, 71.55 (C-a^{1}), 69.7 (d, $^{3}J_{CP}$= 6.3 Hz, C-a^{2}), 66.7 (dd, $^{2}J_{CP}$= 11.8 Hz, $^{3}J_{CP}$= 5.7 Hz, C-5´), 59.5 (t, $^{3}J_{CP}$= 2.8 Hz, C-A), 47.5, 43.1 ($HN(CH_2CH_3)_3^+$), 41.4 (d, $^{3}J_{CP}$= 7.4 Hz, C-b^{1}), 38.8 1 (d, $^{2}J_{CF}$= 20.7 Hz, C-2´), 33.1, 31.02, 31.01, 30.78, 30.75, 30.70, 30.5, 30.3, 30.04, 30.02, 24.30, 24.27, 24.1, 23.7 (C-c^{1}, C-d^{1}, C-d^{2}, C-e^{1}, C-e^{2}, C-f^{2}, C-g^{1}, C-g^{2}, C-h^{2}, C-i, C-j, C-k, C-l, C-m, C-n, C-o, C-p, C-q), 26.7 (C-c^{2}), 24.8 (C-B), 20.7 (t, $^{4}J_{CP}$= 1.4 Hz, C-C), 14.47, 14.46 (C-f^{1}, C-r), 14.0 (C-D), 11.3 (d, $^{3}J_{CP}$= 2.2 Hz, C-h^{1}), 11.6, 9.1 ($HN(CH_2CH_3)_3^+$). **^{31}P NMR (162 MHz, CD_3OD):** δ [ppm] = -12.10 (d, $^{2}J_{pp}$= 17.6 Hz, P-γ), -12.60 (d, $^{2}J_{pp}$= 14.7 Hz, P-α), -23.65 (t, $^{2}J_{pp}$= 17.5 Hz, P-β). **HRMS (ESI$^-$, m/z):** calculated for $C_{36}H_{67}FN_2O_{13}P_3$ [M-H]$^-$ 847.3846; found, 847.3622. **^{19}F-NMR (188 MHz, CD_3OD):** δ [ppm] = -175.2 -- -176.5.

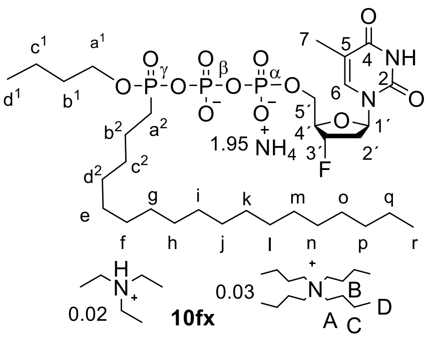

γ-(C4)-γ-C-(C18)-FLTTP 10fx. According to general procedure 2 with 112 mg *H*-phosphinate **15d** (0.3 mmol, 1.0 equiv.), 80 mg NCS (0.6 mmol, 2.0 equiv.), and 169 mg (*n*-$Bu_4N)_2$·FLTMP (0.21 mmol, 0.7 equiv.). Reaction time was 3 h. Yield: 108 mg (0.13 mmol, 63%) white solid. HPLC-UV analysis confirmed purity: > 98%. **^{1}H NMR (400 MHz, CD_3OD):** δ [ppm] = 7.84 (d, $^{4}J_{HH}$= 1.0 Hz, 1H, H-6), 6.40 (dd, $^{3}J_{HH}$= 9.0 Hz, $^{3}J_{HH}$= 5.8 Hz, 1H, H-1´), 5.50 (dt, $^{2}J_{HH}$= 54.6 Hz, $^{3}J_{HH}$= 4.2 Hz, 1H, H-3´), 4.42-4.33 (m, 1H, H-4´), 4.32-4.26 (m, 1H, H-5´$_{a}$), 4.22-4.10 (m, 3H, H-5´$_{b}$, H-a^{1}), 3.27-3.22 (m, 0.24H, H-A), 3.19 (q, $^{3}J_{HH}$= 7.4 Hz, 0.12H, $HN(CH_2CH_3)_3^+$), 2.50-2.35 (m, 2H, H-2´), 2.02-1.97 (m, 2H, H-a^{2}), 1.94 (d, $^{4}J_{HH}$= 1.1 Hz, 3H, H-7), 1.70-1.62 (m, 4.24H, H-B, H-b^{1}, H-b^{2}), 1.47-1.38 (m, 4.24H, H-C, H-c^{1}, H-c^{2}), 1.35-1.25 (m, 28.18H, H-d^{2}, H-e, H-f, H-j, H-h, H-i, H-j, H-k, H-l, H-m, H-n, H-o, H-p, H-q, $HN(CH_2CH_3)_3^+$), 1.03 (t, $^{3}J_{HH}$= 7.4 Hz, 0.36H, H-D), 0.95 (t, $^{3}J_{HH}$= 7.4 Hz, 3H, H-d^{1}), 0.90 (d, $^{3}J_{HH}$= 7.0 Hz, 3H, H-r). **^{13}C NMR (101 MHz, CD_3OD):** δ [ppm] = 166.4 (C-4), 152.5 (C-2), 137.9 (C-6), 112.4 (C-5), 96.1 (dd, $^{1}J_{CF}$= 175.0 Hz, C-3´), 85.9 (C-1´), 85.4 (dd, $^{2}J_{CP}$= 25.3 Hz, $^{3}J_{CP}$= 9.4 Hz, C-4´), 66.9 (C-a^{1}), 66.8 (dd, $^{2}J_{CP}$= 12.9 Hz, $^{3}J_{CP}$= 6.1 Hz, C-5´), 38.8 (d, $^{2}J_{CP}$= 20.1 Hz, C-2´), 33.5 (d, $^{3}J_{CP}$= 7.2 Hz, C-b^{1}), 33.1, 31.6, 31.5, 30.78, 30.75, 30.72, 30.6, 30.5, 30.3, 23.7 (C-c^{2}, C-d^{2}, C-e, C-f, C-g, C-h, C-i, C-j, C-k, C-l, C-m, C-n, C-o, C-p, C-q), 27.4, 26.4 (C-a^{2}), 24.8 (C-B), 23.4 (d, $^{3}J_{CP}$= 5.6 Hz, C-b^{2}), 20.7 (C-C), 19.8 (C-c^{1}), 14.4 (C-r), 14.01 (C-d^{1}), 13.94 (C-D), 12.6 (C-7), 9.2 ($HN(CH_2CH_3)_3^+$). **^{31}P NMR (162 MHz, CD_3OD):** δ [ppm] = 24.2 (d, $^{2}J_{pp}$= 23.4 Hz, P-γ), -11.96 (d, $^{2}J_{pp}$= 20.6 Hz, P-α), -23.45 (t, $^{2}J_{pp}$= 28.8 Hz, P-β). **HRMS (ESI$^-$, m/z):** calculated for $C_{32}H_{60}FN_2O_{12}P_3$ [M-H]$^-$ 775.3270; found, 775.3304. **^{19}F-NMR (188 MHz, CD_3OD):** δ [ppm] = -175.2 -- -176.3.

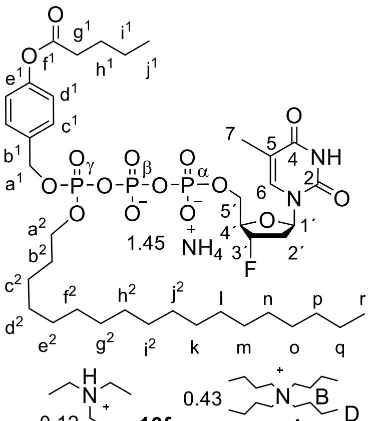

10fy

γ-(AB-C4;C18)-FLTTP 10fy. According to general procedure 2 with 157 mg *H*-phosphonate **15a** (0.3 mmol, 1.0 equiv.), 80 mg NCS (0.6 mmol, 2.0 equiv.), and 169 mg (n-$Bu_4N)_2$·FLTMP (0.21 mmol, 0.7 equiv.). Reaction time was 3 h. Yield: 114 mg (0.11 mmol, 51%) white solid. HPLC-UV analysis confirmed purity: > 97%. **^{1}H NMR (400 MHz, CD_3OD):** δ [ppm] = 7.83 (d, $^{4}J_{HH}$= 1.2 Hz, 1H, H-6), 7.52-7.46 (m, 2H, H-c^1), 7.10-7.06 (m, 2H, H-d^1), 6.40-6.35 (m, 1H, H-1´), 5.54 (dd, $^{2}J_{HH}$= 54.6 Hz, $^{3}J_{HH}$= 2.1 Hz, 1H, H-3´), 5.24-5.16 (m, 2H, H-a^1), 4.40-4.32 (m, 1H, H-4´), 4.32-4.25 (m, 1H, H-5´$_a$), 4.22-4.15 (m, 1H, H-5´$_b$), 4.15-4.06 (m, 2H, H-a^2), 3.28-3.20 (m, 3.44H, H-A), 3.17 (q, $^{3}J_{HH}$= 7.2 Hz, 0.72H, $HN(CH_2CH_3)_3^+$), 2.58 (t, $^{3}J_{HH}$= 7.4 Hz, 2H, H-g^1), 2.46-2.32 (m, 2H, H-2´), 1.93 (dd, $^{3}J_{HH}$= 2.9 Hz, $^{4}J_{HH}$= 1.2 Hz, 3H, H-7), 1.74-1.68 (m, 2H, H-h^1), 1.67-1.58 (m, 5.44H, H-b^2, H-B), 1.50-1.38 (m, 5.44H, H-C, H-i^1), 1.36-1.25 (m, 31.08H, H-c^2, H-d^2, H-e^2, H-f^2, H-g^2, H-h^2, H-i^2, H-j^2, H-k, H-l, H-m, H-n, H-o, H-p, H-q, $HN(CH_2CH_3)_3^+$), 1.02 (t, $^{3}J_{HH}$= 7.3 Hz, 5.16H, H-D), 0.98 (t, $^{3}J_{HH}$= 7.4 Hz, 3H, H-j^1), 0.90 (t, $^{3}J_{HH}$= 7.0 Hz, 3H, H-r). **^{13}C NMR (101 MHz, CD_3OD):** δ [ppm] = 173.7 (C-f^1), 166.4 (C-4), 152.5 (C-2), 152.3 (C-e^1), 137.9 (C-6), 135.1 (d, $^{3}J_{CP}$= 6.4 Hz, C-b^2), 130.4 (d, $^{3}J_{CP}$= 2.8 Hz, C-c^1), 122.8 (C-d^1), 112.4 (C-5), 96.3 (d, $^{1}J_{CF}$= 174.9 Hz, C-3´), 85.9 (C-1´), 85.4 (dd, $^{2}J_{CP}$= 25.5 Hz, $^{3}J_{CP}$= 9.5 Hz, C-4´), 70.2 (dd, $^{3}J_{CP}$= 5.6 Hz, $^{3}J_{CP}$= 3.3 Hz, C-a^1), 69.8 (d, $^{3}J_{CP}$= 6.2 Hz, C-a^2), 66.8 (dd, $^{2}J_{CP}$= 11.9 Hz, $^{3}J_{CP}$= 5.2 Hz, C-5´), 59.5 (t, $^{3}J_{CP}$= 2.6 Hz, C-A), 47.4, ($HN(CH_2CH_3)_3^+$), 38.8 (d, $^{2}J_{CF}$= 20.4 Hz, C-2´), 34.8 (C-g^1), 31.2 (d, $^{3}J_{CP}$= 7.3 Hz, C-b^2), 33.1, 30.79, 30.78, 30.74, 30.71, 30.5, 30.3, 23.7 (C-d^2, C-e^2, C-f^2, C-g^2, C-h^2, C-i^2, C-j^2, C-k, C-l, C-m, C-n, C-o, C-p, C-q), 28.1 (C-h^1), 26.5 (C-c^2), 24.8 (C-B), 23.2 (C-i^1), 20.7 (t, $^{4}J_{CP}$= 1.4 Hz, C-C), 14.5 (C-r), 14.1 (C-j^1), 13.9 (C-D), 12.6 (C-7), 9.14 ($HN(CH_2CH_3)_3^+$). **^{31}P NMR NMR (162 MHz, CD_3OD):** δ [ppm] = -12.07 (d, $^{2}J_{pp}$= 20.6 Hz, P-γ), -12.92 (dd, $^{2}J_{pp}$= 14.8 Hz, $^{3}J_{pp}$= 5.7 Hz, P-α), -23.68 (t, $^{2}J_{pp}$= 17.5 Hz, P-β). **MALDI-MS (m/z):** calculated for $C_{40}H_{66}FN_2O_{15}P_3$ [M-H]$^-$ 925.3587; found, 925.3384. **^{19}F-NMR (188 MHz, CD_3OD):** δ [ppm] = -175.0 -- -176.0.

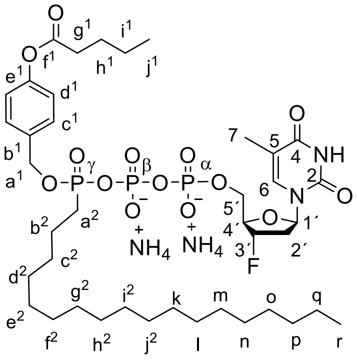

10fz

γ-(AB-C4)-γ-C-(C18)-FLTTP 10fz. According to general procedure 2 with 153 mg *H*-phosphinate **15b** (0.3 mmol, 1.0 equiv.), 80 mg NCS (0.6 mmol, 2.0 equiv.), and 169 mg (n-$Bu_4N)_2$·FLTMP (0.21 mmol, 0.7 equiv.). Reaction time was 3 h. Yield: 95 mg (0.10 mmol, 48%) white solid. HPLC-UV analysis confirmed purity: > 98%. **^{1}H NMR (400 MHz, CD_3OD):** δ [ppm] = 7.83 (t, $^{4}J_{HH}$= 1.3 Hz, 1H, H-6), 7.52-7.46 (m, 2H, H-c^1), 7.12-7.05 (m, 2H, H-d^1), 6.37 (dd, $^{3}J_{HH}$= 9.4 Hz, $^{3}J_{HH}$= 5.8 Hz, 1H, H-1´), 5.48 (dt, $^{2}J_{HH}$= 53.6 Hz, $^{3}J_{HH}$= 3.6 Hz, 1H, H-3´), 5.26-5.15 (m, 2H, H-a^1), 4.40-4.32 (m, 1H, H-4´), 4.32-4.26 (m, 1H, H-5´$_a$), 4.22-4.15 (m, 1H, H-5´$_b$), 2.58 (t, $^{3}J_{HH}$= 7.4 Hz, 2H, H-g^1), 2.55-2.32 (m, 2H, H-2´), 2.07-1.96 (m, 2H, H-a^2), 1.94-1.88 (m, 3H, H-7), 1.75-1.68 (m, 2H, H-h^1), 1.67-1.55 (m, 2H, H-b^2), 1.50-1.42 (m, 2H, H-i^1), 1.40-1.34 (m, 2H, H-c^2), 1.36-1.25 (m, 28H, H-d^2, H-e^2, H-f^2, H-g^2, H-h^2, H-i^2, H-j^2, H-k, H-l, H-m, H-n, H-o, H-p, H-q), 0.98 (t, $^{3}J_{HH}$= 7.4 Hz, 3H, H-j^1), 0.90 (t, $^{3}J_{HH}$= 7.0 Hz, 3H, H-r). **^{13}C NMR (101 MHz, CD_3OD):** δ [ppm] = 173.7 (C-f^1), 166.4 (C-4), 152.5 (C-2), 152.3 (C-e^1), 137.9 (C-6), 135.5 (dd, $^{3}J_{CP}$= 6.8 Hz, $^{3}J_{CP}$= 2.3 Hz, C-b^2), 130.3 (d, $^{3}J_{CP}$= 8.5 Hz, C-c^1), 122.8 (C-d^1), 112.4 (C-5), 96.3 (d, $^{1}J_{CF}$= 175.1 Hz, C-3´), 85.9 (C-1´), 85.3 (dd, $^{2}J_{CP}$= 25.4 Hz, $^{3}J_{CP}$= 9.1 Hz, C-4´), 67.9 (dd,

$^{3}J_{CP}$= 6.9 Hz, $^{3}J_{CP}$= 5.2 Hz, C-a^{1}), 66.8 (dd, $^{2}J_{CP}$= 12.4 Hz, $^{3}J_{CP}$= 5.7 Hz, C-5´), 38.8 (d, $^{2}J_{CF}$= 20.4 Hz, C-2´), 34.8 (C-g^{1}), 33.1, 31.6, 31.4, 30.79, 30.75, 30.71, 30.53, 30.47, 30.2, 23.7 (C-d^{2}, C-e^{2}, C-f^{2}, C-g^{2}, C-h^{2}, C-i^{2}, C-j^{2}, C-k, C-l, C-m, C-n, C-o, C-p, C-q), 28.1 (C-h^{1}), 27.5, 26.6 (C-a^{2}), 23.28 (C-b^{2}), 23.25 (C-i^{1}), 14.4 (C-r), 14.1 (C-j^{1}), 12.6 (C-7). **^{31}P NMR NMR (162 MHz, CD$_3$OD):** δ [ppm] = 24.5 (d, $^{2}J_{pp}$= 23.5 Hz, P-γ), -12.0 (d, $^{2}J_{pp}$= 17.7 Hz, P-α), -23.6 (t, $^{2}J_{pp}$= 22.1 Hz, P-β). **HRMS (ESI$^-$, m/z):** calculated for $C_{40}H_{66}FN_2O_{14}P_3$ [M-H]$^-$ 909.3638; found, 909.3633. **^{19}F-NMR (188 MHz, CD$_3$OD):** δ [ppm] = -175.0 -- -176.5.

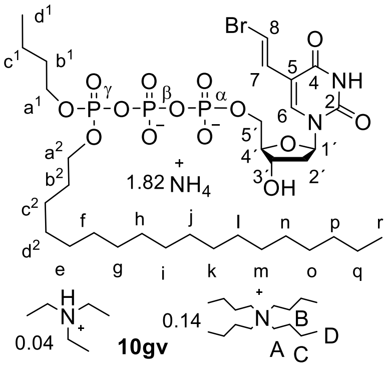

γ-(C4;C18)-BVDUTP 10gv. According to general procedure 2 with 117 mg *H*-phosphonate **15ca** (0.3 mmol, 1.0 equiv.), 80 mg NCS (0.6 mmol, 2.0 equiv.), and 188 mg (*n*-Bu$_4$N)$_2$·BVdUMP (0.21 mmol, 0.7 equiv.). Reaction time was 4 h. Yield: 96 mg (0.10 mmol, 48%) white solid. HPLC-UV analysis confirmed purity: > 95%. **^{1}H-NMR (500 MHz, CD$_3$OD):** δ [ppm] = 8.02 (s, 1H, H-6), 7.42 (d, $^{2}J_{HH}$= 13.5 Hz, 1H, H-8), 7.03 (d, $^{2}J_{HH}$= 13.6 Hz, 1H, H-7), 6.33-6.29 (m, 1H, H-1´), 4.64-4.56 (m, 1H, H-3´), 4.30-4.18 (m, 6H, H-5´, H-a^{1}, H-a^{2}), 4.07-4.03 (m, 1H, H-4´), 3.28-3.21 (m, 1.12H, H-A), 3.20-3.17 (m, 0.24H, HN(CH$_2$CH$_3$)$_3^+$), 2.33-2.22 (m, 2H, H-2´), 1.73-1.64 (m, 5.12H, H-B, H-b^{1}, H-b^{2}), 1.46-1.37 (m, 5.12H, H-C, H-c^{1}, H-c^{2}), 1.35-1.26 (m, 28.36H, HN(CH$_2$CH$_3$)$_3^+$, H-d^{2}, H-e, H-f, H-j, H-h, H-i, H-j, H-k, H-l, H-m, H-n, H-o, H-p, H-q), 1.03 (t, $^{3}J_{HH}$= 7.4 Hz, 1.68H, H-D), 0.95 (t, $^{3}J_{HH}$= 7.4 Hz, 3H, H-d^{1}), 0.90 (t, $^{3}J_{HH}$= 7.0 Hz, 3H, H-r). **^{13}C-NMR (126 MHz, CD$_3$OD):** δ [ppm] = 163.8 (C-4), 151.2 (C-2), 140.3 (C-6), 130.8 (C-7), 112.5 (C-5), 109.2 (C-8), 87.5 (d, $^{3}J_{CP}$= 9.5 Hz, C-4´), 86.4 (C-1´), 72.2 (C-3´), 69.7 (dd, $^{3}J_{CP}$= 6.7 Hz, $^{4}J_{CP}$= 1.2 Hz, C-a^{1}), 69.4 (dd, $^{3}J_{CP}$= 6.6 Hz, $^{4}J_{CP}$= 1.2 Hz, C-a^{2}), 66.6 (d, $^{3}J_{CP}$= 5.7 Hz, C-5´), 59.5 (t, $^{3}J_{CP}$= 2.6 Hz, C-A), 40.7 (C-2´), 33.3 (d, $^{3}J_{CP}$= 7.3 Hz, C-b^{1}), 31.3 (t, $^{3}J_{CP}$= 7.3 Hz, C-b^{2}), 33.1, 30.79, 30.76, 30.71, 30.69, 30.5, 30.3, 23.7 (C-d^{2}, C-e, C-f, C-g, C-h, C-i, C-j, C-k, C-l, C-m, C-n, C-o, C-p, C-q), 26.6 (C-c^{2}), 24.8 (C-B), 20.7 (C-C), 19.8 (C-c^{1}), 14.4 (C-r), 14.00 (C-d^{1}), 13.94 (C-D). **^{31}P NMR (162 MHz, CD$_3$OD):** δ [ppm] = -11.64 (d, $^{2}J_{pp}$= 20.6 Hz, P-α), -11.79 (d, $^{2}J_{pp}$= 17.6 Hz, P-γ), -23.62 (t, $^{2}J_{pp}$= 17.6 Hz, P-β). **HRMS (ESI$^-$, m/z):** calculated for $C_{33}H_{60}BrN_2O_{14}P_3$ [M-H]$^-$ 879.2368; found, 863.2333.

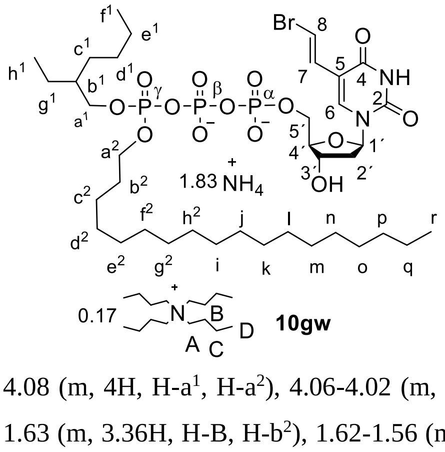

γ-(C8;C18)-BVDUTP 10gw. According to general procedure 2 with 134 mg *H*-phosphonate **15cb** (0.3 mmol, 1.0 equiv.), 80 mg NCS (0.6 mmol, 2.0 equiv.), and 188 mg (*n*-Bu$_4$N)$_2$·BVdUMP (0.21 mmol, 0.7 equiv.). Reaction time was 5 h. Yield: 117 mg (0.12 mmol, 55%) white solid. HPLC-UV analysis confirmed purity: > 98%. **^{1}H-NMR (500 MHz, CD$_3$OD):** δ [ppm] = 8.01 (s, 1H, H-6), 7.42 (d, $^{2}J_{HH}$= 13.5 Hz, 1H, H-8), 7.03 (d, $^{2}J_{HH}$= 13.6 Hz, 1H, H-7), 6.34-6.29 (m, 1H, H-1´), 4.63-4.57 (m, 1H, H-3´), 4.30-4.21 (m, 2H, H-5´), 4.30-4.08 (m, 4H, H-a^{1}, H-a^{2}), 4.06-4.02 (m, 1H, H-4´), 3.27-3.21 (m, 1.36H, H-A), 2.34-2.21 (m, 2H, H-2´), 1.74-1.63 (m, 3.36H, H-B, H-b^{2}), 1.62-1.56 (m, 1.36H, H-b^{1}), 1.46-1.25 (m, 39.36H, H-C, H-c^{1}, H-c^{2}, H-d^{1}, H-d^{2}, H-e^{1}, H-e^{2}, H-f^{2}, H-g^{1}, H-g^{2}, H-h^{2}, H-i, H-j, H-k, H-l, H-m, H-n, H-o, H-p, H-q), 1.03 (t, $^{3}J_{HH}$= 7.4 Hz, 2.04H, H-D), 0.94-0.88 (m, 9H, H-h^{1}, H-f^{1}, H-r). **^{13}C-NMR (126 MHz, CD$_3$OD):** δ [ppm] = 163.8 (C-4), 151.2 (C-2), 140.3

(C-6), 130.8 (C-7), 112.5 (C-5), 109.2 (C-8), 87.4 (d, $^3J_{CP}$= 9.2 Hz, C-4´), 86.4 (C-1´), 72.2 (C-3´), 71.7, 71.6 (C-a^1), 69.8 (dd, $^3J_{CP}$= 6.2 Hz, $^3J_{CP}$= 2.2 Hz, C-a^2), 66.7 (d, $^3J_{CP}$= 5.7 Hz, C-5´), 59.5 (t, $^3J_{CP}$= 2.8 Hz, C-A), 41.4 (d, $^3J_{CP}$= 7.4 Hz, C-b^1), 40.7 (C-2´), 31.3 (t, $^3J_{CP}$= 7.3 Hz, C-b^2), 33.1, 31.0, 30.78, 30.76, 30.71, 30.5, 30.3, 30.07, 30.06, 23.7 (C-c^1, C-d^1, C-d^2, C-e^1, C-e^2, C-f^2, C-g^1, C-g^2, C-h^2, C-i, C-j, C-k, C-l, C-m, C-n, C-o, C-p, C-q), 26.7 (C-c^2), 24.8 (C-B), 20.7 (C-C), 14.47, 14.46 (C-f^1, C-r), 14.0 (C-D), 11.4 (C-h^1). **^{31}P NMR (162 MHz, CD_3OD):** δ [ppm] = -11.72 (d, $^2J_{pp}$= 17.8 Hz, P-α), -12.70 (d, $^2J_{pp}$= 17.5 Hz, P-γ), -23.75 (t, $^2J_{pp}$= 17.6 Hz, P-β). **MALDI-MS (m/z):** calculated for $C_{37}H_{68}BrN_2O_{14}P_3$ [M-H]$^-$ 935.2994; found, 935.4031.

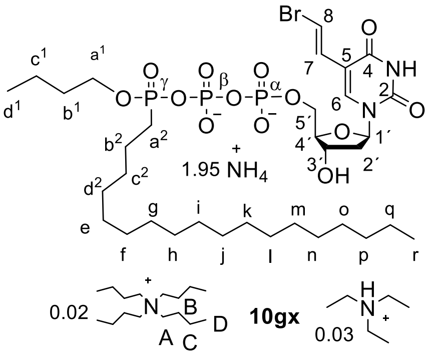

γ-(C4)-γ-C-(C18)-BVDUTP 10gx. According to general procedure 2 with 112 mg *H*-phosphinate **15d** (0.3 mmol, 1.0 equiv.), 80 mg NCS (0.6 mmol, 2.0 equiv.), and 188 mg (n-Bu_4N)$_2$·BVdUMP (0.21 mmol, 0.7 equiv.). Reaction time was 5 h. Yield: 120 mg (0.13 mmol, 63%) white solid. HPLC-UV analysis confirmed purity: > 99%. **^{1}H-NMR (500 MHz, CD_3OD):** δ [ppm] = 8.01 (s, 1H, H-6), 7.42 (d, $^2J_{HH}$= 13.5 Hz, 1H, H-8), 7.03 (d, $^2J_{HH}$= 13.6 Hz, 1H, H-7), 6.34-6.27 (m, 1H, H-1´), 4.63-4.54 (m, 1H, H-3´), 4.30-4.10 (m, 4H, H-a^1, H-5´), 4.07-4.02 (m, 1H, H-4´), 3.28-3.20 (m, 0.16H, H-A), 3.20-3.16 (m, 0.18H, $HN(CH_2CH_3)_3^+$), 2.34-2.21 (m, 2H, H-2´), 2.08-1.92 (m, 2H, H-a^2), 1.74-1.58 (m, 4.16H, H-B, H-b^1, H-b^2), 1.47-1.37 (m, 4.16H, H-C, H-c^1, H-c^2), 1.36-1.25 (m, 28.27H, H-d^2, H-e, H-f, H-g, H-h, H-i, H-j, H-k, H-l, H-m, H-n, H-o, H-p, H-q), 1.02 (t, $^3J_{HH}$= 7.3 Hz, 0.24H, H-D), 0.95 (t, $^3J_{HH}$= 7.4 Hz, 3H, H-d^1), 0.90 (t, $^3J_{HH}$= 6.8 Hz, 3H, H-r). **^{13}C-NMR (126 MHz, CD_3OD):** δ [ppm] = 163.7 (C-4), 151.2 (C-2), 140.3 (C-6), 130.7 (C-7), 112.4 (C-5), 109.2 (C-8), 87.4 (d, $^3J_{CP}$= 9.0 Hz, C-4´), 86.5 (C-1´), 72.1 (C-3´), 71.7 (d, $^3J_{CP}$= 7.7 Hz, C-a^1), 66.7 (d, $^3J_{CP}$= 5.9 Hz, C-5´), 47.5 ($HN(CH_2CH_3)_3^+$), 40.8 (C-2´), 33.5 (d, $^3J_{CP}$= 6.8 Hz, C-b^1), 33.1, 31.6, 31.5, 30.77, 30.74, 30.71, 30.6, 30.5, 30.3, 23.7 (C-c^2, C-d^2, C-e, C-f, C-g, C-h, C-i, C-j, C-k, C-l, C-m, C-n, C-o, C-p, C-q), 27.4, 26.2 (C-a^2), 24.8 (C-B), 23.3 (d, $^3J_{CP}$= 5.6 Hz, C-b^2), 19.9 (C-c^1), 14.5 (C-r), 14.03 (C-d^1), 13.95 (C-D), 9.2 ($HN(CH_2CH_3)_3^+$). **^{31}P NMR NMR (162 MHz, CD_3OD):** δ [ppm] = 24.3 (d, $^2J_{pp}$= 23.6 Hz, P-γ), -11.4 (d, $^2J_{pp}$= 19.6 Hz, P-α), -23.3 (t, $^2J_{pp}$= 21.4 Hz, P-β). **HRMS (ESI$^-$, m/z):** calculated for $C_{33}H_{60}BrN_2O_{13}P_3$ [M-H]$^-$ 863.2419; found, 863.2427.

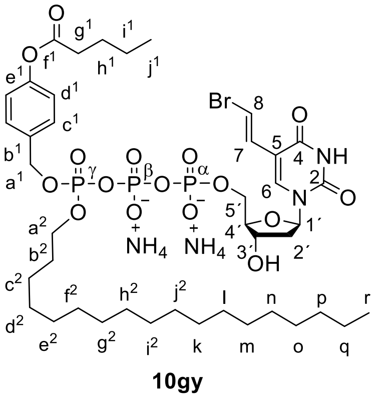

10gy

γ-(AB-C4;C18)-BVDUTP 10gy. According to general procedure 2 with 157 mg *H*-phosphonate **15a** (0.3 mmol, 1.0 equiv.), 80 mg NCS (0.6 mmol, 2.0 equiv.), and 188 mg (n-Bu_4N)$_2$·BVdUMP (0.21 mmol, 0.7 equiv.). Reaction time was 5 h. Yield: 163 mg (0.16 mmol, 74%) white solid. HPLC-UV analysis confirmed purity: > 95%. **^{1}H-NMR (500 MHz, CD_3OD):** δ [ppm] = 7.97 (s, 1H, H-6), 7.52-7.46 (m, 2H, H-c^1), 7.42 (dd, $^2J_{HH}$= 13.5 Hz, $^4J_{HH}$= 1.9 Hz, 1H, H-8), 7.12-7.07 (m, 2H, H-d^1), 7.02 (dd, $^2J_{HH}$= 13.6 Hz, $^4J_{HH}$= 1.2 Hz, 1H, H-7), 6.30 (t, $^3J_{HH}$= 6.7 Hz, 1H, H-1´), 5.24-5.16 (m, 2H, H-a^1), 4.60-4.54 (m, 1H, H-3´), 4.30-4.16 (m, 2H, H-5´), 4.17-4.10 (m, 2H, H-a^2), 4.09-4.04 (m, 1H, H-4´), 2.58 (t, $^3J_{HH}$= 7.5 Hz, 2H, H-g^1), 2.30-2.20 (m, 2H, H-2´), 1.74 (quint, $^3J_{HH}$= 7.5 Hz, 2H, H-h^1), 1.62 (quint, $^3J_{HH}$= 6.1 Hz, 2H, H-b^2), 1.46 (sext, $^3J_{HH}$= 7.5 Hz, 2H, H-i^1), 1.34-1.26 (m, 30H, H-c^2, H-d^2, H-e^2, H-f^2, H-j^2, H-h^2, H-i^2, H-j^2, H-k, H-l, H-m, H-n, H-o, H-p, H-q), 0.98 (t, $^3J_{HH}$=

7.3 Hz, 3H, H-j[1]), 0.90 (t, $^3J_{HH}$= 7.0 Hz, 3H, H-r). **^{13}C-NMR (126 MHz, CD_3OD):** δ [ppm] = 173.7 (C-f[1]), 163.7 (C-4), 152.3 (C-2), 151.1 (C-e[1]), 140.2 (C-6), 135.0 (d, $^3J_{CP}$= 7.4 Hz, C-b[1]), 130.7 (C-7), 130.3 (d, $^3J_{CP}$= 4.6 Hz, C-c[1]), 122.8 (C-d[1]), 112.4 (C-5), 109.2 (C-8), 87.3 (d, $^3J_{CP}$= 8.3 Hz, C-4´), 86.5 (C-1´), 72.1 (d, $^3J_{CP}$= 2.8 Hz, C-3´), 70.3 (dd, $^3J_{CP}$= 5.5 Hz, $^3J_{CP}$= 2.7 Hz, C-a[1]), 69.9 (d, $^3J_{CP}$= 6.6 Hz, C-a[2]), 66.7 (d, $^3J_{CP}$= 5.5 Hz, C-5´), 40.7 (C-2´), 34.7 (C-g[1]), 31.2 (d, $^3J_{CP}$= 7.6 Hz, C-b[2]), 33.0, 30.78, 30.73, 30.71, 30.6, 30.4, 30.3, 23.7 (C-d[2], C-e[2], C-f[2], C-g[2], C-h[2], C-i[2], C-j[2], C-k, C-l, C-m, C-n, C-o, C-p, C-q), 28.0 (C-h[1]), 26.5 (C-c[2]), 23.2 (C-i[1]), 14.5 (C-r), 14.1 (C-j[1]). **^{31}P NMR (162 MHz, CD_3OD):** δ [ppm] = -10.30 (d, $^2J_{pp}$= 18.6 Hz, P-α), -11.70 (d, $^2J_{pp}$= 16.1 Hz, P-γ), -22.22 (t, $^2J_{pp}$= 16.8 Hz, P-β). **MALDI-MS (m/z):** calculated for $C_{41}H_{66}N_2BrO_{16}P_3$ $[M-H]^-$ 1013.276; found, 1013.233.

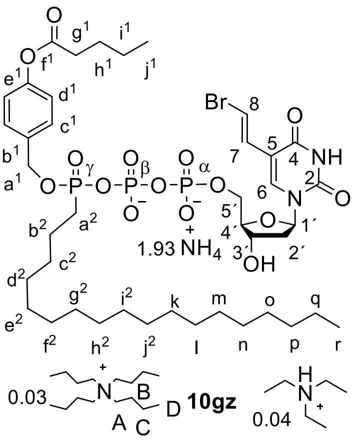

γ-(AB-C4)-γ-C-(C18)-BVDUTP 10gz. According to general procedure 2 with 153 mg *H*-phosphinate **15b** (0.3 mmol, 1.0 equiv.), 80 mg NCS (0.6 mmol, 2.0 equiv.), and 188 mg ($(n\text{-}Bu_4N)_2$·BVdUMP (0.21 mmol, 0.7 equiv.). Reaction time was 4 h. Yield: 125 mg (0.12 mmol, 57%) white solid. HPLC-UV analysis confirmed purity: > 99%. **^{1}H-NMR (500 MHz, CD_3OD):** δ [ppm] = 7.97 (s, 1H, H-6), 7.52-7.46 (m, 2H, H-c[1]), 7.42 (dd, $^2J_{HH}$= 13.5 Hz, $^4J_{HH}$= 1.9 Hz, 1H, H-8), 7.12-7.07 (m, 2H, H-d[1]), 7.02 (dd, $^2J_{HH}$= 13.6 Hz, $^4J_{HH}$= 1.2 Hz, 1H, H-7), 6.30 (t, $^3J_{HH}$= 6.7 Hz, 1H, H-1´), 5.24-5.16 (m, 2H, H-a[1]), 4.60-4.54 (m, 1H, H-3´), 4.30-4.16 (m, 2H, H-5´), 4.17-4.10 (m, 2H, H-a[2]), 4.09-4.04 (m, 1H, H-4´), 2.58 (t, $^3J_{HH}$= 7.5 Hz, 2H, H-g[1]), 2.30-2.20 (m, 2H, H-2´), 1.74 (quint, $^3J_{HH}$= 7.5 Hz, 2H, H-h[1]), 1.62 (quint, $^3J_{HH}$= 6.1 Hz, 2H, H-b[2]), 1.46 (sext, $^3J_{HH}$= 7.5 Hz, 2H, H-i[1]), 1.34-1.26 (m, 30H, H-c[2], H-d[2], H-e[2], H-f[2], H-j[2], H-h[2], H-i[2], H-j[2], H-k, H-l, H-m, H-n, H-o, H-p, H-q), 0.98 (t, $^3J_{HH}$= 7.3 Hz, 3H, H-j[1]), 0.90 (t, $^3J_{HH}$= 7.0 Hz, 3H, H-r). **^{13}C-NMR (126 MHz, CD_3OD):** δ [ppm] = 173.8 (C-f[1]), 163.7 (C-4), 152.2 (C-2), 151.2 (C-e[1]), 140.3 (C-6), 135.5 (dd, $^3J_{CP}$= 7.0 Hz, $^3J_{CP}$= 2.4 Hz, C-b[1]), 130.7 (d, $^3J_{CP}$= 2.1 Hz, C-7), 130.3 (d, $^3J_{CP}$= 9.2 Hz, C-c[1]), 122.8 (C-d[1]), 112.4 (C-5), 109.2 (C-8), 87.4 (d, $^3J_{CP}$= 9.1 Hz, C-4´), 86.5 (C-1´), 72.1 (d, $^3J_{CP}$= 2.2 Hz, C-3´), 67.9 (dd, $^3J_{CP}$= 6.5 Hz, $^3J_{CP}$= 4.5 Hz, C-a[1]), 66.6 (dd, $^3J_{CP}$= 5.5 Hz, $^3J_{CP}$= 2.5 Hz, C-5´), 47.7 ($HN(CH_2CH_3)_3^+$), 40.7 (C-2´), 34.7 (C-g[1]), 31.4 (d, $^3J_{CP}$= 17.6 Hz, C-b[2]), 33.1, 30.78, 30.74, 30.70, 30.53, 30.46, 30.2, 23.7 (C-d[2], C-e[2], C-f[2], C-g[2], C-h[2], C-i[2], C-j[2], C-k, C-l, C-m, C-n, C-o, C-p, C-q), 28.1 (C-h[1]), 27.8, 26.4 (C-a[2]), 24.8 (C-B), 23.29 (C-b[2]), 23.24 (C-i[1]), 20.7 (C-C), 14.4 (C-r), 14.1 (C-j[1]), 13.9 (C-D), 9.2 ($HN(CH_2CH_3)_3^+$). **^{31}P NMR (162 MHz, CD_3OD):** δ [ppm] = 24.6 (d, $^2J_{pp}$= 23.6 Hz, P-γ), -11.4 (d, $^2J_{pp}$= 19.6 Hz, $^3J_{pp}$= 3.9 Hz, P-α), -23.3 (t, $^2J_{pp}$= 21.6 Hz, P-β). **HRMS (ESI$^-$, m/z):** calculated for $C_{41}H_{66}N_2BrO_{15}P_3$ $[M-H]^-$ 997.2787; found, 997.2749.

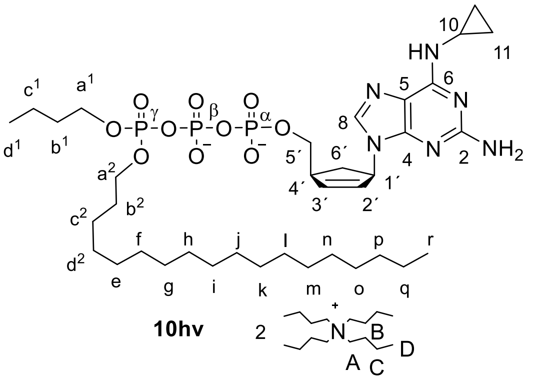

γ-(C4;C18)-ABCTP 10hv. According to general procedure 1 with 117 mg *H*-phosphonate **15ca** (0.3 mmol, 1.0 equiv.), 40 mg NCS (0.3 mmol, 1.0 equiv.), and 175 mg (n-$Bu_4N)_3$·ABCDP (0.15 mmol, 0.5 equiv.). Reaction time was 3 h. Yield: 47 mg (0.04 mmol, 24%) yellow solid. HPLC-UV analysis confirmed purity: > 95%. **^{1}H NMR (400 MHz, CD_3OD):** δ [ppm] = 7.84 (s, 1H, H-8), 6.32-6.27 (m, 1H, H-3´), 5.86-5.81 (m, 1H, H-2´), 5.58-5.50 (m, 1H, H-1´), 4.25-4.05 (m, 6H, H-6´, H-a^1, H-a^2), 3.25-3.20 (m, 16H, H-A), 3.18-3.10 (m, 1H, H-4´), 2.95-2.86 (m, 1H, H-10), 2.83-2.75 (m, 1H, H-5´$_a$), 1.78-1.72 (m, 2H, H-5´$_b$), 1.70-1.60 (m, 20H, H-b^1, H-b^2, H-B), 1.47-1.35 (m, 20H, H-c^1, H-c^2, H-C), 1.32-1.25 (m, 28H, H-d^2, H-e, H-f, H-j, H-h, H-i, H-j, H-k, H-l, H-m, H-n, H-o, H-p, H-q), 1.02 (t, $^3J_{HH}$= 7.4 Hz, 24 H, H-D), 0.95-0.87 (m, 6H, H-d^1, H-r), 0.86-0.80 (m, 2H, H-11$_a$), 0.63-0.56 (m, 2H, H-11$_b$). **^{13}C NMR (101 MHz, CD_3OD):** δ [ppm] = 161.8 (C-2), 157.4 (C-6), 151.6 (C-4), 139.5 (C-3´), 137.5 (C-8), 130.7 (C-2´), 114.7 (C-5), 69.7 (d, $^3J_{CP}$= 6.2 Hz, C-6´), 69.4, 66.1 (2 x d, $^3J_{CP}$= 6.3 Hz, $^3J_{CP}$= 6.3 Hz, C-a^1, C-a^2), 60.4 (C-1´), 59.4 (t, $^3J_{CP}$= 2.7 Hz, C-A), 47.6 (d, $^3J_{CP}$= 8.5 Hz, C-4´), 36.1 (C-5´), 33.4 (d, $^3J_{CP}$= 7.5 Hz, C-b^1), 31.3 (t, $^3J_{CP}$= 7.5 Hz, C-b^2), 33.1, 30.78, 30.75, 30.72, 30.5, 30.4, 23.7 (C-d^2, C-e, C-f, C-g, C-h, C-i, C-j, C-k, C-l, C-m, C-n, C-o, C-p, C-q) 26.7 (C-c^2), 24.8 (C-B), 24.3 (C-10), 20.7 (t, $^4J_{CP}$= 1.3 Hz, C-C), 19.8 (C-c^1), 14.5 (C-r), 14.1 (C-d^1), 13.96 (C-D), 7.6 (C-11). **^{31}P NMR (162 MHz, CD_3OD):** δ [ppm] = -11.7 (d, $^2J_{pp}$= 23.5 Hz, P-γ), -13.2 (d, $^2J_{pp}$= 17.6 Hz, P-α), -24.7 (t, $^2J_{pp}$= 19.1 Hz, P-β). **HRMS (ESI$^-$, m/z):** calculated for $C_{36}H_{65}N_6O_{10}P_3$ $[M-H]^-$ 833.3902; found, 833.3940.

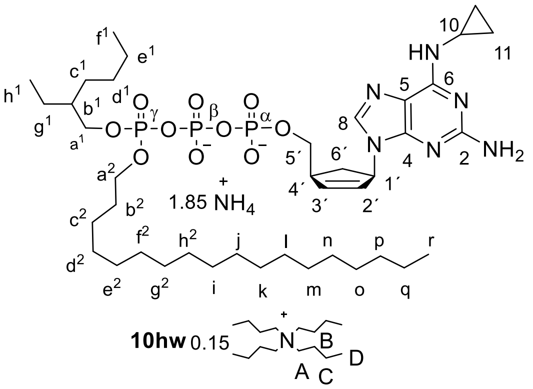

γ-(C8;C18)-ABCTP 10hw. According to general procedure 2 with 134 mg *H*-phosphonate **15cb** (0.3 mmol, 1.0 equiv.), 80 mg NCS (0.6 mmol, 2.0 equiv.), and 178 mg (n-$Bu_4N)_3$·ABCMP (0.21 mmol, 0.7 equiv.). Reaction time was 3 h. Yield: 66 mg (0.06 mmol, 30%) yellow solid. HPLC-UV analysis confirmed purity: > 95%. **^{1}H NMR (400 MHz, CD_3OD):** δ [ppm] = 7.97 (s, 1H, H-8), 6.30-6.17 (m, 1H, H-3´), 5.86-5.75 (m, 1H, H-2´), 5.57-5.46 (m, 1H, H-1´), 4.25-4.05 (m, 6H, H-6´, H-a^1, H-a^2), 3.25-3.20 (m, 1.2H, H-A), 3.18-3.10 (m, 1H, H-4´), 3.10-2.86 (m, 1H, H-10), 2.80-2.68 (m, 1H, H-5´$_a$), 1.98-1.82 (m, 2H, H-5´$_b$), 1.74-1.62 (m, 3.2H, H-B, H-b^2), 1.60-1.52 (m, 1H, H-b^1), 1.46-1.25 (m, 38H, H-C, H-c^1, H-c^2, H-d^1, H-d^2, H-e^1, H-e^2, H-f^2, H-g^1, H-g^2, H-h^2, H-i, H-j, H-k, H-l, H-m, H-n, H-o, H-p, H-q), 1.03 (t, $^3J_{HH}$= 7.4 Hz, 1.8H, H-D), 0.95-0.82 (m, 11H, H-h^1, H-f^1, H-r, H-11$_a$), 0.82-0.65 (m, 2H, H-11$_b$). **^{13}C NMR (101 MHz, CD_3OD):** δ [ppm] = 161.0 (C-2), 156.8 (C-6), 151.6 (C-4), 138.7 (C-3´), 137.6 (C-8), 131.0 (C-2´), 114.5 (C-5), 69.7 (d, $^3J_{CP}$= 6.2 Hz, C-6´), 69.3 (dd, $^2J_{CP}$= 24.1 Hz, $^3J_{CP}$= 6.7 Hz, C-a^1), 66.1 (dd, $^2J_{CP}$= 21.7 Hz, $^3J_{CP}$= 6.1 Hz, C-a^2), 60.5 (C-1´), 59.4 (t, $^3J_{CP}$= 2.8 Hz, C-A), 47.3 (d, $^3J_{CP}$= 8.5 Hz, C-4´), 35.7 (C-5´), 41.0 (C-b^1), 34.08, 33.98, 33.38, 33.28, 33.1, 31.95, 31.85, 31.33, 31.24, 30.79, 30.70, 30.67, 30.5, 30.3, 27.0, 23.7, 20.1 (C-d^1, C-d^2, C-e^1, C-e^2, C-f^2, C-g^1, C-g^2, C-h^2, C-i, C-j, C-k, C-l, C-m, C-n, C-o, C-p, C-q), 26.6 (C-c^2), 24.8 (C-B), 24.4 (C-10), 20.7 (t, $^4J_{CP}$= 1.3 Hz, C-C), 19.7 (C-c^1), 14.5 (C-r), 14.2 (C-f^1), 13.99 (C-D), 7.7 (C-11). **^{31}P NMR (162 MHz, CD_3OD):** δ [ppm] = -11.0 (d,

$^{2}J_{pp}$= 17.8 Hz, P-γ), -12.6 (d, $^{2}J_{pp}$= 15.6 Hz, P-α), -23.67 (t, $^{2}J_{pp}$= 16.0 Hz, P-β). **HRMS (ESI^{-}, m/z):** calculated for $C_{40}H_{73}N_6O_{10}P_3$ [M-H]$^{-}$ 889.4528; found, 889.4523.

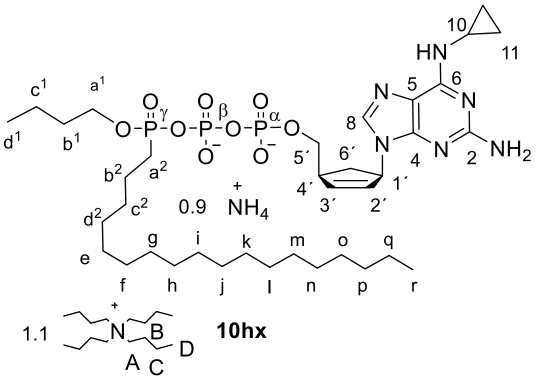

γ-(C4)-γ-C-(C18)-ABCTP 10hx. According to general procedure 2 with 112 mg *H*-phosphinate **15d** (0.3 mmol, 1.0 equiv.), 80 mg NCS (0.6 mmol, 2.0 equiv.), and 178 mg (*n*-$Bu_4N)_3$·ABCMP (0.21 mmol, 0.7 equiv.). Reaction time was 3 h. Yield: 60 mg (0.05 mmol, 26%) yellow solid. HPLC-UV analysis confirmed purity: > 99%. **^{1}H NMR (400 MHz, CD_3OD):** δ [ppm] = 7.80 (s, 1H, H-8), 6.30-6.17 (m, 1H, H-3´), 5.96-5.85 (m, 1H, H-2´), 5.60-5.46 (m, 1H, H-1´), 4.20-4.05 (m, 4H, H-6´, H-a^{1}), 3.29-3.20 (m, 8.8H, H-A), 3.19-3.10 (m, 1H, H-4´), 3.05-2.86 (m, 1H, H-10), 2.86-2.72 (m, 1H, H-5´$_{a}$), 2.06-1.85 (m, 2H, H-a^{2}), 1.85-1.72 (m, 2H, H-5´$_{b}$), 1.74-1.58 (m, 12.8H, H-B, H-b^{1}, H-b^{2}), 1.47-1.35 (m, 12.8H, H-C, H-c^{1}, H-c^{2}), 1.36-1.25 (m, 28H, H-d^{2}, H-e, H-f, H-g, H-h, H-i, H-j, H-k, H-l, H-m, H-n, H-o, H-p, H-q), 1.02 (t, $^{3}J_{HH}$= 7.4 Hz, 13.6H, H-D), 0.95-0.82 (m, 8H, H-d^{1}, H-r, H-11$_{a}$), 0.65-0.57 (m, 2H, H-11$_{b}$). **^{13}C NMR (101 MHz, CD_3OD):** δ [ppm] = 161.4 (C-2), 157.2 (C-6), 151.6 (C-4), 138.7 (C-3´), 137.4 (C-8), 131.3 (C-2´), 114.7 (C-5), 69.6 (dd, $^{3}J_{CP}$= 6.2 Hz, $^{3}J_{CP}$= 3.4 Hz, C-6´), 66.7 (dd, $^{3}J_{CP}$= 7.4 Hz, $^{4}J_{CP}$= 1.4 Hz, C-a^{1}), 60.4 (C-1´), 59.4 (t, $^{3}J_{CP}$= 2.8 Hz, C-A), 47.4 (d, $^{3}J_{CP}$= 8.7 Hz, C-4´), 35.7 (C-5´), 33.5 (d, $^{3}J_{CP}$= 6.8 Hz, C-b^{1}), 33.1, 31.65, 31.4, 30.79, 30.71, 30.52, 30.48, 30.2, 23.7 (C-d^{1}, C-d^{2}, C-e^{1}, C-e^{2}, C-f^{2}, C-g^{1}, C-g^{2}, C-h^{2}, C-i, C-j, C-k, C-l, C-m, C-n, C-o, C-p, C-q), 27.9, 26.0 (C-a^{2}), 24.3 (C-10), 23.3 (d, $^{3}J_{CP}$= 5.3 Hz, C-b^{2}), 24.8 (C-B), 20.7 (t, $^{4}J_{CP}$= 1.3 Hz, C-C), 19.8 (C-c^{1}), 14.5 (C-r), 14.02 (C-d^{1}), 13.98 (C-D), 7.6 (C-11). **^{31}P NMR (162 MHz, CD_3OD):** δ [ppm] = 24.4 (d, $^{2}J_{pp}$= 23.3 Hz, P-γ), -11.6 (d, $^{2}J_{pp}$= 20.6 Hz, P-α), -23.4 (t, $^{2}J_{pp}$= 22.1 Hz, P-β). **HRMS (ESI^{-}, m/z):** calculated for $C_{36}H_{65}N_6O_9P_3$ [M-H]$^{-}$ 817.3953; found, 817.3926.

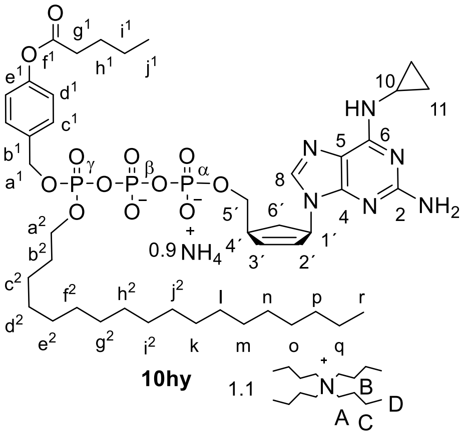

γ-(AB-C4;C18)-ABCTP 10hy. According to general procedure 2 with 157 mg *H*-phosphonate **15a** (0.3 mmol, 1.0 equiv.), 80 mg NCS (0.6 mmol, 2.0 equiv.), and 178 mg (*n*-$Bu_4N)_3$·ABCMP (0.21 mmol, 0.7 equiv.). Reaction time was 3 h. Yield: 189 mg (0.15 mmol, 72%) yellow solid. HPLC-UV analysis confirmed purity: > 95%. **^{1}H-NMR (500 MHz, CD_3OD):** δ [ppm] = 7.79 (d, $^{3}J_{HH}$= 2.8 Hz, 1H, H-8), 7.47-7.42 (m, 2H, H-c^{1}), 7.08-7.02 (m, 2H, H-d^{1}), 6.25-6.15 (m, 1H, H-3´), 5.88-5.82 (m, 1H, H-2´), 5.60-5.47 (m, 1H, H-1´), 5.12 (dd, $^{3}J_{HH}$= 8.3 Hz, $^{3}J_{HH}$= 2.6 Hz, 1H, H-a^{1}), 4.15-3.90 (m, 4H, H-6´, H-a^{2}), 3.29-3.20 (m, 10H, H-A), 3.19-3.05 (m, 1H, H-4´), 2.95-2.86 (m, 1H, H-10), 2.86-2.72 (m, 1H, H-5´$_{a}$), 2.60-2.55 (m, 3H, H-g^{1}), 1.80-1.55 (m, 16H, H-5´$_{b}$, H-B, H-h^{1}, H-b^{2}), 1.49-1.34 (m, 14H, H-C, H-i^{1}, H-c^{2}), 1.34-1.24 (m, 28H, H-d^{2}, H-e^{2}, H-f^{2}, H-g^{2}, H-h^{2}, H-i^{2}, H-j^{2}, H-k, H-l, H-m, H-n, H-o, H-p, H-q), 1.05-0.94 (m, 18H, H-j^{1}, H-D), 0.95-0.82 (m, 5H, H-r, H-11$_{a}$), 0.65-0.57 (m, 2H, H-11$_{b}$). **^{13}C-NMR (126 MHz, CD_3OD):** δ [ppm] = 173.5 (d, $^{2}J_{CP}$= 15.0 Hz, C-f^{1}), 156.8 (C-6), 152.3 (C-e^{1}), 151.6 (C-4), 138.7 (C-3´), 137.5 (d, $^{3}J_{CP}$= 7.7 Hz, C-8), 135.1 (d, $^{3}J_{CP}$= 7.4 Hz, C-b^{1}), 131.2 (C-2´),

130.3, 129.4 (C-c[1]), 122.8, 122.5 (C-d[1]), 114.5 (C-5), 70.0 (d, $^{3}J_{CP}$= 5.6 Hz, C-6´), 69.6 (dd, $^{2}J_{CP}$= 17.1 Hz, $^{3}J_{CP}$= 7.0 Hz, C-a[1]), 66.5 (C-a[2]), 60.5 (C-1´), 59.4 (t, $^{3}J_{CP}$= 2.8 Hz, C-A), 47.3 (d, $^{3}J_{CP}$= 8.8 Hz, C-4´), 35.6 (C-5´), 34.7 (C-g[2]), 33.1, 31.3, 31.2, 30.80, 30.75, 30.71, 30.66, 30.5, 30.3, 26.9, 23.7 (C-d[2], C-e[2], C-f[2], C-g[2], C-h[2], C-i[2], C-j[2], C-k, C-l, C-m, C-n, C-o, C-p, C-q), 28.0 (C-h[1]), 26.5 (C-c[2]), 24.7 (C-B, C-10), 23.2 (C-i[1]), 20.7 (C-C), 14.5 (C-r), 14.1 (C-j[1]), 14.0 (C-D), 7.7 (C-11). **^{31}P NMR (162 MHz, CD_3OD):** δ [ppm] = -11.02 (d, $^{2}J_{pp}$= 19.3 Hz, P-γ), -13.06 (d, $^{2}J_{pp}$= 17.6 Hz, $^{3}J_{pp}$= 3.9 Hz, P-α), -23.80 (t, $^{2}J_{pp}$= 17.8 Hz, P-β). **MALDI-MS (m/z):** calculated for $C_{44}H_{71}N_6O_{12}P_3$ $[M-H]^-$ 967.427; found, 967.390.

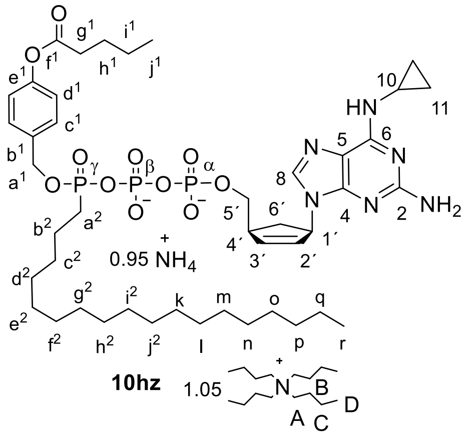

10hz

γ-(AB-C4)-γ-C-(C18)-ABCTP 10hz. According to general procedure 2 with 153 mg *H*-phosphonate **15b** (0.3 mmol, 1.0 equiv.), 80 mg NCS (0.6 mmol, 2.0 equiv.), and 178 mg (n-$Bu_4N)_3$·ABCMP (0.21 mmol, 0.7 equiv.). Reaction time was 3 h. Yield: 54 mg (0.04 mmol, 21%) yellow solid. HPLC-UV analysis confirmed purity: > 99%. **^{1}H-NMR (500 MHz, CD_3OD):** δ [ppm] = 7.92 (s, 1H, H-8), 7.48-7.43 (m, 2H, H-c[1]), 7.06-7.01 (m, 2H, H-d[1]), 6.27-6.11 (m, 1H, H-3´), 5.82-5.72 (m, 1H, H-2´), 5.60-5.47 (m, 1H, H-1´), 5.25-5.15 (m, 2H, H-a[1]), 4.25-4.05 (m, 2H, H-6´), 3.29-3.20 (m, 8.4H, H-A), 3.19-2.85 (m, 1H, H-4´, H-10), 2.76-2.65 (m, 1H, H-5´$_a$), 2.56 (t, $^{3}J_{CP}$= 7.4 Hz, 3H, H-g[1]), 2.22-1.96 (m, 2H, H-a[2]), 1.93-1.82 (m, 1H, H-5´$_b$), 1.75-1.55 (m, 12.5H, H-B, H-h[1], H-b[2]), 1.49-1.36 (m, 10.5H, H-C, H-i[1]), 1.34-1.24 (m, 30H, H-c[2], H-d[2], H-e[2], H-f[2], H-g[2], H-h[2], H-i[2], H-j[2], H-k, H-l, H-m, H-n, H-o, H-p, H-q), 1.03 (t, $^{3}J_{HH}$= 7.4 Hz, 12.6H, H-D), 0.98 (t, $^{3}J_{HH}$= 7.4 Hz, 3H, H-j[1]), 0.93-0.76 (m, 5H, H-r, H-11$_a$), 0.75-0.57 (m, 2H, H-11$_b$). **^{13}C-NMR (126 MHz, CD_3OD):** δ [ppm] = 173.5 (C-f[1]), 161.5 (C-2), 157.1 (C-6), 152.1 (C-e[1]), 151.6 (C-4), 138.7 (C-3´), 137.4 (C-8), 135.5 (d, $^{3}J_{CP}$= 6.8 Hz, C-b[1]), 131.2 (C-2´), 130.2 (C-c[1]), 122.8 (C-d[1]), 114.6 (C-5), 69.6 (d, $^{3}J_{CP}$= 2.7 Hz, C-6´), 67.7 (d, $^{3}J_{CP}$= 6.5 Hz, C-a[1]), 60.4 (C-1´), 59.5 (t, $^{3}J_{CP}$= 2.8 Hz, C-A), 47.6 (d, $^{3}J_{CP}$= 8.6 Hz, C-4´), 35.6 (C-5´), 34.7 (C-g[1]), 33.0, 31.6, 31.4, 30.79, 30.70, 30.49, 30.46, 30.2, 23.7 (C-d[2], C-e[2], C-f[2], C-g[2], C-h[2], C-i[2], C-j[2], C-k, C-l, C-m, C-n, C-o, C-p, C-q), 28.0 (C-h[1]), 26.2 (C-a[2]), 24.7 (C-B), 24.3 (C-10), 23.28 (t, $^{3}J_{CP}$= 5.4 Hz, C-b[2]), 23.21 (C-i[1]), 20.7 (C-C), 14.5 (C-r), 14.2 (C-j[1]), 14.0 (C-D), 7.6 (C-11). **^{31}P NMR (162 MHz, CD_3OD):** δ [ppm] = 24.1 (d, $^{2}J_{pp}$= 23.6 Hz, P-γ), -10.95 (d, $^{2}J_{pp}$= 20.6 Hz, P-α), -23.93 (t, $^{2}J_{pp}$= 21.5 Hz, P-β). **HRMS (ESI^-, m/z):** calculated for $C_{44}H_{71}N_6O_{11}P_3$ $[M-H]^-$ 951.4321; found, 951.4317.

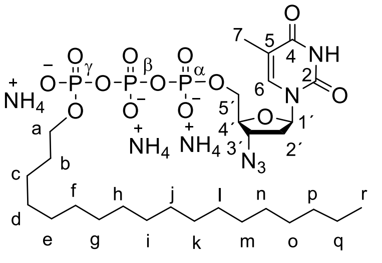

20b

γ-C18-AZTTP 20b. According to general procedure 2 with 116 mg *H*-phosphonate **17a** (0.3 mmol, 1.0 equiv.), 80 mg NCS (0.6 mmol, 2.0 equiv.), 2.3 mL of tetrabutylammonium phosphate (0.9 mmol, 3.0 equiv), 174 mg (n-$Bu_4N)_2$·AZTMP salt (0.21 mmol, 0.7 equiv.), and 0.97 mL 40% $nBu_4N^+OH^-$ in H_2O (1.50 mmol, 5 equiv). Reaction time was 8 h. The crude product was purified by automatic RP18 flash chromatography. The mixture following a Dowex 50WX8 (NH_4^+) ion exchange, freeze-drying, and purified by automatic RP18 flash chromatography again. The desired products were obtained as solids after freeze-drying again. Yield: 73 mg (0.09 mmol, 43%) white solid. HPLC-UV analysis confirmed purity: > 97%.

^{1}H NMR (400 MHz, CD_3OD): δ [ppm] = 7.82 (d, $^{4}J_{HH}$= 1.2 Hz, 1H, H-6), 6.24 (dd, $^{3}J_{HH}$= 8.2 Hz, $^{3}J_{HH}$= 5.8 Hz, 1H, H-1´), 4.66-4.58 (m, 1H, H-3´), 4.28-4.15 (m, 2H, H-5´), 4.12-4.06 (m, 2H, H-4´), 3.99 (q, $^{3}J_{HH}$= 6.7 Hz, 2H, H-a), 2.54-2.40 (m, 1H, H-2´$_{a}$), 2.38-2.26 (m, 1H, H-2´$_{b}$), 1.94 (d, $^{4}J_{HH}$= 1.1 Hz, 3H, H-7), 1.65 (quint, $^{3}J_{HH}$= 6.7 Hz, 2H, H-b), 1.45-1.23 (m, 30H, H-c, H-d, H-e, H-f, H-g, H-h, H-i, H-j, H-k, H-l, H-m, H-n, H-o, H-p, H-q), 0.90 (t, $^{3}J_{HH}$= 6.8 Hz, 3H, H-r). **^{13}C NMR (101 MHz, CD_3OD):** δ [ppm] = 166.5 (C-4), 152.4 (C-2), 138.0 (C-6), 112.2 (C-5), 86.0 (C-1´), 84.7 (d, $^{3}J_{CP}$= 9.6 Hz, C-4´), 67.4 (d, $^{3}J_{CP}$= 6.5 Hz, C-a), 67.1 ($^{3}J_{CP}$= 5.7 Hz, C-5´), 63.2 (C-3´), 37.9 (C-2´), 31.8 (t, $^{3}J_{CP}$= 8.1 Hz, C-b), 33.1, 30.79, 30.75, 30.6, 30.5, 23.7 (C-d, C-e, C-f, C-g, C-h, C-i, C-j, C-k, C-l, C-m, C-n, C-o, C-p, C-q), 26.9 (C-c), 14.4 (C-r), 12.6 (C-7). **^{31}P NMR (162 MHz, CD_3OD):** δ [ppm] = -10.72 (d, $^{2}J_{pp}$=17.6 Hz, P-γ), -11.55 (d, $^{2}J_{pp}$=19.6 Hz, P-α), -22.29 (t, $^{2}J_{pp}$= 18.6 Hz, P-β). **MALDI-MS (m/z):** calculated for $C_{28}H_{52}N_5O_{13}P_3$ $[M-H]^-$ 758.2702; found, 758.1818.

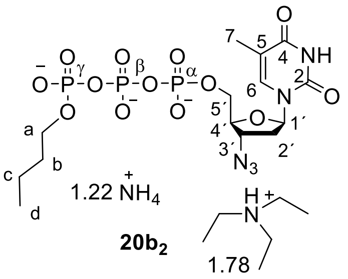

γ-C4-AZTTP 20b$_2$. According to general procedure 2 with 58 mg *H*-phosphonate **17b** (0.3 mmol, 1.0 equiv.), 80 mg NCS (0.6 mmol, 2.0 equiv.), 174 mg (n-$Bu_4N)_2$·AZTMP salt (0.21 mmol, 0.7 equiv.), and 0.97 mL 40% $n$$Bu_4N^+OH^-$ in H_2O (1.50 mmol, 5 equiv). Reaction time was 8 h. Yield: 48 mg (0.08 mmol, 37%) white solid. HPLC-UV analysis confirmed purity: > 98%. **^{1}H NMR (400 MHz, D_2O):** δ [ppm] = 7.80 (d, $^{4}J_{HH}$= 1.2 Hz, 1H, H-6), 6.31 (t, $^{3}J_{HH}$= 6.7 Hz, 1H, H-1´), 4.62-4.55 (m, 1H, H-3´), 4.28-4.15 (m, 3H, H-4´, H-5´), 3.96 (q, $^{3}J_{HH}$= 6.8 Hz, 2H, H-a), 3.23 (q, $^{3}J_{HH}$= 7.3 Hz, 10.68H, $HN(CH_2CH_3)_3^+$), 2.58-2.45 (m, 2H, H-2´), 1.96 (d, $^{4}J_{HH}$= 1.1 Hz, 3H, H-7), 1.65 (quint, $^{3}J_{HH}$= 6.8 Hz, 2H, H-b), 1.37 (sex, $^{3}J_{HH}$= 7.5 Hz, 2H, H-c), 1.31 (q, $^{3}J_{HH}$= 7.3 Hz, 16.02H, $HN(CH_2CH_3)_3^+$), 0.90 (t, $^{3}J_{HH}$= 7.5 Hz, 3H, H-d). **^{13}C NMR (101 MHz, D_2O):** δ [ppm] = 166.5 (C-4), 151.7 (C-2), 137.4 (C-6), 111.8 (C-5), 84.8 (C-1´), 83.0 (d, $^{3}J_{CP}$= 9.4 Hz, C-4´), 66.5 (d, $^{3}J_{CP}$= 6.2 Hz, C-a), 65.5 (C-5´), 60.9 (C-3´), 46.7 ($HN(CH_2CH_3)_3^+$), 36.3 (C-2´), 31.9 (d, $^{3}J_{CP}$= 7.1 Hz, C-b), 18.3 (C-c), 13.0 (C-d), 11.6 (C-7), 8.2 ($HN(CH_2CH_3)_3^+$). **^{31}P NMR (162 MHz, D_2O):** δ [ppm] = -9.75 (d, $^{2}J_{pp}$=20.6 Hz, P-γ), -10.68 (d, $^{2}J_{pp}$=20.6 Hz, P-α), -22.23 (t, $^{2}J_{pp}$= 19.3 Hz, P-β). **HRMS (ESI$^-$, m/z):** calculated for $C_{14}H_{24}N_5O_{13}P_3$ $[M-H]^-$ 562.0511; found, 562.0350.

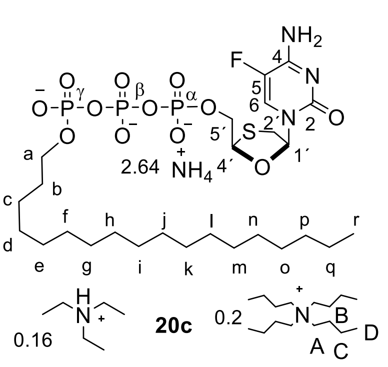

γ-C18-FTCTP 20c. According to general procedure 2 with 116 mg *H*-phosphonate **17a** (0.3 mmol, 1.0 equiv.), 80 mg NCS (0.6 mmol, 2.0 equiv.), 2.3 mL of tetrabutylammonium phosphate (0.9 mmol, 3.0 equiv), 170 mg (n-$Bu_4N)_2$·FTCMP (0.21 mmol, 0.7 equiv.), and 0.97 mL 40% $n$$Bu_4N^+OH^-$ in H_2O (1.50 mmol, 5 equiv). Reaction time was 8 h. The mixture following a Dowex 50WX8 (NH_4^+) ion exchange, freeze-drying, and purified by automatic RP18 flash chromatography again. The desired products were obtained as solids after freeze-drying again. Yield: 68 mg (0.08 mmol, 38%) white solid. HPLC-UV analysis confirmed purity: > 97%. **^{1}H-NMR (600 MHz, CD_3OD):** δ [ppm] = 8.16 (d, $^{3}J_{HH}$= 6.5 Hz, 1H, H-6), 6.21 (dt, $^{3}J_{HH}$= 5.1 Hz, $^{3}J_{HH}$= 1.6 Hz, 1H, H-1´), 5.46-5.42 (m, 1H, H-4´), 4.45-4.32 (m, 2H, H-5´), 4.01 (quint, $^{3}J_{HH}$= 6.6 Hz, 2H, H-a), 3.49 (dd, $^{3}J_{HH}$= 7.2 Hz, 1H, H-2´$_{a}$), 3.28-3.21 (m, 1.6H, H-A), 3.20 (quint, $^{3}J_{HH}$= 6.6 Hz, 0.96H, $HN(CH_2CH_3)_3^+$), 3.15 (dd, $^{3}J_{HH}$= 12.1 Hz, $^{3}J_{HH}$= 4.7 Hz, 1H, H-2´$_{b}$), 1.72-1.60 (m, 3.6H, H-b, H-B), 1.47-1.35 (m, 3.6H, H-c, H-

C), 1.35-1.25 (m, 29.5H, , $HN(CH_2CH_3)_3^+$, H-d, H-e, H-f, H-g, H-h, H-i, H-j, H-k, H-l, H-m, H-n, H-o, H-p, H-q), 1.03 (t, $^3J_{HH}$= 7.4 Hz, 2.4H, H-D), 0.90 (t, $^3J_{HH}$= 7.0 Hz, 3H, H-r). **^{13}C-NMR (151 MHz, CD_3OD):** δ [ppm] = 156.0 (d, $^2J_{CP}$= 15.5 Hz, C-4), 150.0 (C-2), 137.4 (C-5), 127.2, 127.0 (C-6), 89.2 (C-1´), 85.7 (d, $^3J_{CP}$= 9.5 Hz, C-4´), 68.1 (d, $^3J_{CP}$= 5.5 Hz, C-a), 67.6 (d, $^3J_{CP}$= 6.3 Hz, C-5´), 47.5 ($HN(CH_2CH_3)_3^+$), 37.9 (C-2´), 31.8 (d, $^3J_{CP}$= 8.0 Hz, C-b), 33.1, 30.80, 30.79, 30.76, 30.6, 30.5, 23.7, 17.4, 17.3 (C-d, C-e, C-f, C-g, C-h, C-i, C-j, C-k, C-l, C-m, C-n, C-o, C-p, C-q), 26.9 (C-c), 24.8 (C-B), 20.8 (C-C), 14.4 (C-r), 13.9 (C-D), 11.5, 9.1 ($HN(CH_2CH_3)_3^+$). **^{31}P NMR (162 MHz, CD_3OD):** δ [ppm] = -11.02 (d, $^2J_{pp}$= 20.6 Hz, P-α), -11.71 (d, $^2J_{pp}$= 17.7 Hz, $^3J_{pp}$=5.8 Hz, P-γ), -22.80 (t, $^2J_{pp}$= 19.1 Hz, P-β). **MALDI-MS (m/z):** calculated for $C_{26}H_{49}FN_3O_{12}SP_3$ $[M-H]^-$ 738.2161; found, 738.2245. **^{19}F-NMR (188 MHz, CD_3OD):** δ [ppm] = -167.5 -- -167.7.

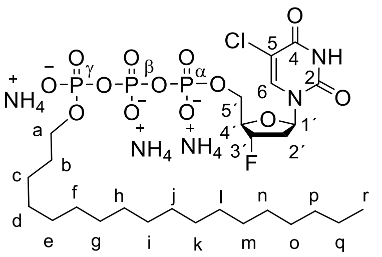

20d

γ-C18-Fdd(Cl)UTP 20d. According to general procedure 2 with 116 mg *H*-phosphonate **17a** (0.3 mmol, 1.0 equiv.), 80 mg NCS (0.6 mmol, 2.0 equiv.), 2.3 mL of tetrabutylammonium phosphate (0.9 mmol, 3.0 equiv), 174 mg ($(n\text{-}Bu_4N)_2$·FddClUMP (0.21 mmol, 0.7 equiv.), and 0.97 mL 40% $nBu_4N^+OH^-$ in H_2O (1.50 mmol, 5 equiv). Reaction time was 8 h. The mixture following a Dowex 50WX8 (NH_4^+) ion exchange, freeze-drying, and purified by automatic RP18 flash chromatography again. The desired products were obtained as solids after freeze-drying again. Yield: 66 mg (0.08 mmol, 39%) white solid. HPLC-UV analysis confirmed purity: > 97%. **^{1}H NMR (400 MHz, CD_3OD):** δ [ppm] = 8.17 (s, 1H, H-6), 6.36 (dd, $^3J_{HH}$= 9.3 Hz, $^3J_{HH}$= 5.5 Hz, 1H, H-1´), 5.41 (dd, $^2J_{HH}$= 53.1 Hz, $^3J_{HH}$= 4.2 Hz, 1H, H-3´), 4.47-4.34 (m, 1H, H-4´), 4.34-4.10 (m, 2H, H-5´), 3.99 (q, $^3J_{HH}$= 6.7 Hz, H-a), 2.4-22.26 (m, 2H, H-2´), 1.65 (q, $^3J_{HH}$= 6.7 Hz, H-b), 1.32-1.25 (m, 30H, H-c, H-d, H-e, H-f, H-j, H-h, H-i, H-j, H-k, H-l, H-m, H-n, H-o, H-p, H-q), 0.90 (d, $^3J_{HH}$= 7.8 Hz, 3H, H-r). **^{13}C NMR (101 MHz, CD_3OD):** δ [ppm] = 161.1 (C-4), 151.4 (C-2), 139.0 (C-6), 110.0 (C-5), 95.80 (d, $^1J_{CF}$= 175.3 Hz, C-3´), 87.0 (C-1´), 85.6 (dd, $^2J_{CP}$= 25.4 Hz, $^3J_{CP}$= 9.2 Hz, C-4´), 67.5 (d, $^3J_{CP}$= 6.2 Hz, C-a), 66.5 (dd, $^2J_{CP}$= 11.4 Hz, $^3J_{CP}$= 5.4 Hz, C-5´), 39.1 (d, $^2J_{CP}$= 20.8 Hz, C-2´), 31.8 (d, $^3J_{CP}$= 7.9 Hz, C-b), 33.1, 30.79, 30.75, 30.6, 30.5, 23.7 (C-d, C-e, C-f, C-g, C-h, C-i, C-j, C-k, C-l, C-m, C-n, C-o, C-p, C-q) 26.9 (C-c), 14.4 (C-r). **^{31}P NMR (162 MHz, CD_3OD):** δ [ppm] = -10.72 (d, $^2J_{pp}$= 17.6 Hz, P-γ), -12.60 (d, $^2J_{pp}$= 20.3 Hz, P-α), -22.23 (t, $^2J_{pp}$= 17.6 Hz, P-β). **MALDI-MS (m/z):** calculated for $C_{27}H_{49}FClN_2O_{13}P_3$ $[M-H]^-$ 755.2047; found,755.2660. **^{19}F-NMR (188 MHz, CD_3OD):** δ [ppm] = -175.0 -- -176.5.

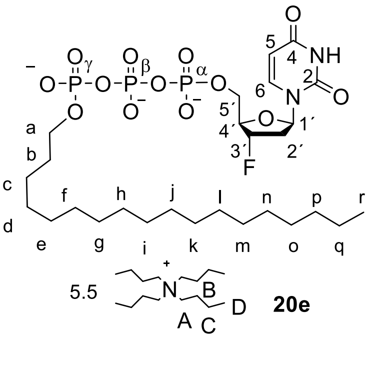

20e

γ-C18-FddUTP 20e. According to general procedure 1 with 116 mg *H*-phosphonate **17a** (0.3 mmol, 1.0 equiv.), 40 mg NCS (0.3 mmol, 1.0 equiv.), 233 mg ($(n\text{-}Bu_4N)_3$·FddUDP (0.21 mmol, 0.7 equiv.), and 0.97 mL 40% $nBu_4N^+OH^-$ in H_2O (1.50 mmol, 5 equiv). Reaction time was 12 h. The mixture following a Dowex 50WX8 (NH_4^+) ion exchange, freeze-drying, and purified by automatic RP18 flash chromatography again. The desired products were obtained as solids after freeze-drying again. Yield: 138 mg (0.07 mmol, 32%) white solid. HPLC-UV analysis confirmed purity: > 98%. **^{1}H NMR (400 MHz, CD_3OD):** δ [ppm] = 8.56 (s, 1H,

NH), 7.82 (d, ${}^3J_{HH}$= 7.6 Hz, 1H, H-6), 6.49 (dd, ${}^3J_{HH}$= 9.8 Hz, ${}^3J_{HH}$= 5.6 Hz, 1H, H-1´), 5.76 (d, ${}^3J_{HH}$= 7.7 Hz, 1H, H-5), 5.58 (dd, ${}^2J_{HH}$= 53.3 Hz, ${}^3J_{HH}$= 3.5 Hz, 1H, H-3´), 4.43-4.10 (m, 3H, H-4´, H-5´), 4.00 (q, ${}^3J_{HH}$= 6.5 Hz, H-a), 3.28-3.19 (m, 44H, H-A), 2.54-2.20 (m, 2H, H-2´), 1.74-1.60 (m, 46H, H-B, H-b), 1.46-1.37 (m, 46H, H-C, H-c), 1.33-1.26 (m, 28H, H-d, H-e, H-f, H-j, H-h, H-i, H-j, H-k, H-l, H-m, H-n, H-o, H-p, H-q), 1.02 (t, ${}^3J_{HH}$= 7.3 Hz, 66H, H-D), 0.90 (t, ${}^3J_{HH}$= 7.0 Hz, 3H, H-r). **^{13}C-NMR (151 MHz, CD_3OD):** δ [ppm] = 170.2 (C-4), 159.7 (C-2), 140.6 (C-6), 104.3 (C-5), 96.6 (d,${}^1J_{CF}$= 175.2 Hz, C-3´), 86.3 (C-1´), 85.1 (dd, ${}^3J_{CP}$= 25.0 Hz, ${}^3J_{CP}$= 9.8 Hz, C-4´), 67.05 (d, ${}^3J_{CP}$= 6.2 Hz, C-a), 66.5 (C-5´), 59.5 (t, ${}^3J_{CP}$= 2.6 Hz, C-A), 39.1 (d, ${}^2J_{CF}$= 20.1 Hz, C-2´), 31.9 (d, ${}^3J_{CP}$= 7.3 Hz, C-b), 33.1, 30.84, 30.79, 30.75, 30.70, 30.5, 23.7 (C-d, C-e, C-f, C-g, C-h, C-i, C-j, C-k, C-l, C-m, C-n, C-o, C-p, C-q), 27.0 (C-c), 24.8 (C-B), 20.7 (t, ${}^4J_{CP}$= 1.4 Hz, C-C), 14.4 (C-r), 13.9 (C-D). **^{31}P NMR (162 MHz, CD_3OD):** δ [ppm] = -10.32 (d, ${}^2J_{pp}$= 17.6 Hz, P-γ), -11.18 (d, ${}^2J_{pp}$= 17.7 Hz, P-α), -21.89 (t, ${}^2J_{pp}$= 19.1 Hz, P-β). **MALDI-MS (m/z):** calculated for $C_{27}H_{50}FN_2O_{13}P_3$ [M-H]$^-$ 721.2437; found, 721.2052. **^{19}F-NMR (188 MHz, CD_3OD):** δ [ppm] = -175.3 - -176.0.

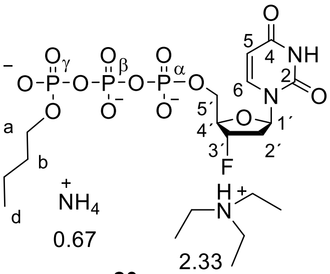

20e$_2$

γ-C4-FddUTP 20e$_2$. According to general procedure 2 with 95 mg *H*-phosphonate **17b** (0.3 mmol, 1.0 equiv.), 80 mg NCS (0.6 mmol, 2.0 equiv.), 167 mg FddUMP 2×nBu$_4$N$^+$ salt (0.21 mmol, 0.7 equiv), and 0.97 mL 40% *n*Bu$_4$N$^+$OH$^-$ in H_2O (1.50 mmol, 5 equiv). Reaction time was 8 h. Yield: 41 mg (0.05 mmol, 25%) white solid. HPLC-UV analysis confirmed purity: > 97%. **^{1}H NMR (600 MHz, D_2O):** δ [ppm] = 8.01 (d, ${}^3J_{HH}$= 8.2 Hz, 1H, H-6), 6.42 (dd, ${}^3J_{HH}$= 9.4 Hz, ${}^3J_{HH}$= 5.6 Hz, 1H, H-1´), 5.99 (d, ${}^3J_{HH}$= 8.2 Hz, 1H, H-5), 5.58 (dd, ${}^2J_{HH}$= 52.6 Hz, ${}^3J_{HH}$= 4.3 Hz, 1H, H-3´), 4.62-4.58 (m, 1H, H-4´), 4.30-4.07 (m, 2H, H-5´), 3.97 (q, ${}^3J_{HH}$= 6.8 Hz, H-a), 3.20 (q, ${}^3J_{HH}$= 7.4 Hz, 14H, $HN(CH_2CH_3)_3^+$), 2.70-2.35 (m, 2H, H-2´), 1.65-1.55 (m, 2H, H-b), 1.40-1.33 (m, 2H, H-c), 1.28 (t, ${}^3J_{HH}$= 7.4 Hz, 21H, $HN(CH_2CH_3)_3^+$), 0.90 (t, ${}^3J_{HH}$= 7.3 Hz, 3H, H-d). **^{13}C-NMR (151 MHz, D_2O):** δ [ppm] = 166.2 (C-4), 151.7 (C-2), 141.8 (C-6), 102.9 (C-5), 95.1 (d, ${}^1J_{CF}$= 173.9 Hz, C-3´), 85.2 (C-1´), 84.0 (dd, ${}^3J_{CP}$= 25.0 Hz, ${}^3J_{CP}$= 8.8 Hz, C-4´), 66.6 (d, ${}^3J_{CP}$= 5.7 Hz, C-a), 65.4 (d, ${}^3J_{CP}$= 12.4 Hz, ${}^3J_{CP}$= 6.2 Hz, C-5´), 46.6 ($HN(CH_2CH_3)_3^+$), 37.5 (d, ${}^2J_{CF}$= 20.5 Hz, C-2´), 31.9 (d, ${}^3J_{CP}$= 7.0 Hz, C-b), 18.3 (C-c), 13.0 (C-d), 8.2 ($HN(CH_2CH_3)_3^+$). **^{31}P NMR (243 MHz, D_2O):** δ [ppm] = -10.65 -- -11.2 (m, 1P, P-γ), -11.50 -- -12.02 (m, 1P, P-α), -22.80 -- -23.80 (m, 1P, P-β). **HRMS (ESI$^-$, m/z):** calculated for $C_{13}H_{21}FN_2O_{13}P_3$ [M-H]$^-$ 525.0246; found, 525.0164. **^{19}F-NMR (188 MHz, D_2O):** δ [ppm] = -174.2 -- -174.8.

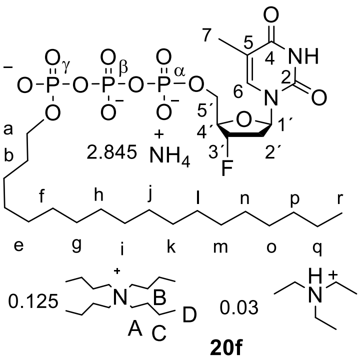

20f

γ-(C18)-FLTTP 20f. According to general procedure 2 with 116 mg *H*-phosphonate **17a** (0.3 mmol, 1.0 equiv.), 80 mg NCS (0.6 mmol, 2.0 equiv.), 2.3 mL of tetrabutylammonium phosphate (0.9 mmol, 3.0 equiv), 169 mg (*n*-Bu$_4$N)$_2$·FLTMP (0.21 mmol, 0.7 equiv.), and 0.97 mL 40% *n*Bu$_4$N$^+$OH$^-$ in H_2O (1.50 mmol, 5 equiv). Reaction time was 8 h. The mixture following a Dowex 50WX8 (NH_4^+) ion exchange, freeze-drying, and purified by automatic RP18 flash chromatography again. The desired products were obtained as solids after freeze-drying again. Yield: 60 mg

(0.07 mmol, 35%) white solid. HPLC-UV analysis confirmed purity: > 98%. **^{1}H NMR (400 MHz, CD_3OD):** δ [ppm]= 7.86 (d, $^4J_{HH}$= 1.2 Hz, 1H, H-6), 6.38 (dd, $^3J_{HH}$= 9.0 Hz, $^3J_{HH}$= 5.8 Hz, 1H, H-1´), 5.54 (dd, $^2J_{HH}$= 54.4 Hz, $^3J_{HH}$=4.2 Hz, 1H, H-3´), 4.42-4.32 (m, 1H, H-4´), 4.32-4.27 (m, 1H, H-5´a), 4.22-4.15 (m, 1H, H-5´b), 3.99 (q, $^3J_{HH}$= 6.6 Hz, 2H, H-a), 3.28-3.21 (m, 1H, H-A), 3.21-3.17 (m, 0.18H, $HN(CH_2CH_3)_3^+$), 2.50-2.34 (m, 2H, H-2´), 1.94 (d, $^4J_{HH}$= 1.1 Hz, 3H, H-7), 1.70-1.60 (m, 3H, H-b, H-B), 1.47-1.35 (m, 3H, H-c, H-C), 1.35-1.25 (m, 28.24H, $HN(CH_2CH_3)_3^+$, H-d, H-e, H-f, H-j, H-h, H-i, H-j, H-k, H-l, H-m, H-n, H-o, H-p, H-q), 1.03 (t, $^3J_{HH}$= 7.4 Hz, 1.5 H, H-D), 0.90 (d, $^3J_{HH}$= 7.1 Hz, 3H, H-r). **^{13}C NMR (101 MHz, CD_3OD):** δ [ppm] = 166.4 (C-4), 152.5 (C-2), 137.9 (C-6), 112.4 (C-5), 96.3 (d, $^1J_{CF}$= 174.6 Hz, C-3´), 86.0 (C-1´), 85.4 (dd, $^2J_{CP}$= 25.9 Hz, $^3J_{CP}$= 9.6 Hz, C-4´), 67.6 (d, $^3J_{CP}$= 6.2 Hz, C-a), 66.8 (dd, $^2J_{CP}$= 12.6 Hz, $^3J_{CP}$= 6.8 Hz, C-5´), 59.5 (t, $^3J_{CP}$= 2.8 Hz, C-A), 47.5 ($HN(CH_2CH_3)_3^+$), 38.8 (d, $^2J_{CP}$= 20.9 Hz, C-2´), 31.8 (d, $^3J_{CP}$= 8.0 Hz, C-b^2), 33.1, 30.79, 30.77, 30.76, 30.6, 30.5, 23.7 (C-d, C-e, C-f, C-g, C-h, C-i, C-j, C-k, C-l, C-m, C-n, C-o, C-p, C-q) 26.9 (C-c), 24.8 (C-B), 20.7 (C-C), 14.4 (C-r), 13.9, (C-D), 12.6 (C-7), 9.1 ($HN(CH_2CH_3)_3^+$). **^{31}P NMR (162 MHz, CD_3OD):** δ [ppm] = -10.82 (d, $^2J_{pp}$= 20.3 Hz, P-γ), -11.78 (d, $^2J_{pp}$= 17.8 Hz, P-α), -22.55 (t, $^2J_{pp}$= 18.0 Hz, P-β). **MALDI-MS (m/z):** calculated for $C_{28}H_{52}FN_2O_{13}P_3$ $[M-H]^-$ 735.2594; found, 735.1581. **^{19}F-NMR (188 MHz, CD_3OD):** δ [ppm] = -175.3 -- -175.8.

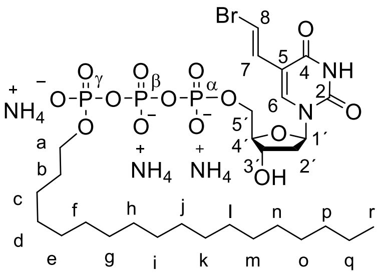

20g

γ-C18-BVDUTP 20g. According to general procedure 2 with 116 mg *H*-phosphonate **17a** (0.3 mmol, 1.0 equiv.), 80 mg NCS (0.6 mmol, 2.0 equiv.), 2.3 mL of tetrabutylammonium phosphate (0.9 mmol, 3.0 equiv), 188 mg (n-$Bu_4N)_2$·BVdUMP (0.21 mmol, 0.7 equiv.), and 0.97 mL 40% $nBu_4N^+OH^-$ in H_2O (1.50 mmol, 5 equiv). Reaction time was 8 h. The mixture following a Dowex 50WX8 (NH_4^+) ion exchange, freeze-drying, and purified by automatic RP18 flash chromatography again. The desired products were obtained as solids after freeze-drying again. Yield: 63 mg (0.07 mmol, 34%) white solid. HPLC-UV analysis confirmed purity: > 98%. **^{1}H-NMR (500 MHz, CD_3OD):** δ [ppm] = 8.01 (s, 1H, H-6), 7.41 (d, $^2J_{HH}$= 13.6 Hz, 1H, H-8), 7.01 (d, $^2J_{HH}$= 13.6 Hz, 1H, H-7), 6.29 (t, $^3J_{HH}$= 6.6 Hz, 1H, H-1´), 4.64-4.56 (m, 1H, H-3´), 4.32-4.16 (m, 2H, H-5´), 4.07-4.03 (m, 1H, H-4´), 3.99 (q, $^3J_{HH}$= 6.7 Hz, 2H, H-a), 2.32-2.23 (m, 2H, H-2´), 1.65 (quint, $^3J_{HH}$= 6.7 Hz, 2H, H-b), 1.45-1.25 (m, 30H, H-c, H-d, H-e, H-f, H-j, H-h, H-i, H-j, H-k, H-l, H-m, H-n, H-o, H-p, H-q), 0.90 (t, $^3J_{HH}$= 6.8 Hz, 3H, H-r). **^{13}C-NMR (126 MHz, CD_3OD):** δ [ppm] = 163.8 (C-4), 151.2 (C-2), 140.3 (C-6), 130.8 (C-7), 112.4 (C-5), 109.2 (C-8), 87.4 (d, $^3J_{CP}$= 9.2 Hz, C-4´), 86.5 (C-1´), 71.8 (C-3´), 66.7 (d, $^3J_{CP}$= 6.5 Hz, C-a), 66.6 (d, $^3J_{CP}$= 5.8 Hz, C-5´), 40.9 (C-2´), 31.7 (t, $^3J_{CP}$= 8.1 Hz, C-b), 33.1, 30.79, 30.75, 30.6, 30.5, 30.3, 23.7 (C-d, C-e, C-f, C-g, C-h, C-i, C-j, C-k, C-l, C-m, C-n, C-o, C-p, C-q), 26.9 (C-c), 14.4 (C-r). **^{31}P NMR (162 MHz, CD_3OD):** δ [ppm] = -10.95 (d, $^2J_{pp}$= 19.6 Hz, P-α), -11.42 (d, $^2J_{pp}$= 19.8 Hz, P-γ), -22.70 (t, $^2J_{pp}$= 18.9 Hz, P-β). **MALDI-MS (m/z):** calculated for $C_{29}H_{52}BrN_2O_{14}P_3$ $[M-H]^-$ 823.1742; found, 823.2665.

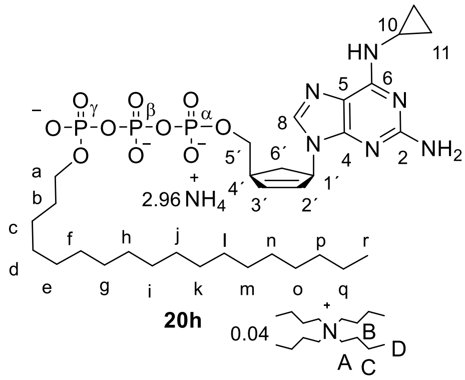

20h

γ-C18-ABCTP 20h. According to general procedure 2 with 116 mg *H*-phosphonate **17a** (0.3 mmol, 1.0 equiv.), 80 mg NCS (0.6 mmol, 2.0 equiv.), 2.3 mL of tetrabutylammonium phosphate (0.9 mmol, 3.0 equiv), 178 mg (n-$Bu_4N)_3$·ABCMP (0.21 mmol, 0.7 equiv.), and 0.97 mL 40% $nBu_4N^+OH^-$ in H_2O (1.50 mmol, 5 equiv). Reaction time was 8 h. The mixture following a Dowex 50WX8 (NH_4^+) ion exchange, freeze-drying, and purified by automatic RP18 flash chromatography again. The desired products were obtained as solids after freeze-drying again. Yield: 39 mg (0.05 mmol, 22%) white solid. HPLC-UV analysis confirmed purity: > 95%. **^{1}H-NMR (500 MHz, CD_3OD):** δ [ppm] = 8.12 (d, $^3J_{HH}$= 2.8 Hz, 1H, H-8), 6.30-6.20 (m, 1H, H-3´), 5.88-5.65 (m, 1H, H-2´), 5.60-5.50 (m, 1H, H-1´), 4.25-4.05 (m, 2H, H-6´), 4.05-3.90 (m, 2H, H-a), 3.29-3.05 (m, 2.32H, H-A, H-4´, H-10), 2.82-2.70 (m, 1H, H-5´$_a$), 2.15-1.85 (m, 1H, H-5´$_b$), 1.70-1.52 (m, 2.32H, H-B, H-b), 1.38-1.24 (m, 30.32H, H-c, H-d, H-e, H-f, H-g, H-h, H-i, H-j, H-k, H-l, H-m, H-n, H-o, H-p, H-q), 1.03 (t, $^3J_{CP}$= 7.2 Hz, 0.48H, H-D), 1.05-0.94 (m, 18H, H-j^1, H-D), 0.95-0.57 (m, 7H, H-r, H-11). **^{13}C-NMR (126 MHz, CD_3OD):** δ [ppm] = 167.2 (C-2), 158.9 (C-6), 157.4 (C-4), 139.6 (C-3´), 137.6 (C-8), 130.6 (C-2´), 114.7 (C-5), 69.4 (d, $^3J_{CP}$= 6.2 Hz, C-6´), 66.9 (d, $^3J_{CP}$= 5.8 Hz, C-a), 60.4 (C-1´), 59.5 (t, $^3J_{CP}$= 2.7 Hz, C-A), 47.6 (d, $^3J_{CP}$= 8.3 Hz, C-4´), 36.1 (C-5´), 31.9 (t, $^3J_{CP}$= 8.1 Hz, C-b), 33.1, 30.79, 30.78, 30.75, 30.67, 30.5, 23.7 (C-d, C-e, C-f, C-g, C-h, C-i, C-j, C-k, C-l, C-m, C-n, C-o, C-p, C-q), 27.0 (C-c), 24.8 (C-B, C-10), 20.7 (t, $^4J_{CP}$= 1.3 Hz, C-C), 14.4 (C-r), 14.0 (C-D), 7.6 (C-11). **^{31}P NMR (162 MHz, CD_3OD):** δ [ppm] = -10.95 (d, $^2J_{pp}$= 19.6 Hz, P-α), -11.42 (d, $^2J_{pp}$= 19.8 Hz, P-γ), -22.70 (t, $^2J_{pp}$= 18.9 Hz, P-β). **MALDI-MS (m/z):** calculated for $C_{32}H_{57}N_6O_{10}P_3$ $[M-H]^-$ 777.3276; found, 777.2652.

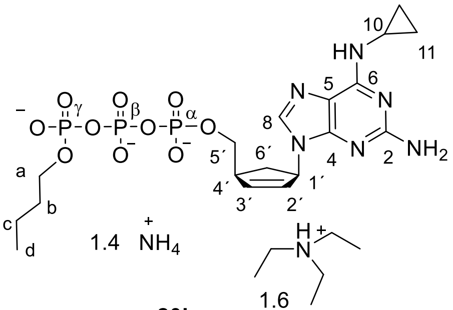

$20h_2$

γ-C4-ABCTP $20h_2$. According to general procedure 2 with 58 mg *H*-phosphonate **17b** (0.3 mmol, 1.0 equiv.), 80 mg NCS (0.3 mmol, 1.0 equiv.), 2.3 mL of tetrabutylammonium phosphate (0.9 mmol, 3.0 equiv), 178 mg (n-$Bu_4N)_3$·ABCMP (0.21 mmol, 0.7 equiv.), and 0.97 mL 40% $nBu_4N^+OH^-$ in H_2O (1.50 mmol, 5 equiv). Reaction time was 8 h. The mixture following a Dowex 50WX8 (NH_4^+) ion exchange, freeze-drying, and purified by automatic RP18 flash chromatography again. The desired products were obtained as solids after freeze-drying again. Yield: 27 mg (0.04 mmol, 17%) white solid. HPLC-UV analysis confirmed purity: > 95%. **^{1}H-NMR (500 MHz, D_2O):** δ [ppm] = 8.02 (s, 1H, H-8), 6.33-6.24 (m, 1H, H-3´), 5.95-5.88 (m, 1H, H-2´), 5.55-5.47 (m, 1H, H-1´), 4.15-3.96 (m, 2H, H-6´), 3.89 (q, $^3J_{CP}$= 6.6 Hz, 2H, H-a), 3.20 (q, $^3J_{HH}$= 7.4 Hz, 9.6H, $HN(CH_2CH_3)_3^+$), 3.10-2.76 (m, 3H, H-4´, H-10, H-5´$_a$), 1.77-1.70 (m, 1H, H-5´$_b$), 1.55-1.45 (m, 2H, H-b), 1.35-1.18 (m, 16.4H, H-c, $HN(CH_2CH_3)_3^+$), 1.05-0.95 (m, 2H, H-10$_a$), 0.80 (t, $^3J_{HH}$= 7.5 Hz, 3H, H-d), 0.78-0.65 (m, 2H, H-10$_b$). **^{13}C-NMR (126 MHz, D_2O):** δ [ppm] = 161.2 (C-6), 160.0 (C-4), 139.5 (C-8), 134.2 (C-3´), 129.0 (C-2´), 114.2 (C-5), 68.2 (d, $^3J_{CP}$= 6.0 Hz, C-6´), 66.5 (d, $^3J_{CP}$= 5.9 Hz, C-a), 60.1 (C-1´), 46.6, 45.6 ($HN(CH_2CH_3)_3^+$), 42.2 (C-4´), 33.7 (C-5´), 31.9 (t, $^3J_{CP}$= 7.3 Hz, C-b), 18.3 (C-c), 13.0 (C-d), 10.5, 8.2 ($HN(CH_2CH_3)_3^+$), 6.8 (C-11), the remaining signals could not be detected. **^{31}P NMR (162 MHz, D_2O):** δ [ppm]

= -10.7 -- -11.3 (m, 2P, P-α, P-γ), -23.36 (t, $^{2}J_{pp}$= 17.9 Hz, P-β). **HRMS (ESI$^-$, m/z):** calculated for $C_{18}H_{29}N_6O_{10}P_3$ [M-H]$^-$ 581.1085; found, 581.1084.

Part 4. Chemical Hydrolysis of Tri*PPP*ro-compounds 10 and 20.

First prepare 50 mM solutions of Tri*PPP*ro-compounds **10** and **20** in DMSO. Second prepare 1.9 mM hydrolysis solutions of Tri*PPP*ro-compounds **10** and **20** in DMSO and H_2O: 22 µL stock solution (50 mM in DMSO), 378 µL Milli-Q water, 200 µL DMSO, and 600 µL phosphate buffer saline (PBS, 50 mM, pH 7.3). The solution was incubated at 37 °C in a thermomixer, at different time 40 µL of solution per extraction and stored in the freezer (-30 °C). Finally, aliquots of 25 µL (injection volume) were analyzed by analytical HPLC (HPLC Method). The half-lives ($t_{1/2}$) of Tri*PPP*ro-compounds **10** and **20** were obtained using Origin.

Part 5. Hydrolysis of Tri*PPP*ro-compounds 10 and 20 with PLE.

First prepare 6.0 mM hydrolysis solutions of Tri*PPP*ro-compounds **10** and **20** in DMSO and H_2O: 10 µL stock solution (50 mM in DMSO), 31.7 µL DMSO, and 41.7 µL Milli-Q water. Second, 125 µL DMSO and 833 µL PBS buffer (50 mM, pH 7.3) were added to 6.0 mM hydrolysis solutions. 62.5 µL of PLE solution in PBS (100 units/mL) was added to the mixture, the reaction was started and the solution was also incubated at 37 °C in a thermomixer. At different times, aliquots of 75 µL were taken and stopped by addition to 79.6 mL MeOH. The mixture was stored in in the freezer (- 30 °C). When testing, aliquots of 60 µL (injection volume) were analyzed by analytical HPLC (HPLC Method). The half-lives ($t_{1/2}$) of Tri*PPP*ro-compounds **10** and **20** were obtained similar to that for the chemical hydrolysis studies.

Part 6. Enzyme-Catalyzed Hydrolysis of Tri*PPP*ro-compounds 10 and 20 in CEM Cell extracts.

First prepare 3.0 mM hydrolysis solutions of Tri*PPP*ro-compounds **10** and **20** in DMSO and H_2O: 21 µL stock solution (50 mM in DMSO) and 154 µL DMSO, and 175 µL Milli-Q water. Three-twelve different samples (20 µL 3.0 mM hydrolysis solutions) were prepared in 2 mL Eppendorf® vials. Then, aliquots of 50 µL human CEM cell extracts were added to these samples and the reaction was started. The samples were incubated at 37 °C in a thermomixer for different time periods. The reaction was stopped by addition of 150 µL MeOH and kept on ice for 5 min. After centrifugation at 14,000 rpm for 12 min, the supernatants were filtered (Chromafil RC-20/15 MS, 0.2 µm) and stored in liquid nitrogen. When testing, aliquots of 60 µL (injection volume) were analyzed by analytical HPLC (HPLC Method). The calculation of $t_{1/2}$ was obtained using Origin again.

Part 7. Hydrolysis of Tri*PPP*ro-compounds 10 and 20 in human plasma.

First prepare 3.0 mM hydrolysis solutions of Tri*PPP*ro-compounds **10** and **20** in DMSO and H_2O: 21 µL stock solution (50 mM in DMSO) and 154 µL DMSO, and 175 µL Milli-Q water. Three-twelve different samples (20 µL 3.0 mM hydrolysis solutions) were prepared in 2 mL Eppendorf® vials. Then, aliquots of 50 µL human Plasma (Heparin or Citrate) were added to these samples and the reaction was started. The samples were

incubated at 37 °C in a thermomixer for different time periods. The reaction was stopped by addition of 150 μL MeOH and kept on ice for 5 min. After centrifugation at 14,000 rpm for 22 min, the supernatants were filtered (Chromafil RC-20/15 MS, 0.2 μm) and stored in liquid nitrogen. When testing, aliquots of 25 0r 30 μL (injection volume) were analyzed by analytical HPLC (HPLC Method). The calculation of $t_{1/2}$ was obtained using Origin again.

Part 8. Preparation of cell extracts and human plasma.

Human CD_4^+ T-lymphocyte CEM cells were prepared following the previously reported procedure.[5] Briefly, CEM cells were grown in RPMI-1640-based cell culture medium to a final density of ~$3\cdot10^6$ cells/mL. Cells were centrifuged for 10 min at 1,250 rpm at 4 °C, washed twice with cold PBS, and the pellet was re-suspended at 10^8 cells/mL and sonicated (Hielscher Ultrasound Techn., 100% amplitude, 3·times for 10 sec) to destroy cell integrity. Next, the resulting cell suspension was centrifuged at 10,000 rpm to remove cell debris. Finally, the supernatant was divided in aliquots and frozen at -80 °C.

Fresh human plasma was obtained from citrate (0.106 mol/L) and lithium heparin (25 IU/mL) anticoagulated whole blood samples upon centrifugation (3,000 x g for 10 min at RT). Finally, the supernatant was divided in aliquots and frozen at -80 °C.

Part 9. Anti-HIV activity assay.

The produce was used as previously reported.[5] Inhibition of HIV-1(III_B)- and HIV-2(ROD)-induced cytopathogenicity in wild-type CEM/0 and thymidine kinase-deficient CEM/TK^- cell cultures was measured in microtiter 96-well plates containing ~$3\cdot10^5$ CEM cells/mL infected with 100 $CCID_{50}$ of HIV per milliliter and containing appropriate dilutions of the test compounds. After 4−5 days of incubation at 37 °C in a CO_2-controlled humidified atmosphere, CEM giant (syncytium) cell formation was examined microscopically. The EC_{50} (50% effective concentration) was defined as the compound concentration required to inhibit HIV-induced giant cell formation by 50%.

Part 10. Primer-extension assays.

Primer extension assays were performed as described before.[5-7] HIV-RT and human polymerases α, β and γ were obtained from Roboklon. The fluorescent labeled primer and template were obtained from Metabion. Gel size was adjusted to the electrophoresis chamber (450 mm×200 mm×1.0 mm)

Primer sequence for the Cy3-fluorescent labeled primer extension experiment:

5´-Cy3-CGTTG GTCCT GAAGG AGGAT AGGTT-3´

Template-sequence:

3´-GCAAC CAGGA CTTCC TCCTA TCCAA AGACA-5´

A-Template-sequence:

5´-Cy3-GCAACCAGGACTTCCTCCTATCCAATAGAC-3´

C-Template-sequence:

5´-Cy3-GCAACCAGGACTTCCTCCTATCCAAGTAGA-3´

T-Template-sequence:

5´-Cy3-GCAACCAGGACTTCCTCCTATCCAAAGACA-3´

G-Template-sequence:

5´-Cy3-GCAAC CAGGA CTTCC TCCTA TCCAA CGTAG-3´

Following conditions were used in the primer extension experiments:

1. Hybridisation: Primer and template (10 µM) were mixed in 1:1.5 reatio. The mixture was incubated for 5 min. at 95 °C and then cooled to -20 °C over 3 hours.

2. HIV-RT assay reaction conditions: 50 mM Tris-HCl (pH 8.6), 10 mM $MgCl_2$, 40 mM KCl, 250 µM dNTPs, HIV-RT (6 U or 3 U), 0.20 µM DNA hybrid, incubated at 37 °C for 30 min, 80 °C for 7 min. Primer extension assays: 50 mA, 45w for 4 h.

3. Human DNA polymerase β assay conditions: 60 mM Tris-HCl (pH 8.7), 5 mM MgOAc, 1.0 mM dithiothreitol, 0.1 mM spermine, 0.01% (w/v) bovine serum albumin, 15% glycerol, 250 µM dNTPs, 2 U human DNA polymerase β, 0.20 µM DNA hybrid, incubated at 37 °C for 60 min, 80 °C for 7 min. Primer extension assays: 50 mA, 45w for 3 h.

4. Human DNA polymeras α assay conditions: 60 mM Tris-HCl (pH 8), 5 mM MgOAc, 1.0 mM dithiothreiol, 0.1 mM spermine, 0.01% (w/v) bovine serum albumin, 250 µM dNTPs, 2 U human polymerase α, 0.20 µM DNA hybrid, incubated at 37 °C for 60 min, 80 °C for 7 min. Primer extension assays: 50 mA, 45w for 3 h.

5. Human polymerase γ assay conditions: 60 mM Tris-HCl (pH 8), 5 mM MgOAc, 1.0 mM dithiothreiol, 0.1 mM spermine, 0.01% (w/v) bovine serum albumin, 250 µM dNTPs, 2 U human polymerase γ, 0.5 µM $MnCl_2$, 0.20 µM DNA hybrid, incubated at 37 °C for 120 min, 80 °C for 7 min. Primer extension assays: 50 mA, 45w for 3 h.

Part 11. Spectral Data for New Compounds.

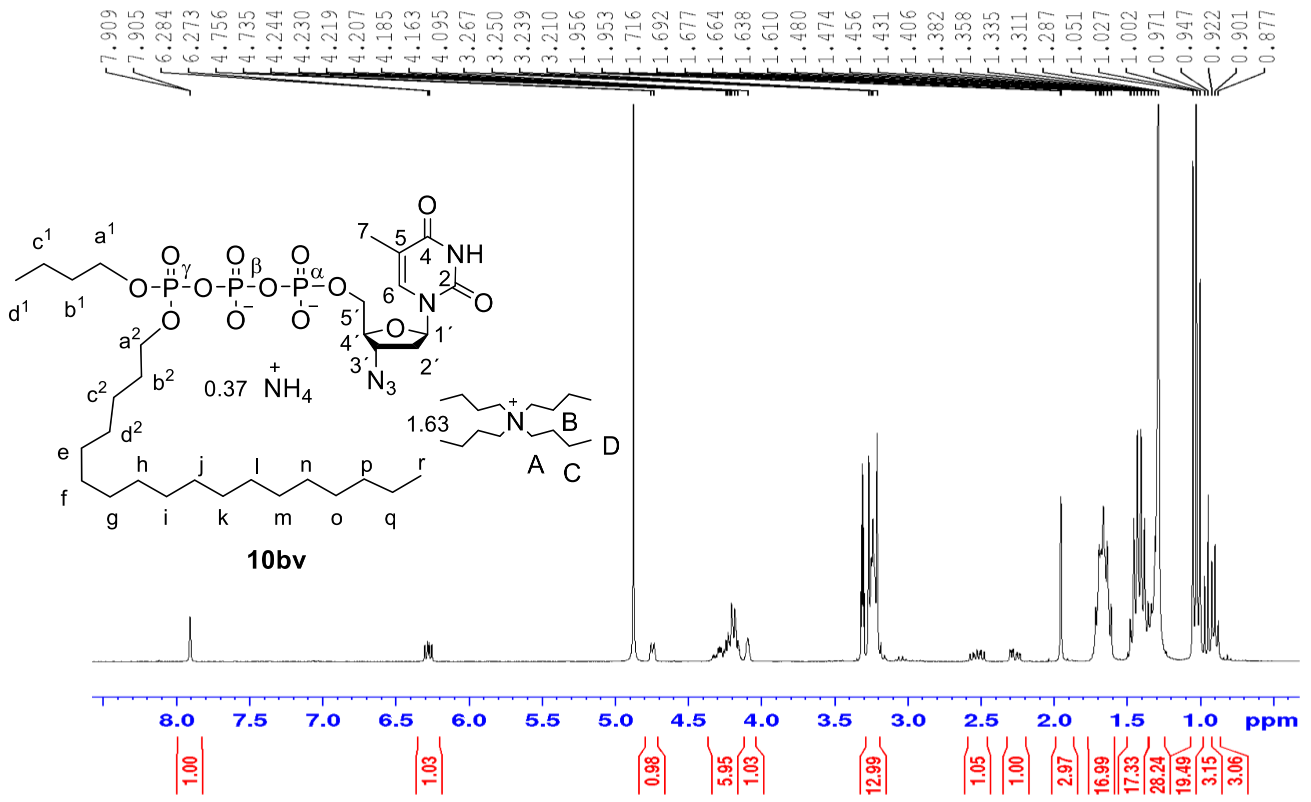

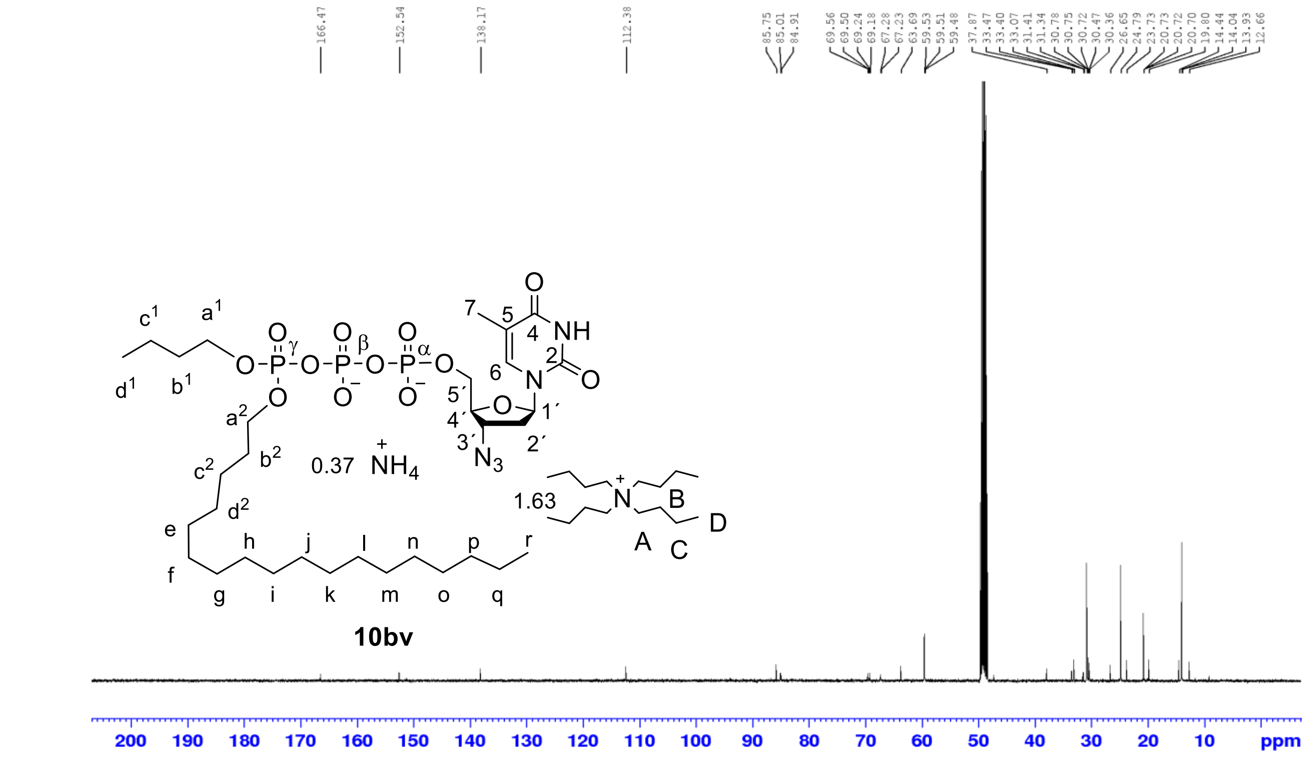

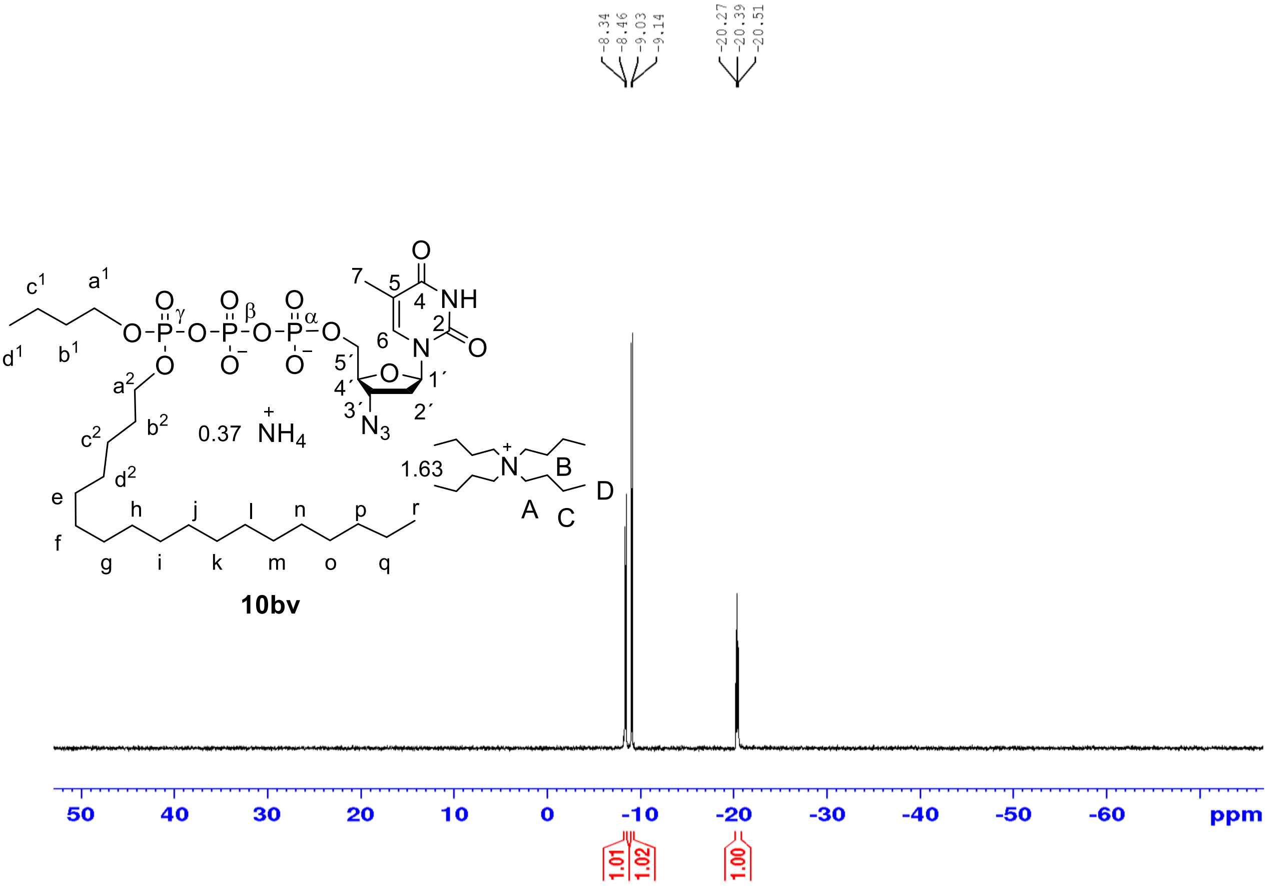

-8.34
-8.46
-9.03
-9.14
-20.27
-20.39
-20.51
0.37 NH4
1.63
A B C D
10bv
50 40 30 20 10 0 -10 -20 -30 -40 -50 -60 ppm
1.01 1.02 1.00

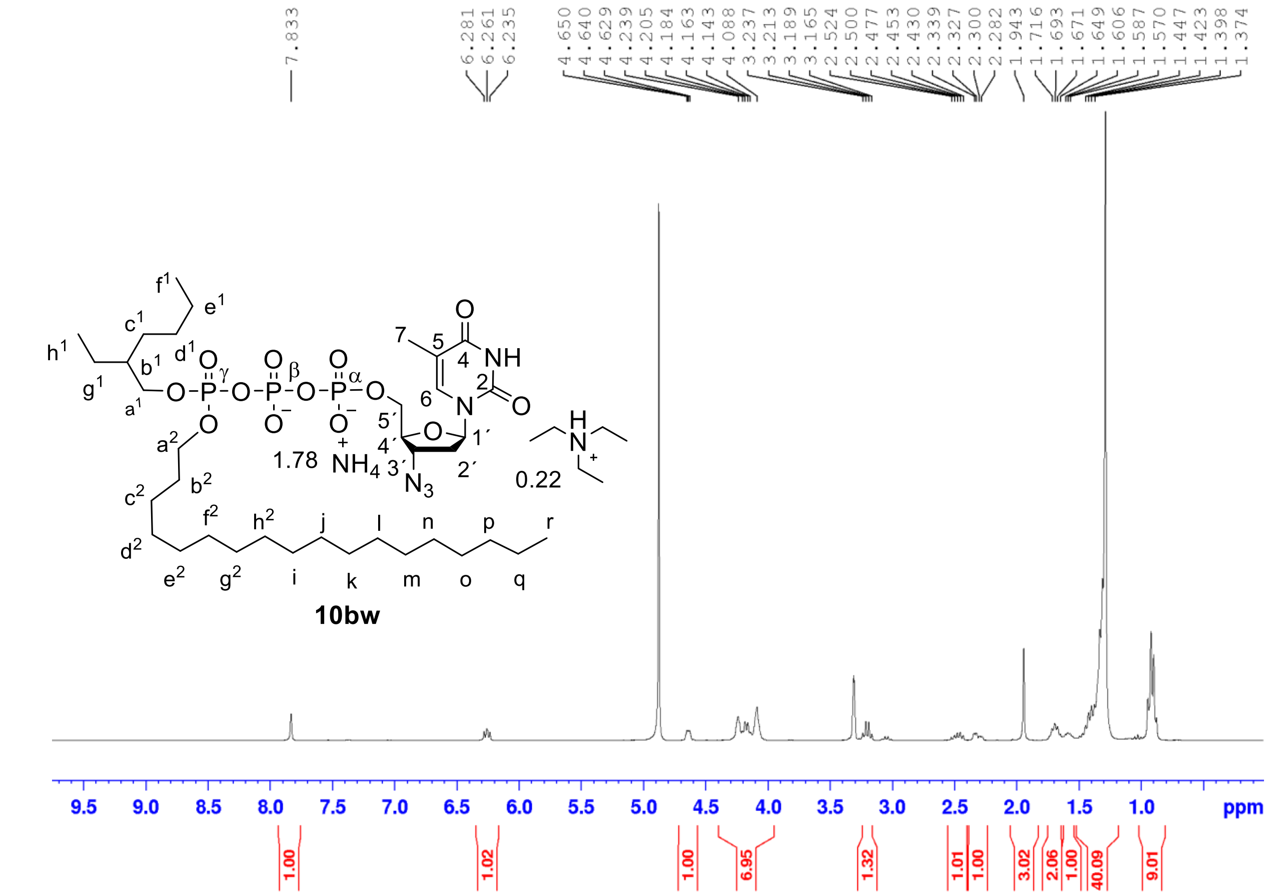

7.833
6.281
6.261
6.235
4.650
4.640
4.629
4.239
4.205
4.184
4.163
4.143
4.088
3.237
3.213
3.189
3.165
2.524
2.500
2.477
2.453
2.430
2.339
2.327
2.300
2.282
1.943
1.716
1.693
1.671
1.649
1.606
1.587
1.570
1.447
1.423
1.398
1.374
1.78 NH4
0.22
10bw
9.5 9.0 8.5 8.0 7.5 7.0 6.5 6.0 5.5 5.0 4.5 4.0 3.5 3.0 2.5 2.0 1.5 1.0 ppm
1.00 1.02 1.00 6.95 1.32 1.01 1.00 3.02 2.06 1.00 40.09 9.01

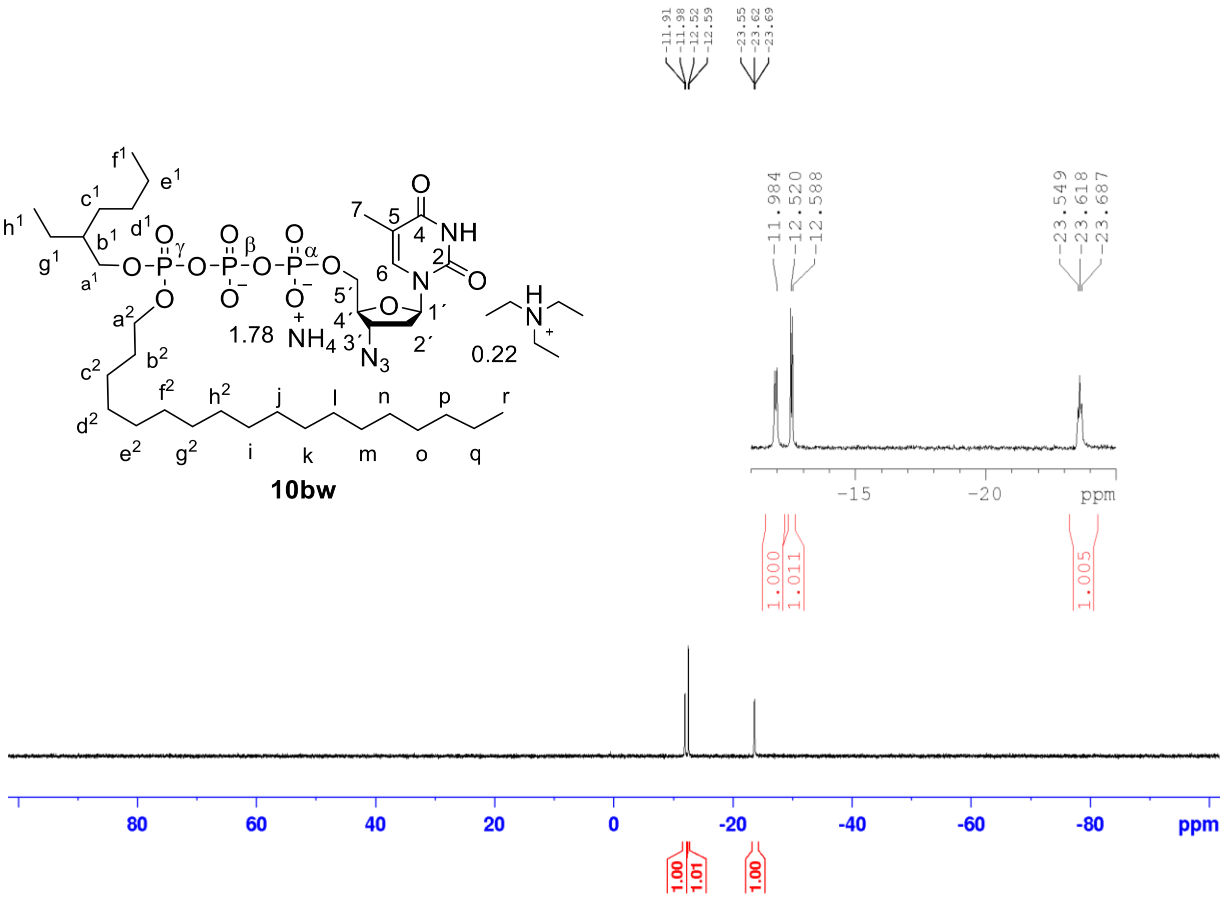

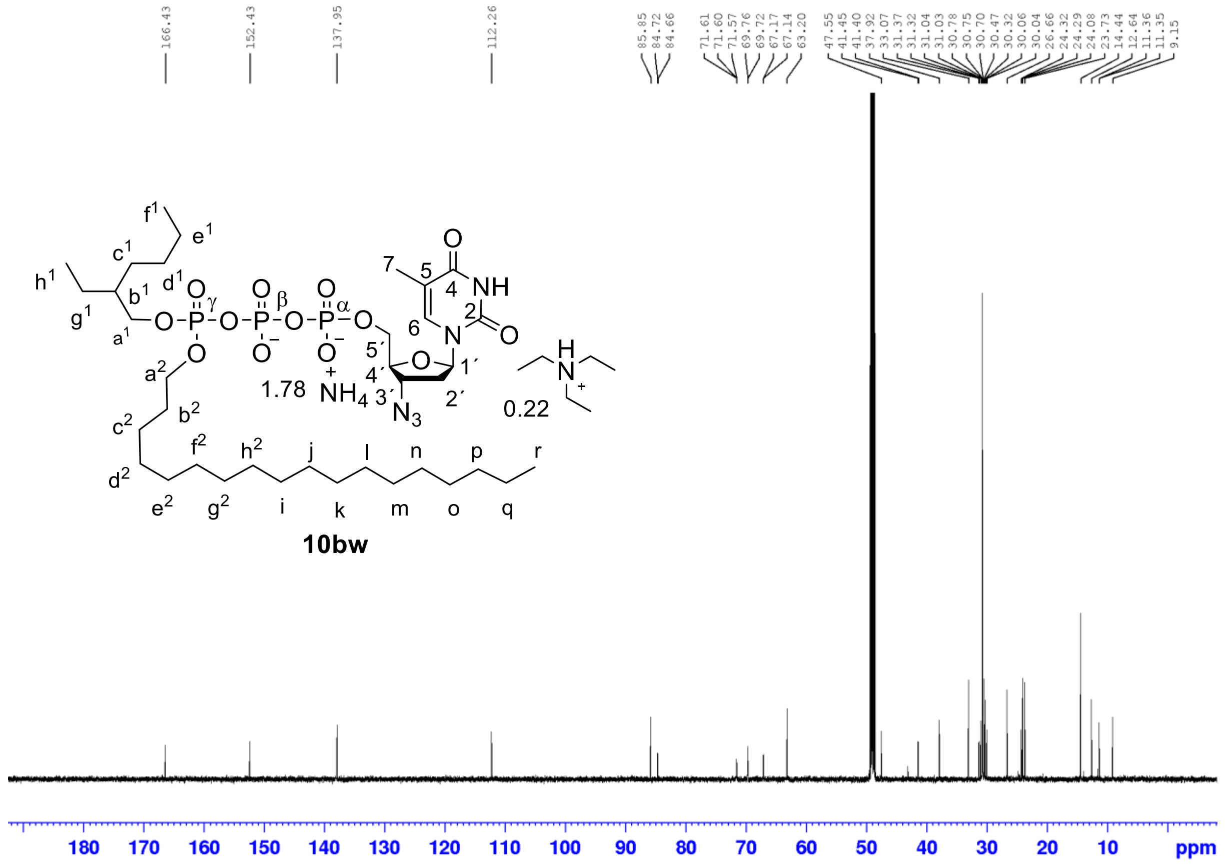

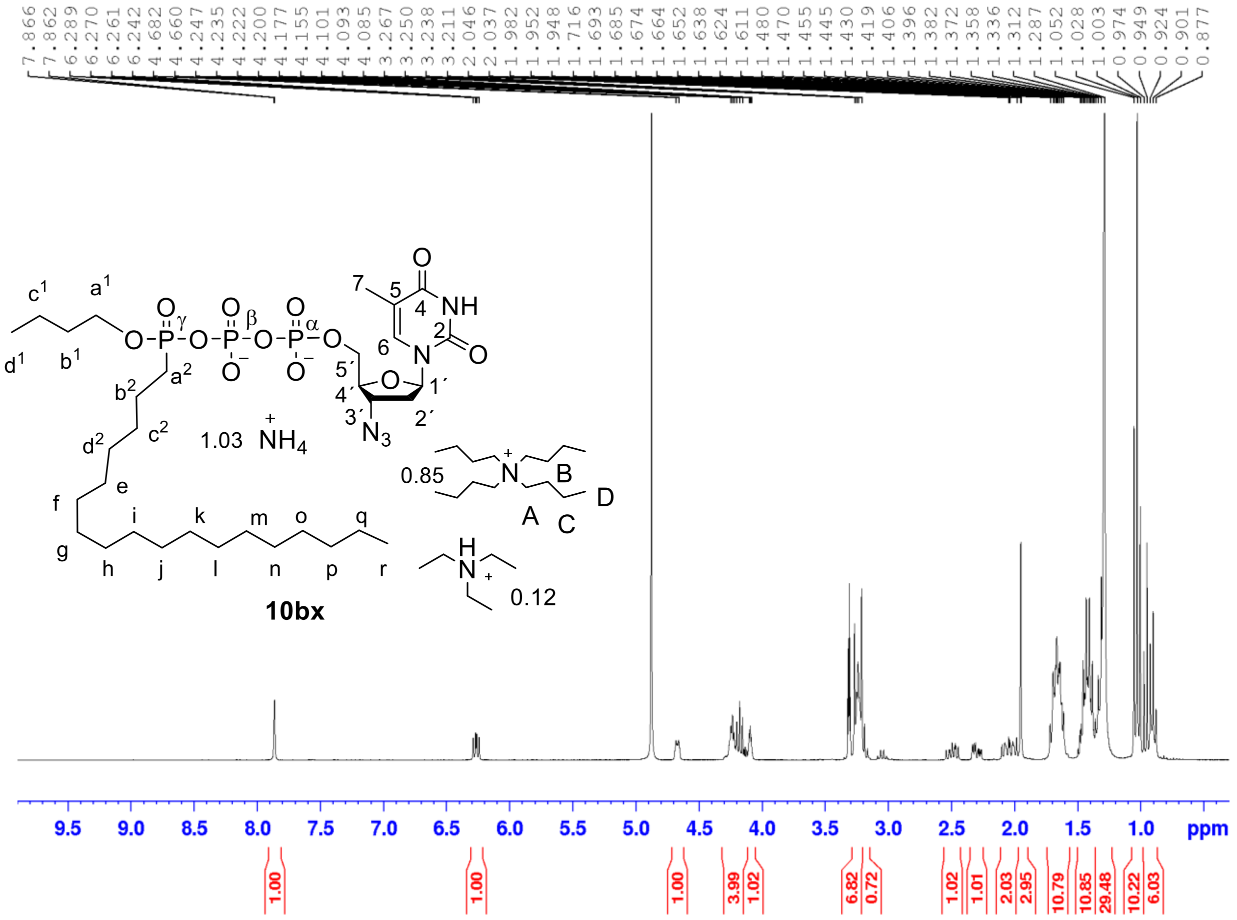

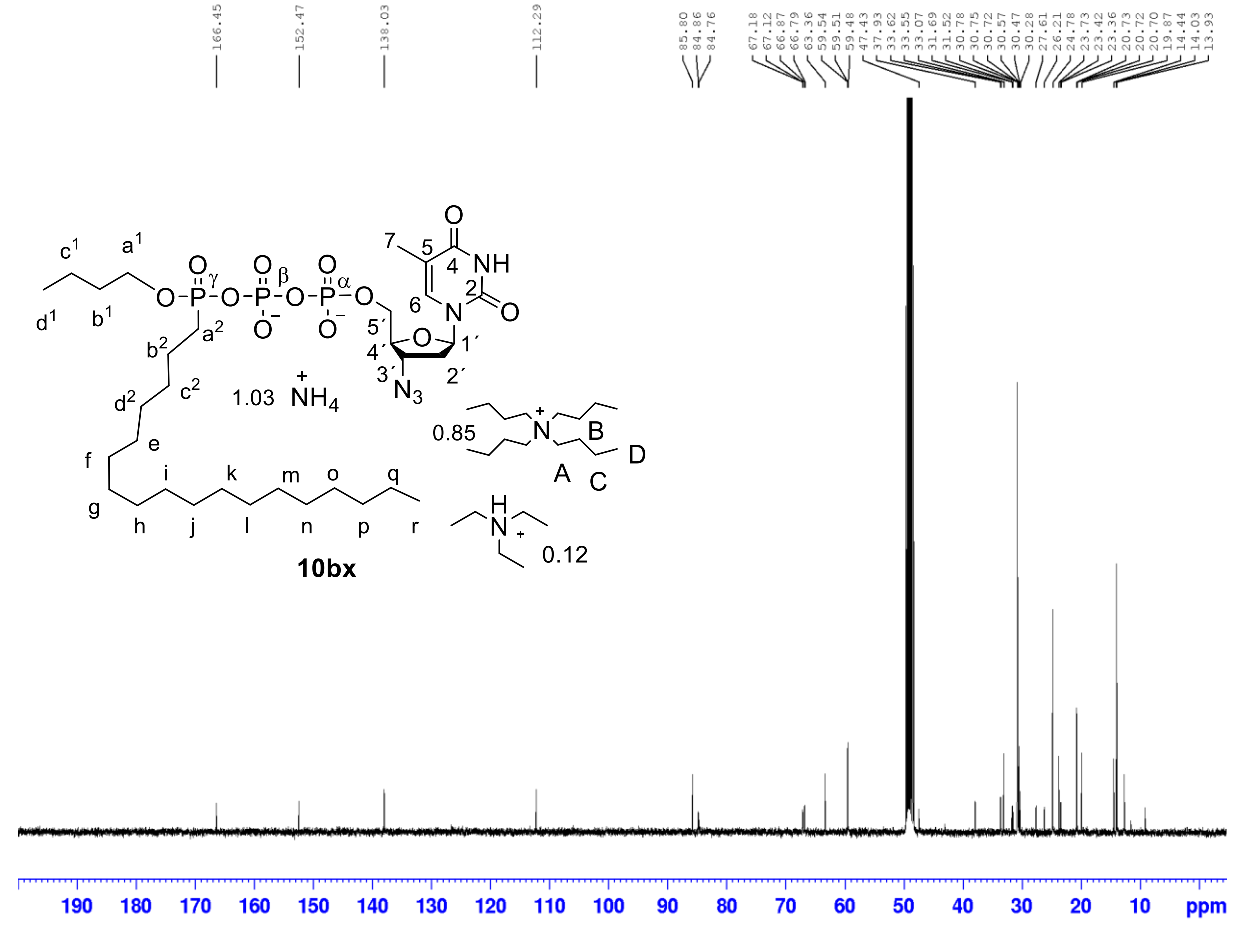

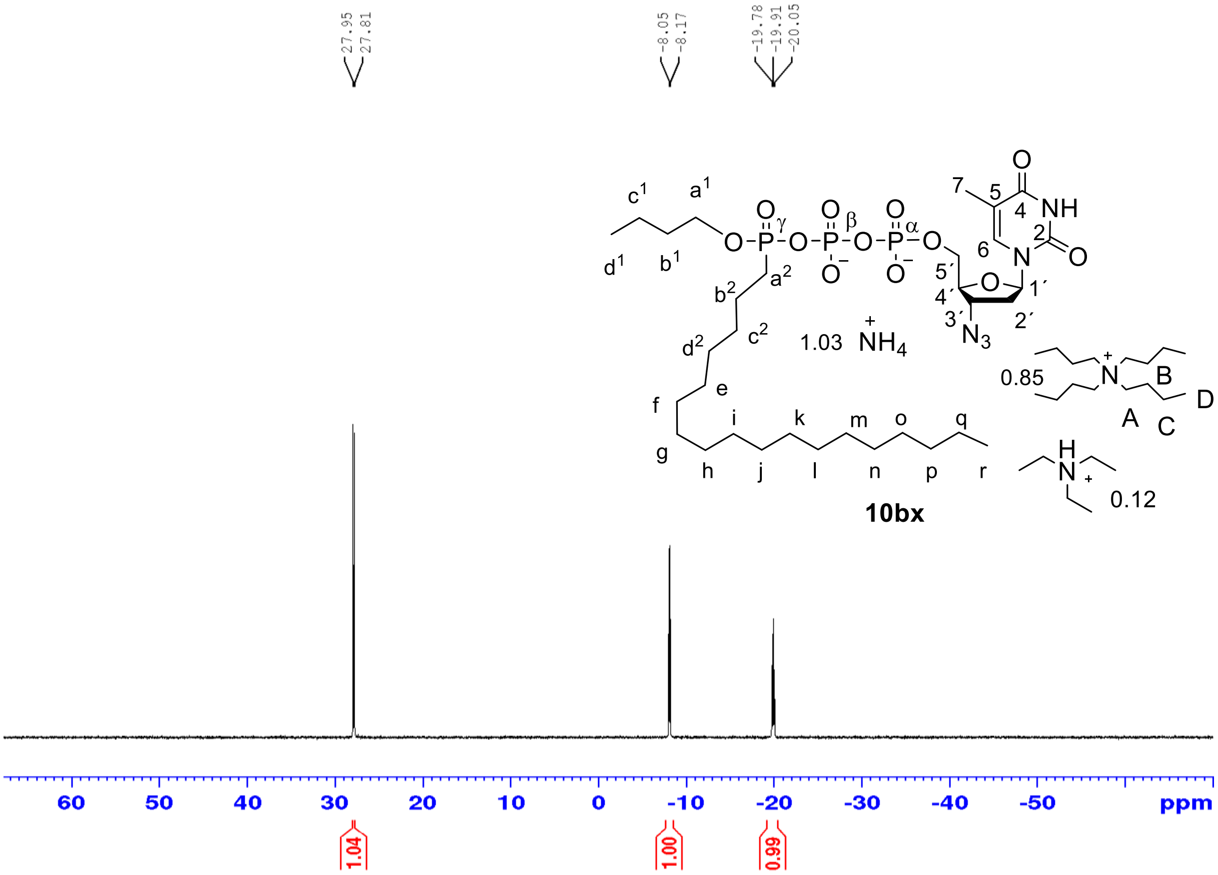
10bx
1.03
0.85
0.12
ppm

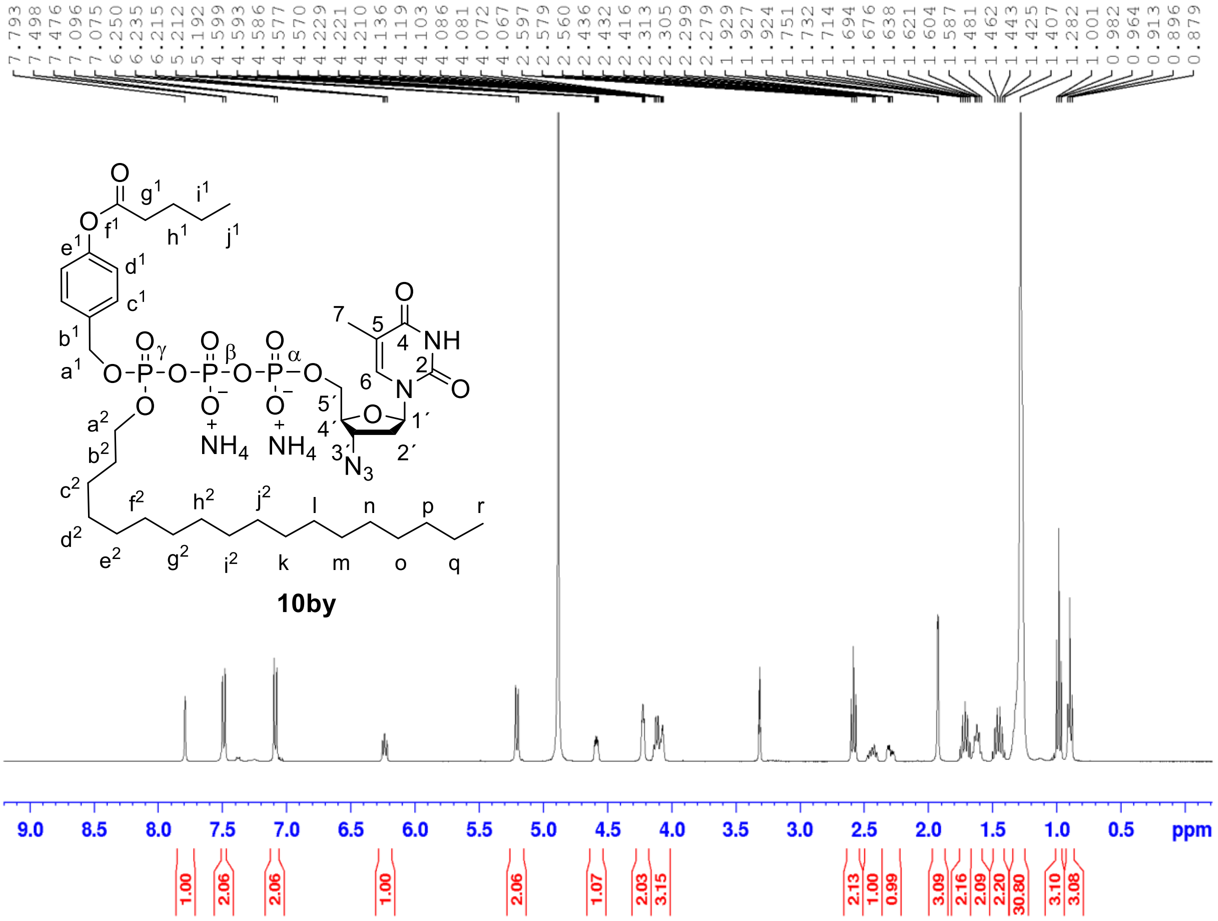
10by
ppm

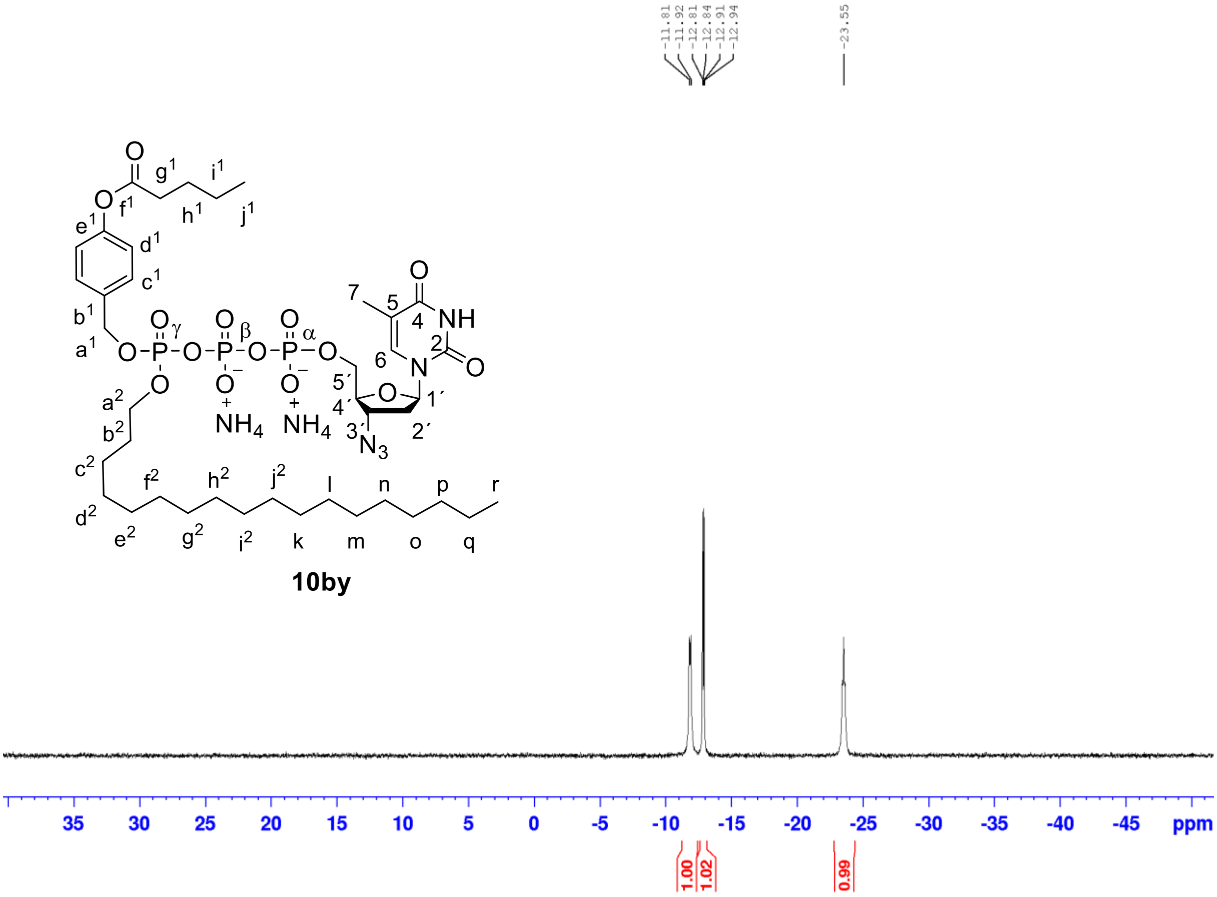

-11.81
-11.92
-12.81
-12.84
-12.91
-12.94
-23.55
10by
35 30 25 20 15 10 5 0 -5 -10 -15 -20 -25 -30 -35 -40 -45 ppm
1.00
1.02
0.99

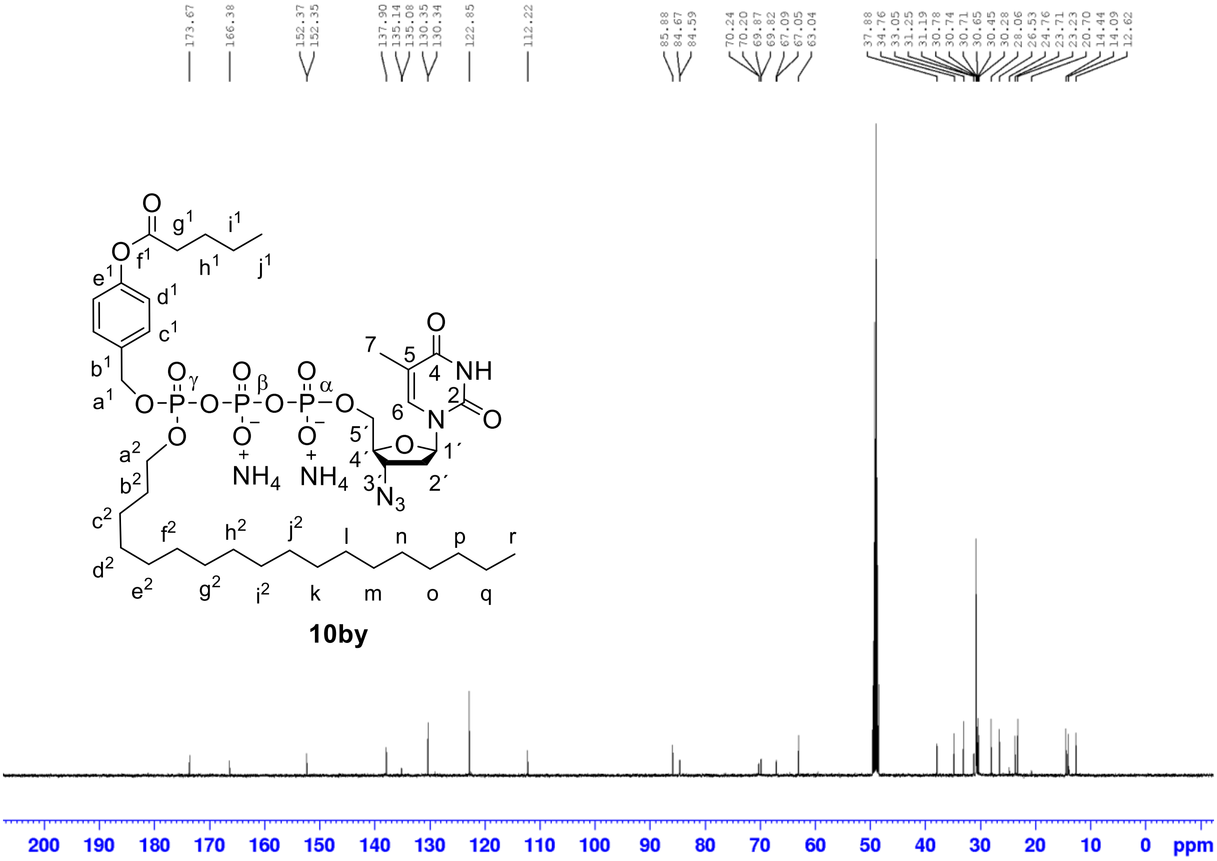

173.67
166.38
152.37
152.35
137.90
135.14
135.08
130.35
130.34
122.85
112.22
85.88
84.67
84.59
70.24
70.20
69.87
69.82
67.09
67.05
63.04
37.88
34.76
33.05
31.25
31.19
30.78
30.74
30.71
30.65
30.45
30.28
28.06
26.53
24.76
23.71
23.23
20.70
14.44
14.09
12.62
10by
200 190 180 170 160 150 140 130 120 110 100 90 80 70 60 50 40 30 20 10 0 ppm

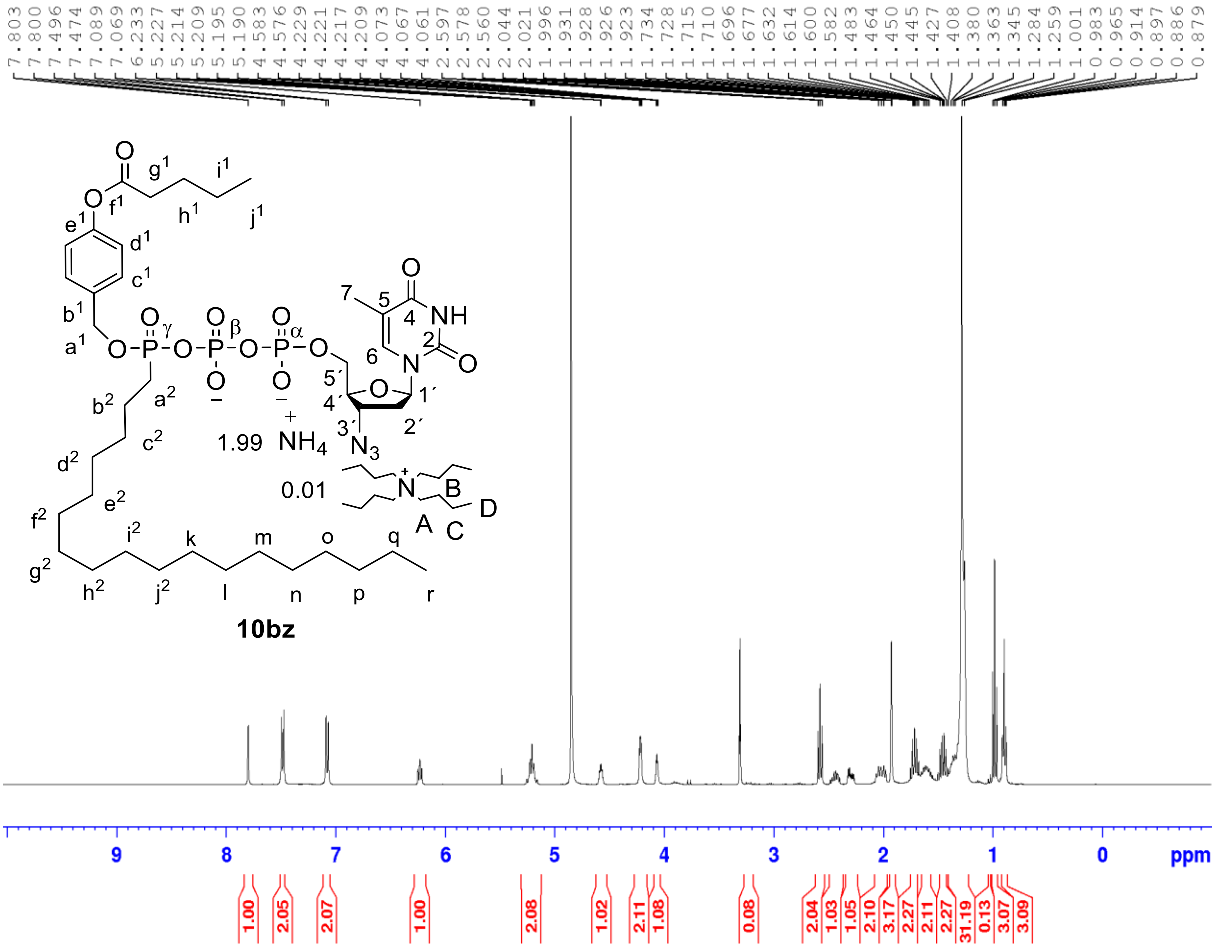

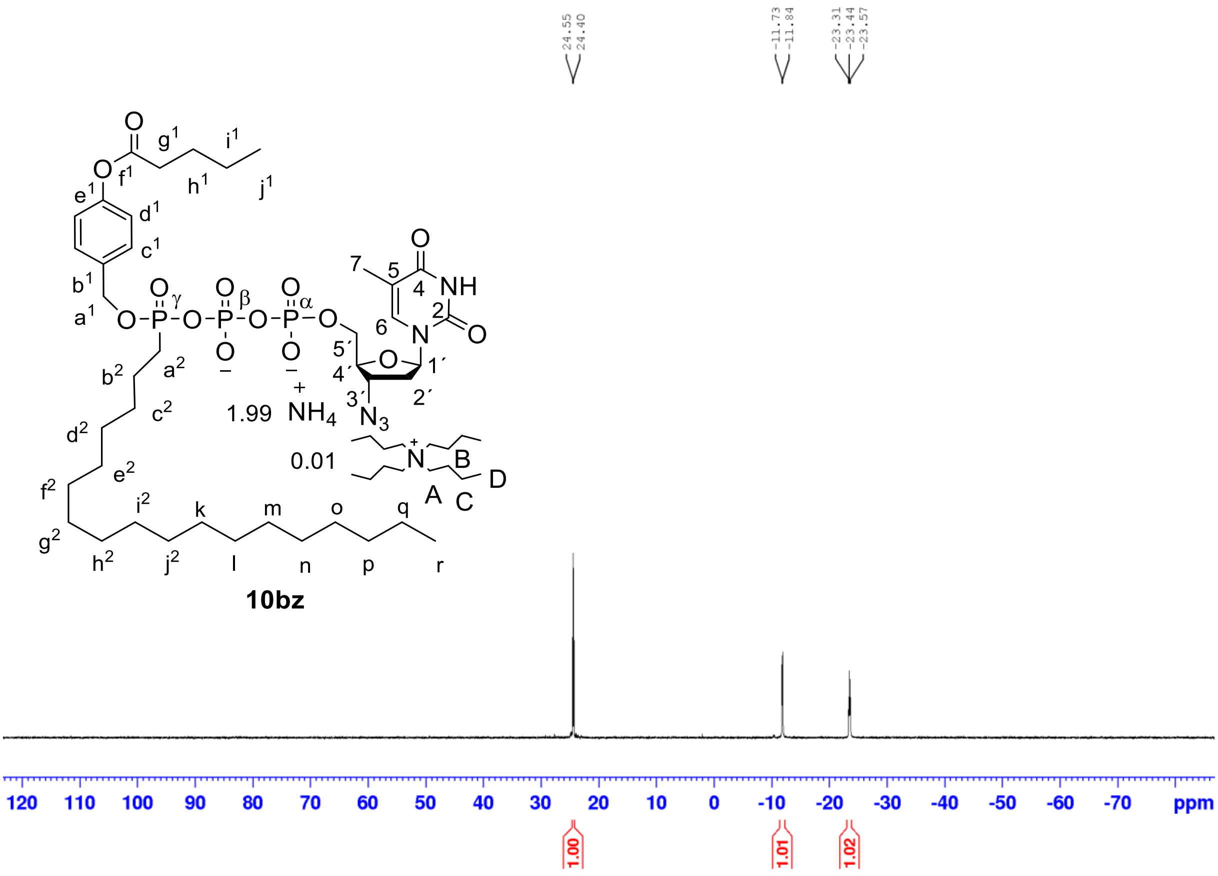

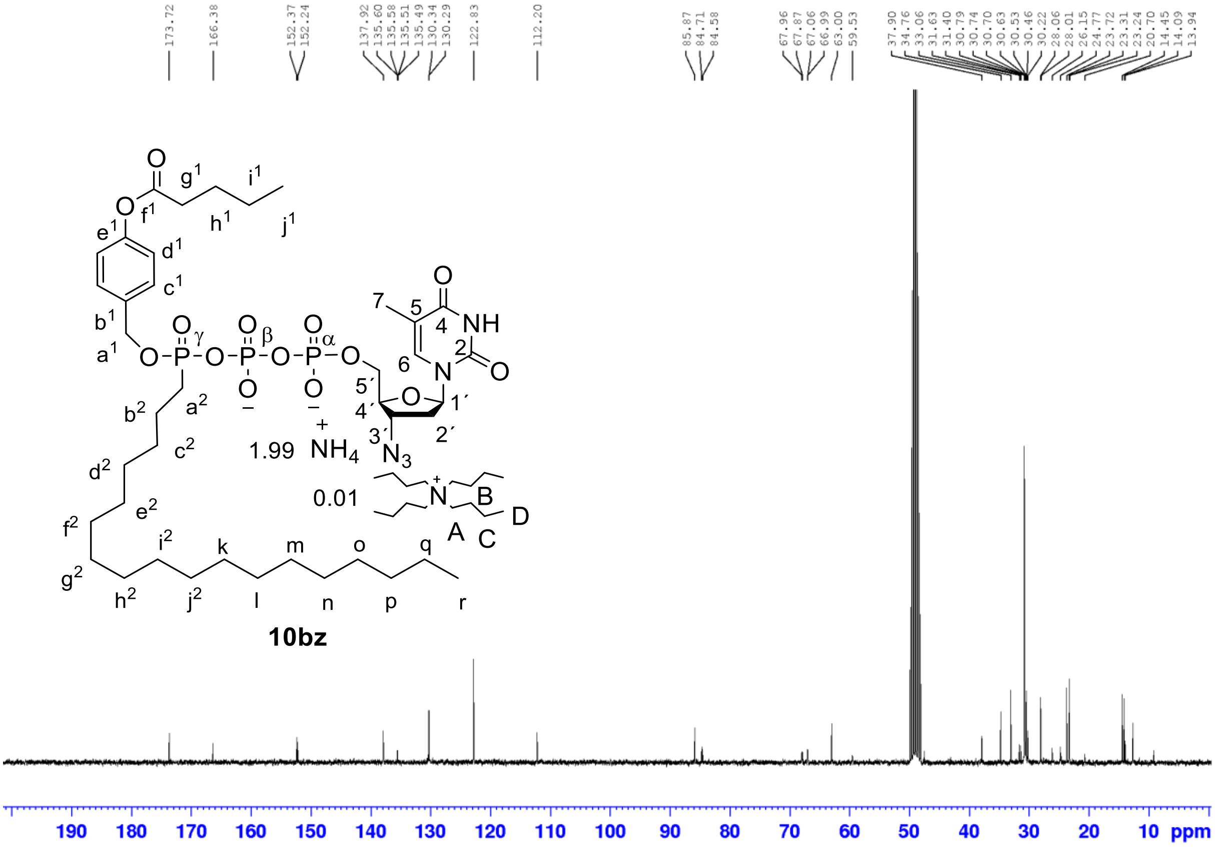

173.72
166.38
152.37
152.24
137.92
135.60
135.58
135.51
135.49
130.34
130.29
122.83
112.20
85.87
84.71
84.58
67.96
67.87
67.06
66.99
63.00
59.53
37.90
34.76
33.06
31.63
31.40
30.79
30.74
30.70
30.63
30.53
30.46
30.22
28.06
28.01
26.15
24.77
23.72
23.31
23.24
20.70
14.45
14.09
13.94
1.99 NH4
0.01
10bz
ppm

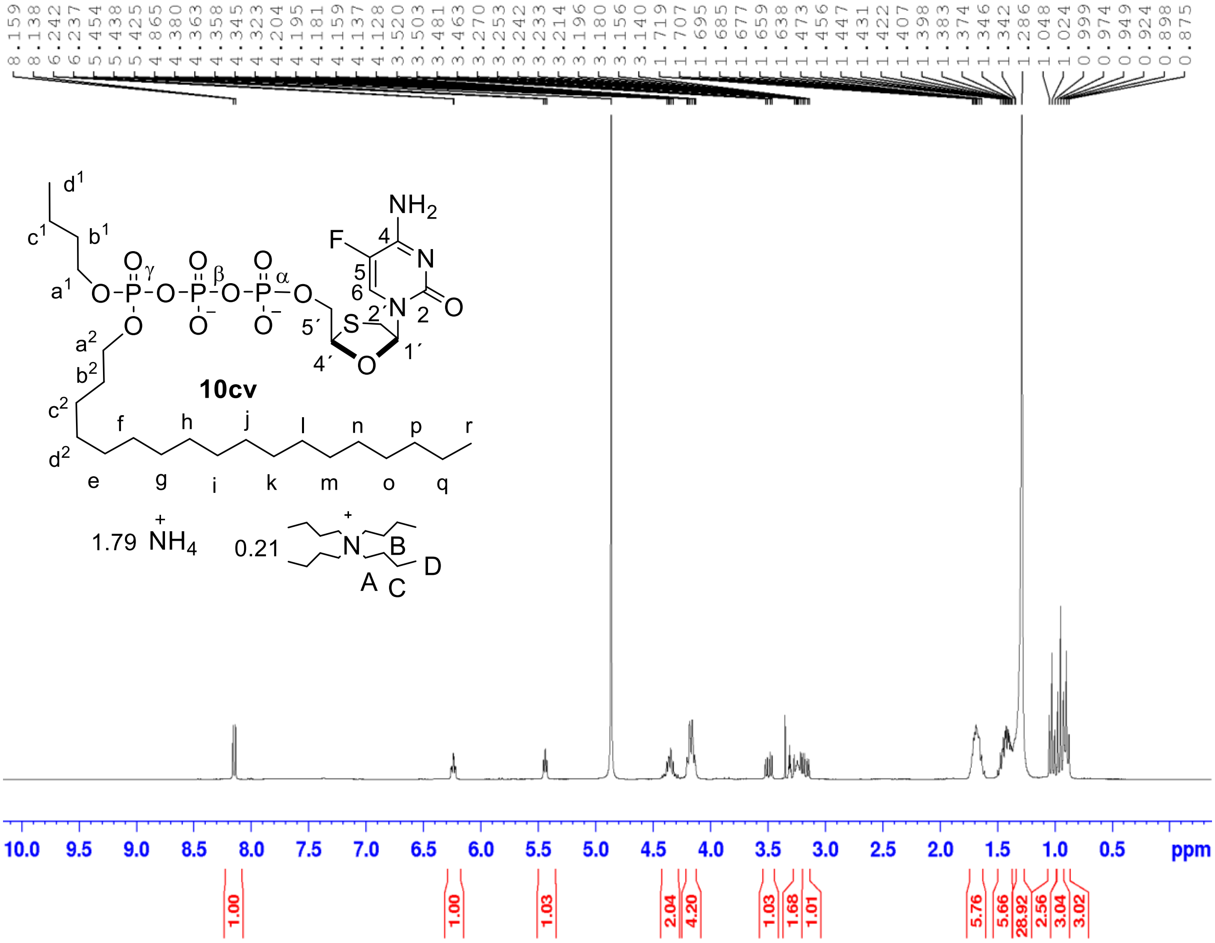

8.159
8.138
6.242
6.237
5.454
5.438
5.425
4.865
4.380
4.363
4.358
4.345
4.323
4.204
4.195
4.181
4.159
4.137
4.128
3.520
3.503
3.481
3.463
3.270
3.253
3.242
3.233
3.214
3.196
3.180
3.156
3.140
1.719
1.707
1.695
1.685
1.677
1.659
1.638
1.473
1.456
1.447
1.431
1.422
1.407
1.398
1.383
1.374
1.346
1.342
1.286
1.048
1.024
0.999
0.974
0.949
0.924
0.898
0.875
10cv
1.79 NH4
0.21
ppm
1.00
1.00
1.03
2.04
4.20
1.03
1.68
1.01
5.76
5.66
28.92
2.56
3.04
3.02

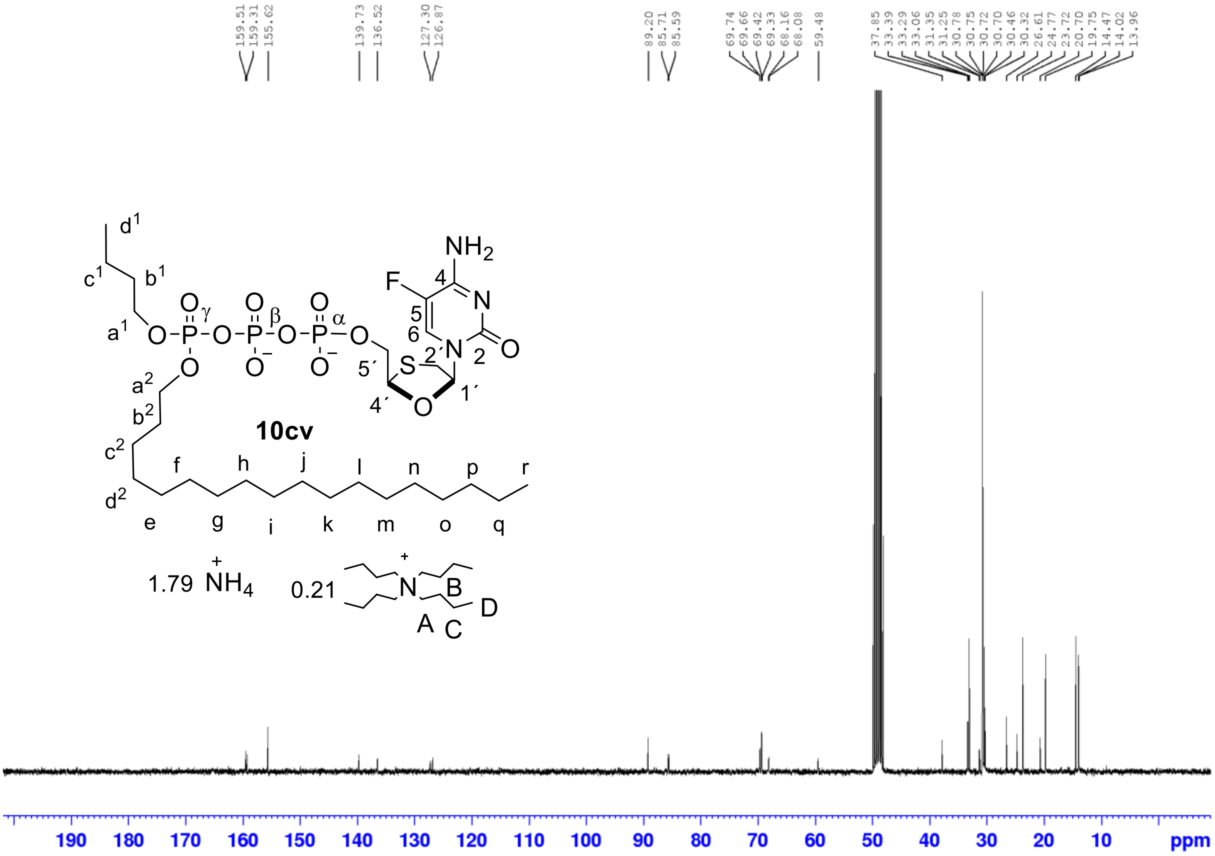
159.51
159.31
155.62
139.73
136.52
127.30
126.87
89.20
85.71
85.59
69.74
69.66
69.42
69.33
68.16
68.08
59.48
37.85
33.39
33.29
33.06
31.35
31.25
30.78
30.75
30.72
30.70
30.46
30.32
26.61
24.77
23.72
20.70
19.75
14.47
14.02
13.96
10cv
1.79 NH4
0.21
190 180 170 160 150 140 130 120 110 100 90 80 70 60 50 40 30 20 10 ppm

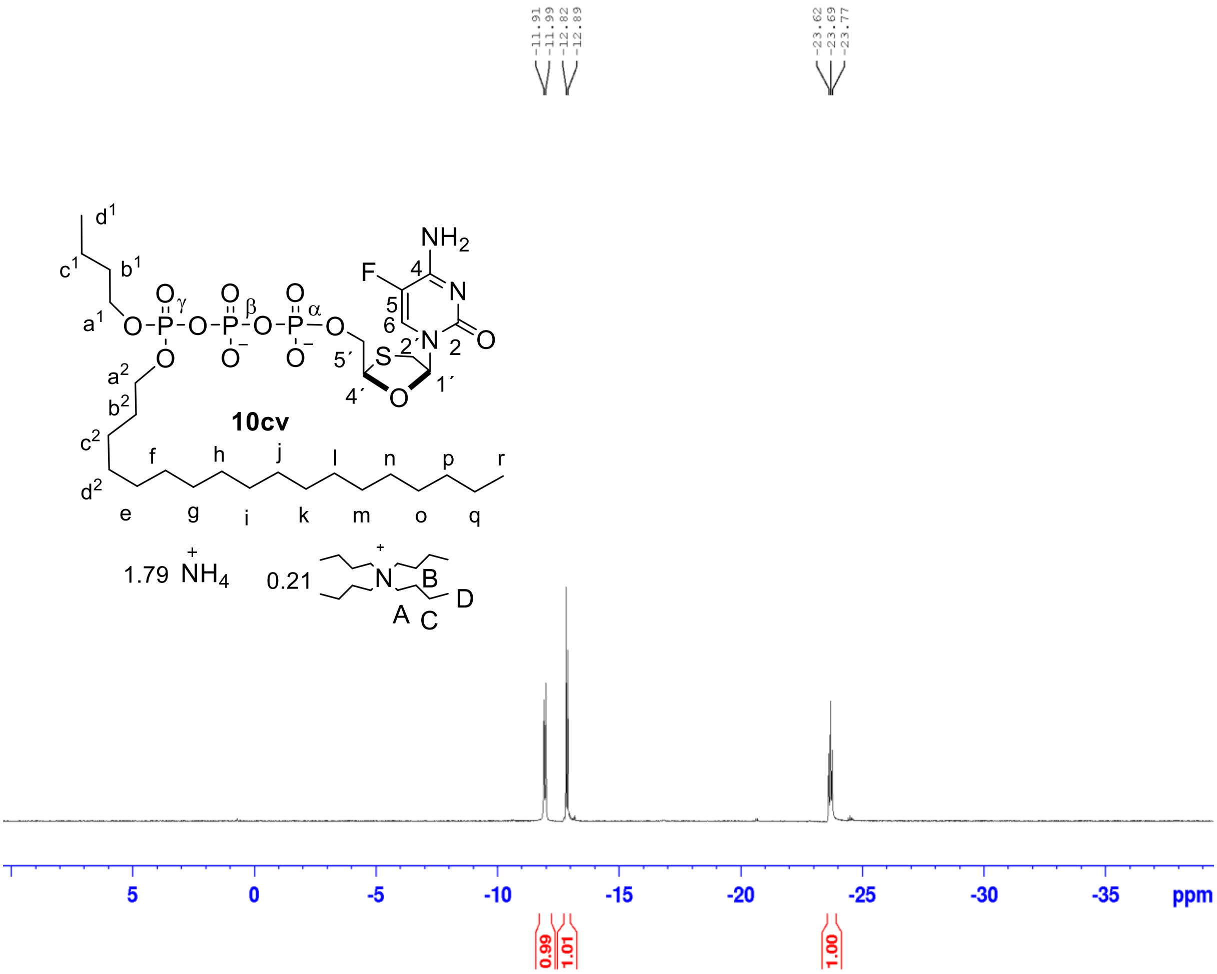
-11.91
-11.99
-12.82
-12.89
-23.62
-23.69
-23.77
10cv
1.79 NH4
0.21
5 0 -5 -10 -15 -20 -25 -30 -35 ppm
0.99
1.01
1.00

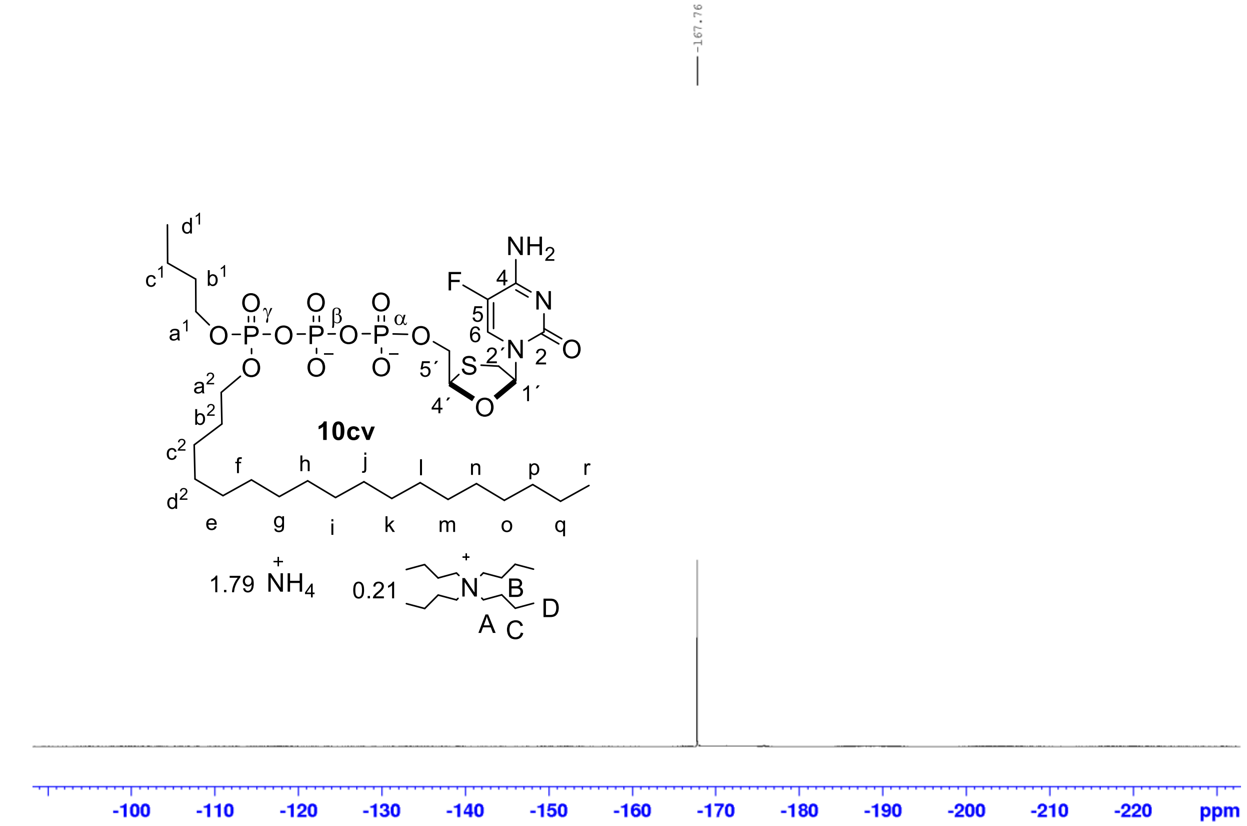
-167.76
10cv
1.79 NH4
0.21
ppm

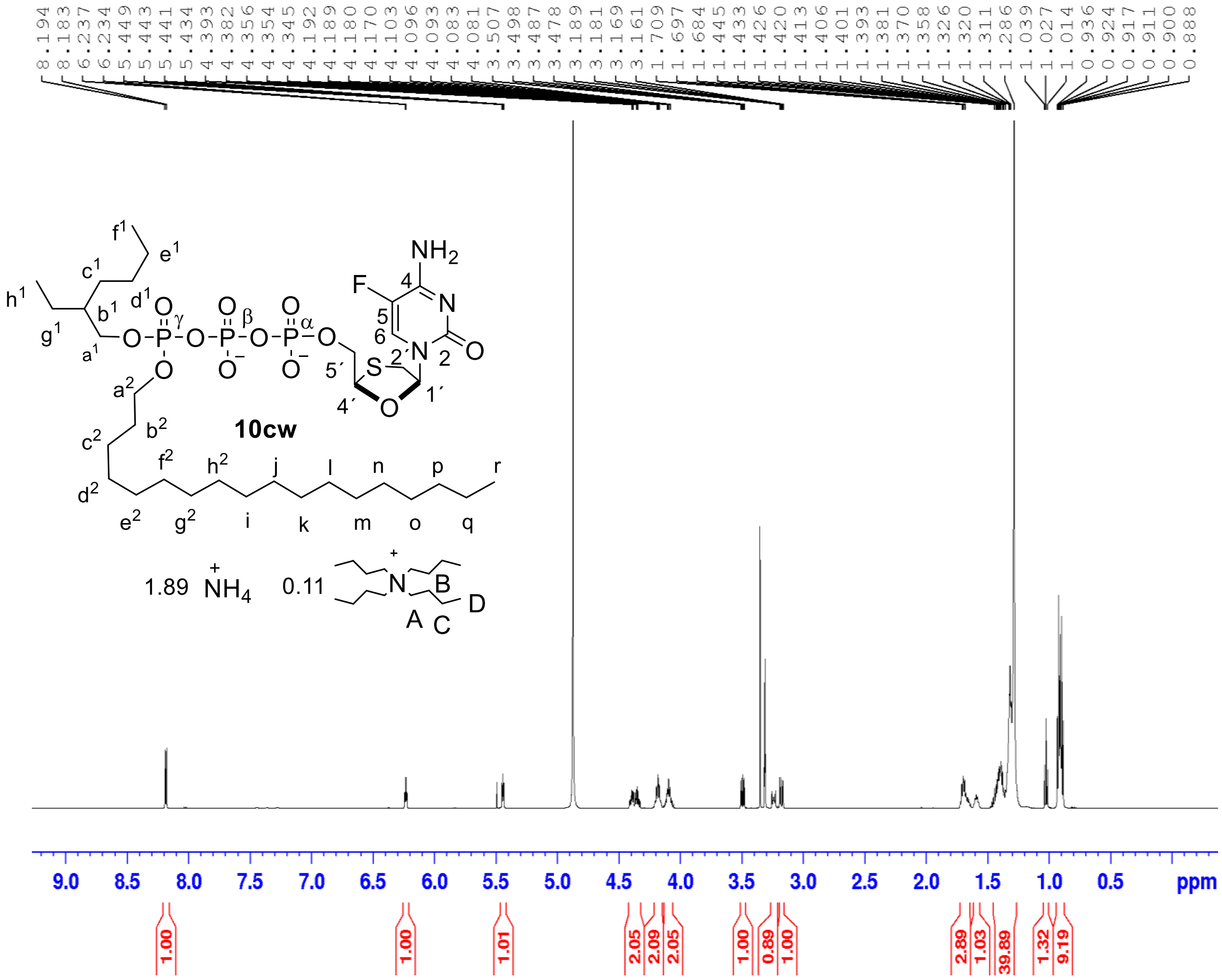
10cw
1.89 NH4
0.11
ppm
1.00
1.00
1.01
2.05
2.09
2.05
1.00
0.89
1.00
2.89
1.03
39.89
1.32
9.19

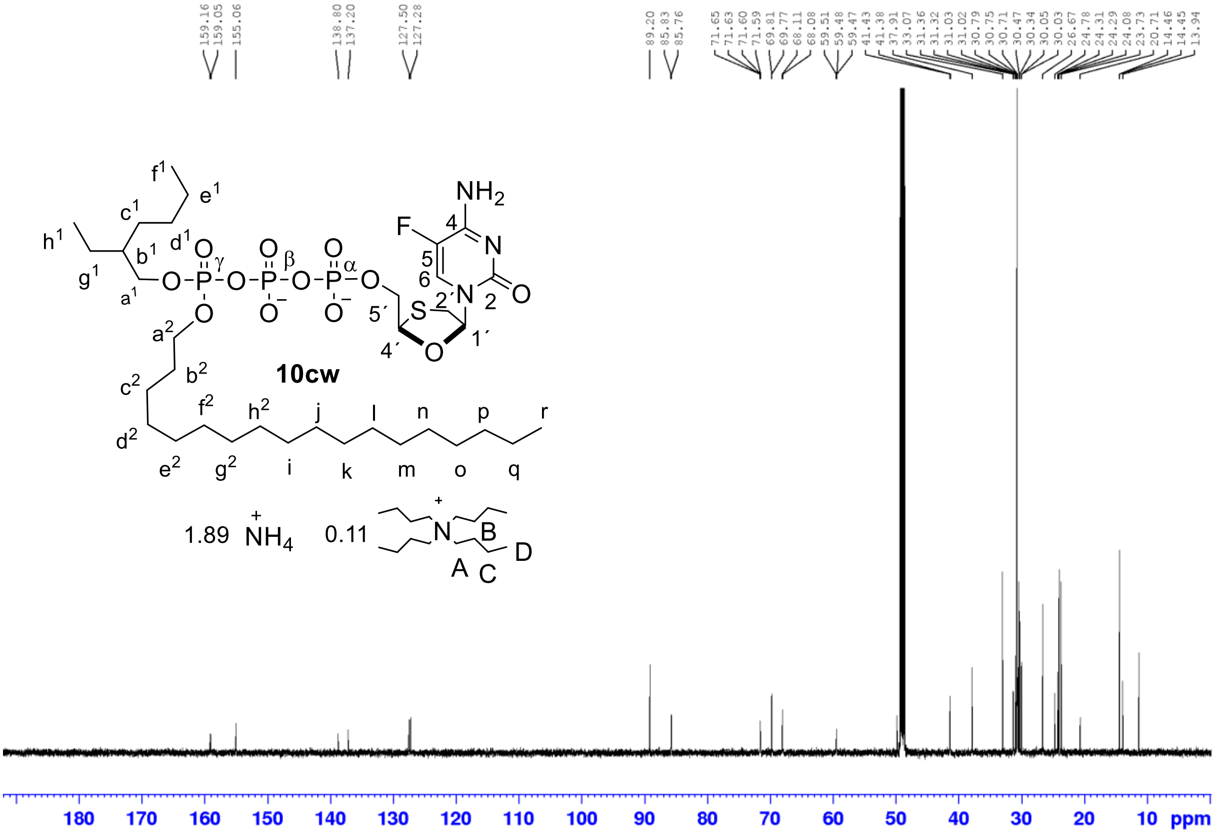
159.16
159.05
155.06
138.80
137.20
127.50
127.28
89.20
85.83
85.76
71.65
71.63
71.60
71.59
69.81
69.77
68.11
68.08
59.51
59.48
59.47
41.43
41.38
37.91
33.07
31.36
31.32
31.03
31.02
30.79
30.75
30.71
30.47
30.34
30.05
30.03
26.67
24.78
24.31
24.29
24.08
23.73
20.71
14.46
14.45
13.94
10cw
1.89 NH4
0.11
180 170 160 150 140 130 120 110 100 90 80 70 60 50 40 30 20 10 ppm

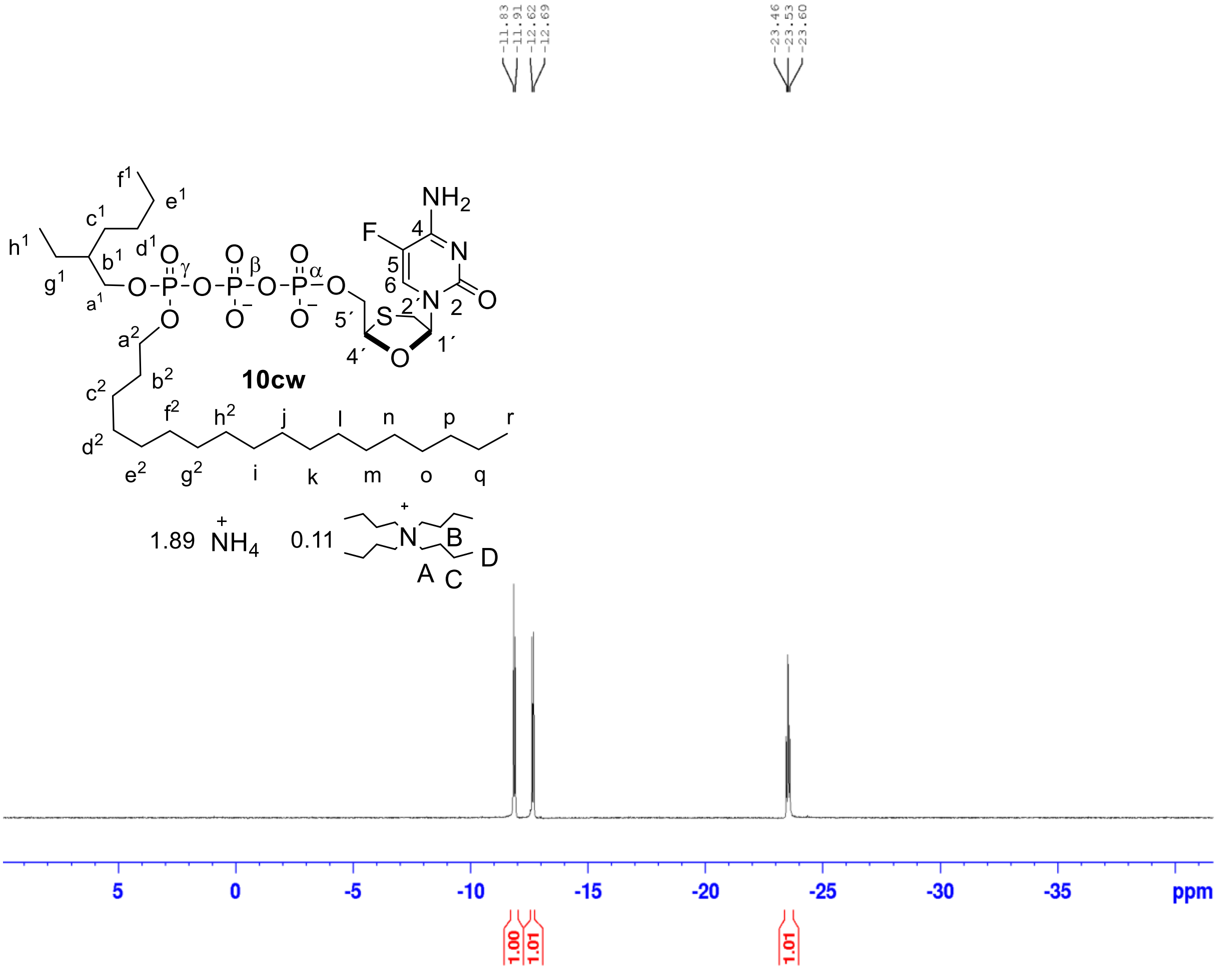
-11.83
-11.91
-12.62
-12.69
-23.46
-23.53
-23.60
10cw
1.89 NH4
0.11
5 0 -5 -10 -15 -20 -25 -30 -35 ppm
1.00
1.01
1.01

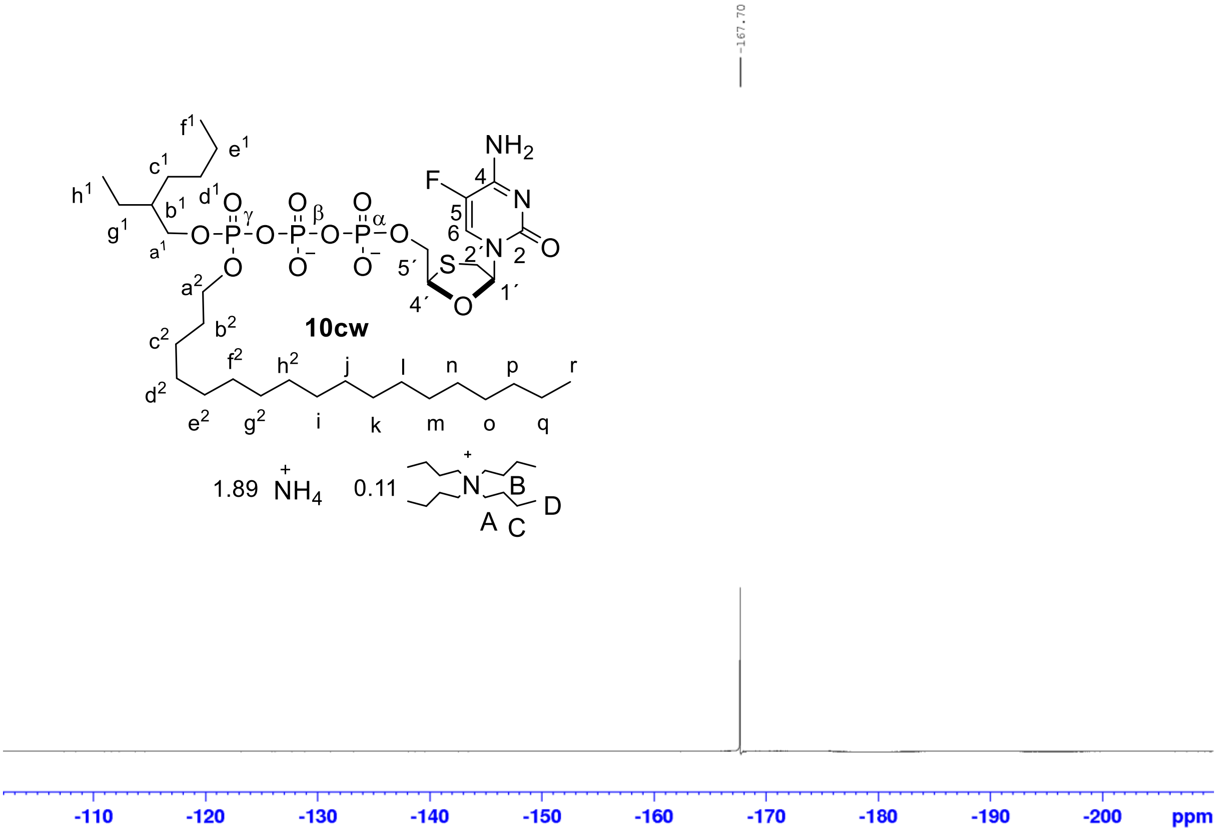

-167.70
10cw
1.89 NH4
0.11
-110 -120 -130 -140 -150 -160 -170 -180 -190 -200 ppm

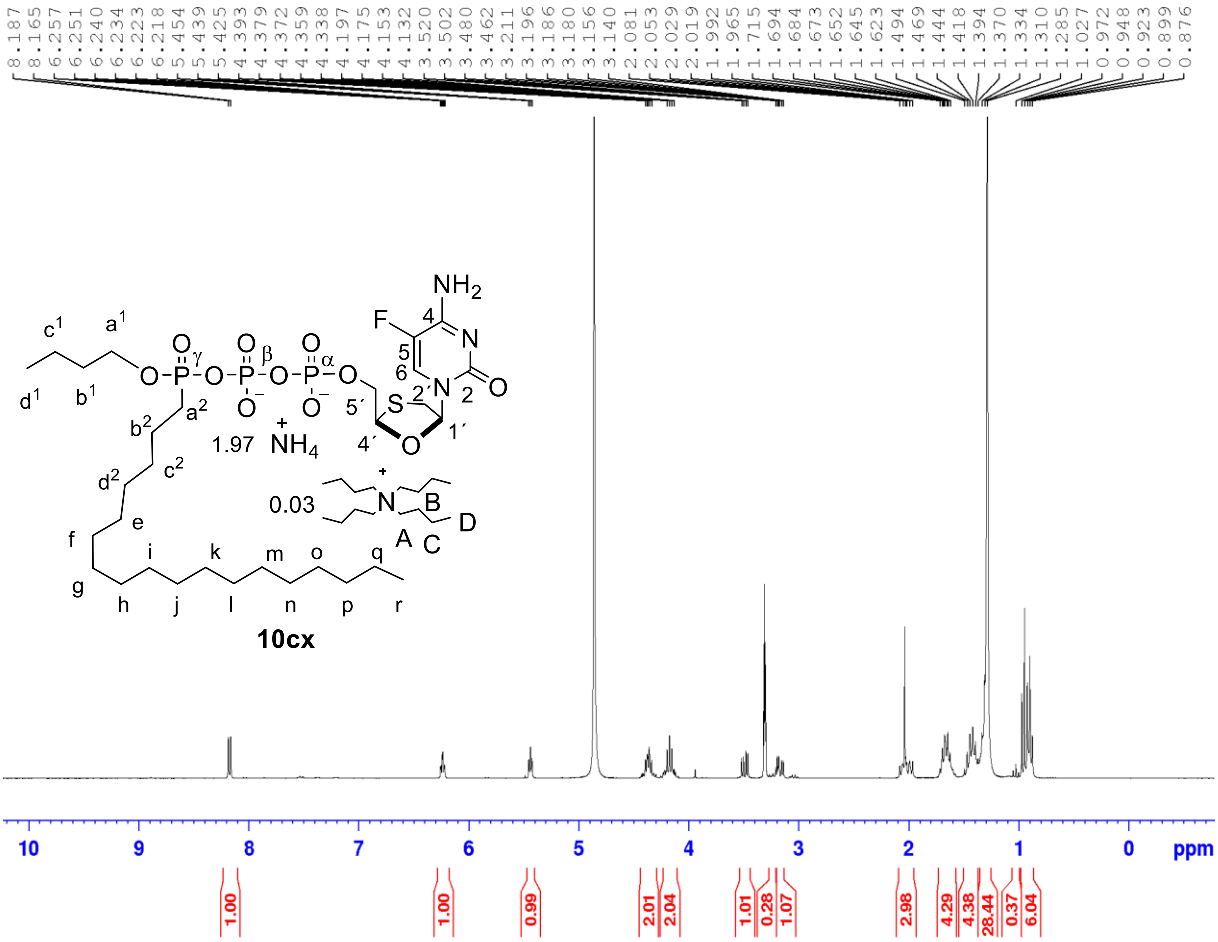

8.187 8.165 6.257 6.251 6.240 6.234 6.223 6.218 5.454 5.439 5.425 4.393 4.379 4.372 4.359 4.338 4.197 4.175 4.153 4.132 3.520 3.502 3.480 3.462 3.211 3.196 3.186 3.180 3.156 3.140 2.081 2.053 2.029 2.019 1.992 1.965 1.715 1.694 1.684 1.673 1.652 1.645 1.623 1.494 1.469 1.444 1.418 1.394 1.370 1.334 1.310 1.285 1.027 0.972 0.948 0.923 0.899 0.876
10cx
1.97 NH4
0.03
10 9 8 7 6 5 4 3 2 1 0 ppm
1.00 1.00 0.99 2.01 2.04 1.01 0.28 1.07 2.98 4.29 4.38 28.44 0.37 6.04

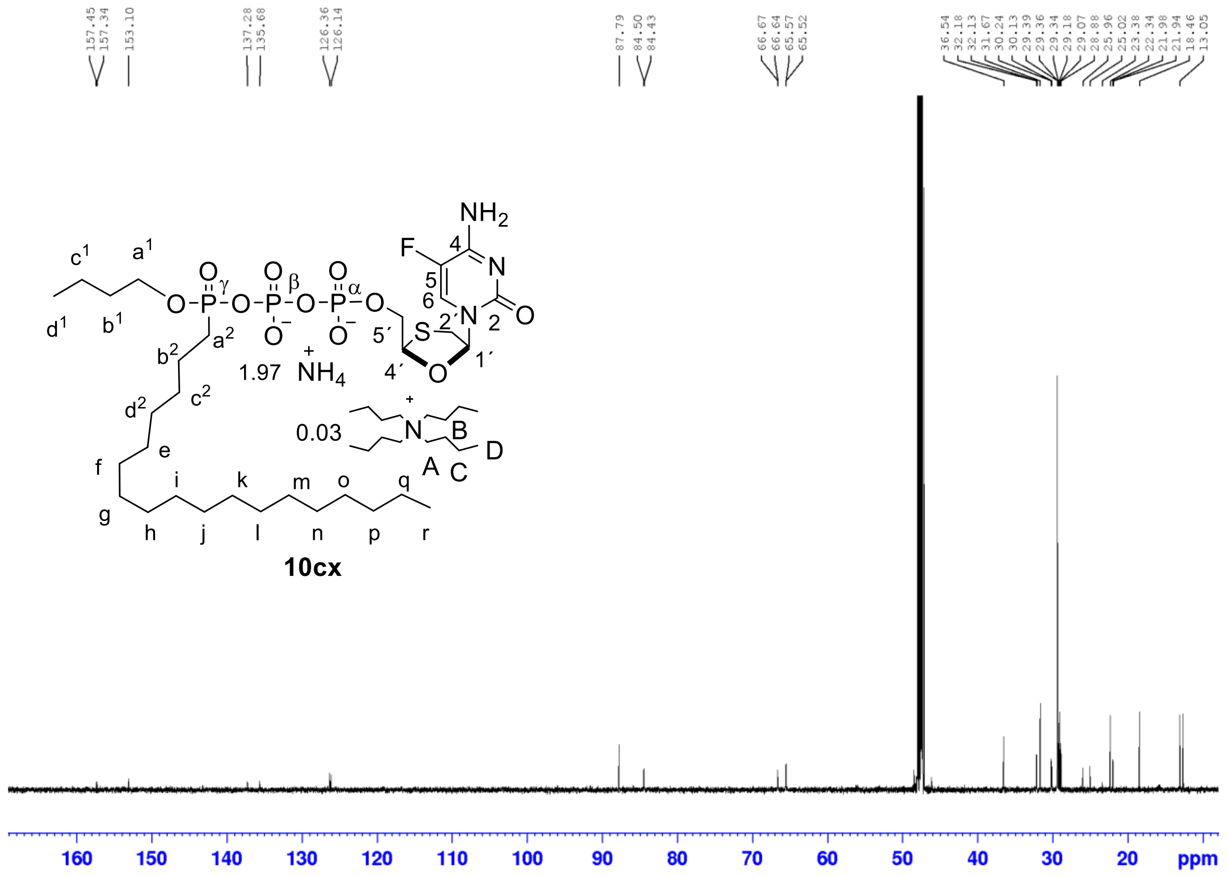

157.45
157.34
153.10
137.28
135.68
126.36
126.14
87.79
84.50
84.43
66.67
66.64
65.57
65.52
36.54
32.18
32.13
31.67
30.24
30.13
29.39
29.36
29.34
29.18
29.07
28.88
25.96
25.02
23.38
22.34
21.98
21.94
18.46
13.05
NH2
F
4
N
5
6
2
O
N
S
2´
5´
1´
4´
O
c1
a1
d1
b1
a2
b2
c2
d2
e
f
g
h
i
j
k
l
m
n
o
p
q
r
γ
β
α
1.97
NH4
0.03
A
B
C
D
10cx
160
150
140
130
120
110
100
90
80
70
60
50
40
30
20
ppm

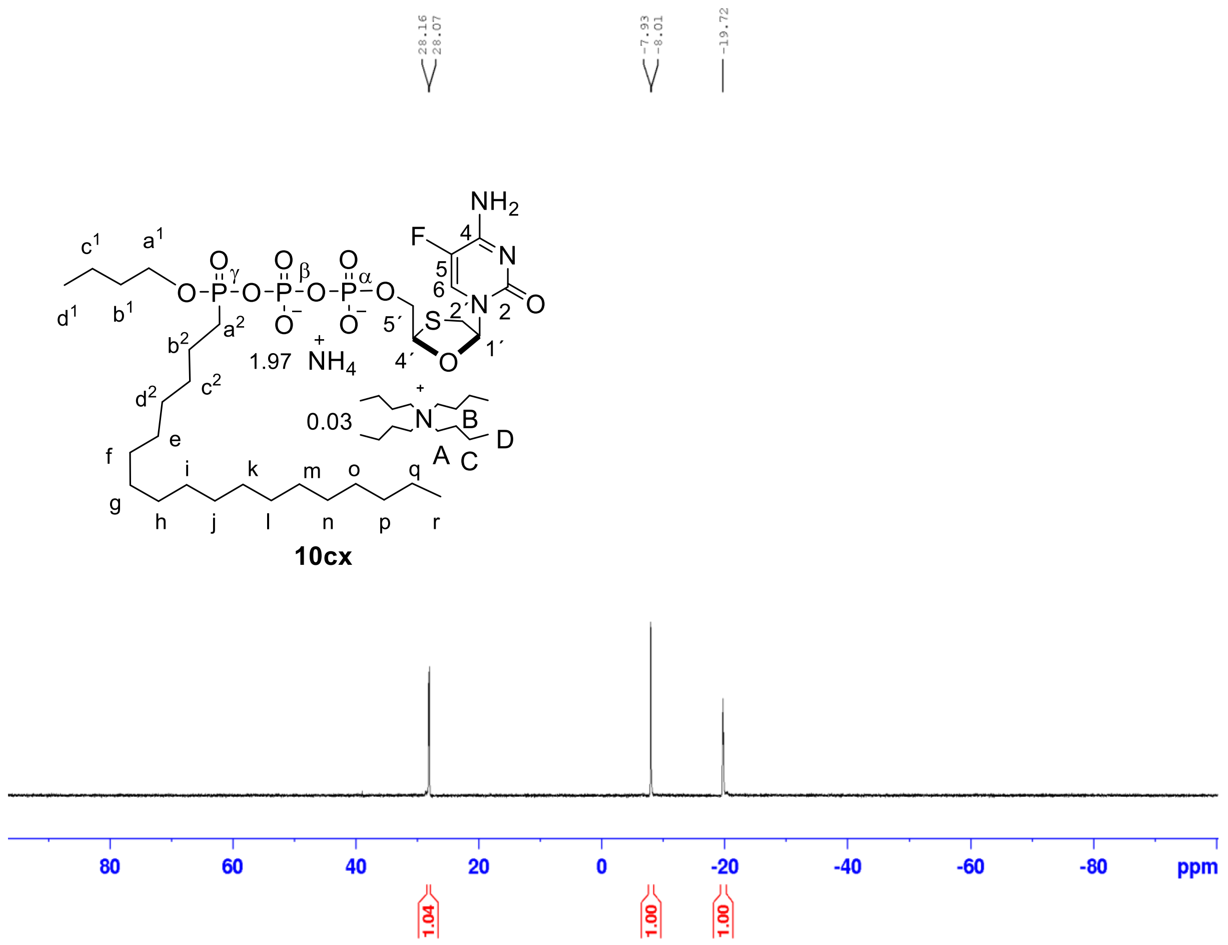

28.16
28.07
-7.93
-8.01
-19.72
NH2
F
4
N
5
6
2
O
N
S
2´
5´
1´
4´
O
c1
a1
d1
b1
a2
b2
c2
d2
e
f
g
h
i
j
k
l
m
n
o
p
q
r
γ
β
α
1.97
NH4
0.03
A
B
C
D
10cx
80
60
40
20
0
-20
-40
-60
-80
ppm
1.04
1.00
1.00

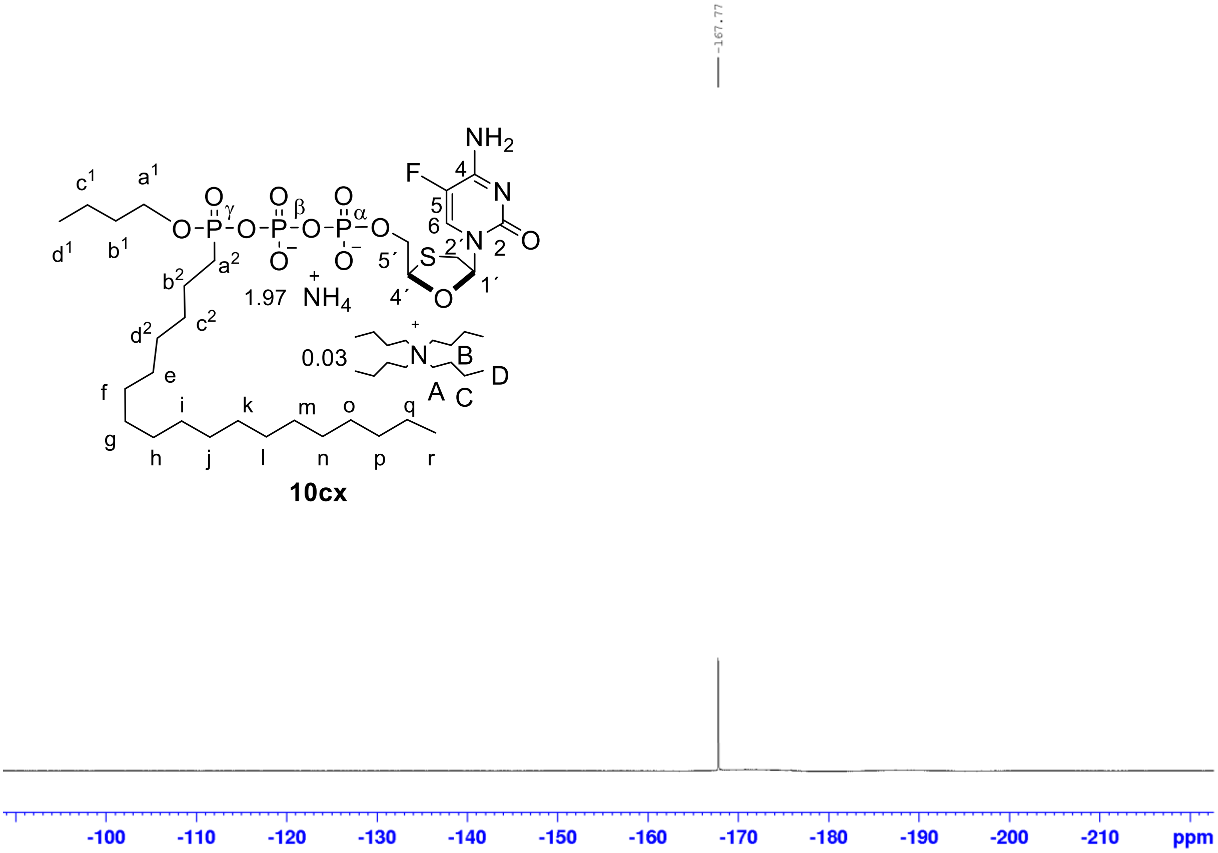
-167.77
1.97 NH4
0.03
ppm

10cx

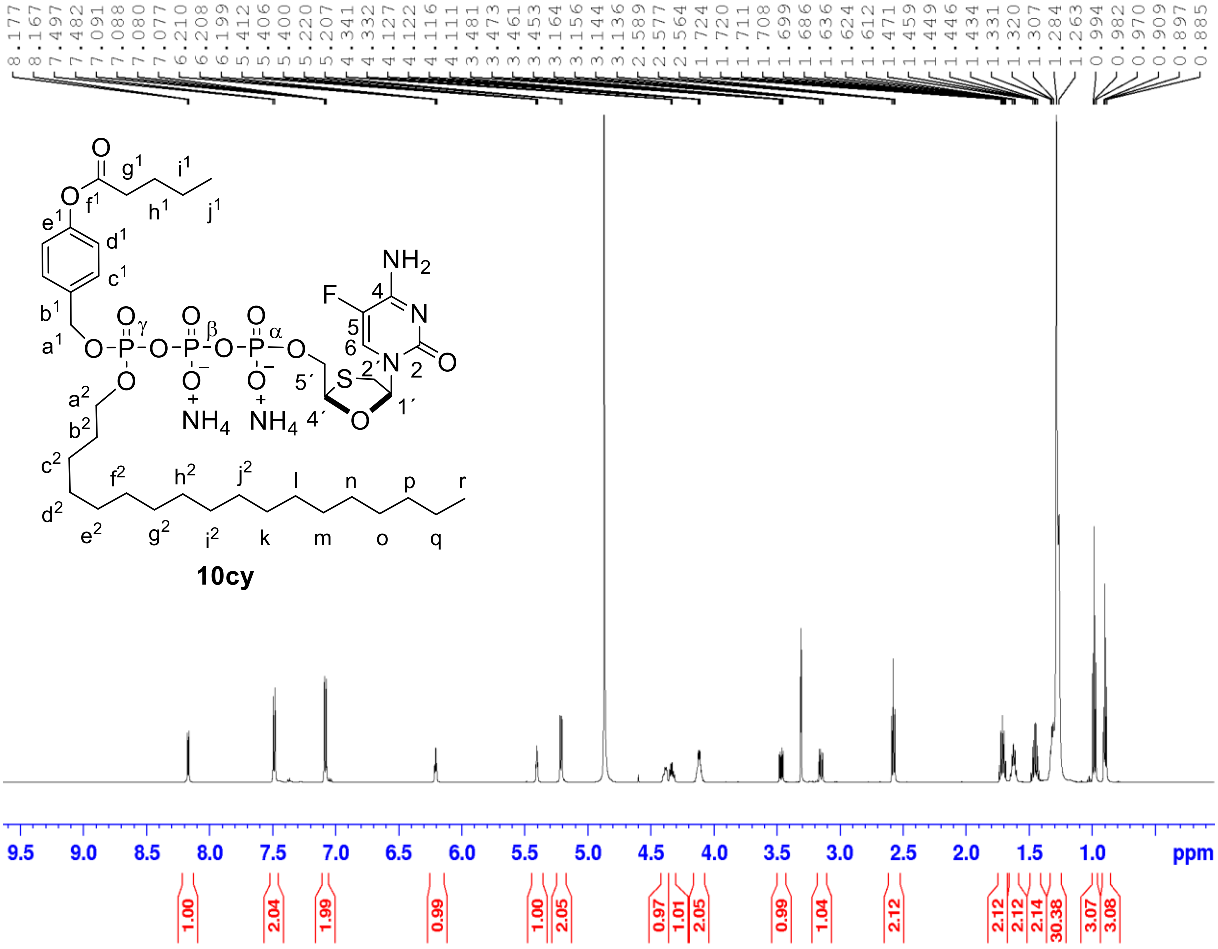
ppm
1.00
2.04
1.99
0.99
1.00
2.05
0.97
1.01
2.05
0.99
1.04
2.12
2.12
2.12
2.14
30.38
3.07
3.08

10cy

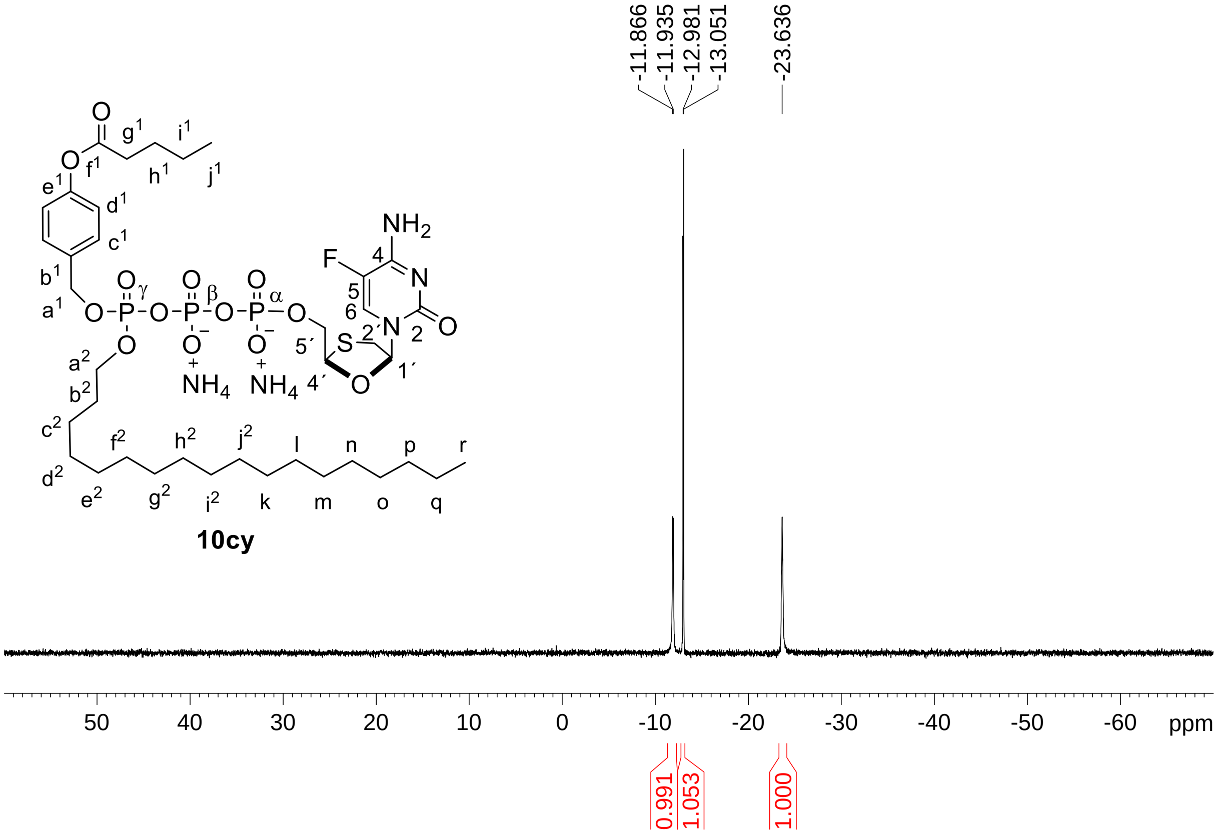
-11.866
-11.935
-12.981
-13.051
-23.636
10cy
0.991
1.053
1.000
ppm

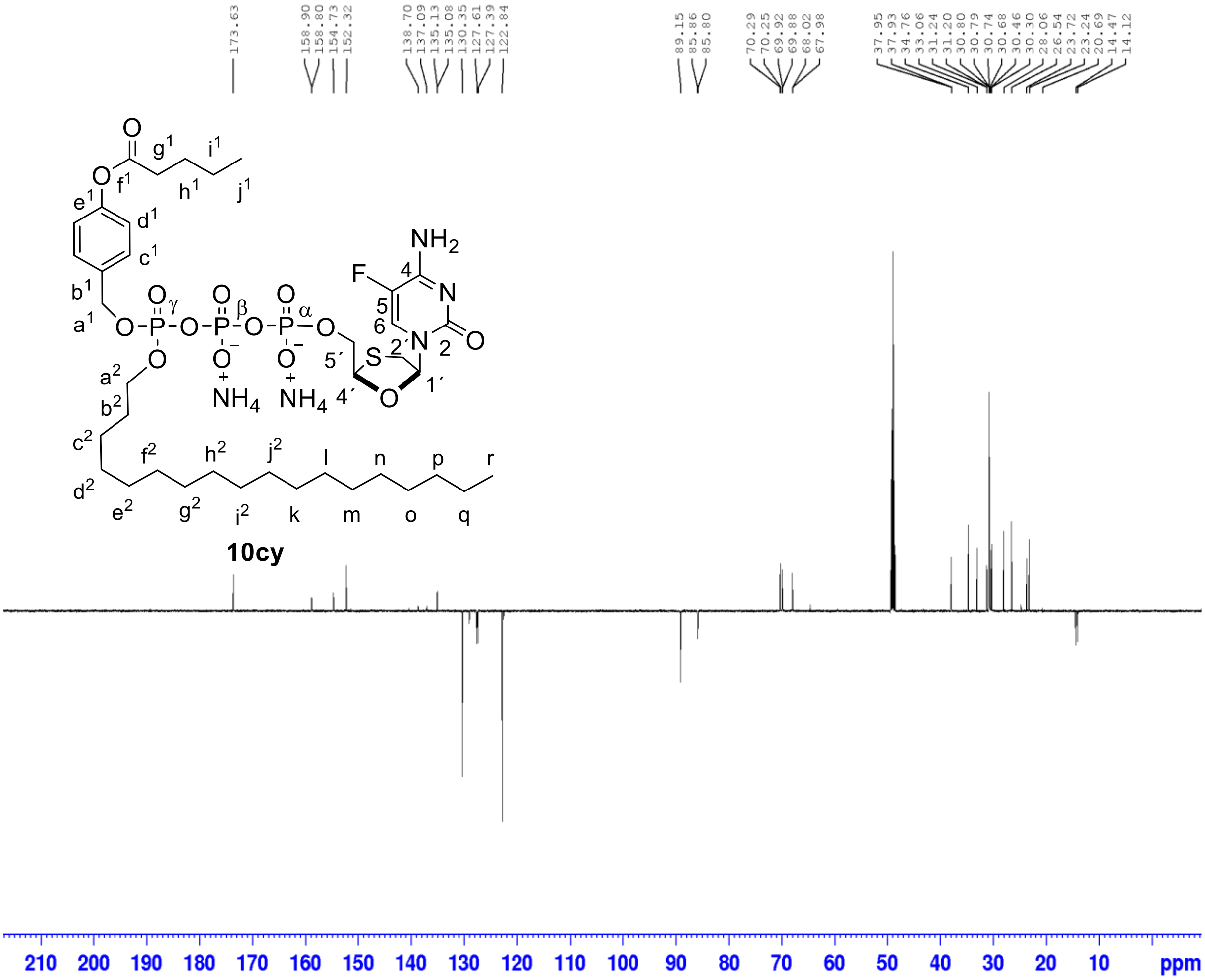
173.63
158.90
158.80
154.73
152.32
138.70
137.09
135.13
135.08
130.35
127.61
127.39
122.84
89.15
85.86
85.80
70.29
70.25
69.92
69.88
68.02
67.98
37.95
37.93
34.76
33.06
31.24
31.20
30.80
30.79
30.74
30.68
30.46
30.30
28.06
26.54
23.72
23.24
20.69
14.47
14.12
10cy
ppm

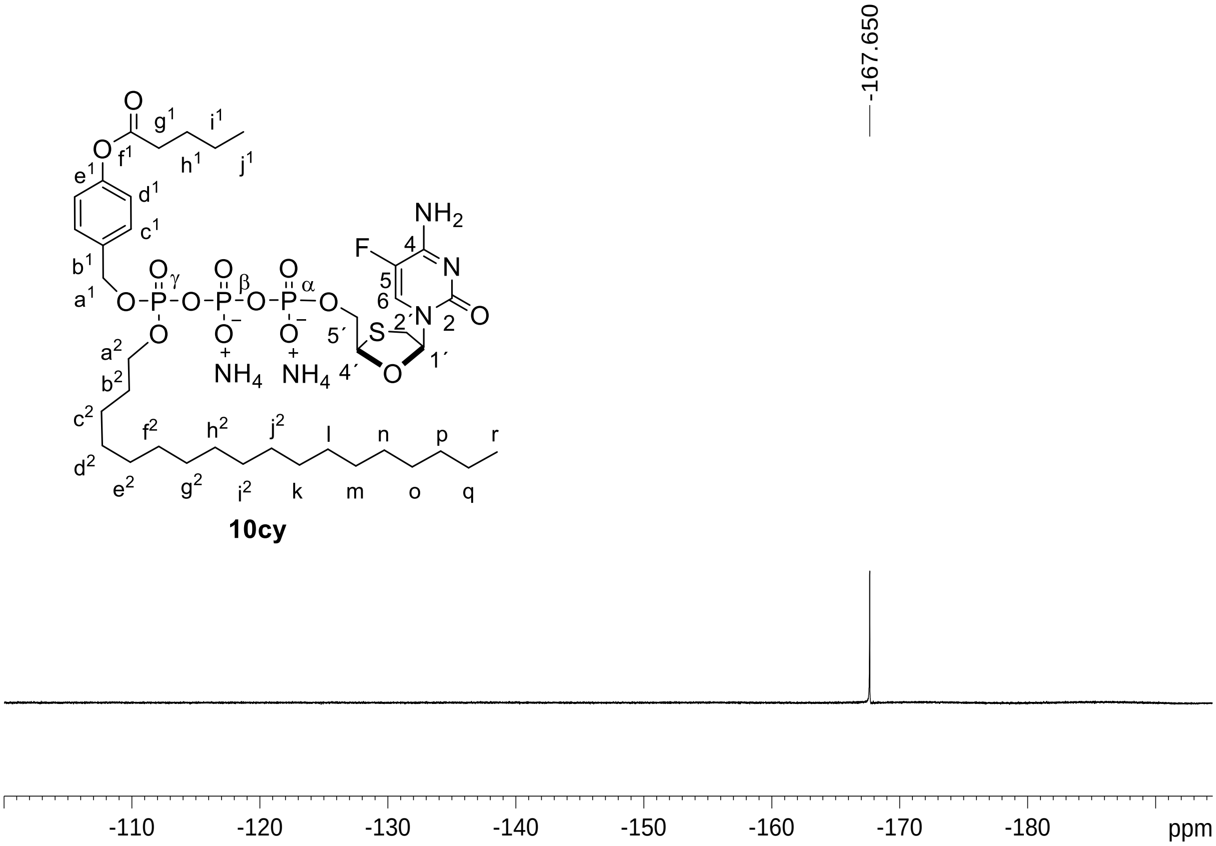
-167.650
10cy
-110
-120
-130
-140
-150
-160
-170
-180
ppm

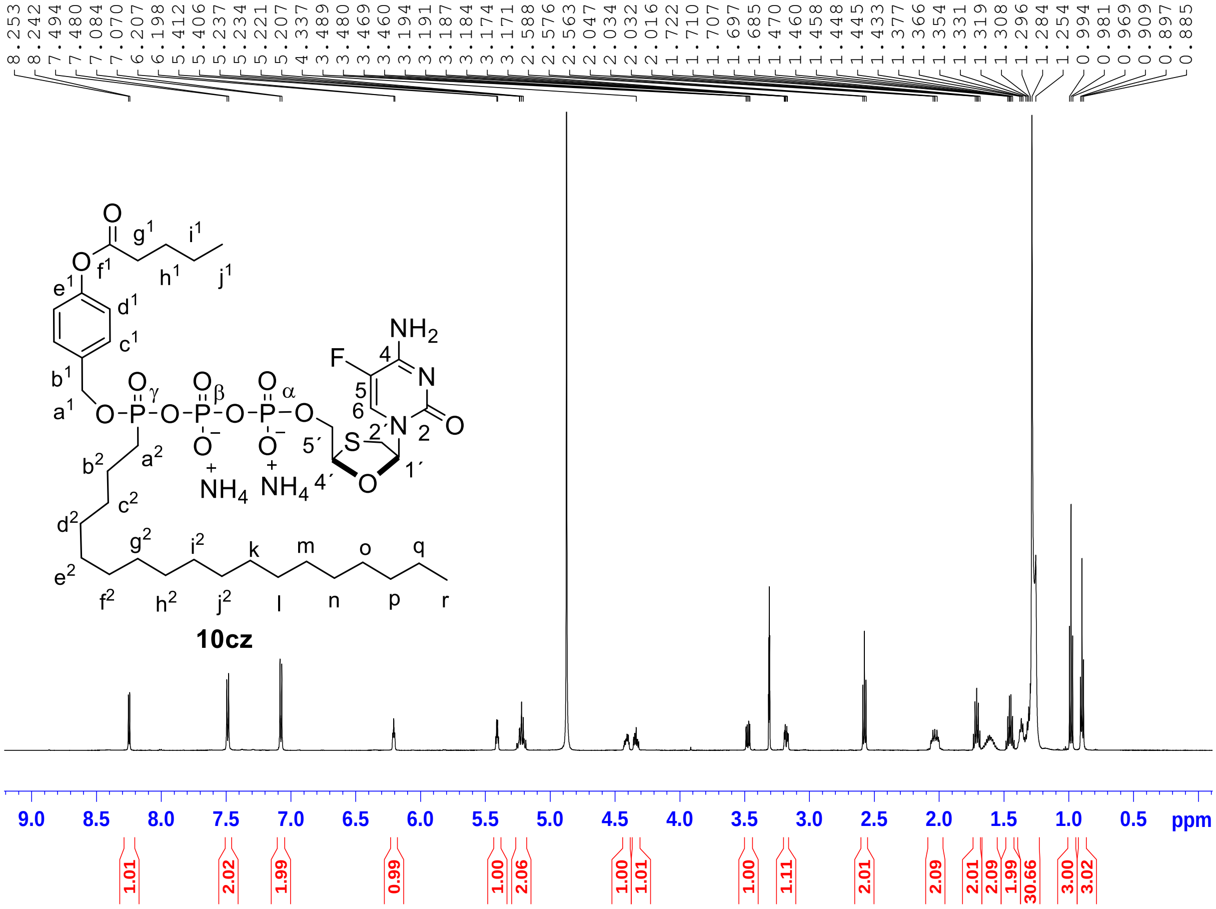
8.253 8.242 7.494 7.480 7.084 7.070 6.207 6.198 5.412 5.406 5.237 5.234 5.221 5.207 4.337 3.489 3.480 3.469 3.460 3.194 3.191 3.187 3.184 3.174 3.171 2.588 2.576 2.563 2.047 2.034 2.032 2.016 1.722 1.710 1.707 1.697 1.685 1.470 1.460 1.458 1.448 1.445 1.433 1.377 1.366 1.354 1.331 1.319 1.308 1.296 1.284 1.254 0.994 0.981 0.969 0.909 0.897 0.885
10cz
9.0 8.5 8.0 7.5 7.0 6.5 6.0 5.5 5.0 4.5 4.0 3.5 3.0 2.5 2.0 1.5 1.0 0.5 ppm
1.01 2.02 1.99 0.99 1.00 2.06 1.00 1.01 1.00 1.11 2.01 2.09 2.01 2.09 1.99 30.66 3.00 3.02

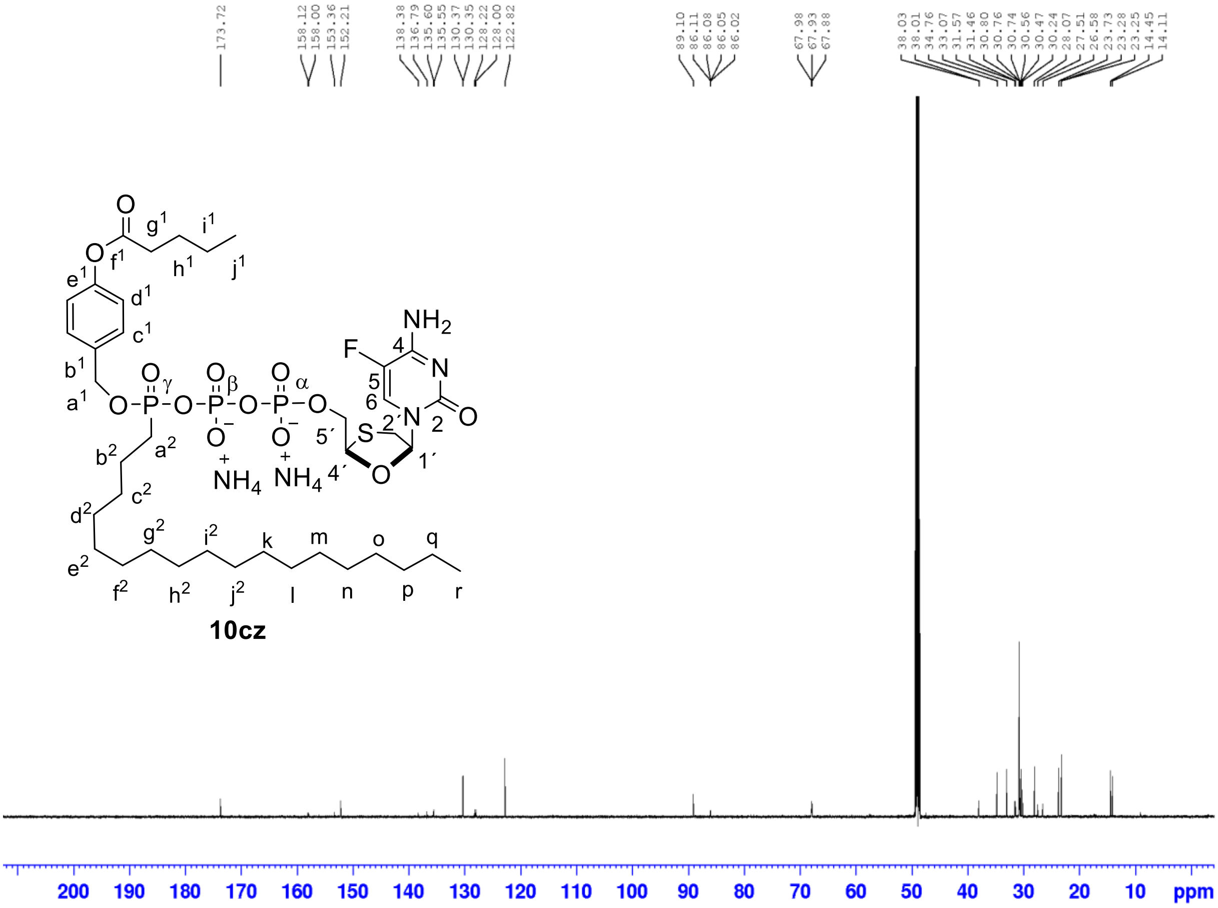
173.72
158.12
158.00
153.36
152.21
138.38
136.79
135.60
135.55
130.37
130.35
128.22
128.00
122.82
89.10
86.11
86.08
86.05
86.02
67.98
67.93
67.88
38.03
38.01
34.76
33.07
31.57
31.46
30.80
30.76
30.74
30.56
30.47
30.24
28.07
27.51
26.58
23.73
23.28
23.25
14.45
14.11
10cz
ppm

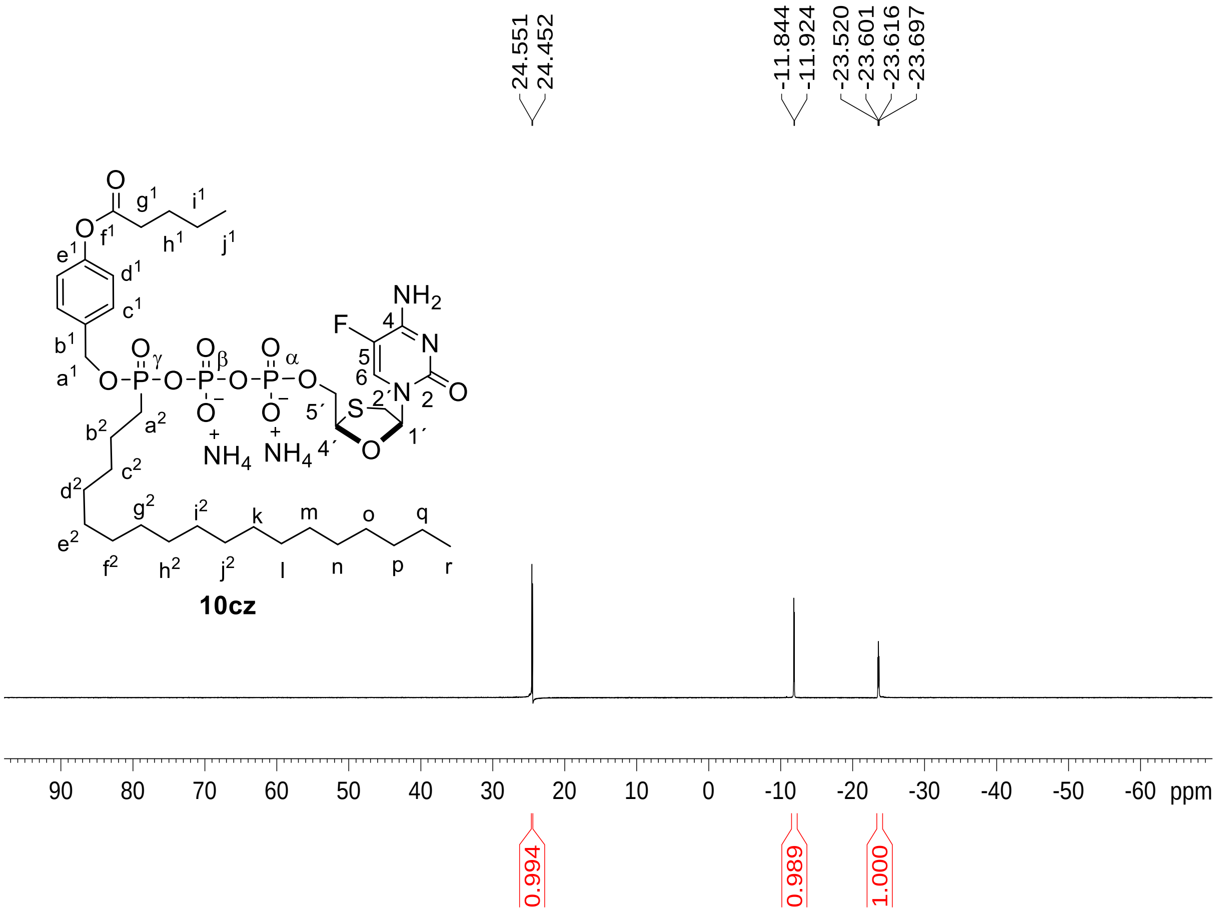
24.551
24.452
-11.844
-11.924
-23.520
-23.601
-23.616
-23.697
10cz
ppm
0.994
0.989
1.000

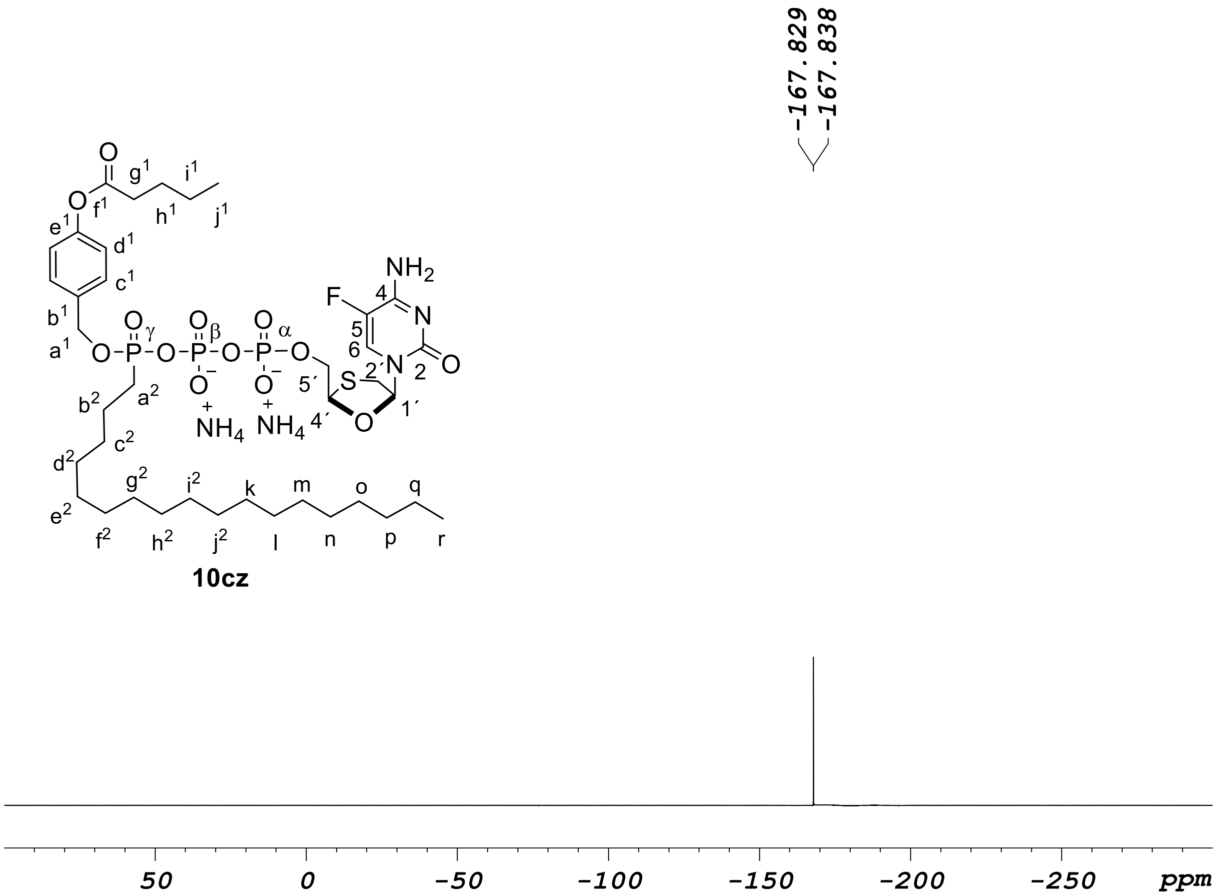
-167.829
-167.838
10cz
NH2
F
O
N
S
NH4
50
0
-50
-100
-150
-200
-250
ppm

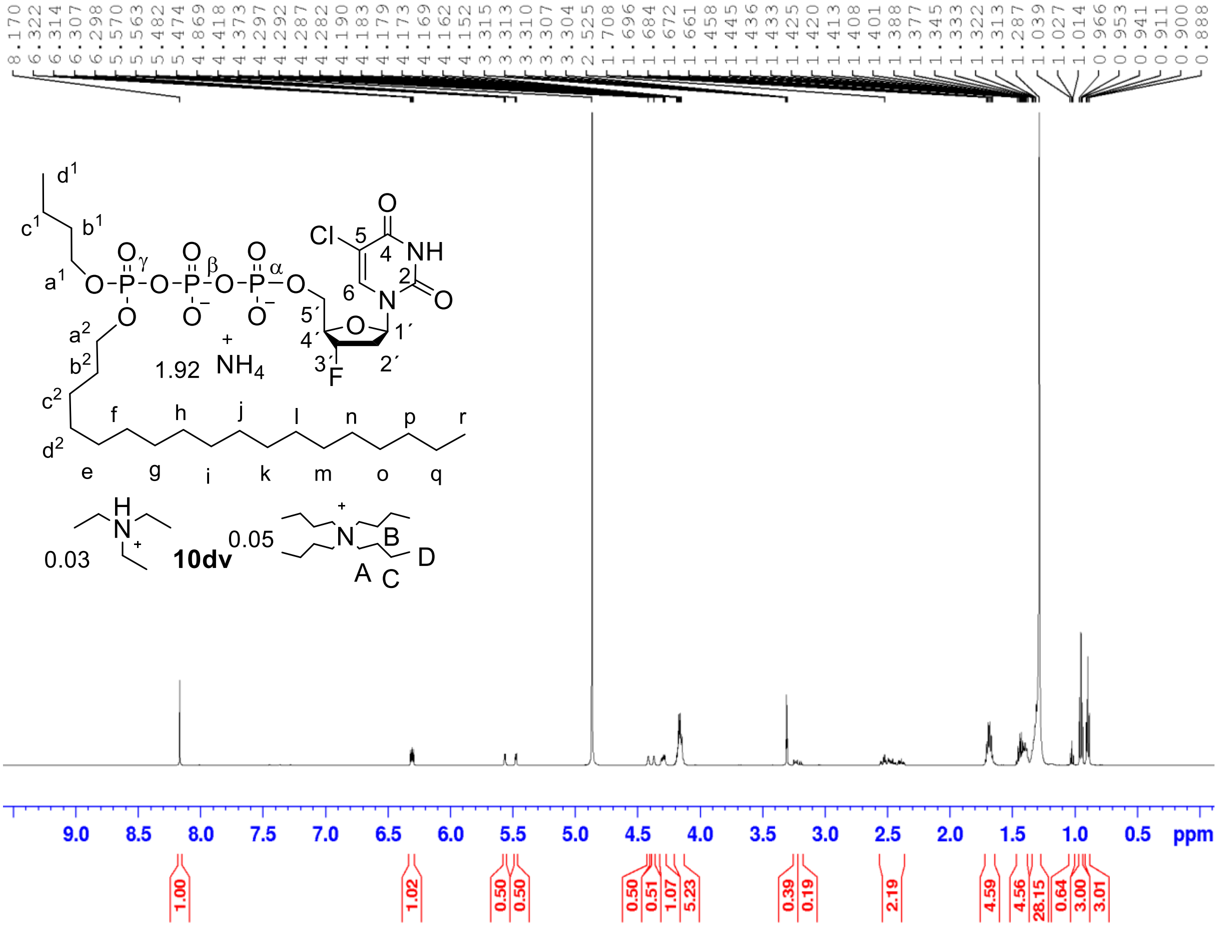
8.170
6.322
6.314
6.307
6.298
5.570
5.563
5.482
5.474
4.869
4.418
4.373
4.297
4.292
4.287
4.282
4.190
4.183
4.179
4.173
4.169
4.162
4.152
3.315
3.313
3.310
3.307
3.304
2.525
1.708
1.696
1.684
1.672
1.661
1.458
1.445
1.436
1.433
1.425
1.420
1.413
1.408
1.401
1.388
1.377
1.345
1.333
1.322
1.313
1.287
1.039
1.027
1.014
0.966
0.953
0.941
0.911
0.900
0.888
Cl
NH
1.92 NH4
0.03
10dv
0.05
9.0
8.5
8.0
7.5
7.0
6.5
6.0
5.5
5.0
4.5
4.0
3.5
3.0
2.5
2.0
1.5
1.0
0.5
ppm
1.00
1.02
0.50
0.50
0.50
0.51
1.07
5.23
0.39
0.19
2.19
4.59
4.56
28.15
0.64
3.00
3.01

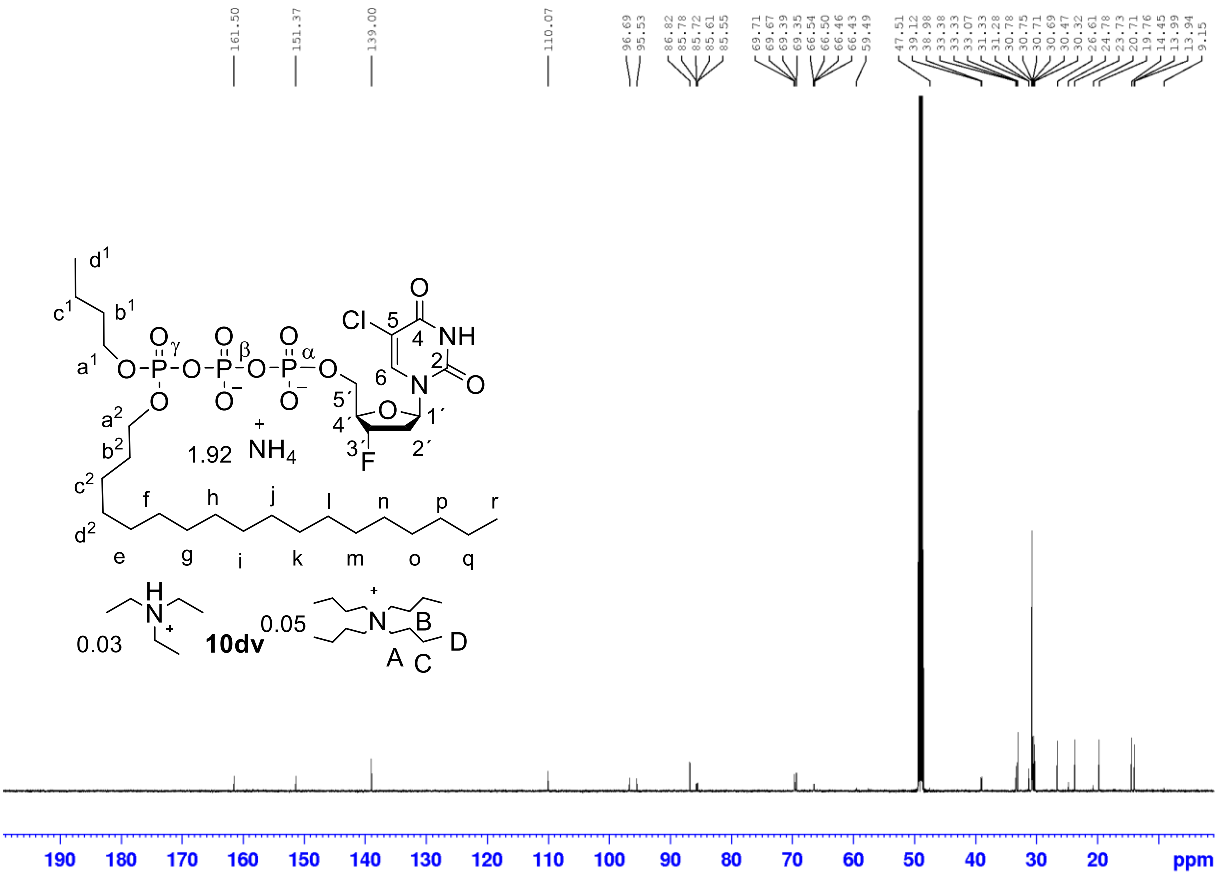

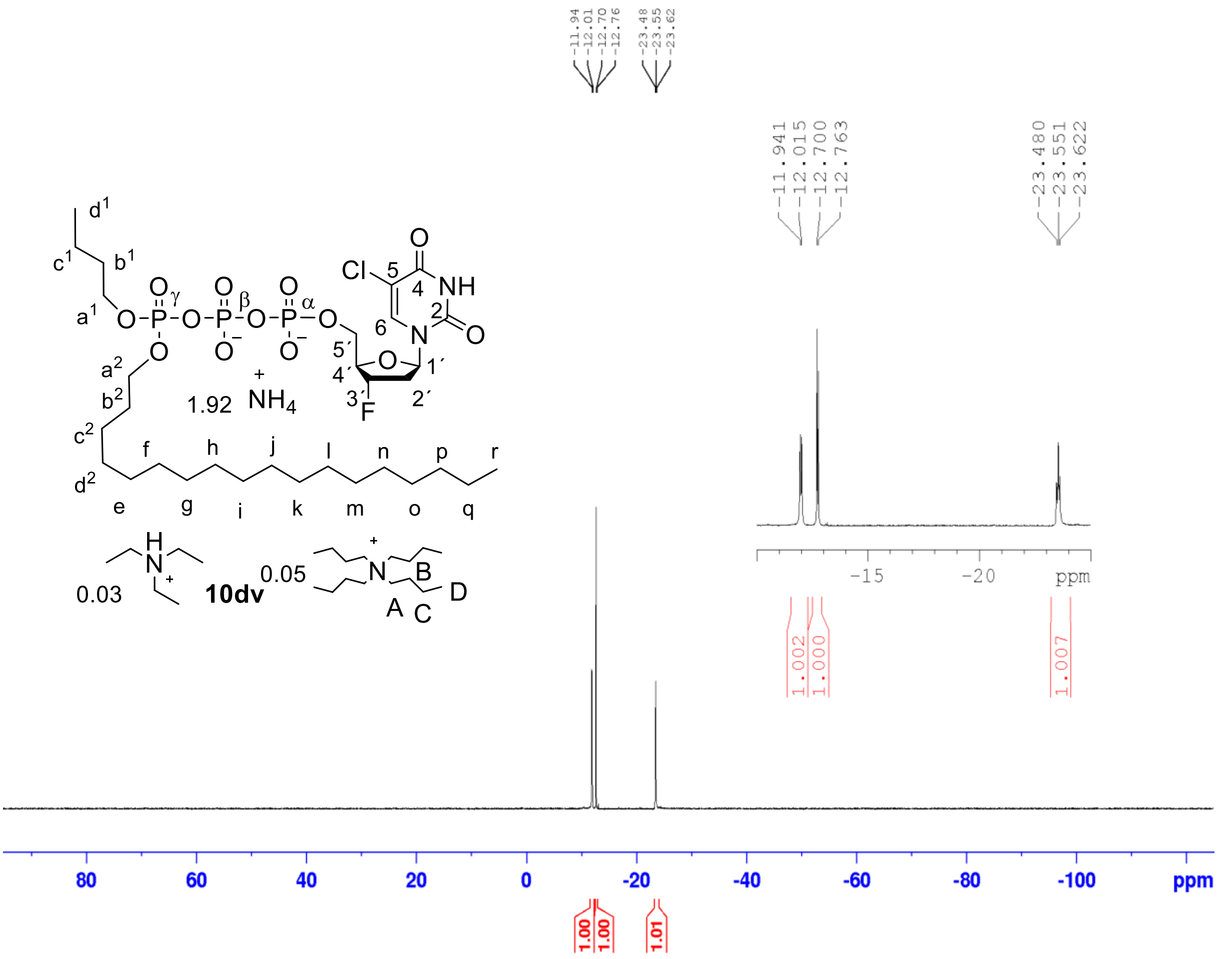

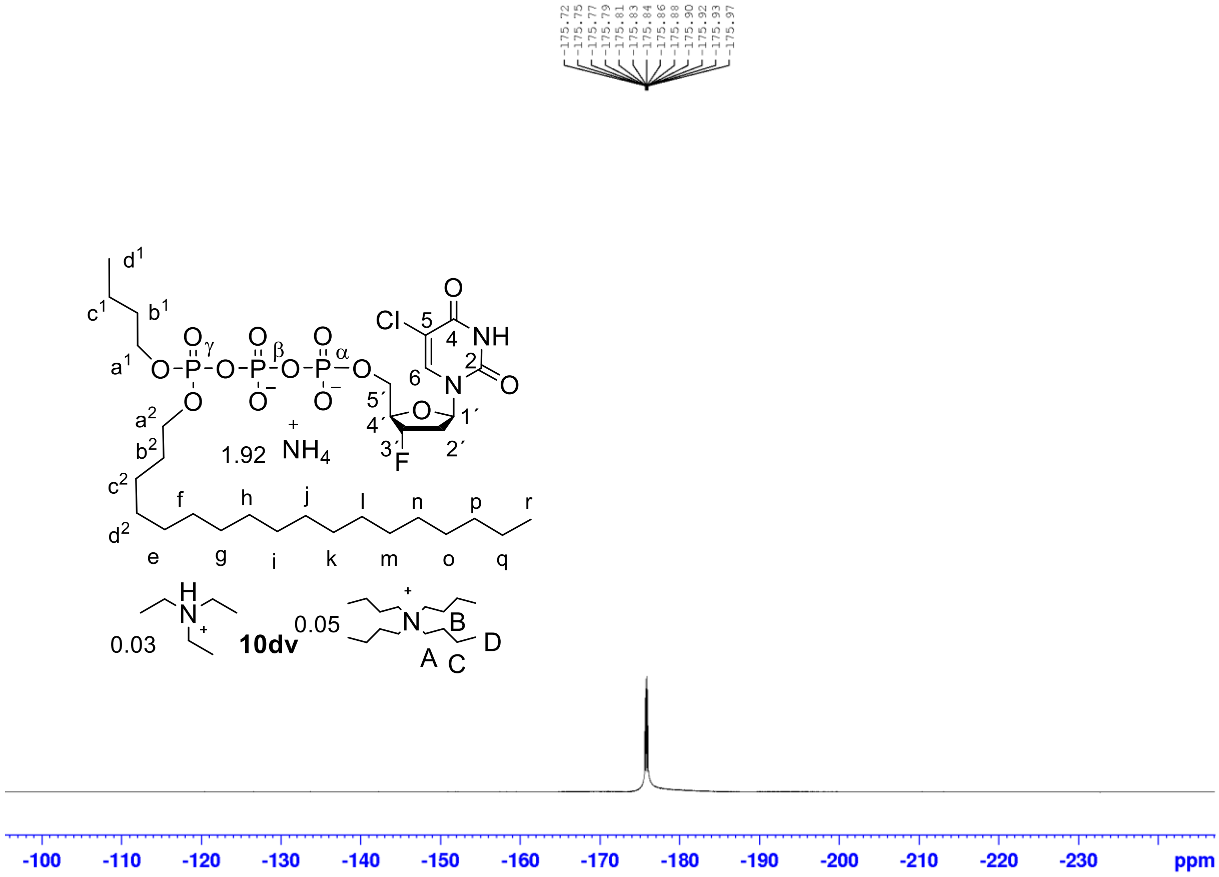
10dv
1.92 NH4
0.03
0.05
ppm

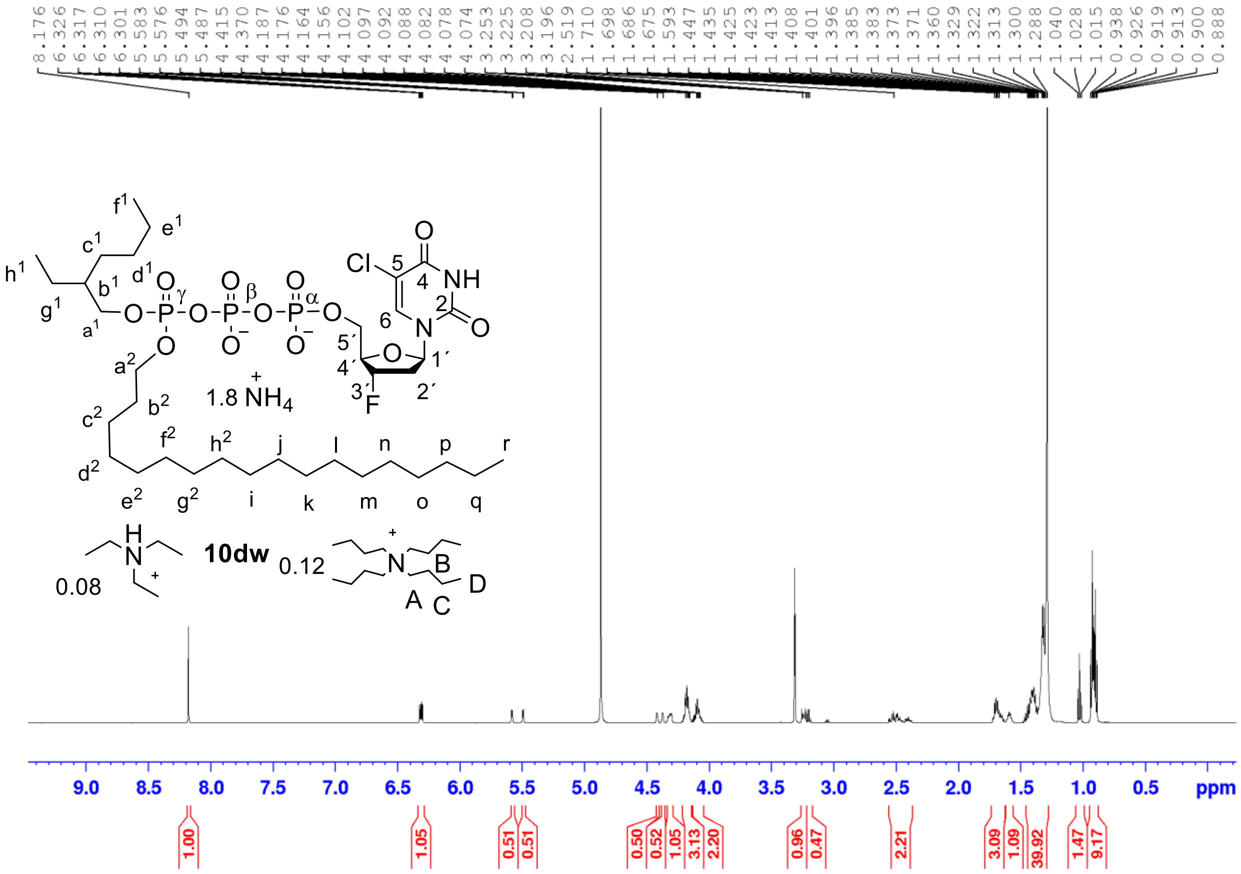
10dw
1.8 NH4
0.08
0.12
ppm

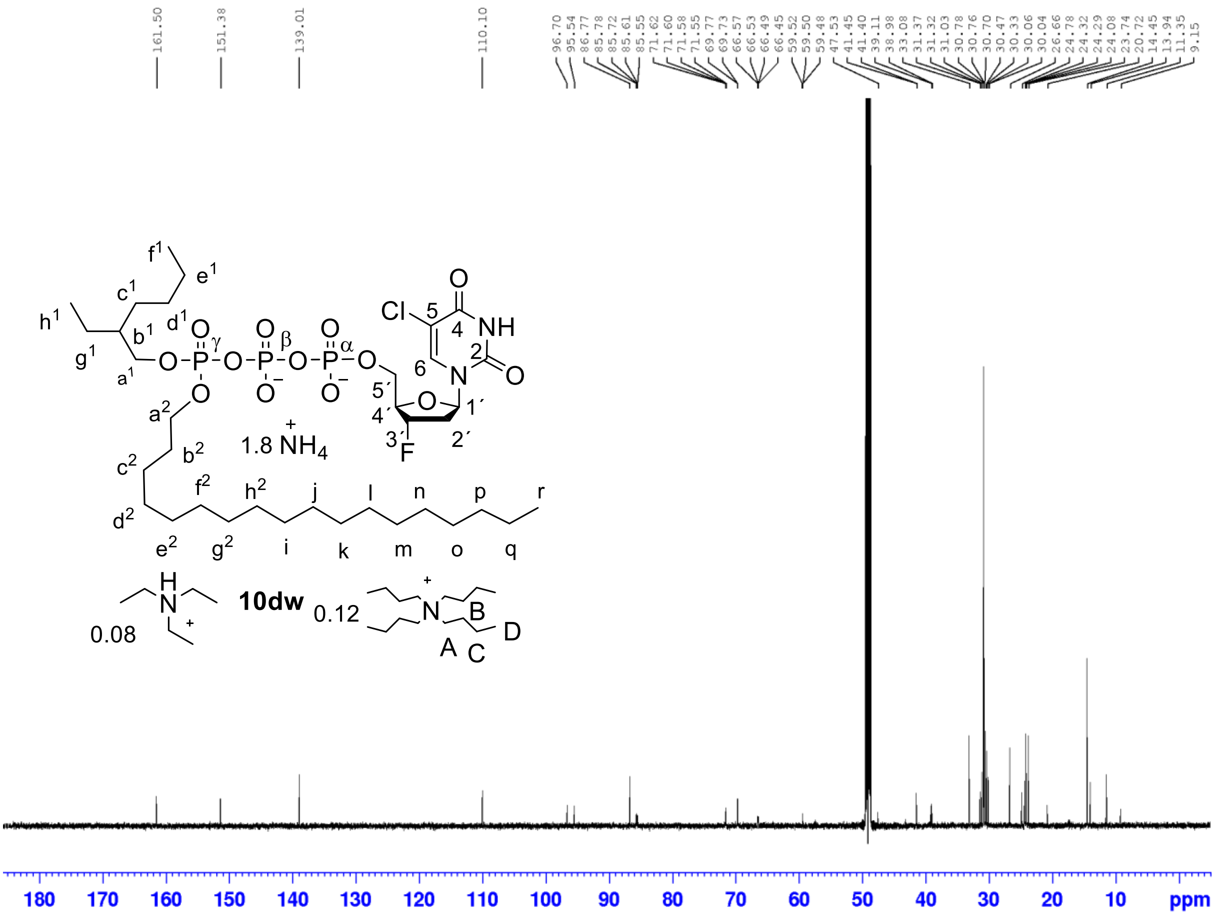
161.50
151.38
139.01
110.10
96.70 95.54 86.77 85.78 85.72 85.61 85.55 71.62 71.60 71.58 71.55 69.77 69.73 66.57 66.53 66.49 66.45 59.52 59.50 59.48 47.53 41.45 41.40 39.11 38.98 33.08 31.37 31.32 31.03 30.78 30.76 30.70 30.47 30.33 30.06 30.04 26.66 24.78 24.32 24.29 24.08 23.74 20.72 14.45 13.94 11.35 9.15
1.8 NH4
10dw
0.08
0.12
180 170 160 150 140 130 120 110 100 90 80 70 60 50 40 30 20 10 ppm

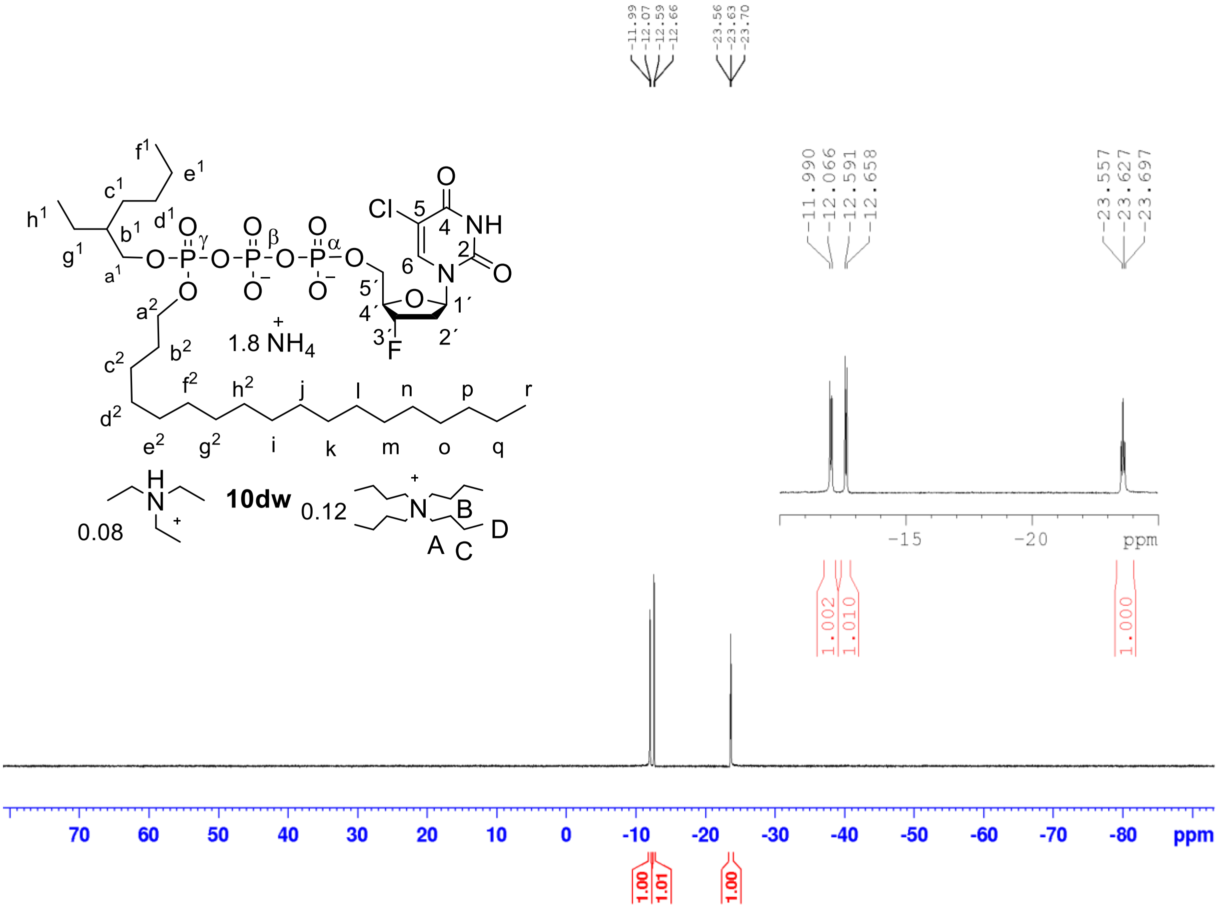
-11.99 -12.07 -12.59 -12.66
-23.56 -23.63 -23.70
-11.990 -12.066 -12.591 -12.658
-23.557 -23.627 -23.697
-15 -20 ppm
1.002 1.010 1.000
1.8 NH4
10dw
0.08
0.12
70 60 50 40 30 20 10 0 -10 -20 -30 -40 -50 -60 -70 -80 ppm
1.00 1.01 1.00

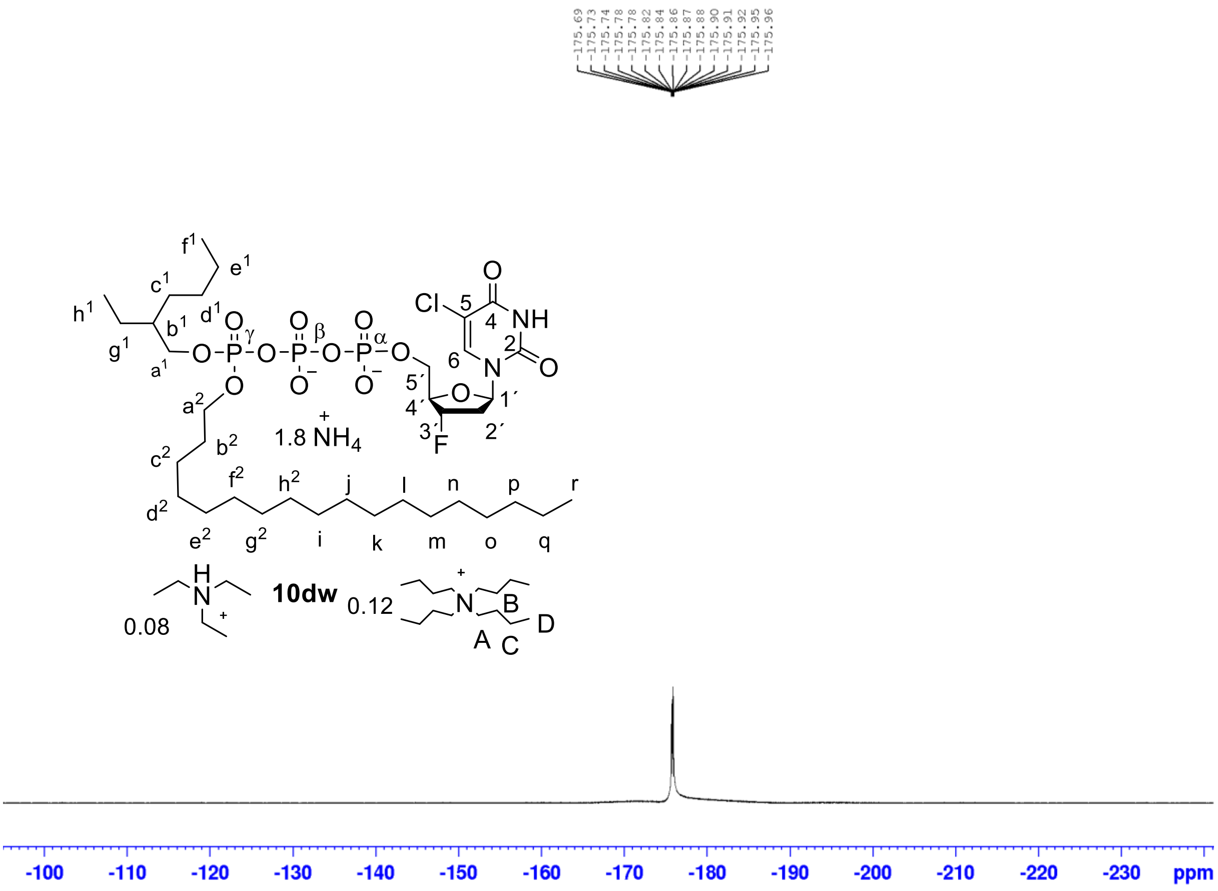
10dw
1.8 NH4
0.08
0.12
ppm

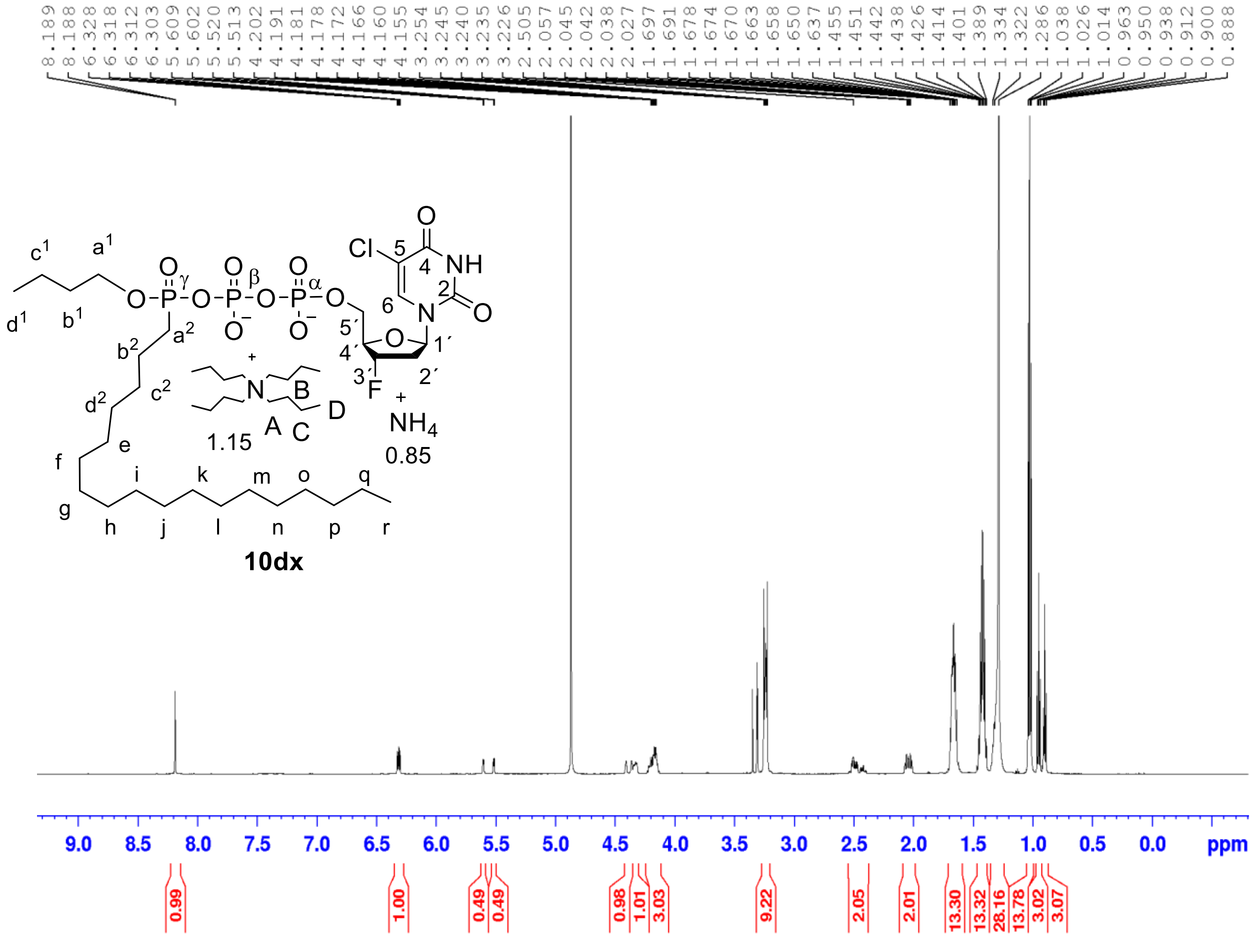
10dx
1.15
NH4
0.85
ppm

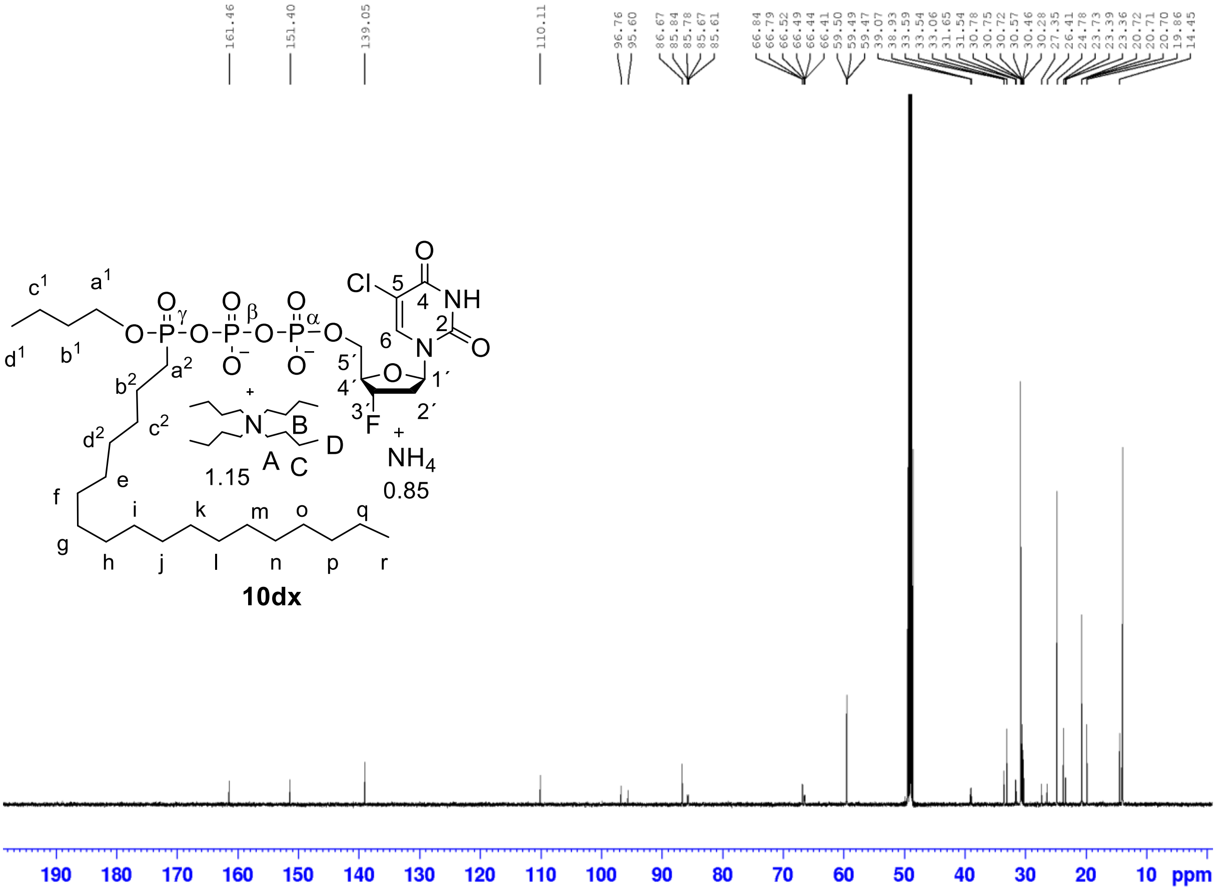
161.46
151.40
139.05
110.11
96.76
95.60
86.67
85.84
85.78
85.67
85.61
66.84
66.79
66.52
66.49
66.44
66.41
59.50
59.49
59.47
39.07
38.93
33.59
33.54
33.06
31.65
31.54
30.78
30.75
30.72
30.57
30.46
30.28
27.35
26.41
24.78
23.73
23.39
23.36
20.72
20.71
20.70
19.86
14.45
10dx
ppm

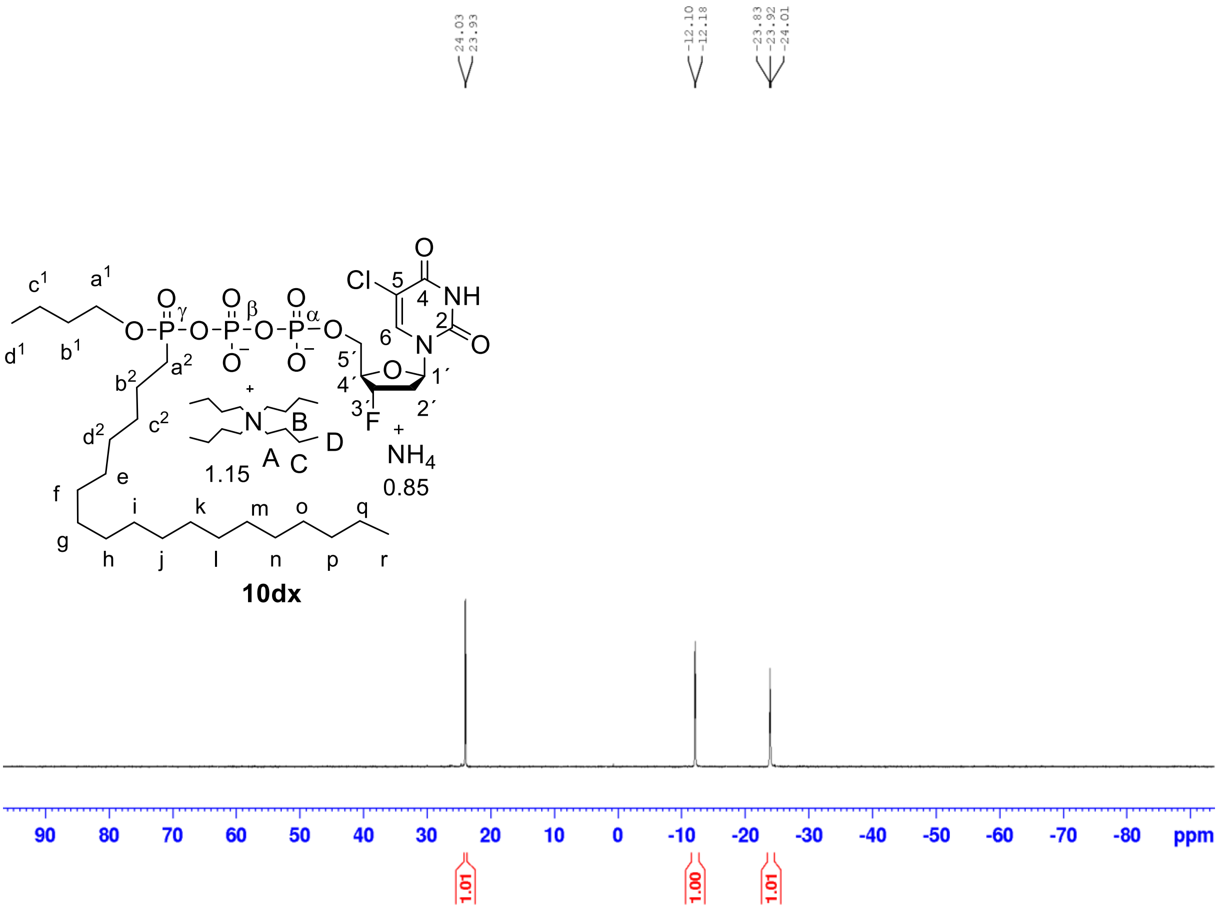
24.03
23.93
-12.10
-12.18
-23.83
-23.92
-24.01
10dx
ppm
1.01
1.00
1.01

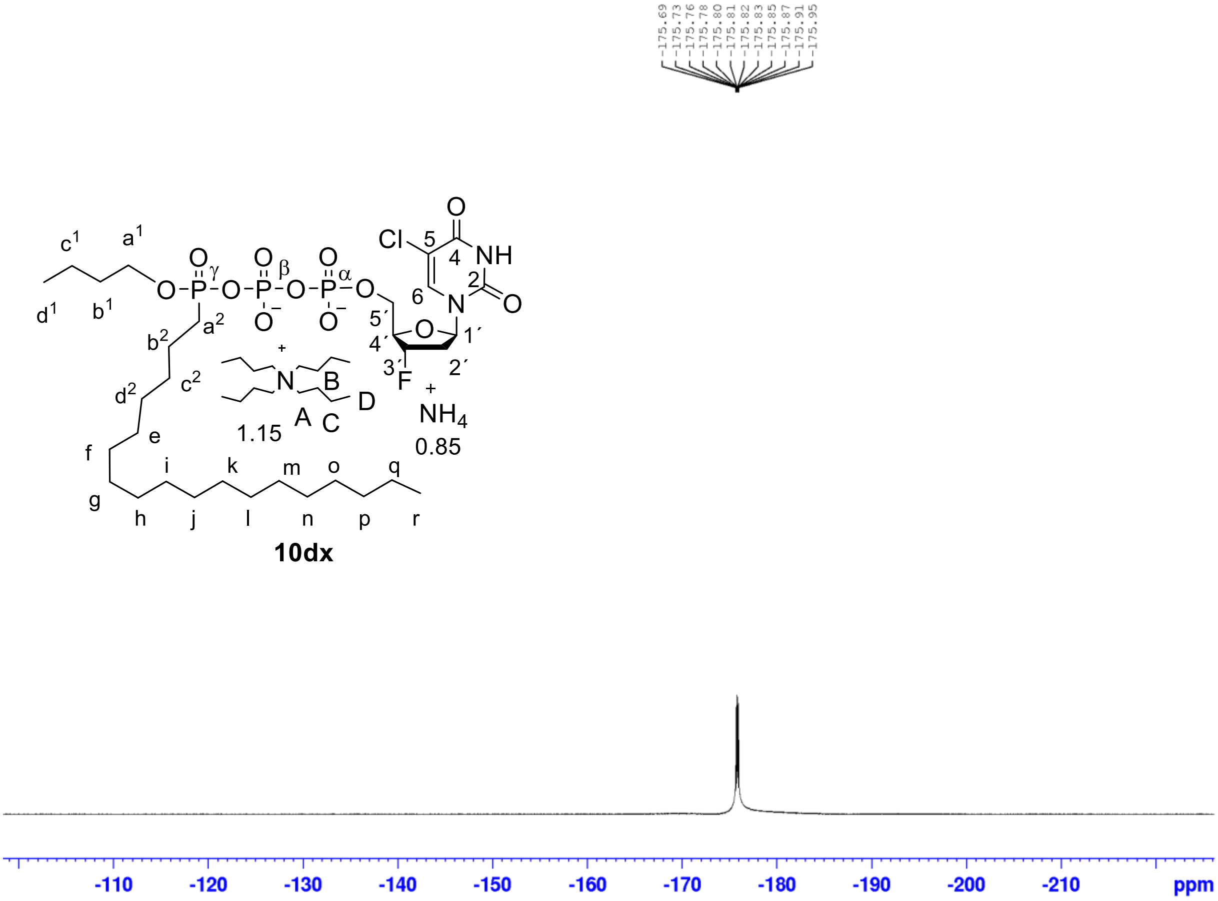

10dx

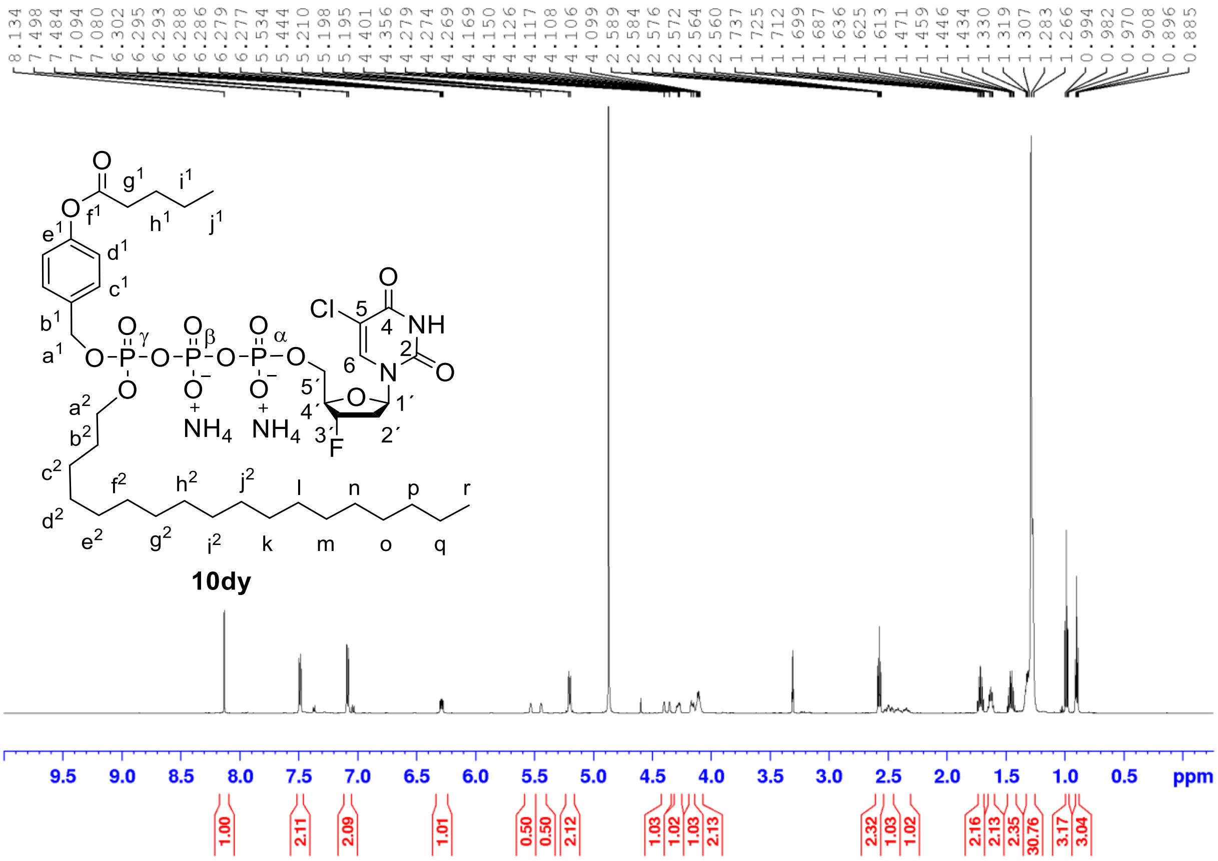

10dy

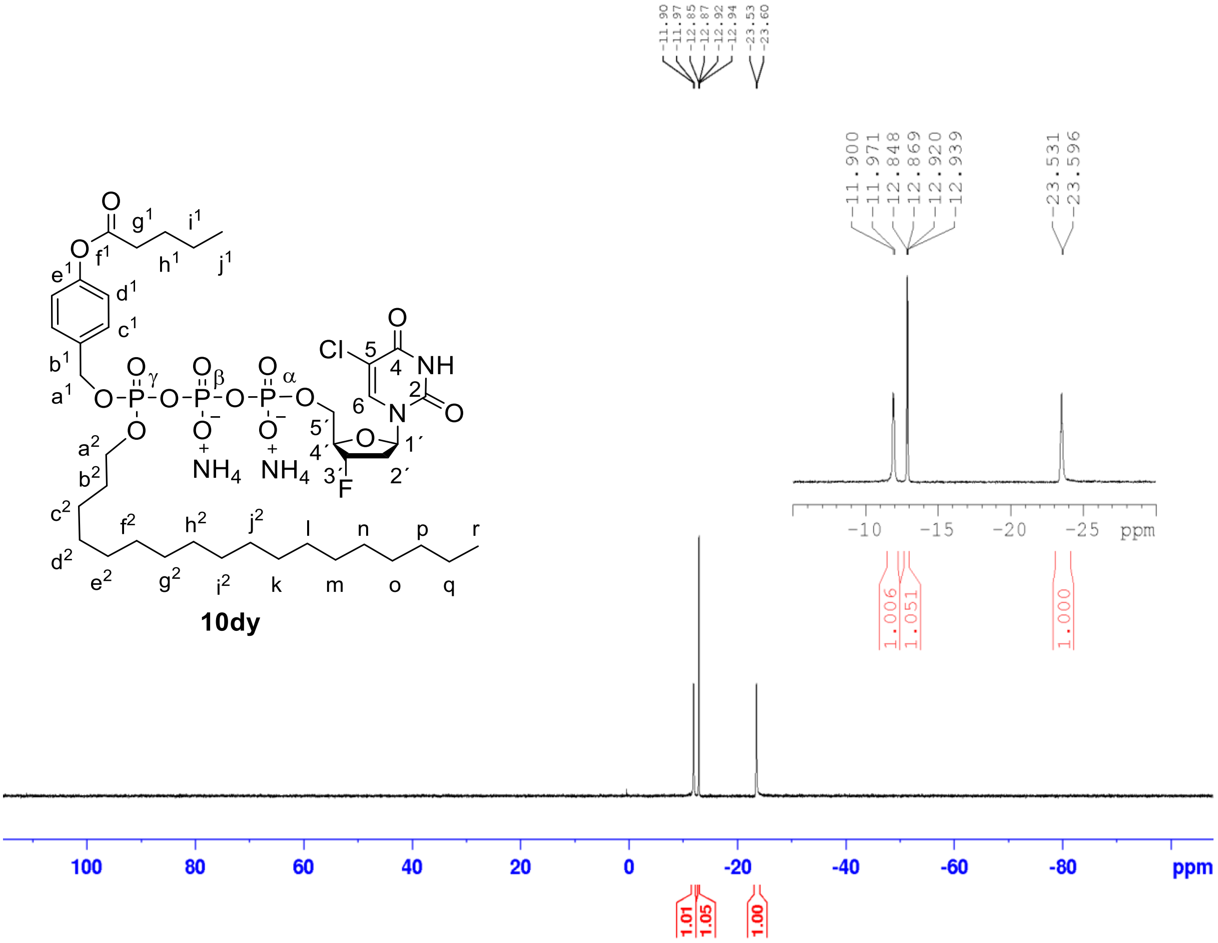
-11.90
-11.97
-12.85
-12.87
-12.92
-12.94
-23.53
-23.60
-11.900
-11.971
-12.848
-12.869
-12.920
-12.939
-23.531
-23.596
1.006
1.051
1.000
-10 -15 -20 -25 ppm
100 80 60 40 20 0 -20 -40 -60 -80 ppm
1.01
1.05
1.00

10dy

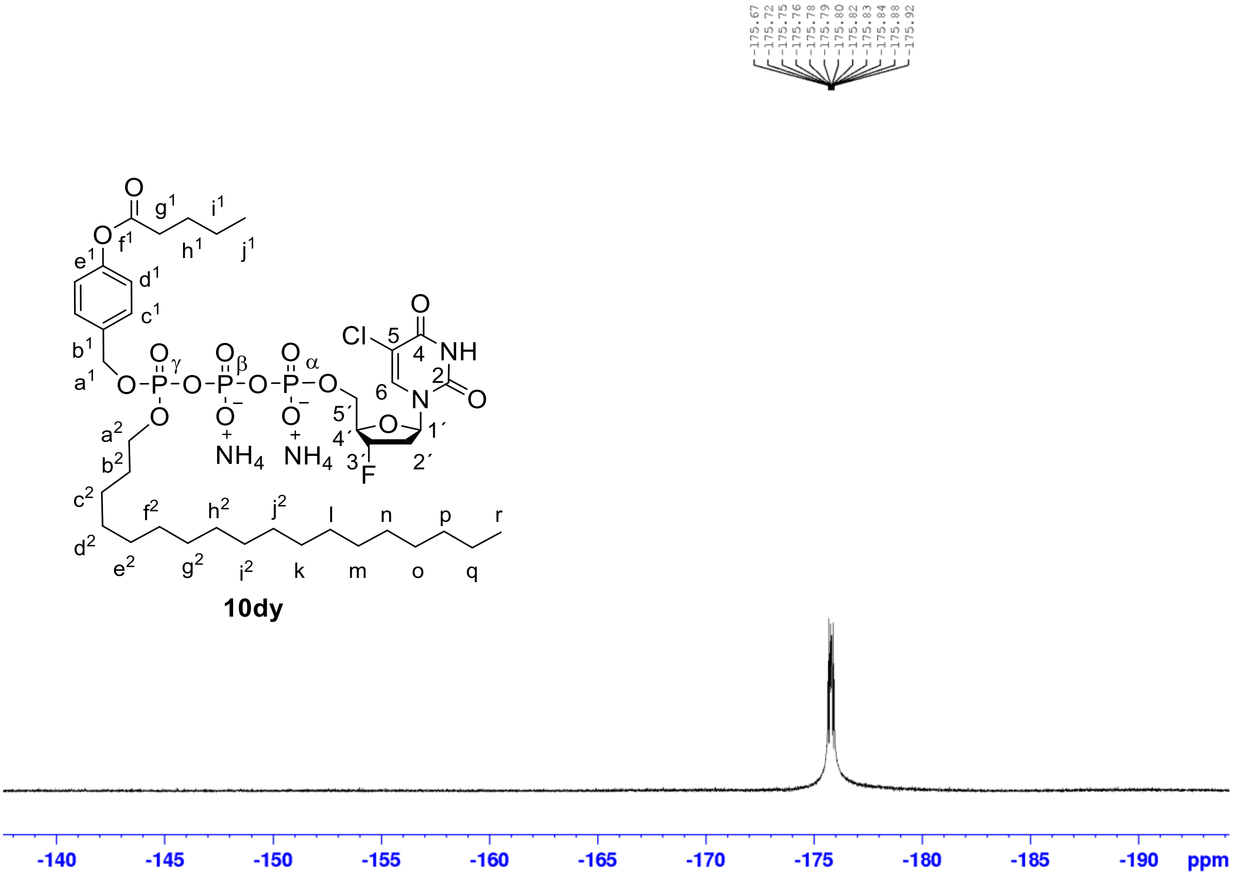
-175.67
-175.72
-175.75
-175.76
-175.78
-175.79
-175.80
-175.82
-175.83
-175.84
-175.88
-175.92
-140 -145 -150 -155 -160 -165 -170 -175 -180 -185 -190 ppm

10dy

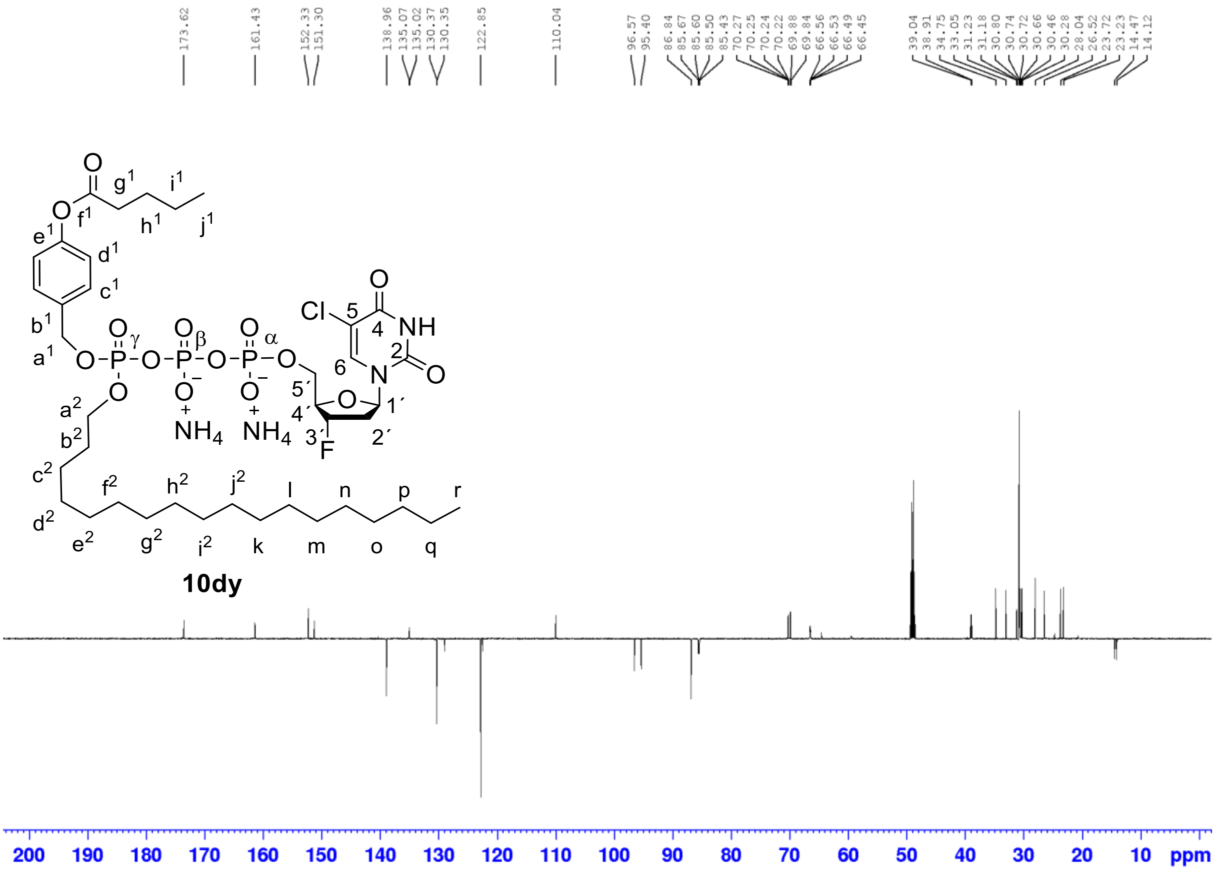
10dy
200 190 180 170 160 150 140 130 120 110 100 90 80 70 60 50 40 30 20 10 ppm

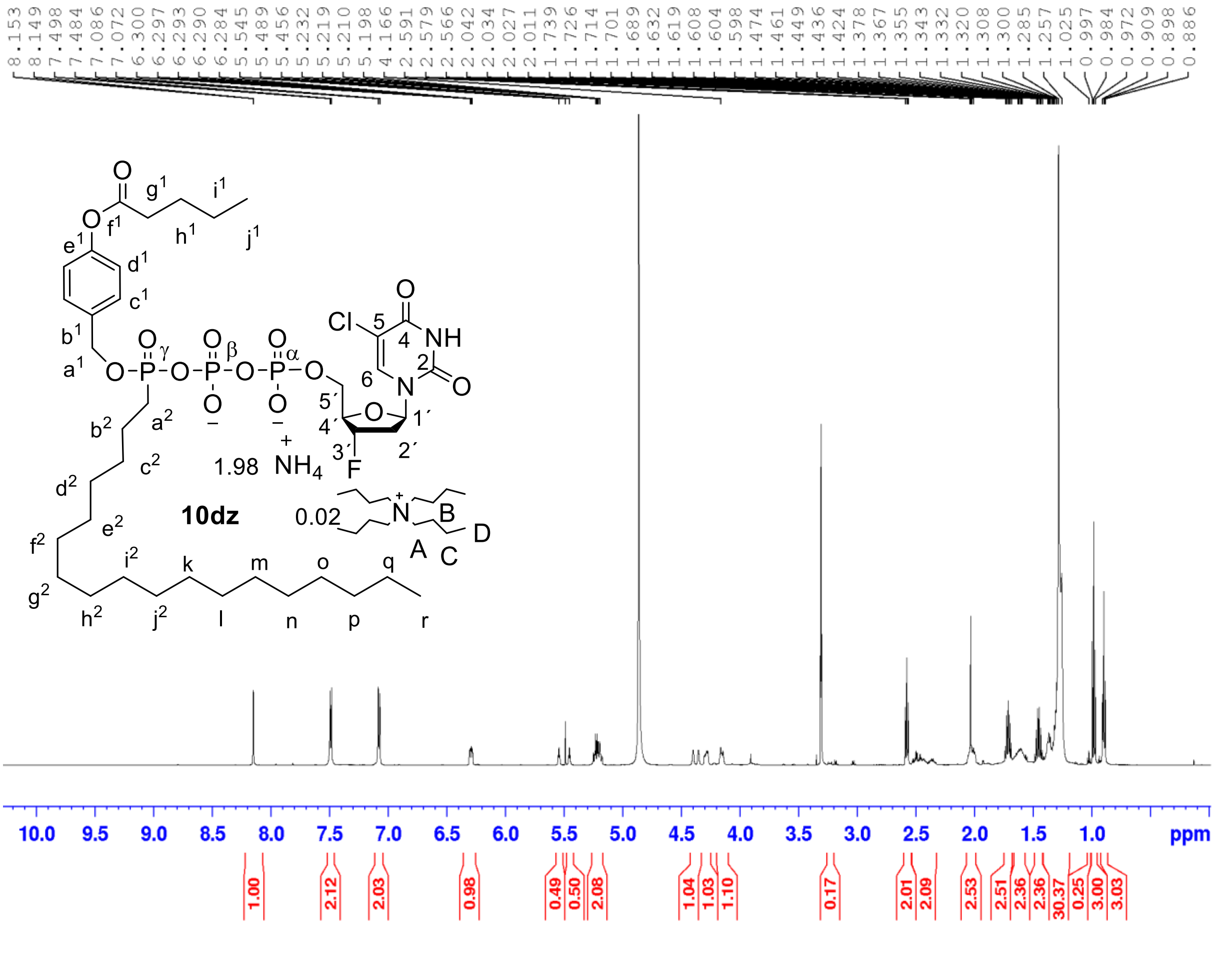
10dz
1.98 NH4
0.02
10.0 9.5 9.0 8.5 8.0 7.5 7.0 6.5 6.0 5.5 5.0 4.5 4.0 3.5 3.0 2.5 2.0 1.5 1.0 ppm

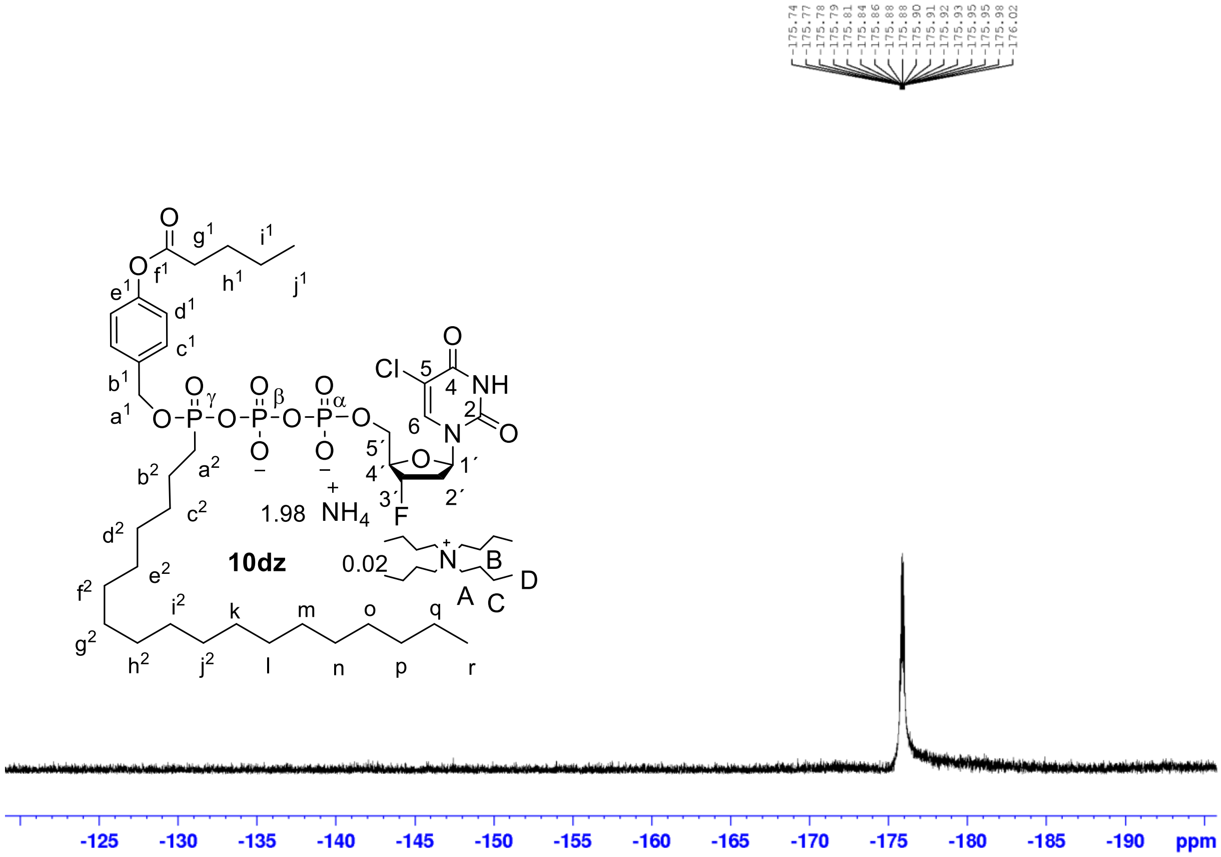
-175.74
-175.77
-175.78
-175.79
-175.81
-175.84
-175.86
-175.88
-175.88
-175.90
-175.91
-175.92
-175.93
-175.95
-175.95
-175.98
-176.02
O
g1
i1
O
f1
h1
j1
e1
d1
c1
b1
O
γ
O
β
O
α
Cl
5
4
NH
a1
O-P-O-P-O-P-O
6
2
N
O
O
O
5´
O
1´
b2
a2
+
4´
3´
2´
c2
1.98 NH4
F
d2
e2
10dz
0.02
N
B
f2
A
C
D
i2
k
m
o
q
g2
h2
j2
l
n
p
r
-125
-130
-135
-140
-145
-150
-155
-160
-165
-170
-175
-180
-185
-190
ppm

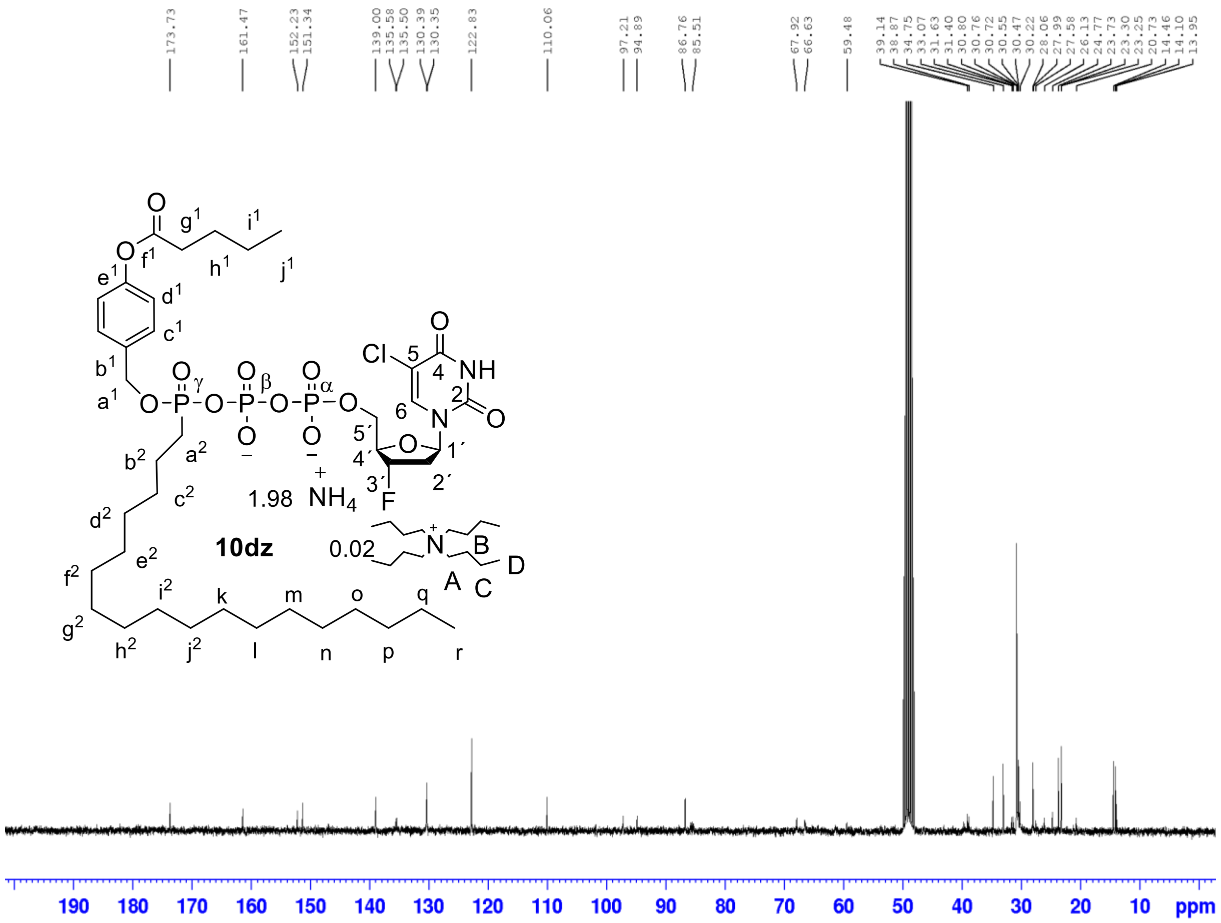
173.73
161.47
152.23
151.34
139.00
135.58
135.50
130.39
130.35
122.83
110.06
97.21
94.89
86.76
85.51
67.92
66.63
59.48
39.14
38.87
34.75
33.07
31.63
31.40
30.80
30.76
30.72
30.55
30.47
30.22
28.06
27.99
27.58
26.13
24.77
23.73
23.30
23.25
20.73
14.46
14.10
13.95
10dz
190
180
170
160
150
140
130
120
110
100
90
80
70
60
50
40
30
20
10
ppm

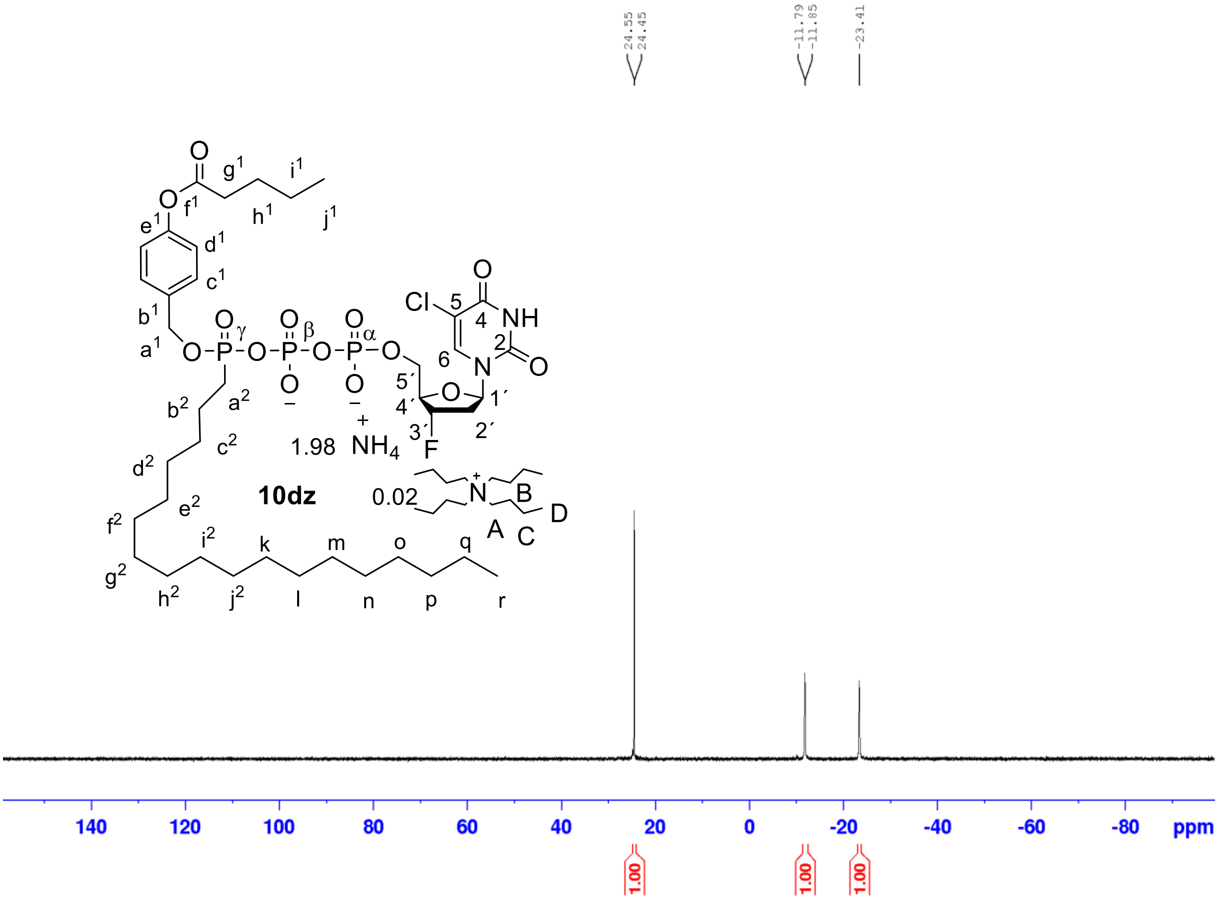
24.55
24.45
-11.79
-11.85
-23.41
10dz
1.98 NH4
0.02
140 120 100 80 60 40 20 0 -20 -40 -60 -80 ppm
1.00
1.00
1.00

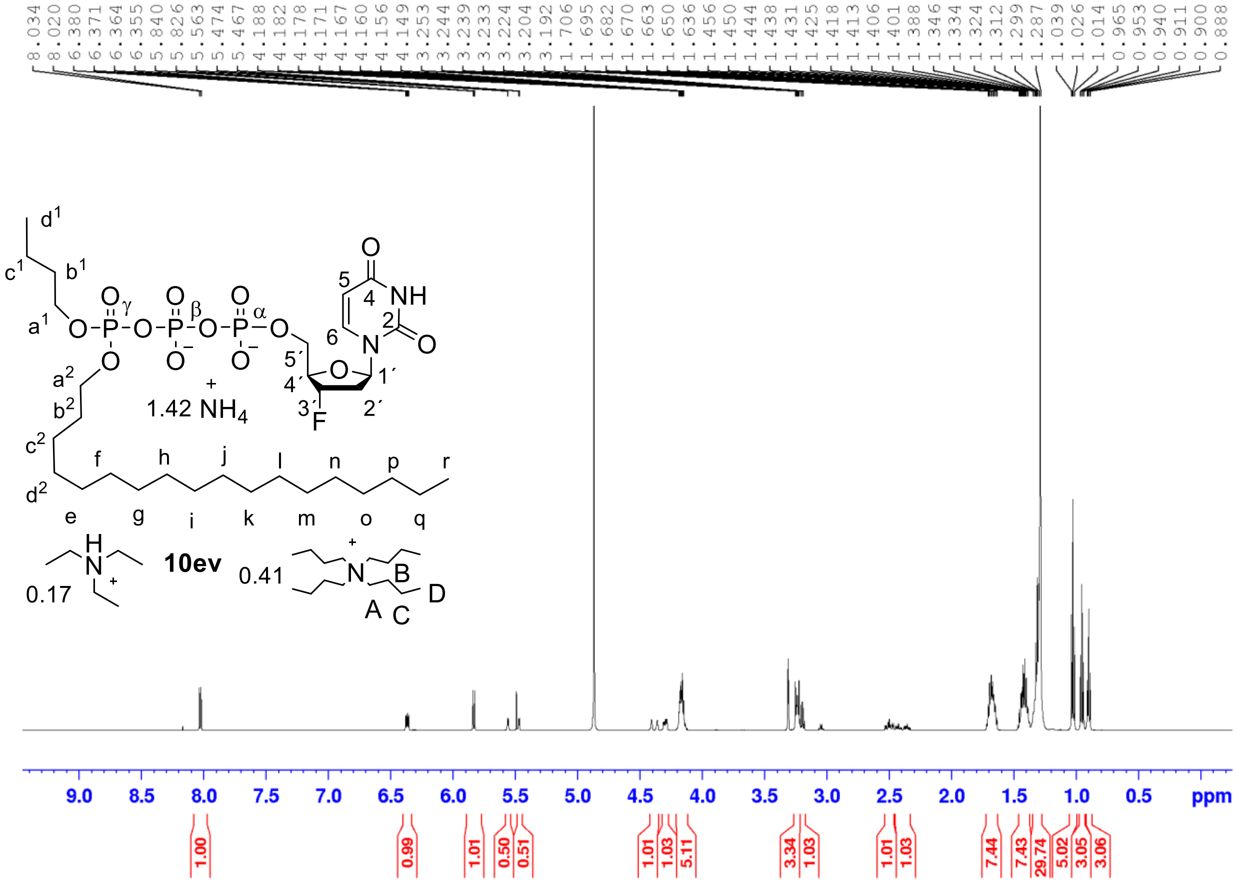
10ev
1.42 NH4
0.17
0.41
9.0 8.5 8.0 7.5 7.0 6.5 6.0 5.5 5.0 4.5 4.0 3.5 3.0 2.5 2.0 1.5 1.0 0.5 ppm
1.00 0.99 1.01 0.50 0.51 1.01 1.03 5.11 3.34 1.03 1.01 1.03 7.44 7.43 29.74 5.02 3.05 3.06

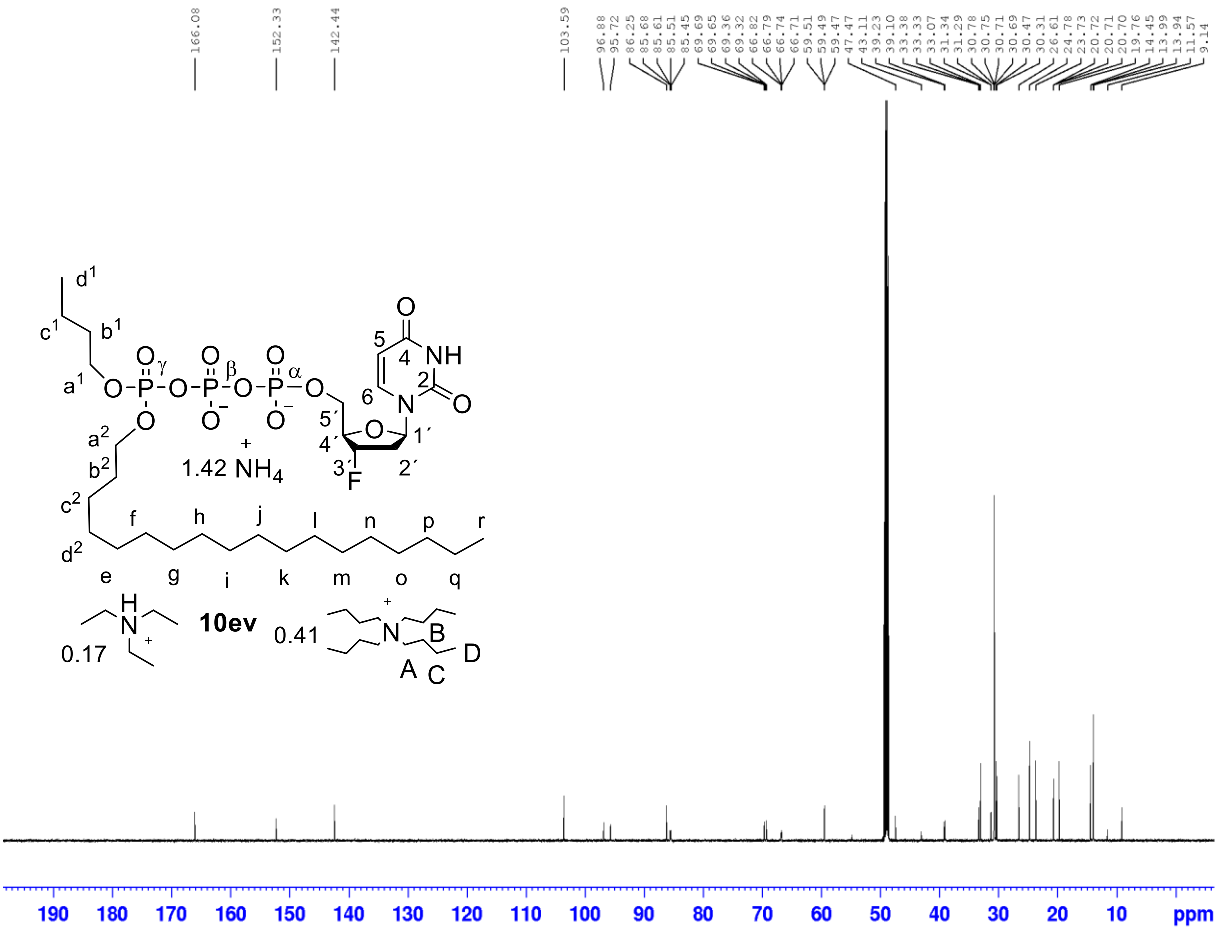
10ev
1.42 NH4
0.17
0.41
ppm

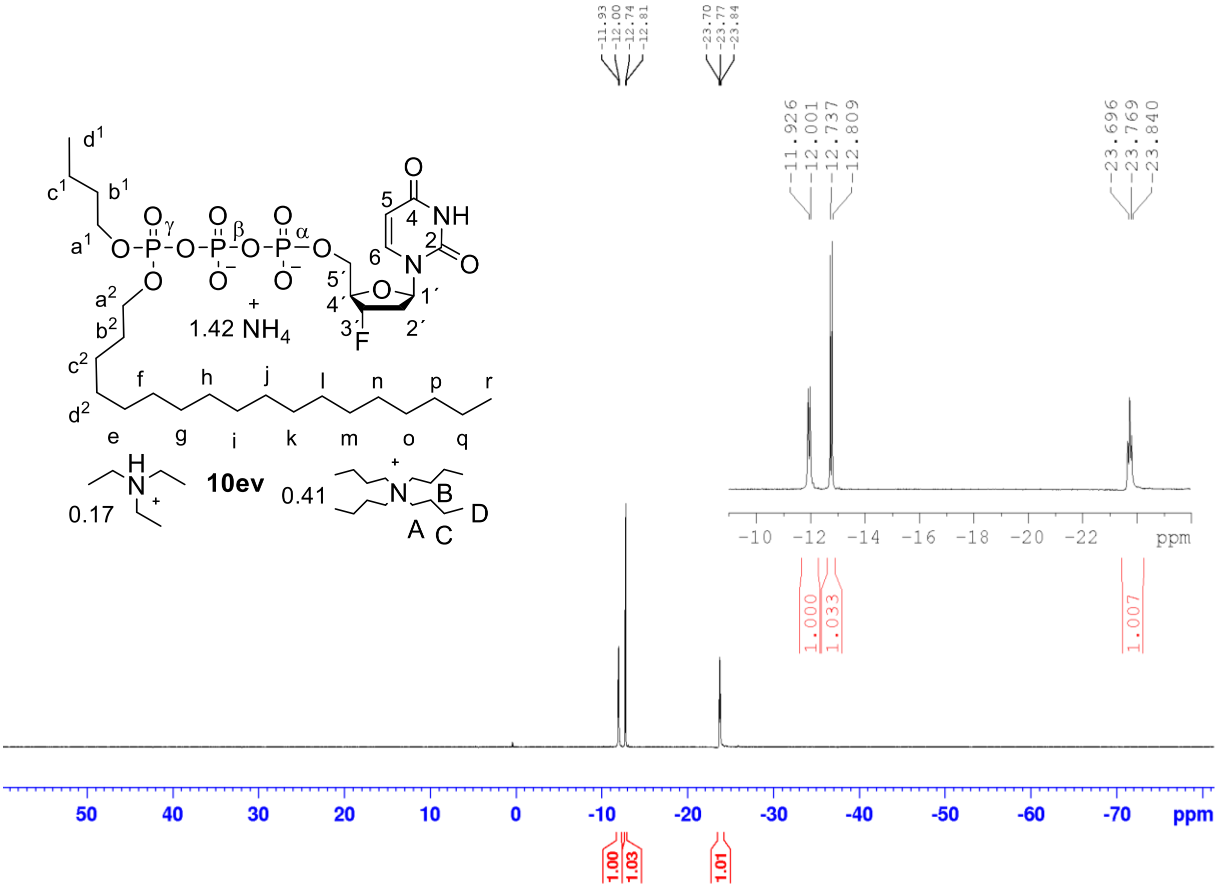
10ev
1.42 NH4
0.17
0.41
ppm

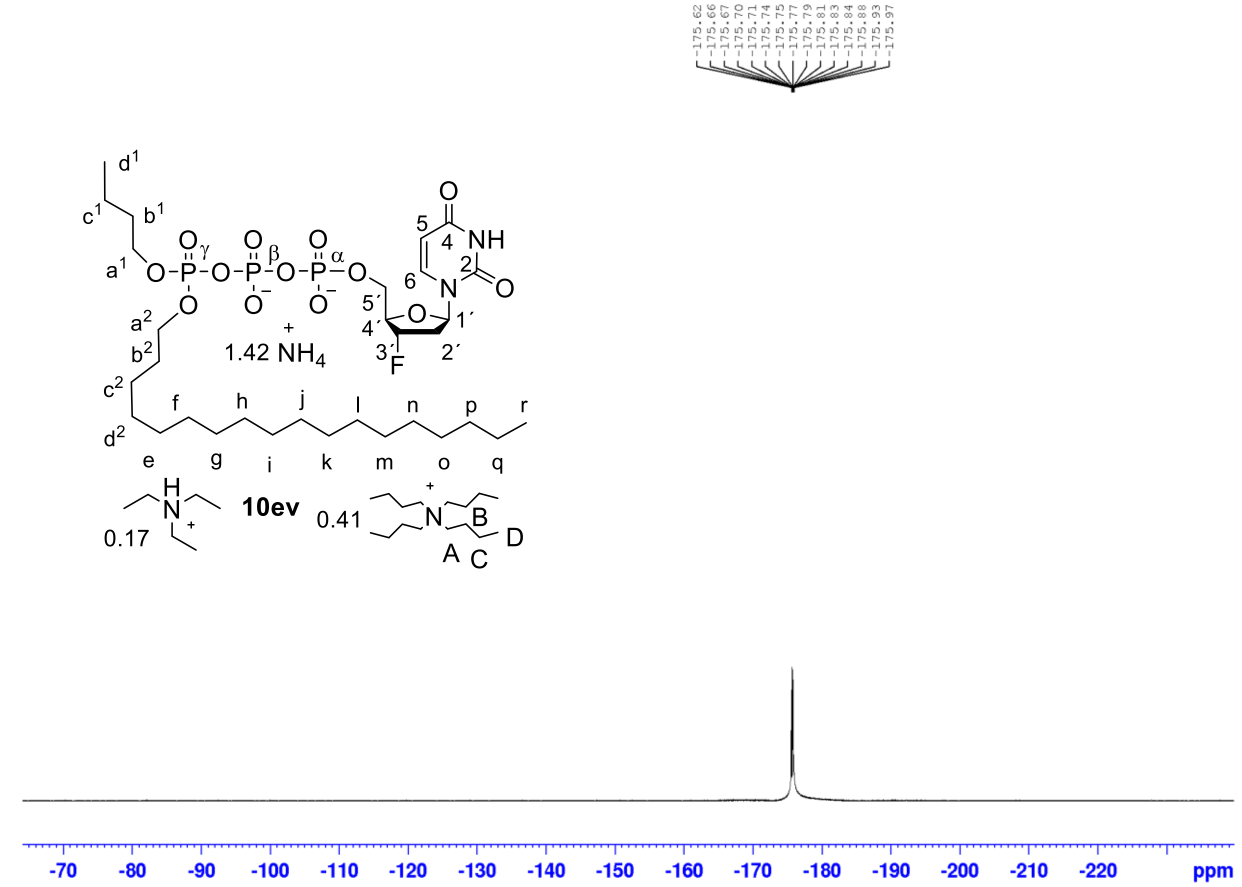
-175.62
-175.66
-175.67
-175.70
-175.71
-175.74
-175.75
-175.77
-175.79
-175.81
-175.83
-175.84
-175.88
-175.93
-175.97
1.42 NH4
0.17
10ev
0.41
-70 -80 -90 -100 -110 -120 -130 -140 -150 -160 -170 -180 -190 -200 -210 -220
ppm

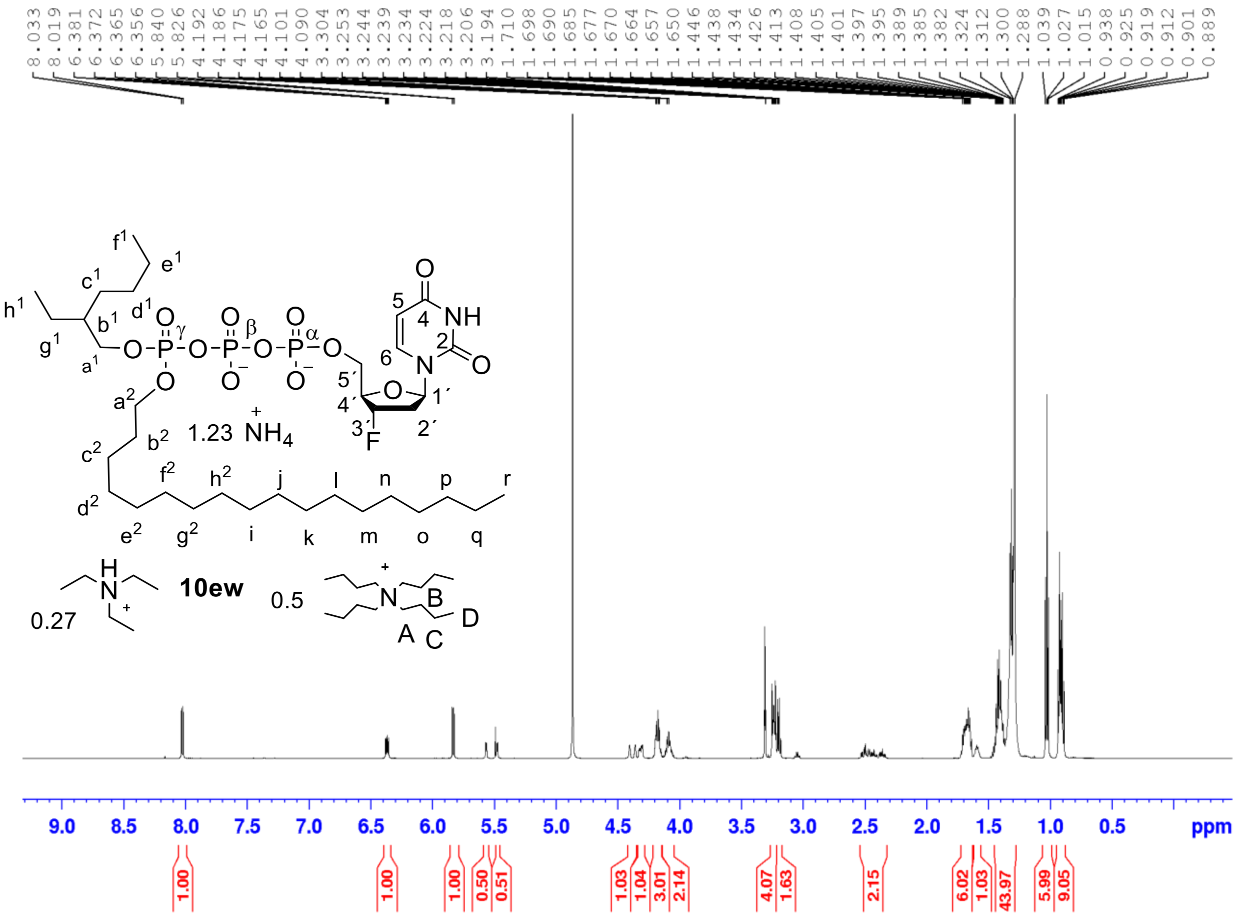
8.033 8.019 6.381 6.372 6.365 6.356 5.840 5.826 4.192 4.186 4.175 4.165 4.101 4.090 3.304 3.253 3.244 3.239 3.234 3.224 3.218 3.206 3.194 1.710 1.698 1.690 1.685 1.677 1.670 1.664 1.657 1.650 1.446 1.438 1.434 1.426 1.413 1.408 1.405 1.401 1.397 1.395 1.389 1.385 1.382 1.324 1.312 1.300 1.288 1.039 1.027 1.015 0.938 0.925 0.919 0.912 0.901 0.889
1.23 NH4
0.27
10ew
0.5
9.0 8.5 8.0 7.5 7.0 6.5 6.0 5.5 5.0 4.5 4.0 3.5 3.0 2.5 2.0 1.5 1.0 0.5
ppm
1.00 1.00 1.00 0.50 0.51 1.03 1.04 3.01 2.14 4.07 1.63 2.15 6.02 1.03 43.97 5.99 9.05

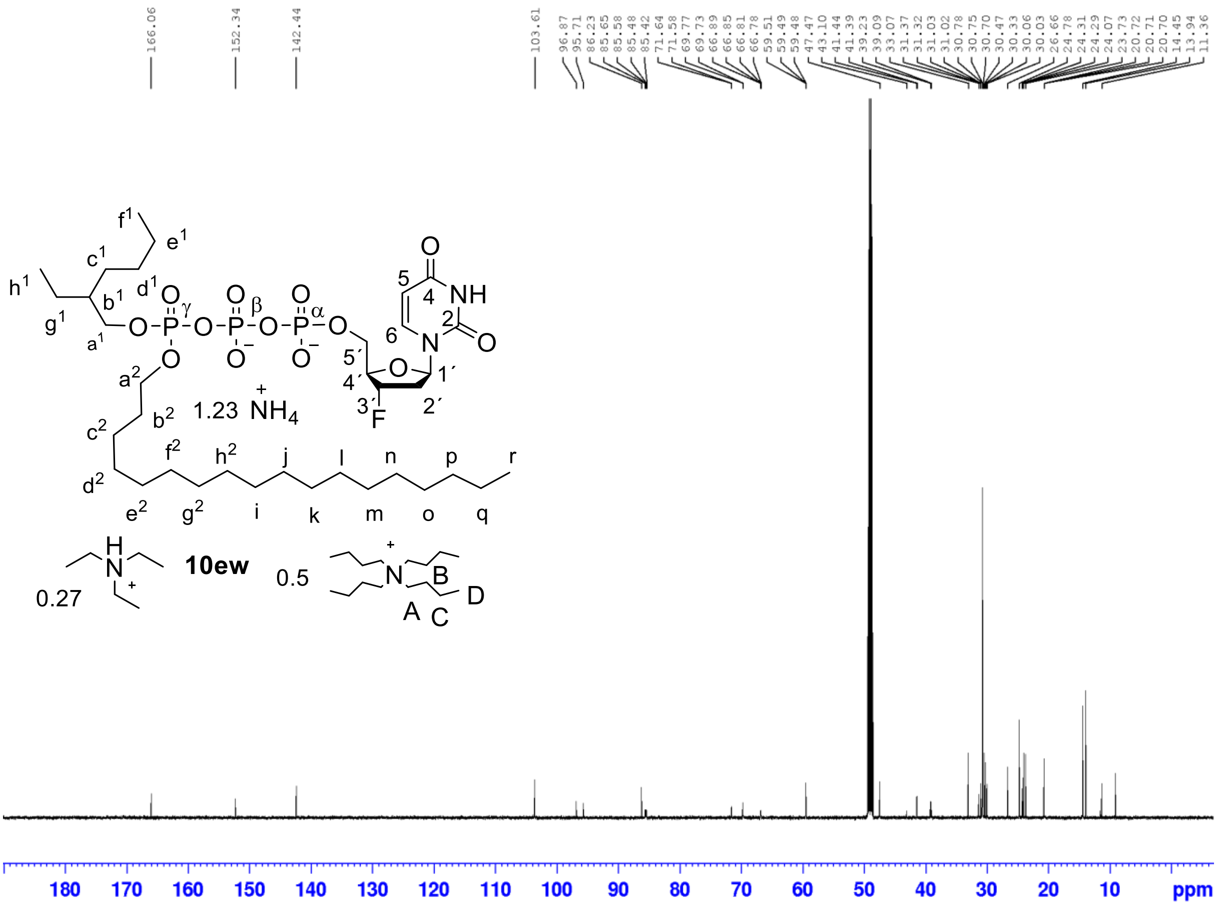
166.06
152.34
142.44
103.61
96.87 95.71 86.23 85.65 85.58 85.48 85.42 71.64 71.58 69.77 69.73 66.89 66.85 66.81 66.78 59.51 59.49 59.48 47.47 43.10 41.44 41.39 39.23 39.09 33.07 31.37 31.32 31.03 31.02 30.78 30.75 30.70 30.47 30.33 30.06 30.03 26.66 24.78 24.31 24.29 24.07 23.73 20.72 20.71 20.70 14.45 13.94 11.36
1.23 NH4
10ew
0.27
0.5
A B C D
180 170 160 150 140 130 120 110 100 90 80 70 60 50 40 30 20 10 ppm

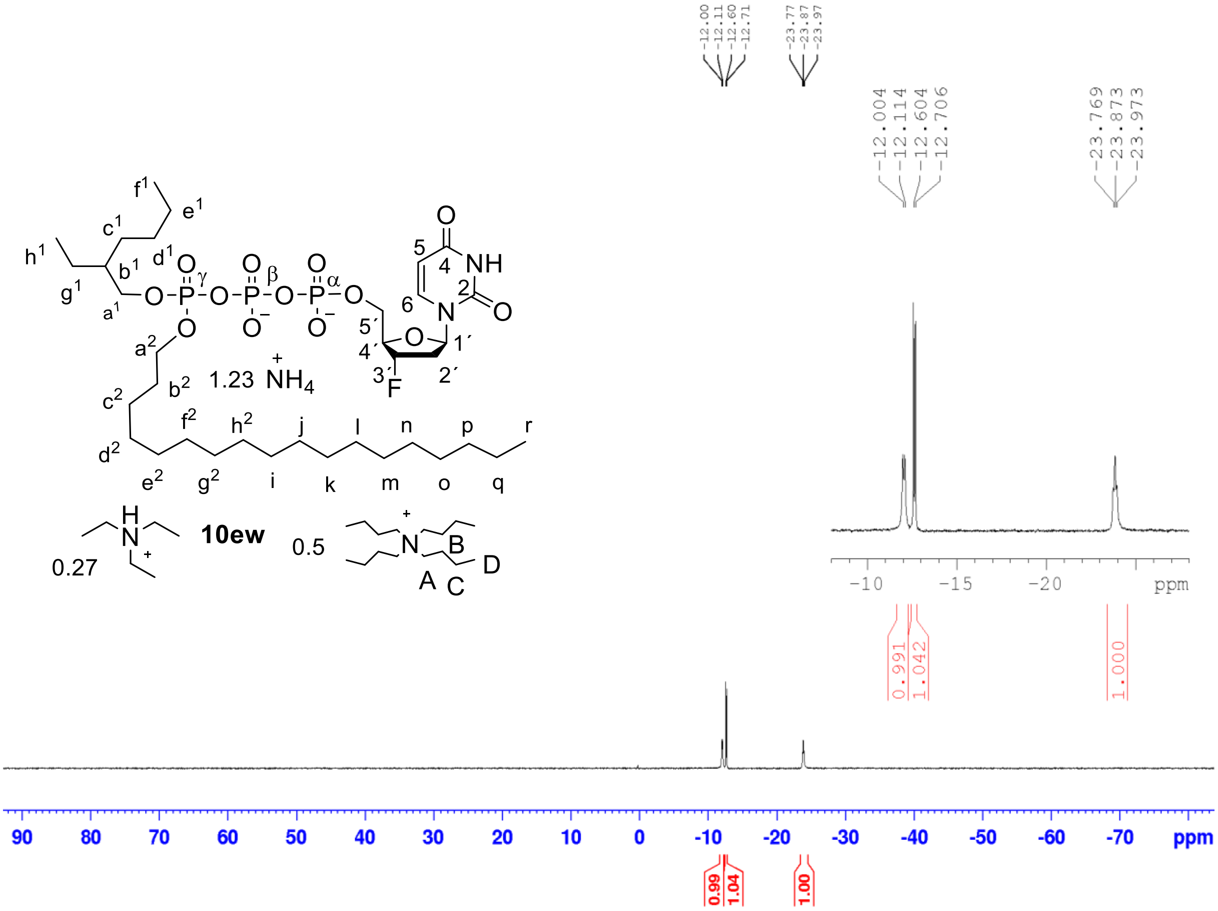
-12.00 -12.11 -12.60 -12.71
-23.77 -23.87 -23.97
-12.004 -12.114 -12.604 -12.706
-23.769 -23.873 -23.973
1.23 NH4
10ew
0.27
0.5
A B C D
-10 -15 -20 ppm
0.991 1.042 1.000
90 80 70 60 50 40 30 20 10 0 -10 -20 -30 -40 -50 -60 -70 ppm
0.99 1.04 1.00

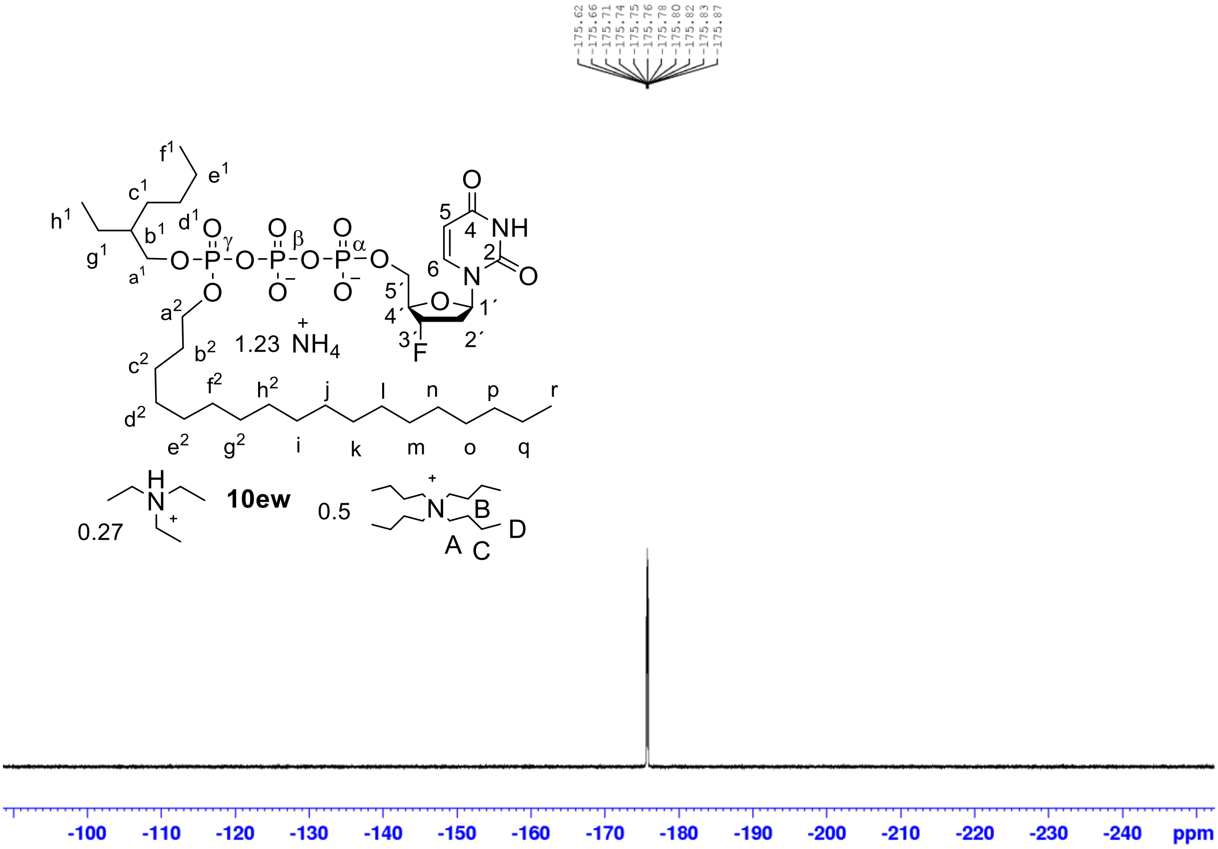
10ew
1.23 NH4
0.27
0.5
ppm

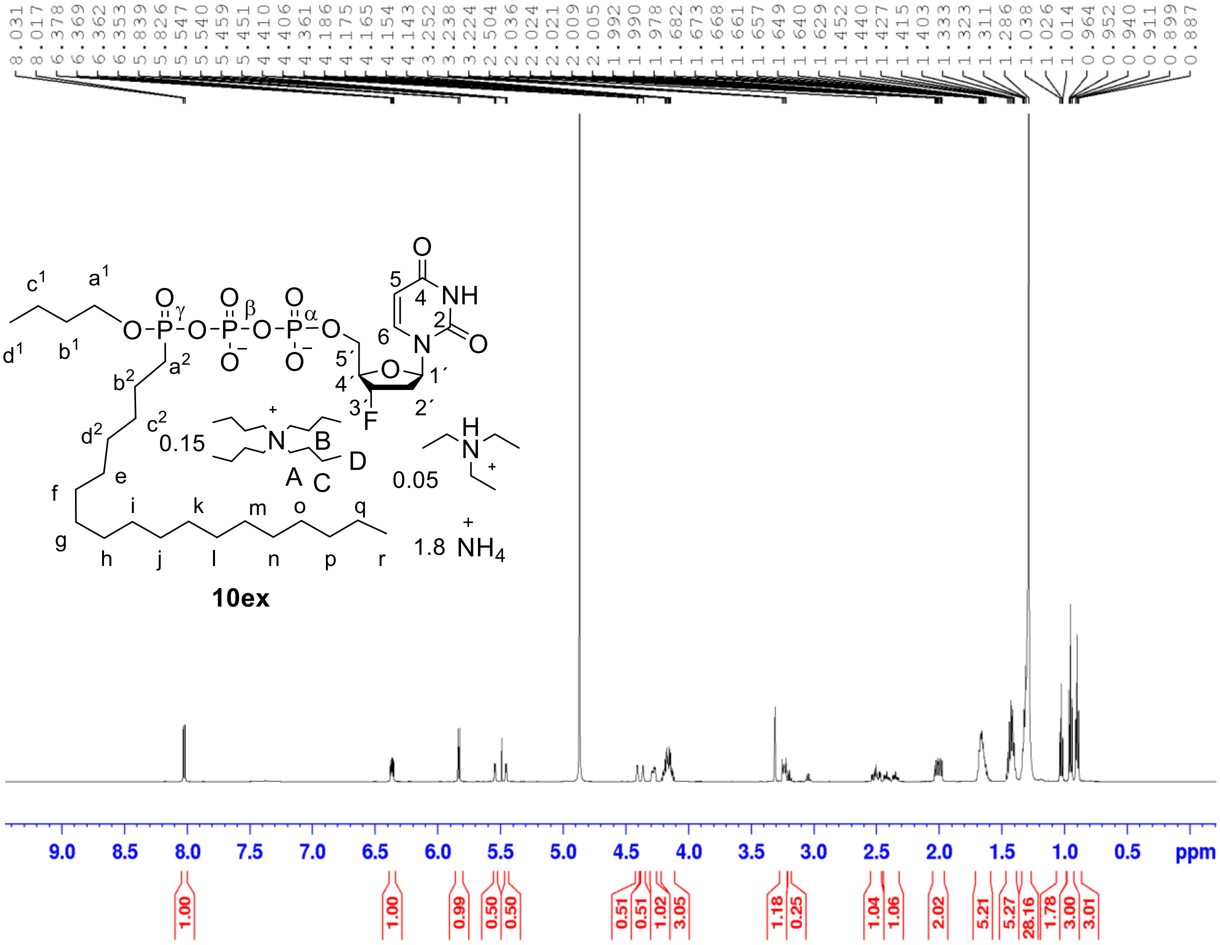
10ex
0.15
0.05
1.8 NH4
ppm

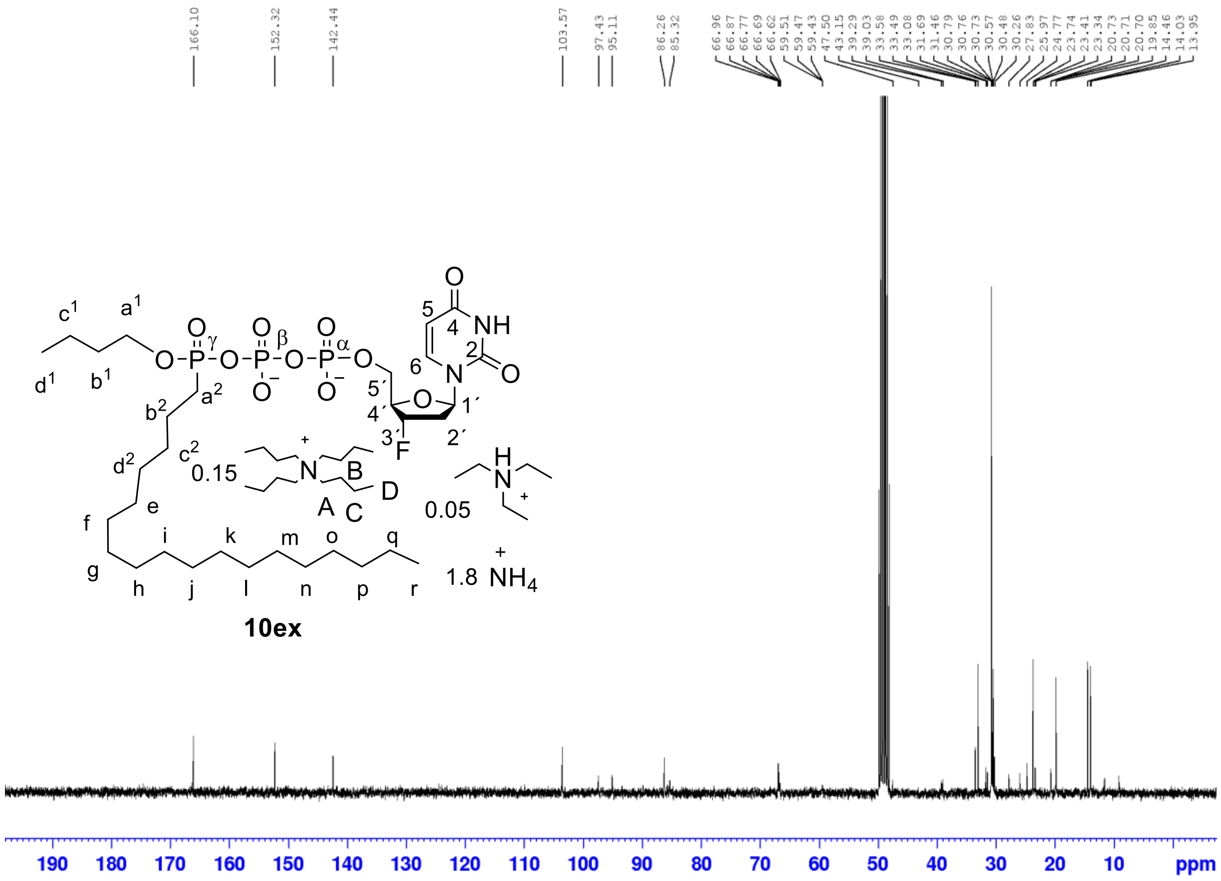
166.10
152.32
142.44
103.57
97.43
95.11
86.26
85.32
66.96
66.87
66.77
66.69
66.62
59.51
59.47
59.43
47.50
43.15
39.29
39.03
33.58
33.49
33.08
31.69
31.46
30.79
30.76
30.73
30.57
30.48
30.26
27.83
25.97
24.77
23.74
23.41
23.34
20.73
20.71
20.70
19.85
14.46
14.03
13.95
0.15
0.05
1.8 NH_4^+
10ex
190 180 170 160 150 140 130 120 110 100 90 80 70 60 50 40 30 20 10 ppm

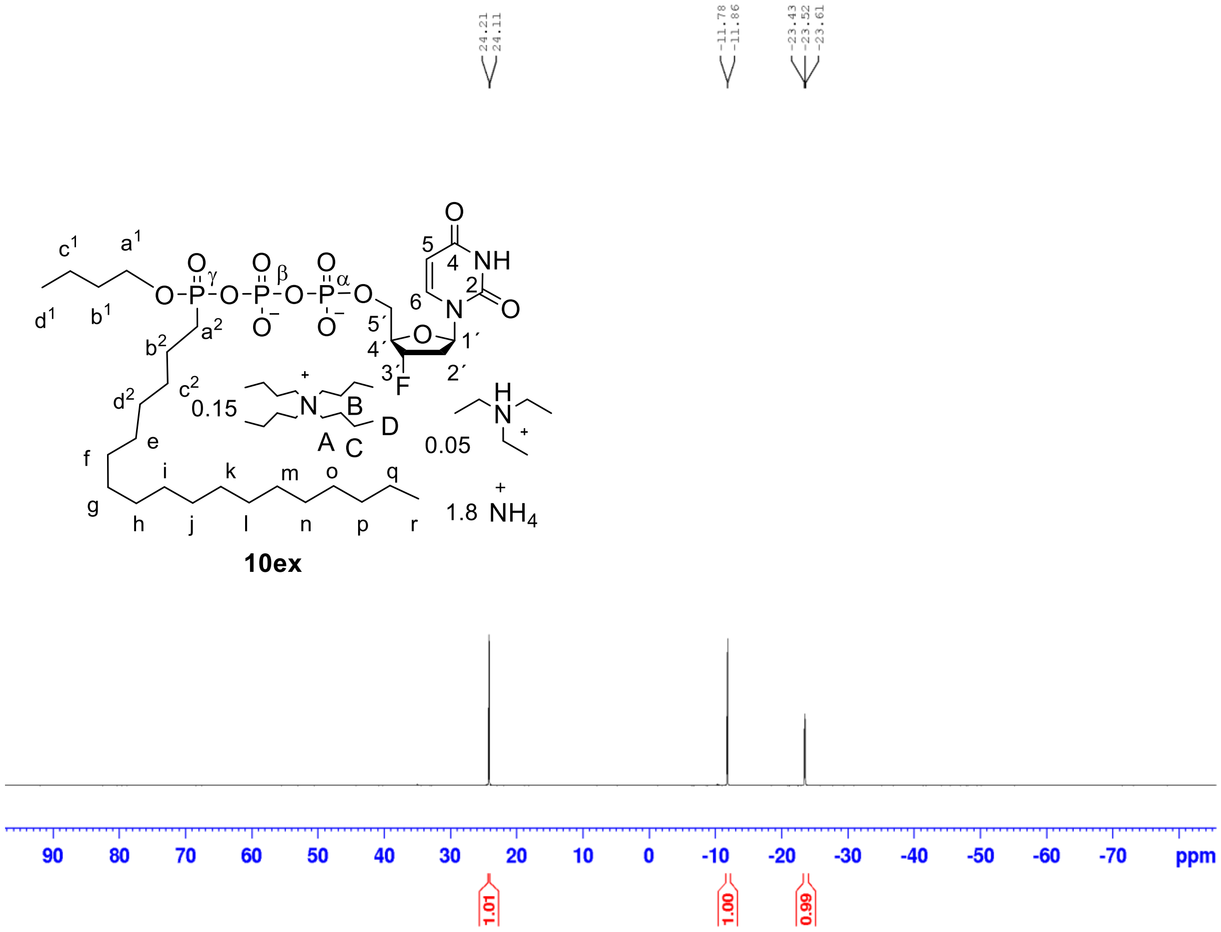
24.21
24.11
-11.78
-11.86
-23.43
-23.52
-23.61
0.15
0.05
1.8 NH_4^+
10ex
90 80 70 60 50 40 30 20 10 0 -10 -20 -30 -40 -50 -60 -70 ppm
1.01
1.00
0.99

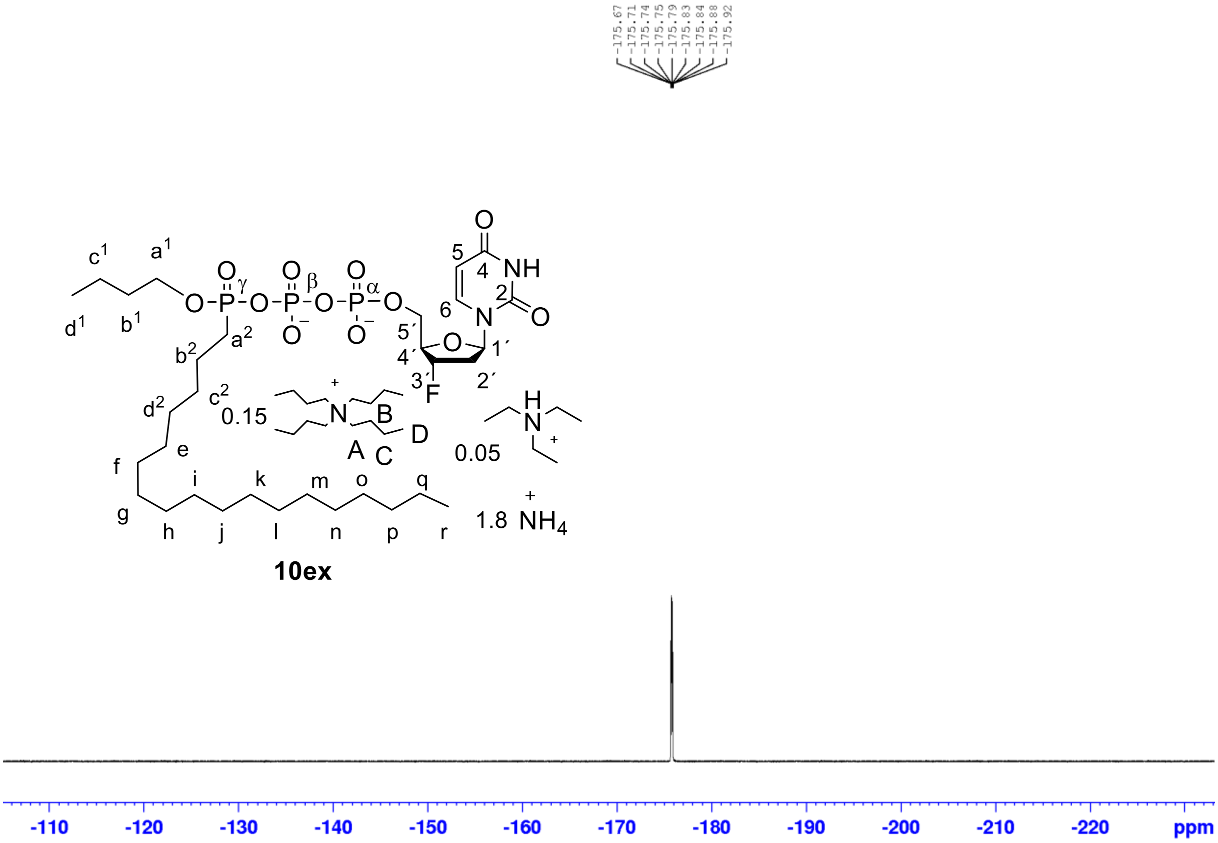
-175.67 -175.71 -175.74 -175.75 -175.79 -175.83 -175.84 -175.88 -175.92
10ex
0.15
0.05
1.8 NH_4^+
-110 -120 -130 -140 -150 -160 -170 -180 -190 -200 -210 -220 ppm

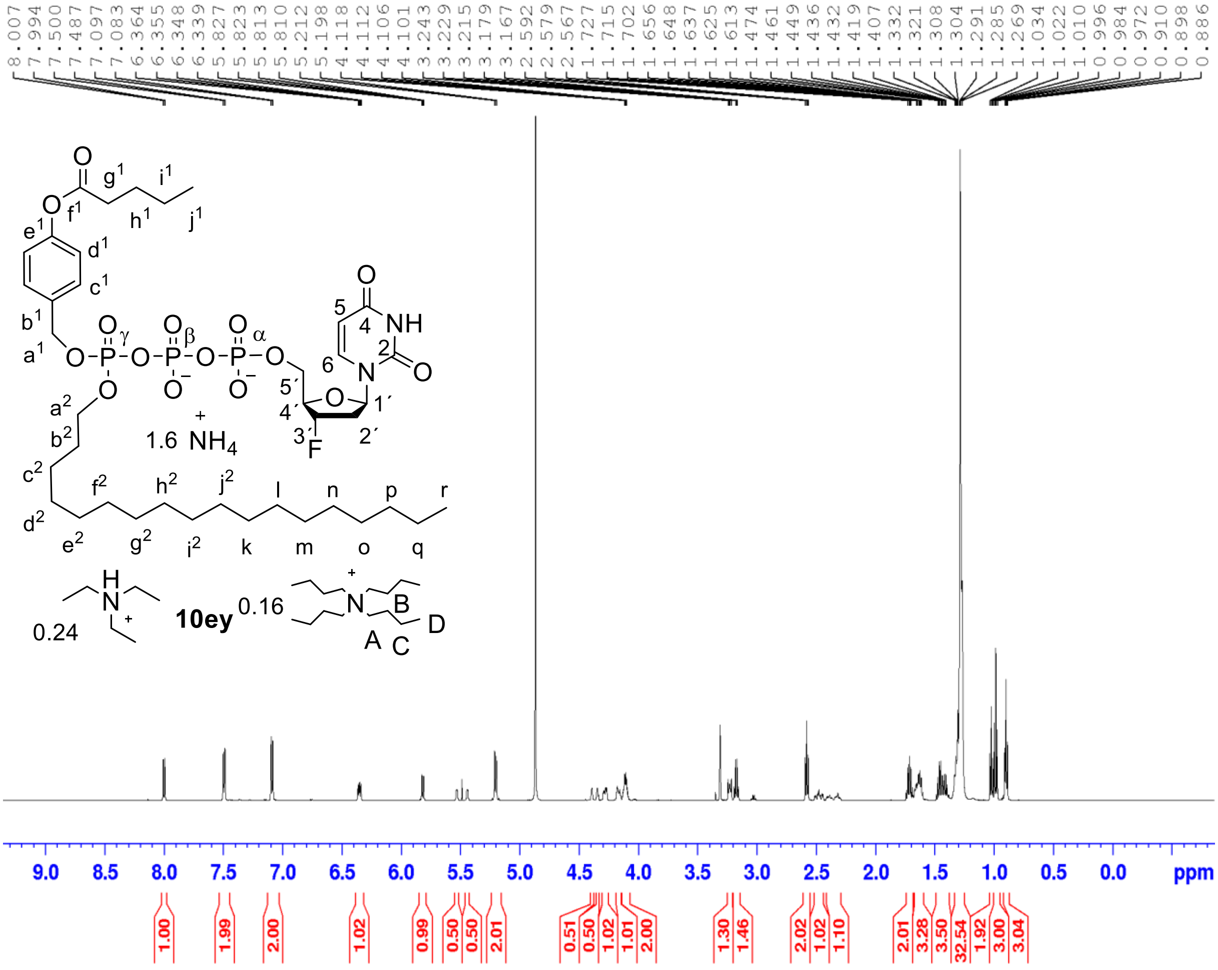
8.007 7.994 7.500 7.487 7.097 7.083 6.364 6.355 6.348 6.339 5.827 5.823 5.813 5.810 5.212 5.198 4.118 4.112 4.106 4.101 3.243 3.229 3.215 3.179 3.167 2.592 2.579 2.567 1.727 1.715 1.702 1.656 1.648 1.637 1.625 1.613 1.474 1.461 1.449 1.436 1.432 1.419 1.407 1.332 1.321 1.308 1.304 1.291 1.285 1.269 1.034 1.022 1.010 0.996 0.984 0.972 0.910 0.898 0.886
10ey
1.6 NH_4^+
0.24
0.16
9.0 8.5 8.0 7.5 7.0 6.5 6.0 5.5 5.0 4.5 4.0 3.5 3.0 2.5 2.0 1.5 1.0 0.5 0.0 ppm
1.00 1.99 2.00 1.02 0.99 0.50 0.50 2.01 0.51 0.50 1.02 1.01 2.00 1.30 1.46 2.02 1.02 1.10 2.01 3.28 3.50 32.54 1.92 3.00 3.04

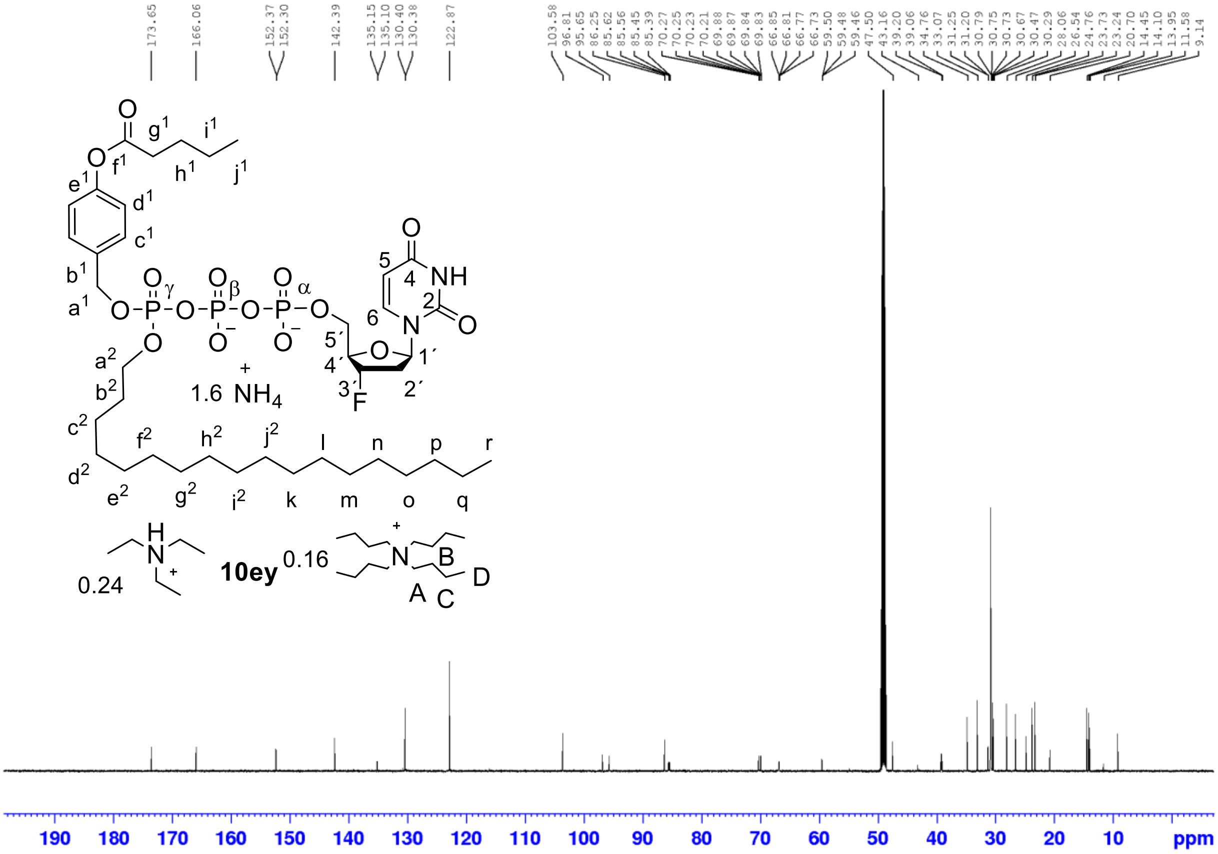

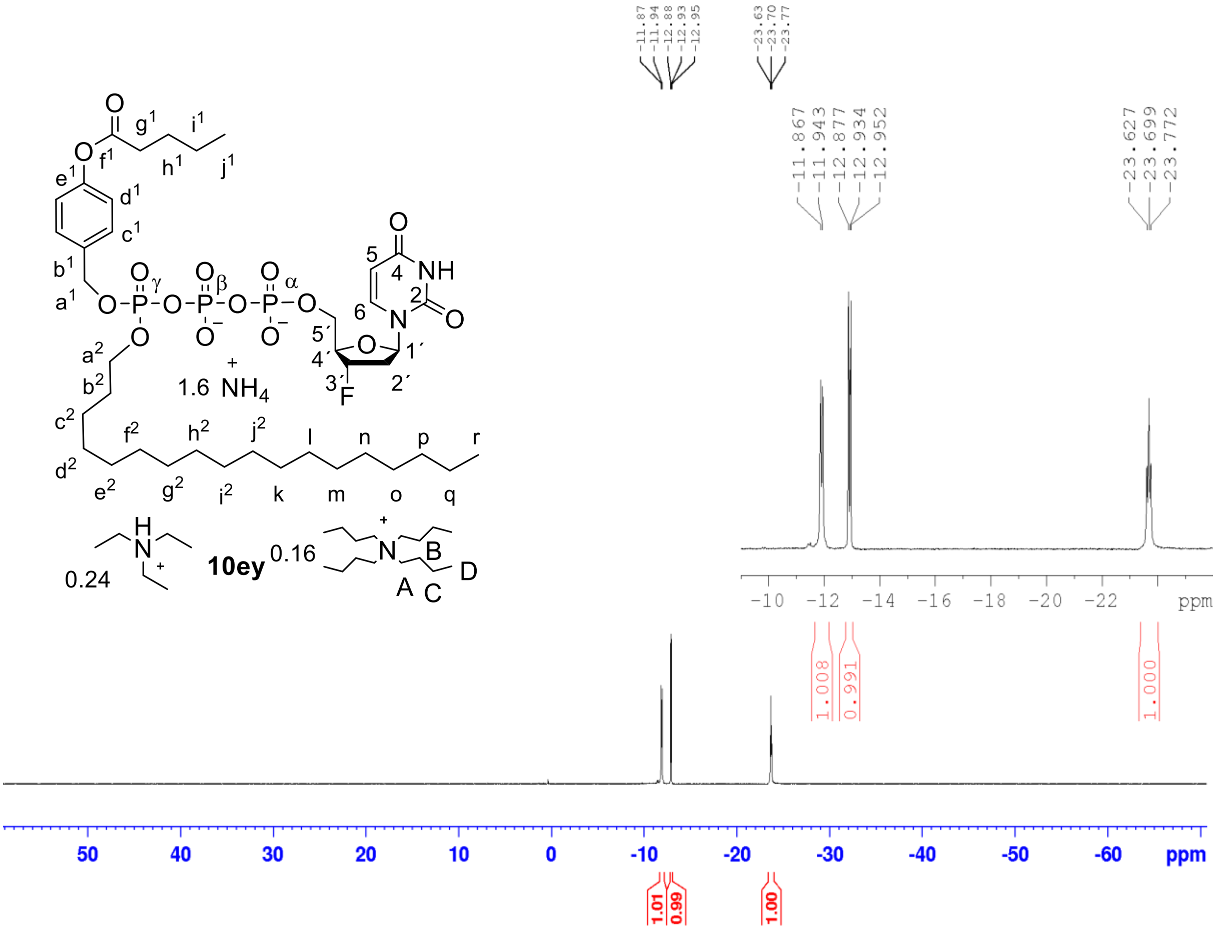

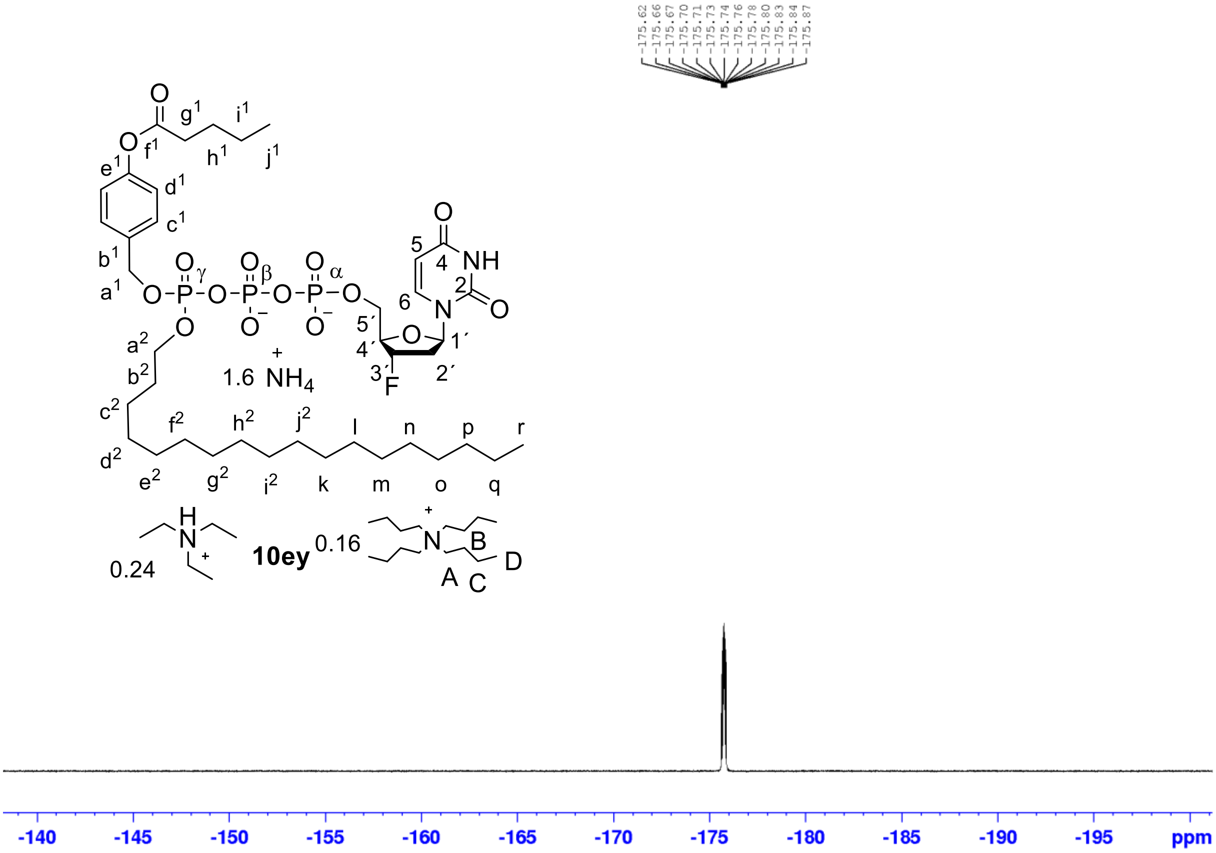
-175.62
-175.66
-175.67
-175.70
-175.71
-175.73
-175.74
-175.76
-175.78
-175.80
-175.83
-175.84
-175.87
1.6 NH4
0.24
10ey
0.16
-140 -145 -150 -155 -160 -165 -170 -175 -180 -185 -190 -195 ppm

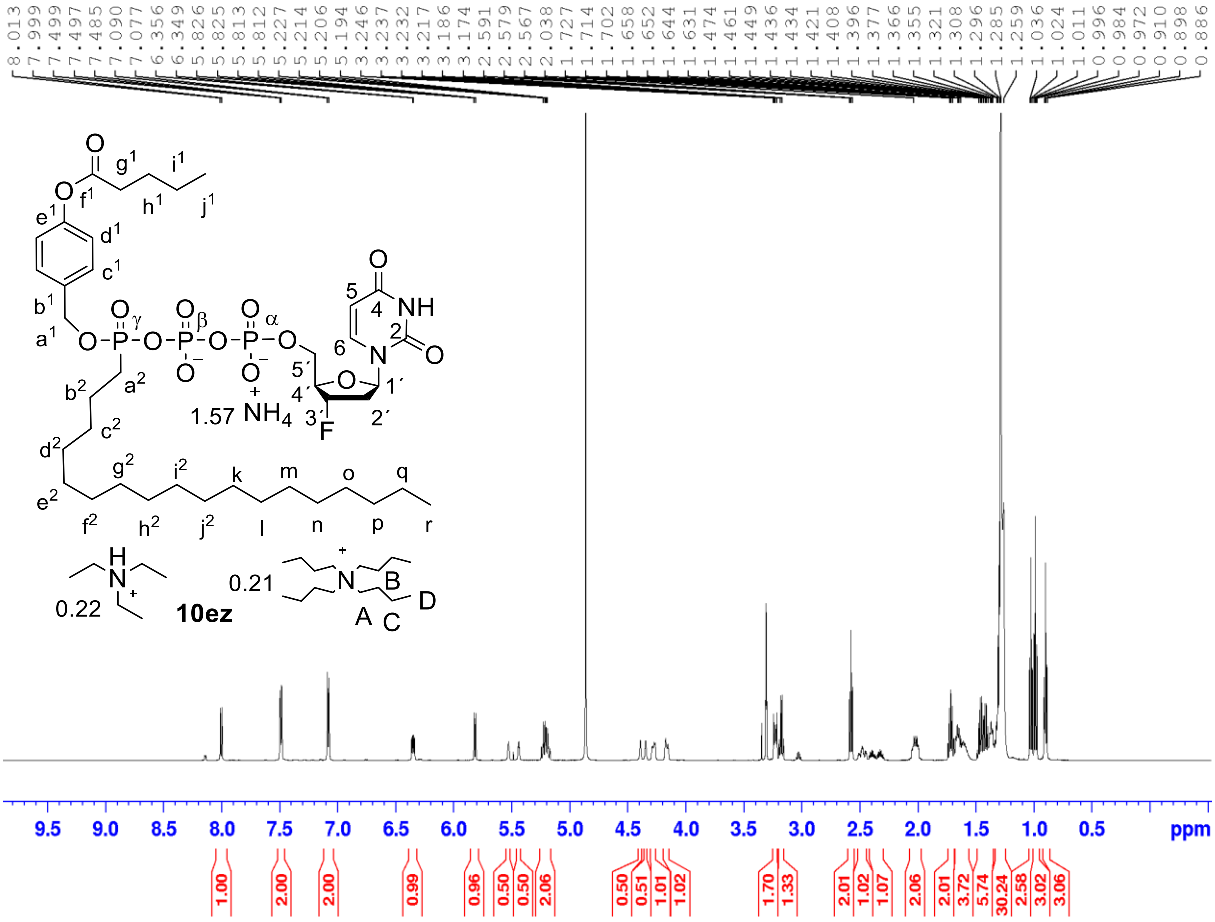
8.013 7.999 7.499 7.497 7.485 7.090 7.077 6.356 6.349 5.826 5.825 5.813 5.812 5.227 5.214 5.206 5.194 3.246 3.237 3.232 3.217 3.186 3.174 2.591 2.579 2.567 2.038 1.727 1.714 1.702 1.658 1.652 1.644 1.631 1.474 1.461 1.449 1.436 1.434 1.421 1.408 1.396 1.377 1.366 1.355 1.321 1.308 1.296 1.285 1.259 1.036 1.024 1.011 0.996 0.984 0.972 0.910 0.898 0.886
1.57 NH4
0.22
10ez
0.21
9.5 9.0 8.5 8.0 7.5 7.0 6.5 6.0 5.5 5.0 4.5 4.0 3.5 3.0 2.5 2.0 1.5 1.0 0.5 ppm
1.00 2.00 2.00 0.99 0.96 0.50 0.50 0.50 2.06 0.50 0.51 1.01 1.02 1.70 1.33 2.01 1.02 1.07 2.06 2.01 3.72 5.74 30.24 2.58 3.02 3.06

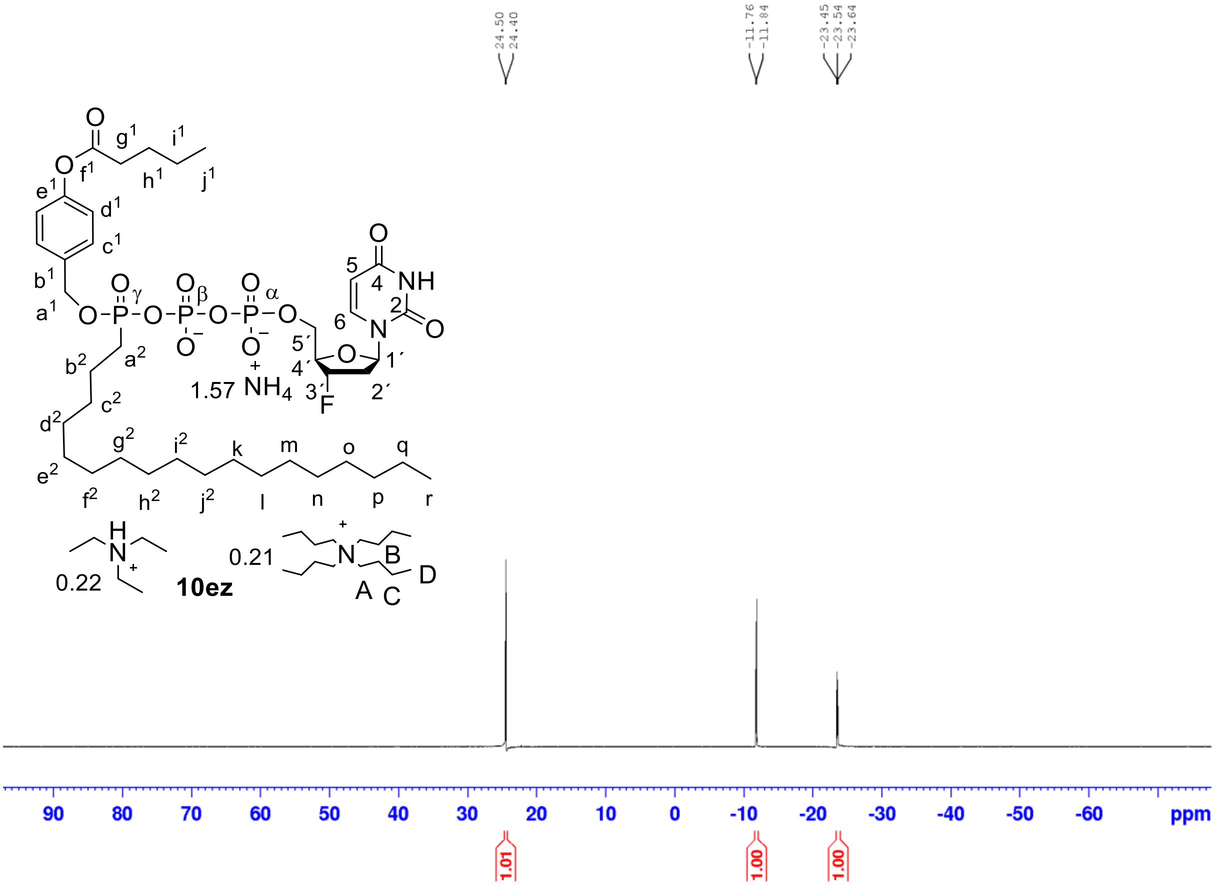
24.50
24.40
-11.76
-11.84
-23.45
-23.54
-23.64
10ez
1.57 NH4
0.22
0.21
ppm
1.01
1.00
1.00

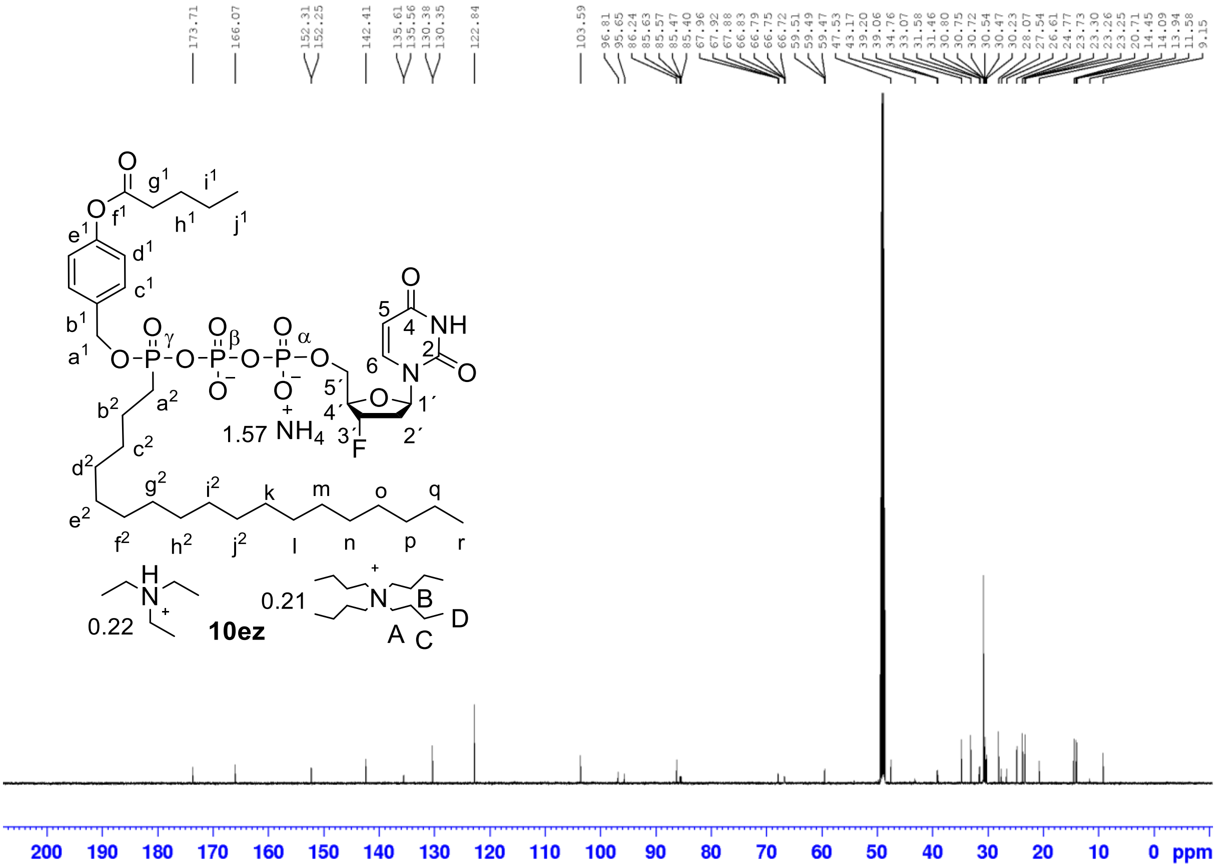
173.71
166.07
152.31
152.25
142.41
135.61
135.56
130.38
130.35
122.84
103.59
96.81
95.65
86.24
85.63
85.57
85.47
85.40
67.96
67.92
67.88
66.83
66.79
66.75
66.72
59.51
59.49
59.47
47.53
43.17
39.20
39.06
34.76
33.07
31.58
31.46
30.80
30.75
30.72
30.54
30.47
30.23
28.07
27.54
26.61
24.77
23.73
23.30
23.26
23.25
20.71
14.45
14.09
13.94
11.58
9.15
10ez
1.57 NH4
0.22
0.21
ppm

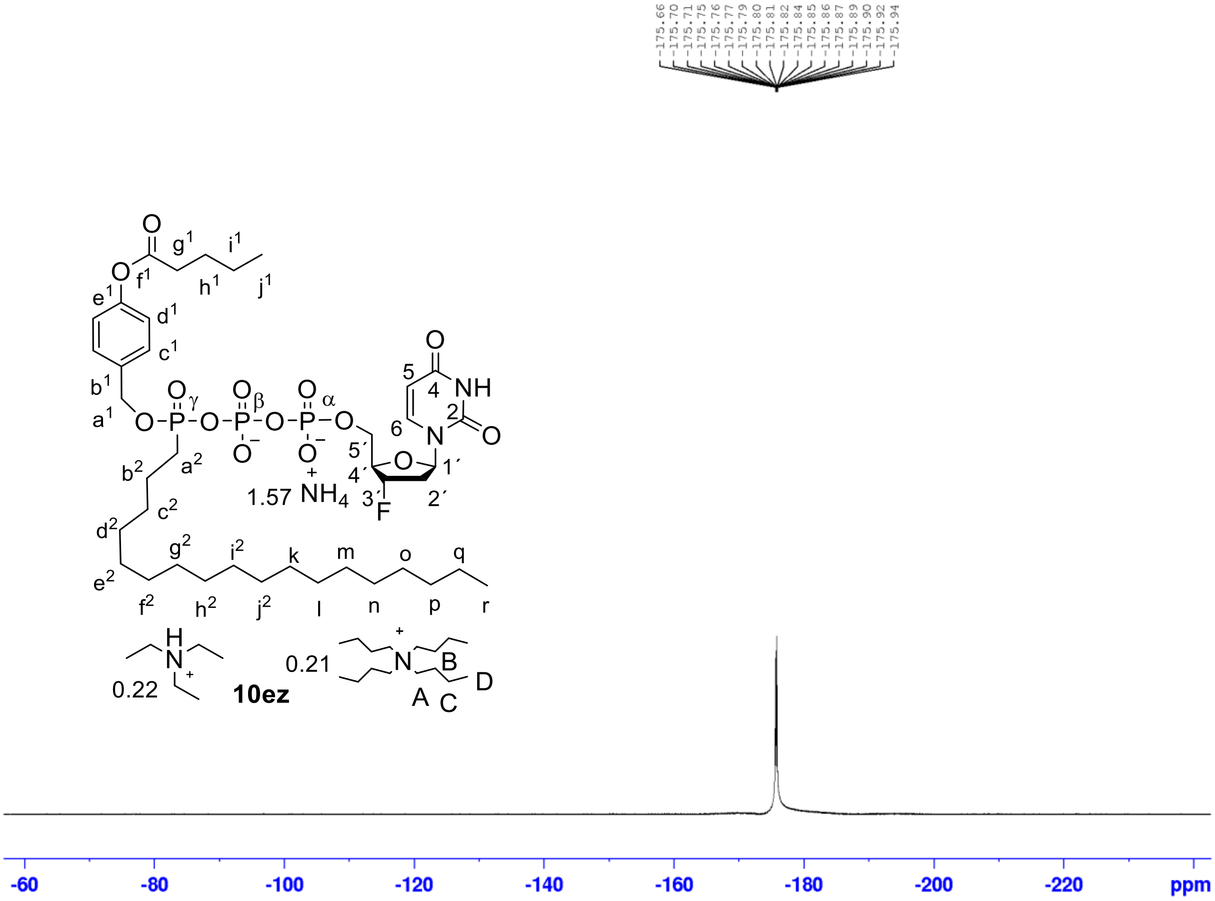
-175.66 -175.70 -175.71 -175.75 -175.76 -175.77 -175.79 -175.80 -175.81 -175.82 -175.84 -175.85 -175.86 -175.87 -175.89 -175.90 -175.92 -175.94
1.57 NH4
0.22
0.21
10ez
-60 -80 -100 -120 -140 -160 -180 -200 -220 ppm

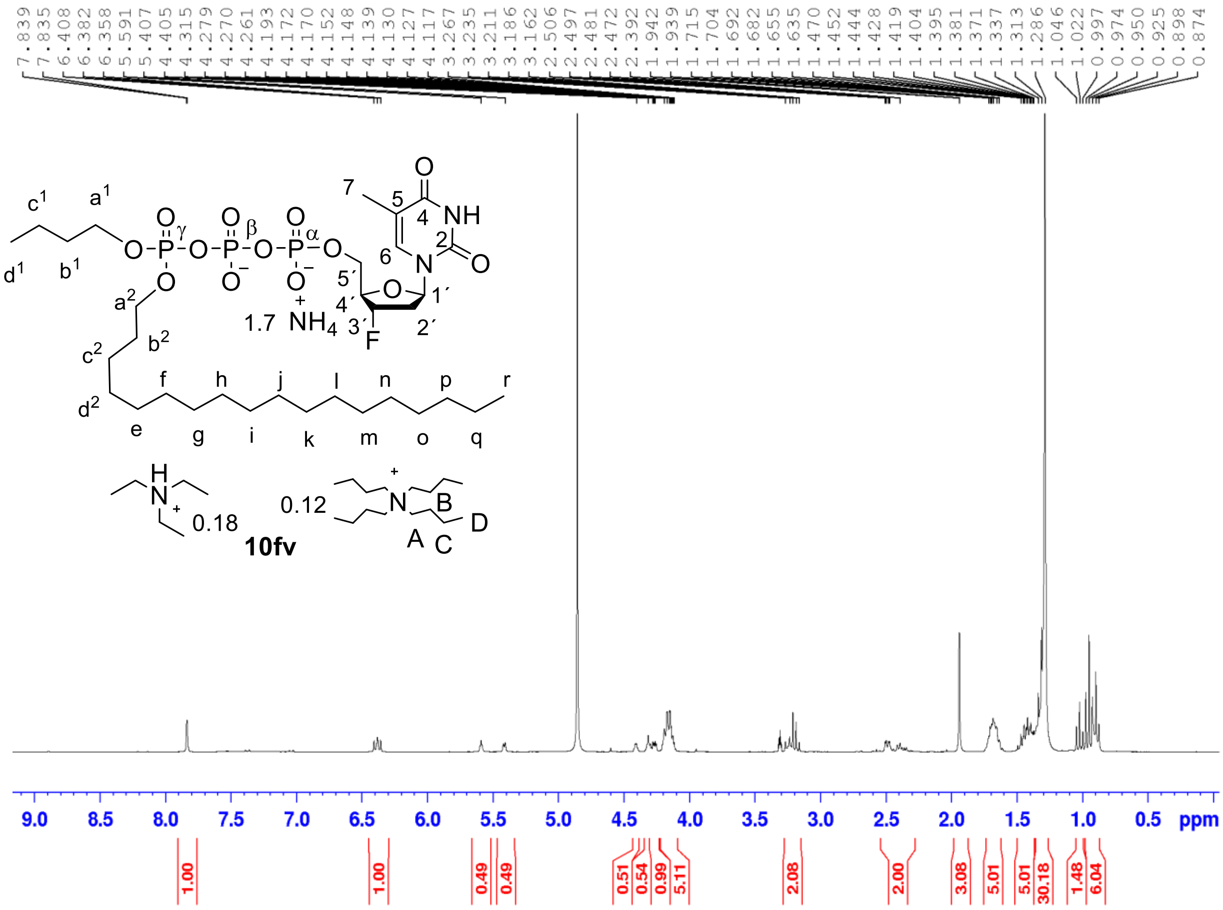
7.839 7.835 6.408 6.382 6.358 5.591 5.407 5.405 4.315 4.279 4.270 4.261 4.193 4.172 4.170 4.152 4.148 4.139 4.130 4.127 4.117 3.267 3.235 3.211 3.186 3.162 2.506 2.497 2.481 2.472 2.392 1.942 1.939 1.715 1.704 1.692 1.682 1.655 1.635 1.470 1.452 1.444 1.428 1.419 1.404 1.395 1.381 1.371 1.337 1.313 1.286 1.046 1.022 0.997 0.974 0.950 0.925 0.898 0.874
1.7 NH4
0.18
0.12
10fv
9.0 8.5 8.0 7.5 7.0 6.5 6.0 5.5 5.0 4.5 4.0 3.5 3.0 2.5 2.0 1.5 1.0 0.5 ppm
1.00 1.00 0.49 0.49 0.51 0.54 0.99 5.11 2.08 2.00 3.08 5.01 5.01 30.18 1.48 6.04

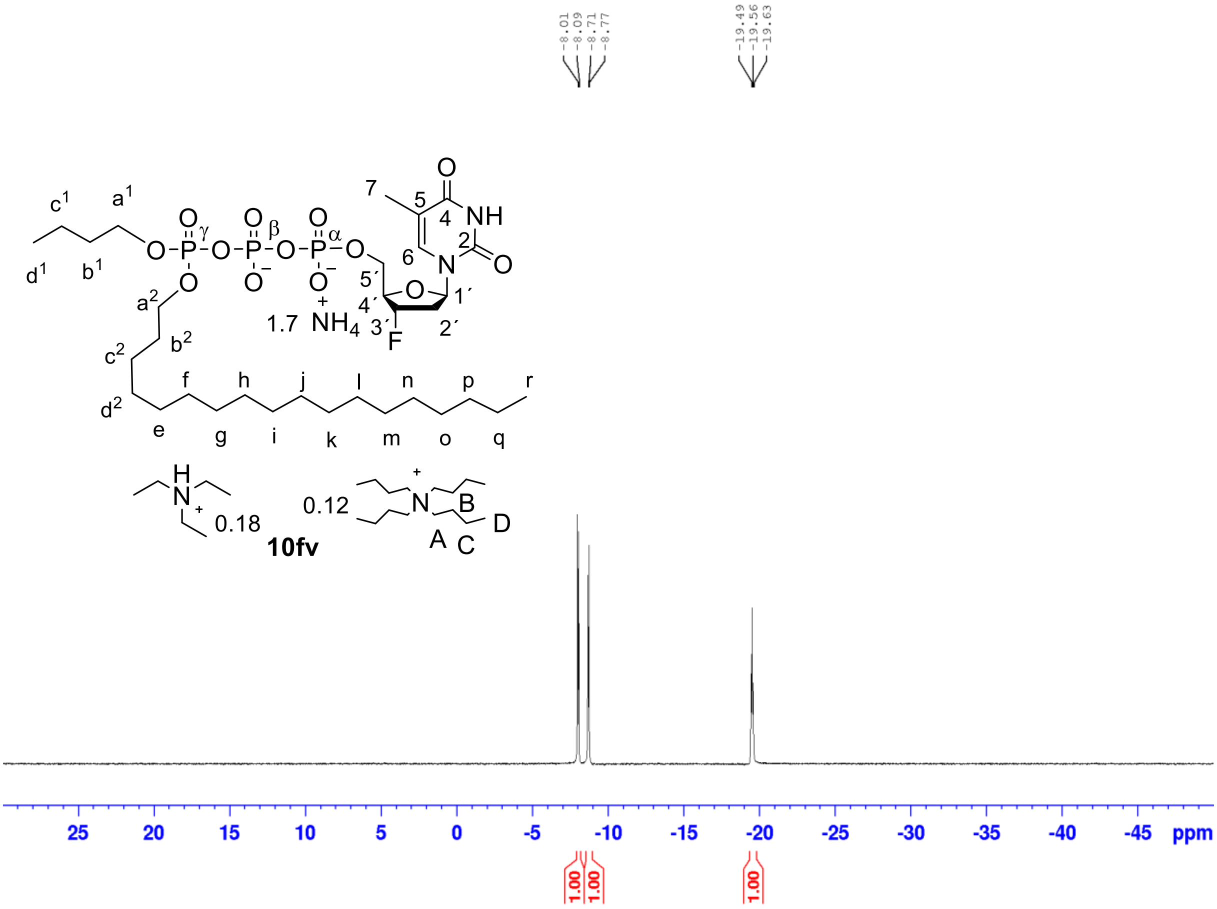
-8.01
-8.09
-8.71
-8.77
-19.49
-19.56
-19.63
10fv
1.7 NH4
0.18
0.12
25 20 15 10 5 0 -5 -10 -15 -20 -25 -30 -35 -40 -45 ppm
1.00
1.00
1.00

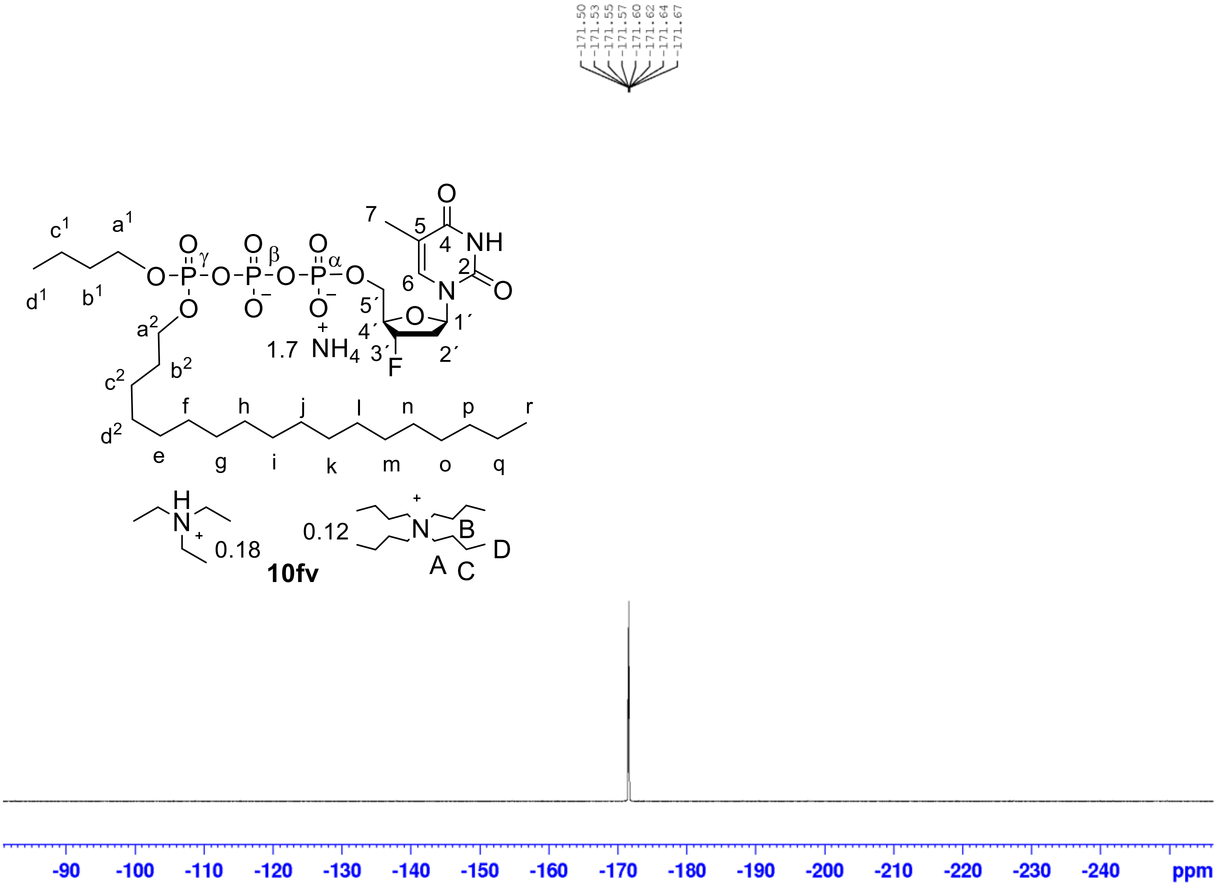
-171.50
-171.53
-171.55
-171.57
-171.60
-171.62
-171.64
-171.67
10fv
1.7 NH4
0.18
0.12
-90 -100 -110 -120 -130 -140 -150 -160 -170 -180 -190 -200 -210 -220 -230 -240 ppm

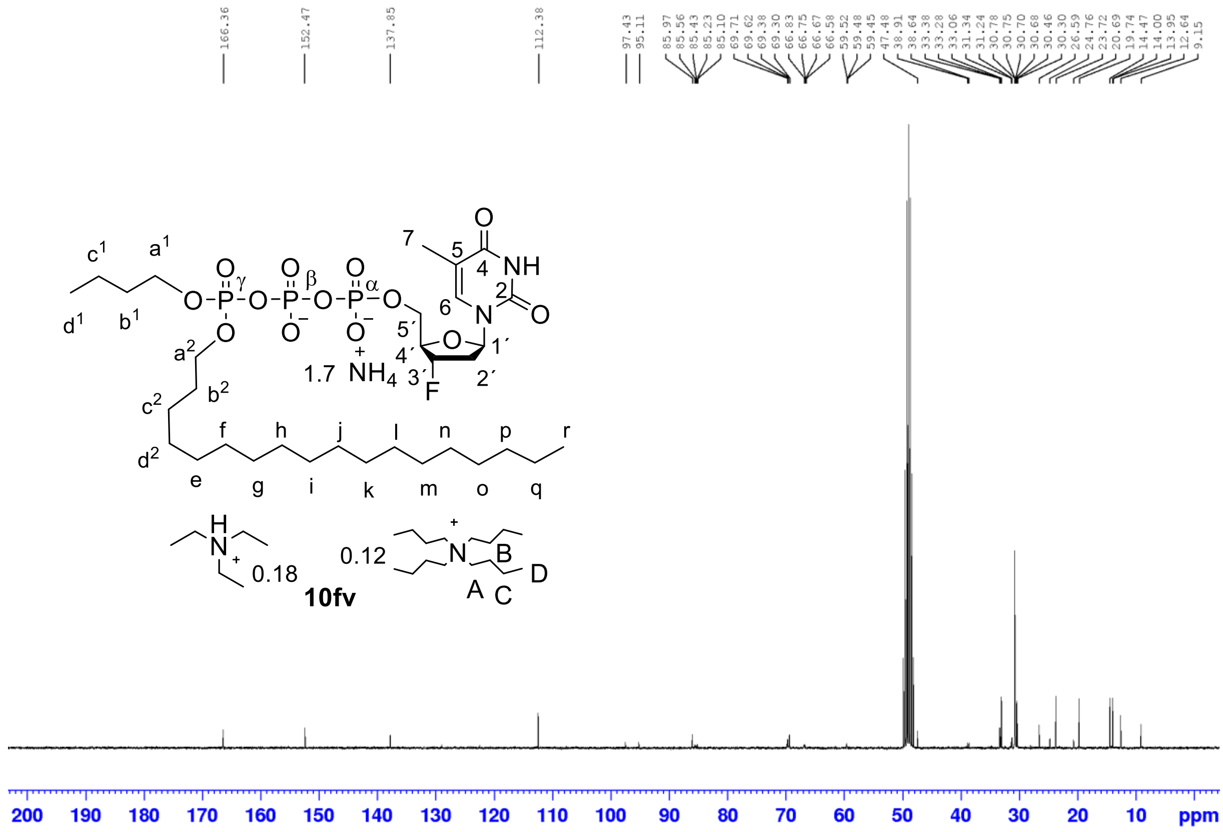

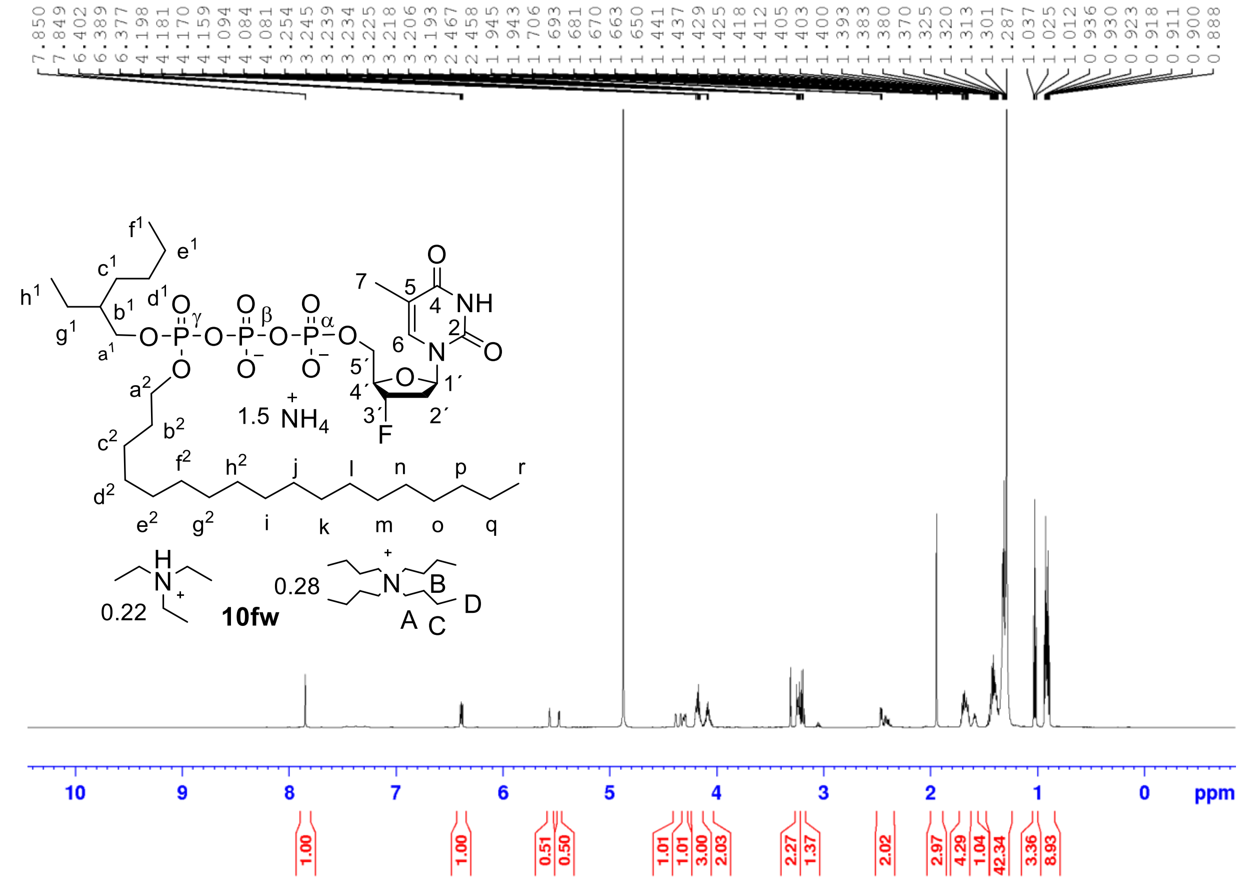

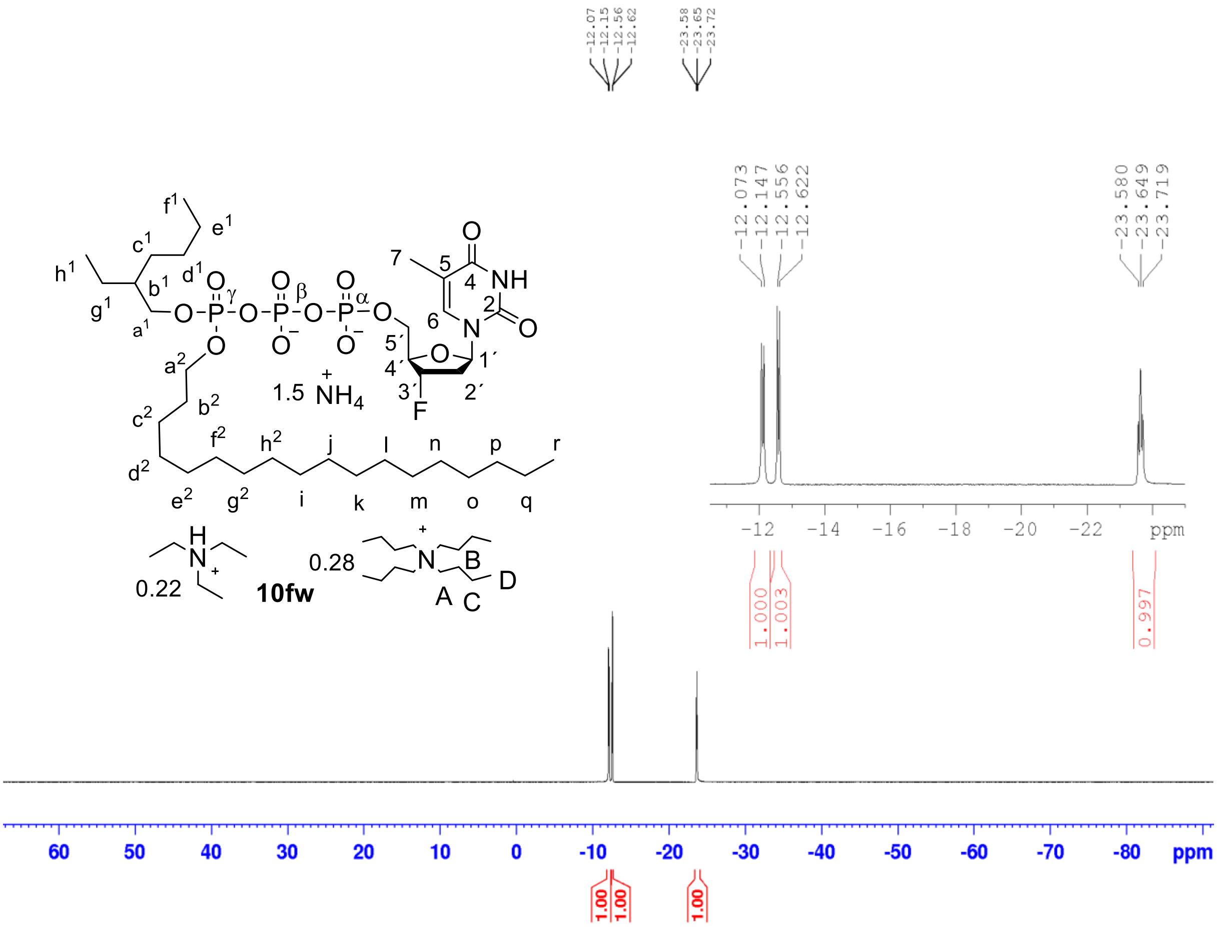
-12.07
-12.15
-12.56
-12.62
-23.58
-23.65
-23.72
-12.073
-12.147
-12.556
-12.622
-23.580
-23.649
-23.719
1.5 NH4
0.22
0.28
10fw
1.000
1.003
0.997
ppm
1.00
1.00
1.00

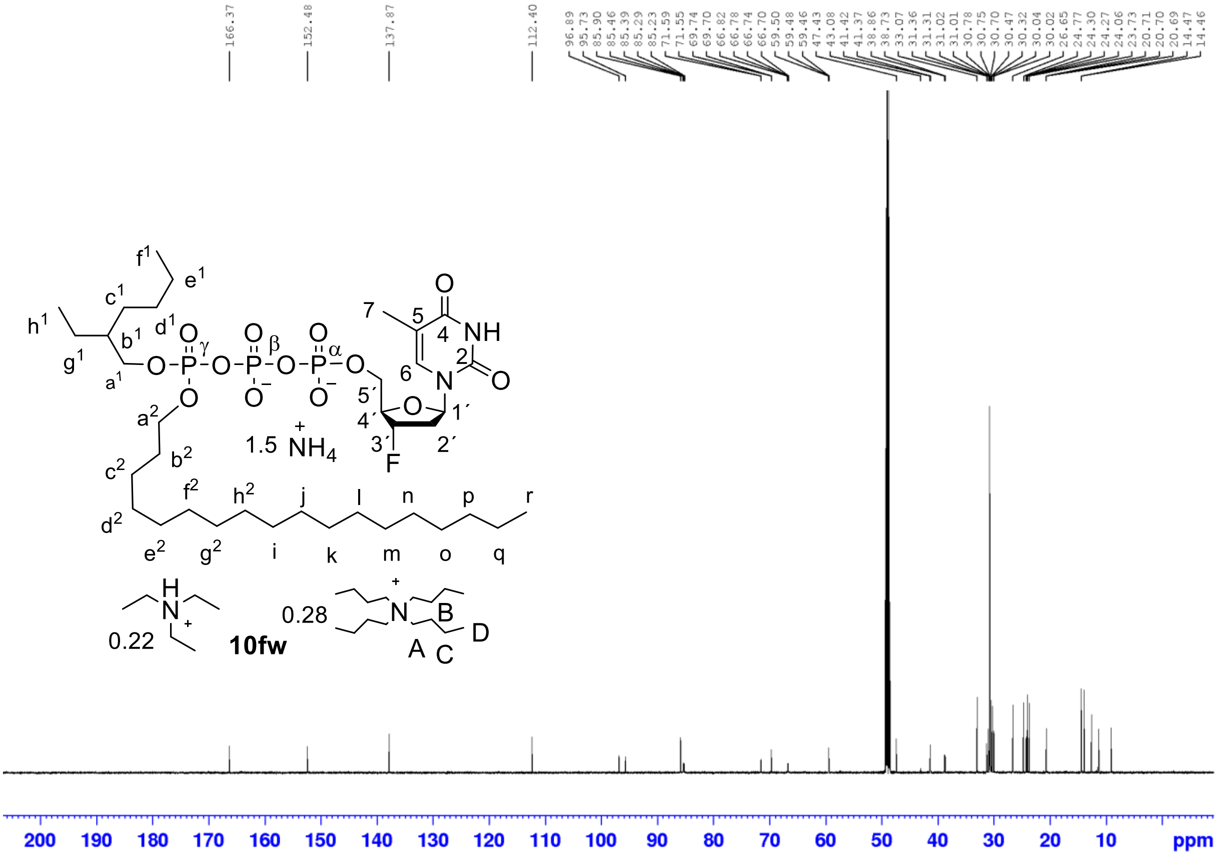
166.37
152.48
137.87
112.40
96.89
95.73
85.90
85.46
85.39
85.29
85.23
71.55
69.74
69.70
66.82
66.78
66.74
66.70
59.50
59.48
59.46
47.43
43.08
41.42
41.37
38.86
38.73
33.07
31.36
31.31
31.02
31.01
30.78
30.75
30.70
30.47
30.32
30.04
30.02
26.65
24.77
24.30
24.27
24.06
23.73
20.71
20.70
20.69
14.47
14.46
1.5 NH4
0.22
0.28
10fw
ppm

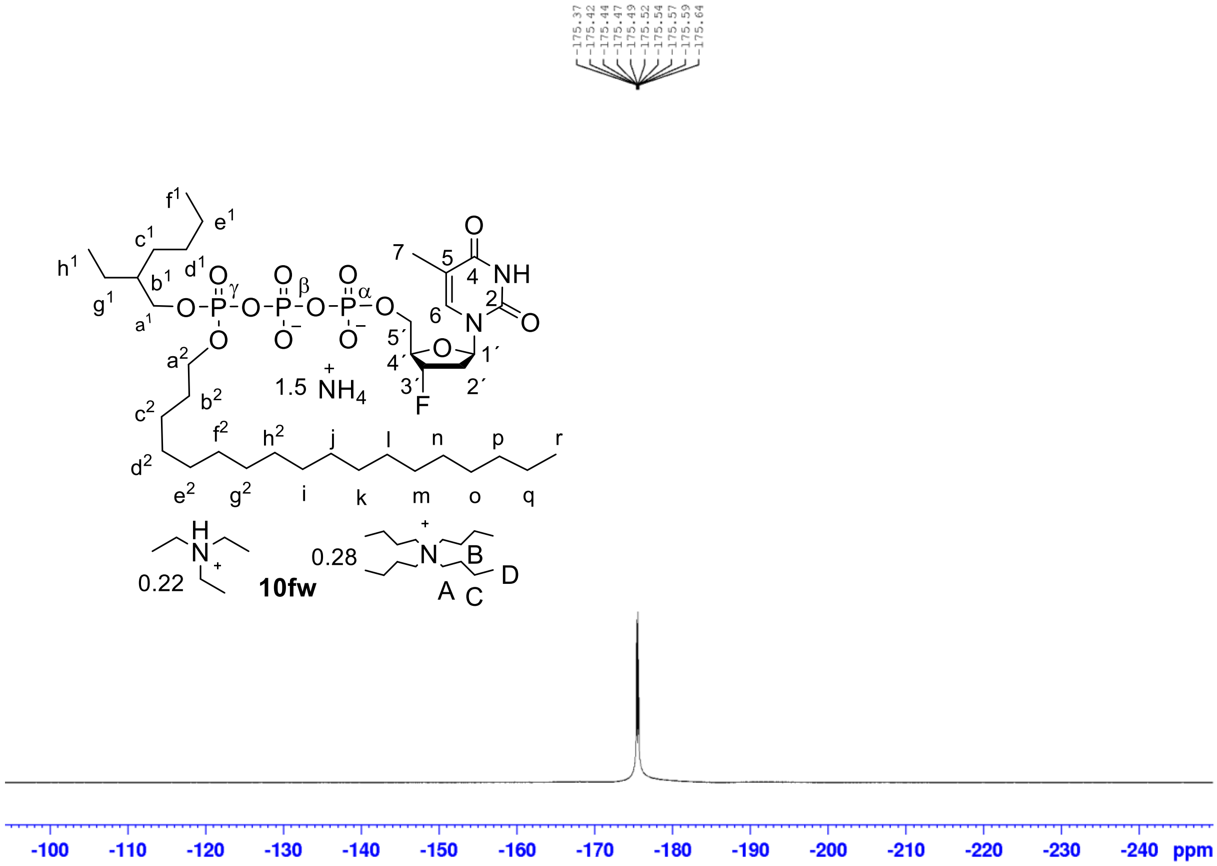

-175.37
-175.42
-175.44
-175.47
-175.49
-175.52
-175.54
-175.57
-175.59
-175.64
1.5 NH4
0.22
10fw
0.28
A B C D
-100 -110 -120 -130 -140 -150 -160 -170 -180 -190 -200 -210 -220 -230 -240 ppm

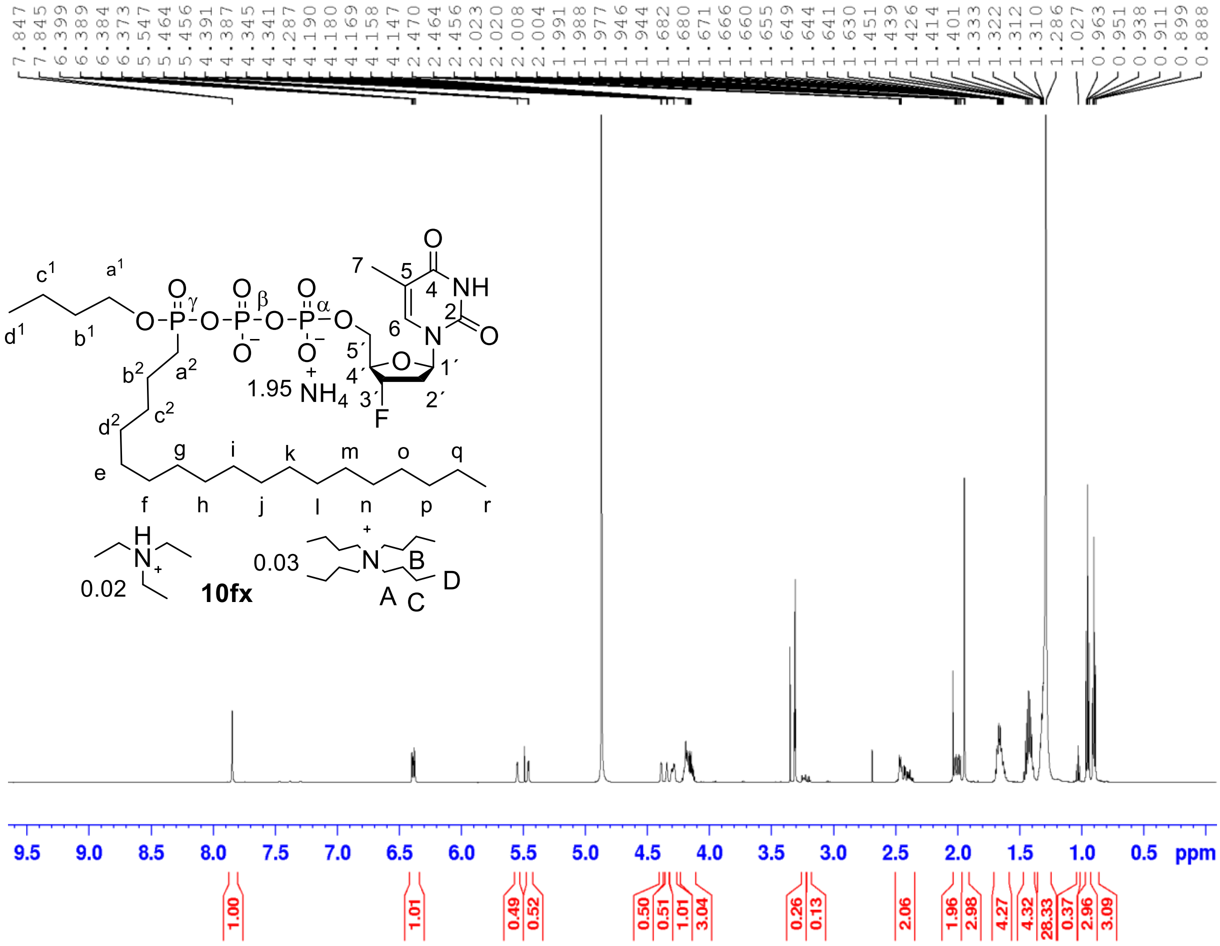

7.847
7.845
6.399
6.389
6.384
6.373
5.547
5.464
5.456
4.391
4.387
4.345
4.341
4.287
4.190
4.180
4.169
4.158
4.147
2.470
2.464
2.456
2.023
2.020
2.008
2.004
1.991
1.988
1.977
1.946
1.944
1.682
1.680
1.671
1.666
1.660
1.655
1.649
1.644
1.641
1.630
1.451
1.439
1.426
1.414
1.401
1.333
1.322
1.312
1.310
1.286
1.027
0.963
0.951
0.938
0.911
0.899
0.888
1.95 NH4
0.02
10fx
0.03
A B C D
9.5 9.0 8.5 8.0 7.5 7.0 6.5 6.0 5.5 5.0 4.5 4.0 3.5 3.0 2.5 2.0 1.5 1.0 0.5 ppm
1.00
1.01
0.49
0.52
0.50
0.51
1.01
3.04
0.26
0.13
2.06
1.96
2.98
4.27
4.32
28.33
0.37
2.96
3.09

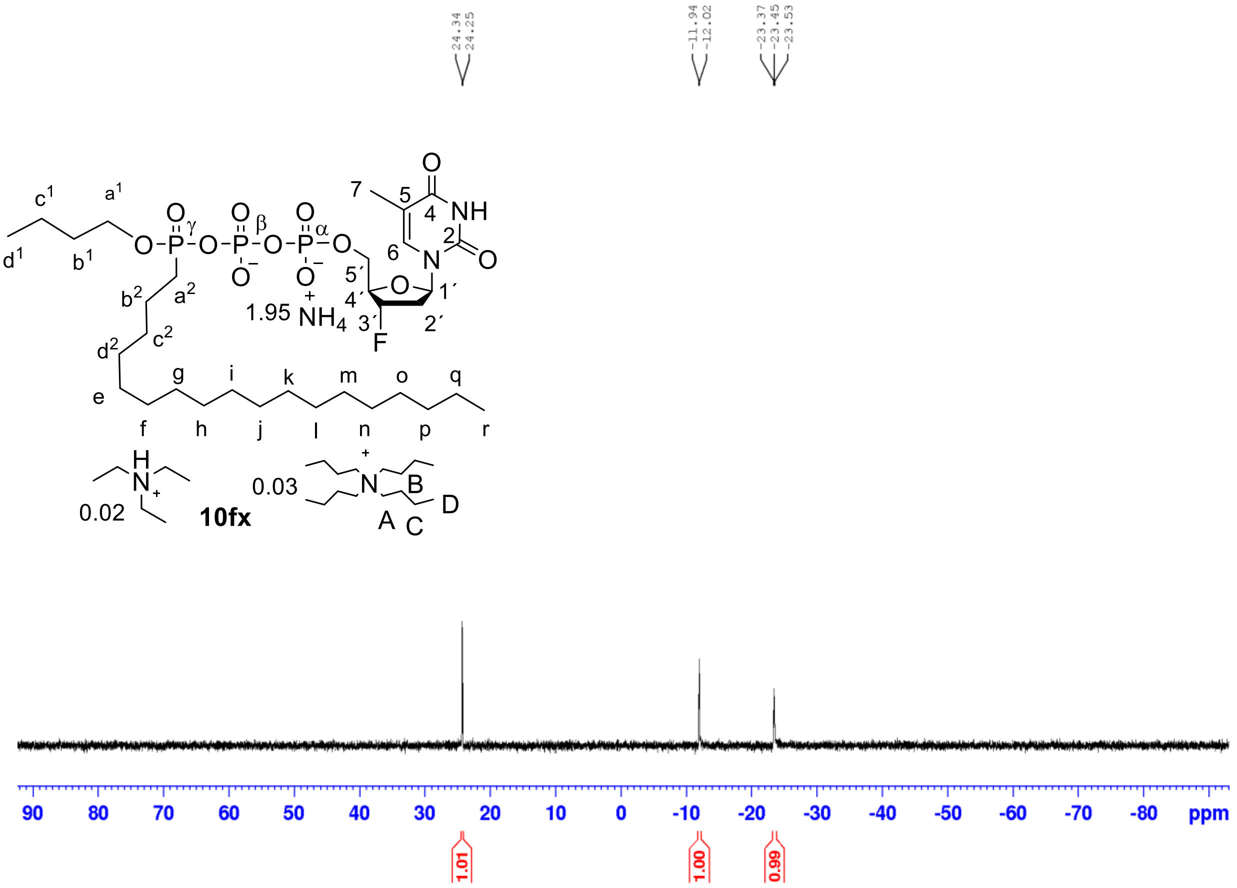
24.34
24.25
-11.94
-12.02
-23.37
-23.45
-23.53
1.95 NH4
0.02
0.03
10fx
90 80 70 60 50 40 30 20 10 0 -10 -20 -30 -40 -50 -60 -70 -80 ppm
1.01
1.00
0.99

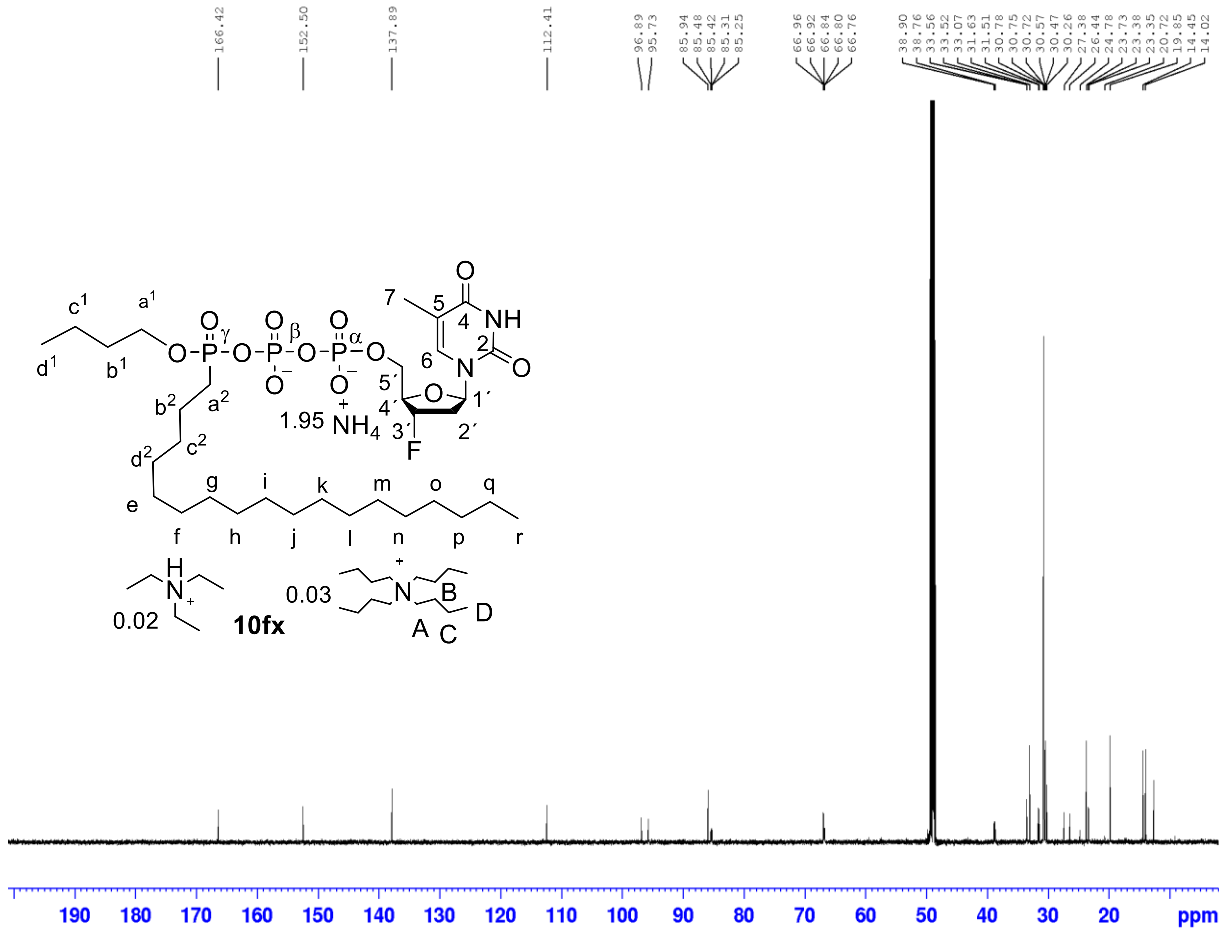
166.42
152.50
137.89
112.41
96.89
95.73
85.94
85.48
85.42
85.31
85.25
66.96
66.92
66.84
66.80
66.76
38.90
38.76
33.56
33.52
33.07
31.63
31.51
30.78
30.75
30.72
30.57
30.47
30.26
27.38
26.44
24.78
23.73
23.38
23.35
20.72
19.85
14.45
14.02
1.95 NH4
0.02
0.03
10fx
190 180 170 160 150 140 130 120 110 100 90 80 70 60 50 40 30 20 ppm

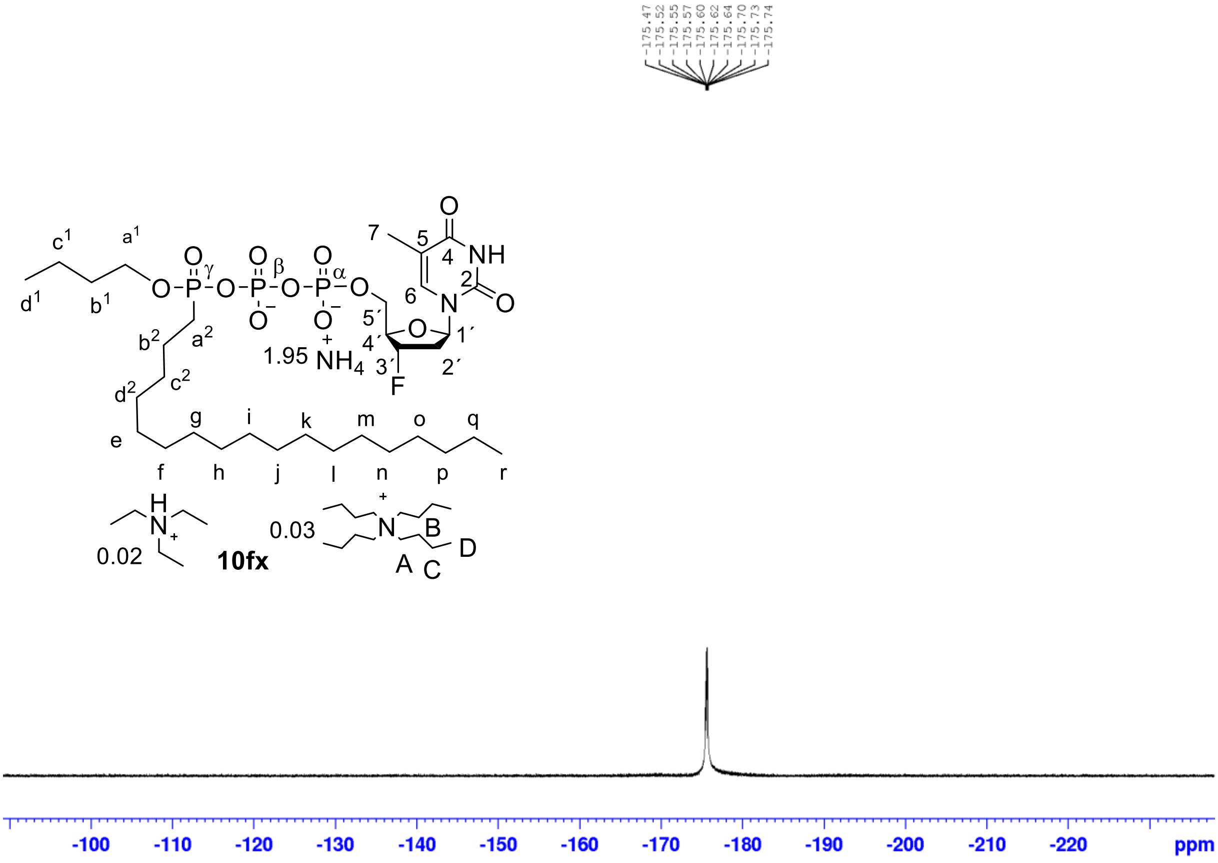
-175.47
-175.52
-175.55
-175.57
-175.60
-175.62
-175.64
-175.70
-175.73
-175.74
1.95
0.02
10fx
0.03
-100 -110 -120 -130 -140 -150 -160 -170 -180 -190 -200 -210 -220 ppm

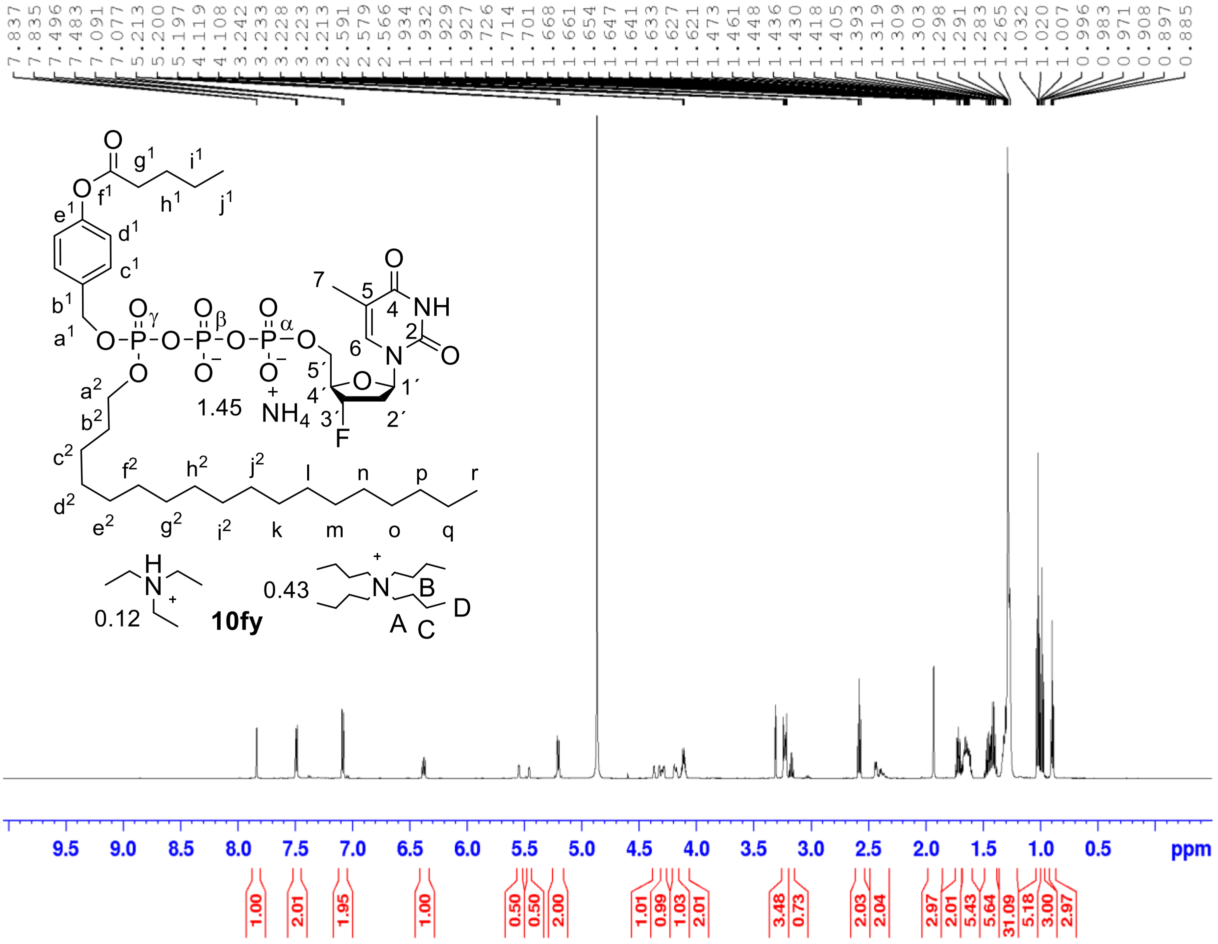
7.837 7.835 7.496 7.483 7.091 7.077 5.213 5.200 5.197 5.119 4.108 3.242 3.233 3.228 3.223 3.213 2.591 2.579 2.566 1.934 1.932 1.929 1.927 1.726 1.714 1.701 1.668 1.661 1.654 1.647 1.641 1.633 1.627 1.621 1.473 1.461 1.448 1.436 1.430 1.418 1.405 1.393 1.319 1.309 1.303 1.298 1.291 1.283 1.265 1.032 1.020 1.007 0.996 0.983 0.971 0.908 0.897 0.885
1.45
0.12
10fy
0.43
9.5 9.0 8.5 8.0 7.5 7.0 6.5 6.0 5.5 5.0 4.5 4.0 3.5 3.0 2.5 2.0 1.5 1.0 0.5 ppm
1.00 2.01 1.95 1.00 0.50 0.50 2.00 1.01 0.99 1.03 2.01 3.48 0.73 2.03 2.04 2.97 2.01 5.43 5.64 31.09 5.18 3.00 2.97

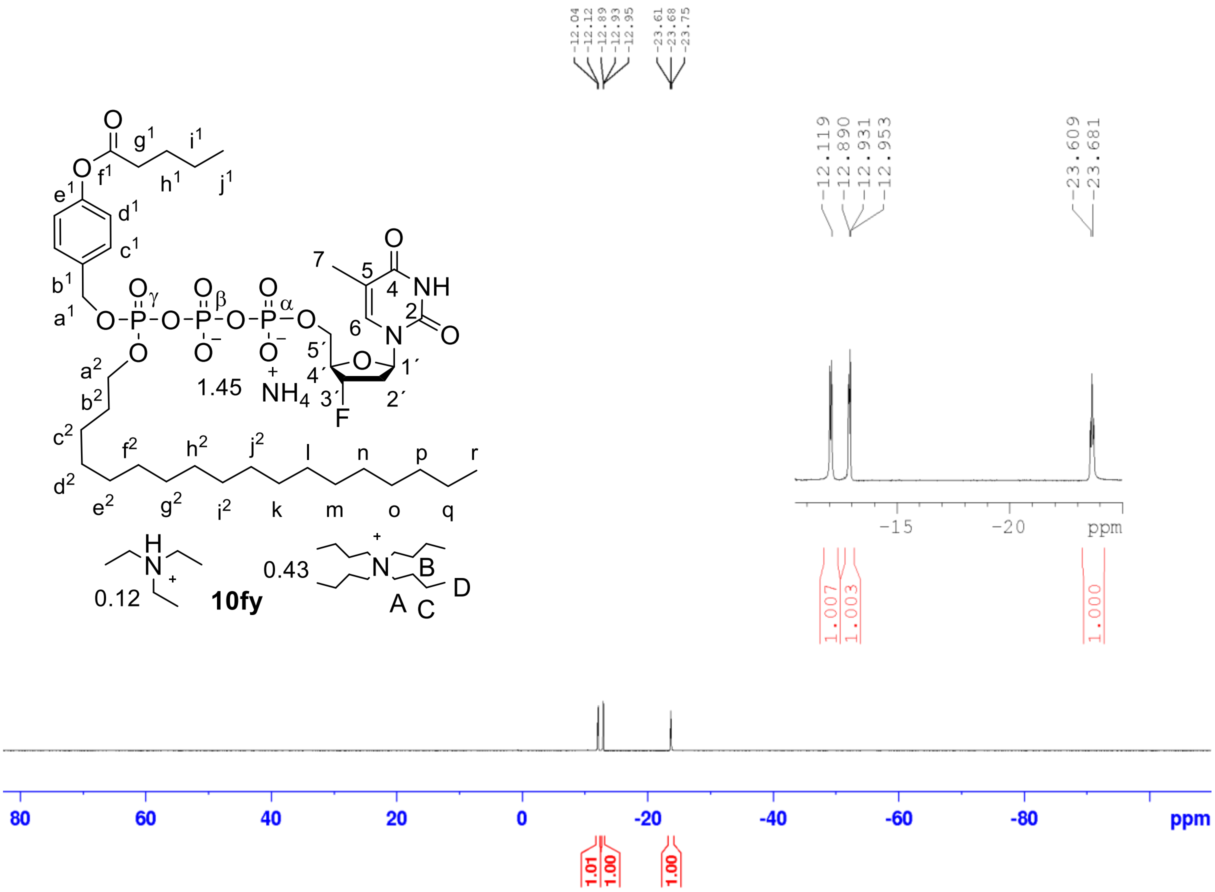
-12.04
-12.12
-12.89
-12.93
-12.95
-23.61
-23.68
-23.75
-12.119
-12.890
-12.931
-12.953
-23.609
-23.681
1.45
0.43
0.12
10fy
1.007
1.003
1.000
1.01
1.00
1.00
ppm

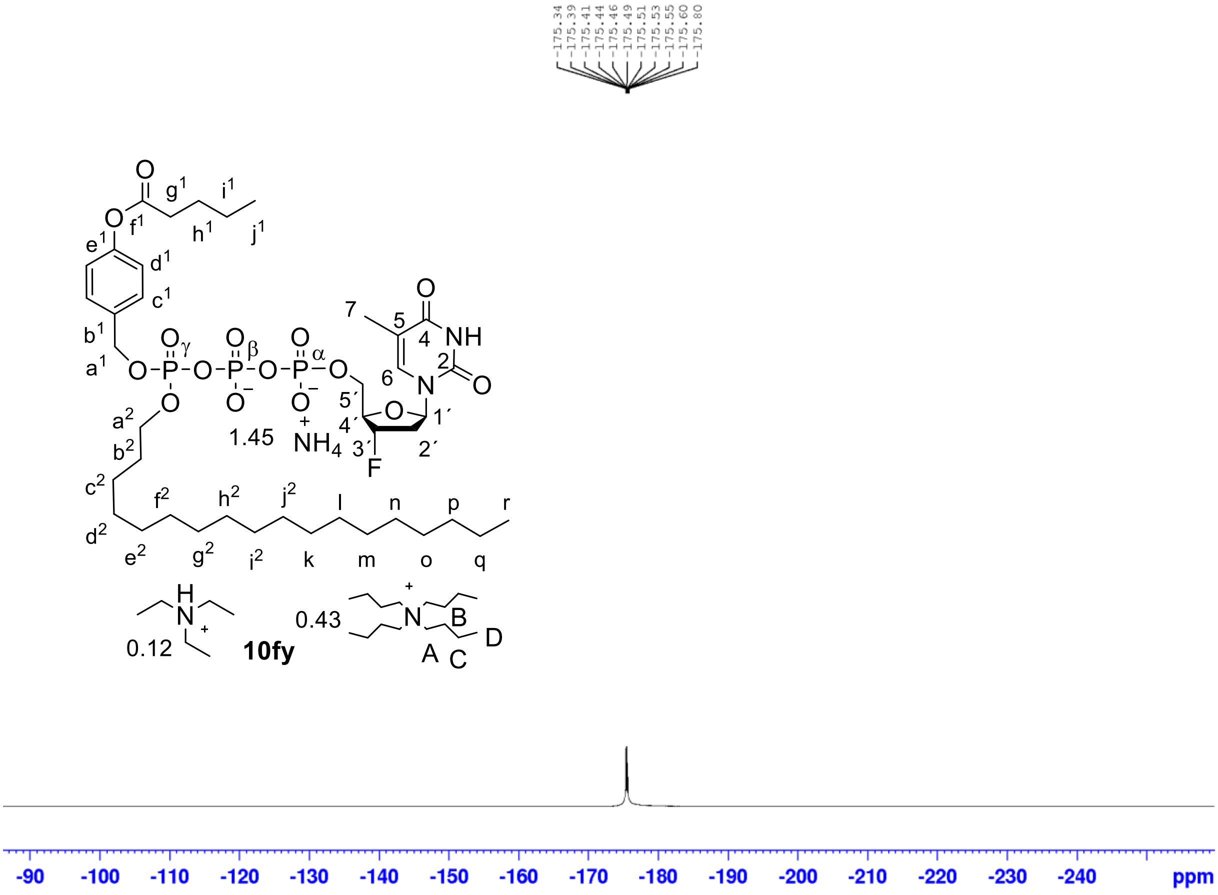
-175.34
-175.39
-175.41
-175.44
-175.46
-175.49
-175.51
-175.53
-175.55
-175.60
-175.80
1.45
0.43
0.12
10fy
ppm

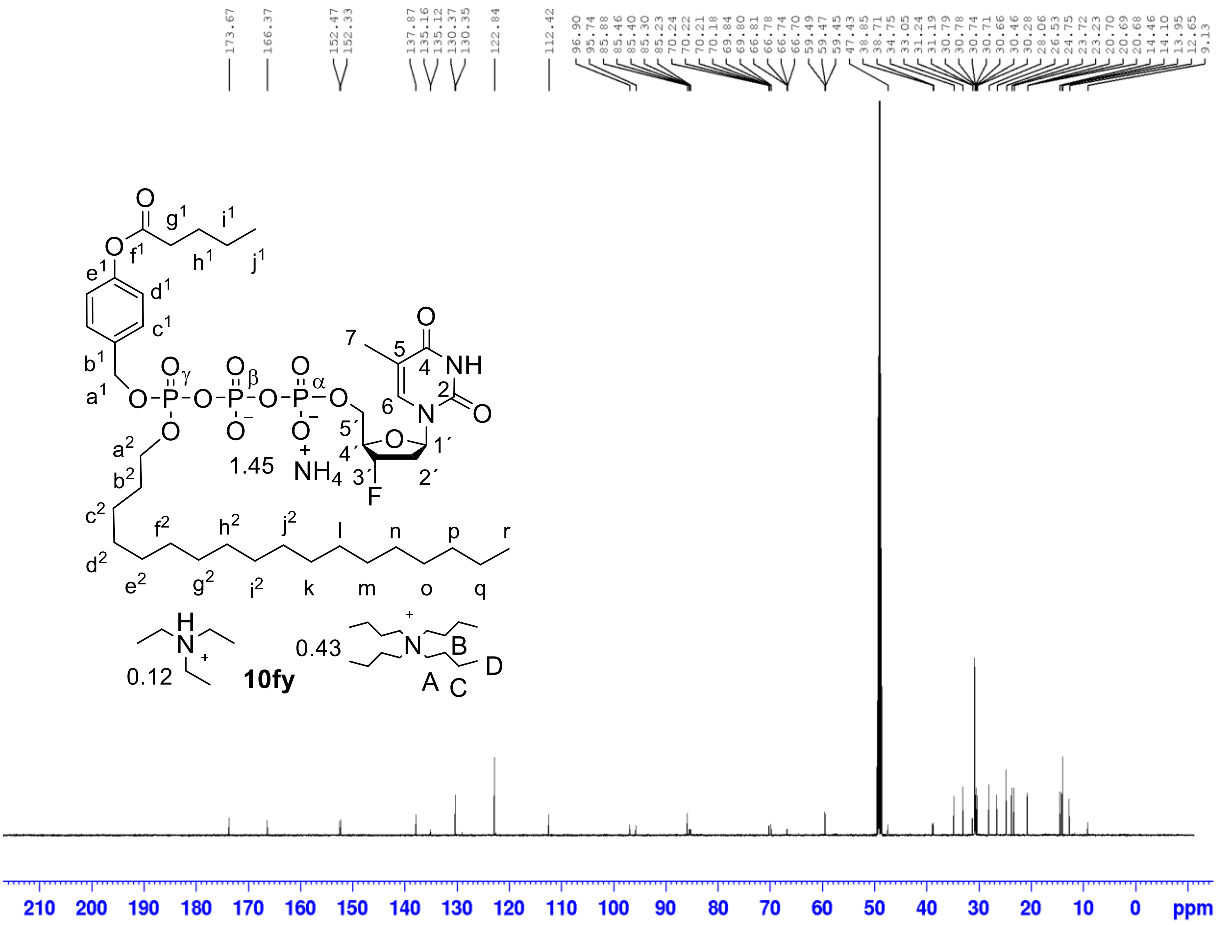
173.67
166.37
152.47
152.33
137.87
135.16
135.12
130.37
130.35
122.84
112.42
96.90
95.74
85.88
85.46
85.40
85.30
85.23
70.24
70.22
70.21
70.18
69.84
69.80
66.81
66.78
66.74
66.70
59.49
59.47
59.45
47.43
38.85
38.71
34.75
33.05
31.24
31.19
30.79
30.74
30.71
30.66
30.46
30.28
28.06
26.53
24.75
23.72
23.23
20.70
20.69
20.68
14.46
14.10
13.95
12.65
9.13
1.45
0.43
0.12
10fy
A
B
C
D
210 200 190 180 170 160 150 140 130 120 110 100 90 80 70 60 50 40 30 20 10 0 ppm

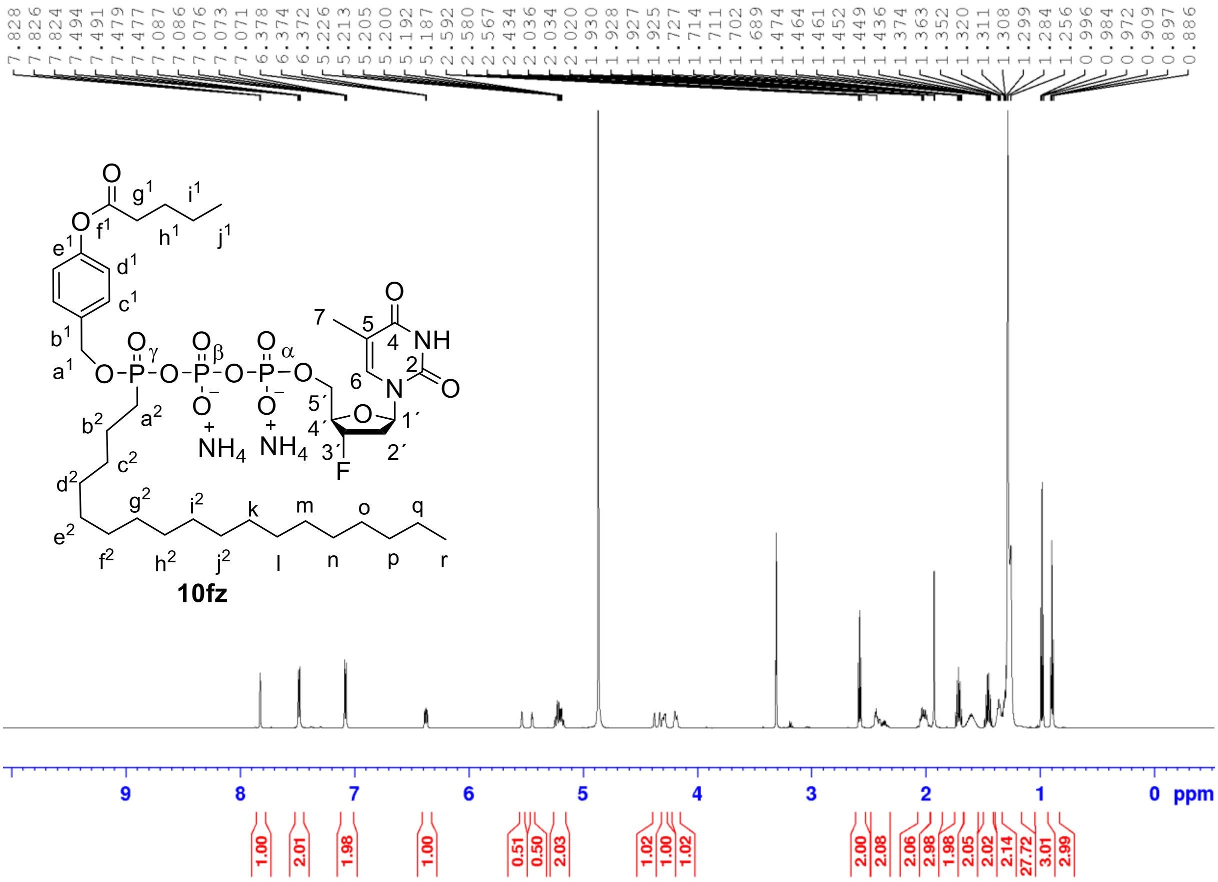
7.828
7.826
7.824
7.494
7.491
7.479
7.477
7.087
7.086
7.076
7.073
7.071
6.378
6.374
6.372
5.226
5.213
5.205
5.200
5.192
5.187
2.592
2.580
2.567
2.434
2.036
2.034
2.020
1.930
1.928
1.927
1.925
1.727
1.714
1.711
1.702
1.689
1.474
1.464
1.461
1.452
1.449
1.436
1.374
1.363
1.352
1.320
1.311
1.308
1.299
1.284
1.256
0.996
0.984
0.972
0.909
0.897
0.886
10fz
9 8 7 6 5 4 3 2 1 0 ppm
1.00
2.01
1.98
1.00
0.51
0.50
2.03
1.02
1.00
1.02
2.00
2.08
2.06
2.98
1.98
2.05
2.02
2.14
27.72
3.01
2.99

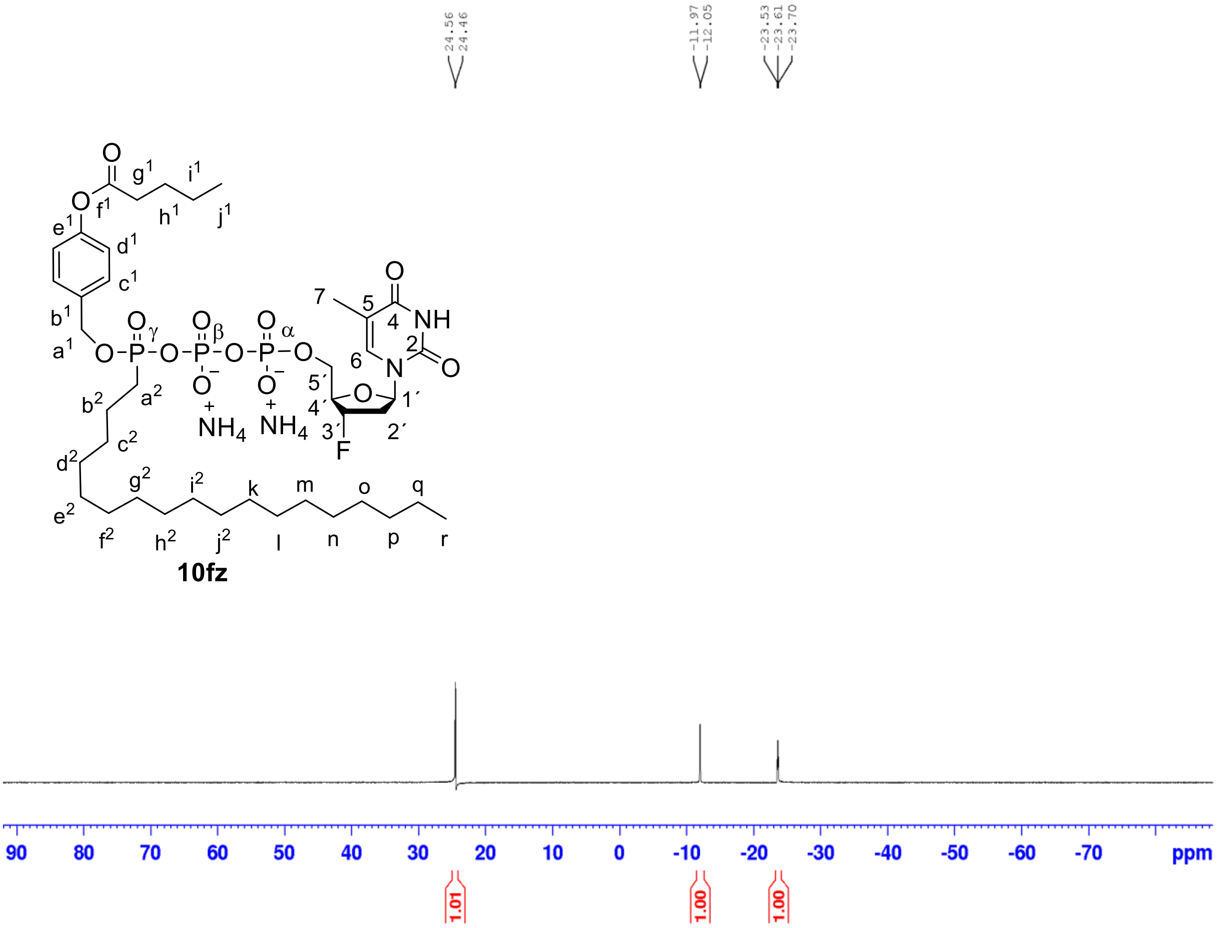

24.56
24.46
-11.97
-12.05
-23.53
-23.61
-23.70
10fz
90 80 70 60 50 40 30 20 10 0 -10 -20 -30 -40 -50 -60 -70 ppm
1.01
1.00
1.00

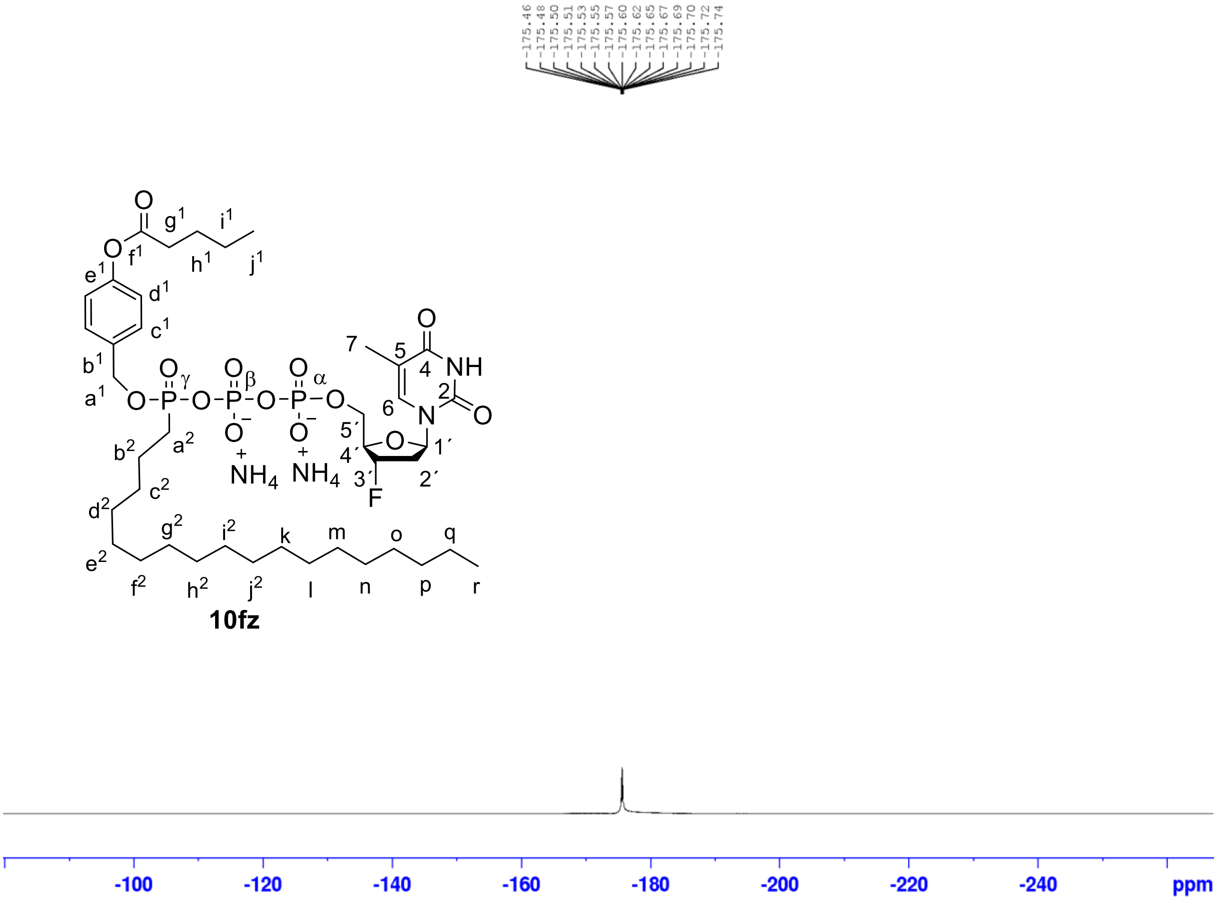

-175.46
-175.48
-175.50
-175.51
-175.53
-175.55
-175.57
-175.60
-175.62
-175.65
-175.67
-175.69
-175.70
-175.72
-175.74
10fz
-100 -120 -140 -160 -180 -200 -220 -240 ppm

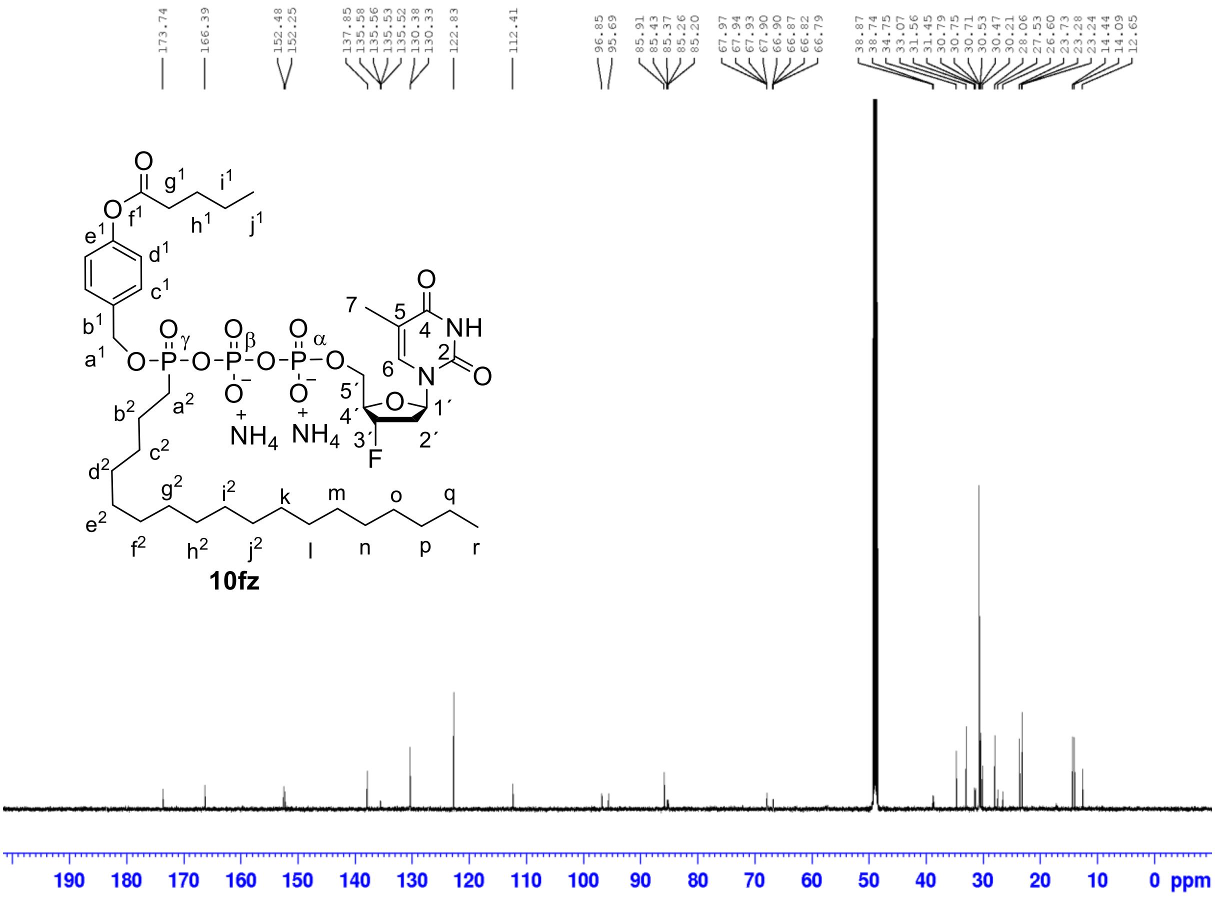
173.74
166.39
152.48
152.25
137.85
135.58
135.56
135.53
135.52
130.38
130.33
122.83
112.41
96.85
95.69
85.91
85.43
85.37
85.26
85.20
67.97
67.94
67.93
67.90
66.90
66.87
66.82
66.79
38.87
38.74
34.75
33.07
31.56
31.45
30.79
30.75
30.71
30.53
30.47
30.21
28.06
27.53
26.60
23.73
23.28
23.24
14.44
14.09
12.65
10fz
ppm

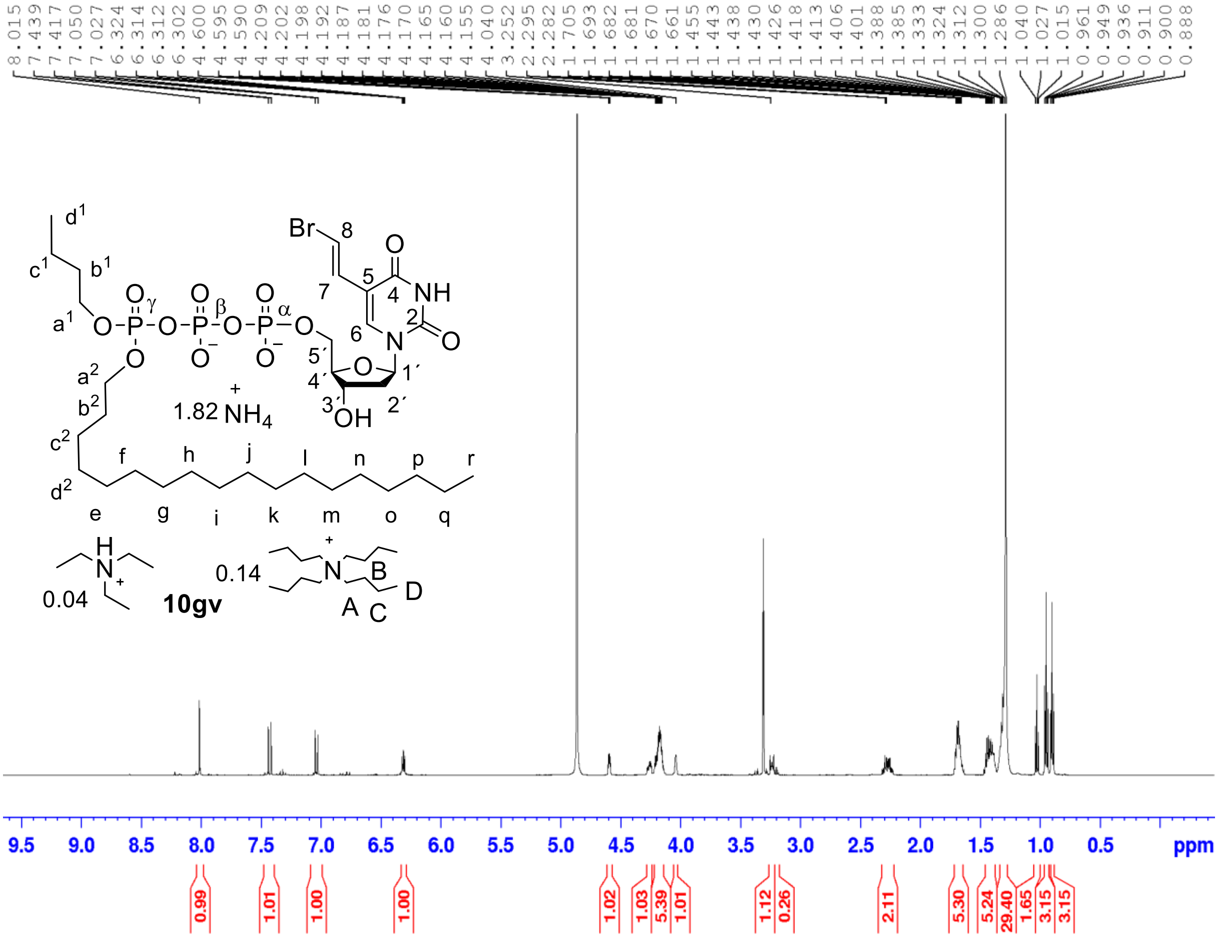
8.015
7.439
7.417
7.050
7.027
6.324
6.314
6.312
6.302
4.600
4.595
4.590
4.209
4.202
4.198
4.192
4.187
4.181
4.176
4.170
4.165
4.160
4.155
4.040
3.252
2.295
2.282
1.705
1.693
1.682
1.681
1.670
1.661
1.455
1.443
1.438
1.430
1.426
1.418
1.413
1.406
1.401
1.388
1.385
1.333
1.324
1.312
1.300
1.286
1.040
1.027
1.015
0.961
0.949
0.936
0.911
0.900
0.888
10gv
1.82 NH4
0.04
0.14
ppm
0.99
1.01
1.00
1.00
1.02
1.03
5.39
1.01
1.12
0.26
2.11
5.30
5.24
29.40
1.65
3.15
3.15

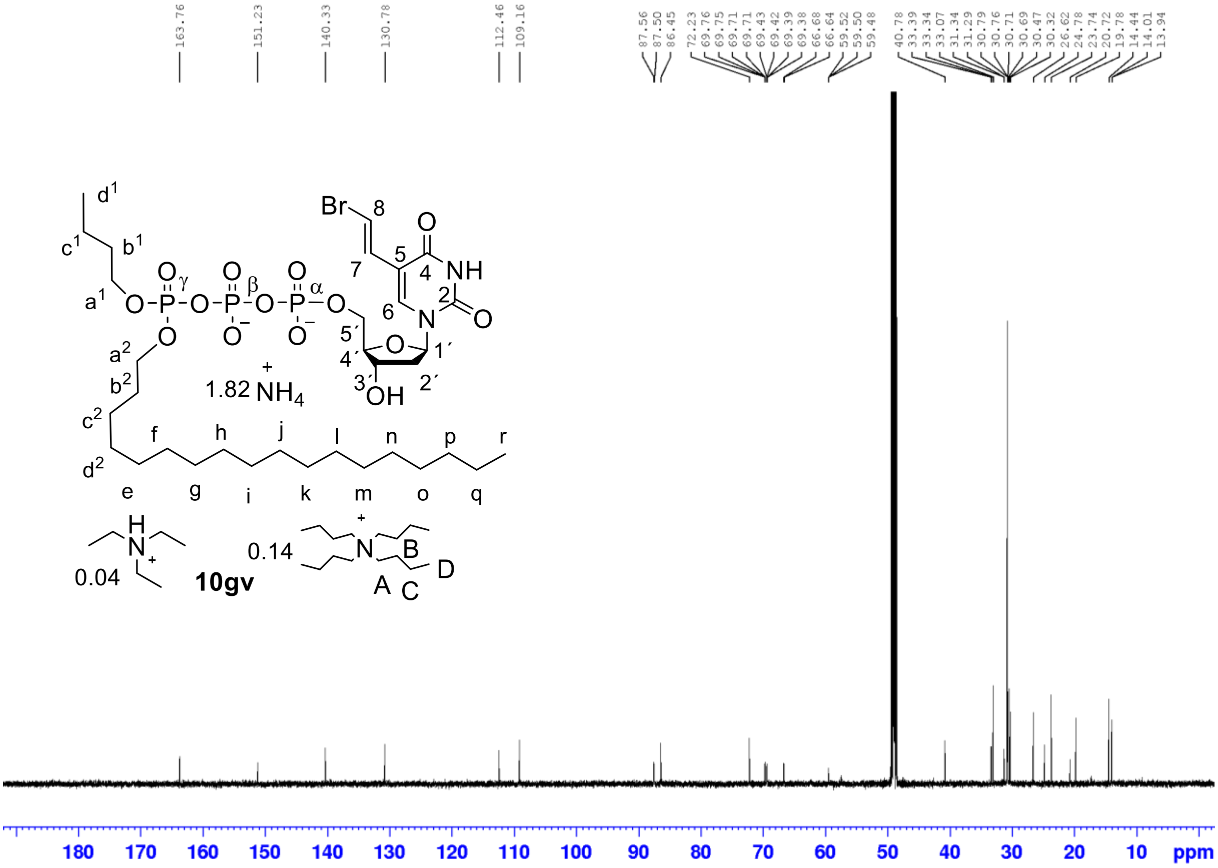
163.76
151.23
140.33
130.78
112.46
109.16
87.56 87.50 86.45 72.23 69.76 69.75 69.71 69.71 69.43 69.42 69.39 69.38 66.68 66.64 59.52 59.50 59.48
40.78 33.39 33.34 33.07 31.34 31.29 30.79 30.76 30.71 30.69 30.47 30.32 26.62 24.78 23.74 20.72 19.78 14.44 14.01 13.94
1.82 NH4
0.04
0.14
10gv
ppm

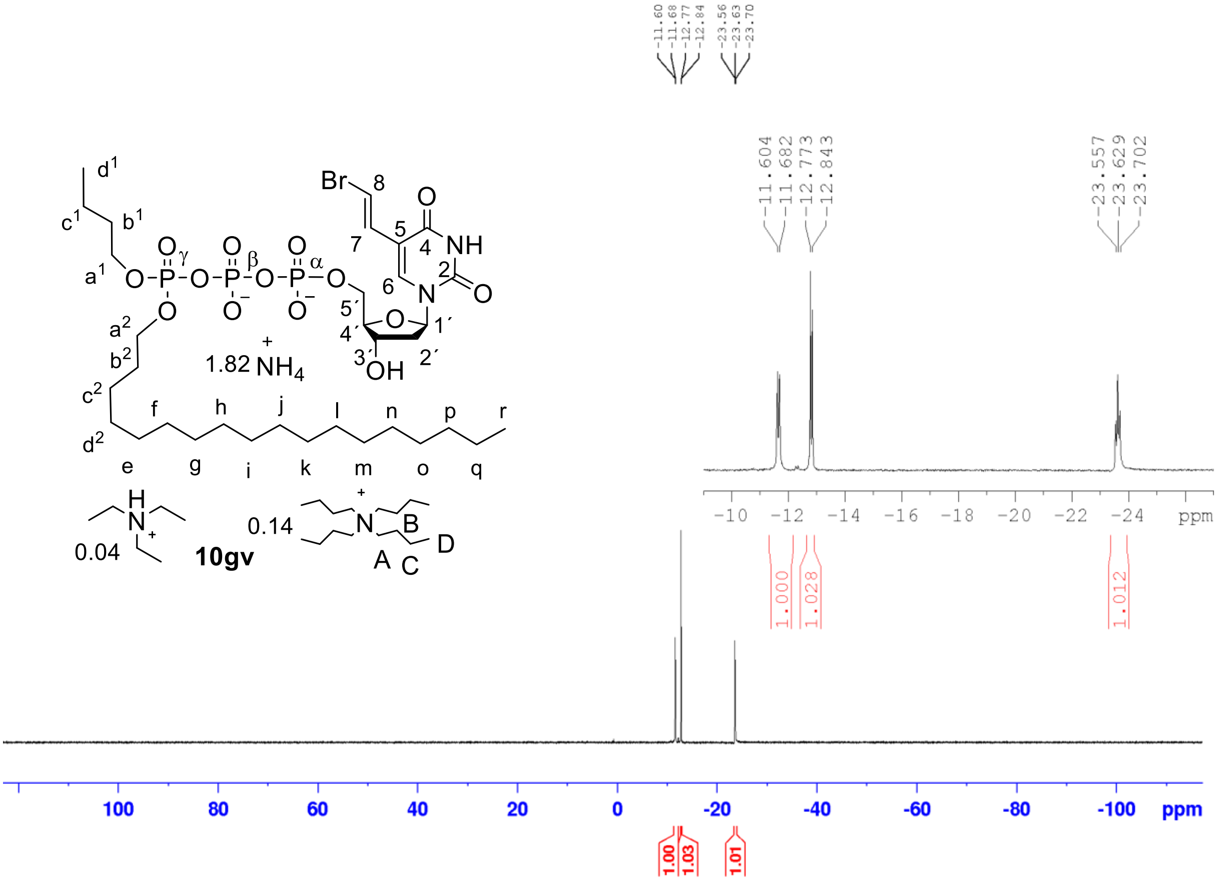
-11.60 -11.68 -12.77 -12.84 -23.56 -23.63 -23.70
-11.604 -11.682 -12.773 -12.843
-23.557 -23.629 -23.702
1.000 1.028 1.012
1.82 NH4
0.04
0.14
10gv
1.00 1.03 1.01
ppm

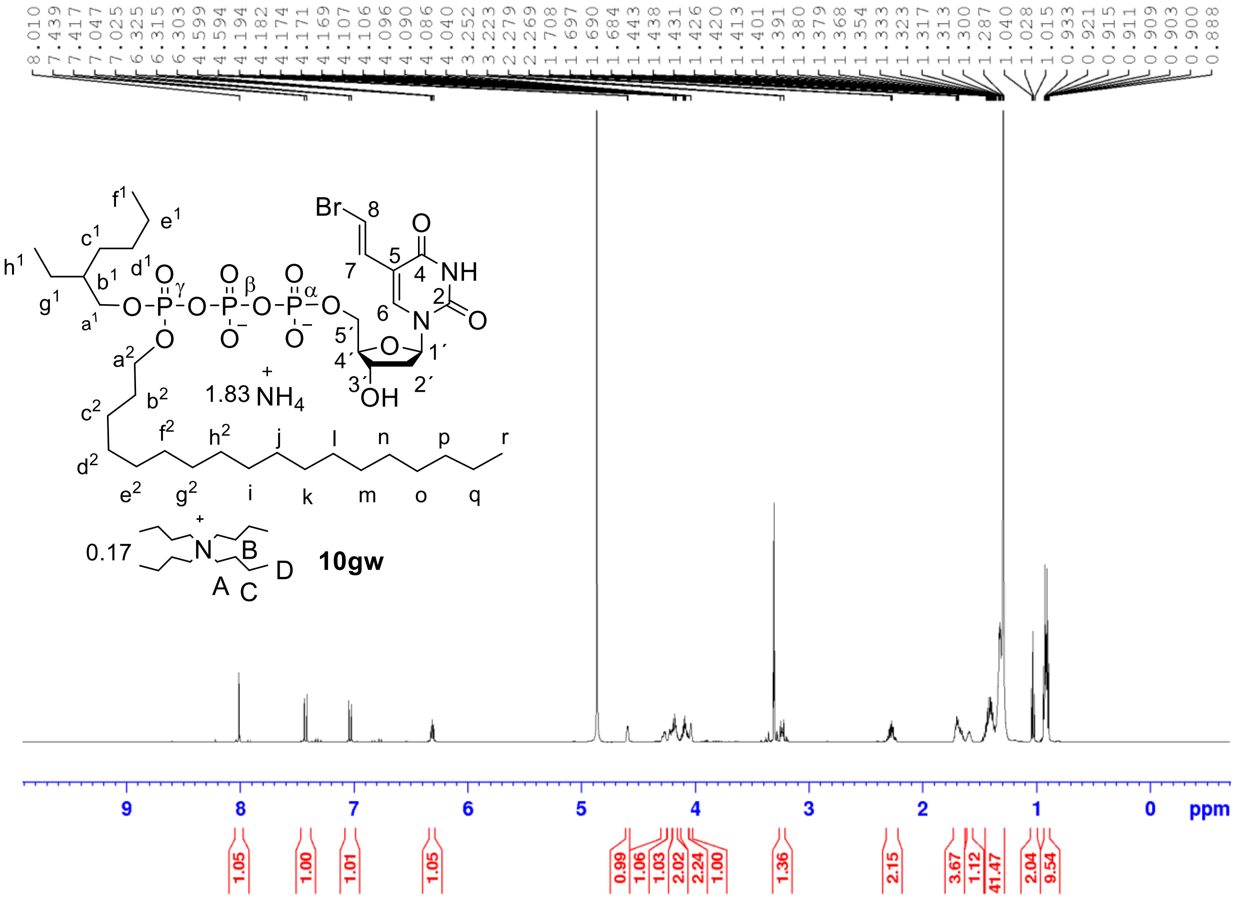
8.010 7.439 7.417 7.047 7.025 6.325 6.315 6.303 4.599 4.594 4.194 4.182 4.174 4.171 4.169 4.107 4.106 4.096 4.090 4.086 4.040 3.252 3.223 2.279 2.269 1.708 1.697 1.690 1.684 1.443 1.438 1.431 1.426 1.420 1.413 1.401 1.391 1.380 1.379 1.368 1.354 1.333 1.323 1.317 1.313 1.300 1.287 1.040 1.028 1.015 0.933 0.921 0.915 0.911 0.909 0.903 0.900 0.888
1.83 NH4
0.17
10gw
ppm
1.05 1.00 1.01 1.05 0.99 1.06 1.03 2.02 2.24 1.00 1.36 2.15 3.67 1.12 41.47 2.04 9.54

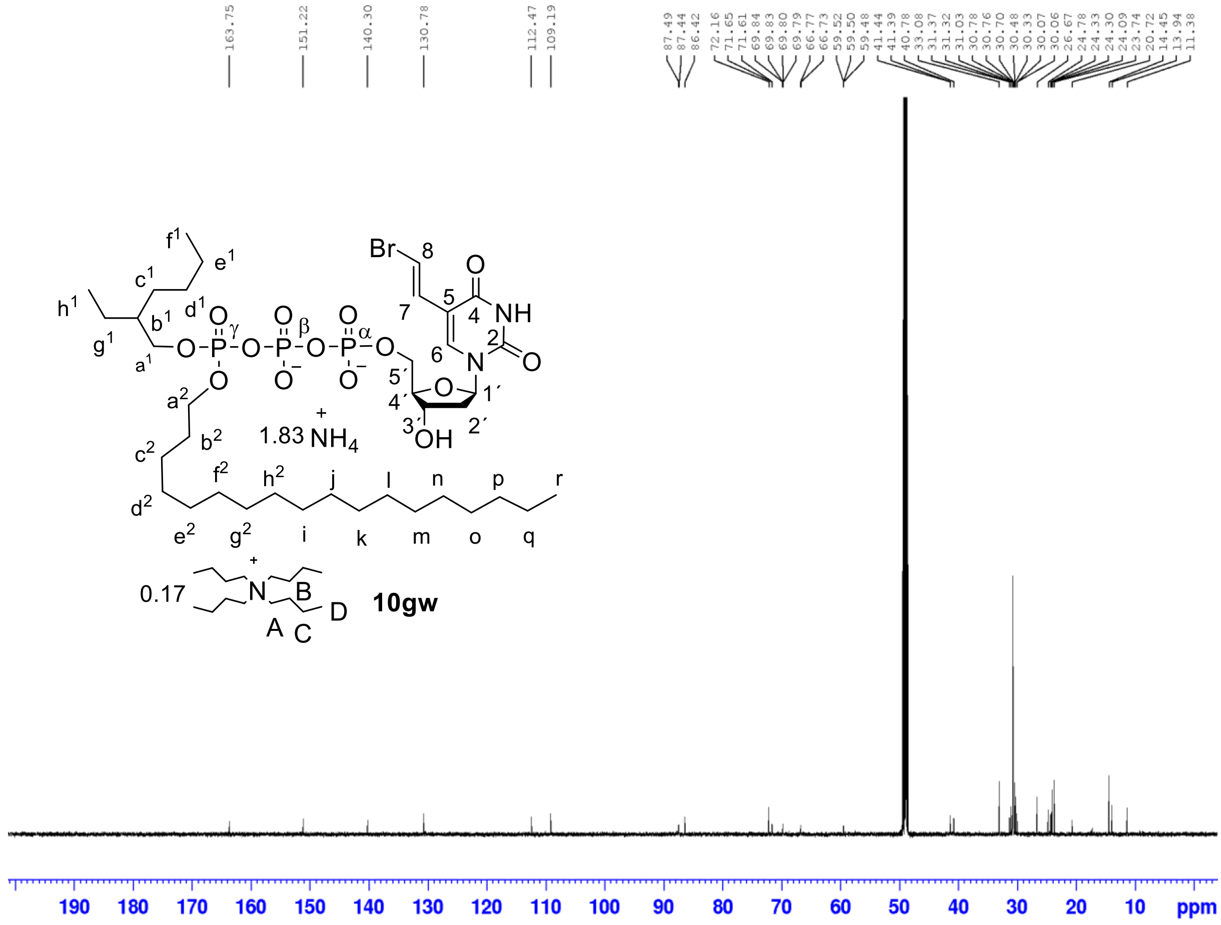
163.75 151.22 140.30 130.78 112.47 109.19 87.49 87.44 86.42 72.16 71.65 71.61 69.84 69.83 69.80 69.79 66.77 66.73 59.52 59.50 59.48 41.44 41.39 40.78 33.08 31.37 31.32 31.03 30.78 30.76 30.70 30.48 30.33 30.07 30.06 26.67 24.78 24.33 24.30 24.09 23.74 20.72 14.45 13.94 11.38
1.83 NH4
0.17
10gw
ppm

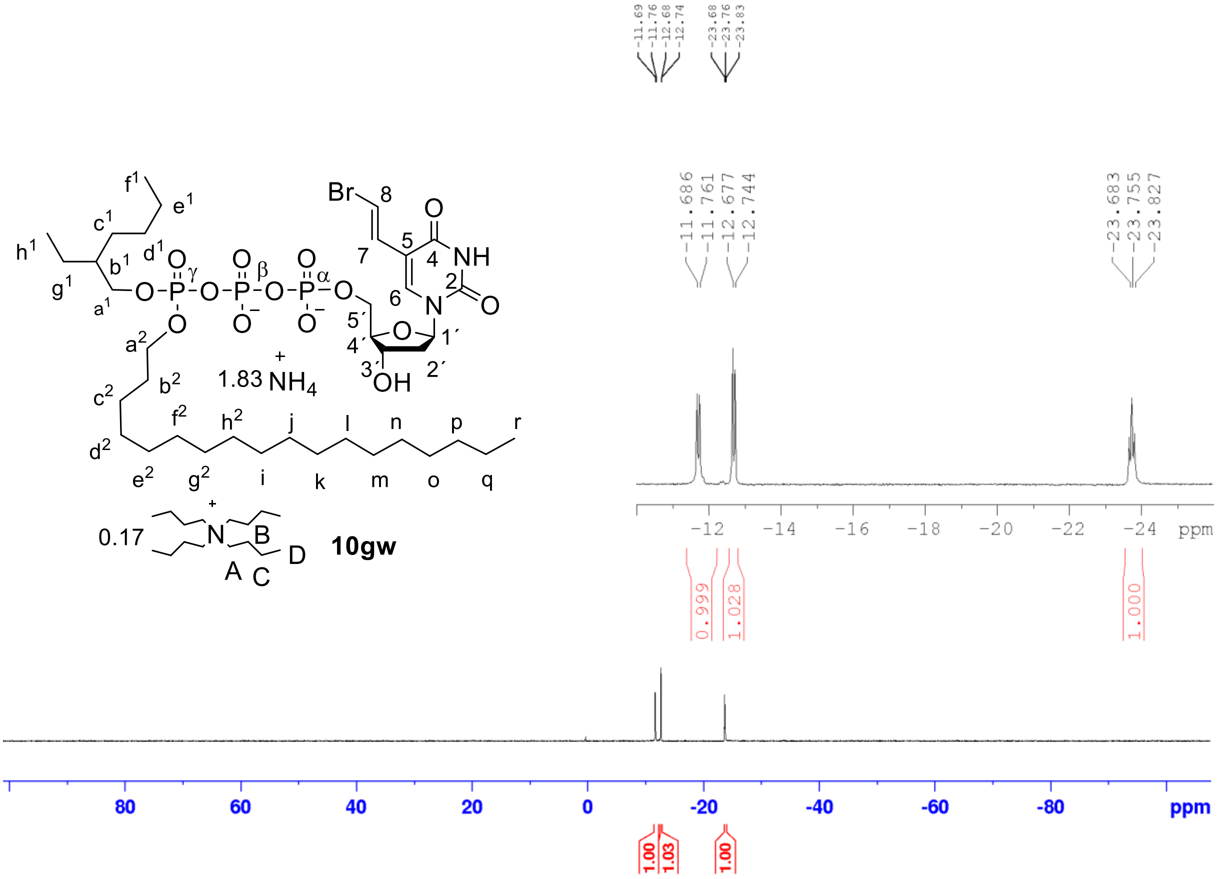

Br
NH
1.83 NH4
0.17
10gw
-11.686
-11.761
-12.677
-12.744
-23.683
-23.755
-23.827
ppm
0.999
1.028
1.000
1.00
1.03
1.00

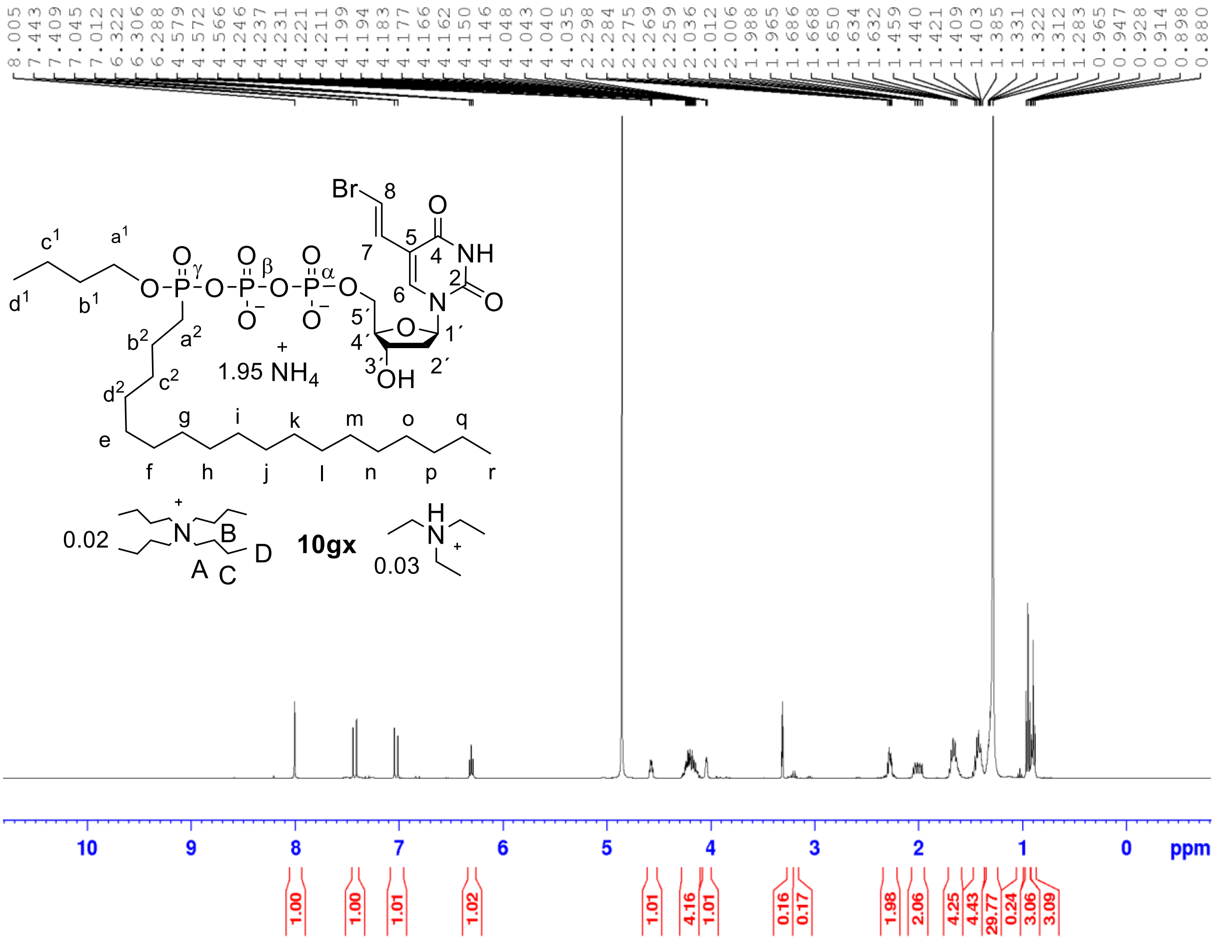

Br
NH
1.95 NH4
0.02
10gx
0.03
ppm
1.00
1.00
1.01
1.02
1.01
4.16
1.01
0.16
0.17
1.98
2.06
4.25
4.43
29.77
0.24
3.06
3.09

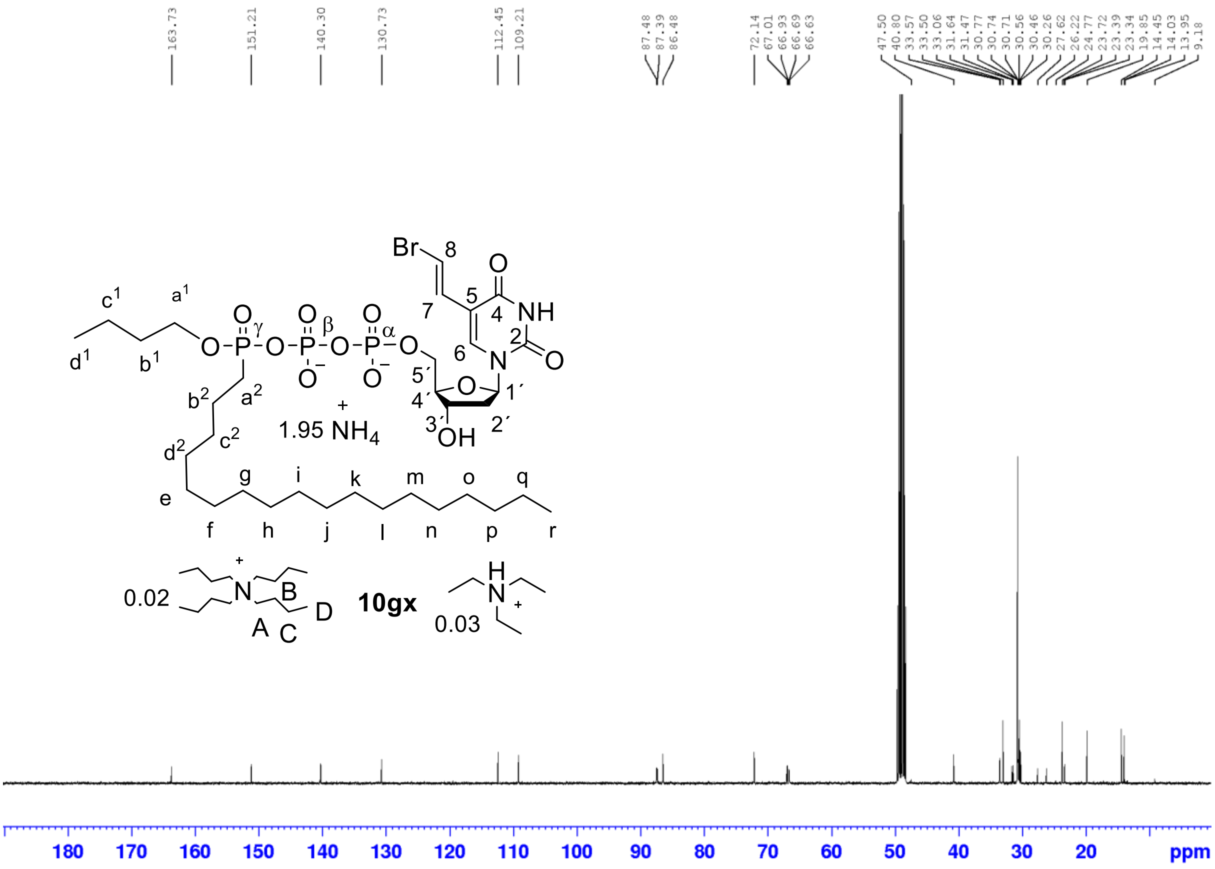
Br
1.95 NH4
0.02
10gx
0.03
ppm

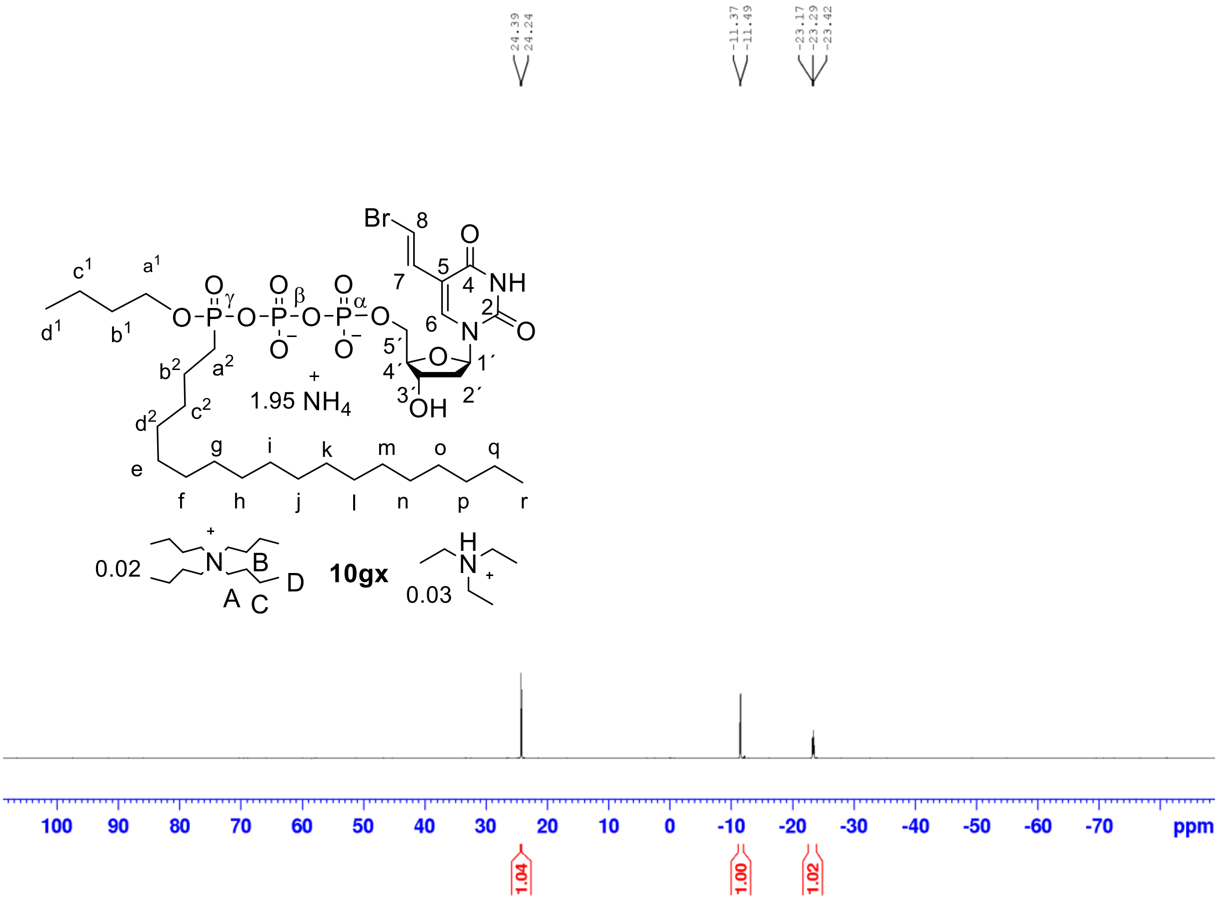
Br
1.95 NH4
0.02
10gx
0.03
ppm
1.04
1.00
1.02

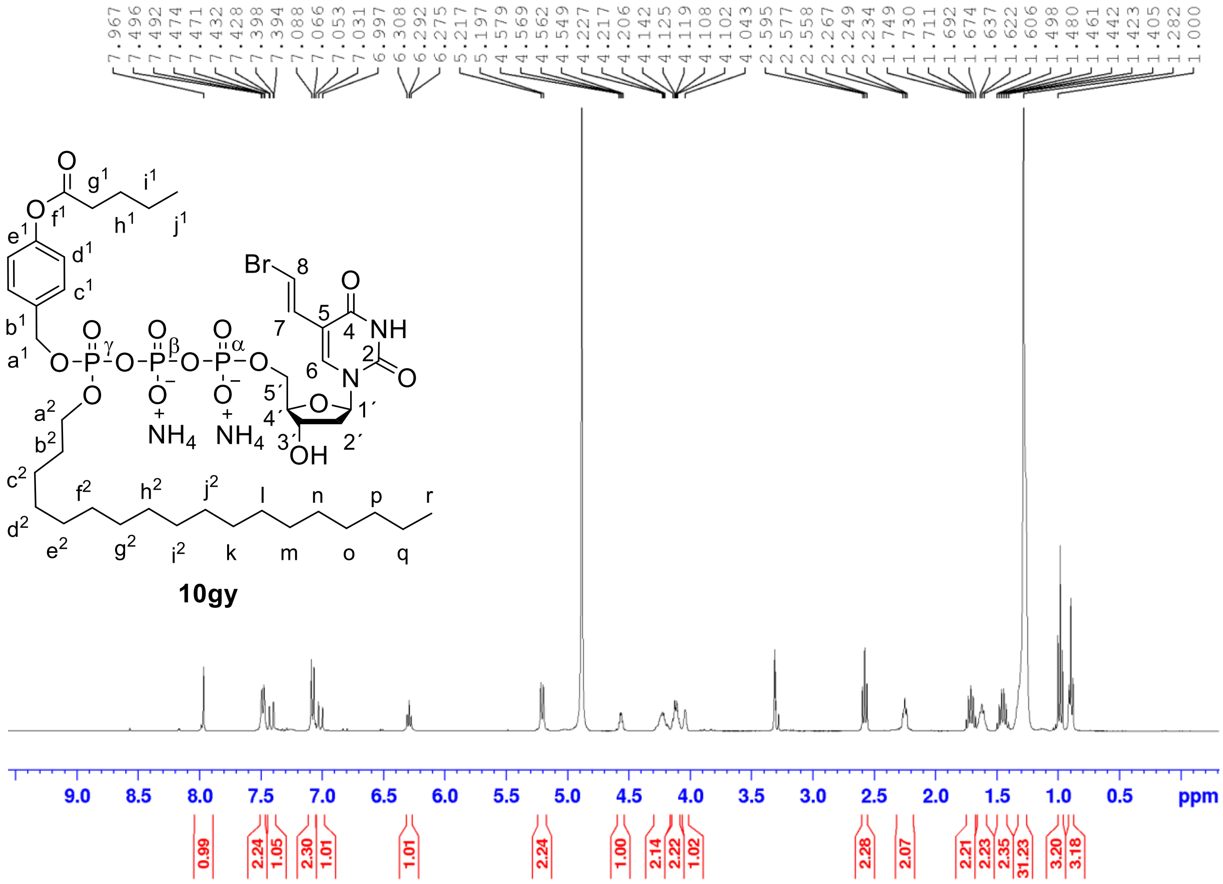
10gy
ppm
0.99
2.24
1.05
2.30
1.01
1.01
2.24
1.00
2.14
2.22
1.02
2.28
2.07
2.21
2.23
2.35
31.23
3.20
3.18

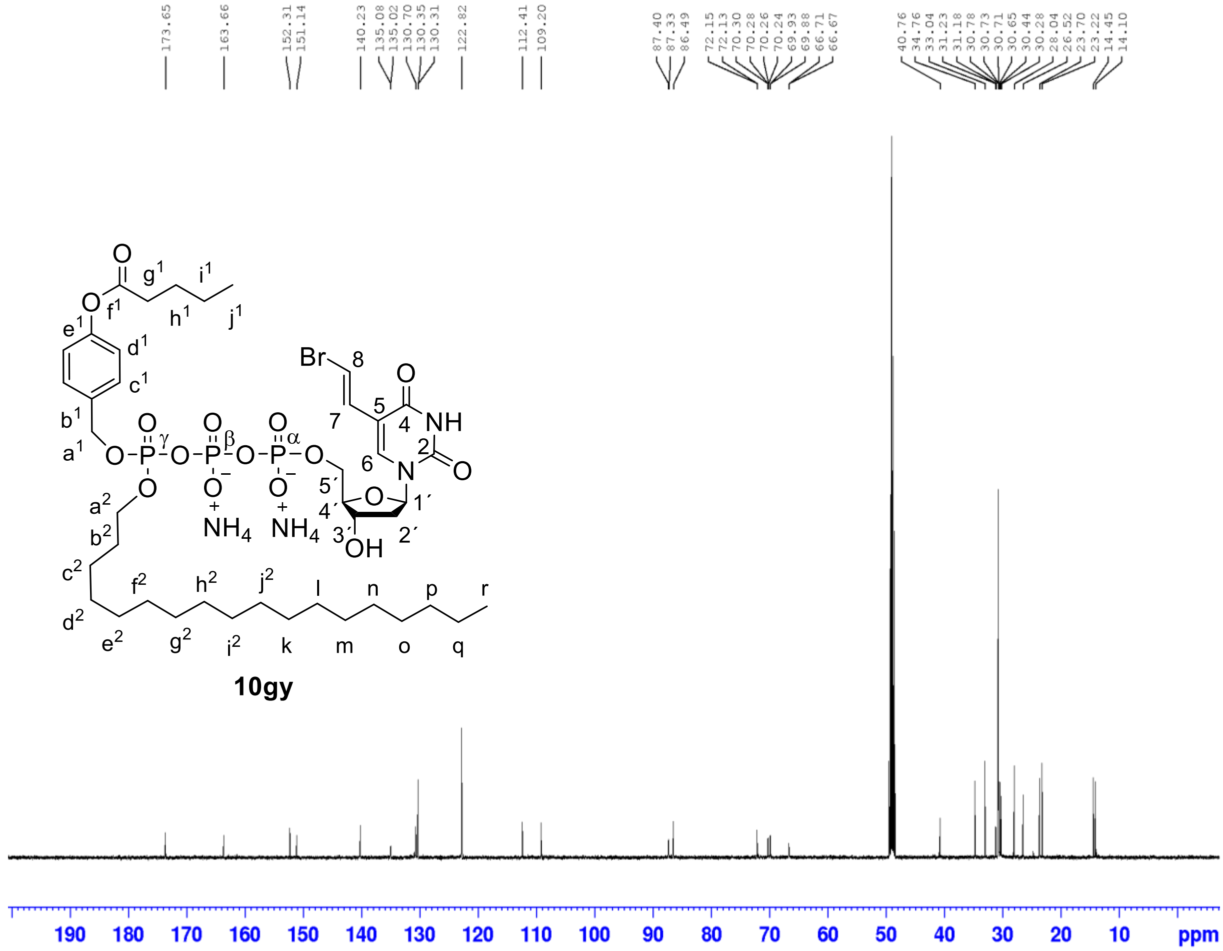
10gy
ppm

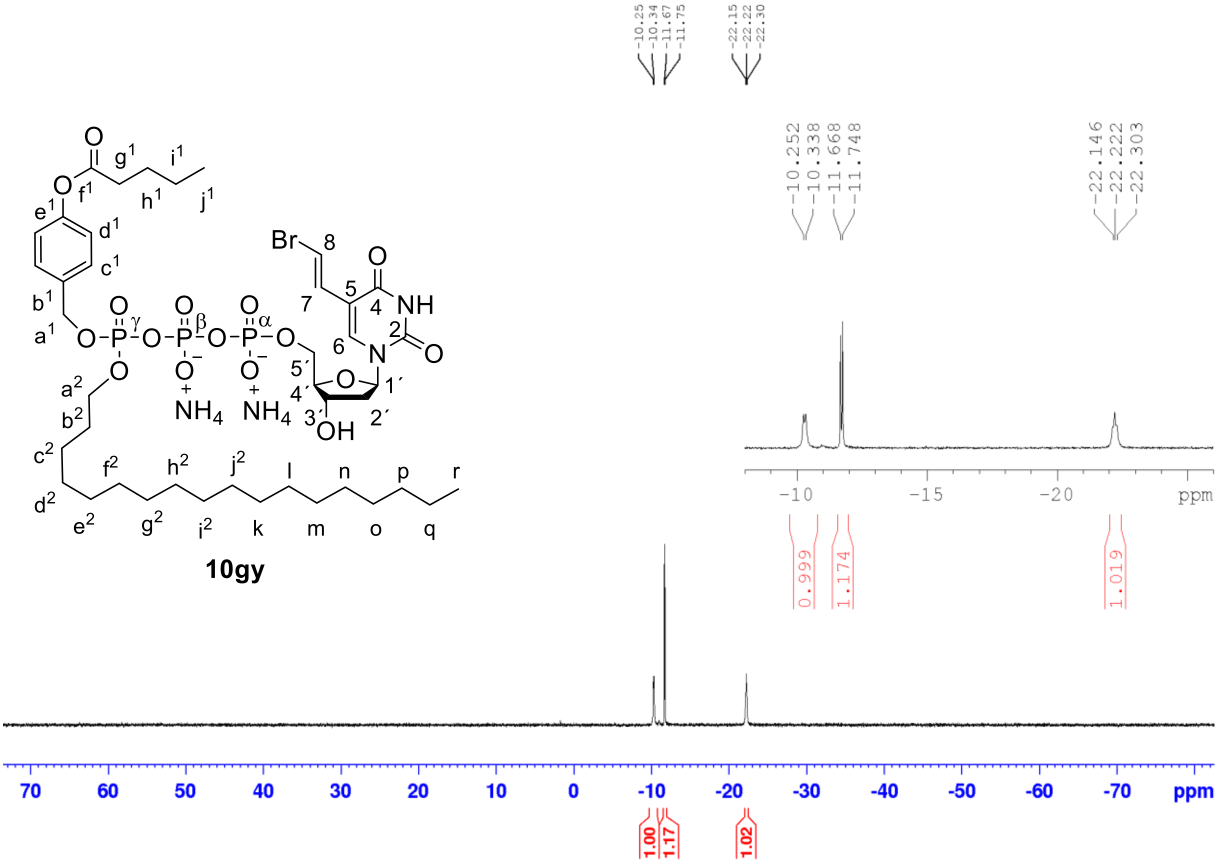
10gy
ppm

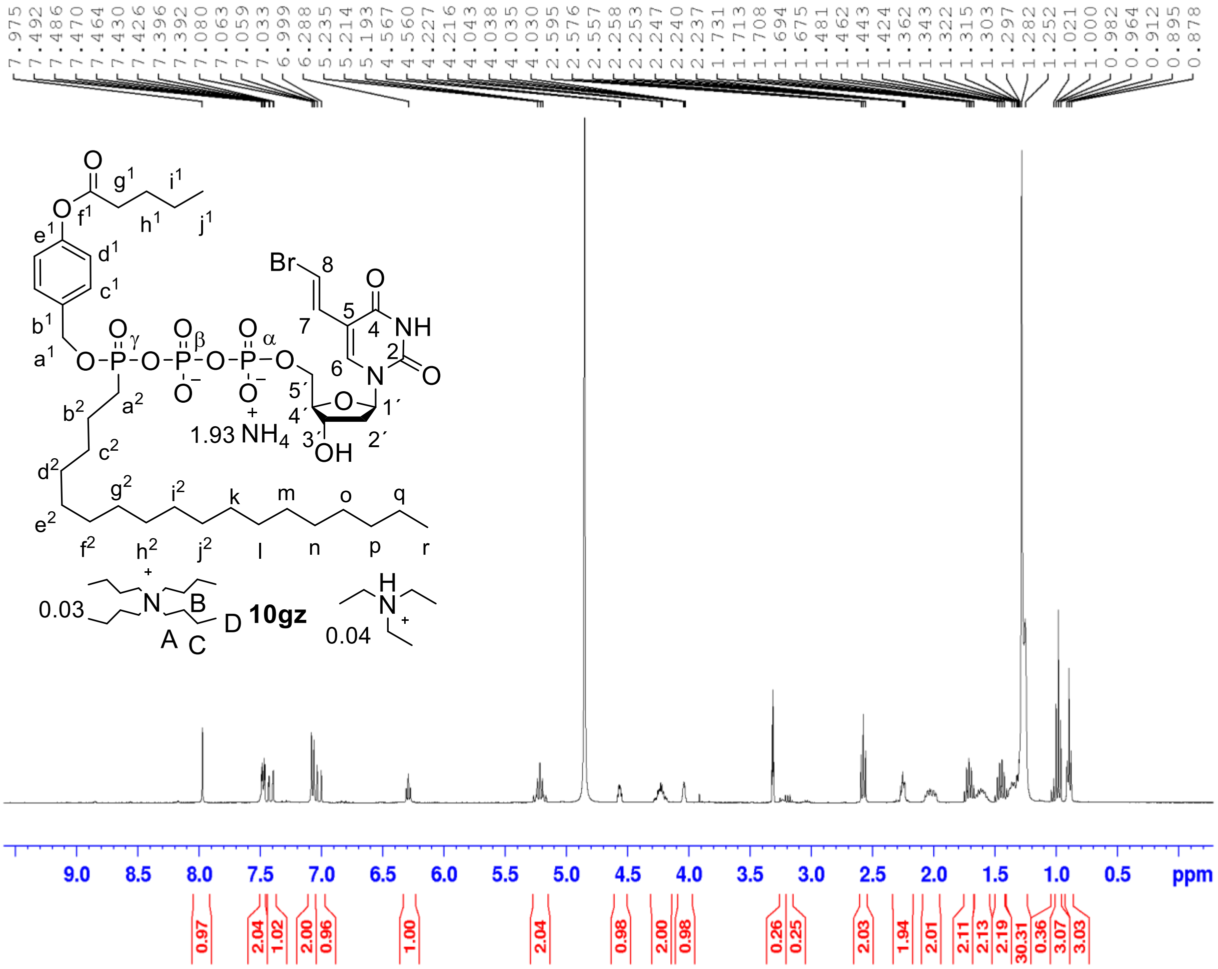
10gz
0.03
0.04
ppm

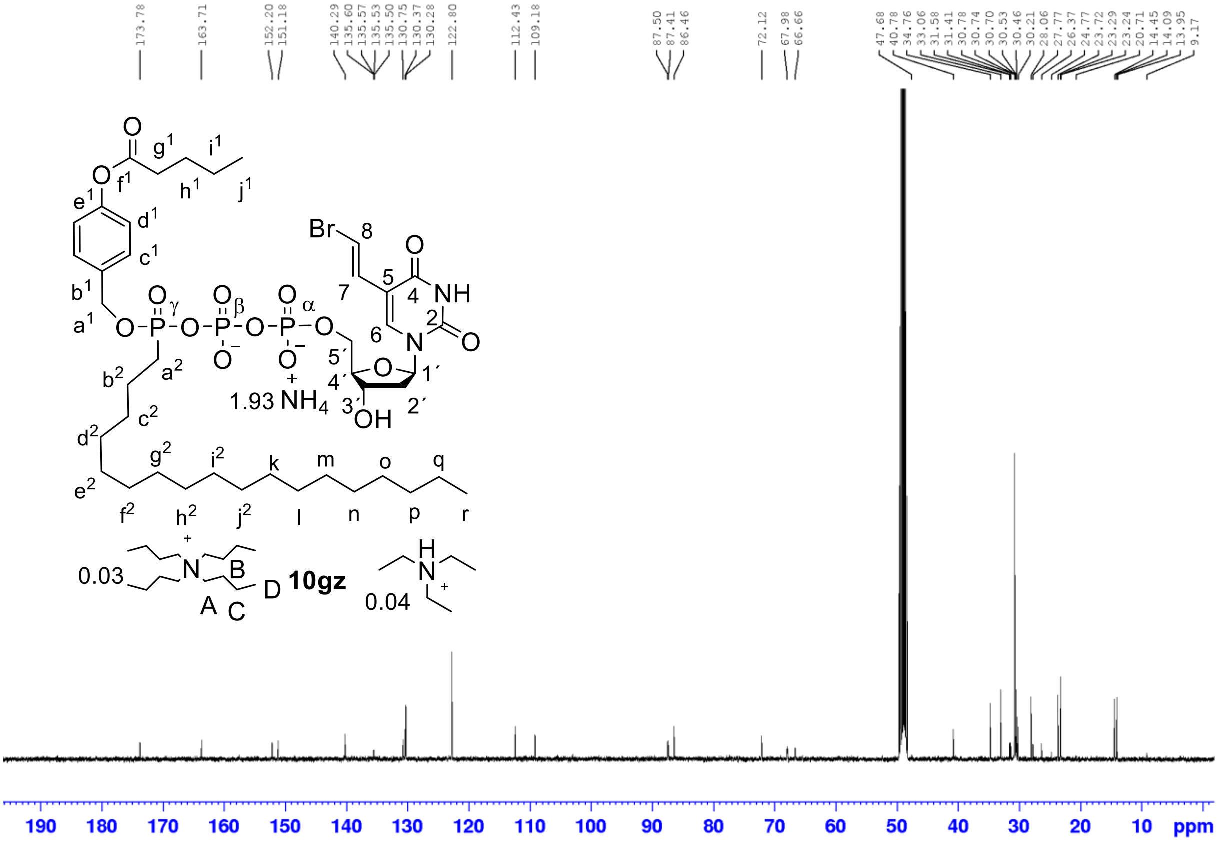
173.78
163.71
152.20
151.18
140.29
135.60
135.57
135.53
135.50
130.75
130.37
130.28
122.80
112.43
109.18
87.50
87.41
86.46
72.12
67.98
66.66
47.68
40.78
34.76
33.06
31.58
31.41
30.78
30.74
30.70
30.53
30.46
30.21
28.06
27.77
26.37
24.77
23.72
23.29
23.24
20.71
14.45
14.09
13.95
9.17
1.93 NH4
0.03
0.04
10gz
190 180 170 160 150 140 130 120 110 100 90 80 70 60 50 40 30 20 10 ppm

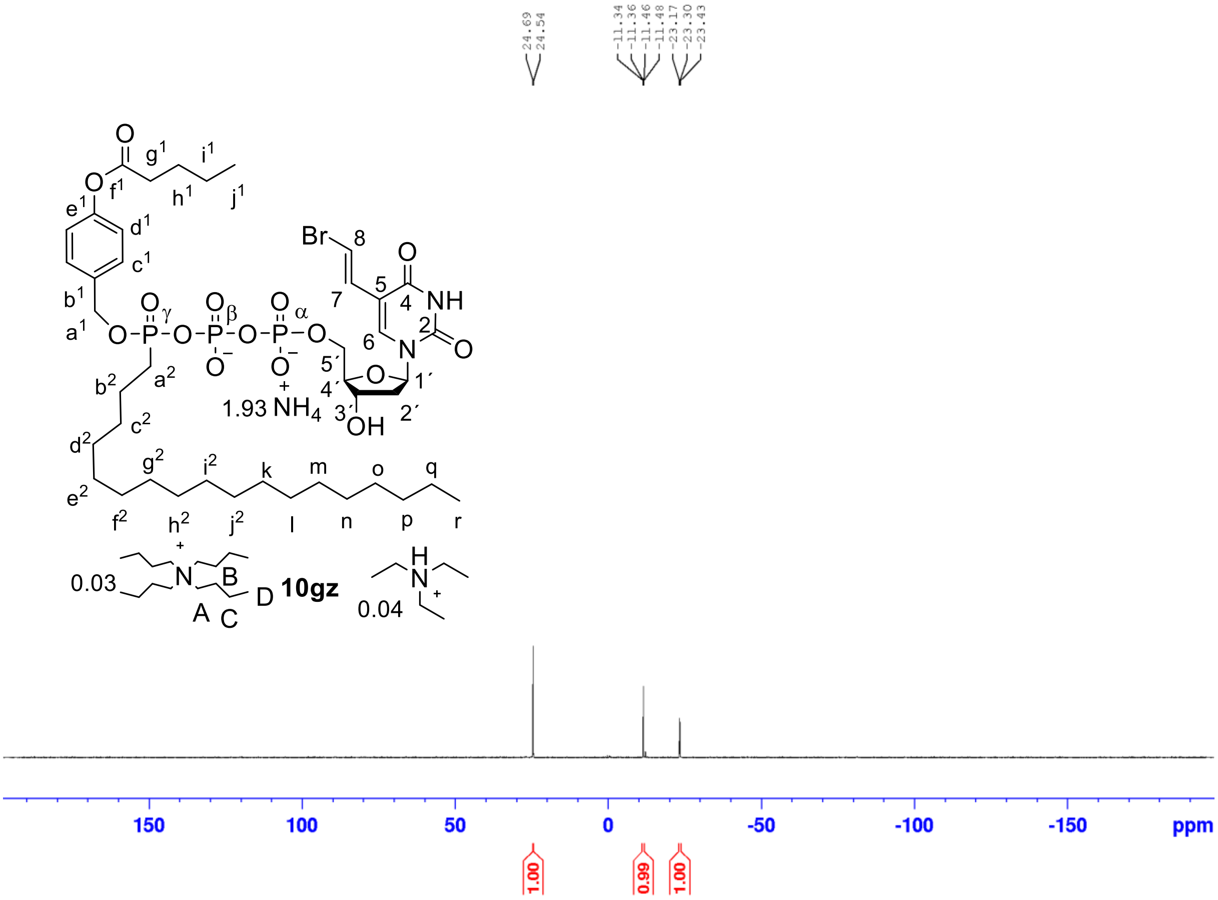
24.69
24.54
-11.34
-11.36
-11.46
-11.48
-23.17
-23.30
-23.43
1.93 NH4
0.03
0.04
10gz
150 100 50 0 -50 -100 -150 ppm
1.00
0.99
1.00

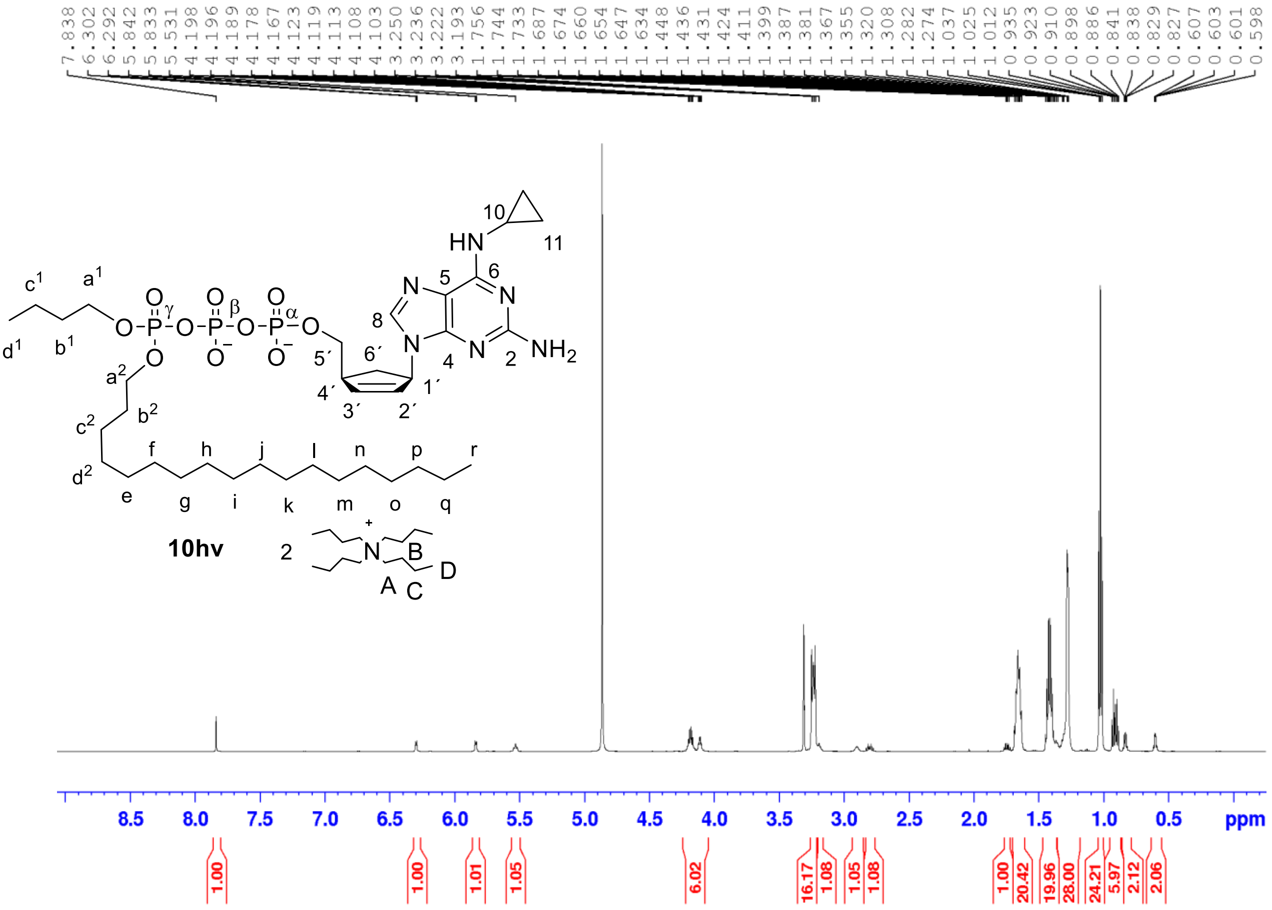
7.838 6.302 6.292 5.842 5.833 5.531 4.198 4.196 4.189 4.178 4.167 4.123 4.119 4.113 4.108 4.103 3.250 3.236 3.222 3.193 1.756 1.744 1.733 1.687 1.674 1.660 1.654 1.647 1.634 1.448 1.436 1.431 1.424 1.411 1.399 1.387 1.381 1.367 1.355 1.320 1.308 1.282 1.274 1.037 1.025 1.012 0.935 0.923 0.910 0.898 0.886 0.841 0.838 0.829 0.827 0.607 0.603 0.601 0.598
10hv
ppm
1.00 1.00 1.01 1.05 6.02 16.17 1.08 1.05 1.08 1.00 20.42 19.96 28.00 24.21 5.97 2.12 2.06

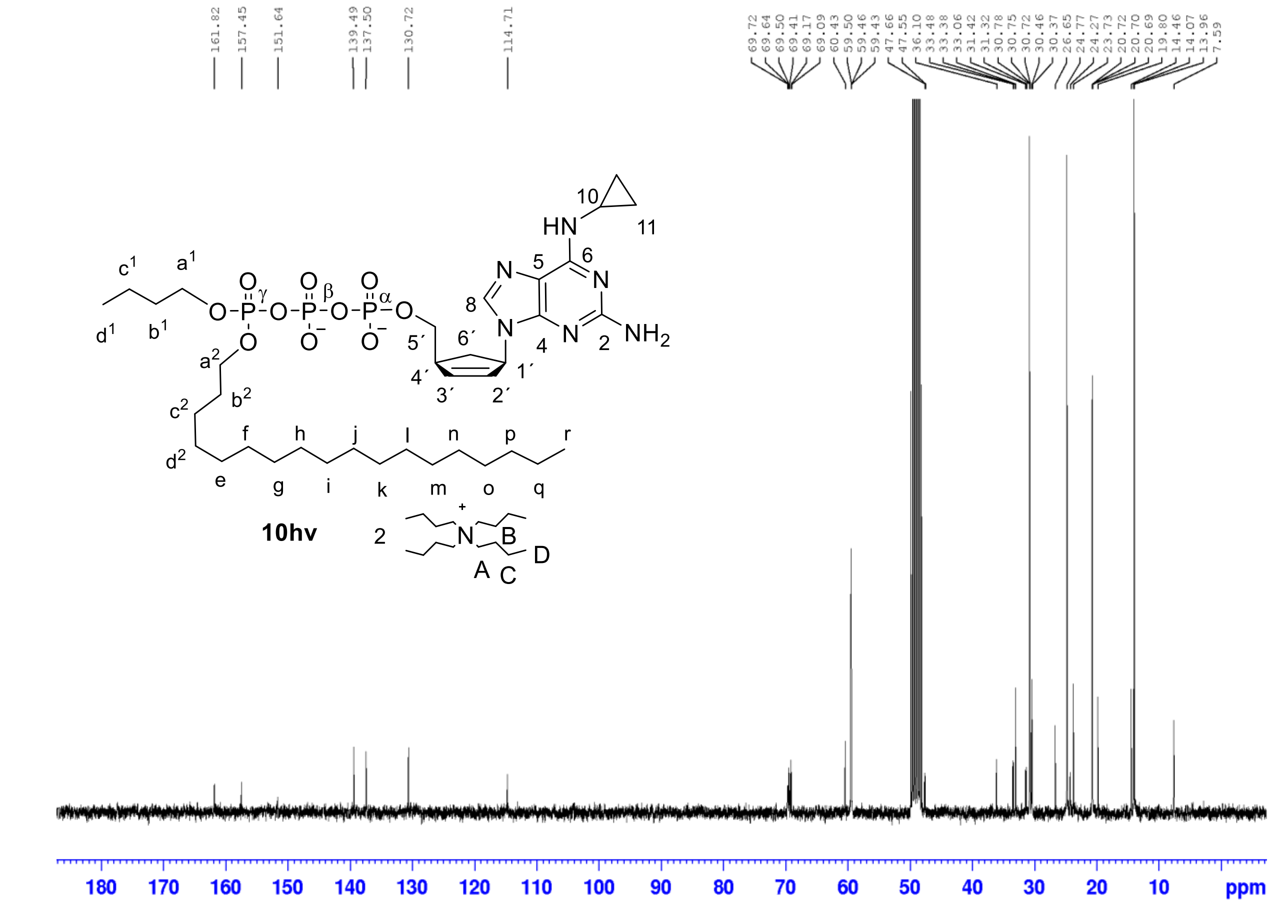
161.82 157.45 151.64 139.49 137.50 130.72 114.71
69.72 69.64 69.50 69.41 69.17 69.09 60.43 59.50 59.46 59.43 47.66 47.55 36.10 33.48 33.38 33.06 31.42 31.32 30.78 30.75 30.72 30.46 30.37 26.65 24.77 24.27 23.73 20.72 20.70 20.69 19.80 14.46 14.07 13.96 7.59
10hv
ppm

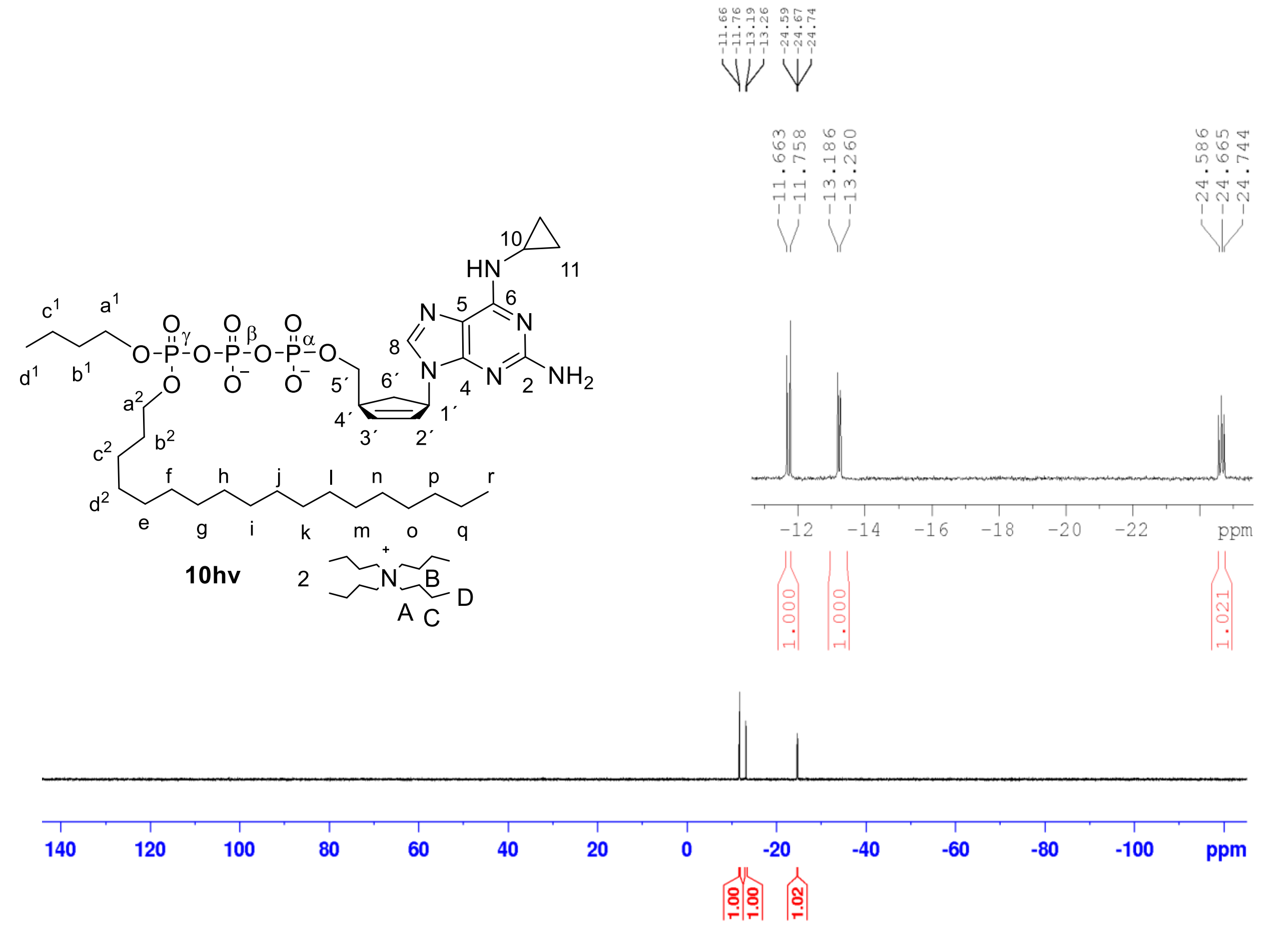
10hv
2
ppm
1.000
1.000
1.021
1.00
1.00
1.02

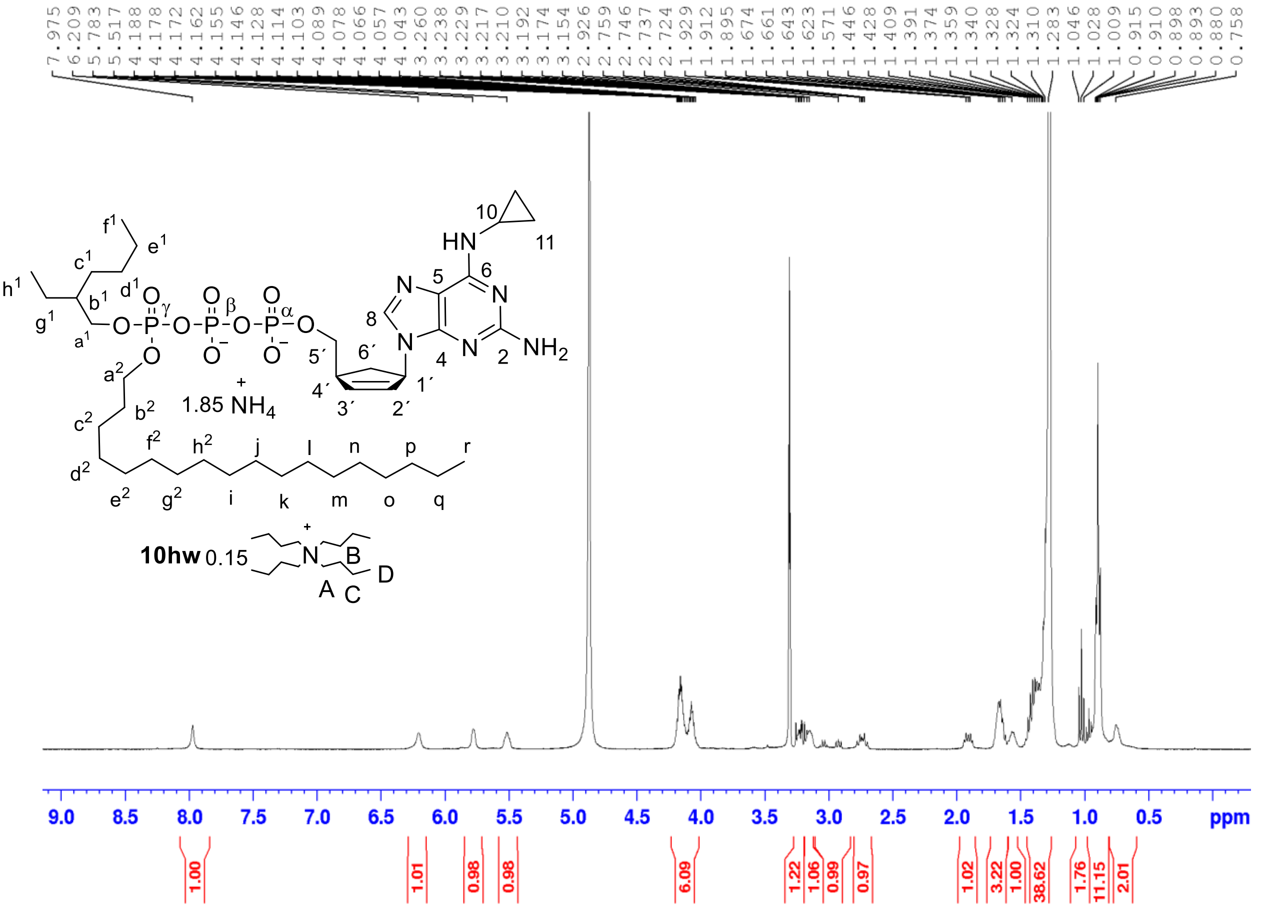
10hw
0.15
1.85 NH4
ppm
1.00
1.01
0.98
0.98
6.09
1.22
1.06
0.99
0.97
1.02
3.22
1.00
38.62
1.76
11.15
2.01

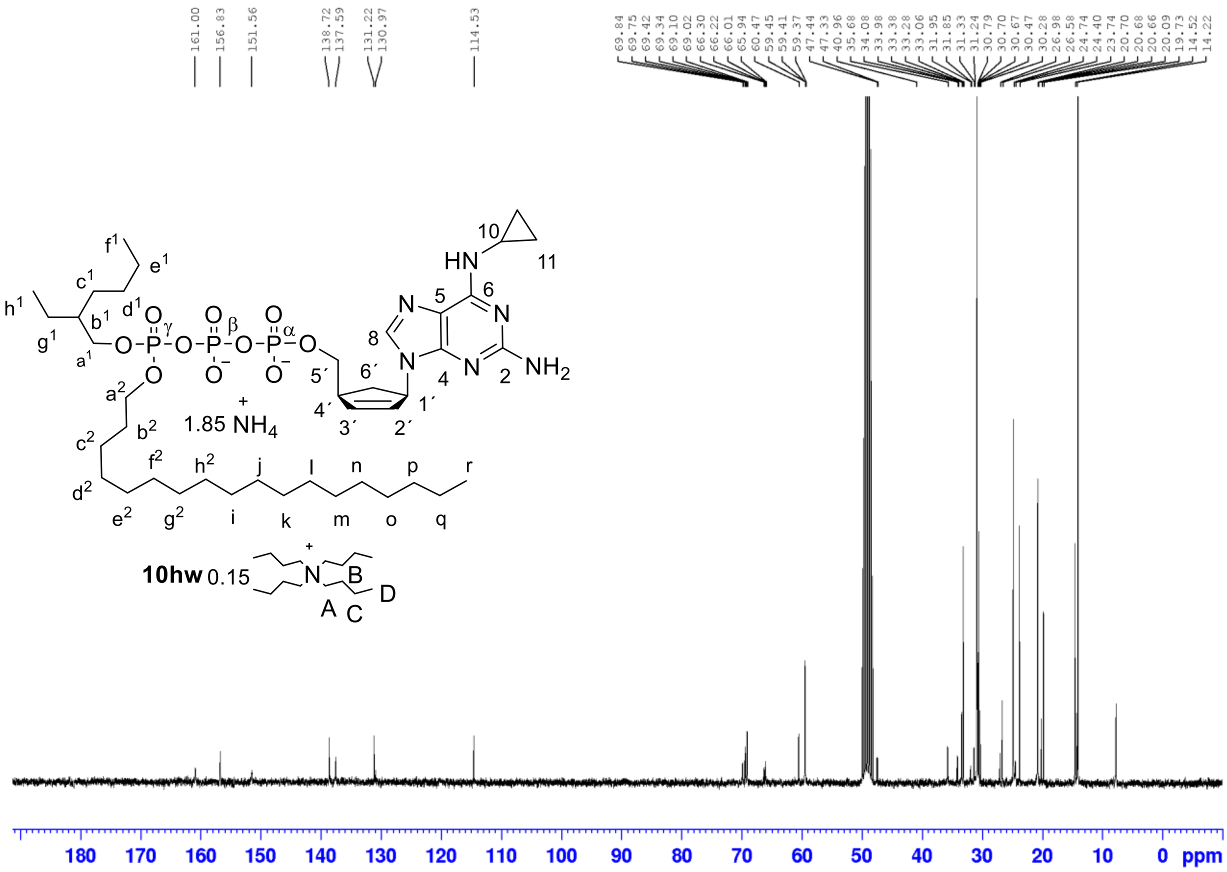
161.00 156.83 151.56 138.72 137.59 131.22 130.97 114.53
69.84 69.75 69.42 69.34 69.10 69.02 66.30 66.22 66.01 65.94 60.47 59.45 59.41 59.37 47.44 47.33 40.96 35.68 34.08 33.98 33.38 33.28 33.06 31.95 31.85 31.33 31.24 30.79 30.70 30.67 30.47 30.28 26.98 26.58 24.74 24.40 23.74 20.70 20.68 20.66 20.09 19.73 14.52 14.22
1.85 NH4
10hw 0.15
ppm

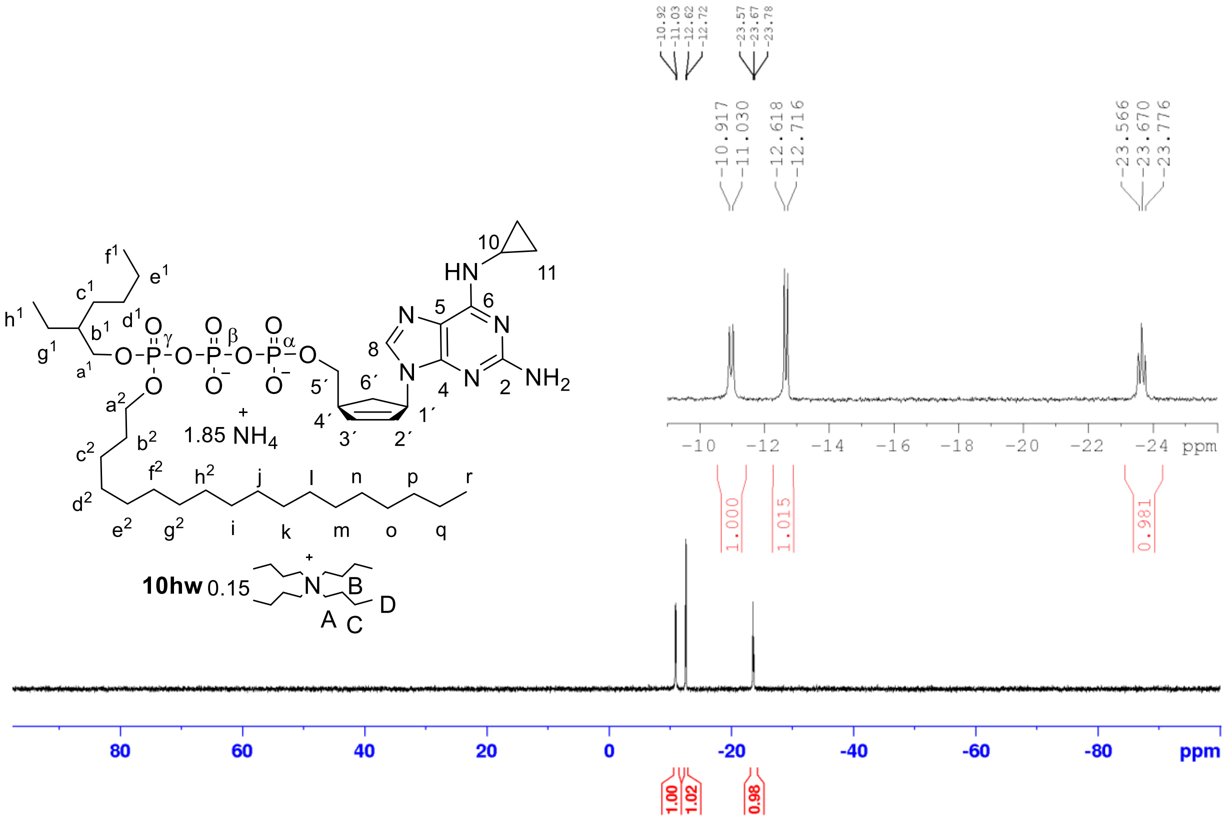
-10.92 -11.03 -12.62 -12.72 -23.57 -23.67 -23.78
-10.917 -11.030 -12.618 -12.716 -23.566 -23.670 -23.776
1.000 1.015 0.981
1.85 NH4
10hw 0.15
1.00 1.02 0.98
ppm

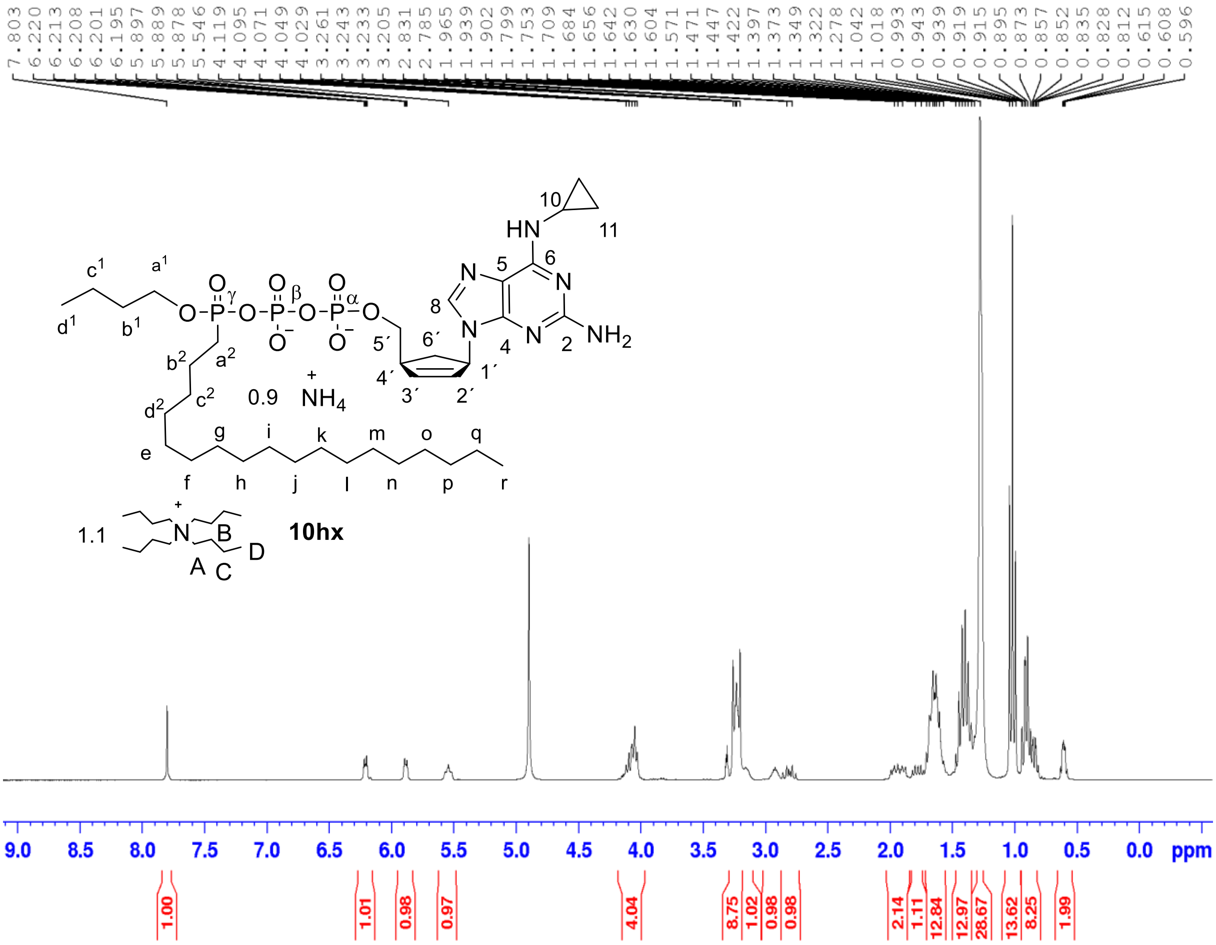

7.803
6.220
6.213
6.208
6.201
6.195
5.897
5.889
5.878
5.546
4.119
4.095
4.071
4.049
4.029
3.261
3.243
3.233
3.205
2.831
2.785
1.965
1.939
1.902
1.799
1.753
1.709
1.684
1.656
1.642
1.630
1.604
1.571
1.471
1.447
1.422
1.397
1.373
1.349
1.322
1.278
1.042
1.018
0.993
0.943
0.939
0.919
0.915
0.895
0.873
0.857
0.852
0.835
0.828
0.812
0.615
0.608
0.596
10hx
0.9 NH4
1.1
9.0 8.5 8.0 7.5 7.0 6.5 6.0 5.5 5.0 4.5 4.0 3.5 3.0 2.5 2.0 1.5 1.0 0.5 0.0 ppm
1.00
1.01
0.98
0.97
4.04
8.75
1.02
0.98
0.98
2.14
1.11
12.84
12.97
28.67
13.62
8.25
1.99

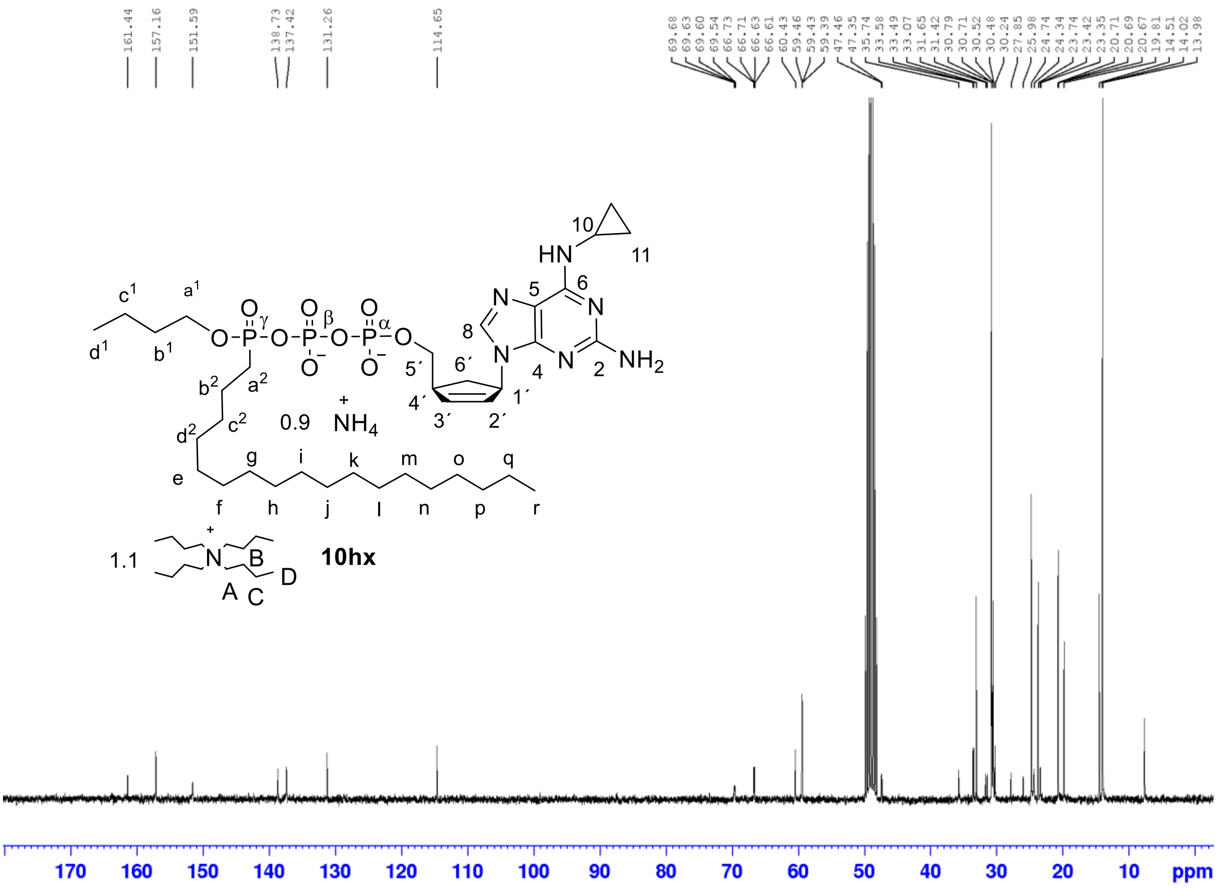

161.44
157.16
151.59
138.73
137.42
131.26
114.65
69.68
69.63
69.60
69.54
66.73
66.71
66.63
66.61
60.43
59.46
59.43
59.39
47.46
47.35
35.74
33.58
33.49
33.07
31.65
31.42
30.79
30.71
30.52
30.48
30.24
27.85
25.98
24.74
24.34
23.74
23.42
23.35
20.71
20.69
20.67
19.81
14.51
14.02
13.98
10hx
0.9 NH4
1.1
170 160 150 140 130 120 110 100 90 80 70 60 50 40 30 20 10 ppm

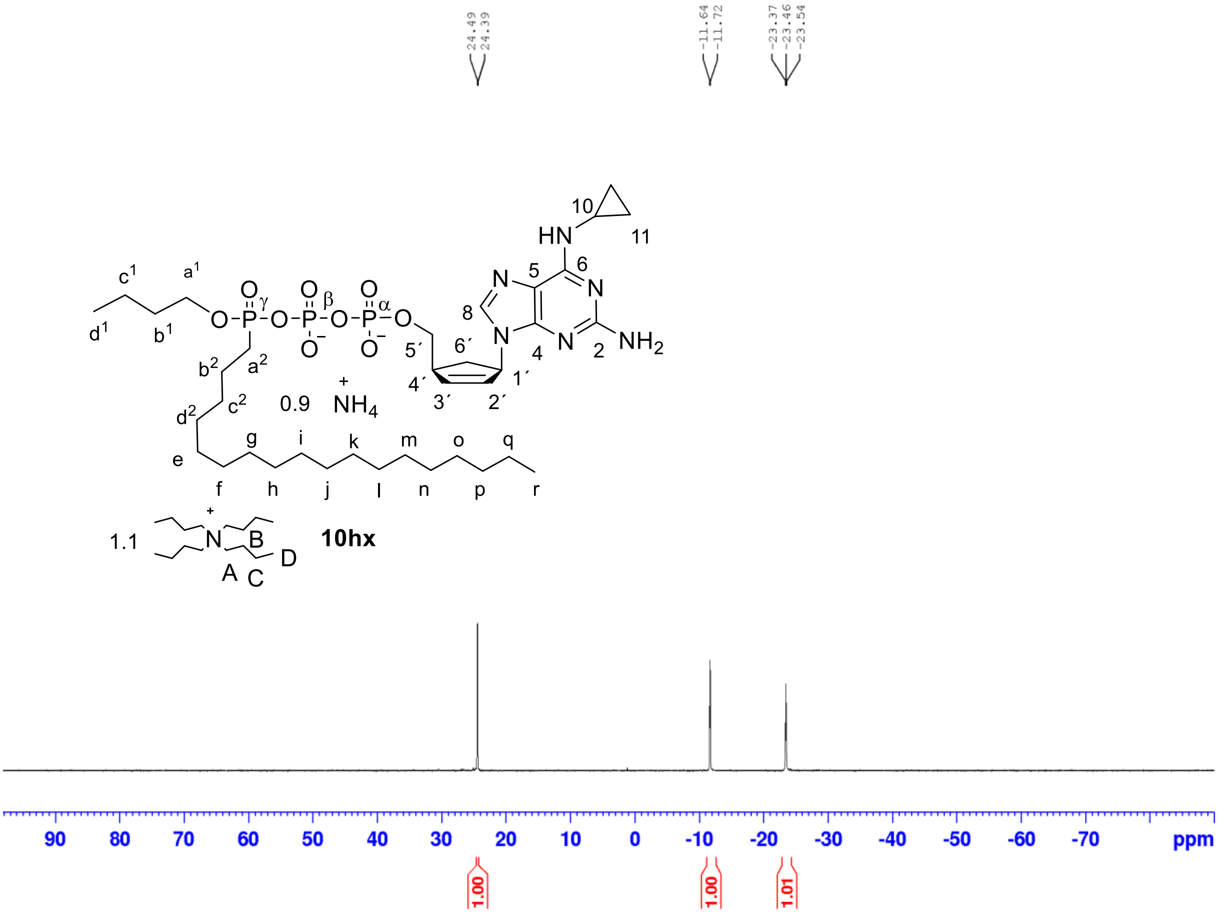
10hx
24.49
24.39
-11.64
-11.72
-23.37
-23.46
-23.54
ppm

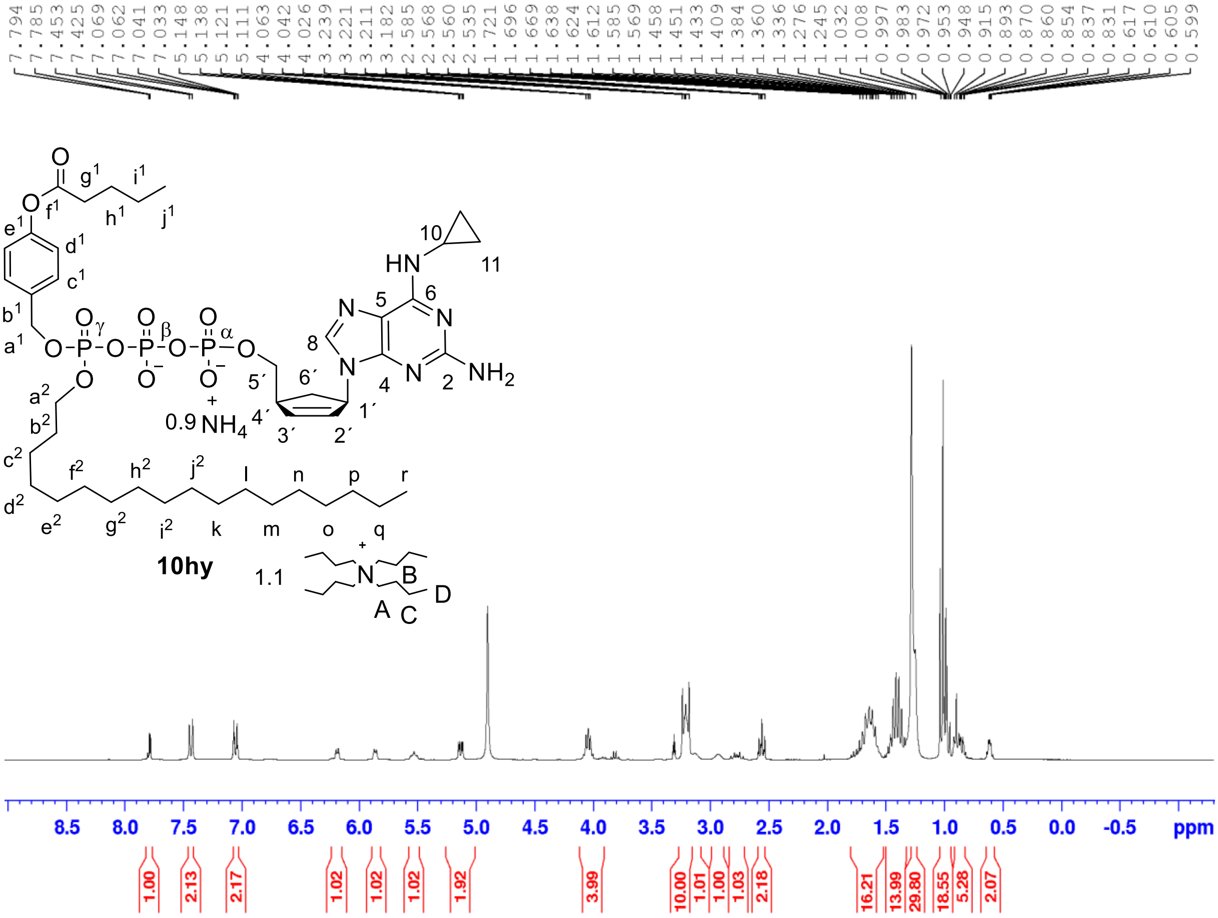
10hy
ppm

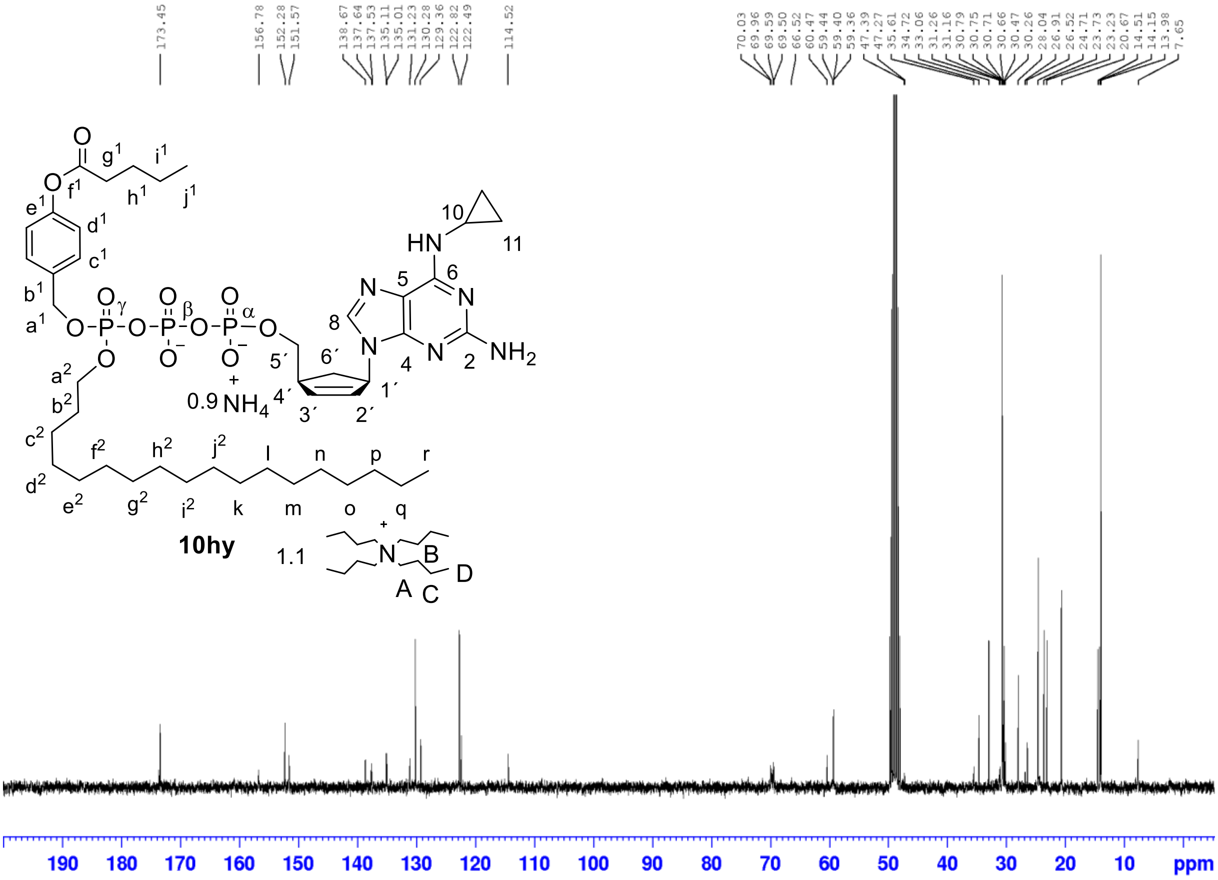
173.45
156.78
152.28
151.57
138.67
137.64
137.53
135.11
135.01
131.23
130.28
129.36
122.82
122.49
114.52
70.03
69.96
69.59
69.50
66.52
60.47
59.44
59.40
59.36
47.39
47.27
35.61
34.72
33.06
31.26
31.16
30.79
30.75
30.71
30.66
30.47
30.26
28.04
26.91
26.52
24.71
23.73
23.23
20.67
14.51
14.15
13.98
7.65
10hy
ppm

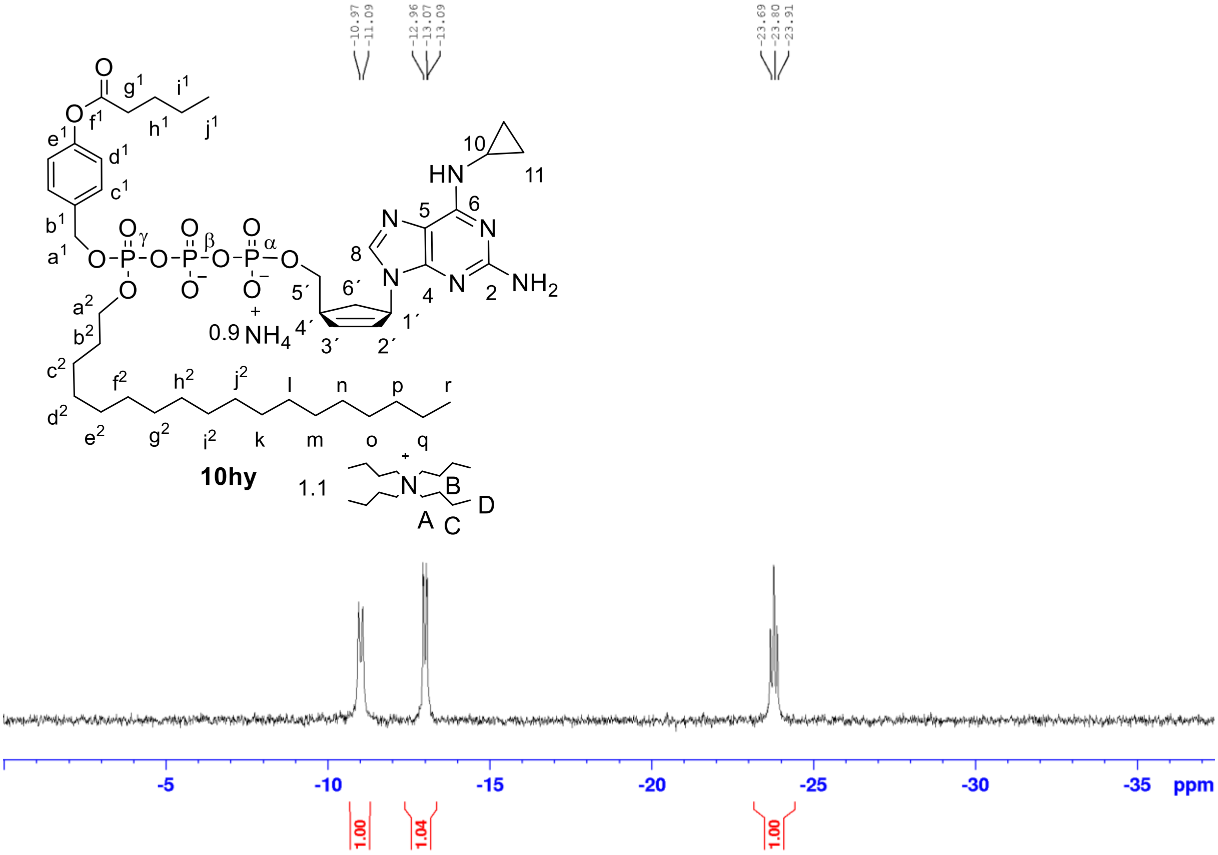
-10.97
-11.09
-12.96
-13.07
-13.09
-23.69
-23.80
-23.91
10hy
ppm
1.00
1.04
1.00

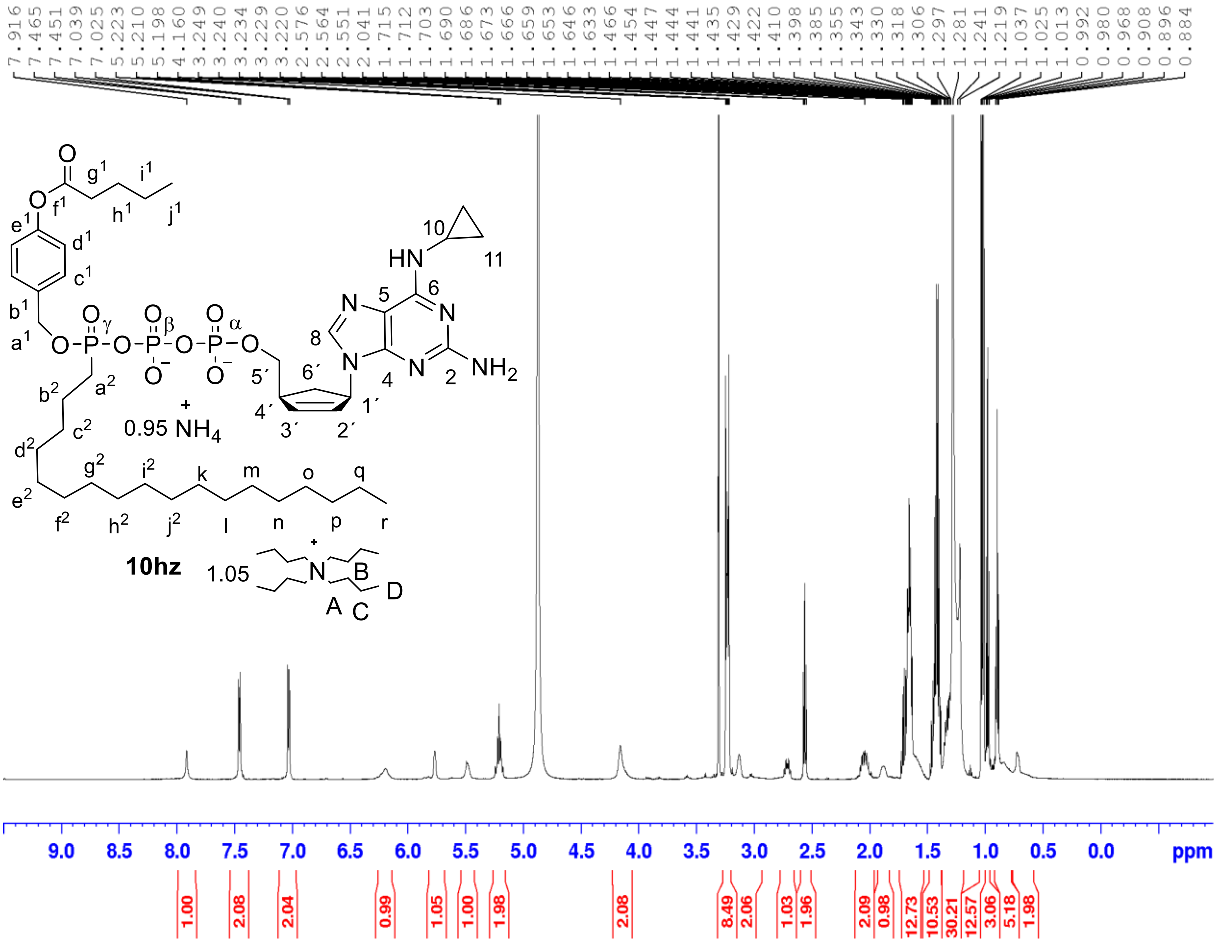

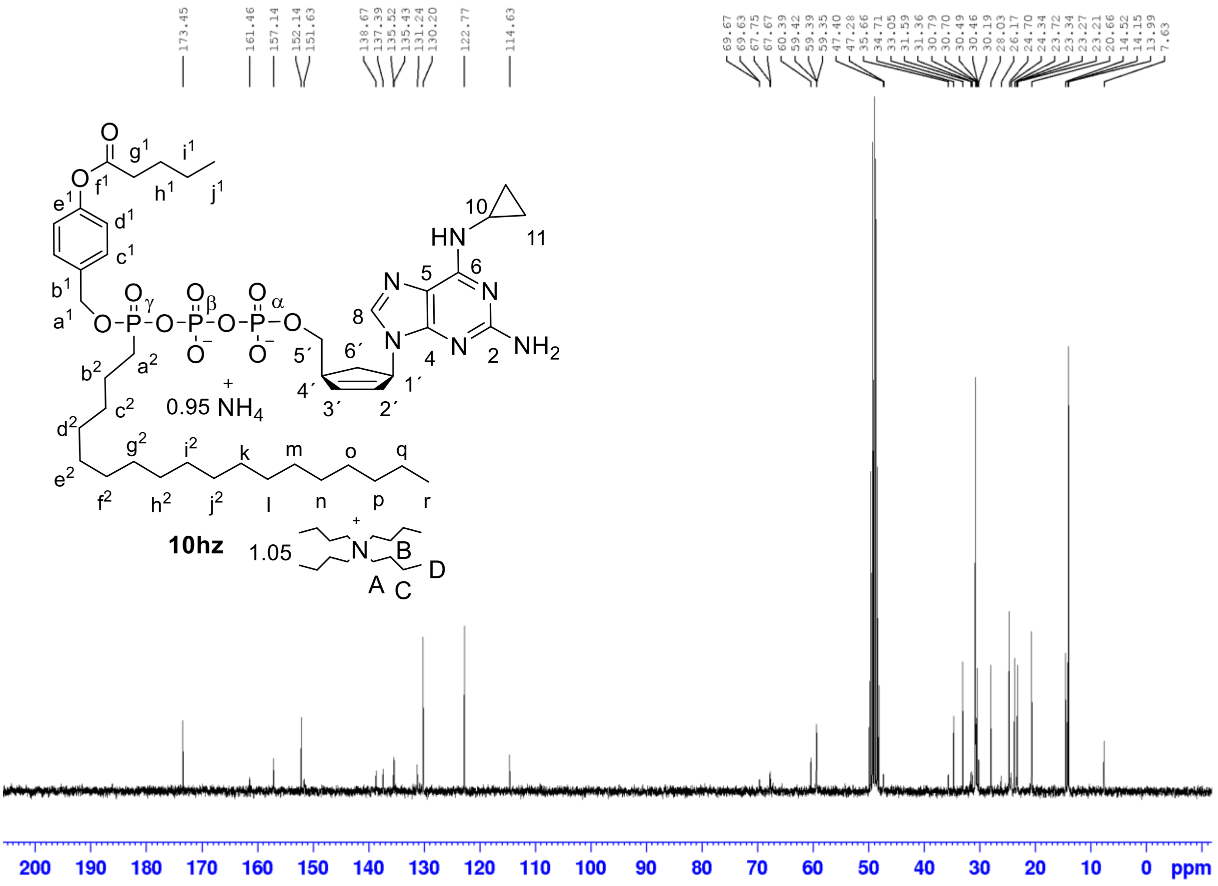

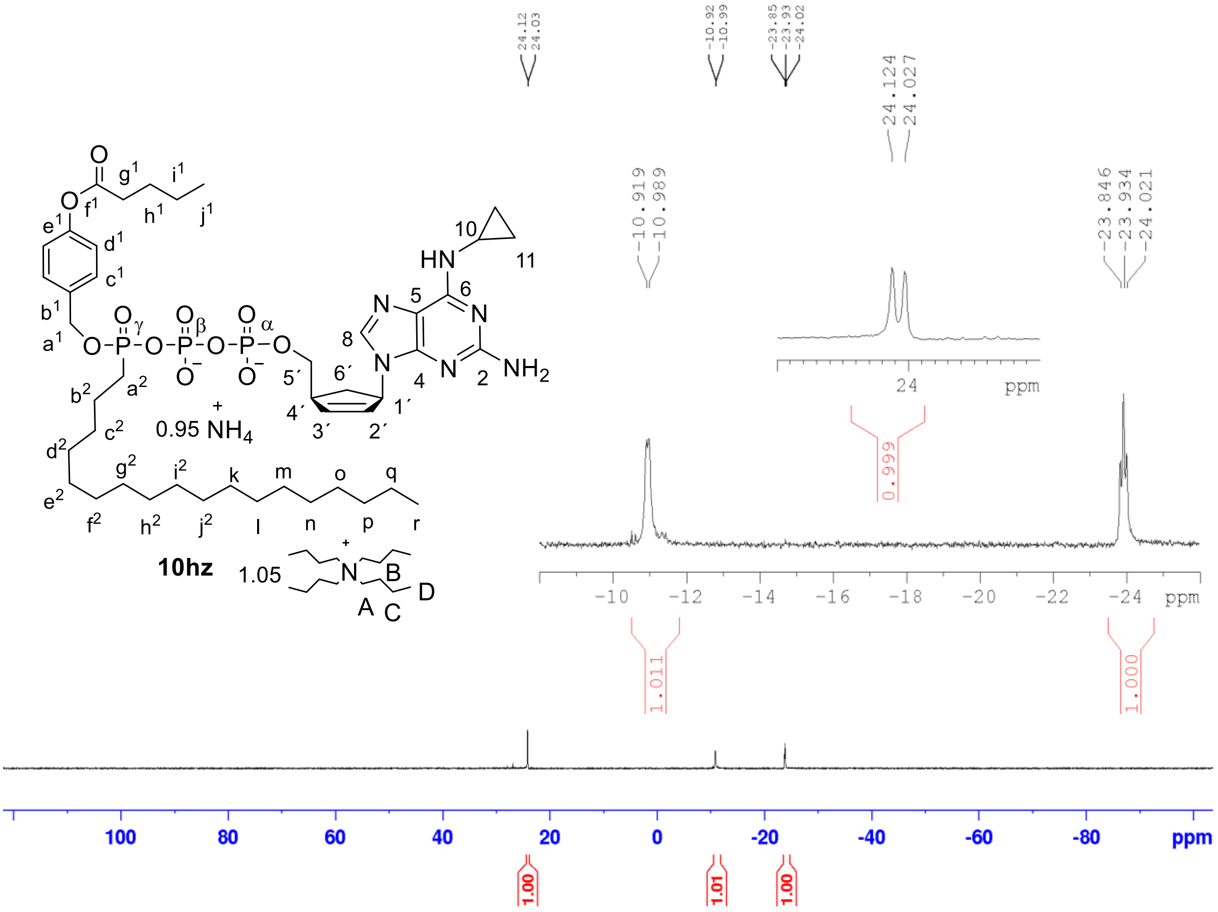
10hz
0.95 NH_4
1.05
ppm

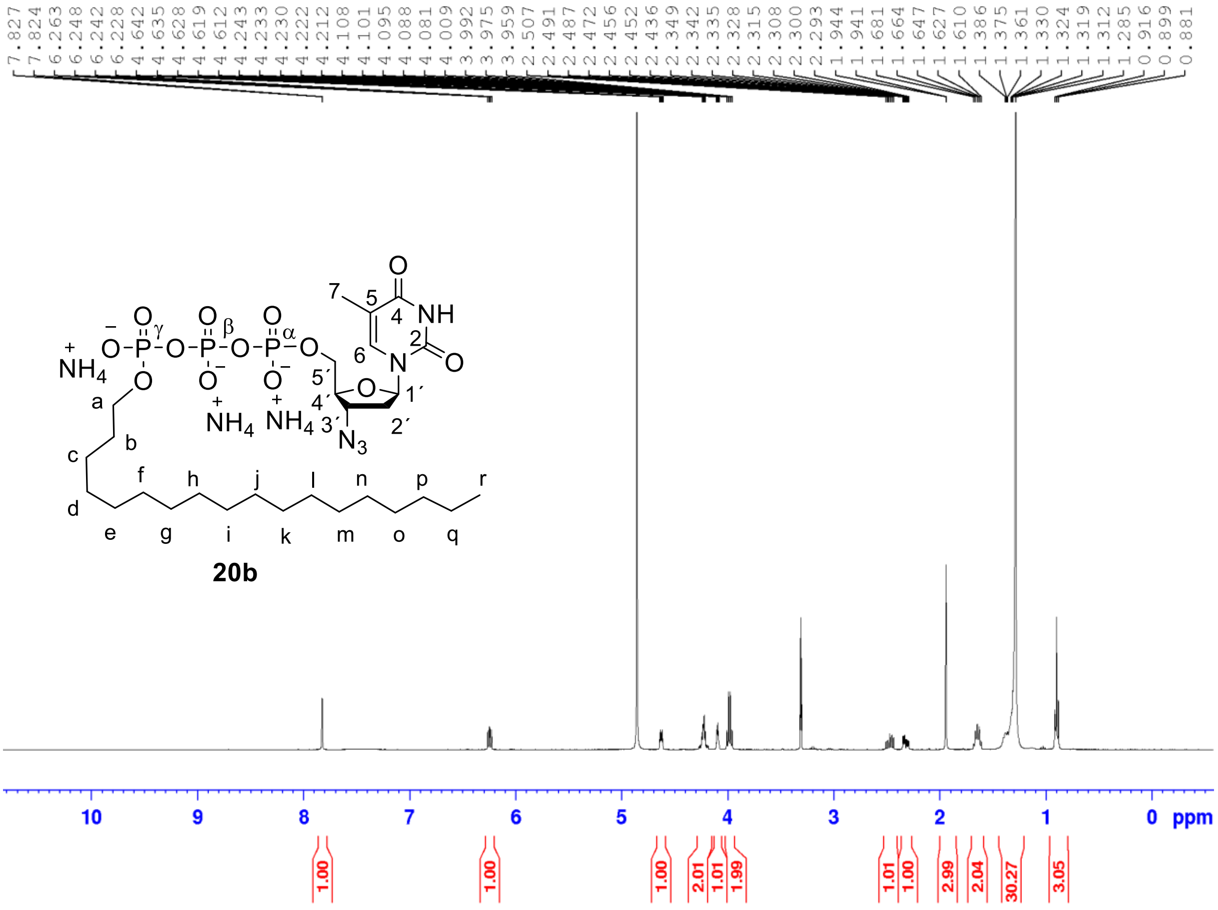
20b
ppm

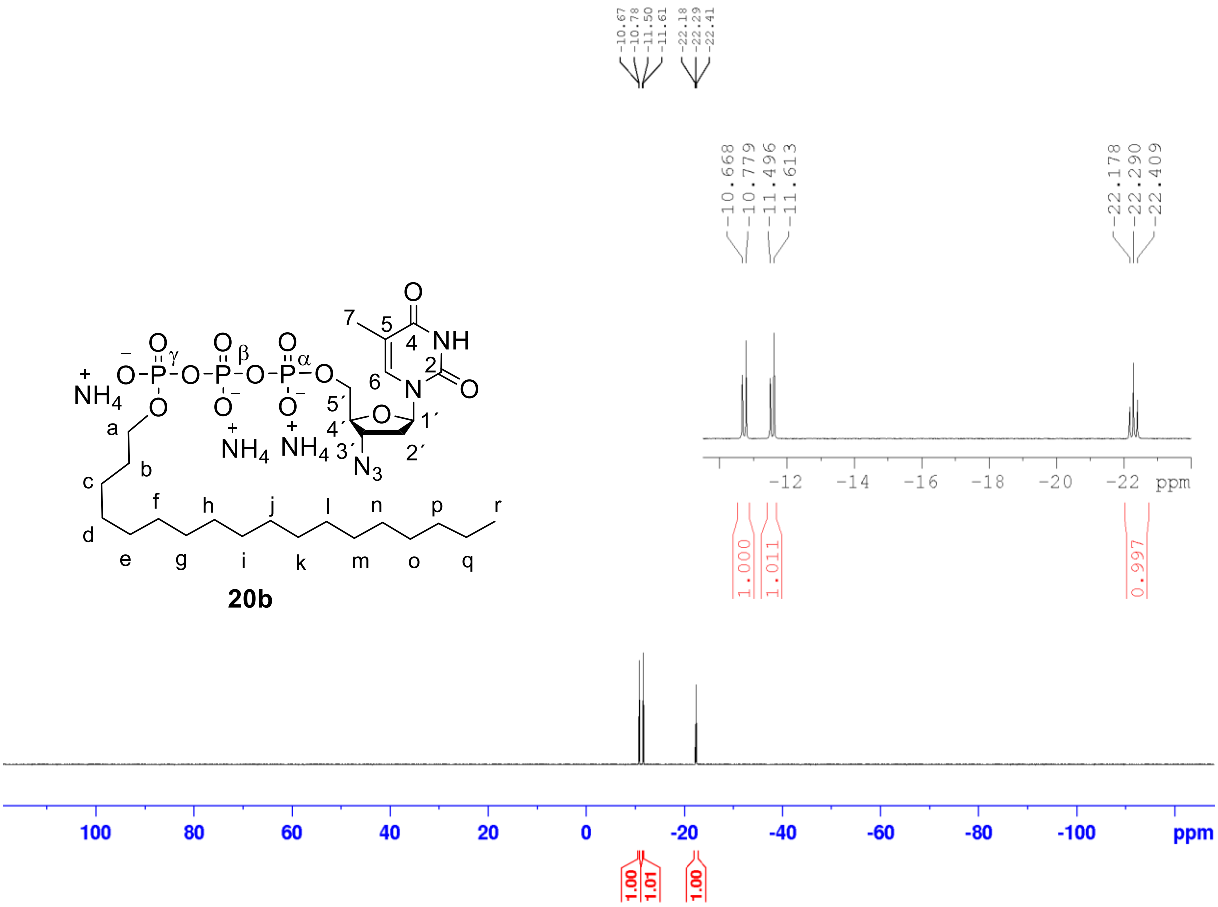
-10.67
-10.78
-11.50
-11.61
-22.18
-22.29
-22.41
-10.668
-10.779
-11.496
-11.613
-22.178
-22.290
-22.409
1.000
1.011
0.997
-12 -14 -16 -18 -20 -22 ppm
100 80 60 40 20 0 -20 -40 -60 -80 -100 ppm
1.00
1.01
1.00
20b

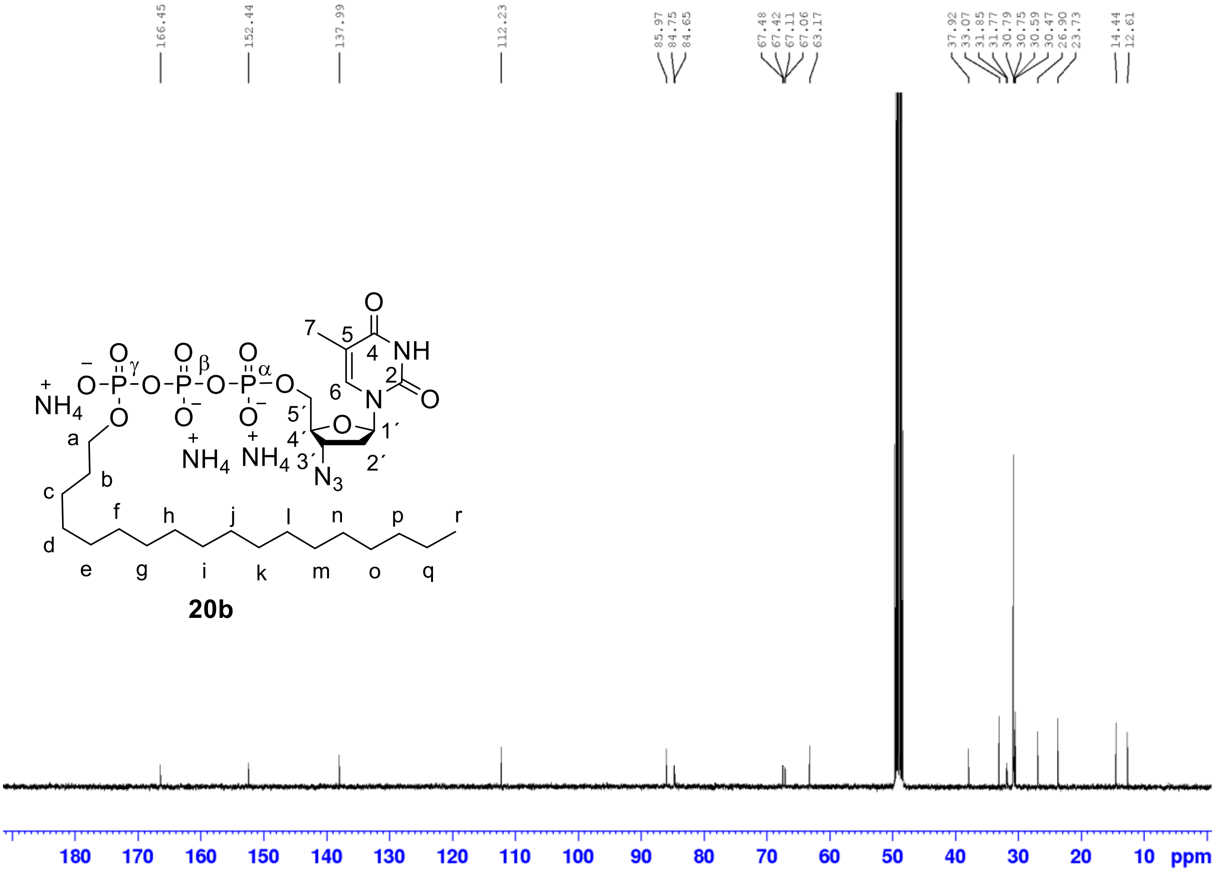
166.45
152.44
137.99
112.23
85.97
84.75
84.65
67.48
67.42
67.11
67.06
63.17
37.92
33.07
31.85
31.77
30.79
30.75
30.59
30.47
26.90
23.73
14.44
12.61
180 170 160 150 140 130 120 110 100 90 80 70 60 50 40 30 20 10 ppm
20b

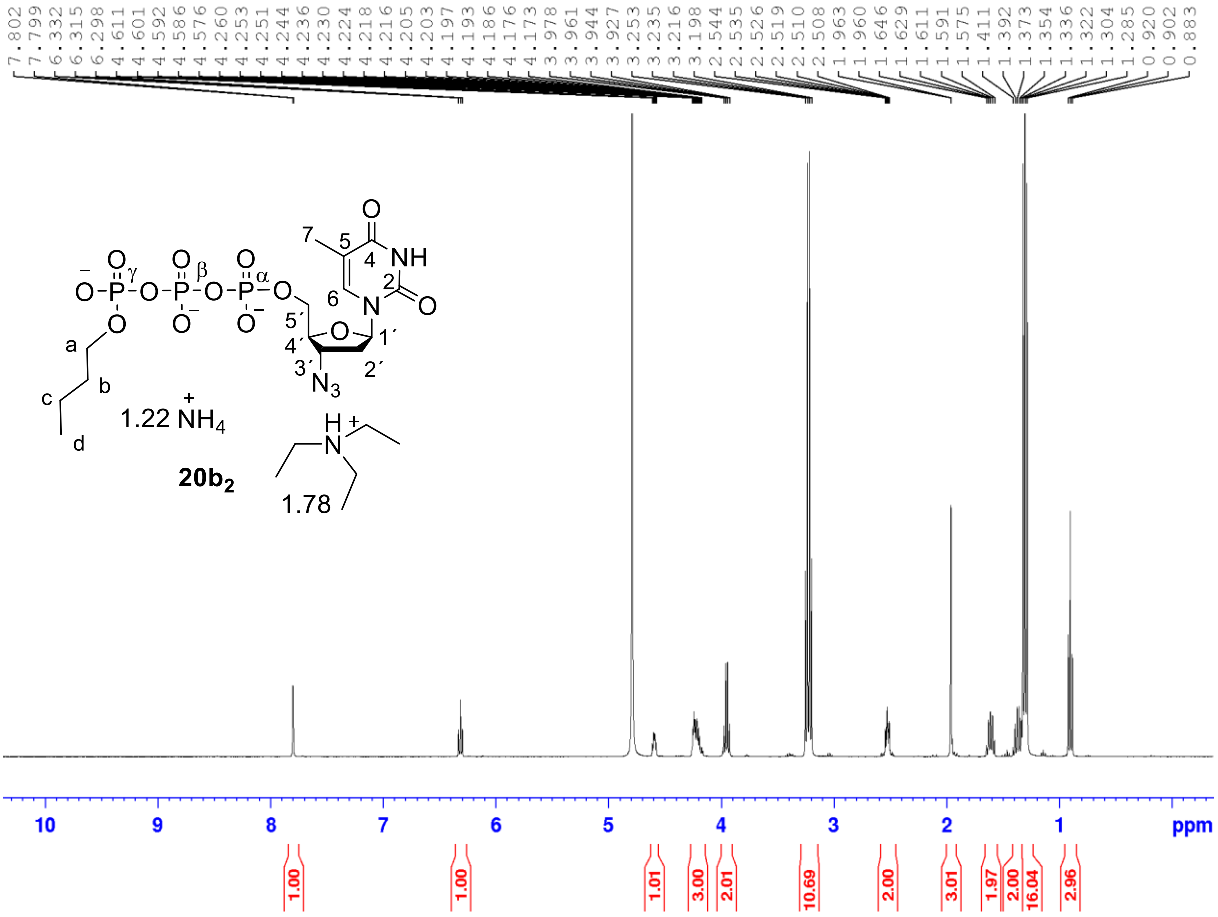
7.802 7.799 6.332 6.315 6.298 4.611 4.601 4.592 4.586 4.576 4.260 4.253 4.251 4.244 4.236 4.230 4.224 4.218 4.216 4.205 4.203 4.197 4.193 4.186 4.176 4.173 3.978 3.961 3.944 3.927 3.253 3.235 3.216 3.198 2.544 2.535 2.526 2.519 2.510 2.508 1.963 1.960 1.646 1.629 1.611 1.591 1.575 1.411 1.392 1.373 1.354 1.336 1.322 1.304 1.285 0.920 0.902 0.883
1.22 NH4
1.78
20b2
ppm
1.00 1.00 1.01 3.00 2.01 10.69 2.00 3.01 1.97 2.00 16.04 2.96

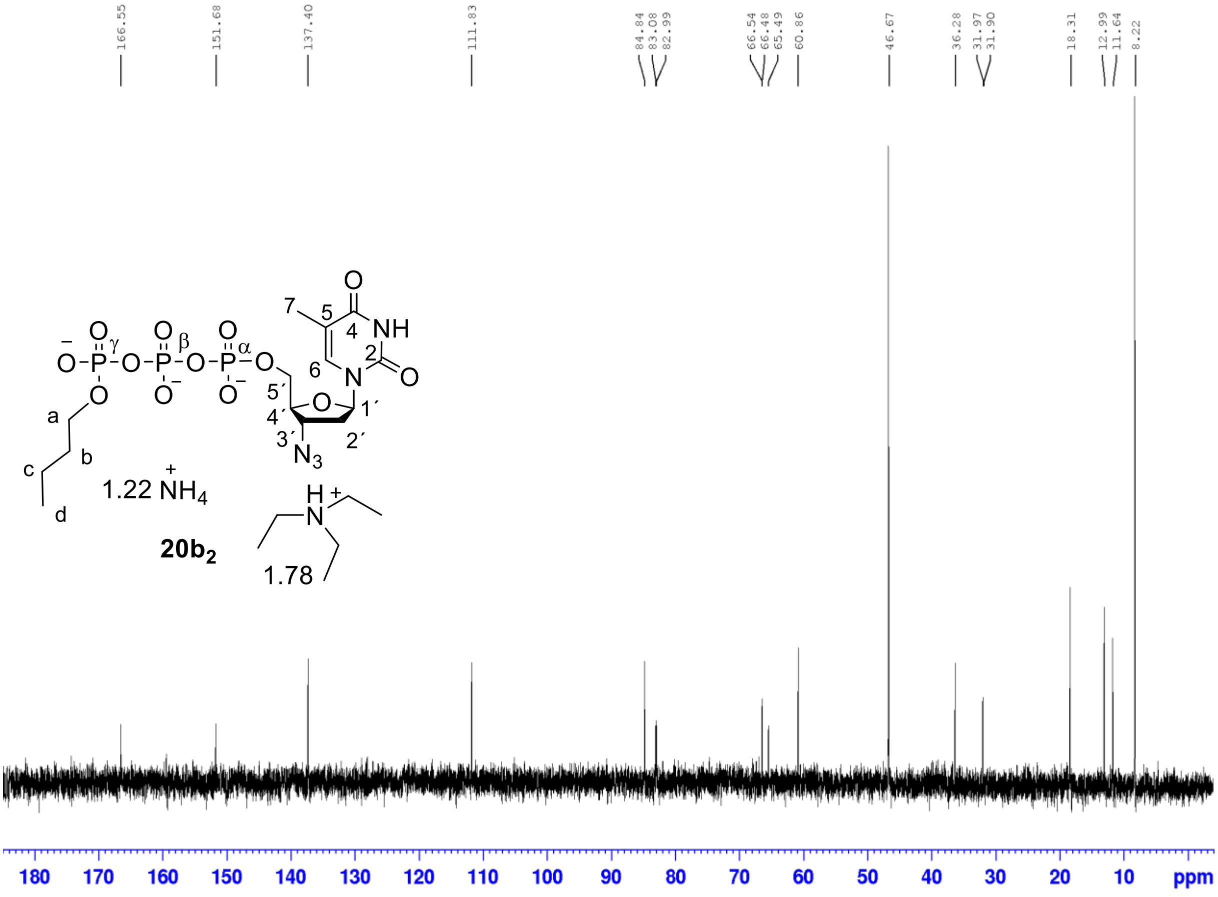
166.55 151.68 137.40 111.83 84.84 83.08 82.99 66.54 66.48 65.49 60.86 46.67 36.28 31.97 31.90 18.31 12.99 11.64 8.22
1.22 NH4
1.78
20b2
ppm

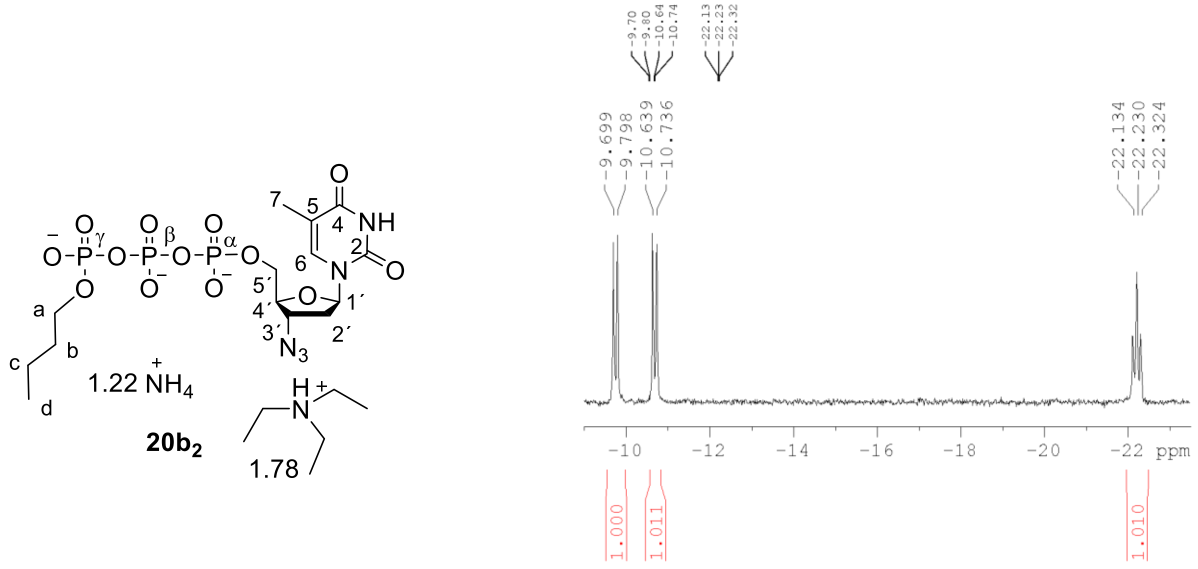
1.22 NH4
20b2
1.78
-9.699
-9.798
-10.639
-10.736
-22.134
-22.230
-22.324
ppm
1.000
1.011
1.010

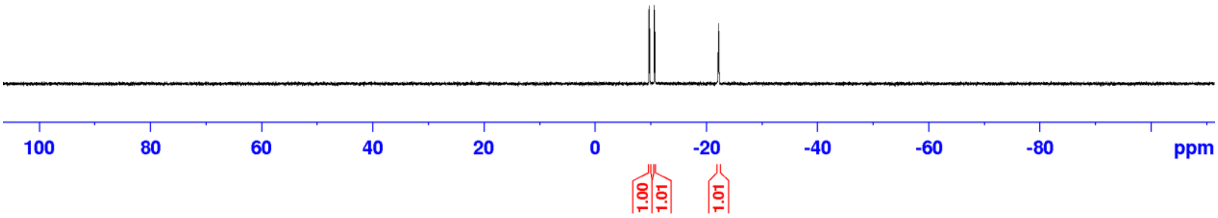
ppm
1.00
1.01
1.01

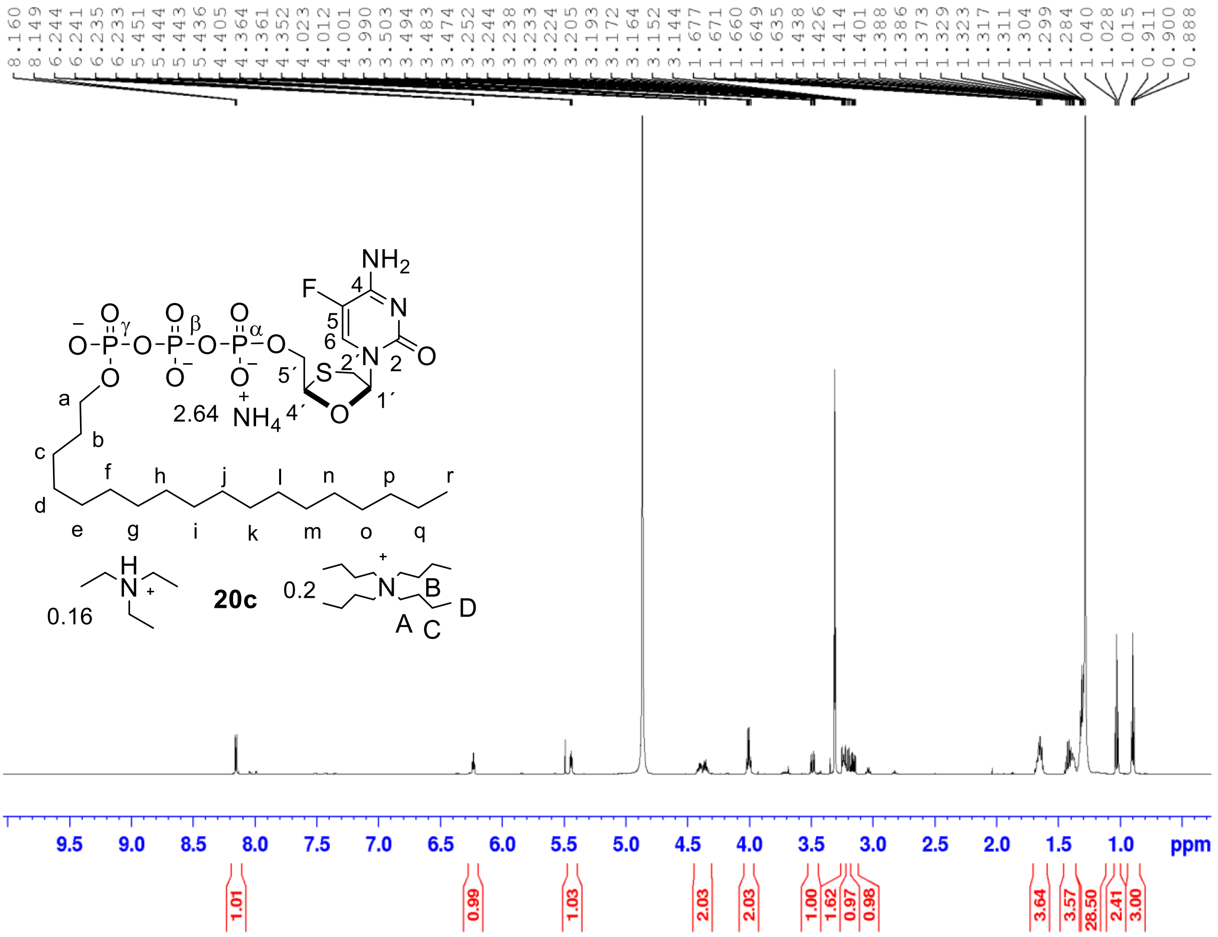
2.64 NH4
20c
0.16
0.2
ppm
1.01
0.99
1.03
2.03
2.03
1.00
1.62
0.97
0.98
3.64
3.57
28.50
2.41
3.00

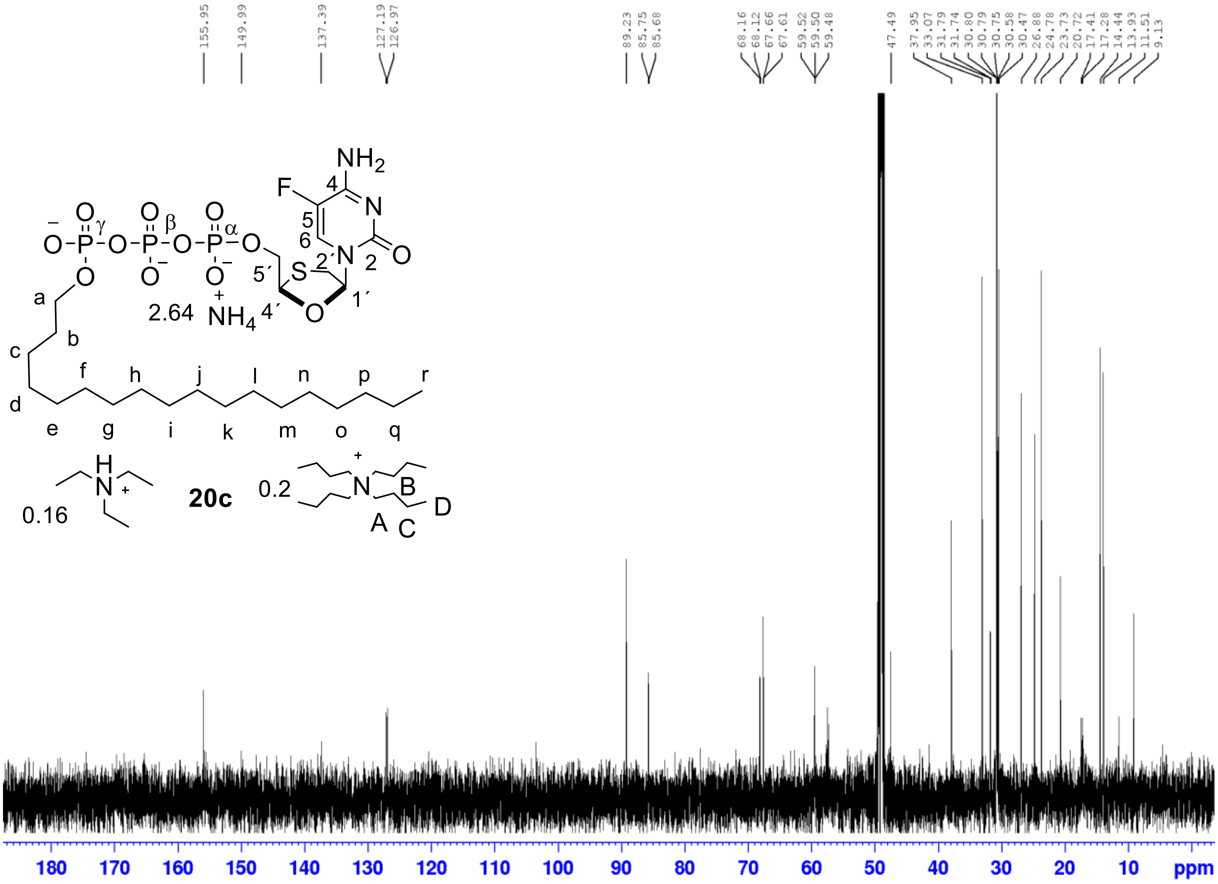
155.95
149.99
137.39
127.19
126.97
89.23
85.75
85.68
68.16
68.12
67.66
67.61
59.52
59.50
59.48
47.49
37.95
33.07
31.79
31.74
30.80
30.79
30.75
30.58
30.47
26.88
24.78
23.73
20.72
17.41
17.28
14.44
13.93
11.51
9.13
NH2
F
4
5
6
N
O
2
2´
1´
4´
5´
S
γ
β
α
2.64
NH4
a
b
c
d
e
f
g
h
i
j
k
l
m
n
o
p
q
r
0.16
20c
0.2
A
B
C
D
ppm

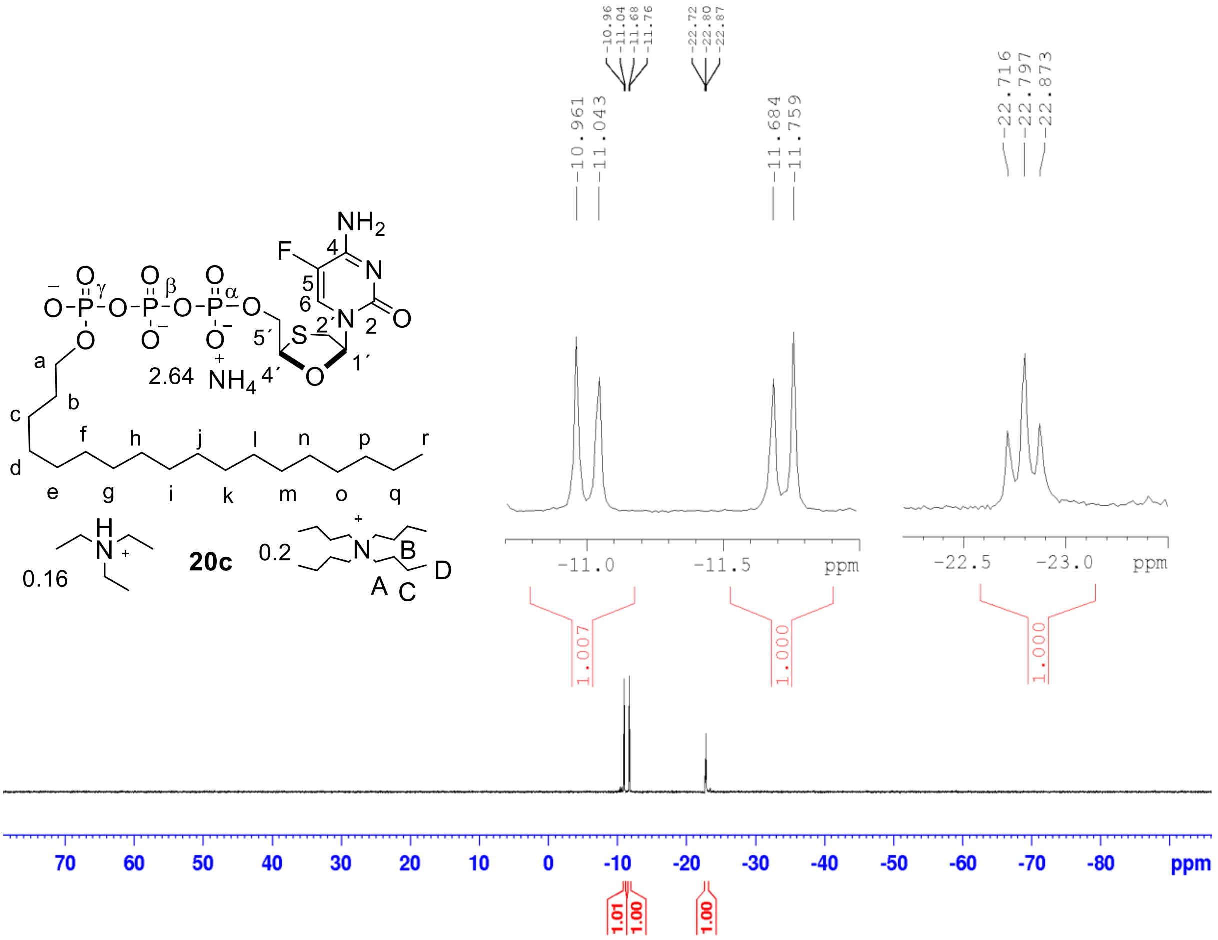
-10.96
-11.04
-11.68
-11.76
-22.72
-22.80
-22.87
-10.961
-11.043
-11.684
-11.759
-22.716
-22.797
-22.873
-11.0
-11.5
ppm
-22.5
-23.0
1.007
1.000
1.000
0.16
20c
0.2
2.64
NH4
ppm
1.01
1.00
1.00

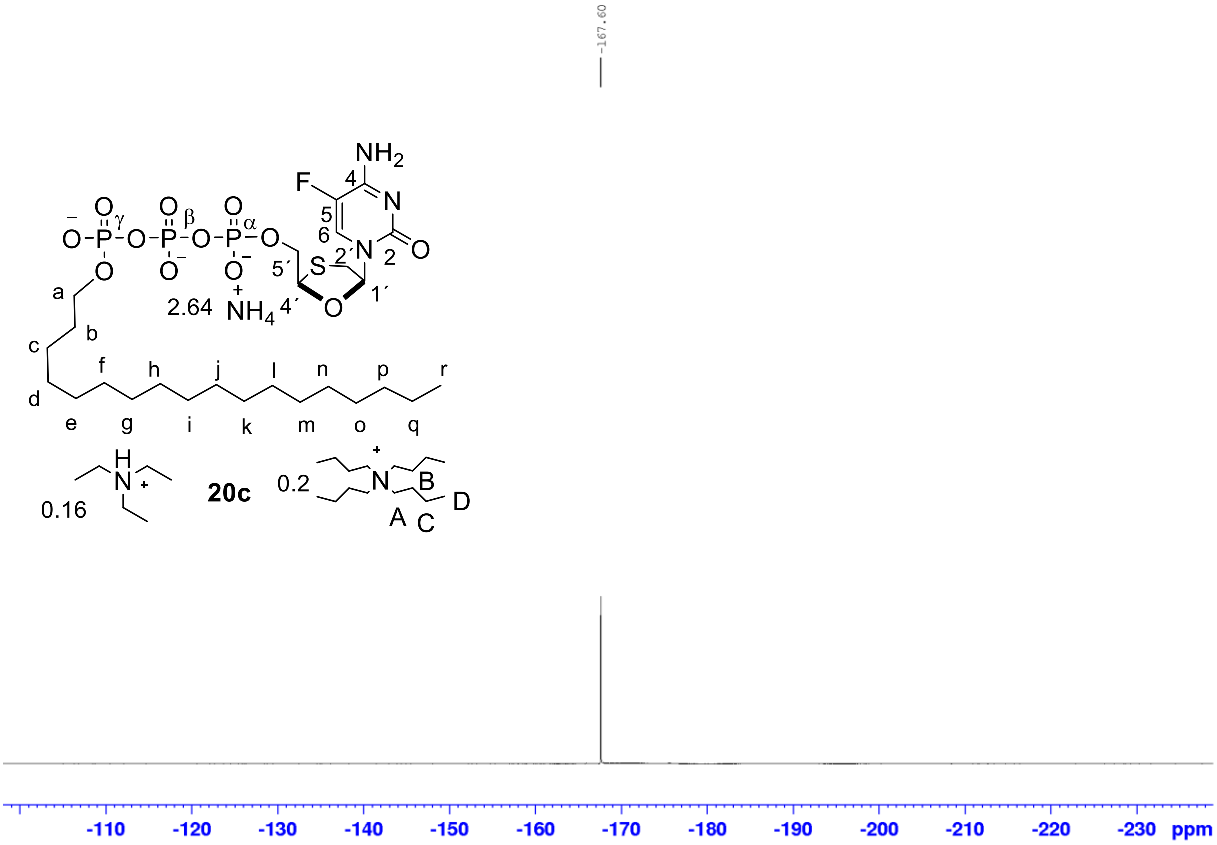
-167.60
2.64
0.16
20c
0.2
ppm

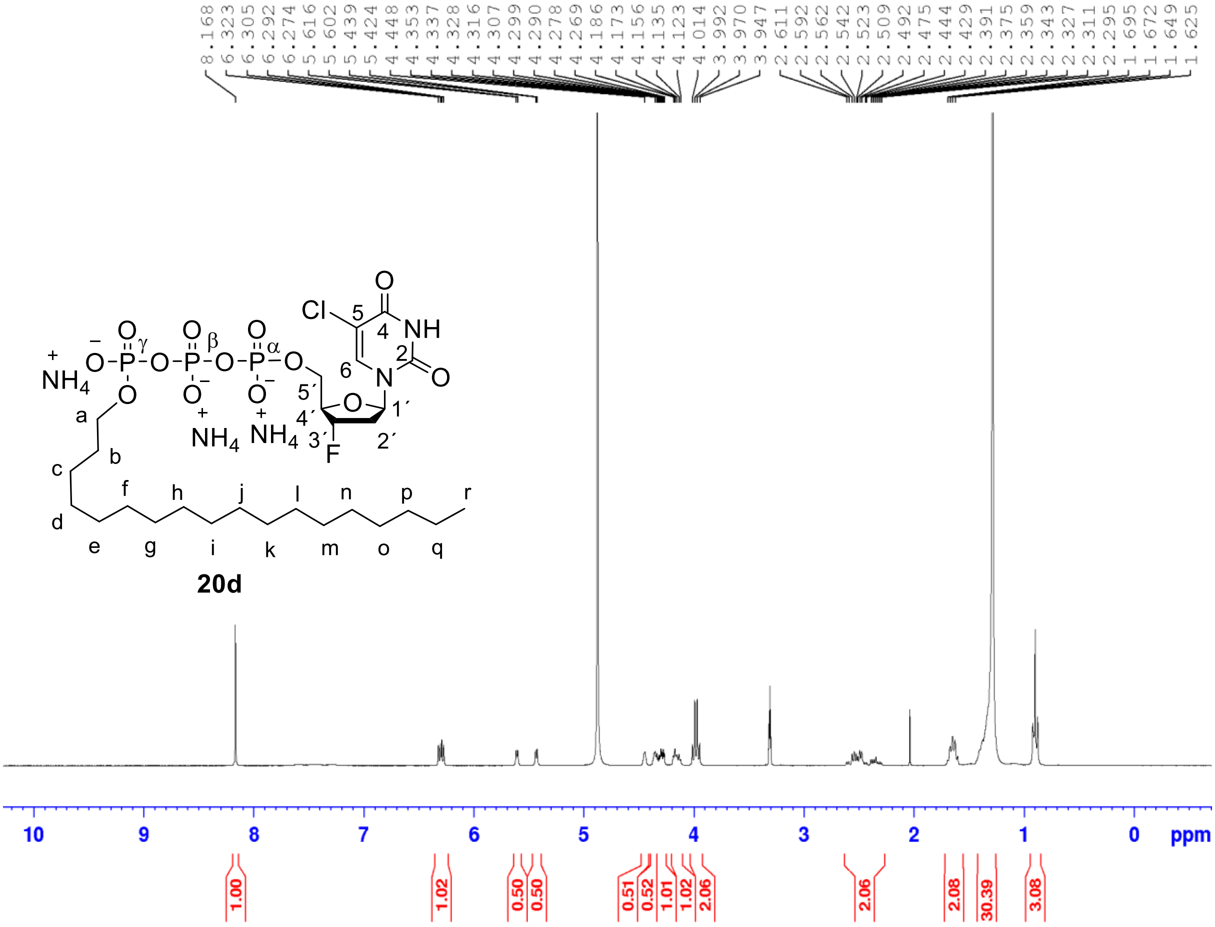
20d
ppm
1.00
1.02
0.50
0.50
0.51
0.52
1.01
1.02
2.06
2.06
2.08
30.39
3.08

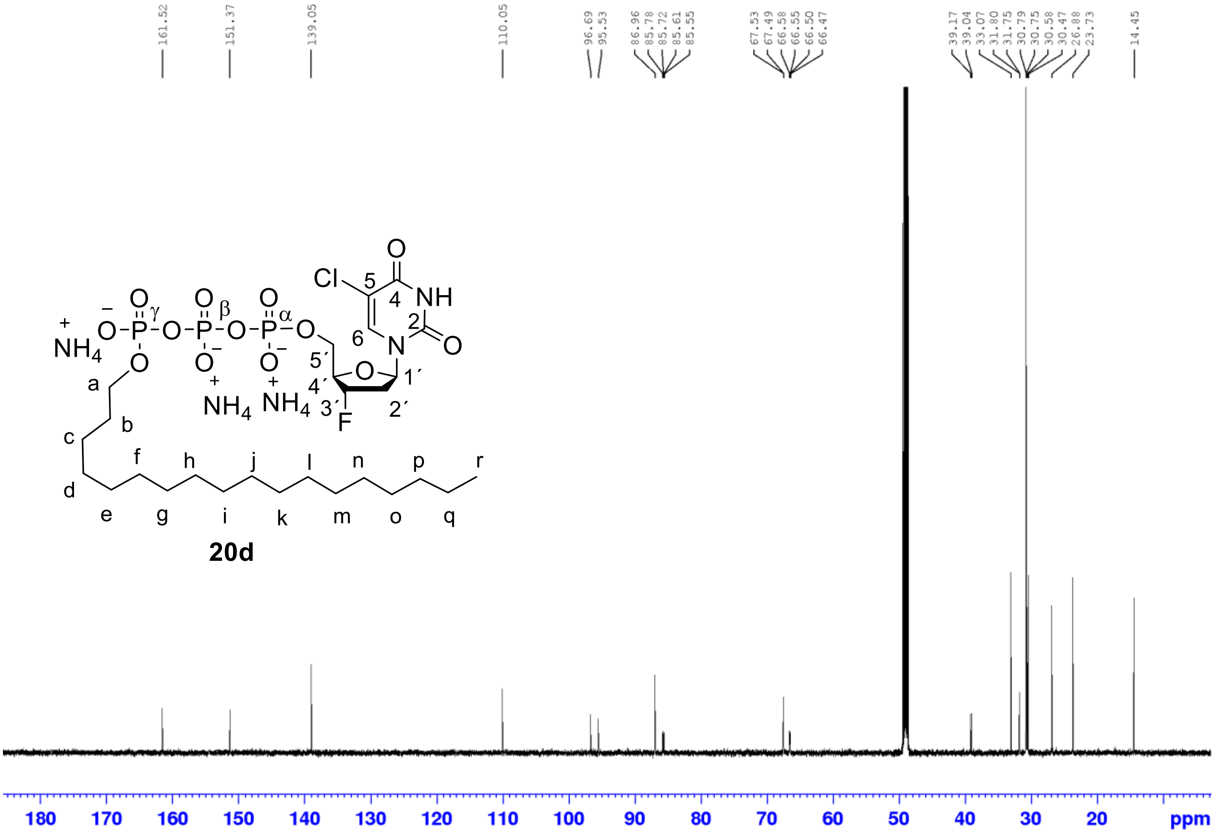

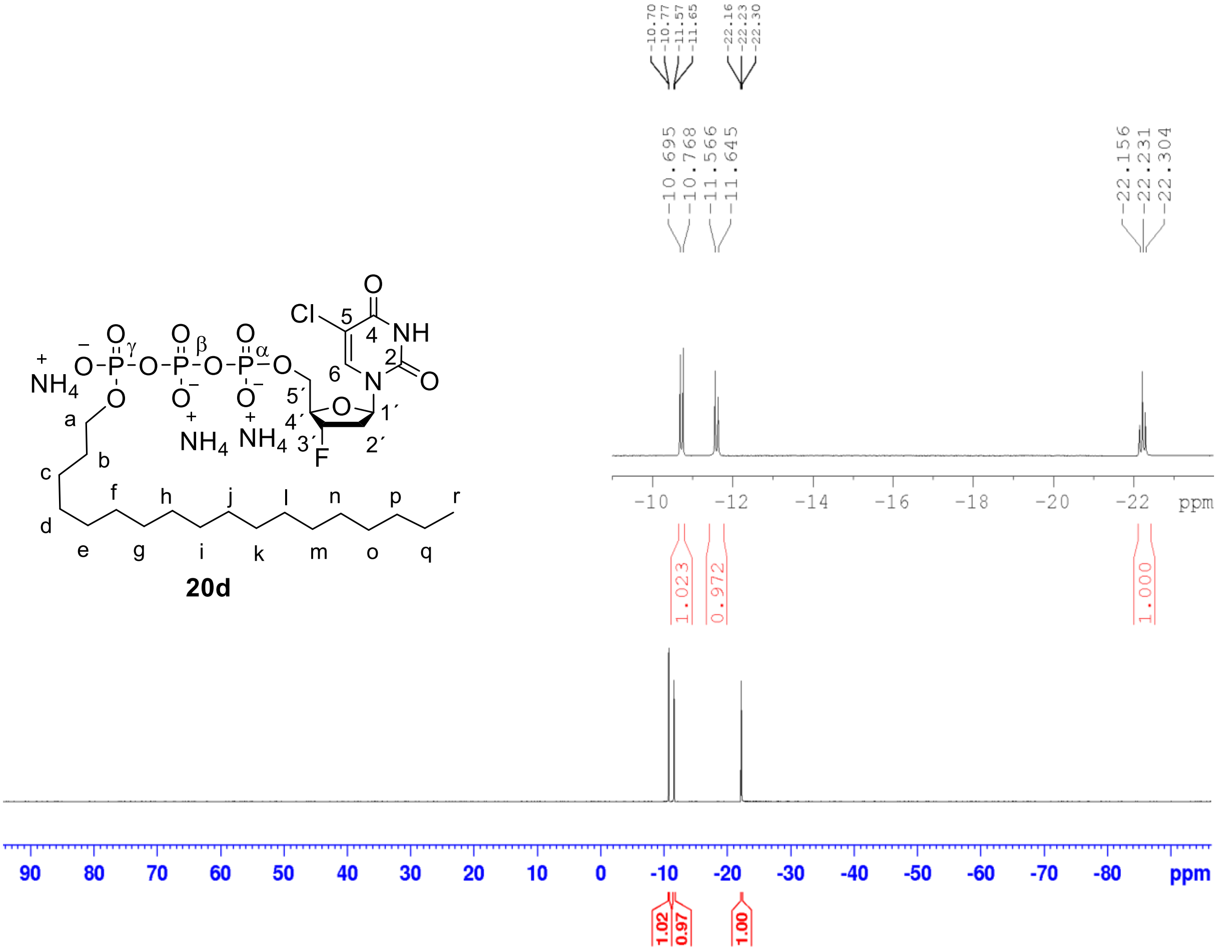

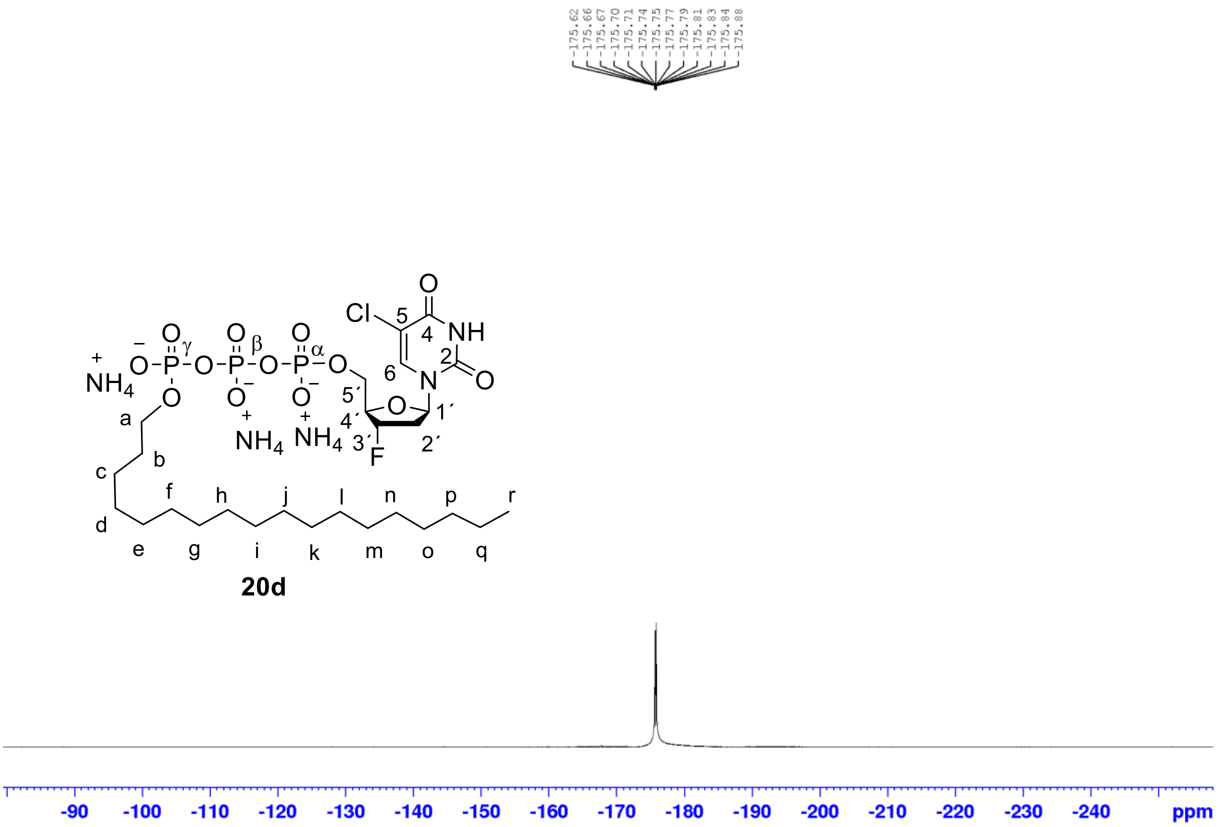
-175.62 -175.66 -175.67 -175.70 -175.71 -175.74 -175.75 -175.77 -175.79 -175.81 -175.83 -175.84 -175.88
20d
-90 -100 -110 -120 -130 -140 -150 -160 -170 -180 -190 -200 -210 -220 -230 -240 ppm

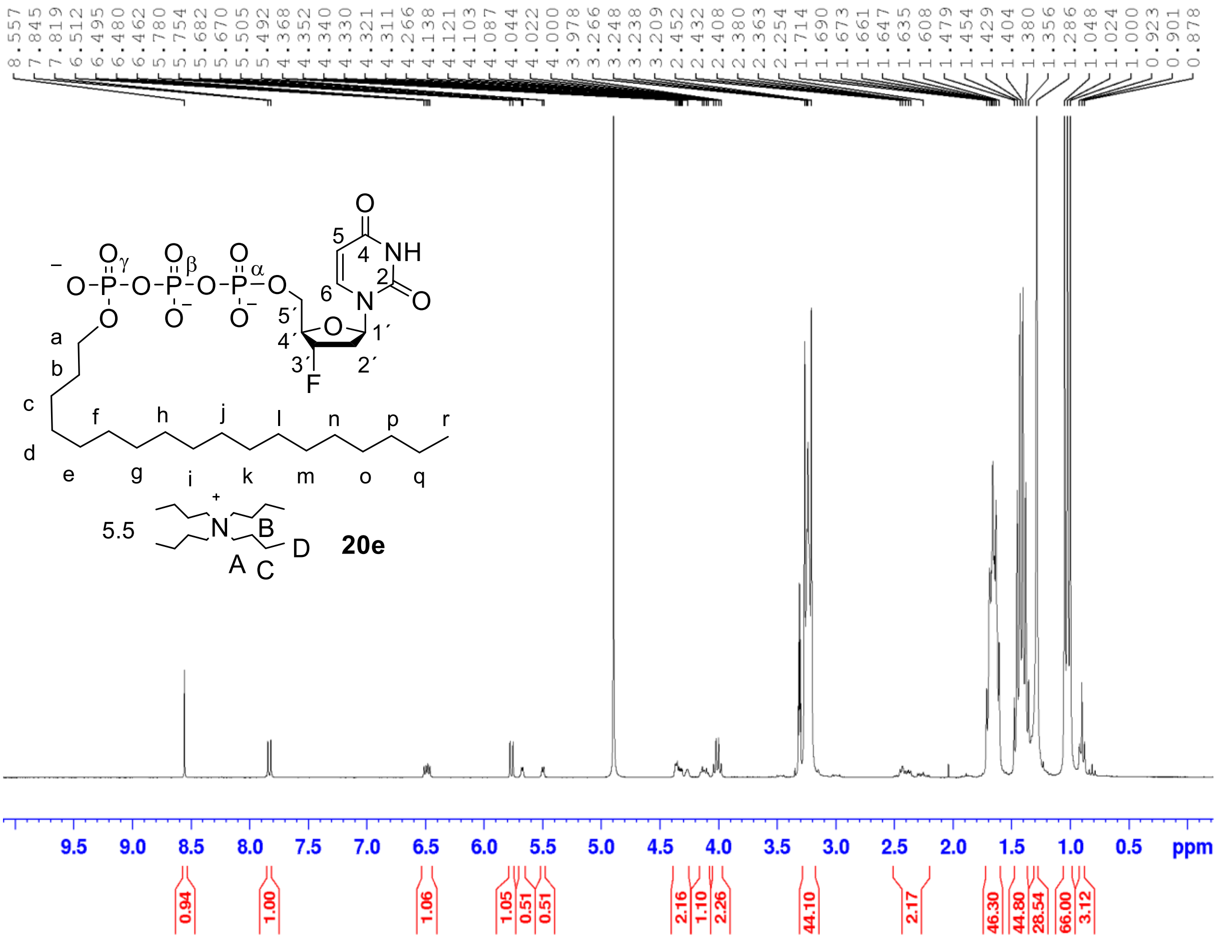
8.557 7.845 7.819 6.512 6.495 6.480 6.462 5.780 5.754 5.682 5.670 5.505 5.492 4.368 4.352 4.340 4.330 4.321 4.311 4.266 4.138 4.121 4.103 4.087 4.044 4.022 4.000 3.978 3.266 3.248 3.238 3.209 2.452 2.432 2.408 2.380 2.363 2.254 1.714 1.690 1.673 1.661 1.647 1.635 1.608 1.479 1.454 1.429 1.404 1.380 1.356 1.286 1.048 1.024 1.000 0.923 0.901 0.878
5.5
20e
9.5 9.0 8.5 8.0 7.5 7.0 6.5 6.0 5.5 5.0 4.5 4.0 3.5 3.0 2.5 2.0 1.5 1.0 0.5 ppm
0.94 1.00 1.06 1.05 0.51 0.51 2.16 1.10 2.26 44.10 2.17 46.30 44.80 28.54 66.00 3.12

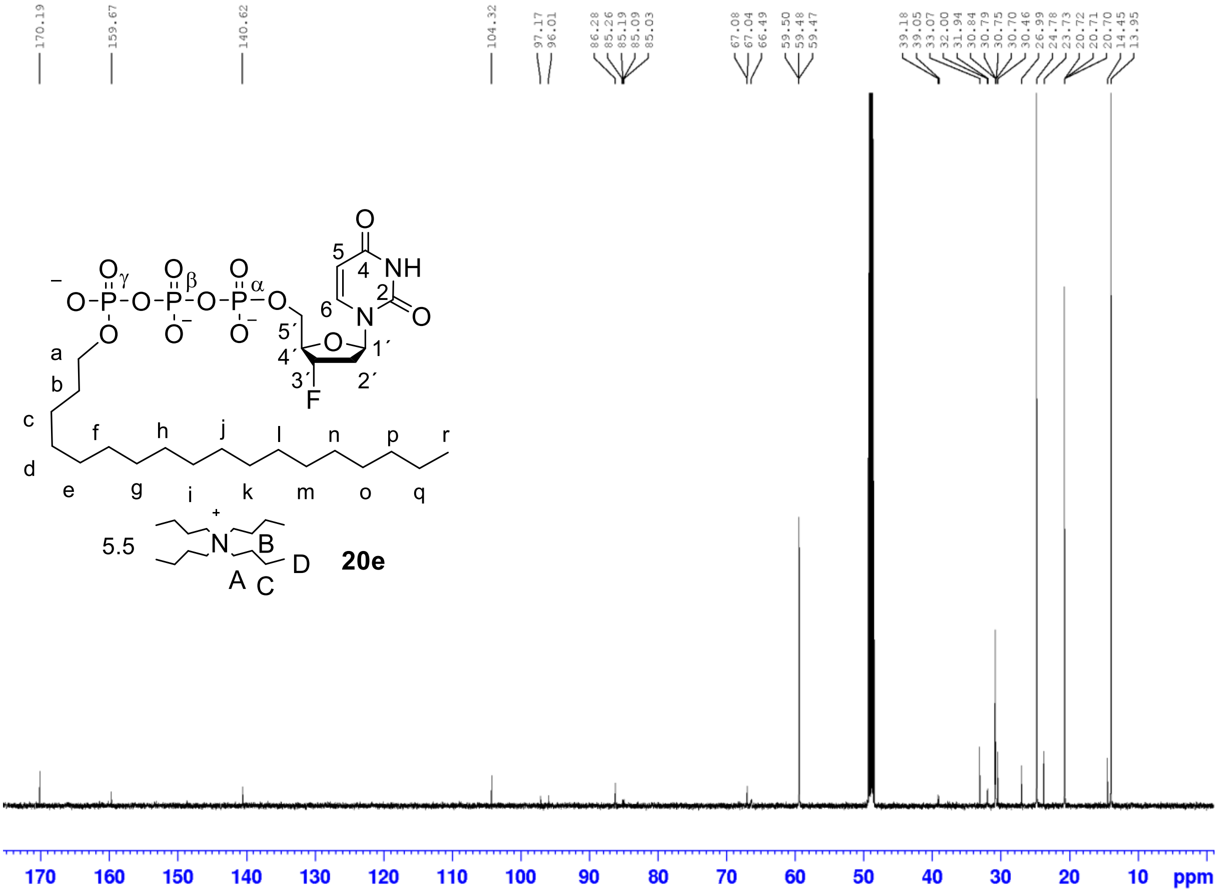
170.19
159.67
140.62
104.32
97.17
96.01
86.28
85.26
85.19
85.09
85.03
67.08
67.04
66.49
59.50
59.48
59.47
39.18
39.05
33.07
32.00
31.94
30.84
30.79
30.75
30.70
30.46
26.99
24.78
23.73
20.72
20.71
20.70
14.45
13.95
5.5
20e
ppm

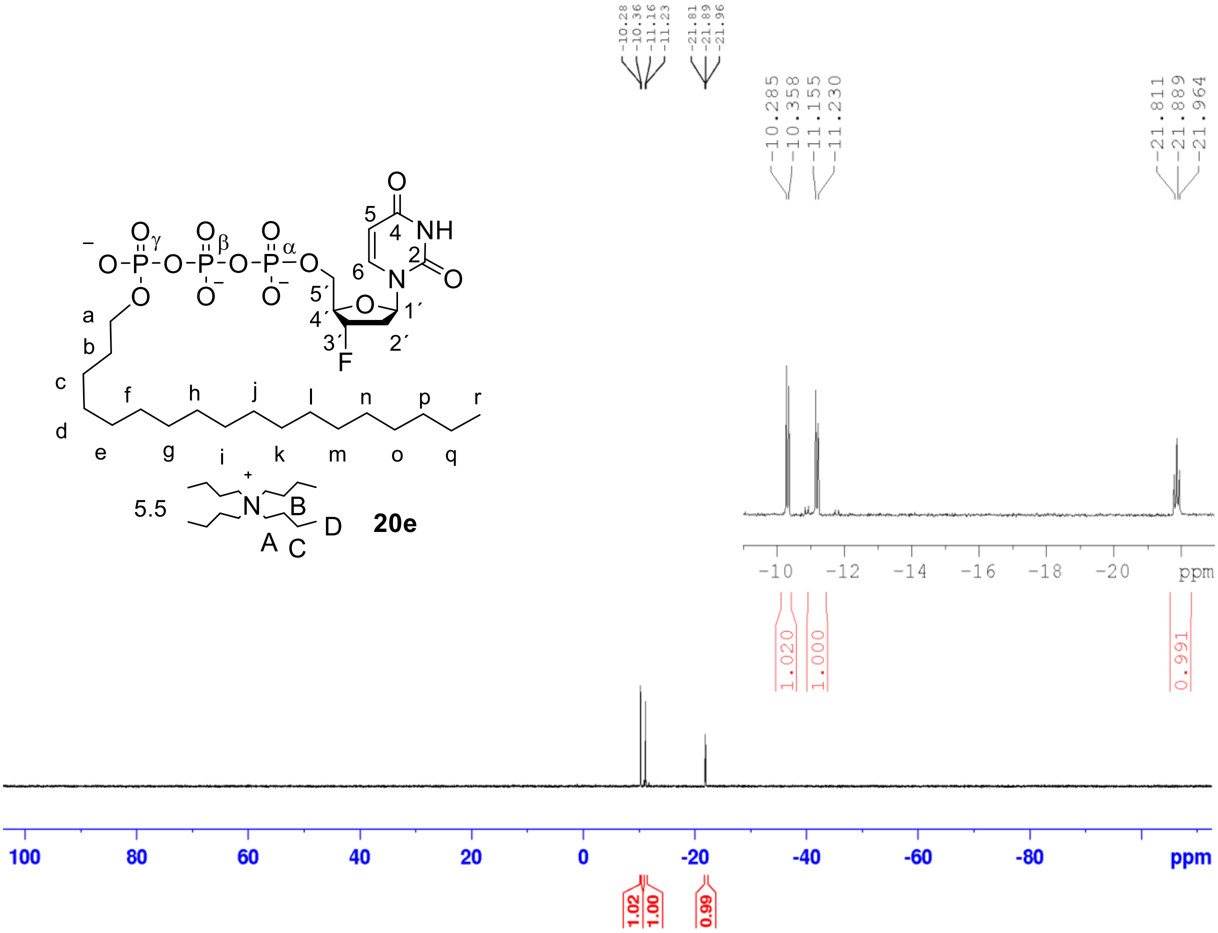
-10.28
-10.36
-11.16
-11.23
-21.81
-21.89
-21.96
-10.285
-10.358
-11.155
-11.230
-21.811
-21.889
-21.964
1.020
1.000
0.991
5.5
20e
ppm
1.02
1.00
0.99

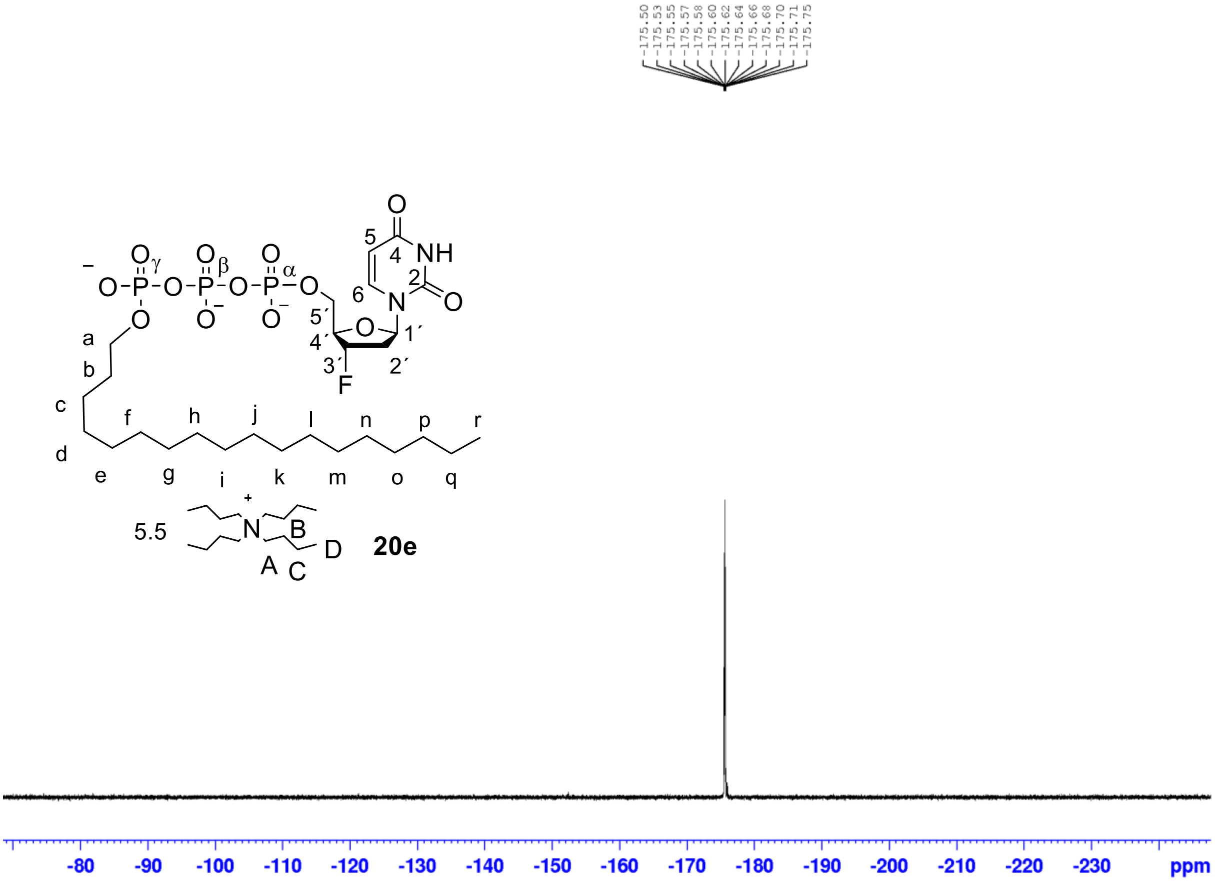
-175.50
-175.53
-175.55
-175.57
-175.58
-175.60
-175.62
-175.64
-175.66
-175.68
-175.70
-175.71
-175.75
5.5
20e
-80 -90 -100 -110 -120 -130 -140 -150 -160 -170 -180 -190 -200 -210 -220 -230 ppm

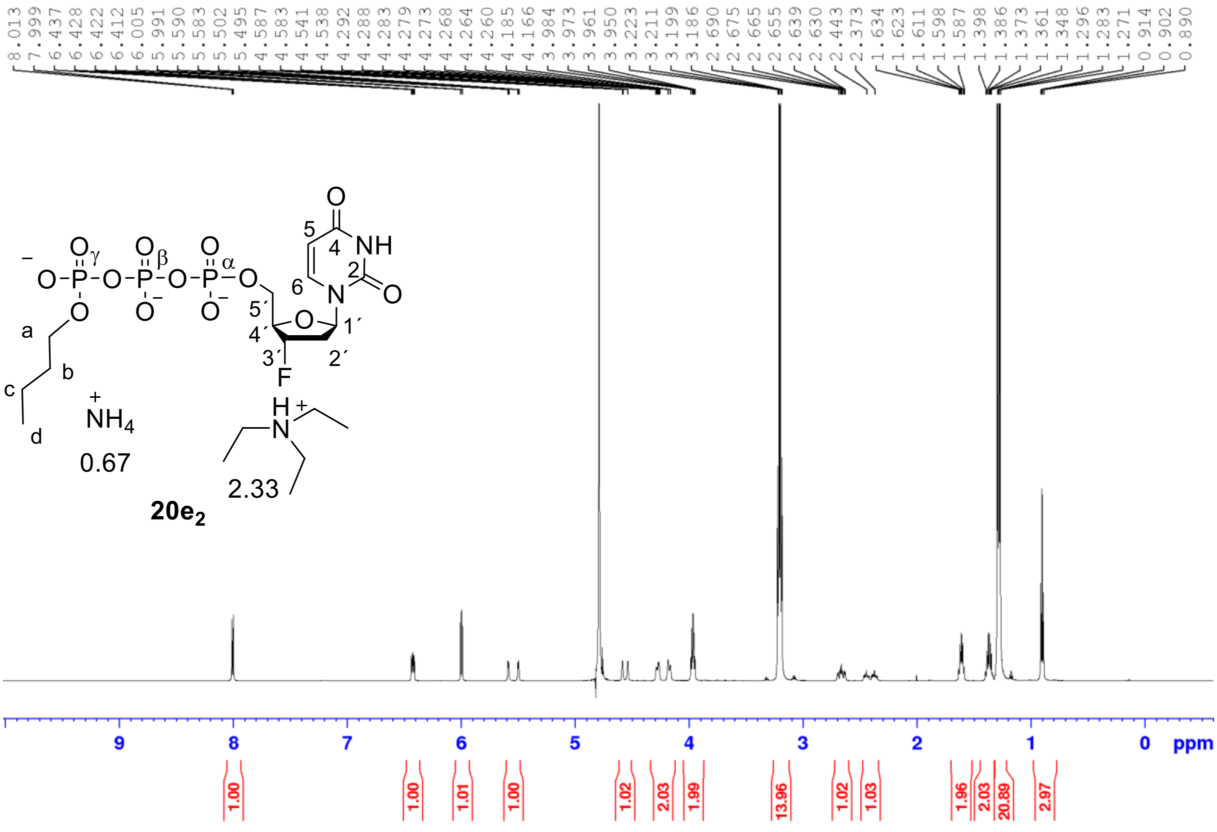
8.013
7.999
6.437
6.428
6.422
6.412
6.005
5.991
5.590
5.583
5.502
5.495
4.587
4.583
4.541
4.538
4.292
4.288
4.283
4.279
4.273
4.268
4.264
4.260
4.185
4.166
3.984
3.973
3.961
3.950
3.223
3.211
3.199
3.186
2.690
2.675
2.665
2.655
2.639
2.630
2.443
2.373
1.634
1.623
1.611
1.598
1.587
1.398
1.386
1.373
1.361
1.348
1.296
1.283
1.271
0.914
0.902
0.890
0.67
2.33
20e2
9 8 7 6 5 4 3 2 1 0 ppm
1.00 1.00 1.01 1.00 1.02 2.03 1.99 13.96 1.02 1.03 1.96 2.03 20.89 2.97

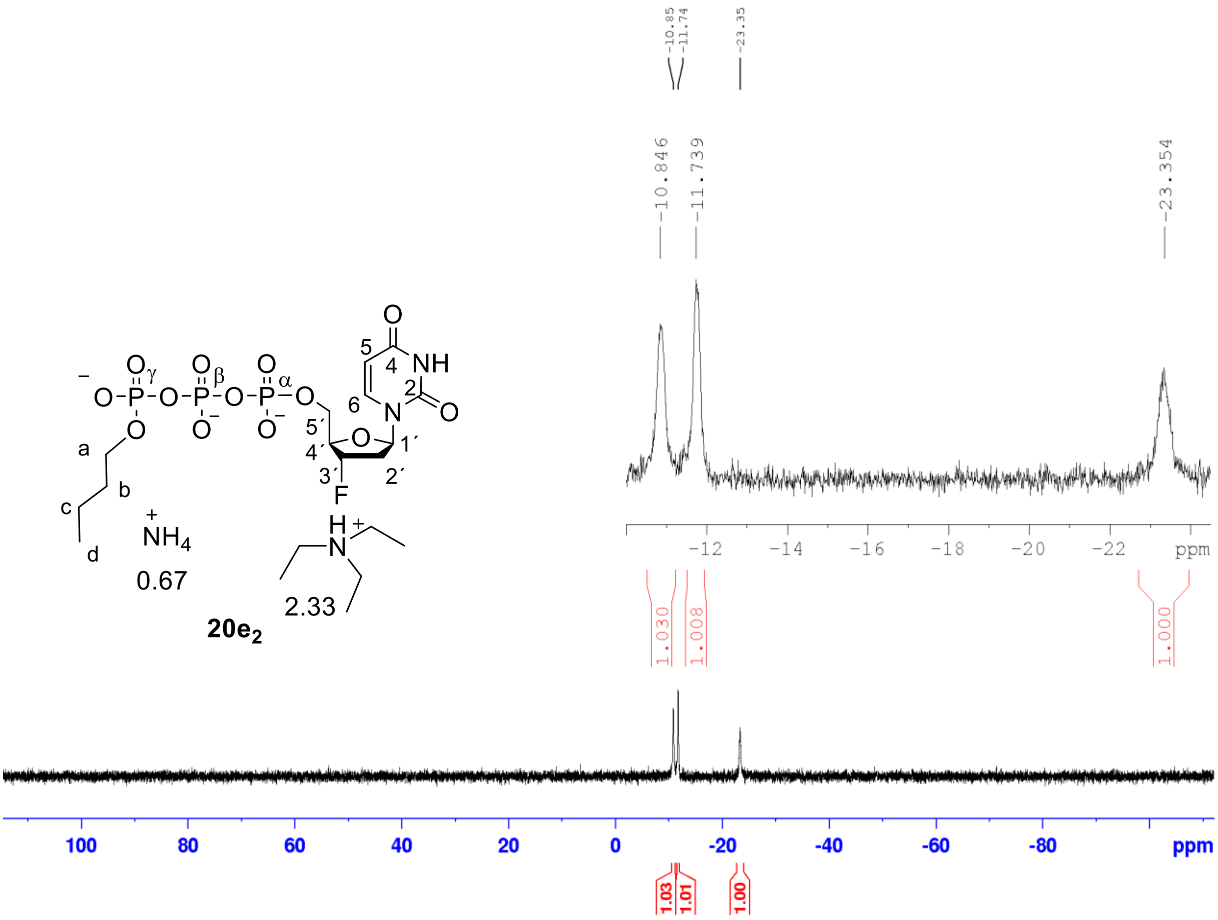
20e₂
0.67
2.33
-10.846
-11.739
-23.354
1.030
1.008
1.000
-10.85
-11.74
-23.35
1.03
1.01
1.00
ppm

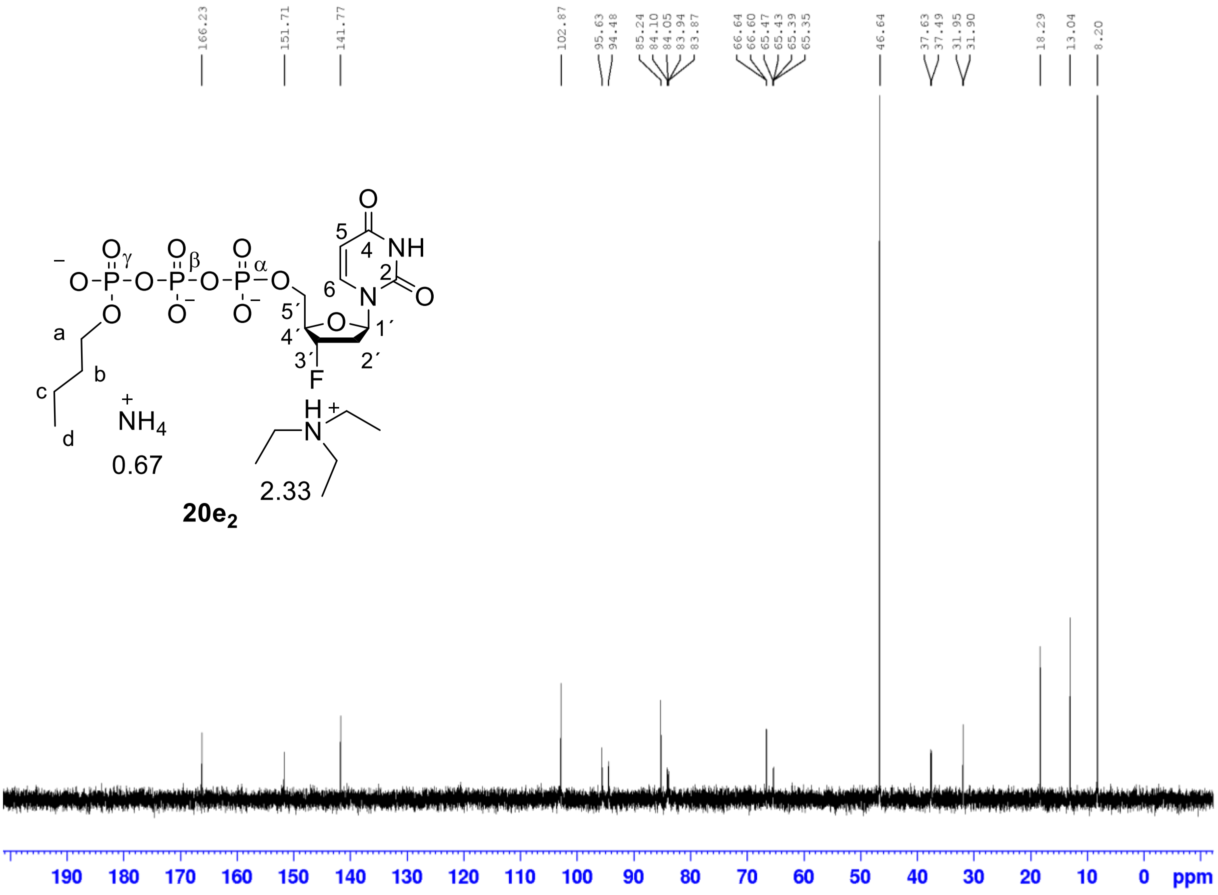
20e₂
0.67
2.33
166.23
151.71
141.77
102.87
95.63
94.48
85.24
84.10
84.05
83.94
83.87
66.64
66.60
65.47
65.43
65.39
65.35
46.64
37.63
37.49
31.95
31.90
18.29
13.04
8.20
ppm

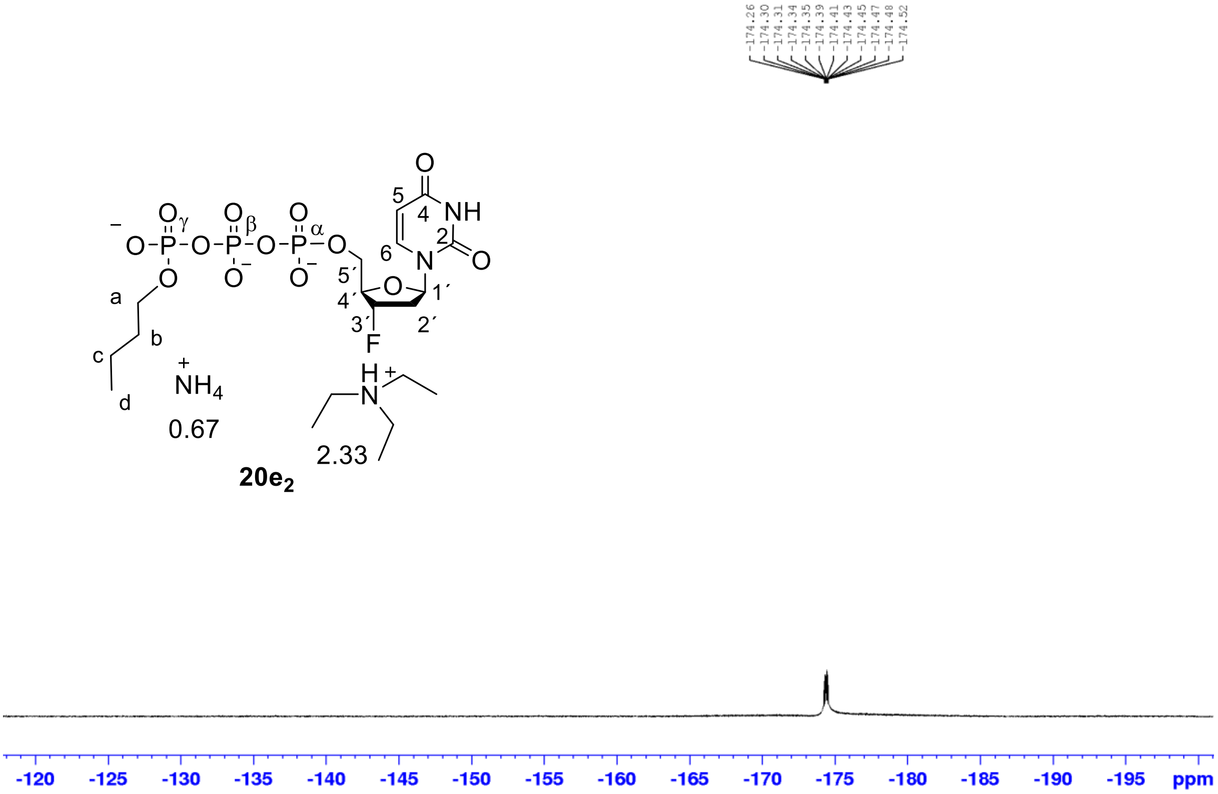

0.67
2.33
$20e_2$
ppm

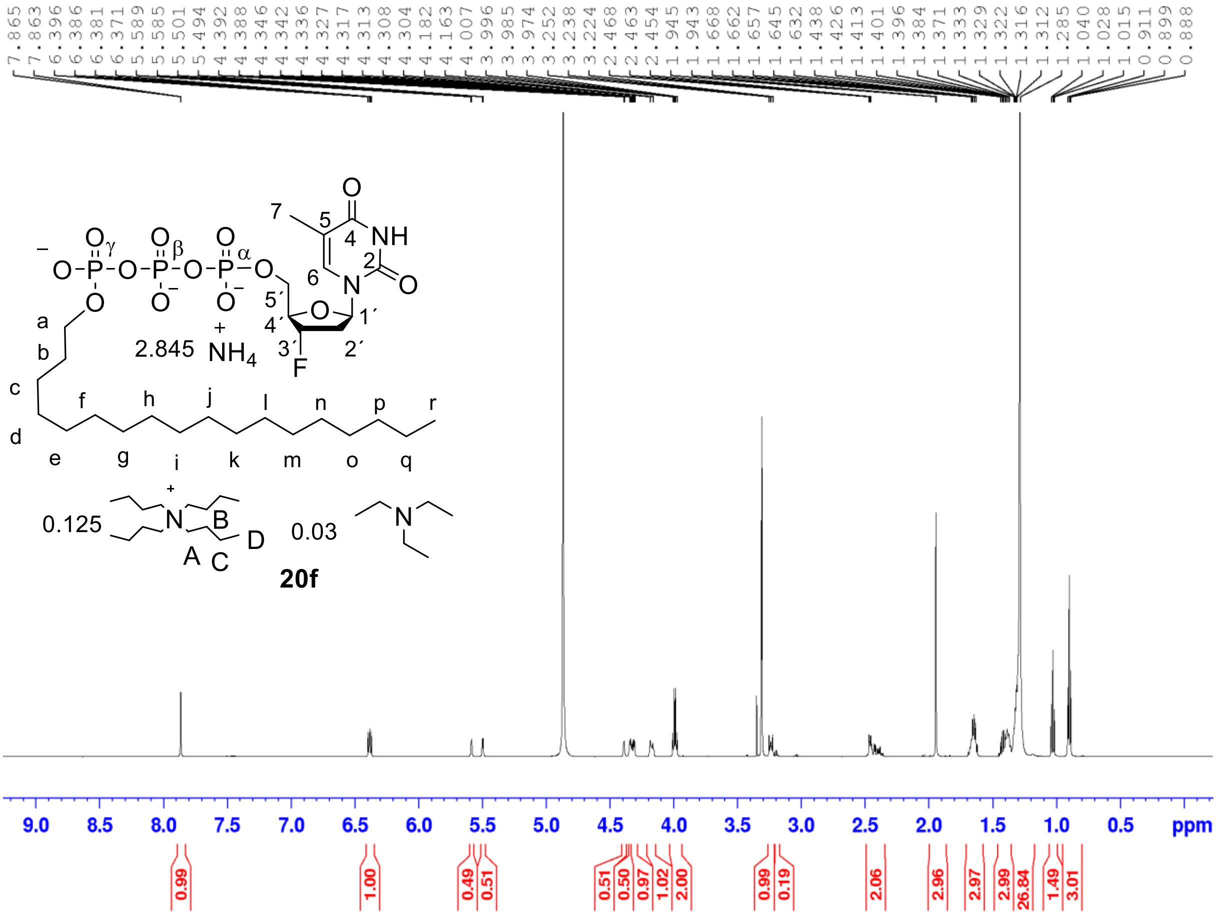

2.845
0.125
0.03
20f
ppm

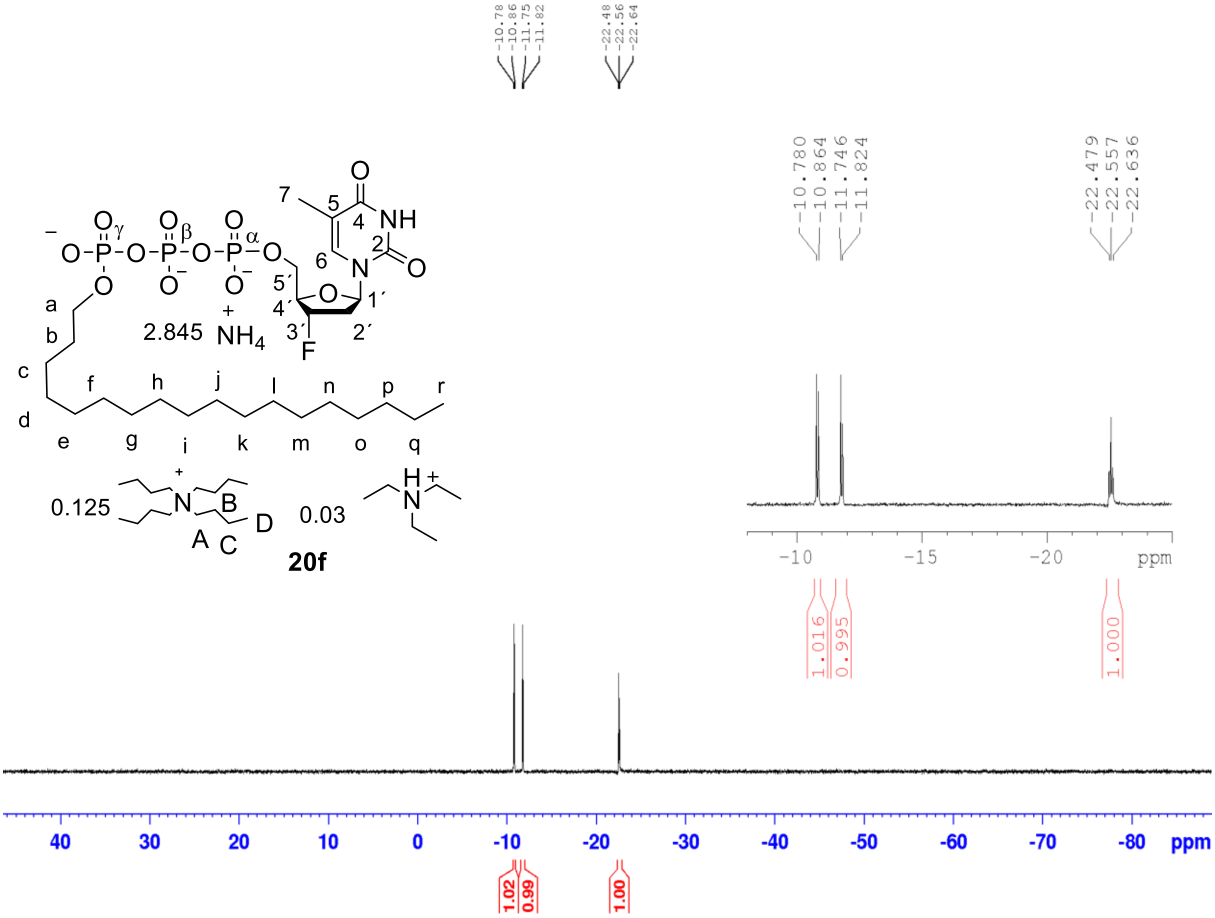
-10.78
-10.86
-11.75
-11.82
-22.48
-22.56
-22.64
-10.780
-10.864
-11.746
-11.824
-22.479
-22.557
-22.636
2.845
0.125
0.03
20f
1.016
0.995
1.000
1.02
0.99
1.00
ppm

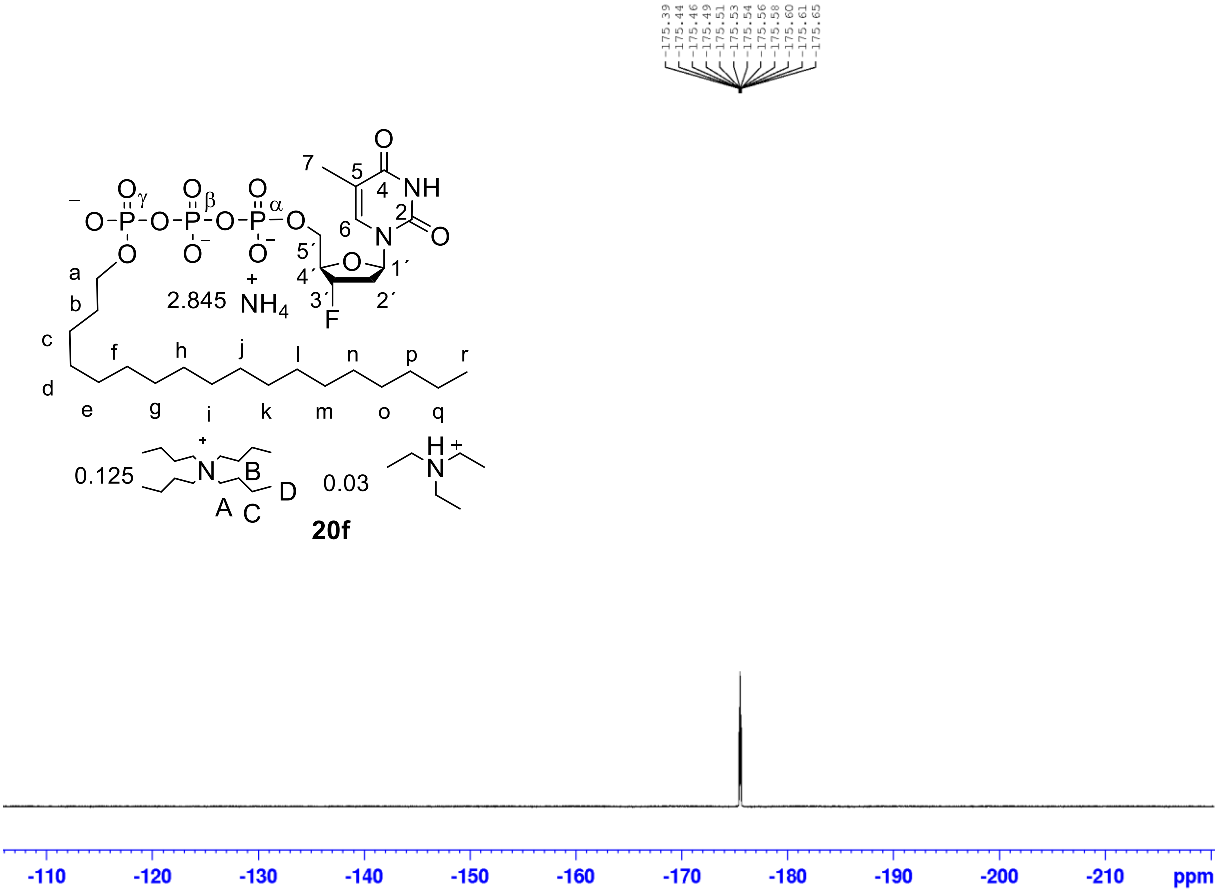
-175.39
-175.44
-175.46
-175.49
-175.51
-175.53
-175.54
-175.56
-175.58
-175.60
-175.61
-175.65
2.845
0.125
0.03
20f
ppm

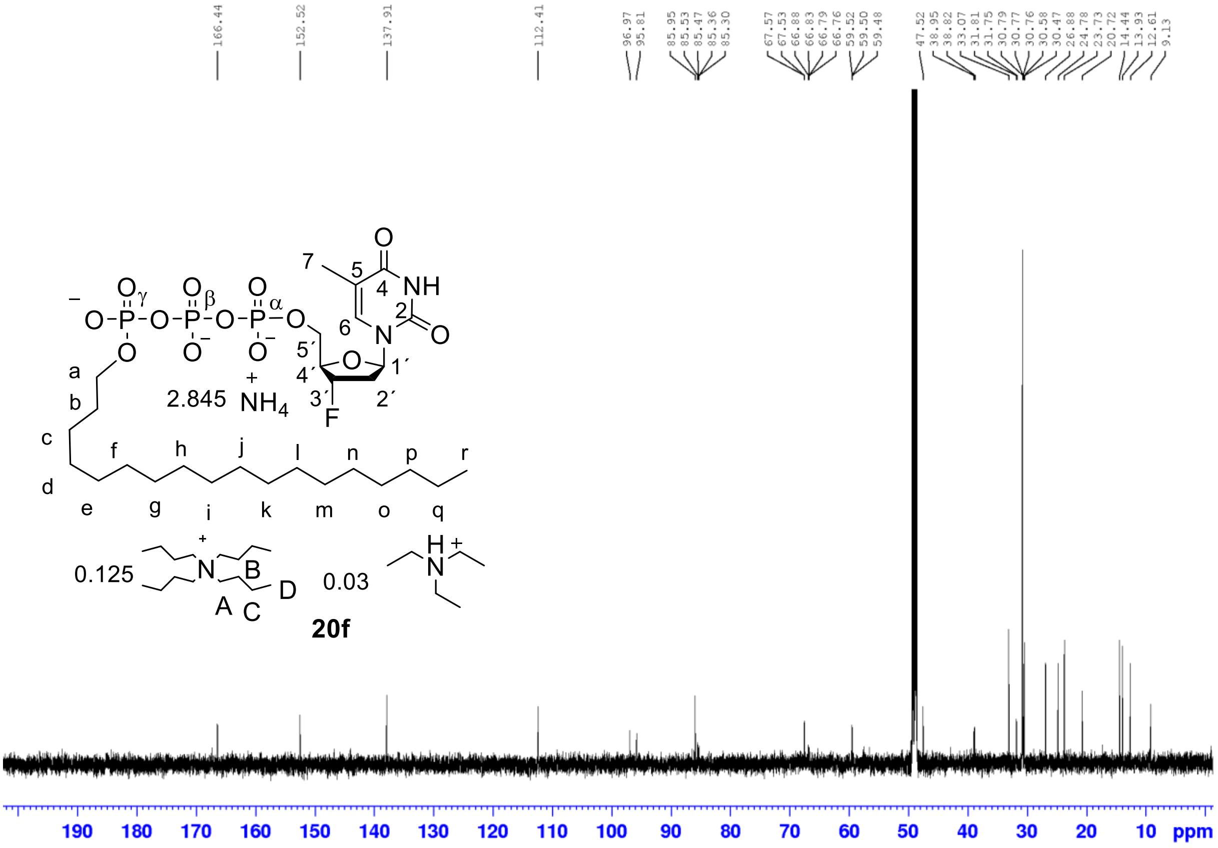

20f
2.845
0.125
0.03
ppm

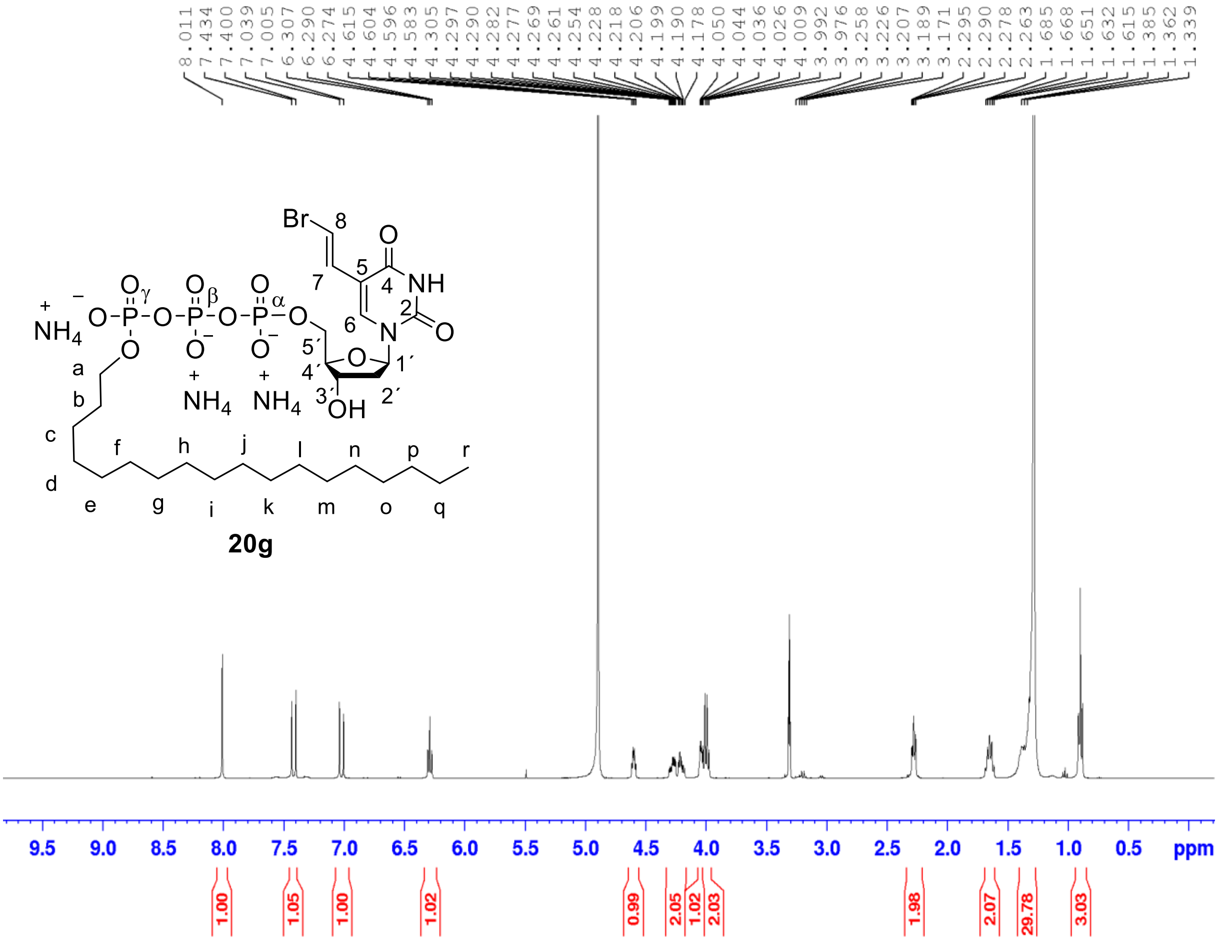

20g
ppm

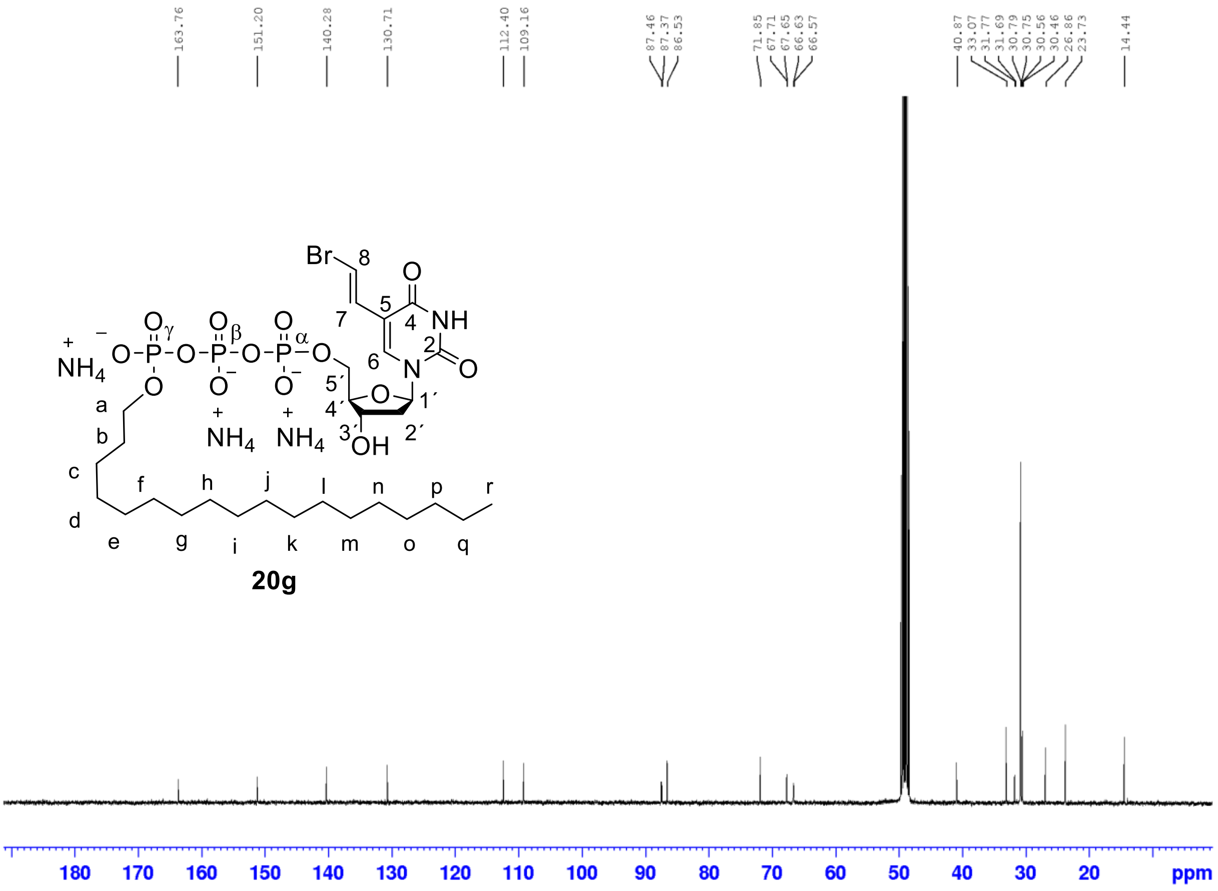
163.76
151.20
140.28
130.71
112.40
109.16
87.46
87.37
86.53
71.85
67.71
67.65
66.63
66.57
40.87
33.07
31.77
31.69
30.79
30.75
30.56
30.46
26.86
23.73
14.44
20g
180 170 160 150 140 130 120 110 100 90 80 70 60 50 40 30 20 ppm

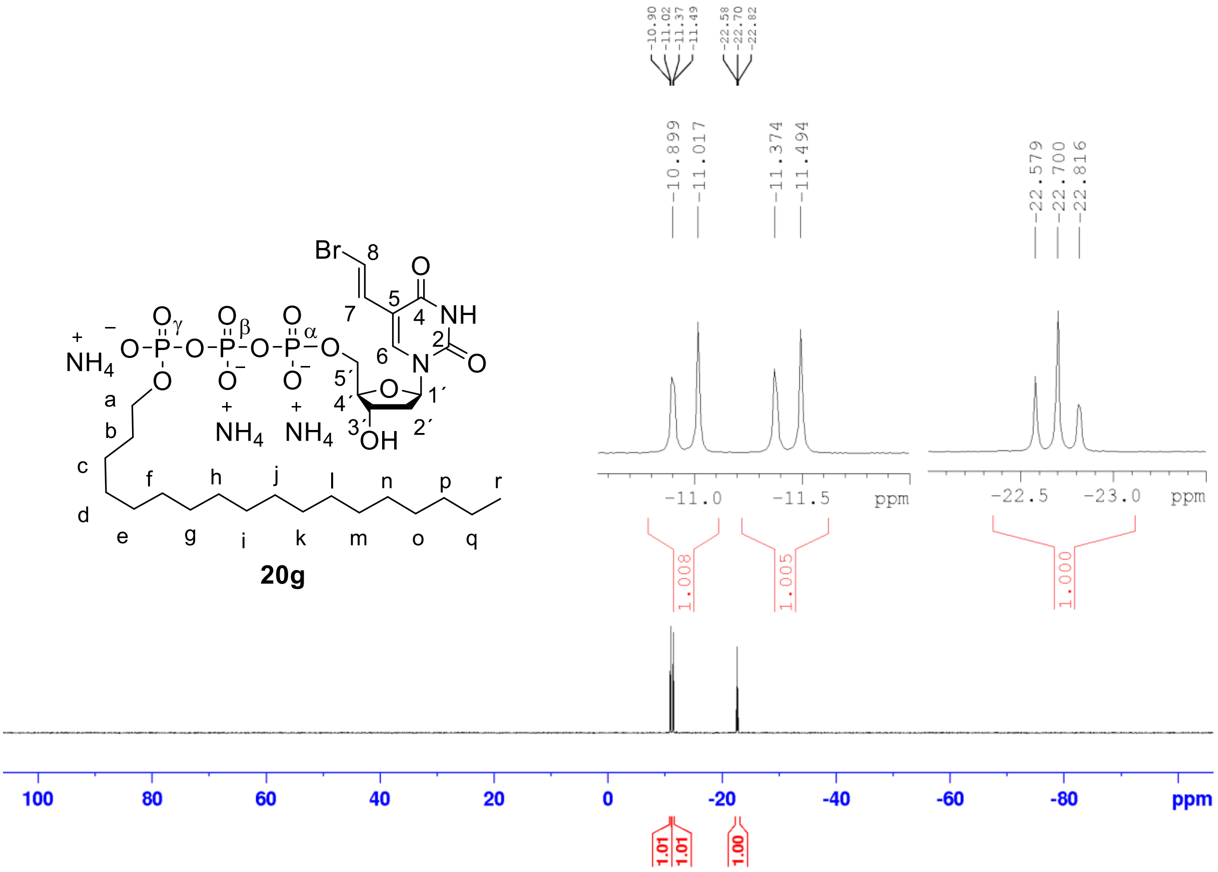
-10.90
-11.02
-11.37
-11.49
-22.58
-22.70
-22.82
-10.899
-11.017
-11.374
-11.494
-22.579
-22.700
-22.816
20g
-11.0
-11.5
ppm
-22.5
-23.0
ppm
1.008
1.005
1.000
100 80 60 40 20 0 -20 -40 -60 -80 ppm
1.01
1.01
1.00

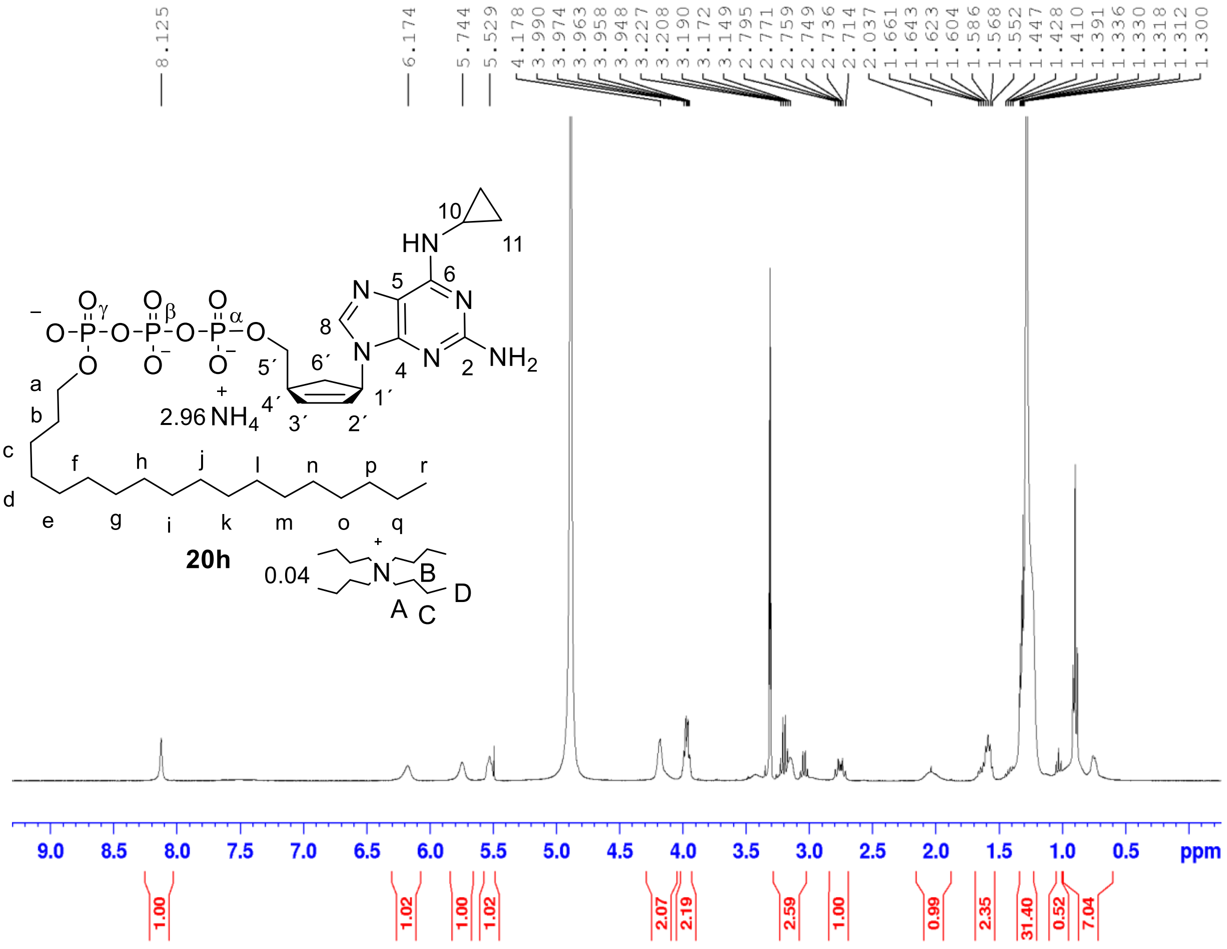
8.125
6.174
5.744
5.529
4.178 3.990 3.974 3.963 3.958 3.948 3.227 3.208 3.190 3.172 3.149 2.795 2.771 2.759 2.749 2.736 2.714 2.037 1.661 1.643 1.623 1.604 1.586 1.568 1.552 1.447 1.428 1.410 1.391 1.336 1.330 1.318 1.312 1.300
20h
2.96 NH4+
0.04
ppm
1.00 1.02 1.00 1.02 2.07 2.19 2.59 1.00 0.99 2.35 31.40 0.52 7.04

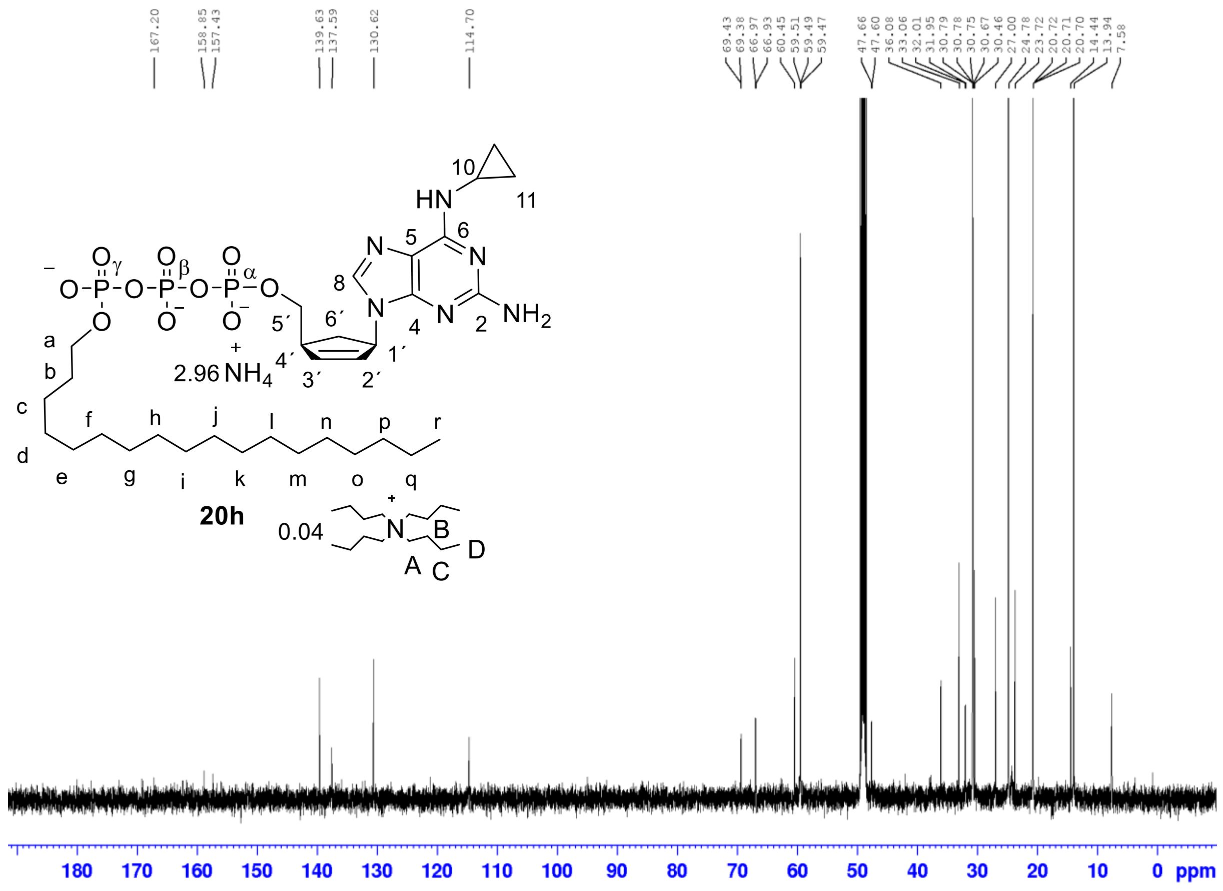
167.20
158.85 157.43
139.63 137.59
130.62
114.70
69.43 69.38 66.97 66.93 60.45 59.51 59.49 59.47
47.66 47.60 36.08 33.06 32.01 31.95 30.79 30.78 30.75 30.67 30.46 27.00 24.78 23.72 20.72 20.71 20.70 14.44 13.94 7.58
20h
2.96 NH4+
0.04
ppm

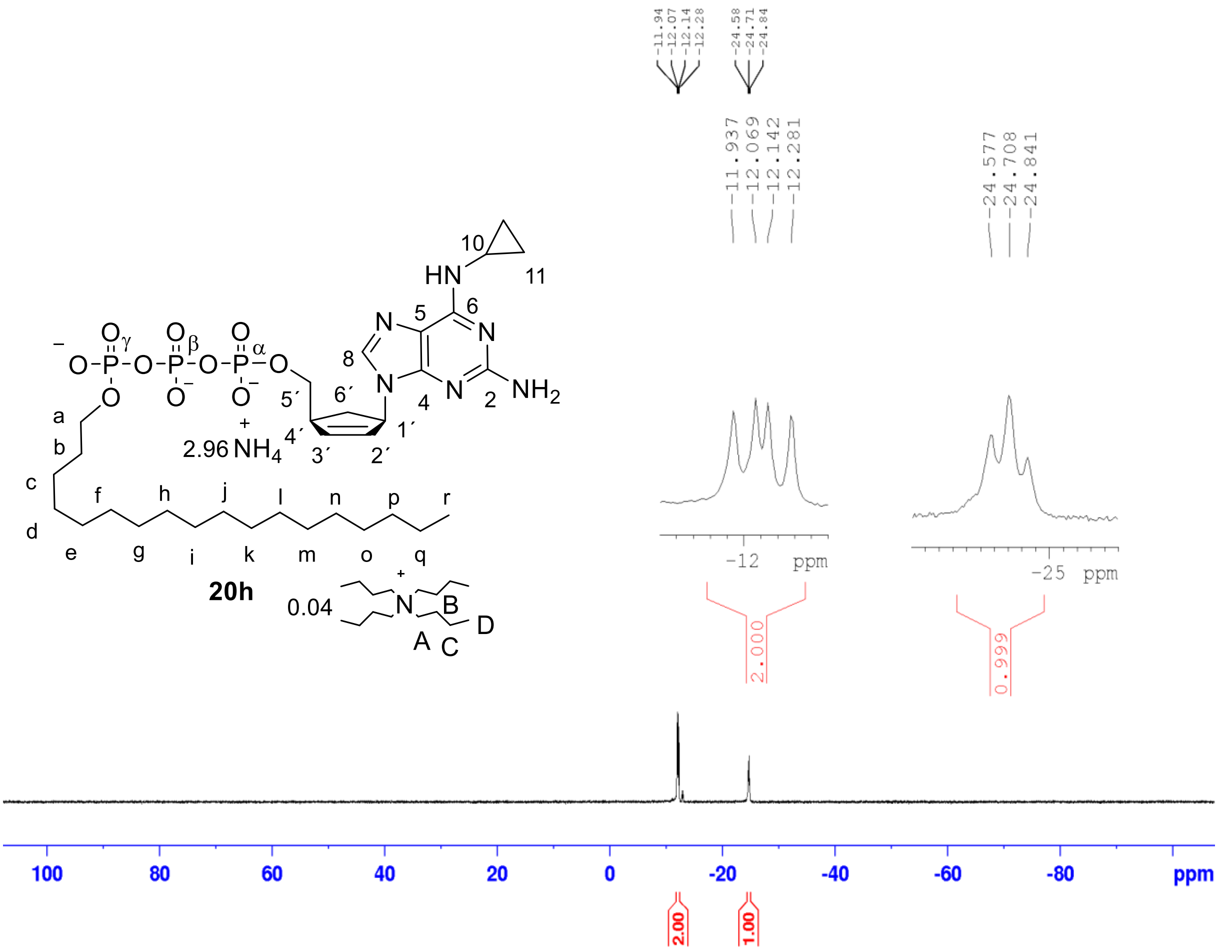
20h
2.96 NH4
0.04
ppm
2.000
0.999
2.00
1.00

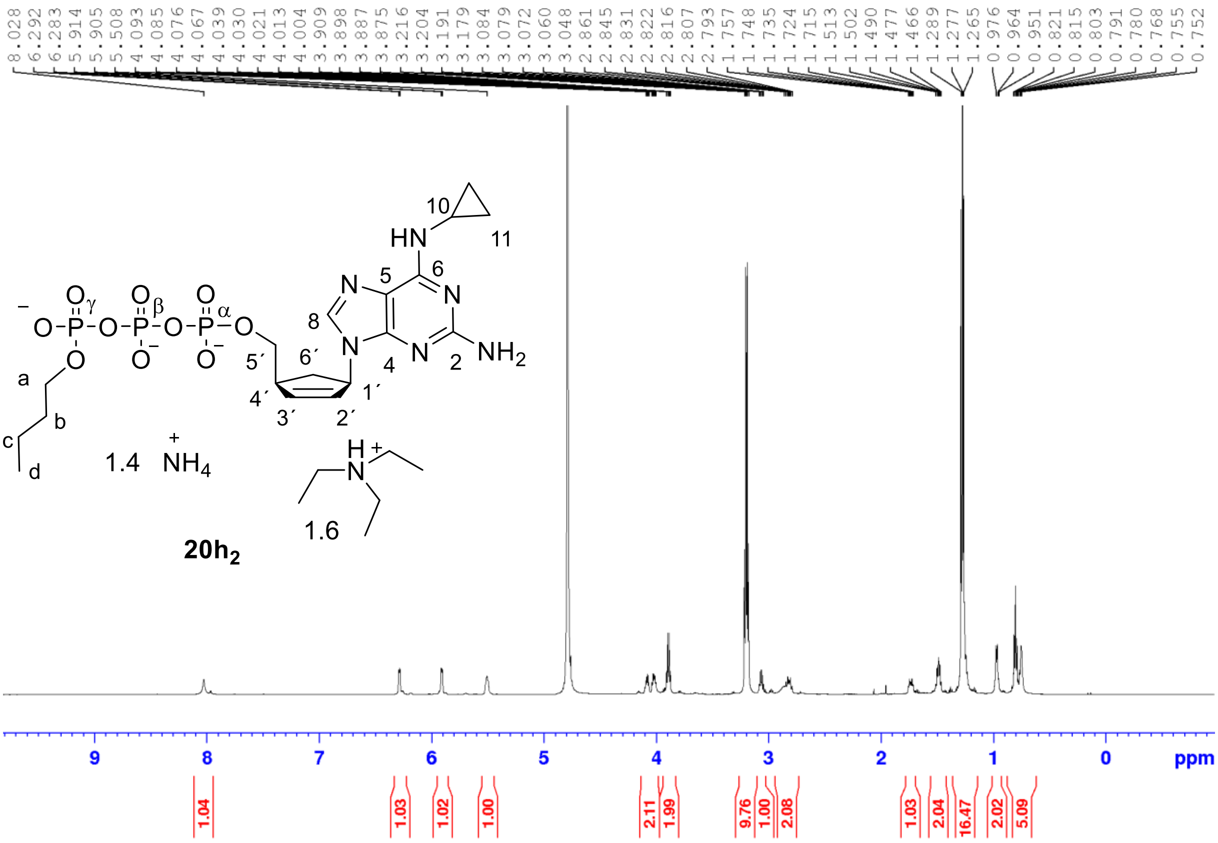
20h2
1.4 NH4
1.6
ppm
1.04
1.03
1.02
1.00
2.11
1.99
9.76
1.00
2.08
1.03
2.04
16.47
2.02
5.09

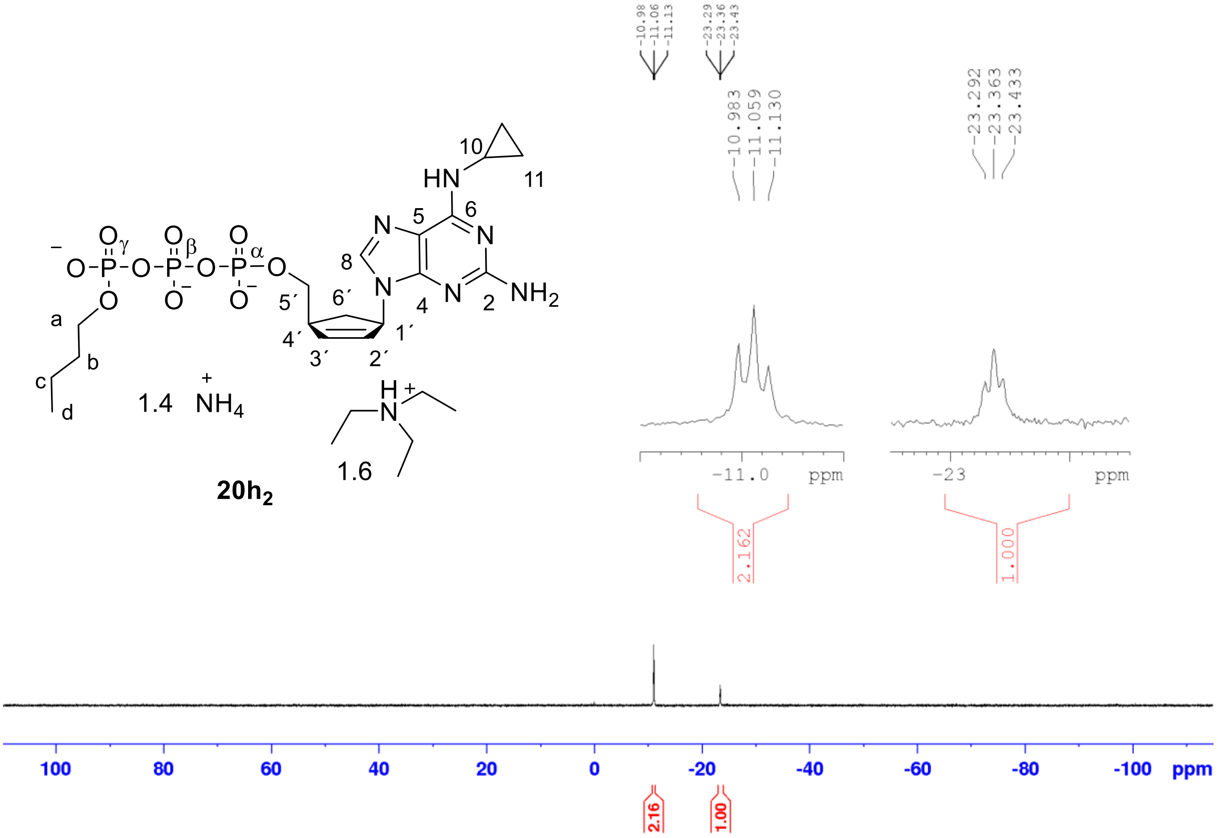
-10.98
-11.06
-11.13
-23.29
-23.36
-23.43
-10.983
-11.059
-11.130
-23.292
-23.363
-23.433
-11.0
ppm
-23
ppm
2.162
1.000
20h2
1.4 NH4
1.6
2.16
1.00
100
80
60
40
20
0
-20
-40
-60
-80
-100
ppm

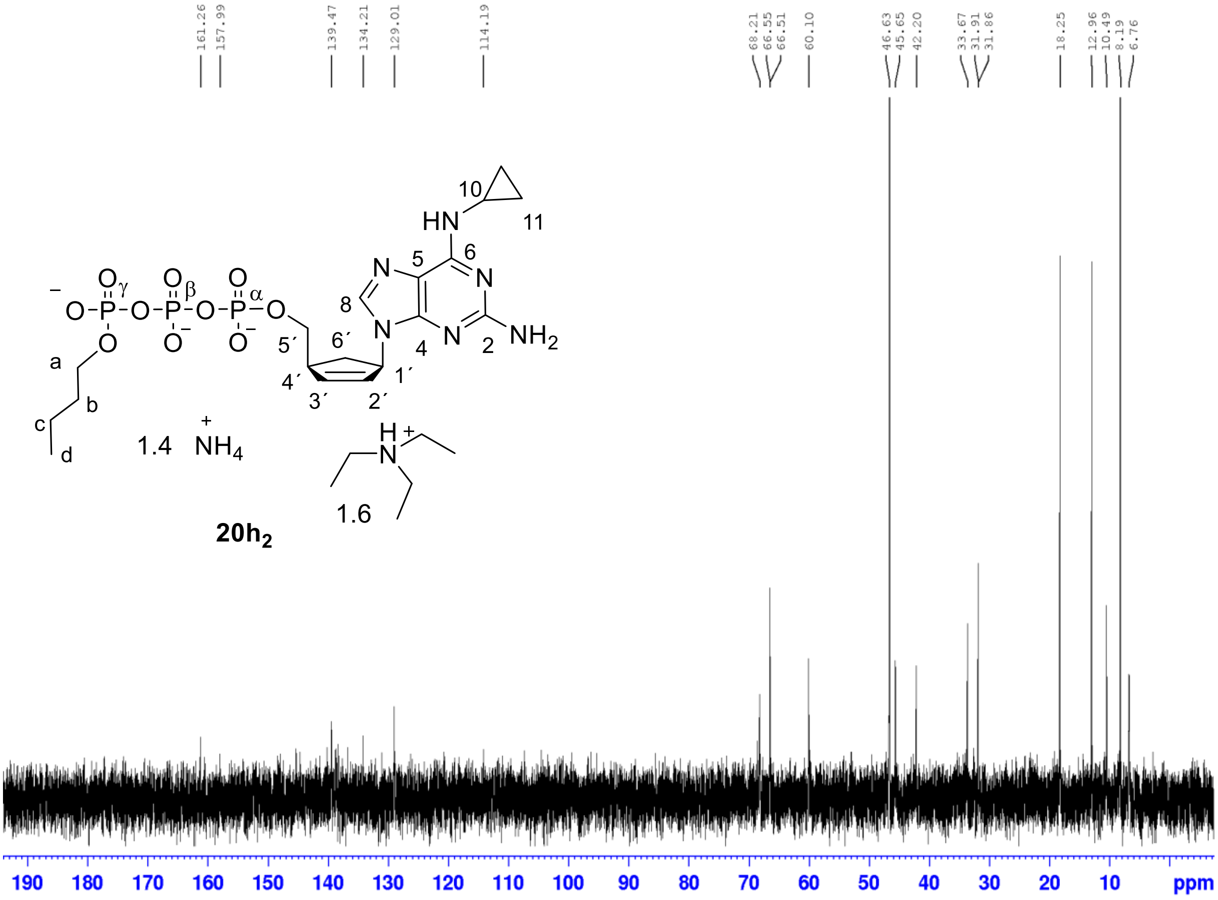
161.26
157.99
139.47
134.21
129.01
114.19
68.21
66.55
66.51
60.10
46.63
45.65
42.20
33.67
31.91
31.86
18.25
12.96
10.49
8.19
6.76
20h2
1.4 NH4
1.6
190
180
170
160
150
140
130
120
110
100
90
80
70
60
50
40
30
20
10
ppm

Reference.

[1] X. Jia, D. Schols, C. Meier, *J. Med. Chem.* **2020**, 63, 6991-7007.

[2] T. Sowa, S. Ouchi, *B. Chem. Soc. Jpn.* **1975**, *48*, 2084-2090.

[3] A. R. Kore, Z. J. Xiao, A. Senthilvelan, I. Charles, M. Shanmugasundaram, S. Mukundarajan, B. Srinivasan, *Nucleosides, Nucleotides Nucleic Acids.* **2012**, *31*, 567-573.

[4] A. R. Kore, M. Shanmugasundaram, A. Senthilvelan, B. Srinivasan, *Nucleosides, Nucleotides Nucleic Acids.* **2012**, *31*, 423-431.

[5] X. Jia, S. Weber, D. Schols, C. Meier, *J. Med. Chem.* **2020**, *63*, 11990-12007.

[6] C. Zhao, S. Weber, D. Schols, J. Balzarini, C. Meier, *Angew. Chem., Int. Ed.* **2020**, *59*, 22063-22071.

[7] T. Nack, T. D. de Oliveira, S. Weber, D. Schols, J. Balzarini, C. Meier, *J. Med. Chem.* **2020**, *63*, 13745-13761.
